# Fully-automated identification of fish species based on otolith contour: using short-time Fourier transform and discriminant analysis (STFT-DA)

**DOI:** 10.7717/peerj.1664

**Published:** 2016-02-22

**Authors:** Nima Salimi, Kar Hoe Loh, Sarinder Kaur Dhillon, Ving Ching Chong

**Affiliations:** 1 Institute of Biological Sciences, University of Malaya, Kuala Lumpur, Malaysia; 2 Institute of Ocean & Earth Sciences, University of Malaya, Kuala Lumpur, Malaysia

**Keywords:** Automated taxon identification, Otolith shape analysis, Short-time Fourier transform, Discriminant analysis

## Abstract

**Background.** Fish species may be identified based on their unique otolith shape or contour. Several pattern recognition methods have been proposed to classify fish species through morphological features of the otolith contours. However, there has been no fully-automated species identification model with the accuracy higher than 80%. The purpose of the current study is to develop a fully-automated model, based on the otolith contours, to identify the fish species with the high classification accuracy.

**Methods.** Images of the right sagittal otoliths of 14 fish species from three families namely Sciaenidae, Ariidae, and Engraulidae were used to develop the proposed identification model. Short-time Fourier transform (STFT) was used, for the first time in the area of otolith shape analysis, to extract important features of the otolith contours. Discriminant Analysis (DA), as a classification technique, was used to train and test the model based on the extracted features.

**Results.** Performance of the model was demonstrated using species from three families separately, as well as all species combined. Overall classification accuracy of the model was greater than 90% for all cases. In addition, effects of STFT variables on the performance of the identification model were explored in this study.

**Conclusions.** Short-time Fourier transform could determine important features of the otolith outlines. The fully-automated model proposed in this study (STFT-DA) could predict species of an unknown specimen with acceptable identification accuracy. The model codes can be accessed at http://mybiodiversityontologies.um.edu.my/Otolith/ and https://peerj.com/preprints/1517/. The current model has flexibility to be used for more species and families in future studies.

## Introduction

Automated taxon identification (ATI) systems which rely on pattern recognition and machine learning techniques have been developed in different areas of biology (Arbuckle et al., 2001; Chun et al., 2007; Cope et al., 2012; Culverhouse et al., 1996; Dietrich & Pooley, 1994; Farr & Chesmore, 2007; Gaston & O’Neill, 2004; Jonker et al., 2000; La Salle et al., 2009; Larios et al., 2008; MacLeod, Benfield & Culverhouse, 2010; Parisi-Baradad et al., 2010; Potamitis, 2014; Watson, O’Neill & Kitching, 2003; Watson & Dallwitz, 1991; Zhao et al., 2013). In marine biology, identification of the fish species based on the otolith image analysis has been an interesting area due to its applications in the palaeontological and ecological sciences (Aguirre & Lombarte, 1999; Arellano et al., 1995; Bowen, 2000; Fitch & Brownell Jr, 1968; Lombarte & Castellón, 1991; Reichenbacher et al., 2007). Parisi-Baradad et al. (2010) developed the first automated taxon classification system through the shape analysis of the otolith contour. In order to extract the important morphological features of the otolith contour, external outline of the otolith was first converted to a one-dimensional (1D) signal. This representative signal was obtained by calculating the distances between the outline points and the center of gravity of the otolith image. Then, wavelet transform (WT) was applied on the 1D signal to extract useful features of the otolith outline. Using WT, irregularities of the otolith contours were quantified and localized appropriately; this is the advantage of WT over other feature extractors such as Fourier transform (FT) and elliptical Fourier descriptors (EFD) used in the other studies (Parisi-Baradad et al., 2005; Sadighzadeh et al., 2012). Even though their proposed model could identify the family of the specimens with 94% accuracy, the performance of the system dropped significantly at the species level (72%) (Parisi-Baradad et al., 2010). Therefore, the aim of the present study is to develop a fully-automated identification model with improved classification accuracy at the level of species.

Fourteen fish species from three different families namely Engraulidae, Sciaenidae, and Ariidae were used in this study. Short-time Fourier transform (STFT) is a conventional signal processing technique (Allen, 1997; Oppenheim, Schafer & Buck, 1999; Rabiner & Schafer, 1978) which to our knowledge has not yet been employed in the area of otolith image processing. STFT was applied in this study to extract morphological features of the otolith contours.

## Materials and Methods

**Figure 1:**
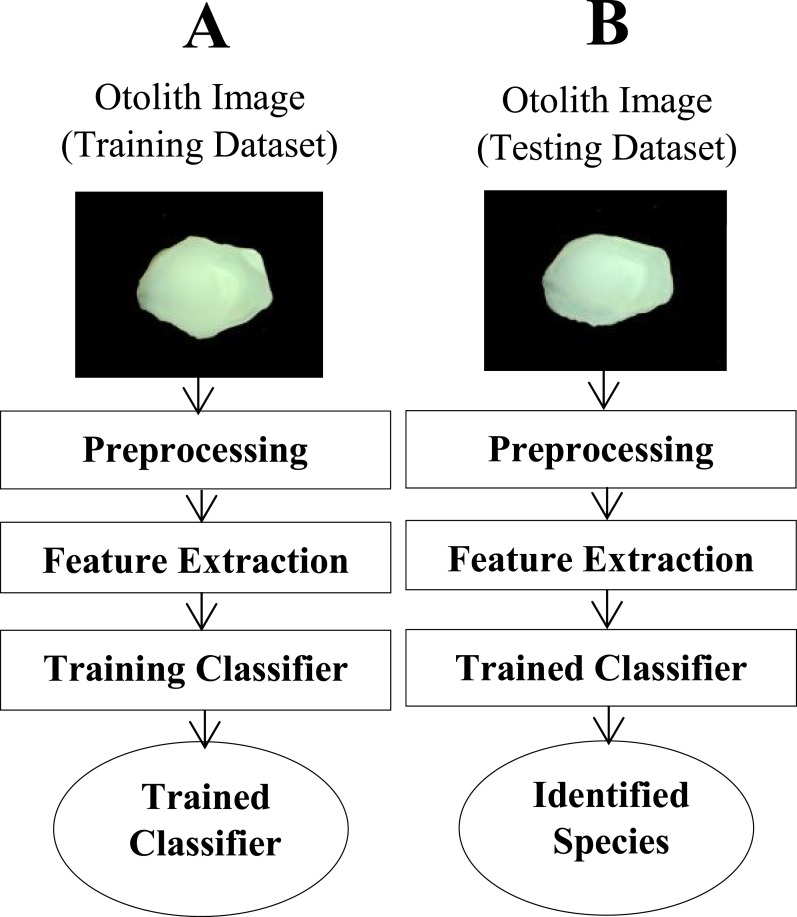
A schematic diagram of the proposed image identification system. (A) shows different stages for training the model, and the testing part of the system is illustrated in the (B).

Images of the right sagittal otoliths were captured using a stereomicroscope (Olympus DP25FW, 6.3X magnification) attached with a digital camera. Proximal view of the otolith, dorsal edge facing up and posterior end facing the positive direction, was used in this study. The proposed image identification system was implemented in MATLAB (MATLAB^®^ Release 2013a, The MathWorks, Inc., Kuala Lumpur, Malaysia). Figure 1 illustrates the schematic diagram of the fully-automated image recognition model represented in this study. Different stages of this system are detailed as follows.

### Preprocessing

**Figure 2:**
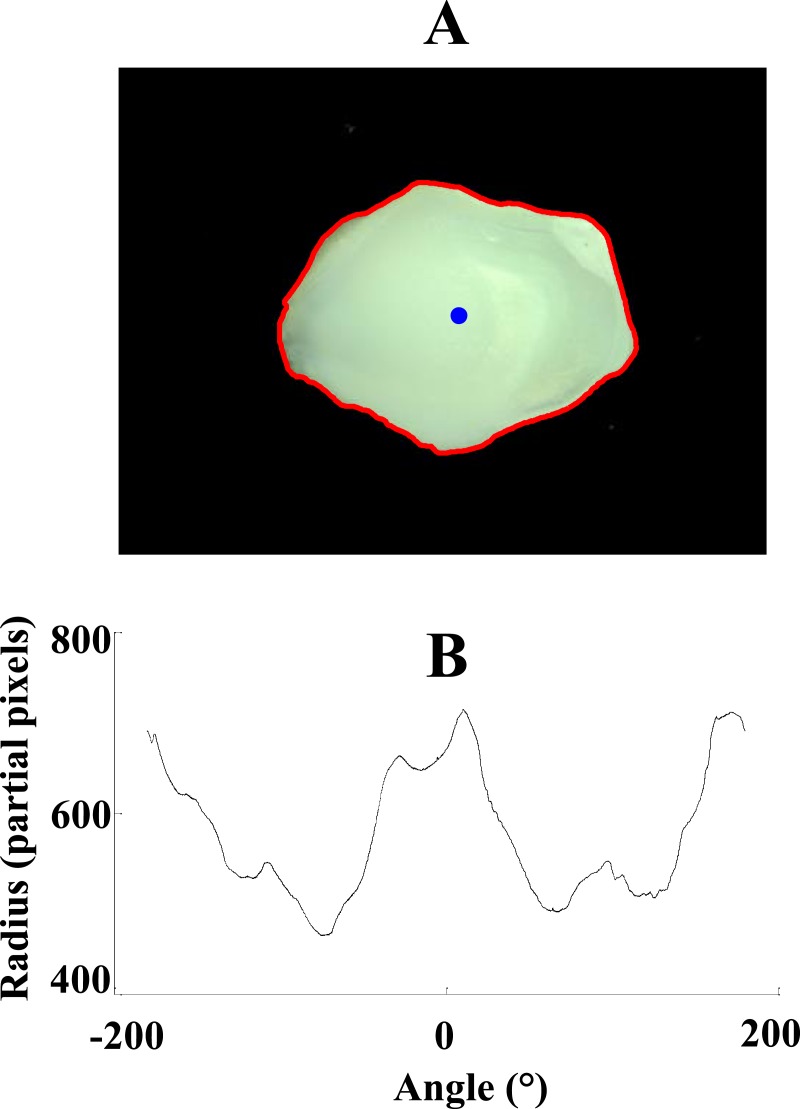
Image of an otolith (A) with its corresponding 1D signal (B). 1D signal was obtained by calculating the radius, distances between the boundary pixels (red) and the center of gravity (blue), as a function of angle.

Discrimination among different fish species was based on the 1D representation of the otolith outline. Firstly, the external outline of the surface contours of the otolith had to be extracted and then, distances between the center of gravity and the contour points had to be calculated. For this purpose, the grayscale image of the otolith was converted to the binary image with the threshold value of 0.1. Choice of this threshold value (0.1) resulted in obtaining the binary images for the otoliths with a wide range of transparency. After clearing the borders and filling the holes, the small objects (objects that had fewer than 50,000 pixels) were removed from the binary images. Then, coordinates of the boundary (outline) pixels as well as the center of gravity were calculated. By having these coordinates, characteristic 1D signals, which are the distances between the boundary pixels and center of gravity as a function of the corresponding angles, were determined. Figure 2 shows an image of the otolith with its representative 1D signal.

### Feature extraction

**Figure 3:**
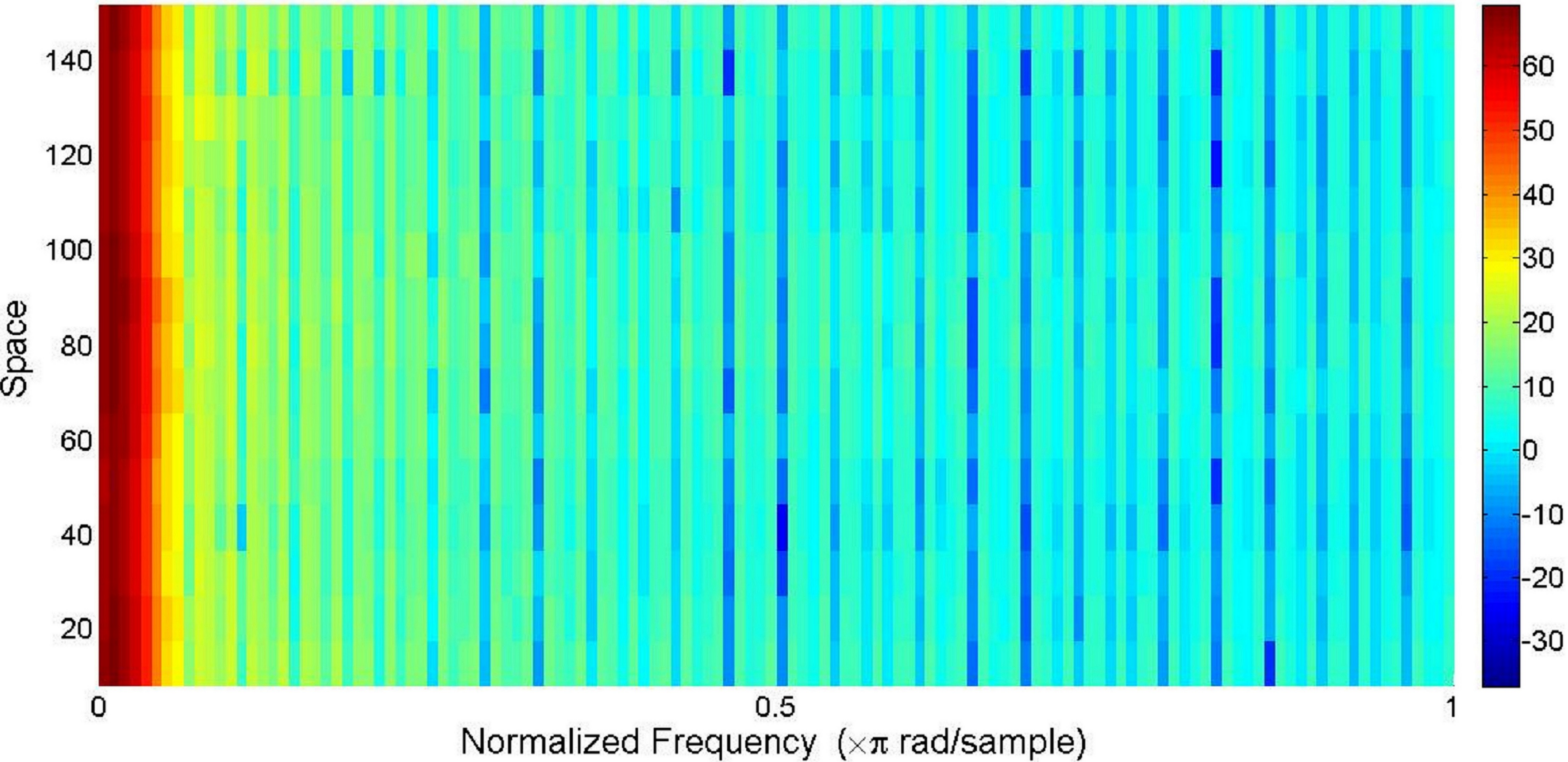
The spectrogram of the characteristic signal shown in Fig. 2. The original signal was resampled to 1,000 points before calculating the short-time Fourier transform (STFT). The color bar indicates estimates of the power spectral density (PSD). STFT of the spatial-domain signal was calculated with sampling frequency of 2*π*.

1D spatial-domain signals obtained from the previous stage were down-sampled to 1,000 points (samples) by interpolation using fast Fourier transform (FFT). In this study, short-time Fourier transform (STFT) was applied as a feature extraction method on the resampled signals. STFT of the original (1D) signals were determined by using Gaussian window function. Repeated trials of many combinations of two parameters, the number of points of the window function and the number of overlapped samples, were made to achieve the best classification result. The best match of 100 points of the window and 40 overlapped samples resulted in the division of each signal into 16 segments. The type of windowing function also affected the performance of the identification system. To explore this effect, results obtained using different windowing techniques were compared in the next section. Figure 3 shows the spectrogram (using STFT with the sampling frequency (*f*_*s*_) of 2*π*) obtained from the 1D spatial-domain signal illustrated in Fig. 2. The color bar in Fig. 3 indicates the power spectral density (PSD) estimate of each segment. Each segment of the original signal consisted of 129 frequency components. Absolute values and phase angles of the frequency components of each segment were determined and then standardized by calculating the corresponding *z*-scores (Z_ABSs: *z*-scores of the absolute values and Z_ANGs: *z*-scores of the angles). In each segment of the signal, two important parameters were determined: maximum of the Z_ABSs (MAX_ABS_) and maximum of Z_ANGs (MAX_ANG_). Having 16 segments in each signal, 32 attributes (16 MAX_ABS_ + 16 MAX_ANG_) were extracted from each representative signal. In this way, each otolith image was converted to a 32-element vector in which the first 16 elements were MAX_ABS_ values and the rest were the values of MAX_ANG_. The contribution of each feature type (absolute and angle) to the performance of the model was also explored and the obtained results are demonstrated in the next section.

### Classification

The characteristic vectors obtained from the previous stage were utilized as inputs to the Discriminant Analysis (DA) classifier in order to train and test the identification system. Fourteen species from three different families were used in this study (Table 1). All otoliths were extracted from fish obtained from fish landing sites or the wet markets. No ethics clearance was required from the University of Malaya—Institutional Animal Care and Use Committee (UM-IACUC).

**Table 1:** Fish species used in the proposed fully-automated identification system.

Species	Family
*Dendrophysa russelli*	Sciaenidae
*Johnius belangerii*	”
*Johnius carouna*	”
*Otolithes ruber*	”
*Panna microdon*	”
*Nemapteryx caelata*	Ariidae
*Arius maculatus*	”
*Cryptarius truncatus*	”
*Hexanematichtys sagor*	”
*Osteogeneiosus militaris*	”
*Plicofollis argyropleuron*	”
*Coilia dussumieri*	Engraulidae
*Setipinna taty*	”
*Thryssa hamiltonii*	”

## Results

Three different fish families (Sciaenidae, Ariidae, and Engraulidae) were used separately to train and test the model. In addition, the proposed image identification model was evaluated for all 14 species combined.

### Engraulidae family

Three species namely *Coilia dussumieri*, *Setipinna taty* and *Thryssa hamiltonii* from the Engraulidae family were used in this study. From each species, 20 specimens (otolith images) were used for training the model. Then, the trained model was tested with 10 specimens per species (total of 30 images for testing the model). Table 2 demonstrates the confusion matrix obtained from the predicted species in this family.

**Table 2:** Confusion matrix for the classification results of the Engraulidae family. The predicted species (columns) are compared with the species confirmed by an expert (rows).

	*Coilia dussumieri*	*Setipinna taty*	*Thryssa hamiltonii*
*Coilia dussumieri*	**10** **(100%)**	0 (0%)	0 (0%)
*Setipinna taty*	0 (0%)	**10** **(100%)**	0 (0%)
*Thryssa hamiltonii*	0 (0%)	1 (10%)	**9** **(90%)**

All of the 10 specimens from the *Coilia dussumieri* and *Setipinna taty* species were classified correctly. For the *Thryssa hamiltonii* species, one specimen was misclassified as the *Setipinna taty* species. Overall, 29 out of 30 specimens from the Engraulidae family (∼97%) were correctly predicted as the target species.

### Sciaenidae family

Five species of the Sciaenidae family were also used to evaluate performance of the identification system. In this family, 19 specimens per species (total number of 95 specimens) were used to train the system, and then the trained model was tested with 50 specimens (10 specimens per species). The predicted results of this family are presented in Table 3. Among five species in this family, three species (*Johnius belangerii*, *Johnius carouna* and *Panna microdon*) were identified with 100% accuracy. Two other species (*Dendrophysa russelli* and *Otolithes ruber*) had one misclassified specimen each. In this family, similar to the Engraulidae family, there was no species with classification accuracy of less than 90%. The proposed model identified five species of the Sciaenidae family with an overall accuracy of 96%.

**Table 3:** Confusion matrix obtained from five species of the Sciaenidae family. The columns indicate the predicted species by the identification model, while rows indicate the target species.

	*Dendrophysa russelli*	*Johnius belangerii*	*Johnius carouna*	*Otolithes ruber*	*Panna microdon*
*Dendrophysa russelli*	**9** **(90%)**	0 (0%)	0 (0%)	1 (10%)	0 (0%)
*Johnius belangerii*	0 (0%)	**10** **(100%)**	0 (0%)	0 (0%)	0 (0%)
*Johnius carouna*	0 (0%)	0 (0%)	**10** **(100%)**	0 (0%)	0 (0%)
*Otolithes ruber*	1 (10%)	0 (0%)	0 (0%)	**9** **(90%)**	0 (0%)
*Panna microdon*	0 (0%)	0 (0%)	0 (0%)	0 (0%)	**10** **(100%)**

### Ariidae family

Six species from the Ariidae family were also used in this study. The number of specimens per species for training and testing the model were 18 and 10, respectively. The classification results obtained from this family are shown in Table 4. Overall accuracy of the model in this family was ∼93% which is slightly less than the other two families. The lowest classification accuracy (80%) in this family was for the *Nemapteryx caelatus*. Two specimens of the *Nemapteryx caelatus species* were predicted as the *Cryptarius truncatus.* Three species namely *Arius maculatus*, *Hexanematichtys sagor* and *Plicofollis argyropleuron* had 100% correct prediction results. The accuracy of the model for the *Cryptarius truncatus* and *Osteogeneiosus militaris* species was 90%. Both of these species had one specimen that was misclassified as *Nemapteryx caelatus*.

**Table 4:** Classification results (confusion matrix) of the Ariidae family. Outputs of the identification model (columns) are compared with the target species (rows).

	*Nemapteryx caelatus*	*Arius maculatus*	*Cryptarius truncatus*	*Hexanematichtys sagor*	*Osteogeneiosus militaris*	*Plicofollis argyropleuron*
*Nemapteryx caelatus*	**8** **(80%)**	0 (0%)	2 (20%)	0 (0%)	0 (0%)	0 (0%)
*Arius maculatus*	0 (0%)	**10** **(100%)**	0 (0%)	0 (0%)	0 (0%)	0 (0%)
*Cryptarius truncatus*	1 (10%)	0 (0%)	**9** **(90%)**	0 (0%)	0 (0%)	0 (0%)
*Hexanematichtys sagor*	0 (0%)	0 (0%)	0 (0%)	**10** **(100%)**	0 (0%)	0 (0%)
*Osteogeneiosus militaris*	1 (10%)	0 (0%)	0 (0%)	0 (0%)	**9** **(90%)**	0 (0%)
*Plicofollis argyropleuron*	0 (0%)	0 (0%)	0 (0%)	0 (0%)	0 (0%)	**10** **(100%)**

### All three families

To test the model with more species, all three families were combined (total number of 14 species) and the results of the classification are demonstrated in Table 5. From each species, 18 and 10 specimens were used to train and test the model, respectively (total numbers of 252 images for the training and 140 images for the testing). All 14 species were predicted by the proposed model with an overall accuracy of ∾92%. Eight of these species, three from the Sciaenidae, three from the Ariidae, and two from the Engraulidae family, were classified with the accuracy of 100%. Three species showed the identification accuracy of less than 90% (*Dendrophysa russelli*: 80%, *Nemapteryx caelatus*: 70%, and *Cryptarius truncatus*: 70%). *Nemapteryx caelatus* and *Cryptarius truncates*, both from the Ariidae family, had the most numbers of misclassified specimens among the 14 species used in this study. The classification accuracy for *Otolithes ruber*, *Osteogeneiosus militaris*, and *Setipinna taty* was 90%. It is worth-noting that there was no cross-family misclassification for all six species that had at least one misclassified specimen (all six species had specimens correctly classified in their families). As a result, developing a model that first identifies the family and then species cannot lead to an improvement in the overall accuracy of the system.

**Table 5:** Confusion matrix for the identification results obtained from 14 species of three different families. In each target species (rows), numbers of specimens are indicated in the corresponding predicted species (columns). Species are *Dendrophysa russelli* (1), *Johnius belangerii* (2), *Johnius carouna* (3), *Otolithes ruber* (4), *Panna microdon* (5), *Nemapteryx caelatus* (6), *Arius maculatus* (7), *Cryptarius truncatus* (8), *Hexanematichtys sagor* (9), *Osteogeneiosus militaris* (10), *Plicofollis argyropleuron* (11), *Coilia dussumieri* (12), *Setipinna taty* (13), *Thryssa hamiltonii* (14).

	1	2	3	4	5	6	7	8	9	10	11	12	13	14
1	**8**	0	0	2	0	0	0	0	0	0	0	0	0	0
2	0	**10**	0	0	0	0	0	0	0	0	0	0	0	0
3	0	0	**10**	0	0	0	0	0	0	0	0	0	0	0
4	1	0	0	**9**	0	0	0	0	0	0	0	0	0	0
5	0	0	0	0	**10**	0	0	0	0	0	0	0	0	0
6	0	0	0	0	0	**7**	0	2	0	0	1	0	0	0
7	0	0	0	0	0	0	**10**	0	0	0	0	0	0	0
8	0	0	0	0	0	3	0	**7**	0	0	0	0	0	0
9	0	0	0	0	0	0	0	0	**10**	0	0	0	0	0
10	0	0	0	0	0	0	0	1	0	**9**	0	0	0	0
11	0	0	0	0	0	0	0	0	0	0	**10**	0	0	0
12	0	0	0	0	0	0	0	0	0	0	0	**10**	0	0
13	0	0	0	0	0	0	0	0	0	0	0	0	**9**	1
14	0	0	0	0	0	0	0	0	0	0	0	0	0	**10**

### Contribution of MAX_ABS_ and MAX_ANG_ to the system performance

To explore the contribution of MAX_ABS_ and MAX_ANG_ to the performance of the system, each feature type was separately used to train and test the model. Table 6 shows the classification results obtained by using each feature type separately (16-element vector) and their combined features (32-element vector). For all four data sets used in this study, the best identification result was achieved using the 32-element vector. For the Engraulidae, Sciaenidae and the combined families, using only the MAX_ANG_ resulted in higher accuracy compared to using only the MAX_ABS_. However, the performance of the model was better for the Ariidae family by using the 16-element vector obtained from the absolute features (MAX_ABS_). This result suggests that both phase and absolute features should be taken into account when the model is trained with different fish families.

**Table 6:** Performance of the model using absolute, phase angle, and combined features (rows) for all four data sets (columns) used in this study.

Extracted features	Overall accuracy			
	Engraulidae family	Sciaenidae family	Ariidae family	All families
16 MAX_ABS_	87%	84%	87%	84%
16 MAX_ANG_	93%	94%	78%	86%
16 MAX_ABS_ + 16MAX_ANG_	**97%**	**96%**	**93%**	**92%**

**Table 7:** Classification results of the model for 16 different window functions. Using each window function (rows), the model performance was calculated for all four datasets (columns).

Window functions	Overall accuracy			
	Engraulidae family	Sciaenidae family	Ariidae family	All families
Bartlett-Hann	87%	82%	85%	83%
Bartlett	90%	82%	90%	85%
Blackman	60%	80%	88%	77%
Blackman-Harris	50%	80%	85%	62%
Bohman	53%	82%	87%	63%
Chebyshev	47%	78%	78%	69%
Flat top	40%	68%	80%	64%
Gaussian	**97%**	**96%**	**93%**	92%
Hamming	93%	**96%**	92%	92%
Hann	70%	88%	88%	84%
Kaiser	90%	**96%**	82%	93%
Nuttall’s	57%	84%	85%	68%
Parzen	50%	68%	90%	66%
Rectangular	87%	**96%**	83%	**94%**
Tapered cosine	93%	88%	87%	89%
Triangular	57%	84%	90%	84%

### Effect of the windowing function

As mentioned in the previous section, the windowing function used to calculate STFT of the representative signals could influence the performance of the model. To explore this effect, the identification system was trained and tested with several types of the window function. However, the number of points of window (100) and the number of overlapped points (40) were fixed for all types of window function tested. The overall accuracy obtained from three families, as well as the combined families, are compared and shown in Table 7.

Using the Gaussian window function led to the highest classification accuracy (97%) in the Engraulidae family. In the Sciaenidae family, the best result (96%) was achieved by using four functions namely Gaussian, Hamming, Kaiser, and Rectangular. The most accurate prediction (93%) in the Ariidae family was obtained by using the Gaussian function. In the combined families, using the Rectangular function resulted in the highest overall accuracy (94%). However, utilizing the Rectangular windowing function led to relatively poor performance of the model in the Engraulidae (87%) and Ariidae (83%) families. Taking into accounts all the results obtained using these 16 functions, the Gaussian window function was selected in this study due to its good performance in all the four data sets.

## Discussion

The identification model proposed in this study could predict the species of an unknown specimen from the Engraulidae, Sciaenidae, and Ariidae family with the overall accuracy of 97%, 96%, and 93%, respectively. Even after combining three families the accuracy of the model remained above 90% (∼92%), which is noticeably higher than the results obtained by the identification model proposed in the most related study (∼72%) (Parisi-Baradad et al., 2010). It is noted that training datasets used in the present study were relatively small (19, 20, and 18 specimens per species for Sciaenidae, Engraulidae, and Ariidae family, respectively). Using more samples in the training sets could lead to increasing the accuracy of the model.

Two spectral analysis methods namely Fourier transform (FT) and wavelet transform (WT) have been applied in the previous studies as the feature extractors (Castonguay, Simard & Gagnon, 1991; Parisi-Baradad et al., 2005; Parisi-Baradad et al., 2010). Short-time Fourier transform (STFT) has been utilized in the present study, for the first time in the area of otolith image recognition, to extract the spectral features of the 1D signal obtained from the fish otolith contour. By using the maximum (standardized) values of the absolutes and phase angles of the STFT-transformed signal, a relatively low number of features (32) was extracted which is desired for the classification systems applying machine learning techniques. On the other hand, multiscale decomposition of the 1D signal using wavelet transform (WT) as proposed by Parisi-Baradad et al. (2005) and Parisi-Baradad et al. (2010) resulted in the extraction of a large number of attributes.

As was demonstrated in the previous section (Table 7), the choice of window function had a direct effect on the performance of the system. In addition to the type of windowing function, the number of points of the window function and the number of overlapped samples played important roles in the classification results. The proposed model was also tested with a variety of these two parameters (not reported here), and the best match was selected (i.e., 100 window points and 40 overlapped samples). Each 1D signal was broken into 16 segments by setting these two parameters to the optimized values. These two parameters were however optimized for the Gaussian window only. The performance of other window types (see Table 7) may be increased by changing the values of these two parameters (i.e., changing the number of segments/spatial resolution).

In this study, only proximal view of the otolith image was used to develop the identification model. However, adding other views (e.g., anterior, dorsal) could lead to improving the performance of the model. Adding other views would be more crucial when other families and species are added to the system. The same procedure, as used for the proximal view, can be applied on the other views of the otolith image. However, other types of the window function, probably with different spatial resolutions, could be more effective in analyzing the other views. In that case, a characteristic vector can be extracted from each view of the otolith. Consequently, each specimen can be represented by a combination of up to six vectors (depending on the number of views), rather than only one vector corresponding to the proximal view. By this way, more important morphological features could be extracted from the otolith contour.

Two classification techniques namely Decision Tree and Discriminant Analysis were tested in this study (the results obtained by the Decision Tree are not shown here) and the latter was selected due to more accurate results. However, there are other classification methods such as Naive Bayes, Nearest Neighbors, Support Vector Machine, and Neural Network which may improve the performance of the model in future studies.

Comparison among existing otolith classification models, in terms of correct species identification, is a difficult task since these models are developed based on different datasets (different fish species obtained from different geographical regions). For instance, the AFORO database of fish otoliths, as used by Parisi-Baradad et al. (2010), consisted of 420 species from mainly western Mediterranean and Antarctic waters (Lombarte et al., 2006; Parisi-Baradad et al., 2010). However, the performance of the automated taxon identification (ATI) model of Parisi-Baradad et al. (2010) was evaluated based on only five species (from five families) in one test, and 50 species (from 35 families) in another test. In both tests, the accuracy of the ATI model at the species level was 72%. The STFT-DA model proposed in the present study classified five species within the same family (Sciaenidae) with an overall accuracy of 96%; while 14 species (from three families) were classified with an overall 92% accuracy. The STFT-DA model could also be tested with species from the AFORO or other databases if the model is trained with sufficient number of samples per species from the corresponding databases. It is expected that the addition of more species to the STFT-DA model would decrease the prediction accuracy. On the other hand, model prediction of species may be improved if the size of the training sets is increased, more outline features as in other views are added, or better classification techniques are used. Moreover, combining the outline features extracted by the STFT-DA model with other morphological attributes such as features of the sulcus acusticus (Tuset, Lombarte & Assis, 2008) may further improve classification accuracy for databases comprising a large number of species. Nonetheless, given the high number of marine fish species in the world, so far over 15,300 species reported by the first Census of Marine Life (ScienceDaily, 2003), a more pragmatic approach would be to limit the number of species by classifying them within a smaller geographical region, water body, country or fish habitat.

## Conclusions

A fully-automated identification system (STFT-DA) has been proposed in this study to classify the fish species based on the morphological characteristics of the otolith outline contour. Fourteen species from three families were used to develop and evaluate performance of the model. Combining the short-time Fourier transform (STFT), as the feature extractor, with the Discriminant Analysis (DA), as the classifier, led to improving the accuracy of the species classification in comparison with the existing automated model. The STFT windowing as well as classification technique had significant effects on the performance of the model. Future enhancements of the proposed model may be needed to include more species into the system.

## Supplemental Information

10.7717/peerj.1664/supp-1 Supplemental Information 1 MATLAB scripts and function used for training and testing the model

10.7717/peerj.1664/supp-2 Supplemental Information 2 Otolith images of *Arius maculatus* species (Ariidae family) used to train and test the model

10.7717/peerj.1664/supp-3 Supplemental Information 3 Otolith images of *Cryptarius truncatus* species from Ariidae family

10.7717/peerj.1664/supp-4 Supplemental Information 4 Otolith images of *Hexanematichtys sagor* species from Ariidae family

10.7717/peerj.1664/supp-5 Supplemental Information 5 Otolith images of *Nemapteryx caelata* species from Ariidae family

10.7717/peerj.1664/supp-6 Supplemental Information 6 Otolith images of *Osteogeneiosus militaris* species from Ariidae family

10.7717/peerj.1664/supp-7 Supplemental Information 7 Otolith images of *Plicofollis argyropleuron* species from Ariidae family

10.7717/peerj.1664/supp-8 Supplemental Information 8 Otolith images of *Coilia dussumieri* species from Engraulidae family

10.7717/peerj.1664/supp-9 Supplemental Information 9 Otolith images of *Setipinna taty* species from Engraulidae family

10.7717/peerj.1664/supp-10 Supplemental Information 10 Otolith images of *Thryssa hamiltonii* species from Engraulidae family

10.7717/peerj.1664/supp-11 Supplemental Information 11 Otolith images of *Dendrophysa russelli* species from Sciaenidae family

10.7717/peerj.1664/supp-12 Supplemental Information 12 Otolith images of *Johnius belangerii* species from Sciaenidae family

10.7717/peerj.1664/supp-13 Supplemental Information 13 Otolith images of *Johnius carouna* species from Sciaenidae family

10.7717/peerj.1664/supp-14 Supplemental Information 14 Otolith images of *Otolithes ruber* species from Sciaenidae family

10.7717/peerj.1664/supp-15 Supplemental Information 15 Otolith images of *Panna microdon* species from Sciaenidae family

## Additional Information and Declarations

### Data Availability

The following information was supplied regarding data availability: Data sets (Otolith images) as well as MATLAB codes are available as Supplemental Information.
