# Supplementary figures and images for: Fully-automated identification of fish species based on otolith contour: using short-time Fourier transform and discriminant analysis (STFT-DA) (part 1 of 5)
Source: PeerJ. 2016 Feb 22;4:e1664. doi: 10.7717/peerj.1664 (PMC4768690; doi:10.7717/peerj.1664)

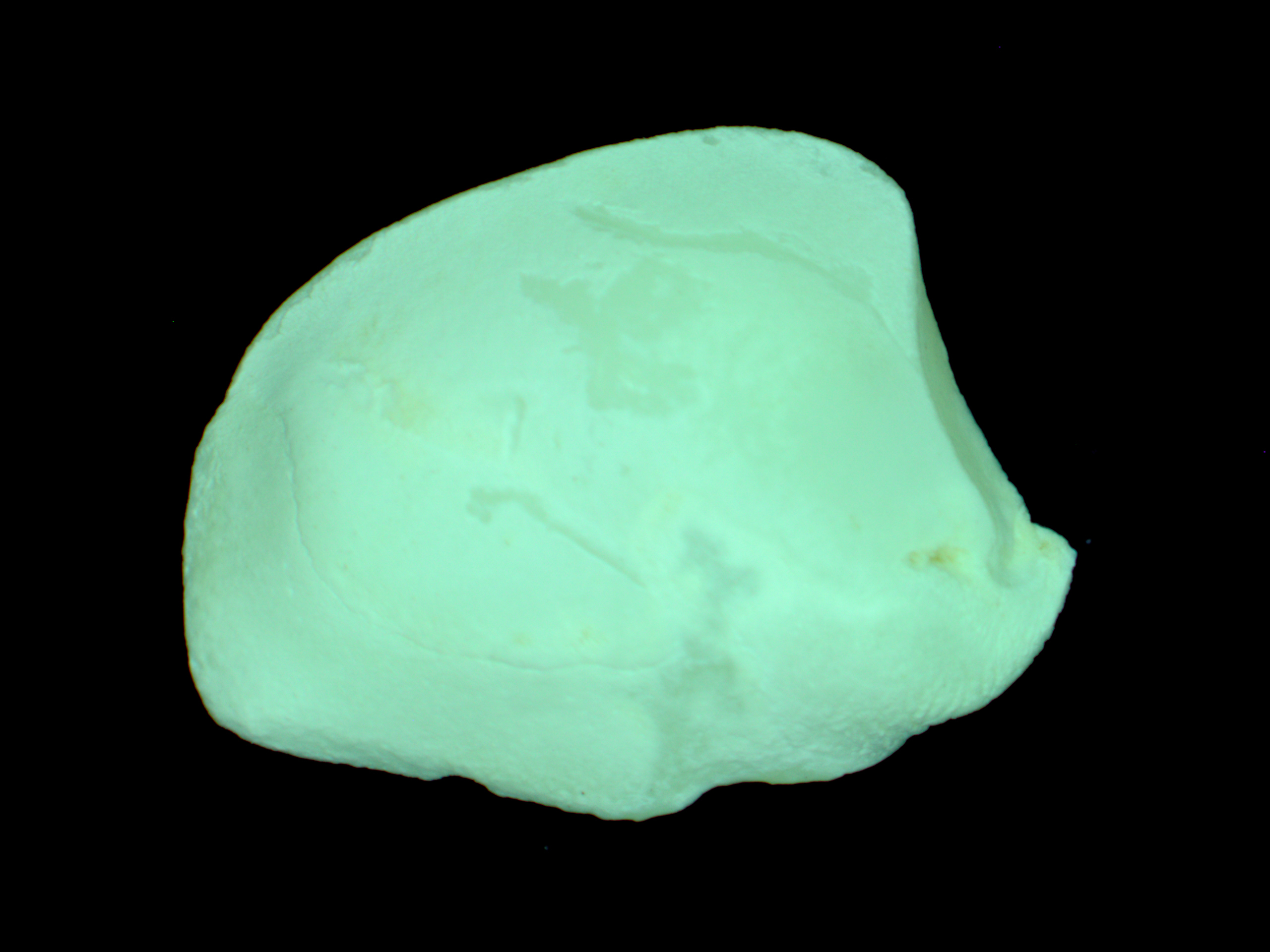

Supplement: Supplemental Information 2 [file peerj-04-1664-s002.zip › AriMac/testing/ARI428_R1.jpg]

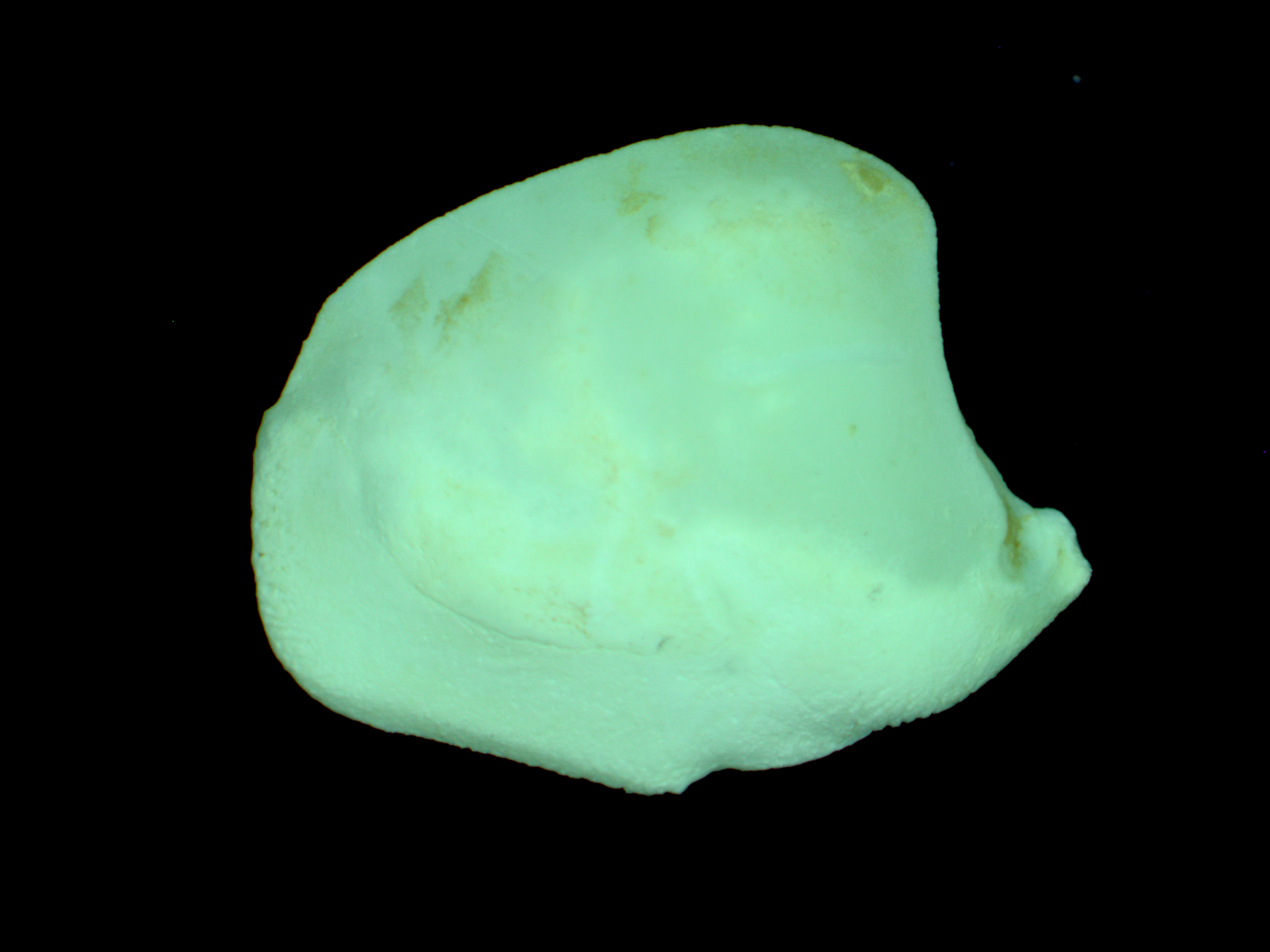

Supplement: Supplemental Information 2 [file peerj-04-1664-s002.zip › AriMac/testing/ARI429_R1.jpg]

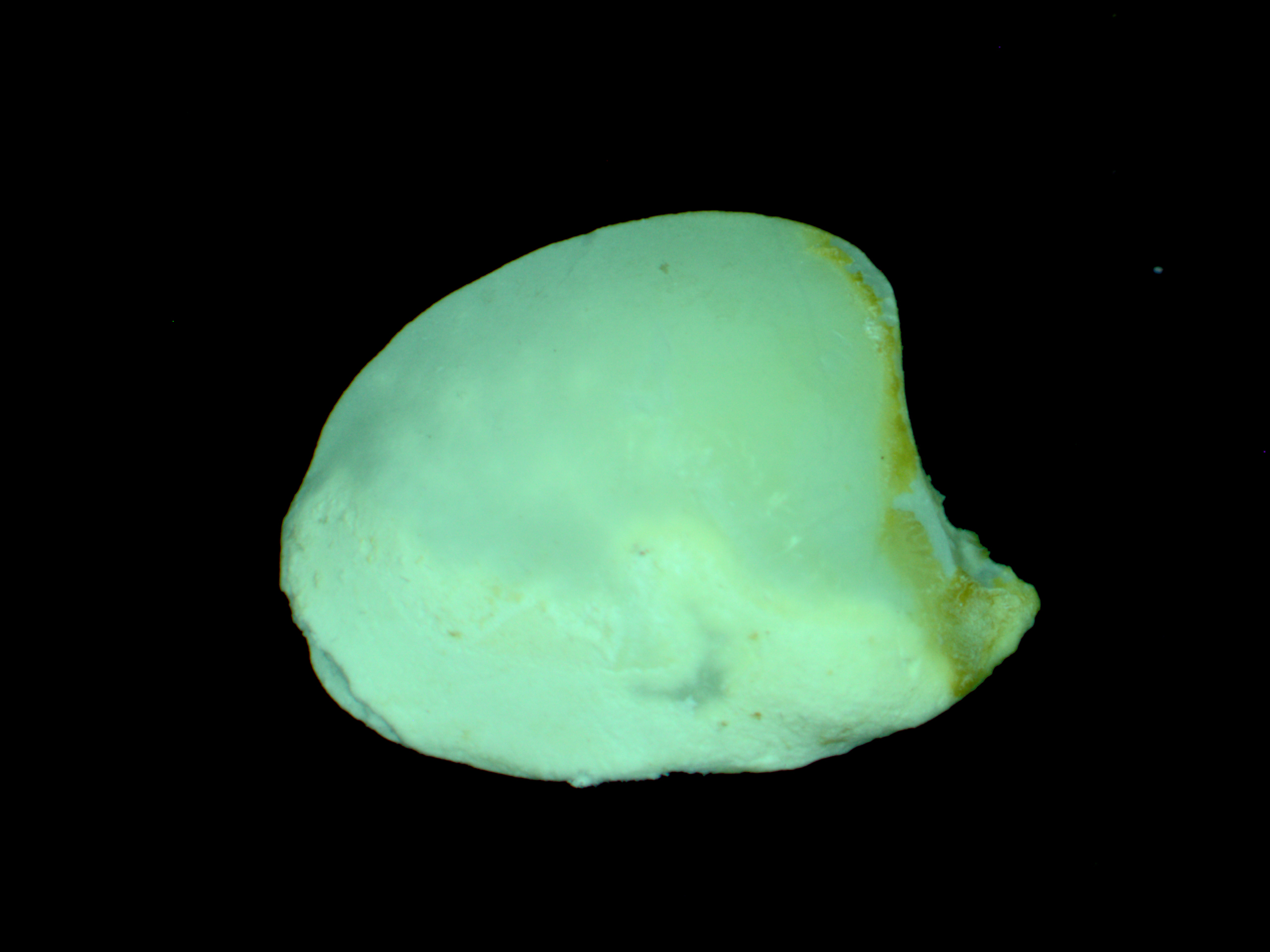

Supplement: Supplemental Information 2 [file peerj-04-1664-s002.zip › AriMac/testing/ARI430_R1.jpg]

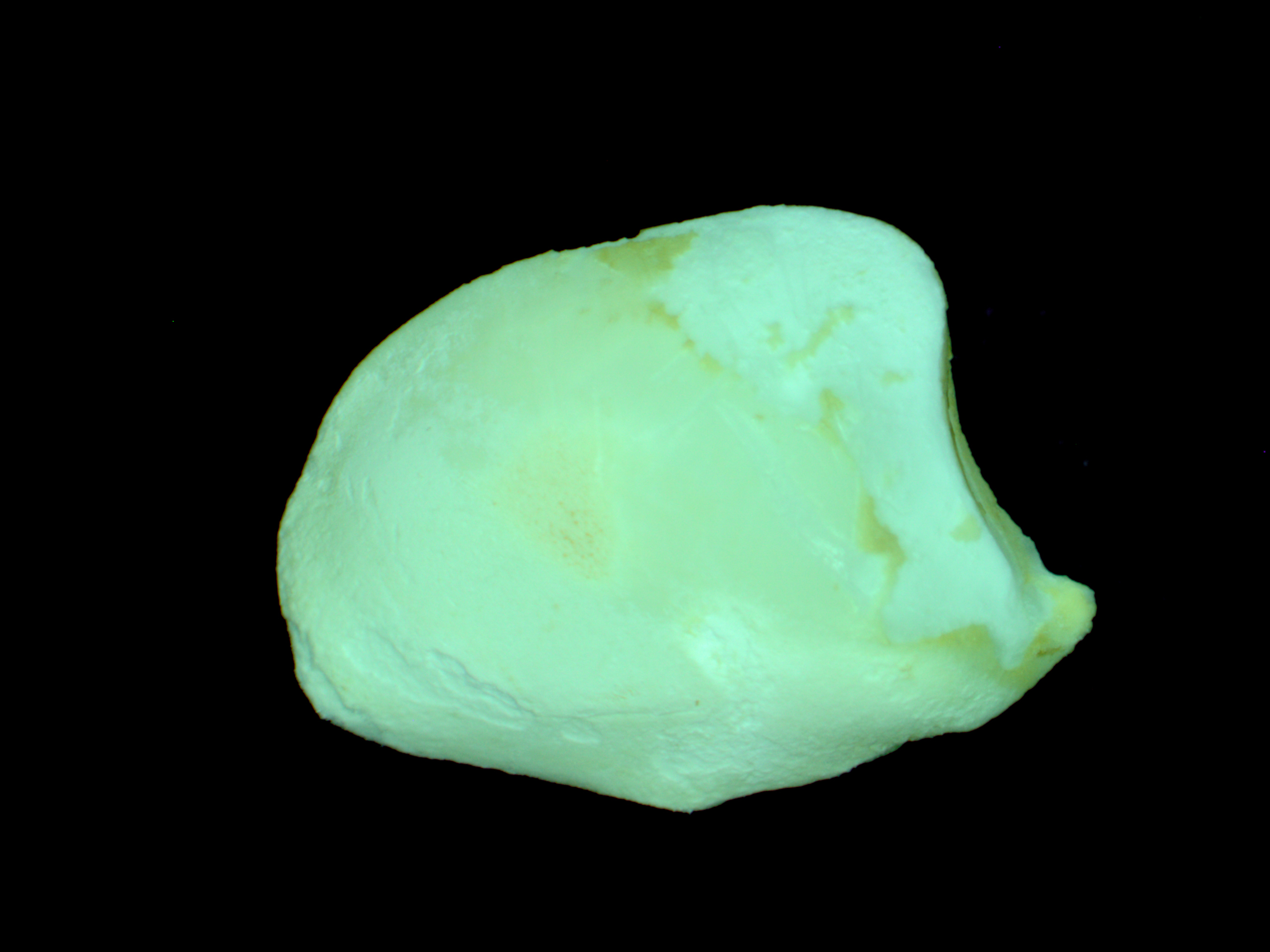

Supplement: Supplemental Information 2 [file peerj-04-1664-s002.zip › AriMac/testing/ARI431_R1.jpg]

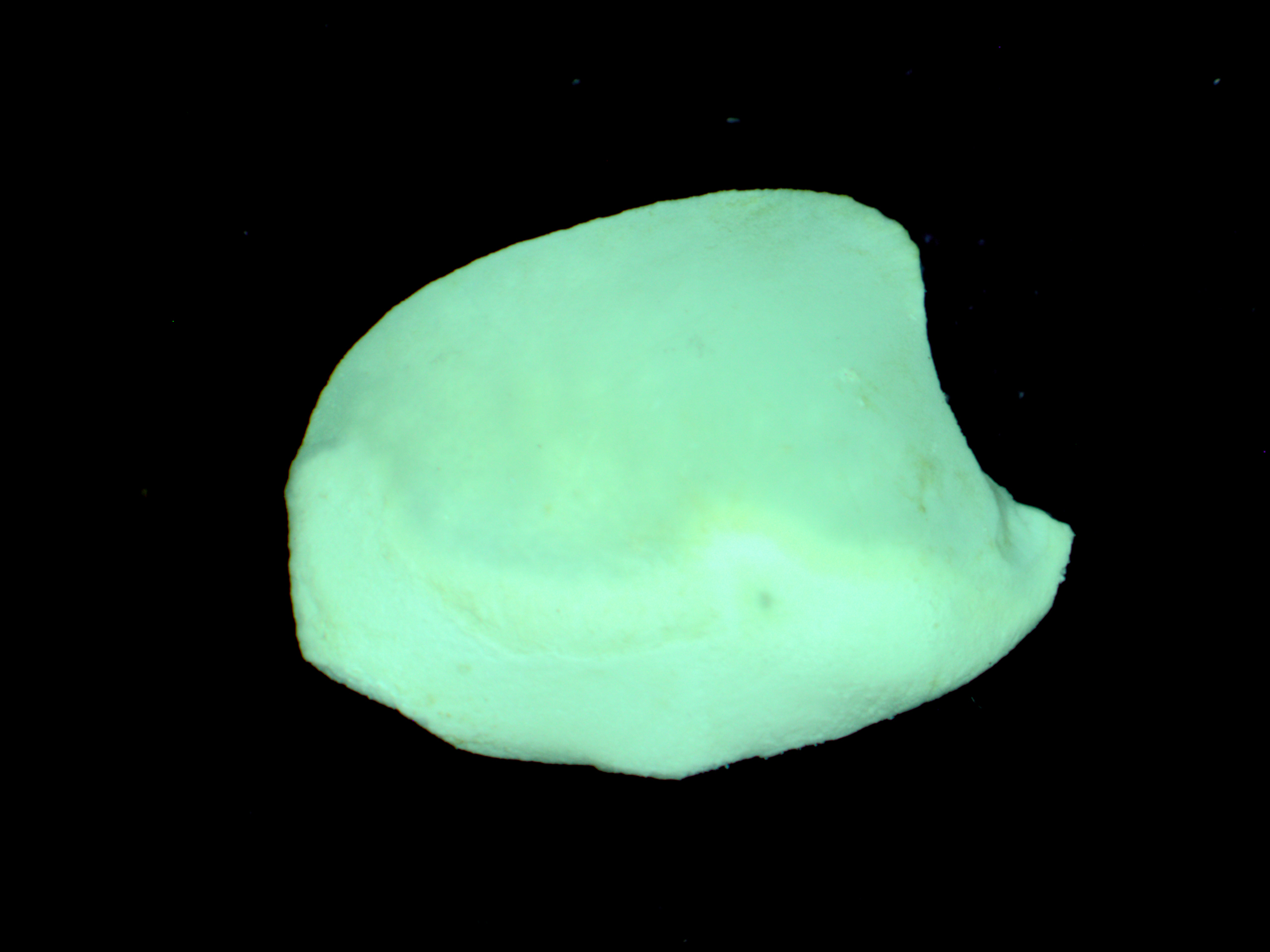

Supplement: Supplemental Information 2 [file peerj-04-1664-s002.zip › AriMac/testing/ARI433_R1.jpg]

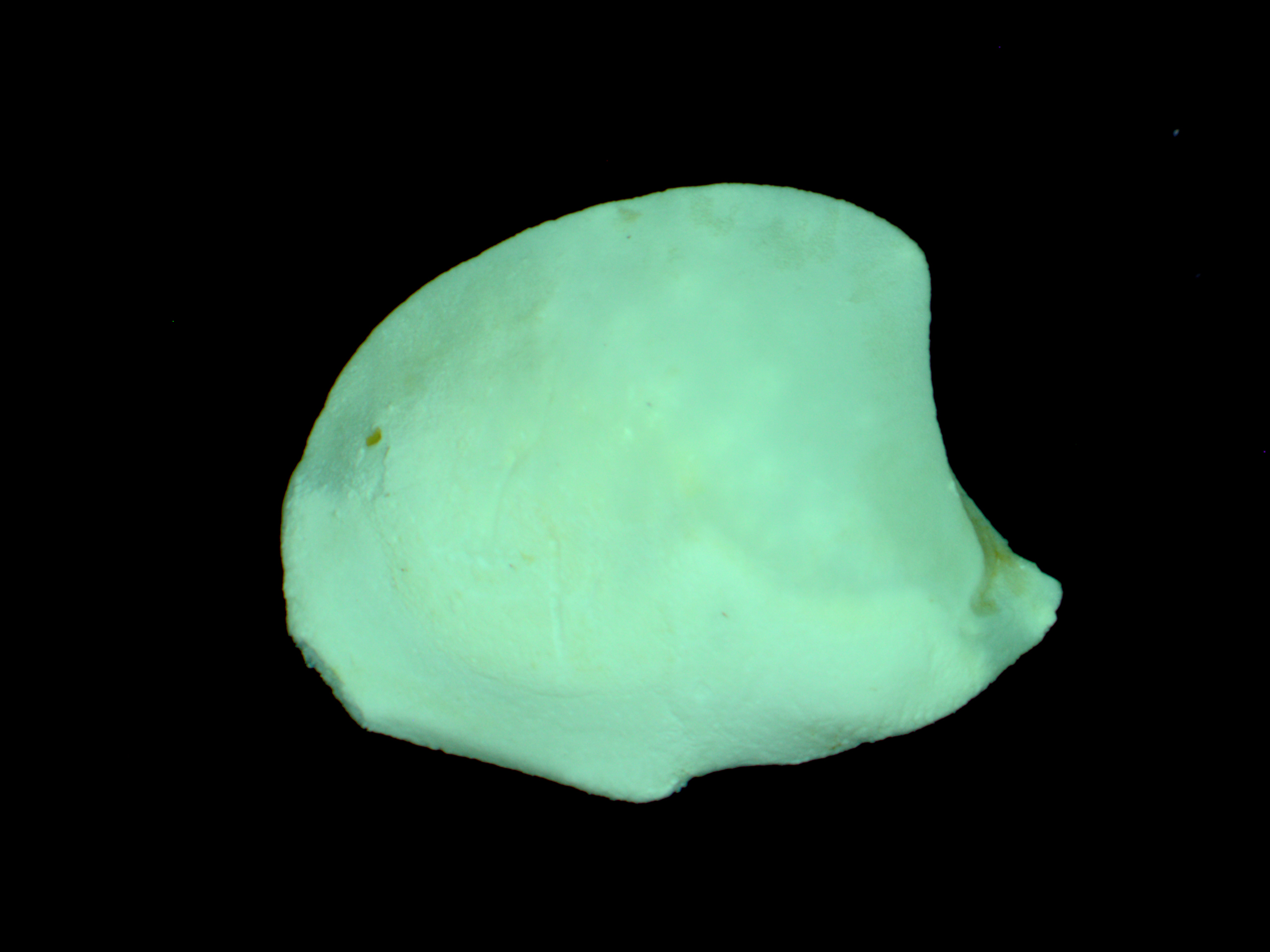

Supplement: Supplemental Information 2 [file peerj-04-1664-s002.zip › AriMac/testing/ARI435_R1.jpg]

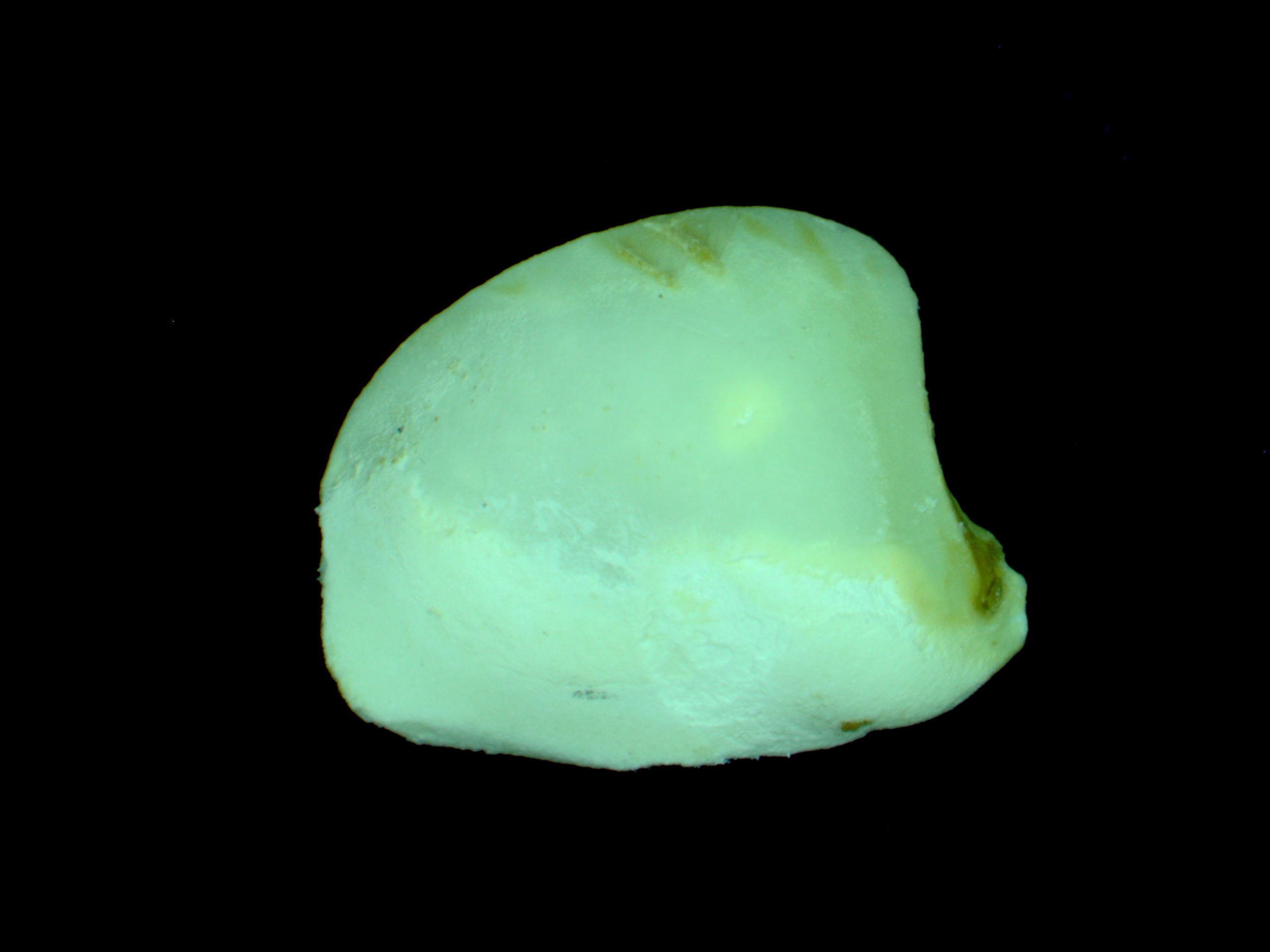

Supplement: Supplemental Information 2 [file peerj-04-1664-s002.zip › AriMac/testing/ARI436_R1.jpg]

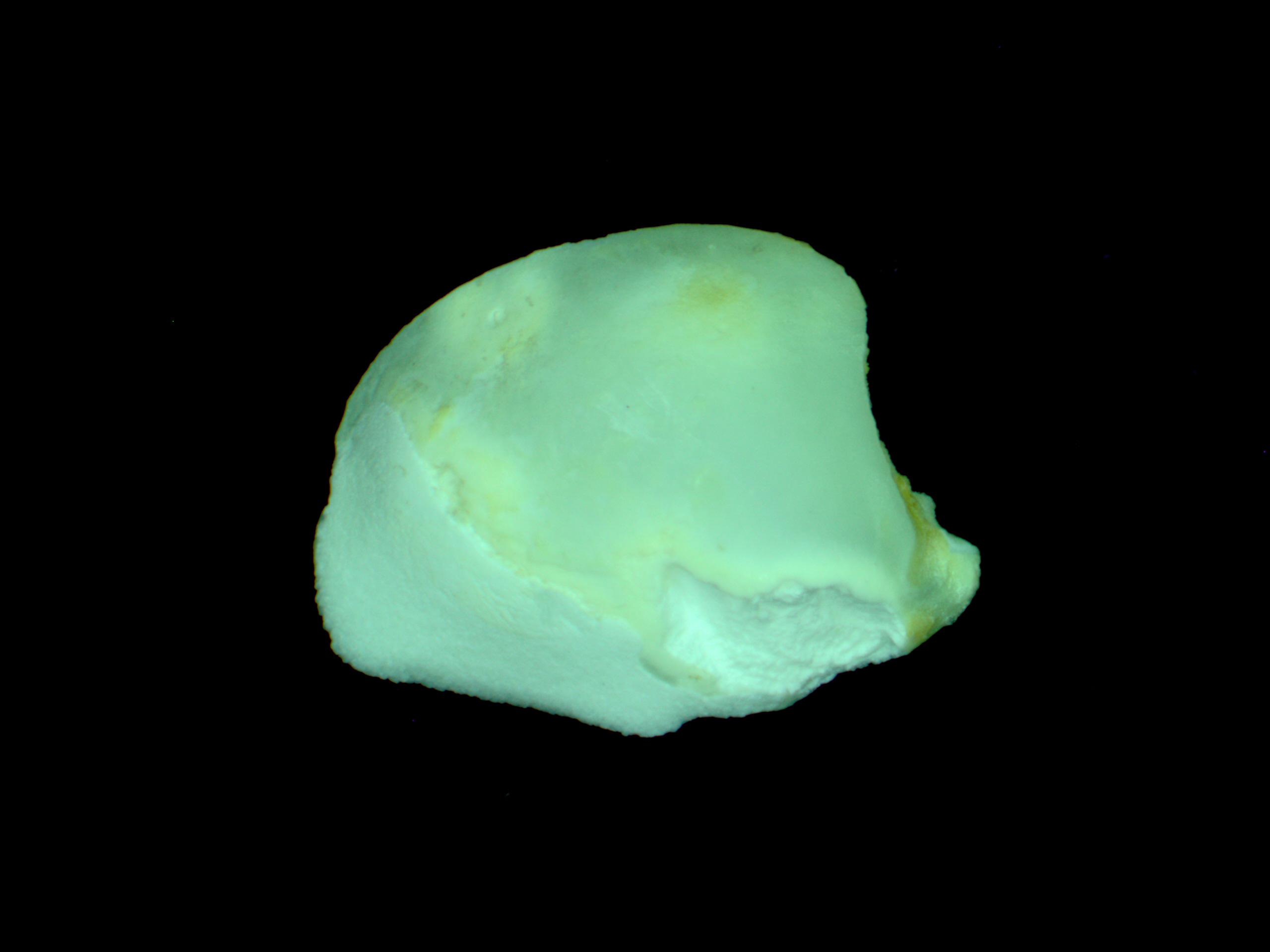

Supplement: Supplemental Information 2 [file peerj-04-1664-s002.zip › AriMac/testing/ARI438_R1.jpg]

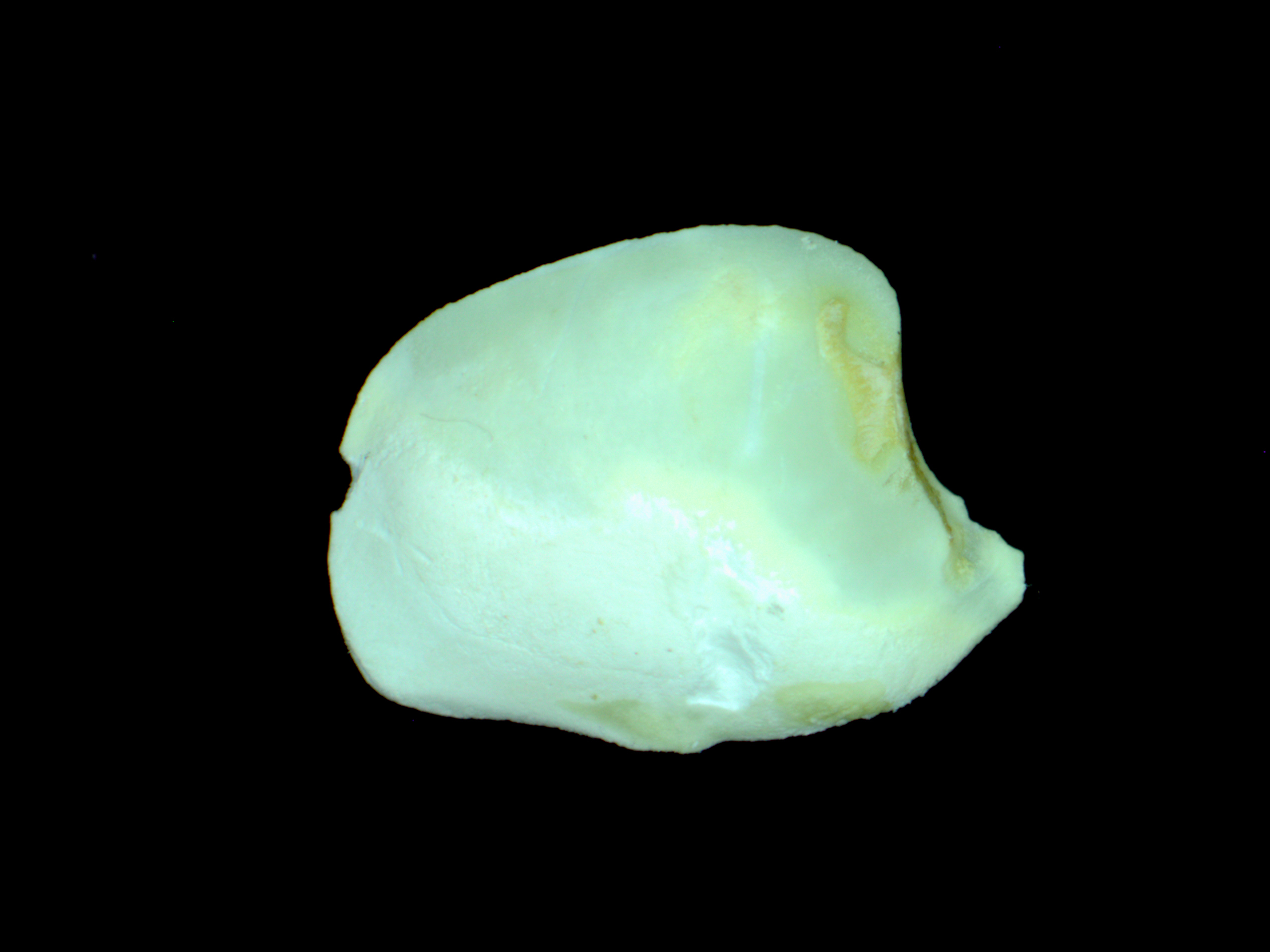

Supplement: Supplemental Information 2 [file peerj-04-1664-s002.zip › AriMac/testing/ARI440_R1.jpg]

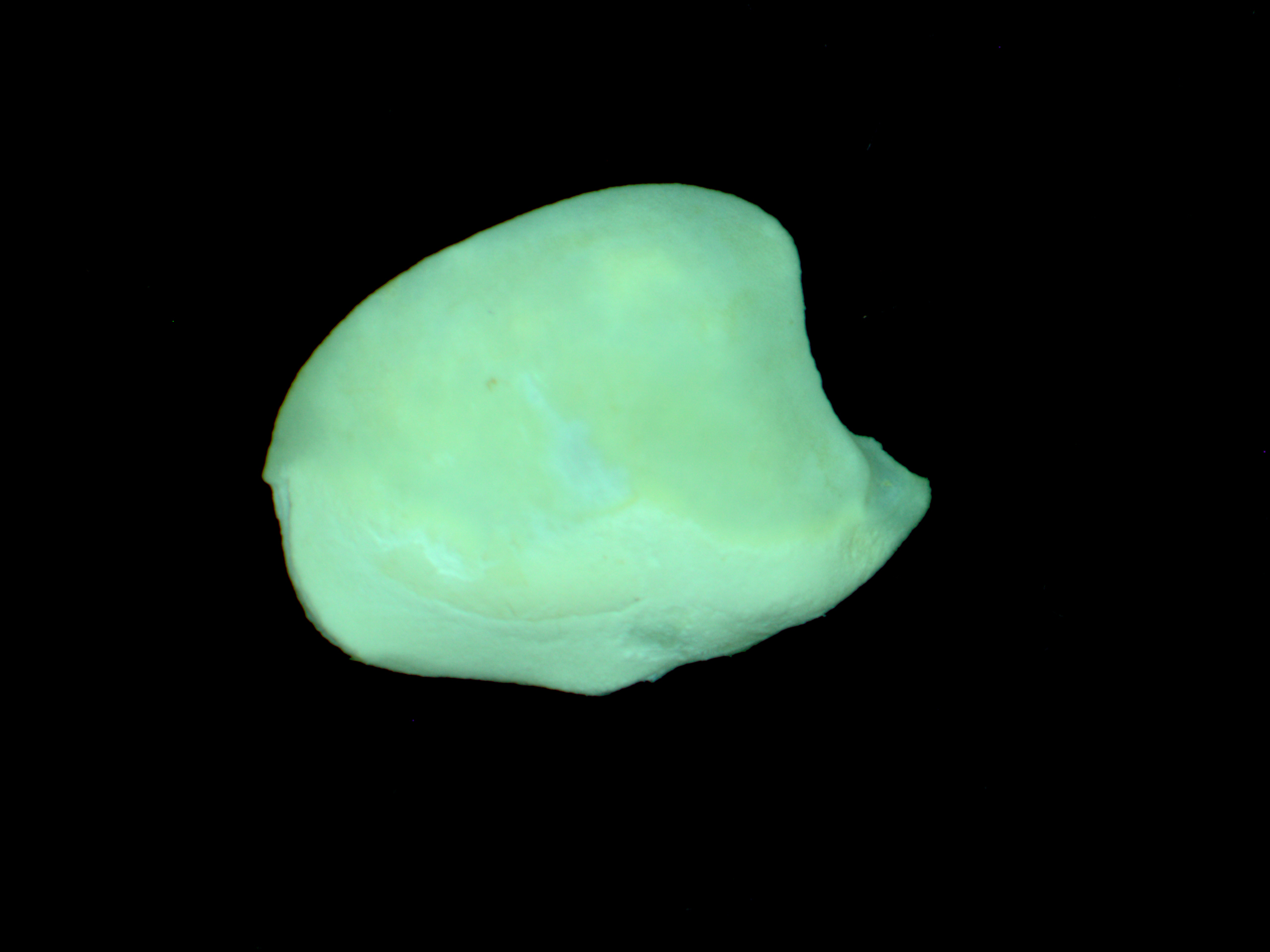

Supplement: Supplemental Information 2 [file peerj-04-1664-s002.zip › AriMac/testing/ARI445_R1.jpg]

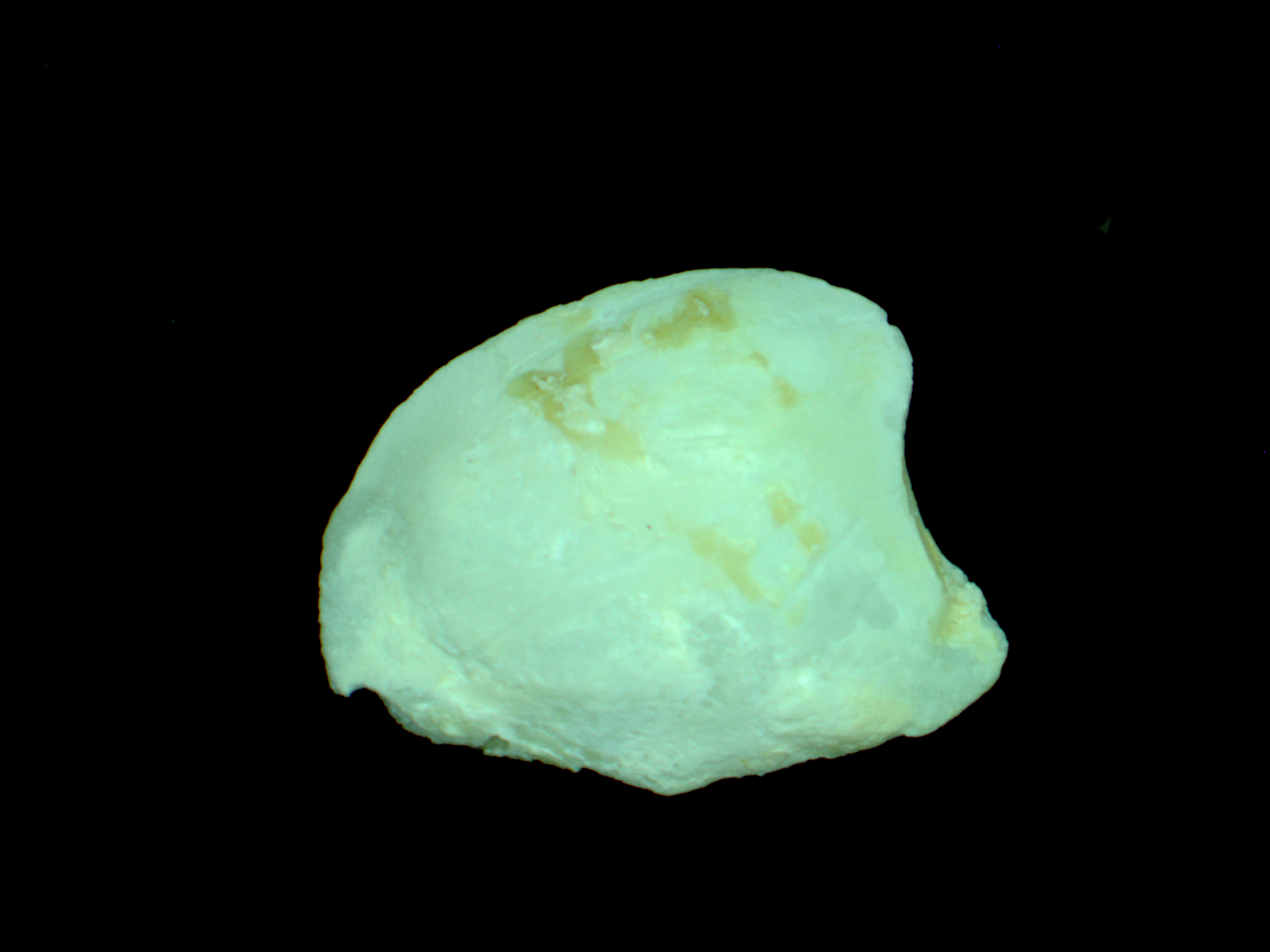

Supplement: Supplemental Information 2 [file peerj-04-1664-s002.zip › AriMac/training/ARI303_R1.jpg]

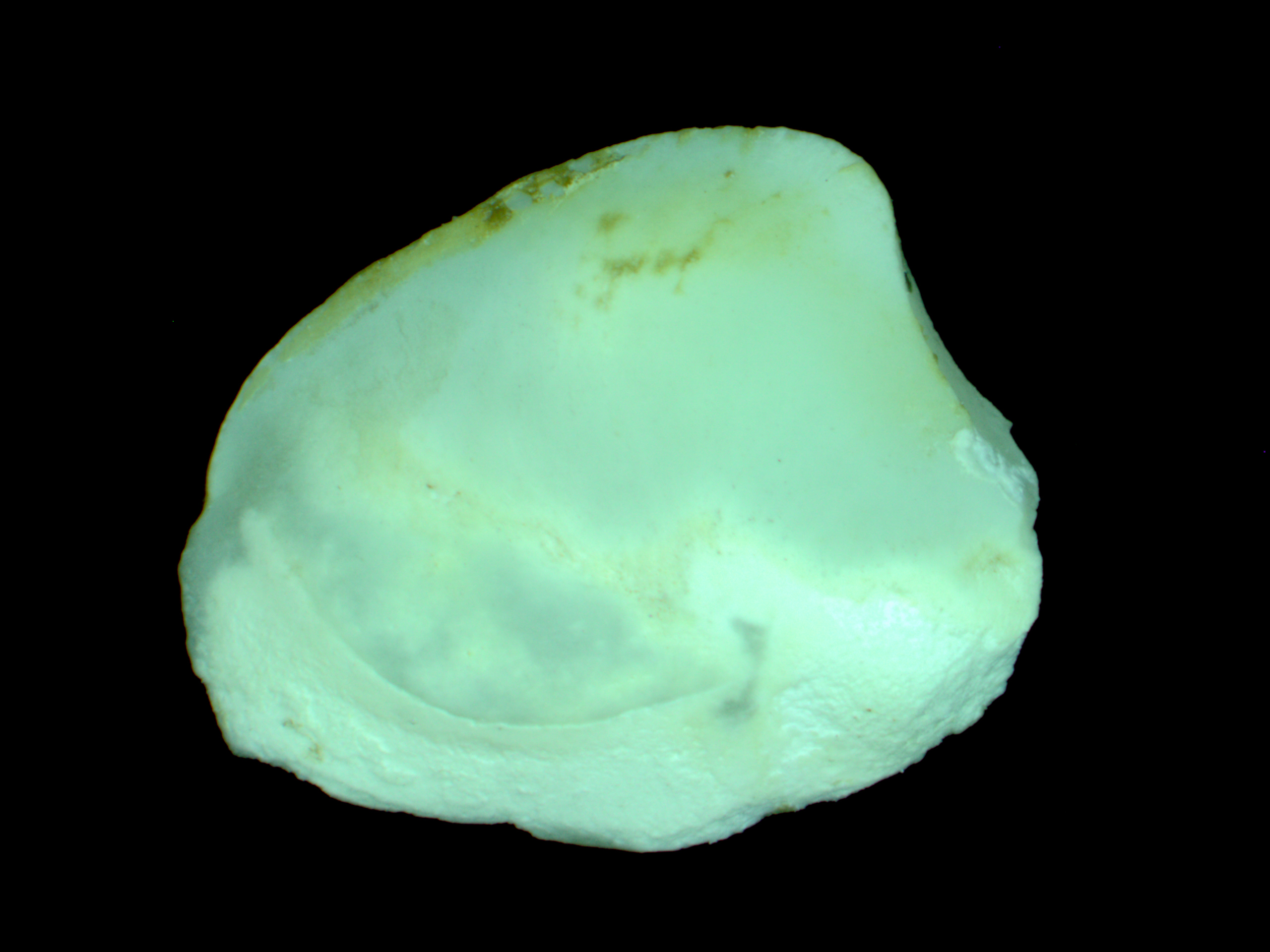

Supplement: Supplemental Information 2 [file peerj-04-1664-s002.zip › AriMac/training/ARI351_R1.jpg]

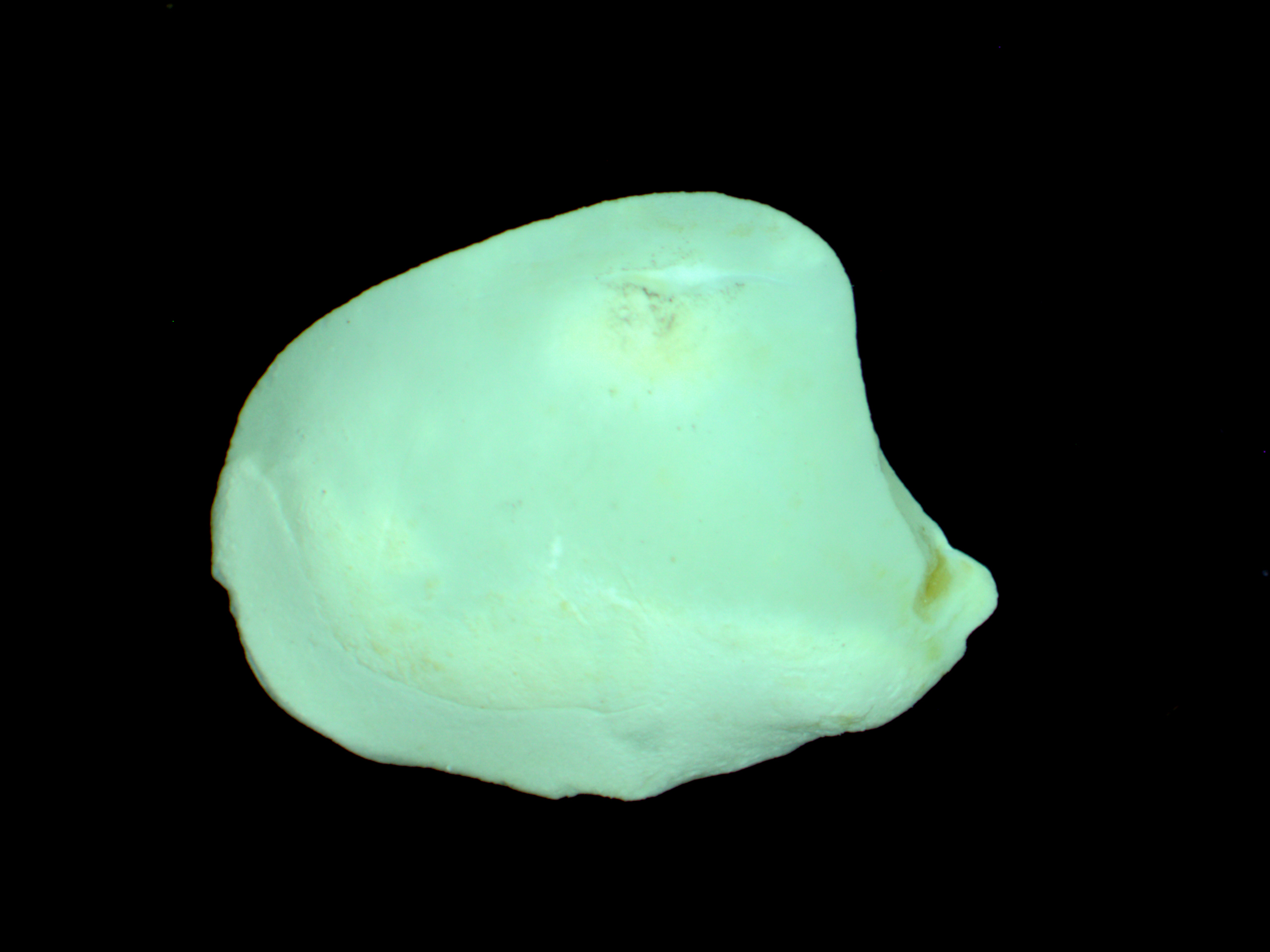

Supplement: Supplemental Information 2 [file peerj-04-1664-s002.zip › AriMac/training/ARI396_R1.jpg]

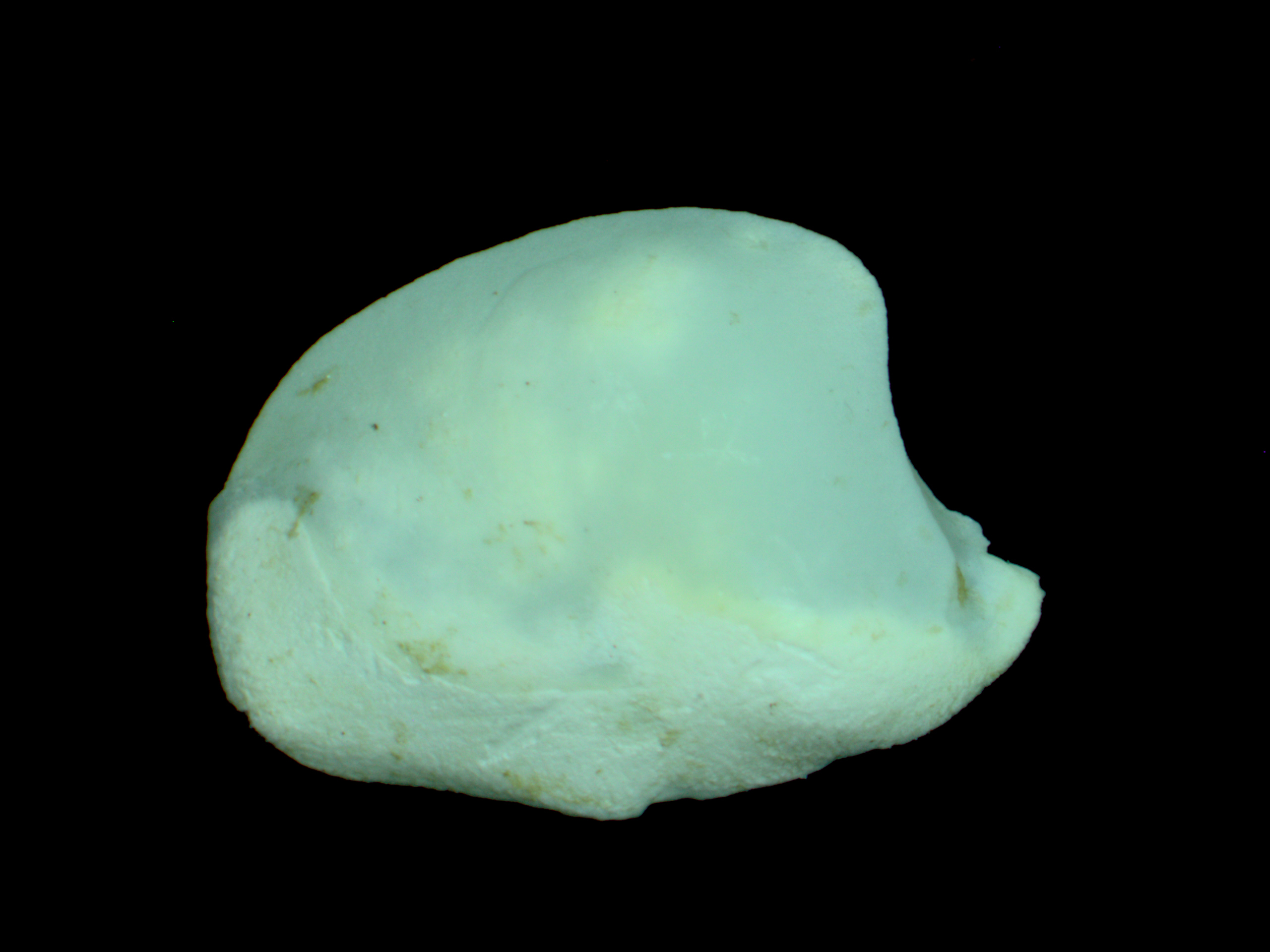

Supplement: Supplemental Information 2 [file peerj-04-1664-s002.zip › AriMac/training/ARI397_R1.jpg]

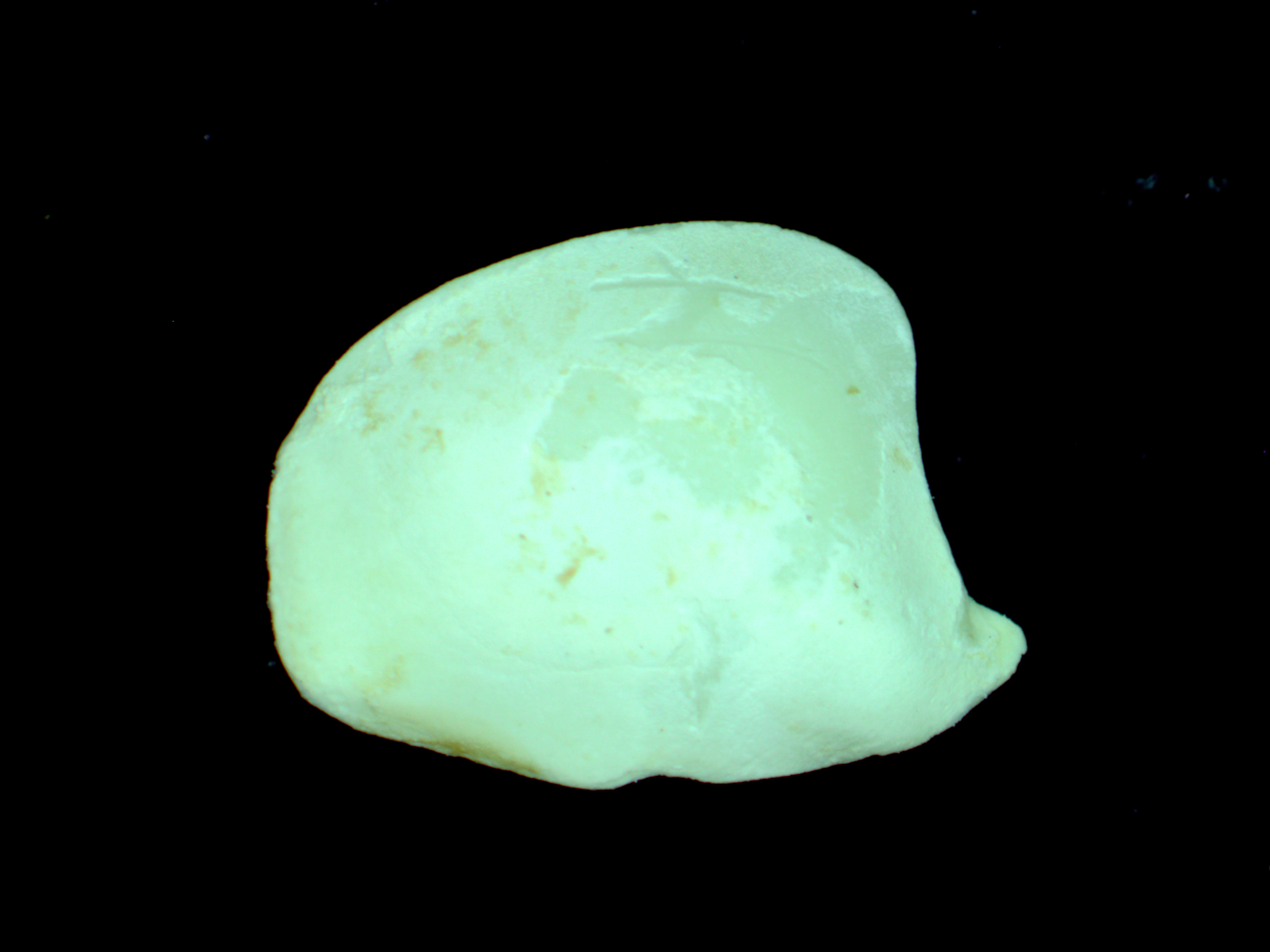

Supplement: Supplemental Information 2 [file peerj-04-1664-s002.zip › AriMac/training/ARI398_R1.jpg]

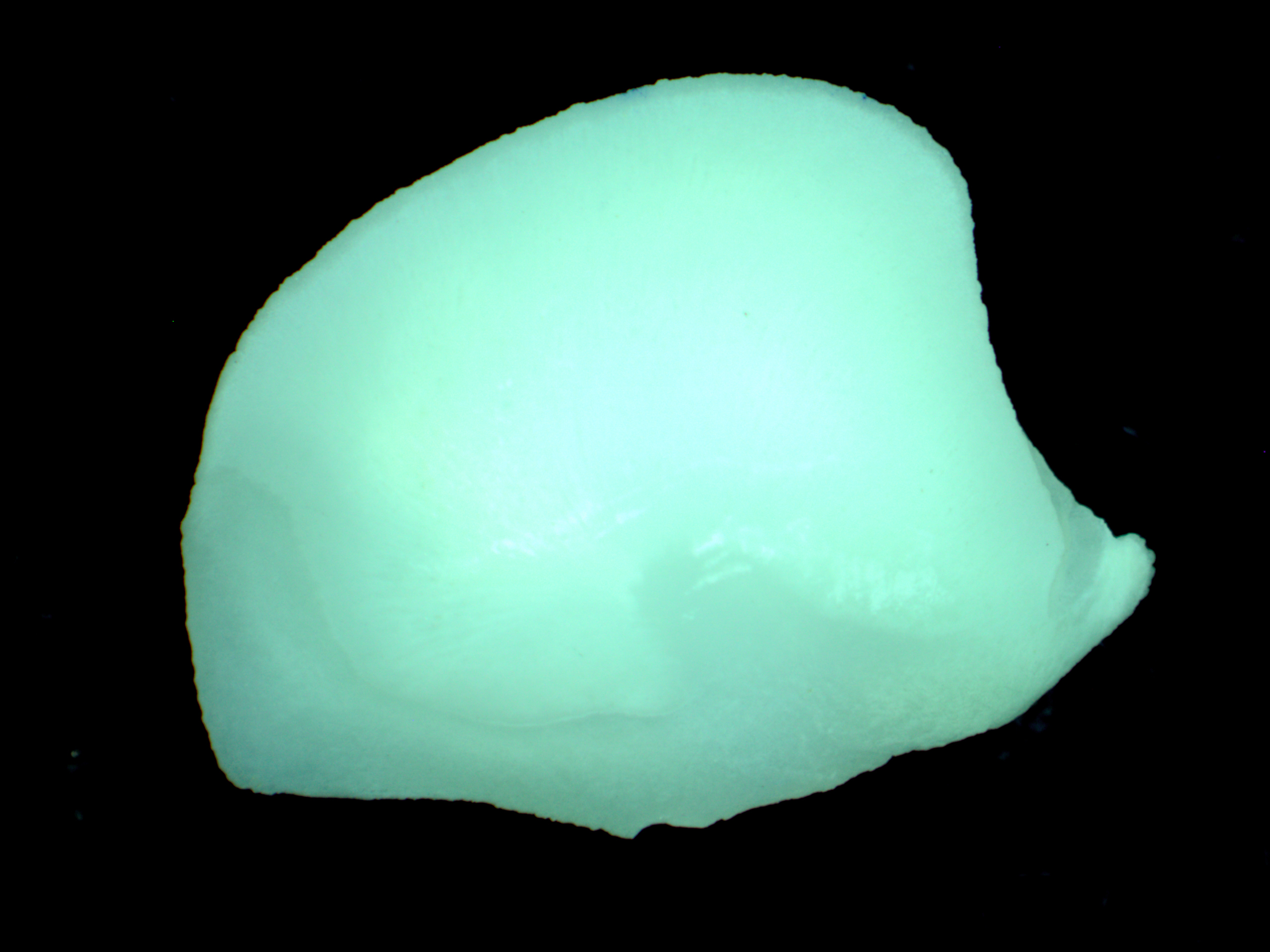

Supplement: Supplemental Information 2 [file peerj-04-1664-s002.zip › AriMac/training/ARI3_R1.jpg]

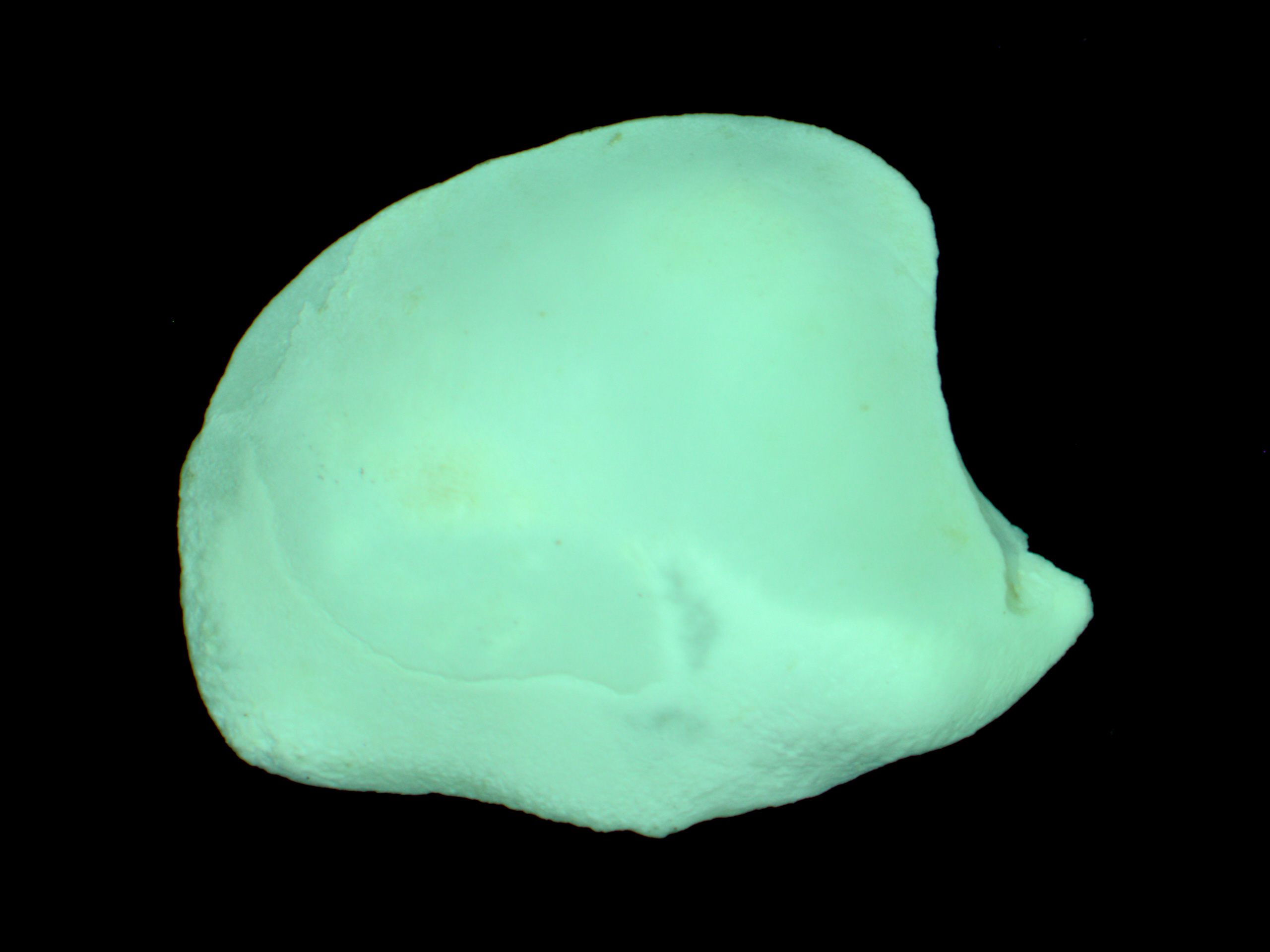

Supplement: Supplemental Information 2 [file peerj-04-1664-s002.zip › AriMac/training/ARI409_R1.jpg]

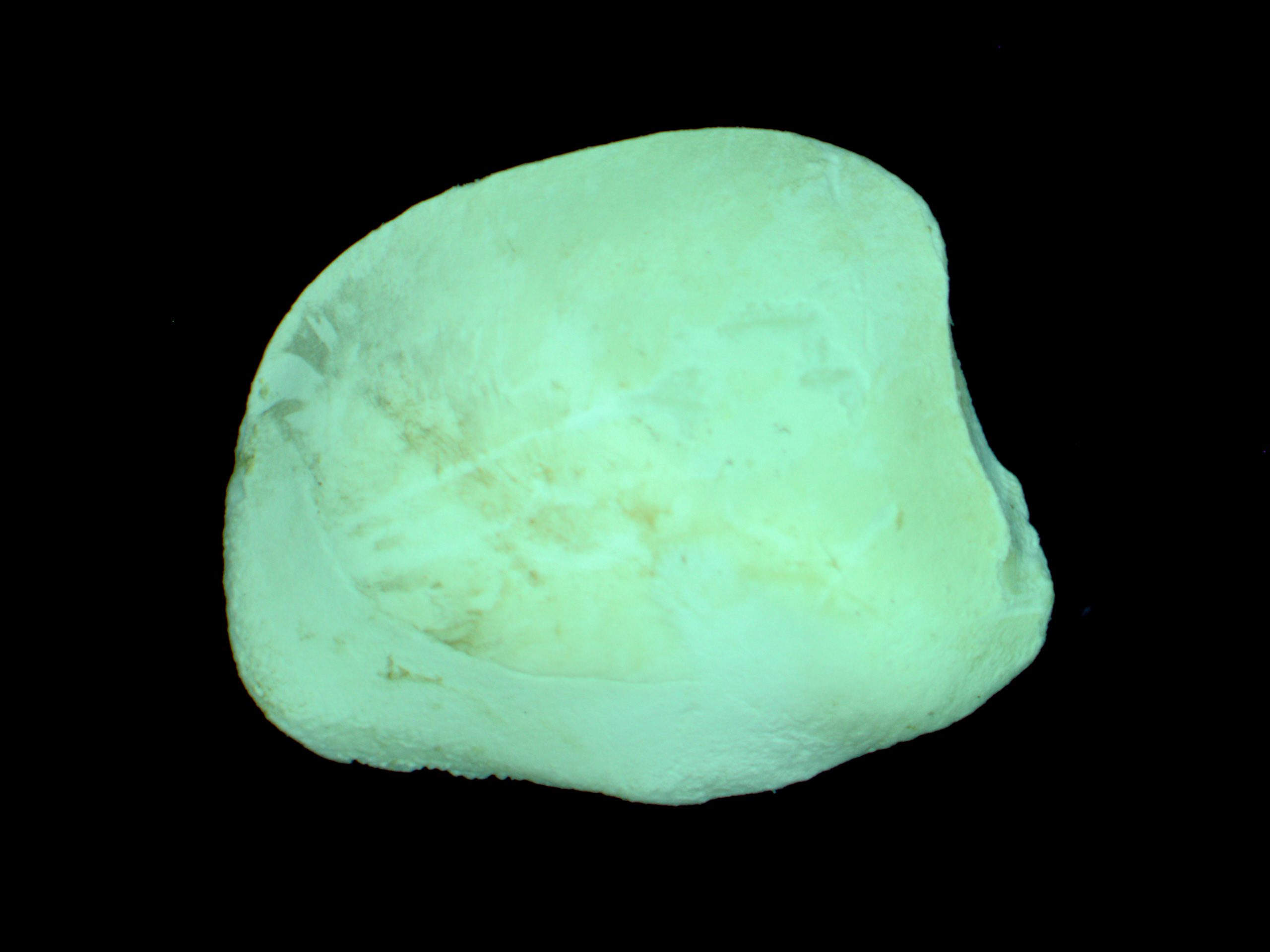

Supplement: Supplemental Information 2 [file peerj-04-1664-s002.zip › AriMac/training/ARI410_R1.jpg]

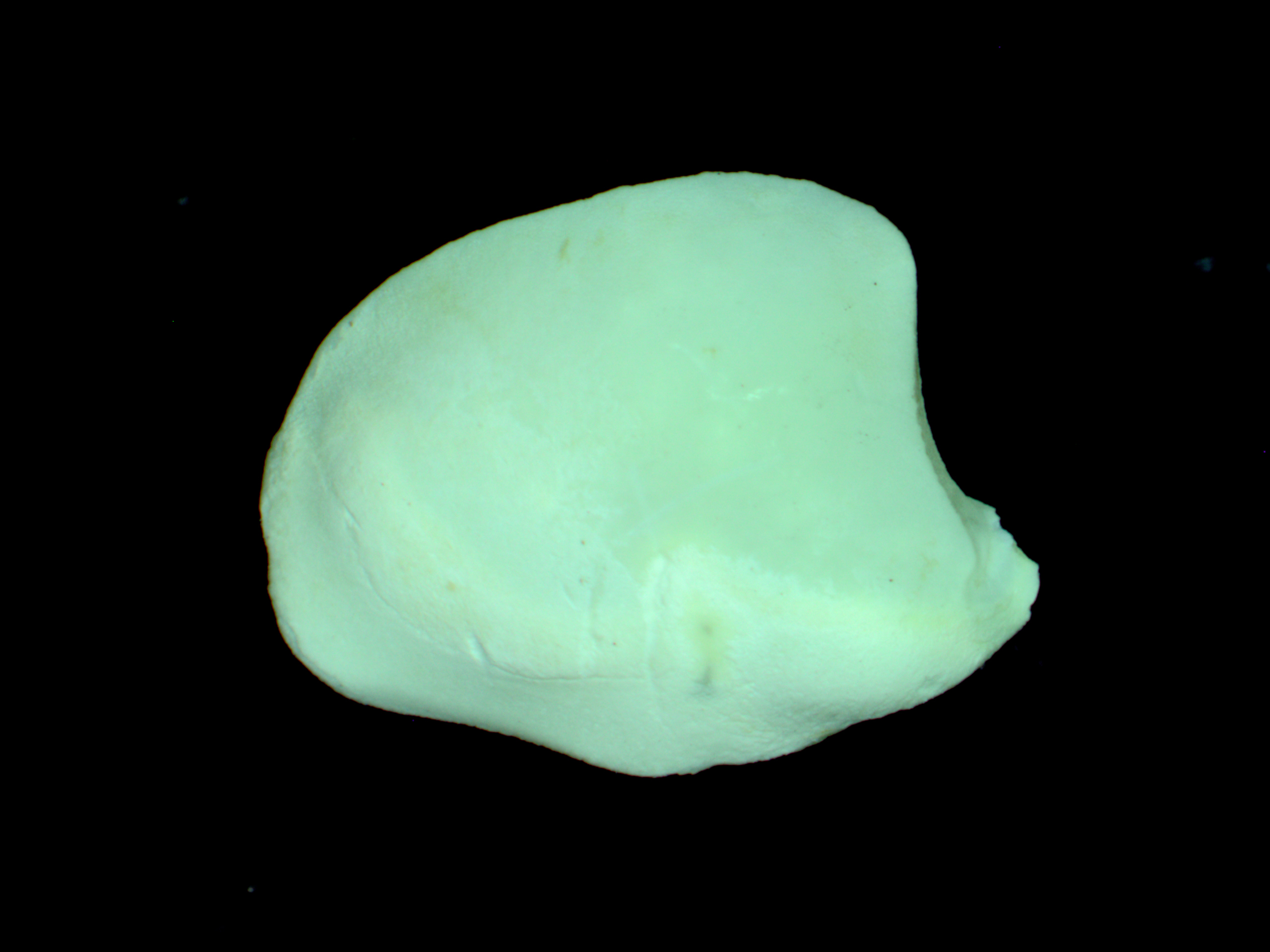

Supplement: Supplemental Information 2 [file peerj-04-1664-s002.zip › AriMac/training/ARI412_R1.jpg]

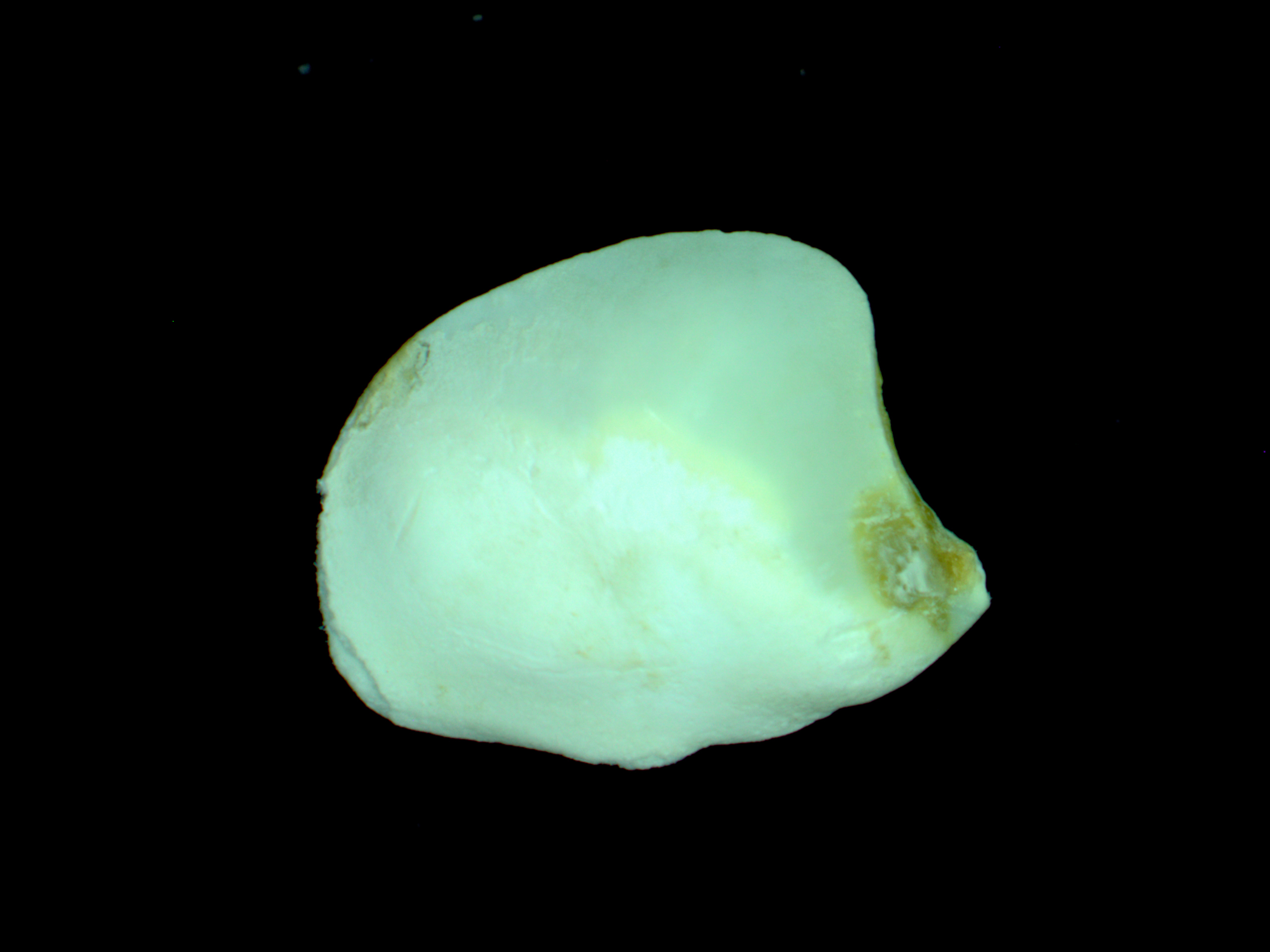

Supplement: Supplemental Information 2 [file peerj-04-1664-s002.zip › AriMac/training/ARI413_R1.jpg]

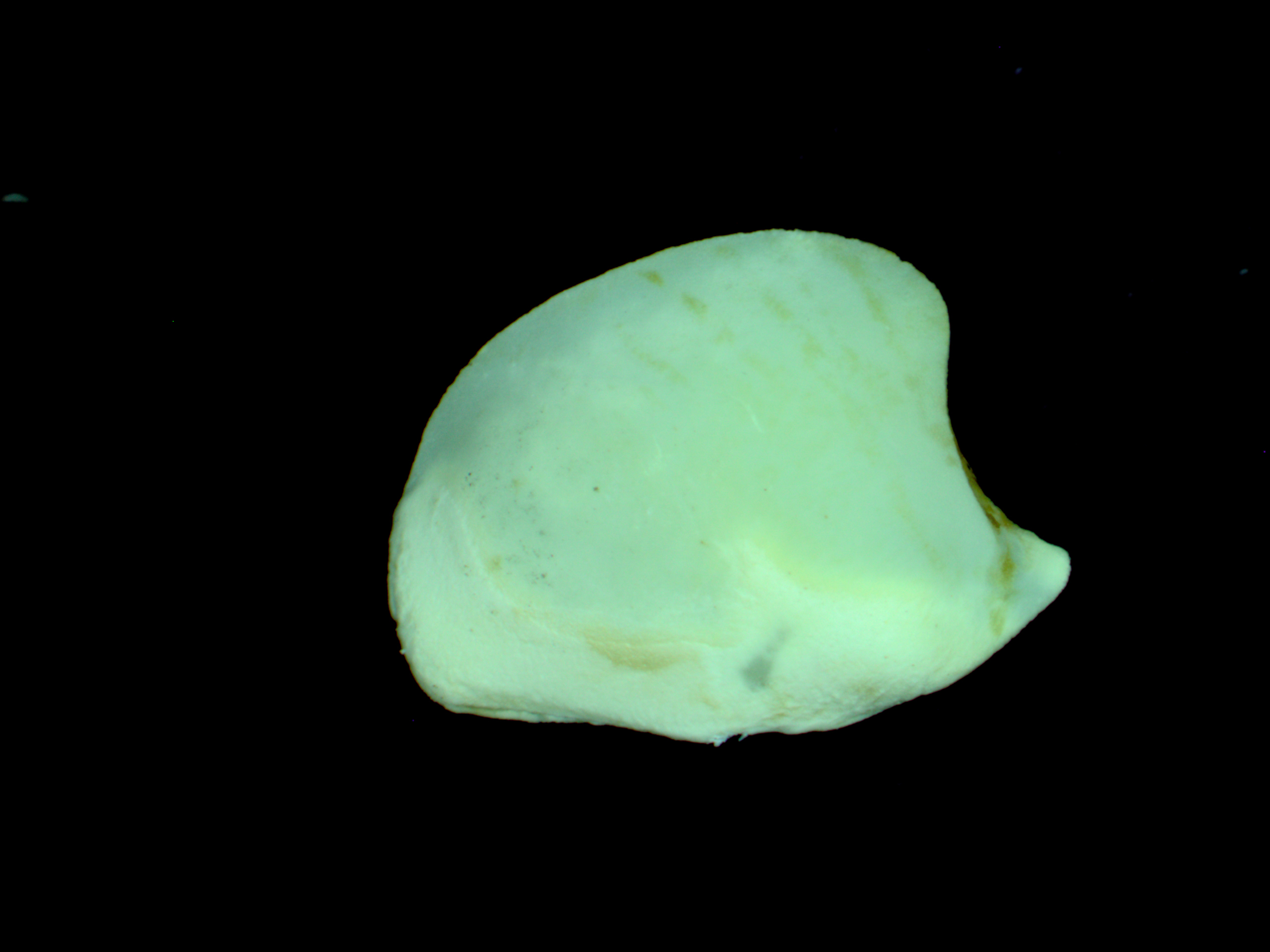

Supplement: Supplemental Information 2 [file peerj-04-1664-s002.zip › AriMac/training/ARI415_R1.jpg]

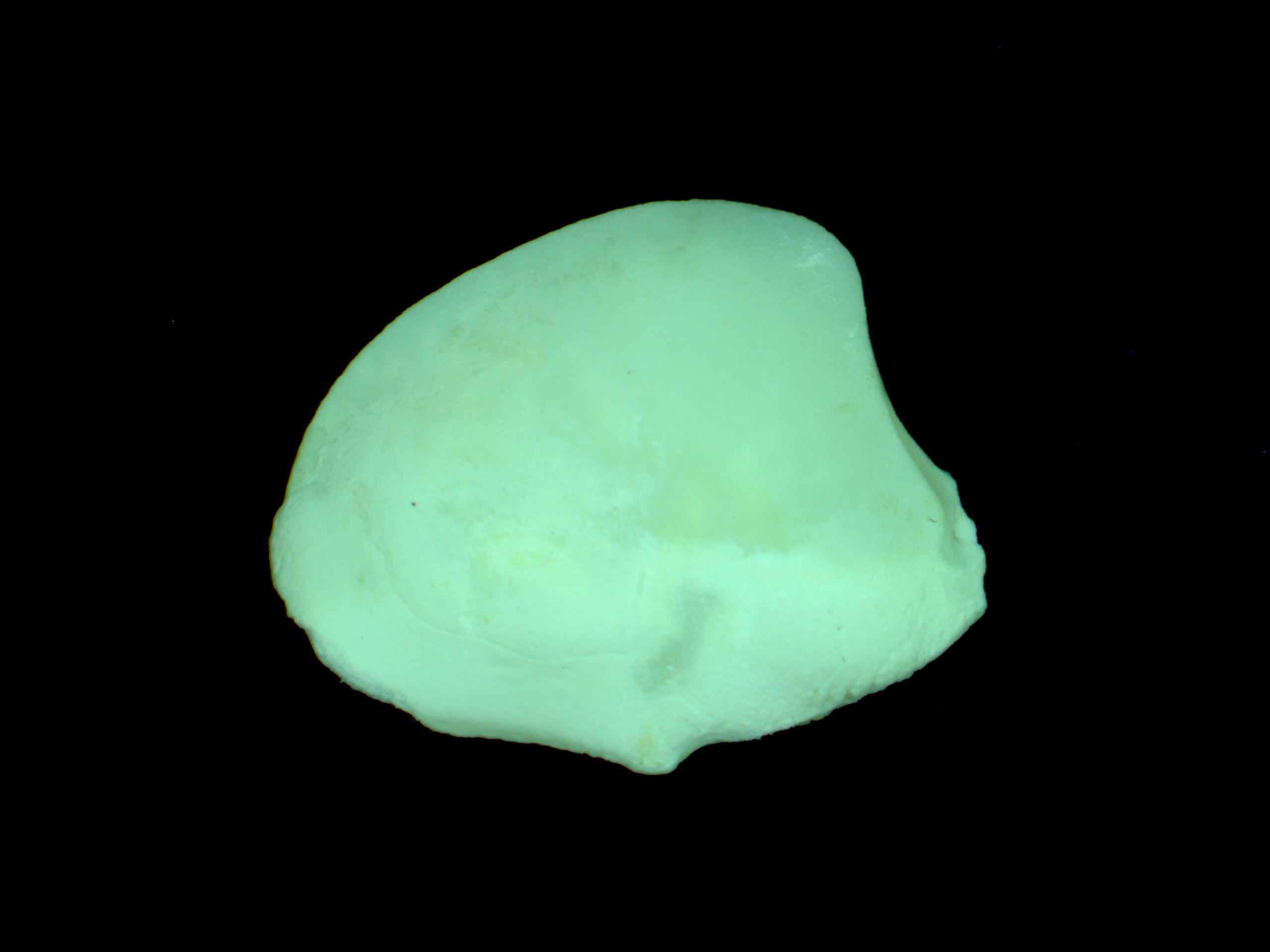

Supplement: Supplemental Information 2 [file peerj-04-1664-s002.zip › AriMac/training/ARI417_R1.jpg]

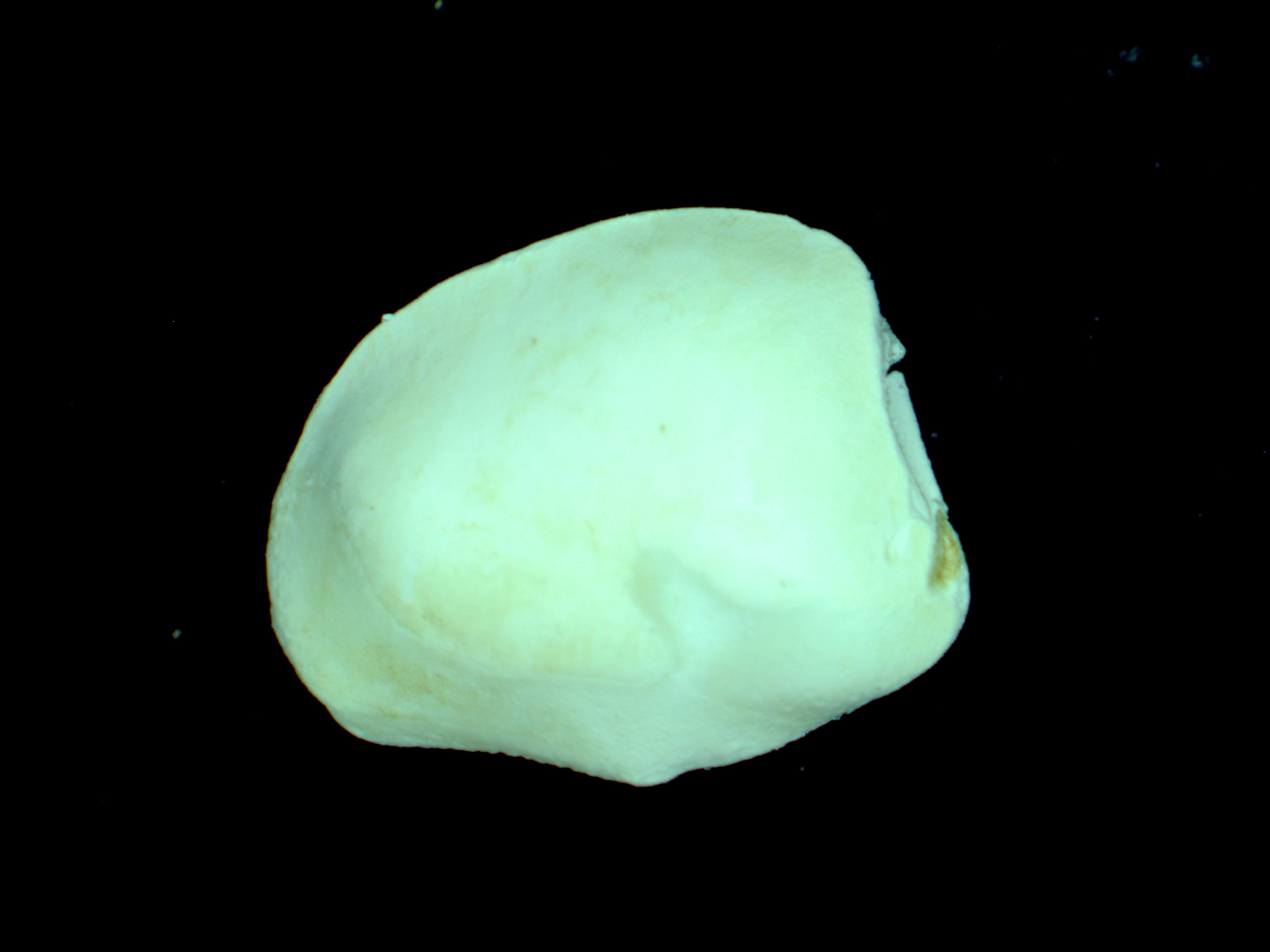

Supplement: Supplemental Information 2 [file peerj-04-1664-s002.zip › AriMac/training/ARI418_R1.jpg]

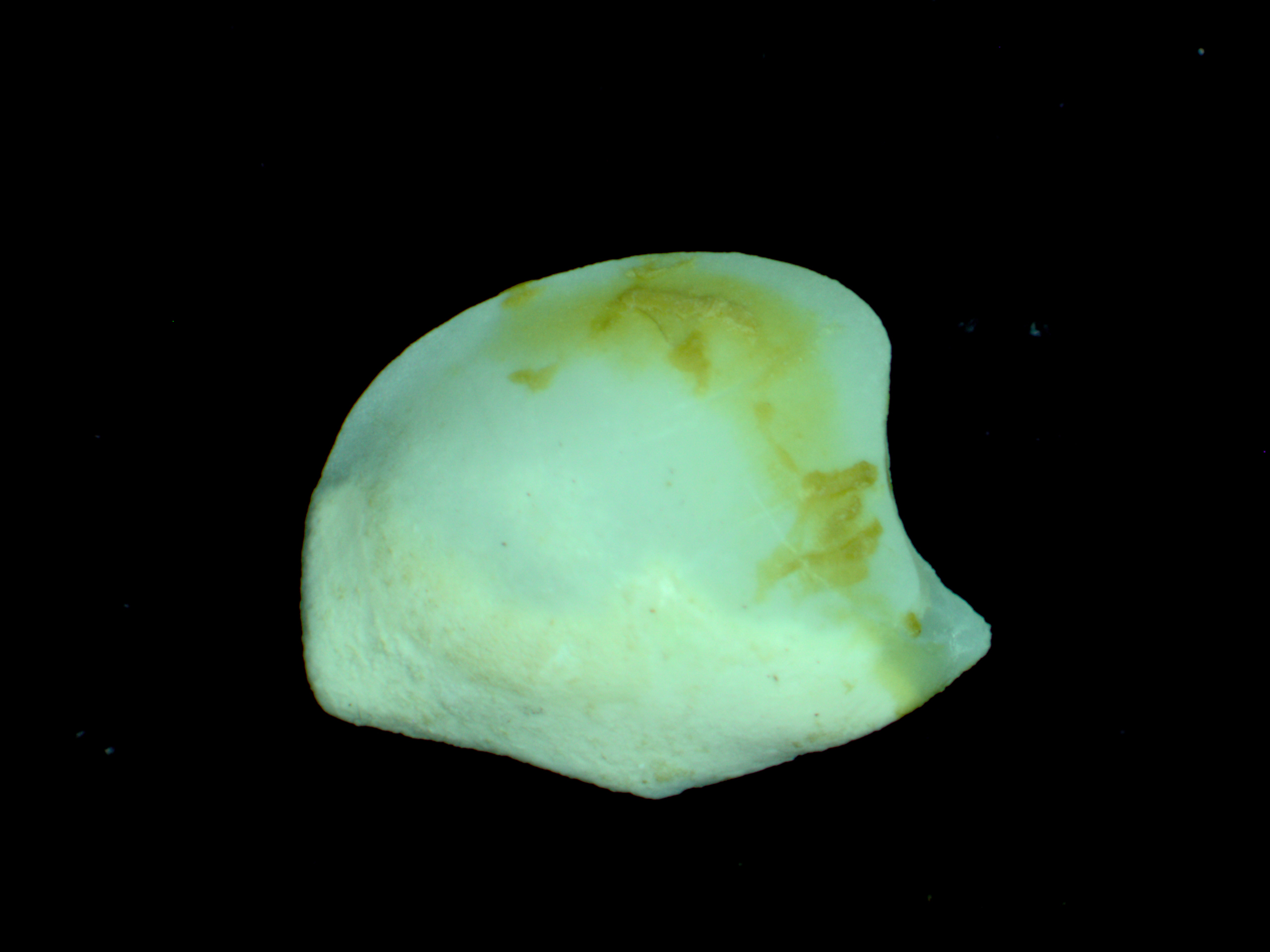

Supplement: Supplemental Information 2 [file peerj-04-1664-s002.zip › AriMac/training/ARI421_R1.jpg]

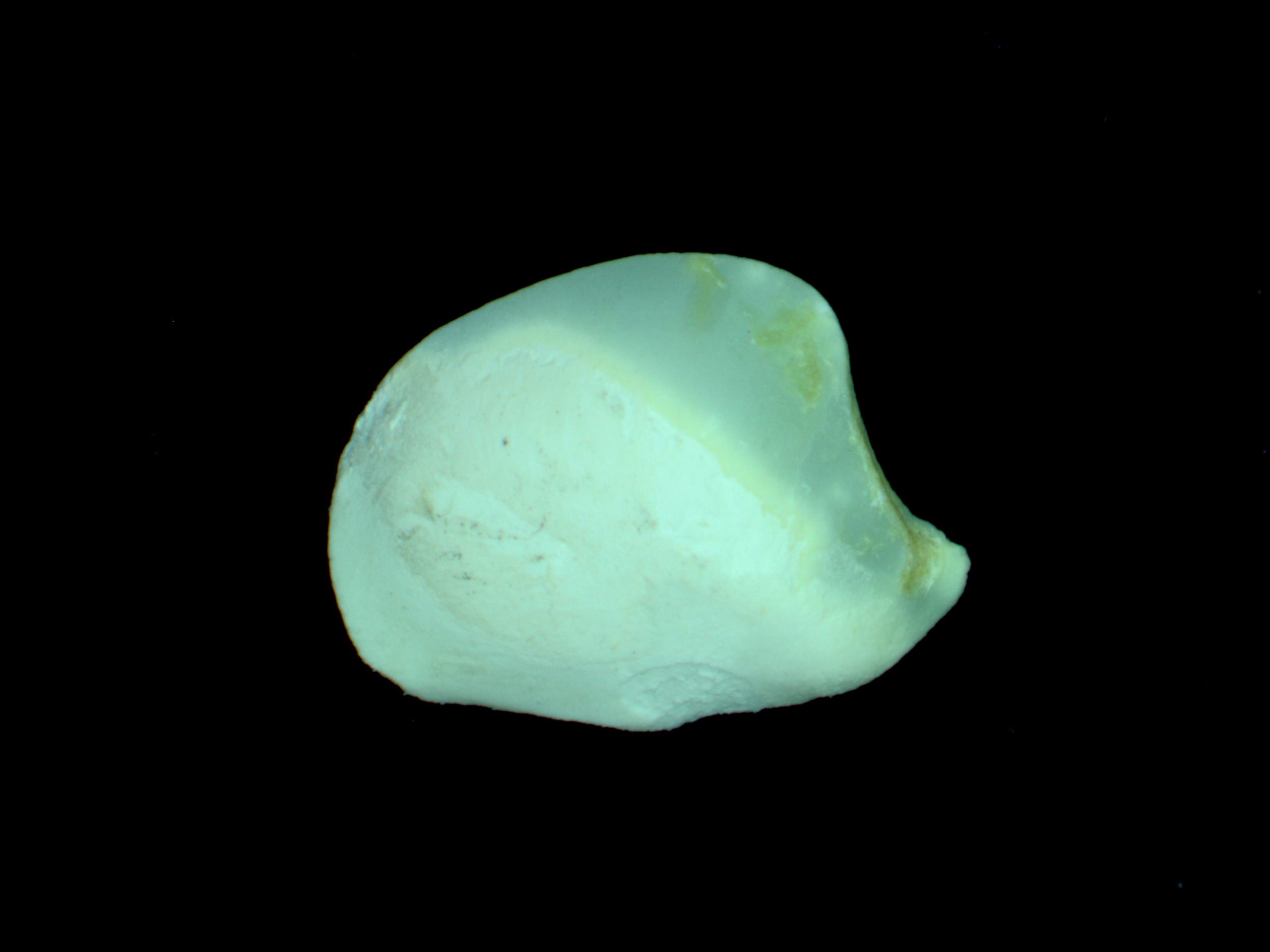

Supplement: Supplemental Information 2 [file peerj-04-1664-s002.zip › AriMac/training/ARI425_R1.jpg]

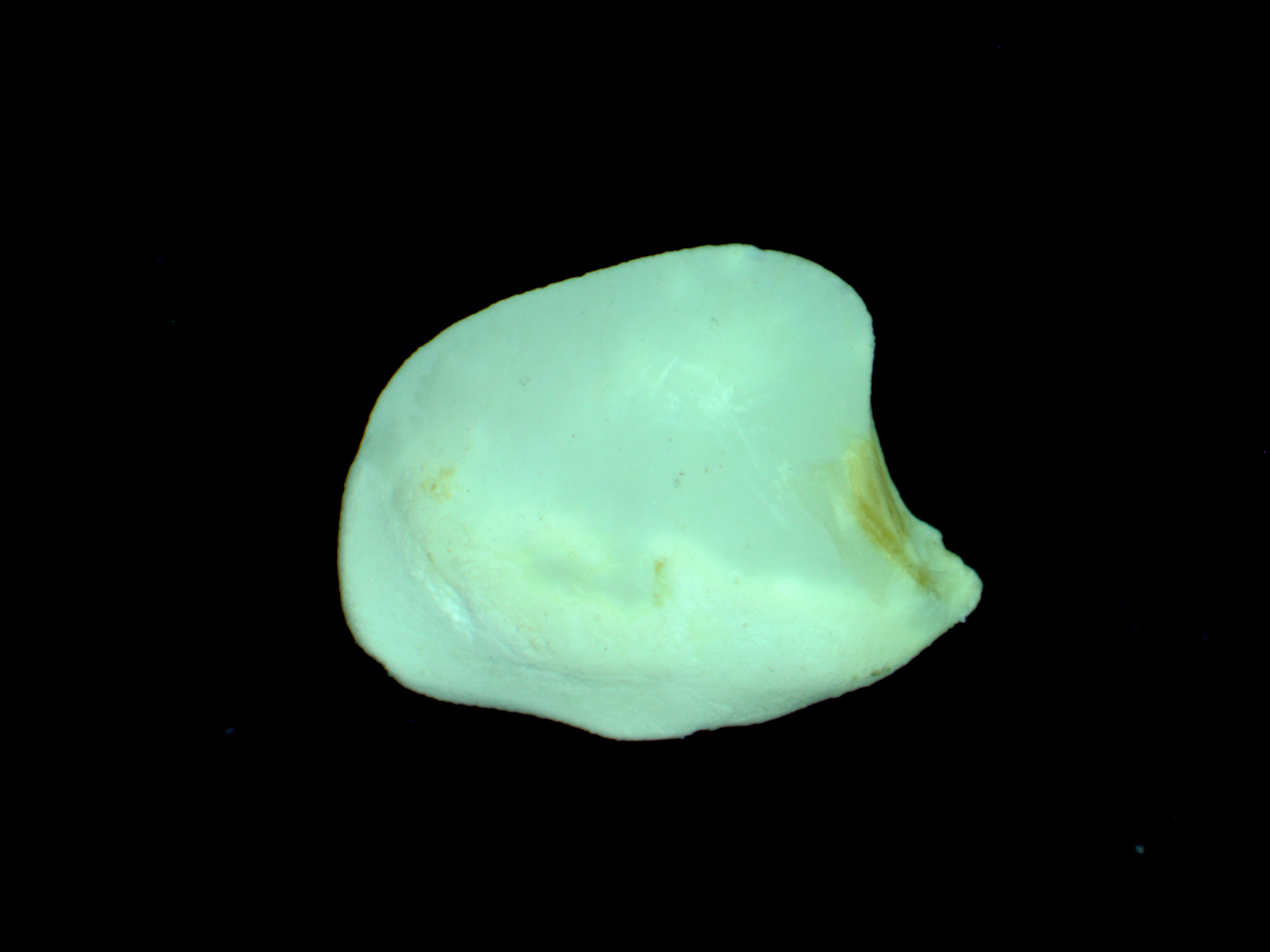

Supplement: Supplemental Information 2 [file peerj-04-1664-s002.zip › AriMac/training/ARI426_R1.jpg]

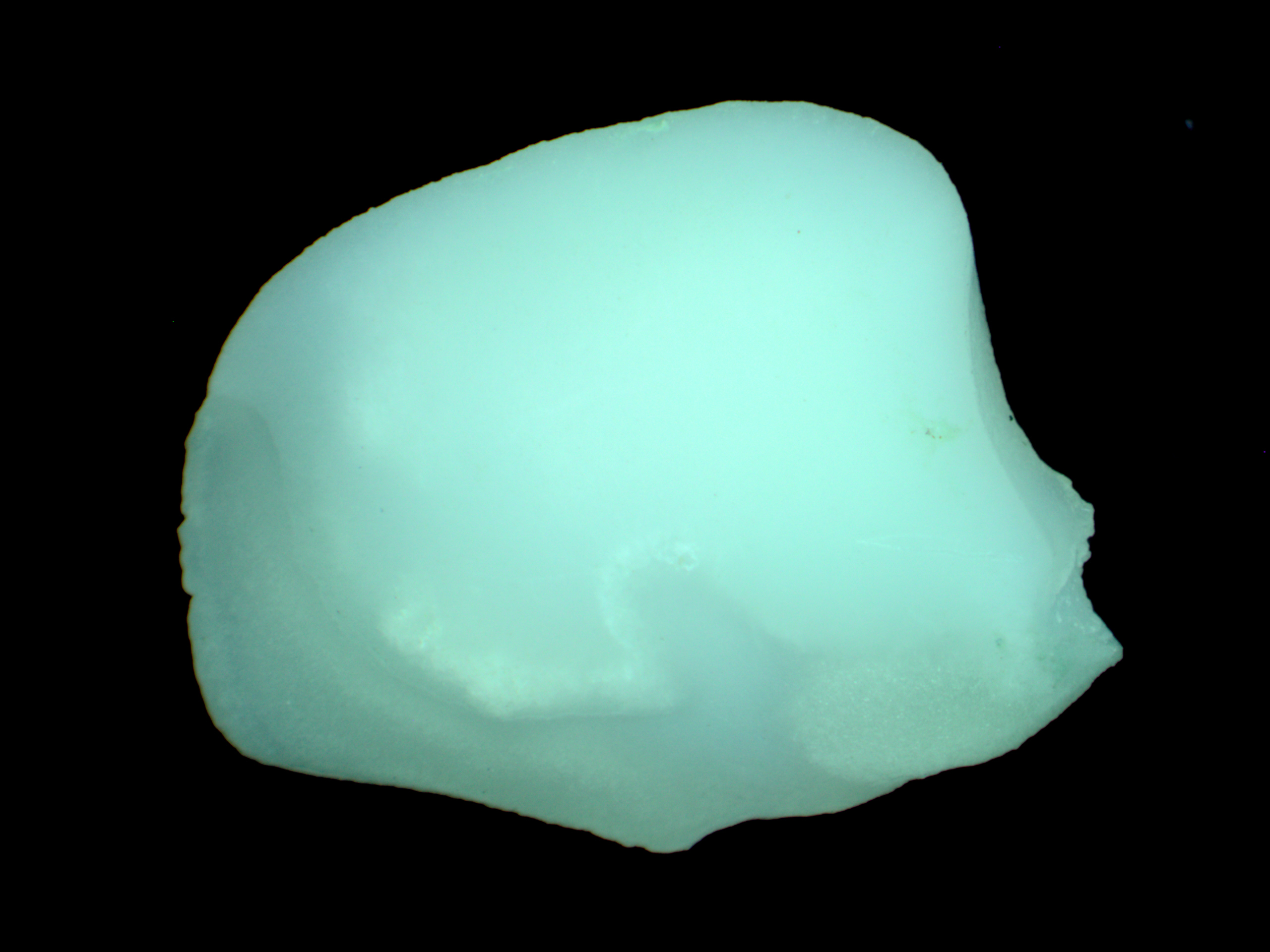

Supplement: Supplemental Information 2 [file peerj-04-1664-s002.zip › AriMac/training/ARI4_R1.jpg]

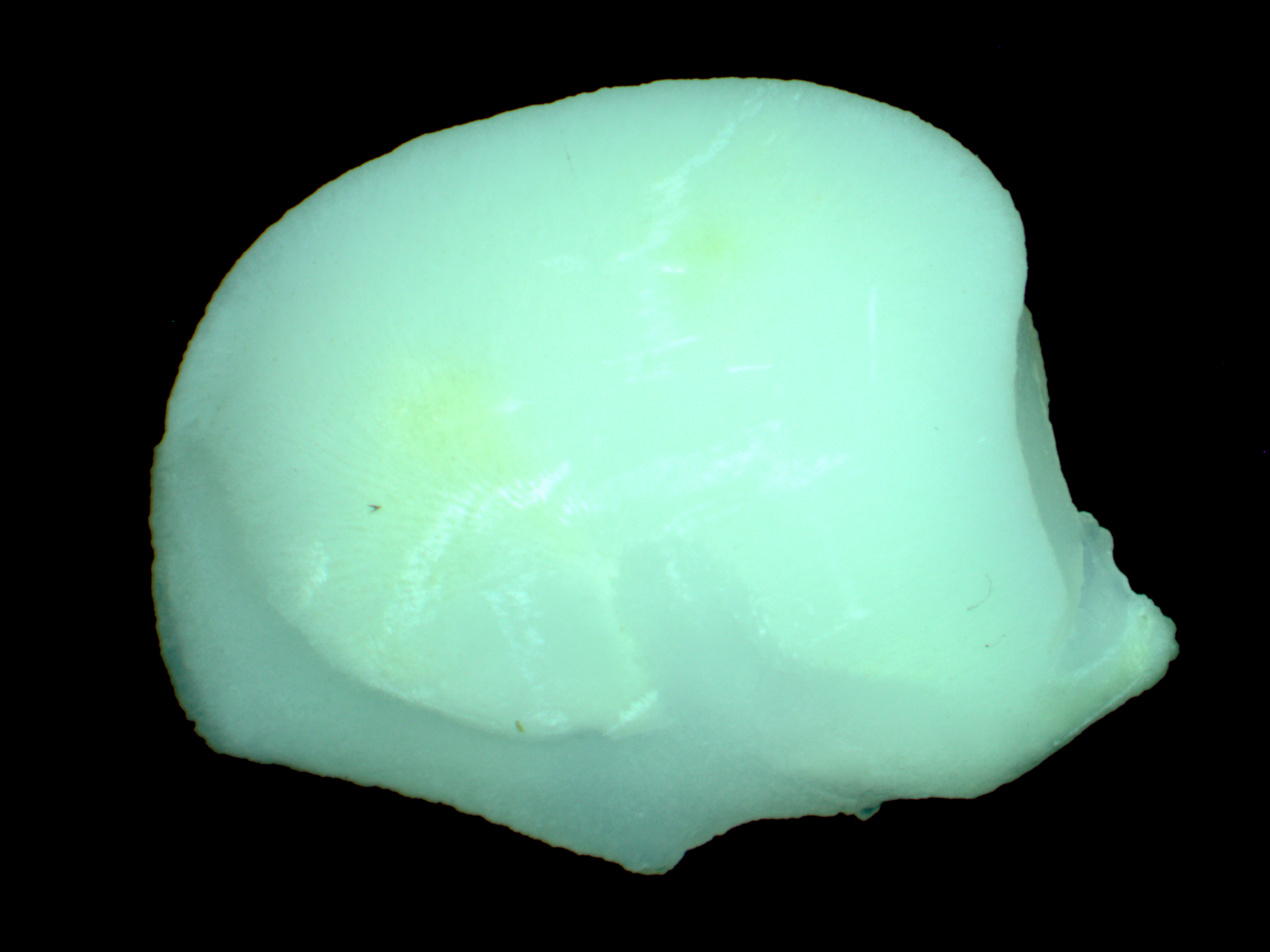

Supplement: Supplemental Information 2 [file peerj-04-1664-s002.zip › AriMac/training/ARI5_R1.jpg]

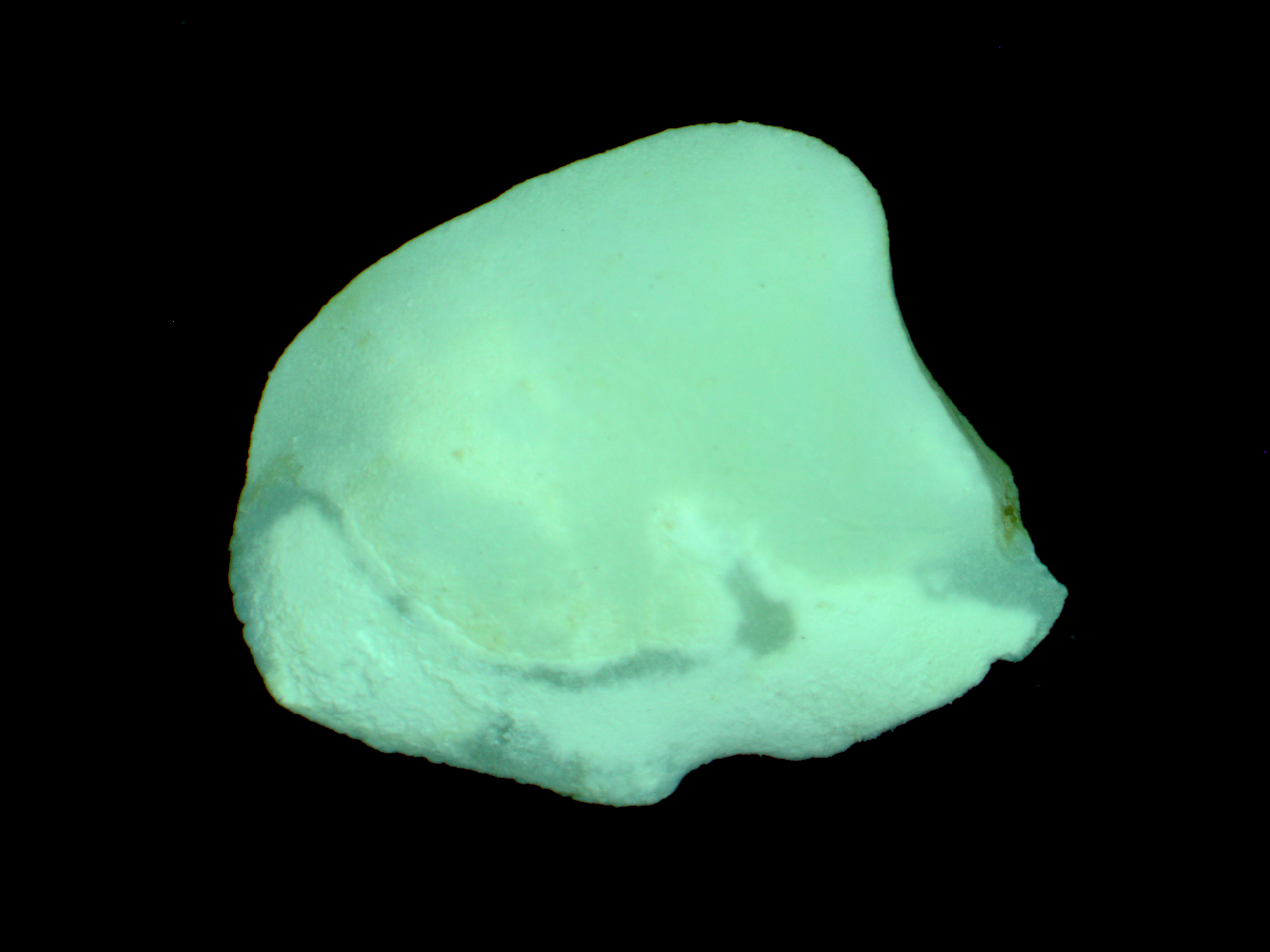

Supplement: Supplemental Information 3 [file peerj-04-1664-s003.zip › CryTru/tetsing/ARI668_R1.jpg]

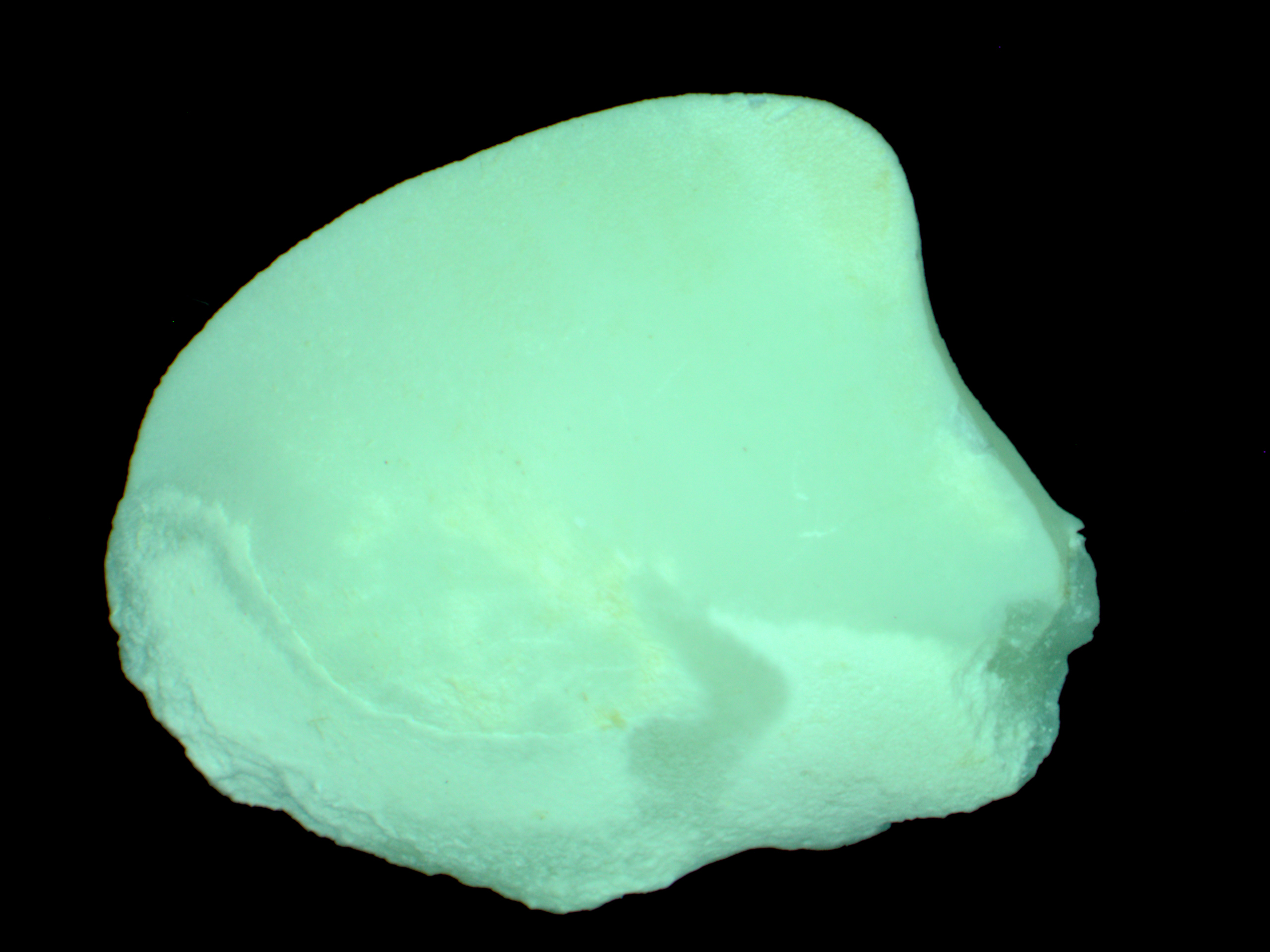

Supplement: Supplemental Information 3 [file peerj-04-1664-s003.zip › CryTru/tetsing/ARI672_R1.jpg]

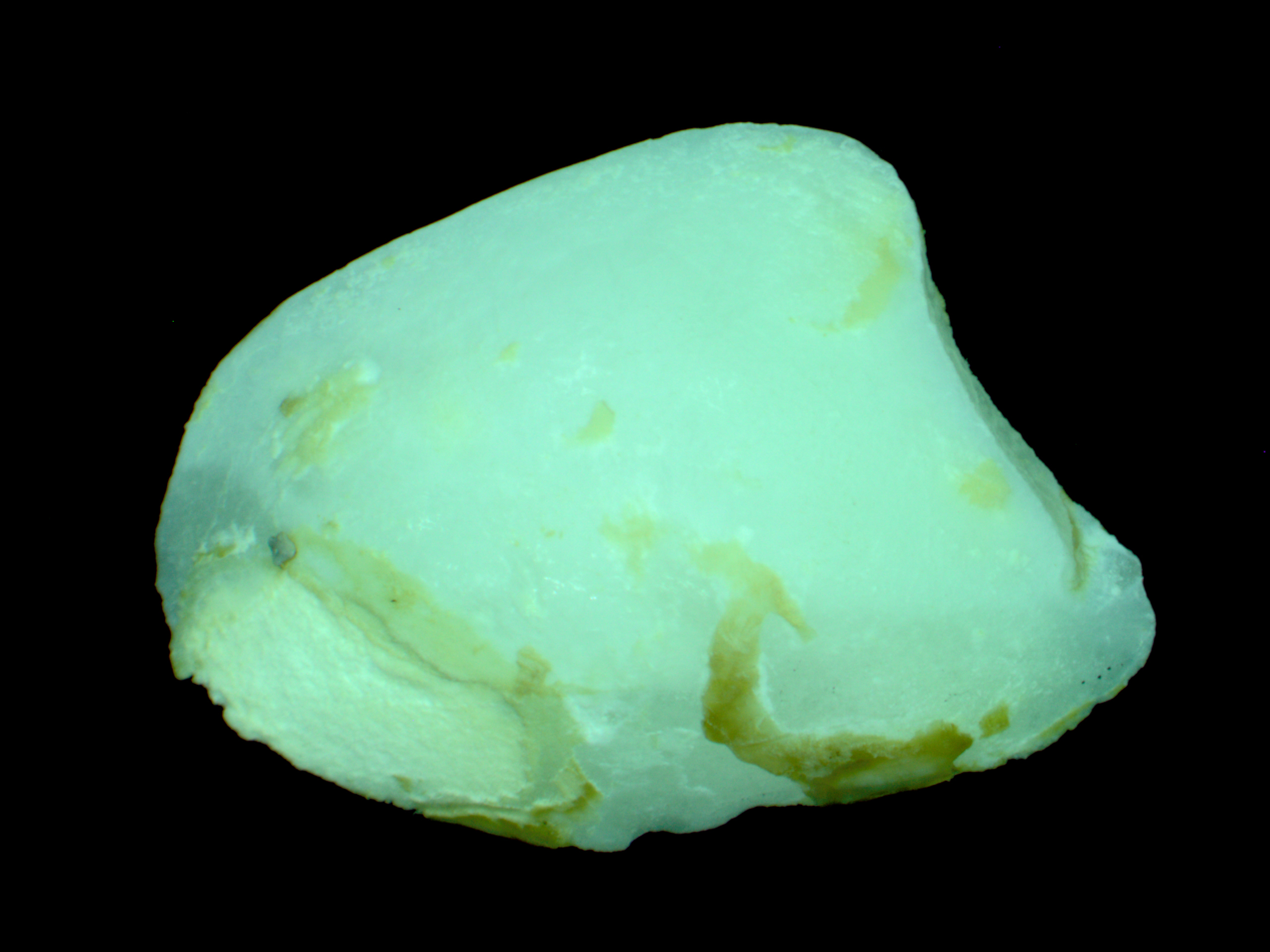

Supplement: Supplemental Information 3 [file peerj-04-1664-s003.zip › CryTru/tetsing/ARI673_R1.jpg]

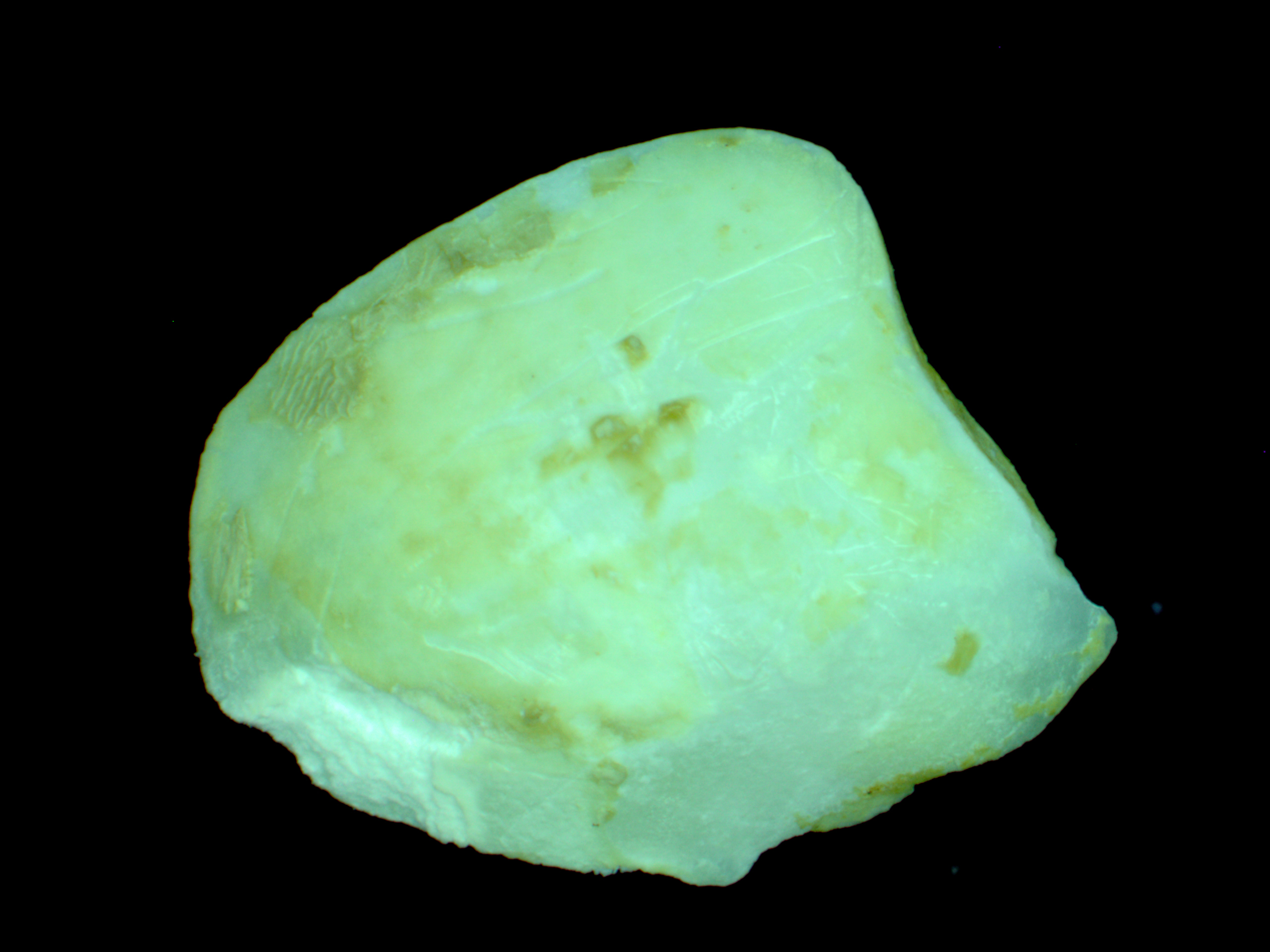

Supplement: Supplemental Information 3 [file peerj-04-1664-s003.zip › CryTru/tetsing/ARI674_R1.jpg]

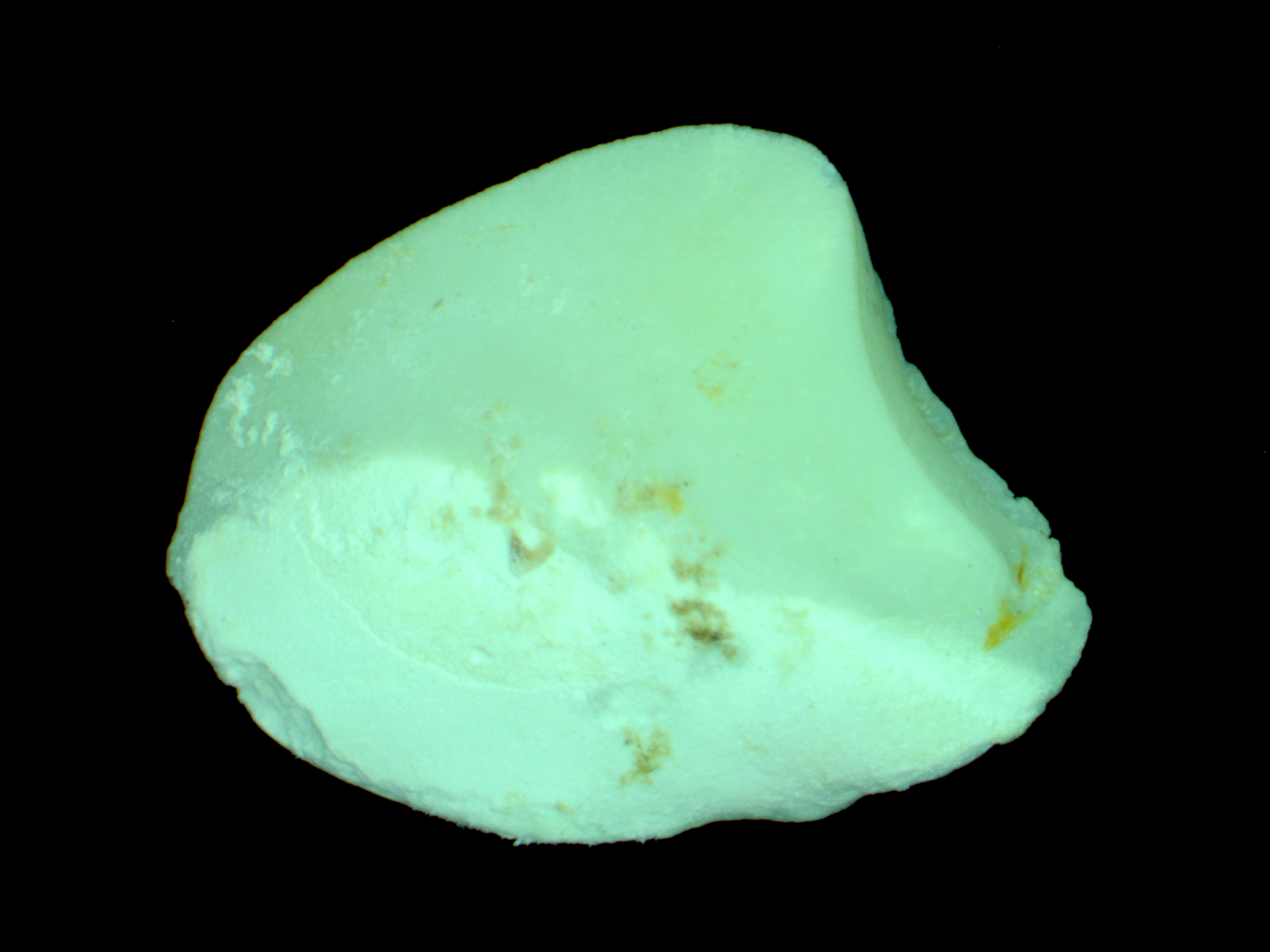

Supplement: Supplemental Information 3 [file peerj-04-1664-s003.zip › CryTru/tetsing/ARI676_R1.jpg]

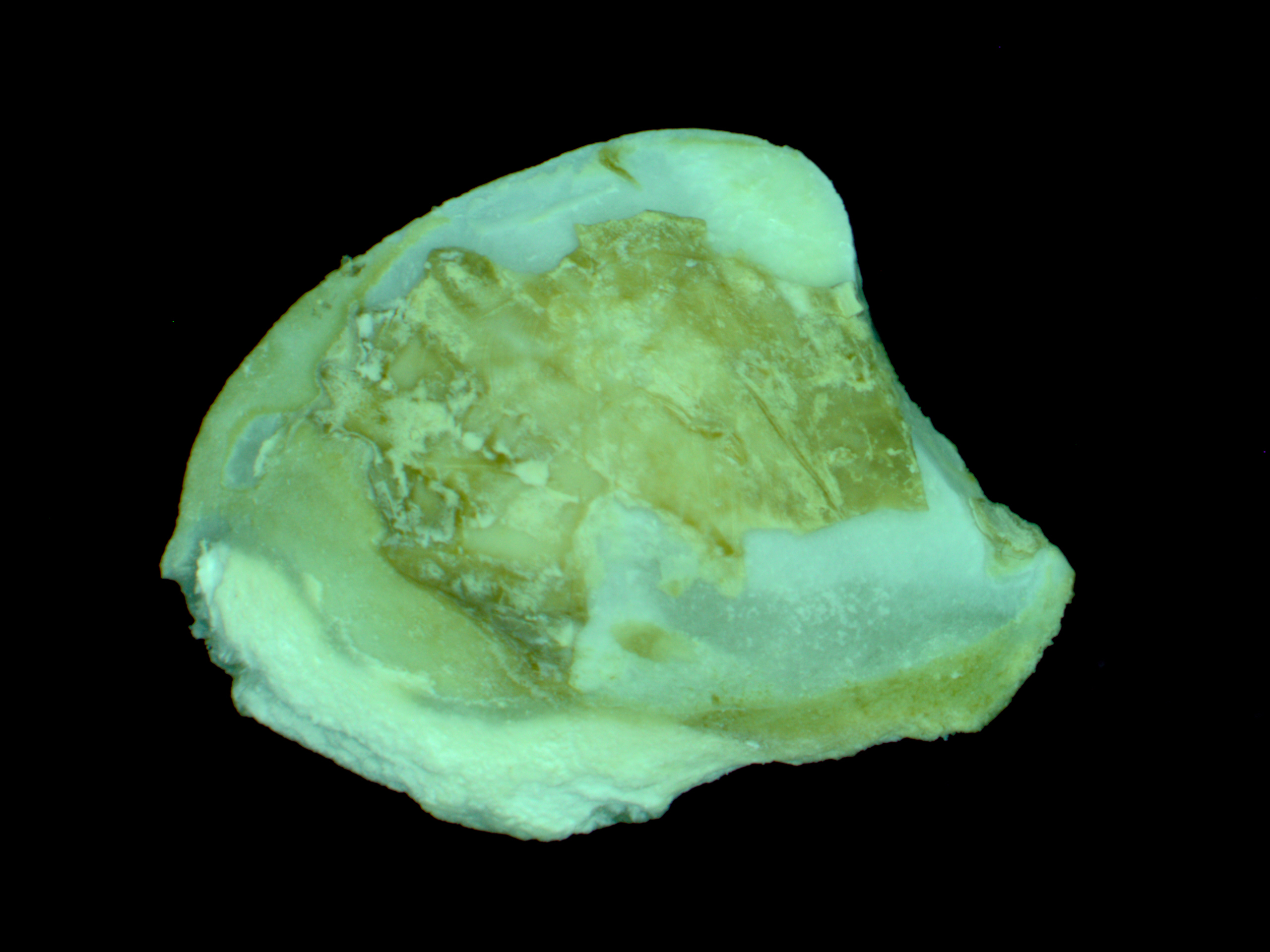

Supplement: Supplemental Information 3 [file peerj-04-1664-s003.zip › CryTru/tetsing/ARI677_R1.jpg]

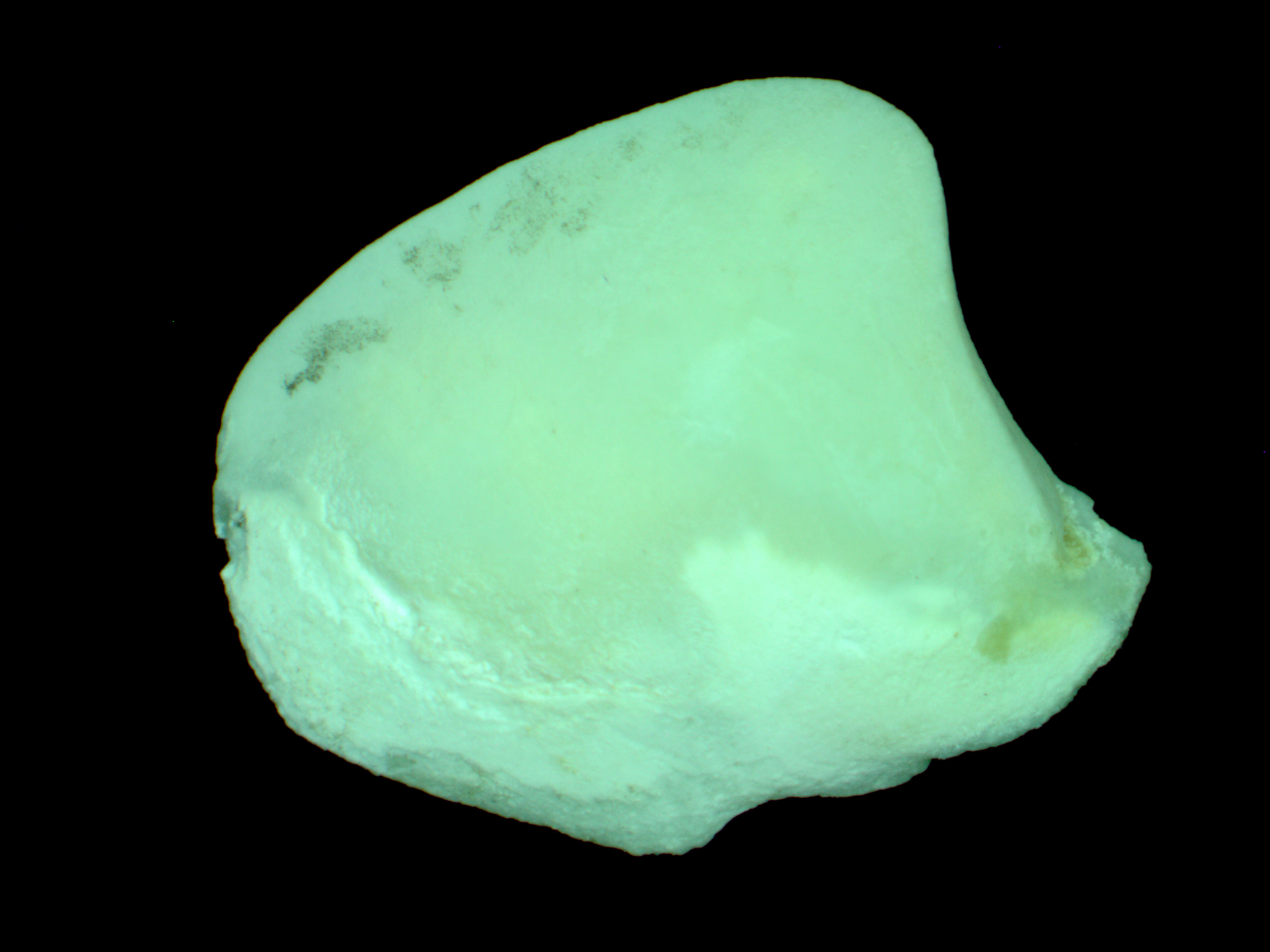

Supplement: Supplemental Information 3 [file peerj-04-1664-s003.zip › CryTru/tetsing/ARI678_R1.jpg]

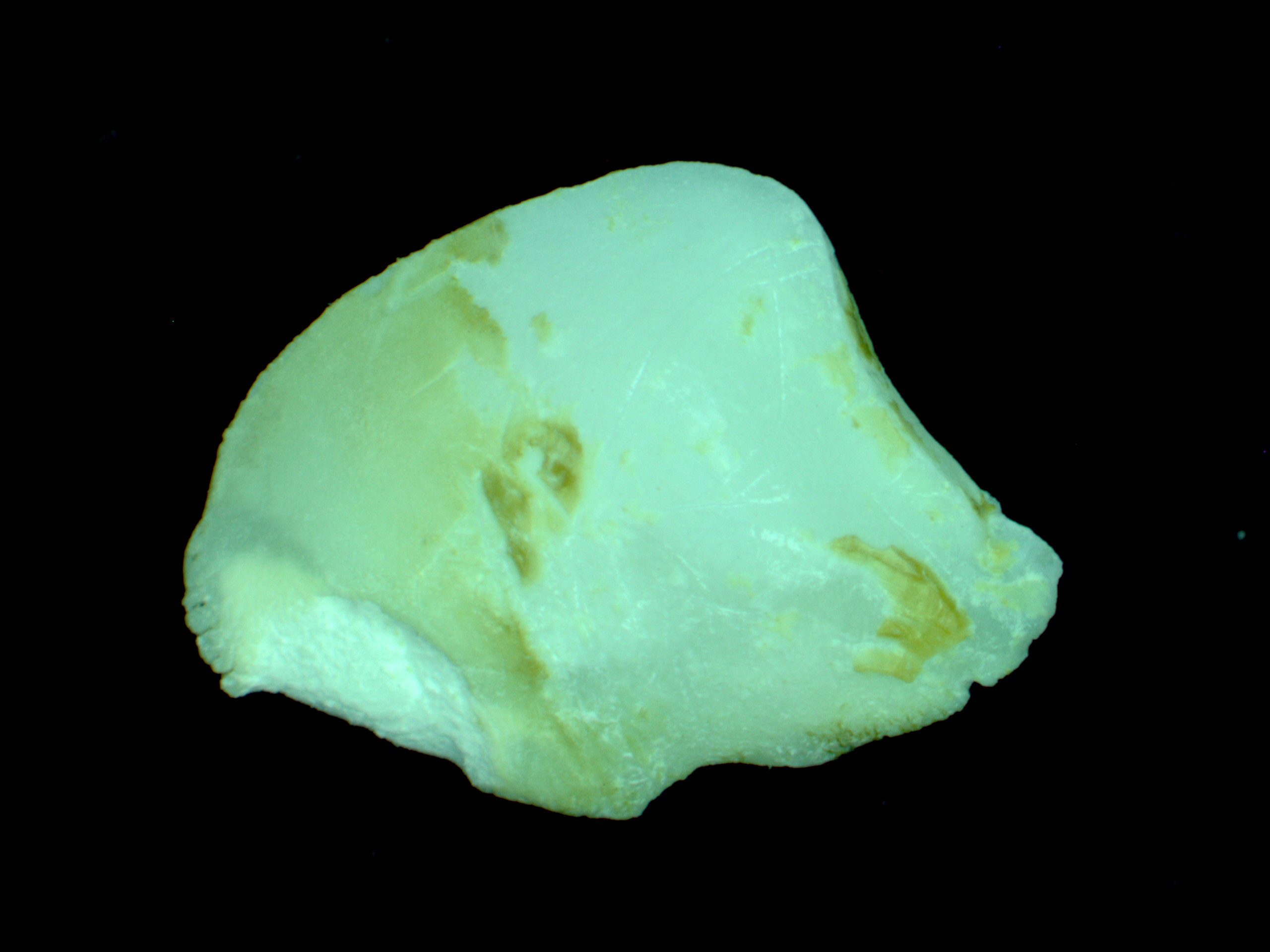

Supplement: Supplemental Information 3 [file peerj-04-1664-s003.zip › CryTru/tetsing/ARI679_R1.jpg]

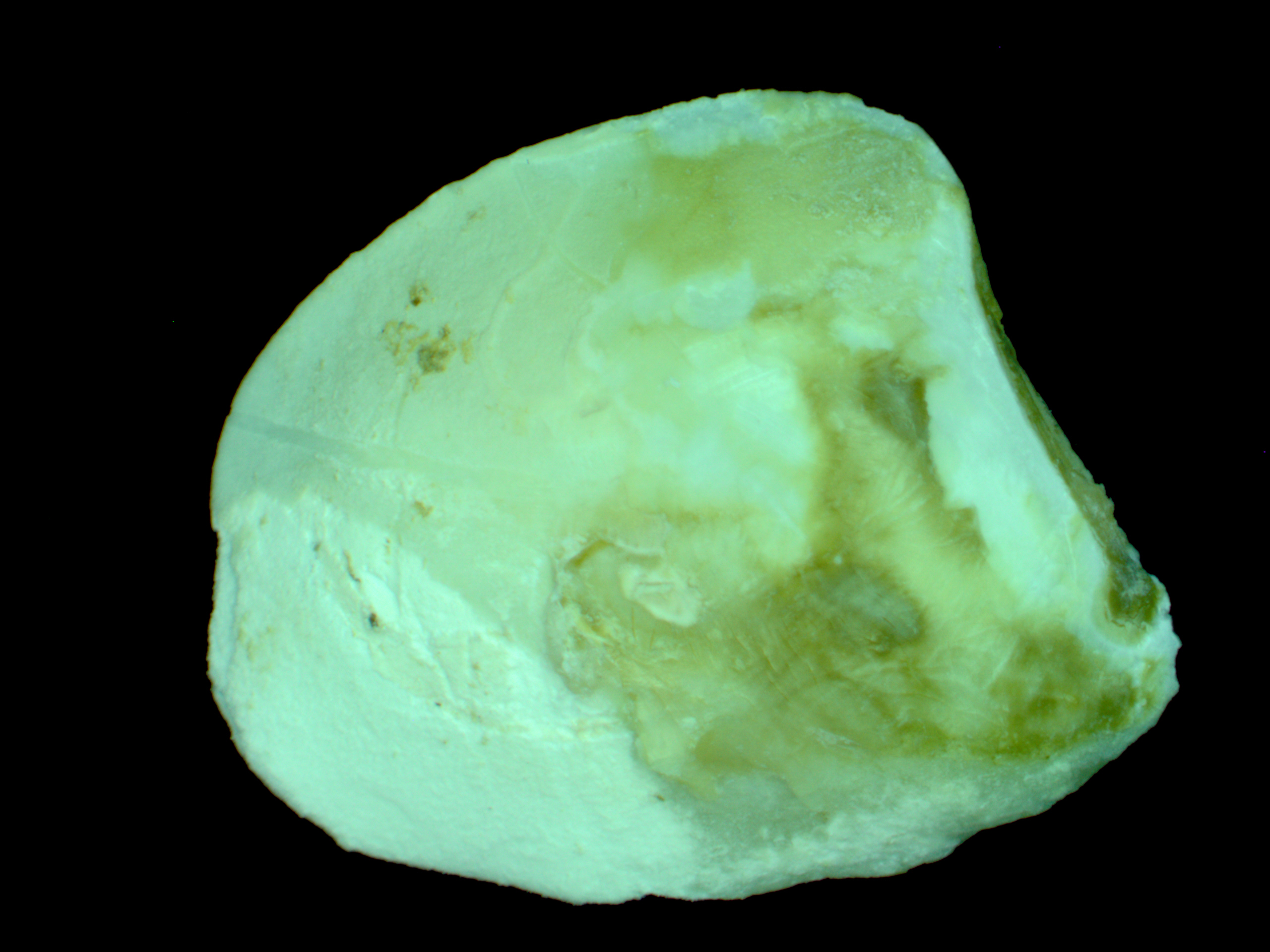

Supplement: Supplemental Information 3 [file peerj-04-1664-s003.zip › CryTru/tetsing/ARI681_R1.jpg]

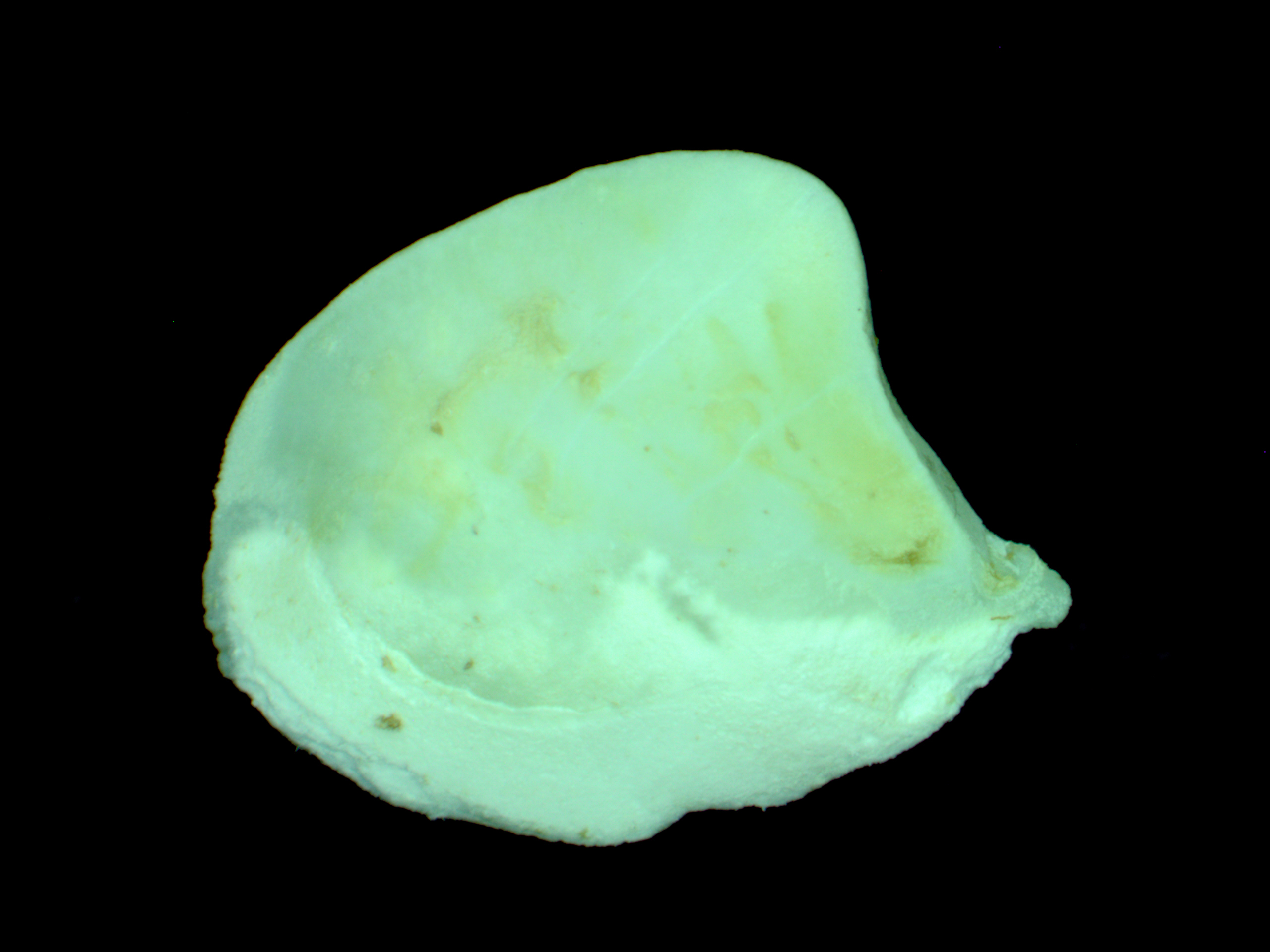

Supplement: Supplemental Information 3 [file peerj-04-1664-s003.zip › CryTru/tetsing/ARI683_R1.jpg]

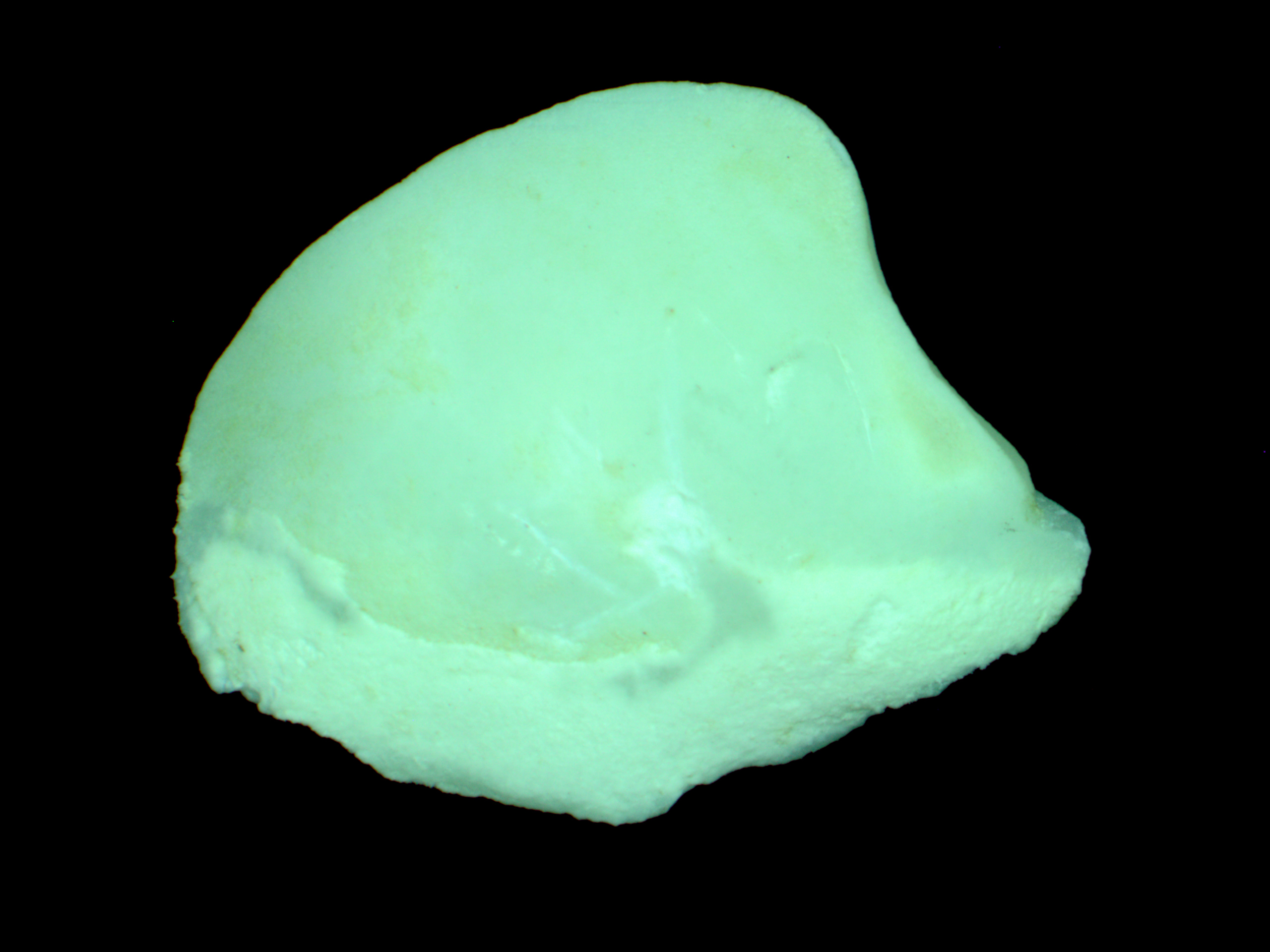

Supplement: Supplemental Information 3 [file peerj-04-1664-s003.zip › CryTru/training/ARI38_R1.jpg]

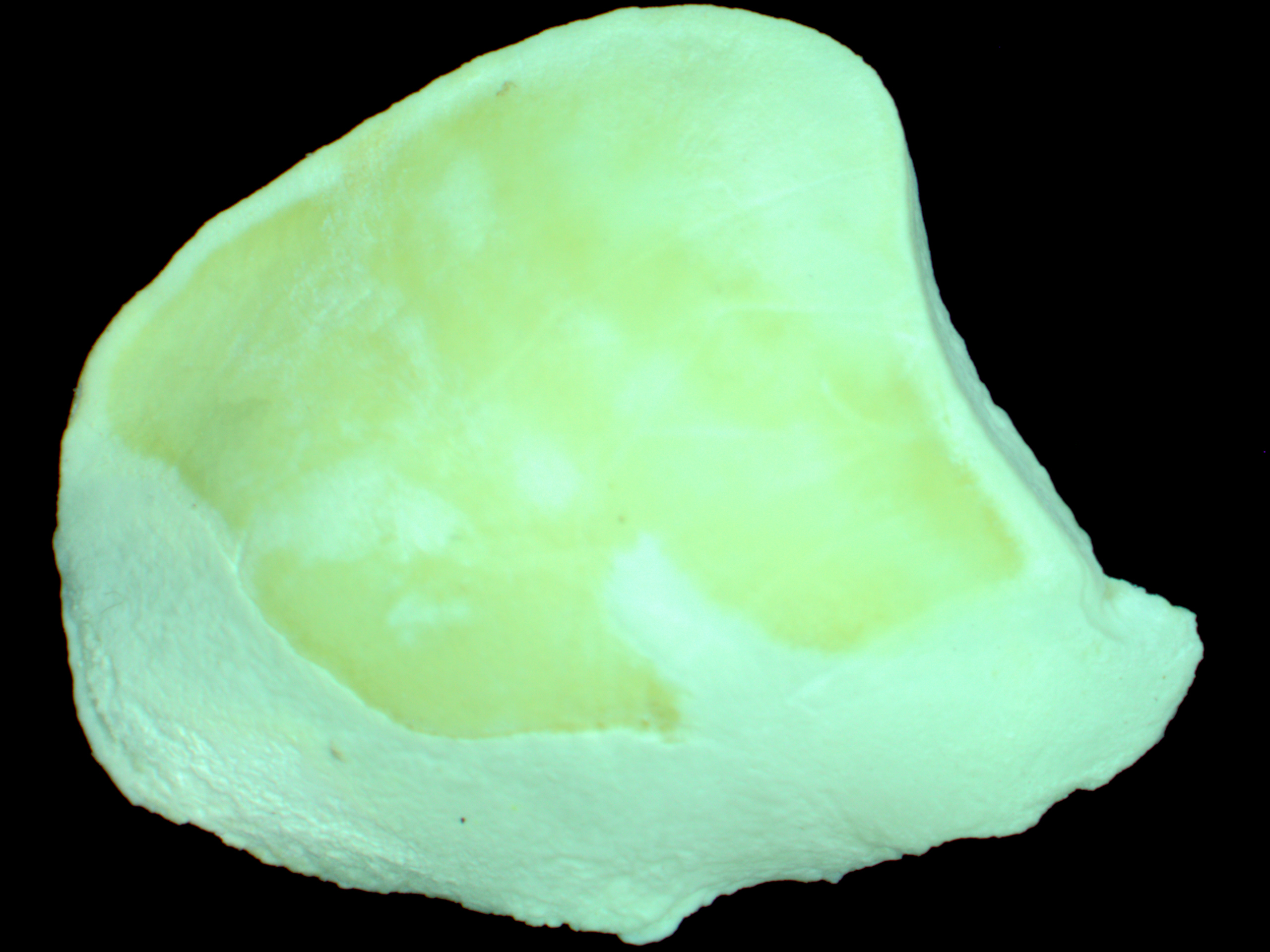

Supplement: Supplemental Information 3 [file peerj-04-1664-s003.zip › CryTru/training/ARI542_R1.jpg]

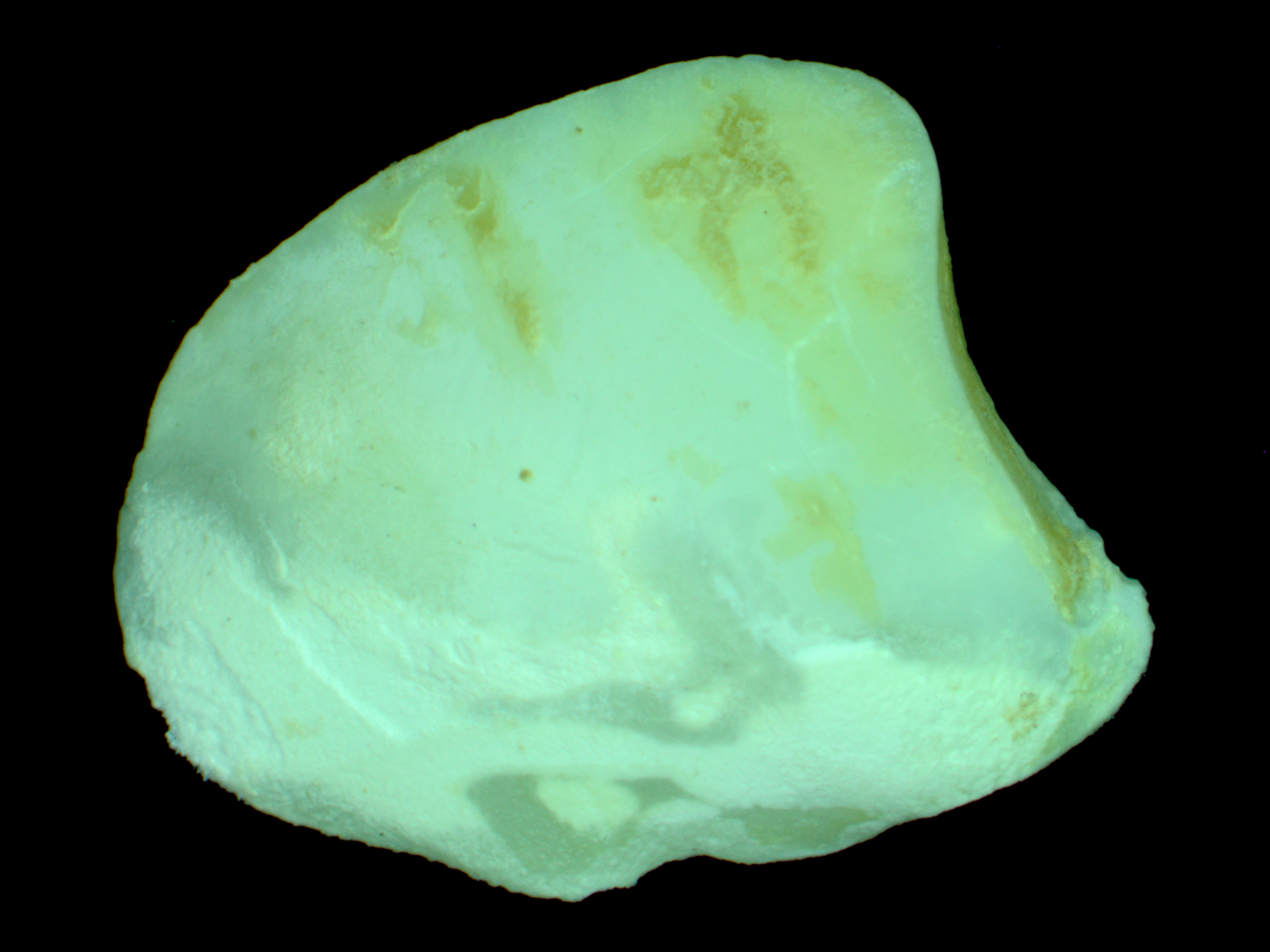

Supplement: Supplemental Information 3 [file peerj-04-1664-s003.zip › CryTru/training/ARI645_R1.jpg]

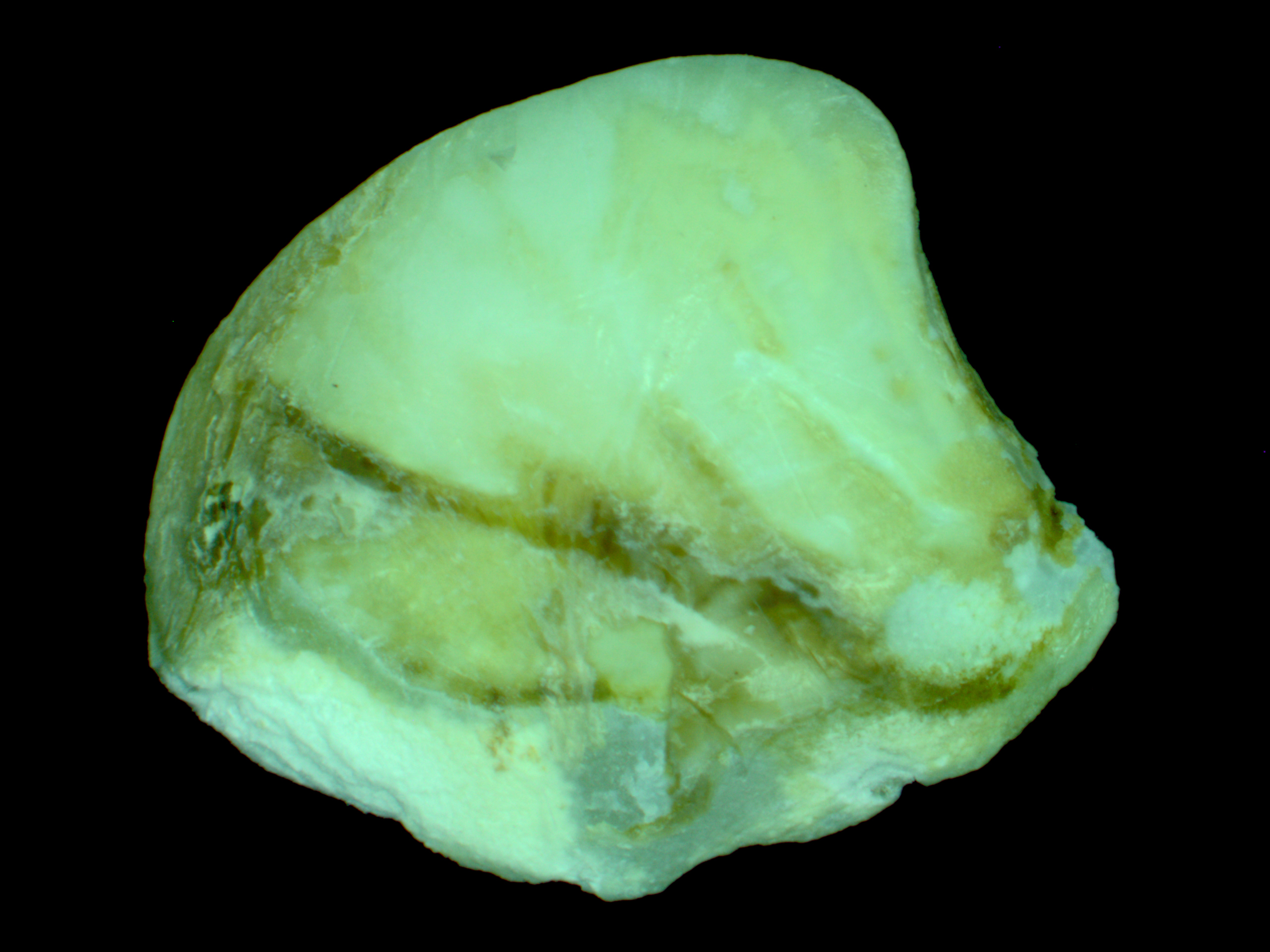

Supplement: Supplemental Information 3 [file peerj-04-1664-s003.zip › CryTru/training/ARI646_R1.jpg]

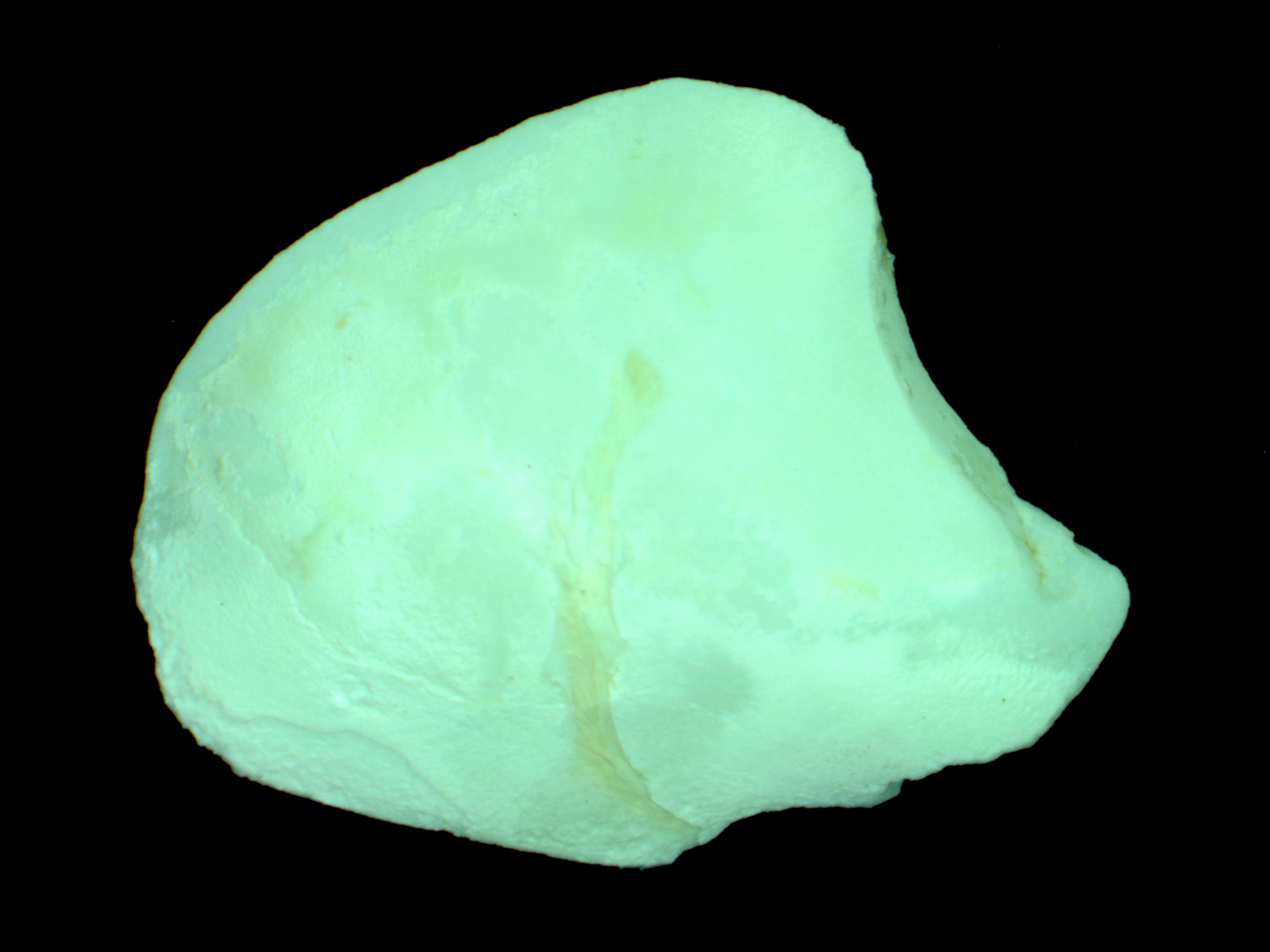

Supplement: Supplemental Information 3 [file peerj-04-1664-s003.zip › CryTru/training/ARI647_R1.jpg]

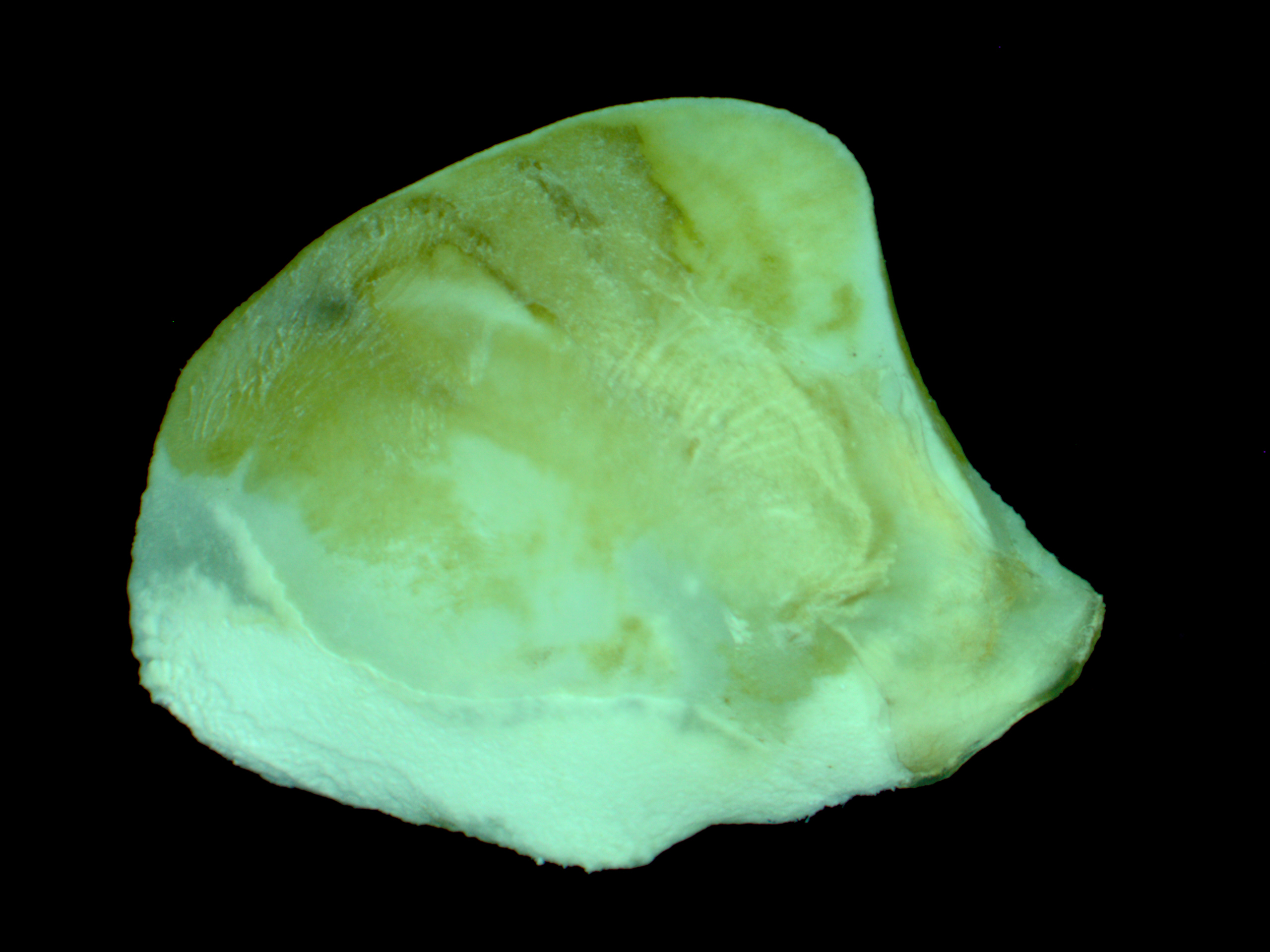

Supplement: Supplemental Information 3 [file peerj-04-1664-s003.zip › CryTru/training/ARI648_R1.jpg]

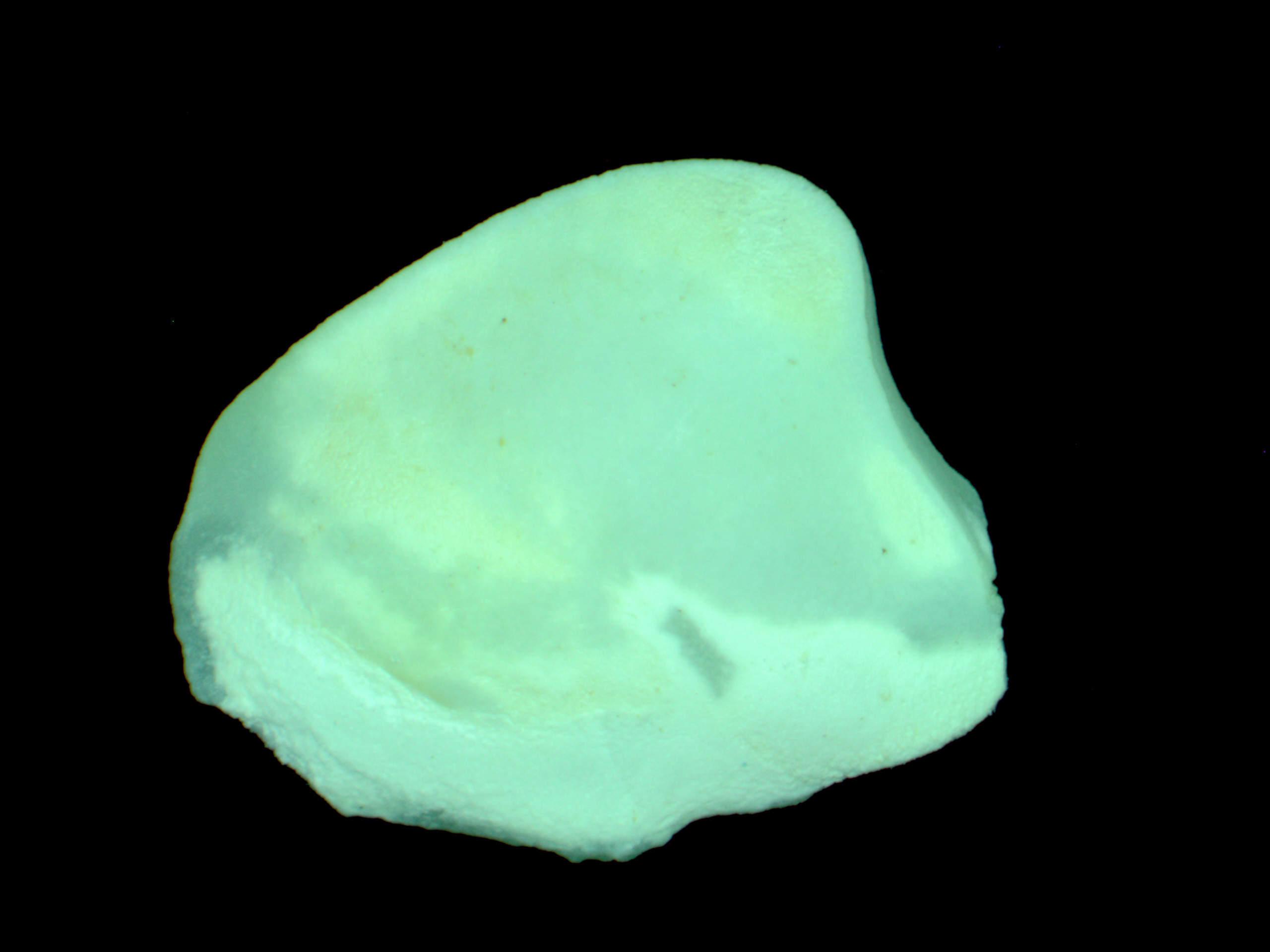

Supplement: Supplemental Information 3 [file peerj-04-1664-s003.zip › CryTru/training/ARI650_R1.jpg]

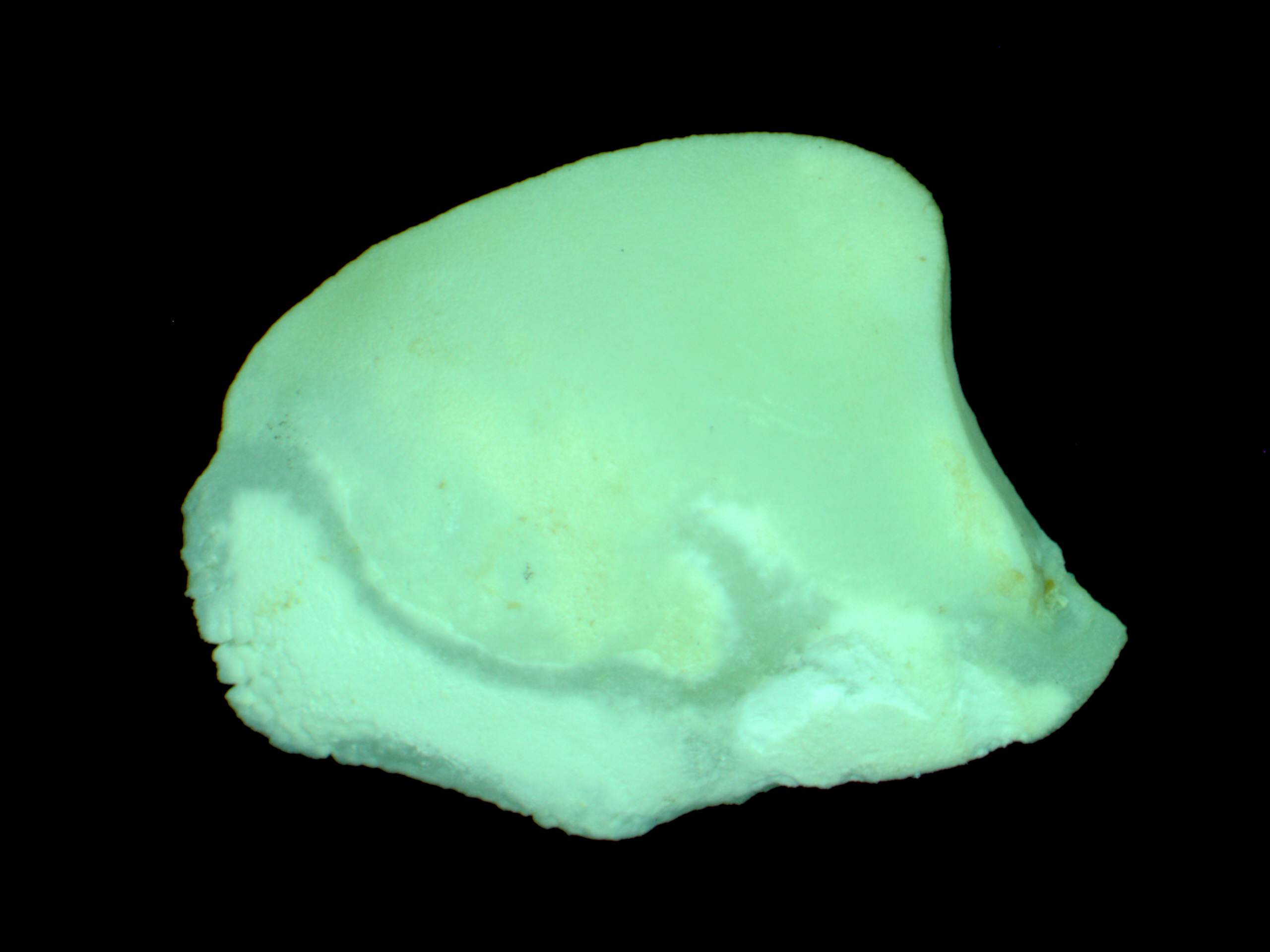

Supplement: Supplemental Information 3 [file peerj-04-1664-s003.zip › CryTru/training/ARI651_R1.jpg]

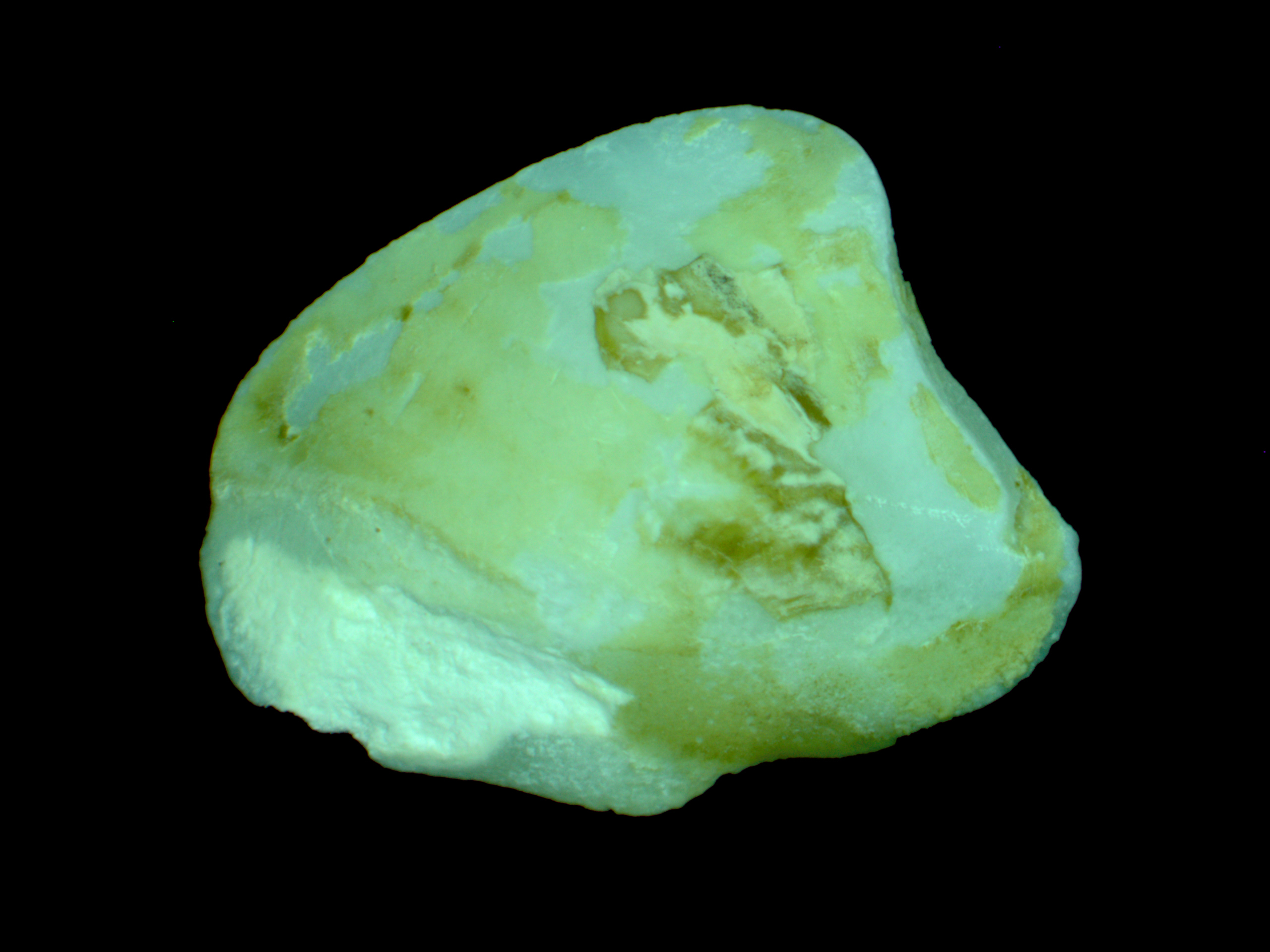

Supplement: Supplemental Information 3 [file peerj-04-1664-s003.zip › CryTru/training/ARI653_R1.jpg]

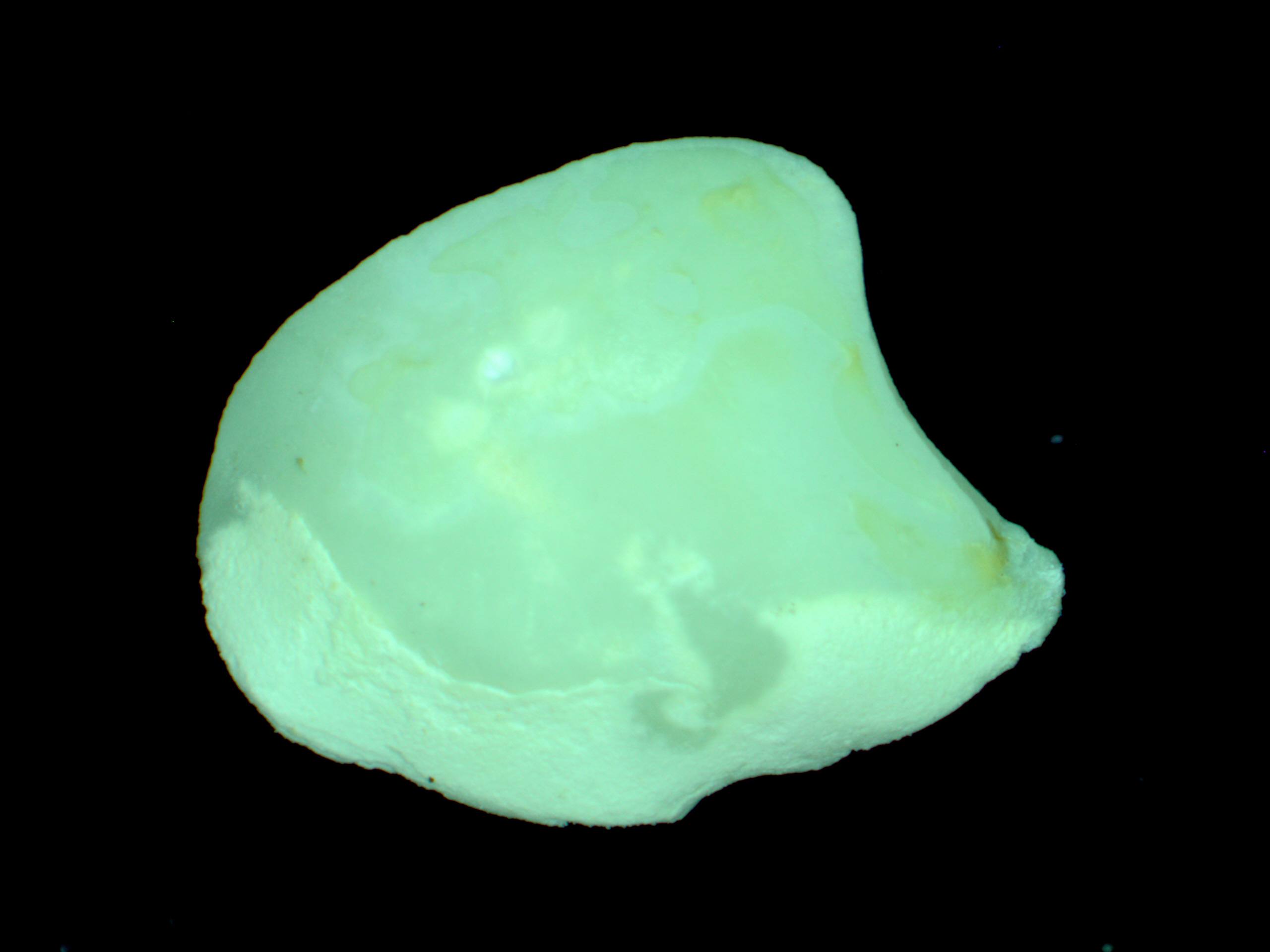

Supplement: Supplemental Information 3 [file peerj-04-1664-s003.zip › CryTru/training/ARI654_R1.jpg]

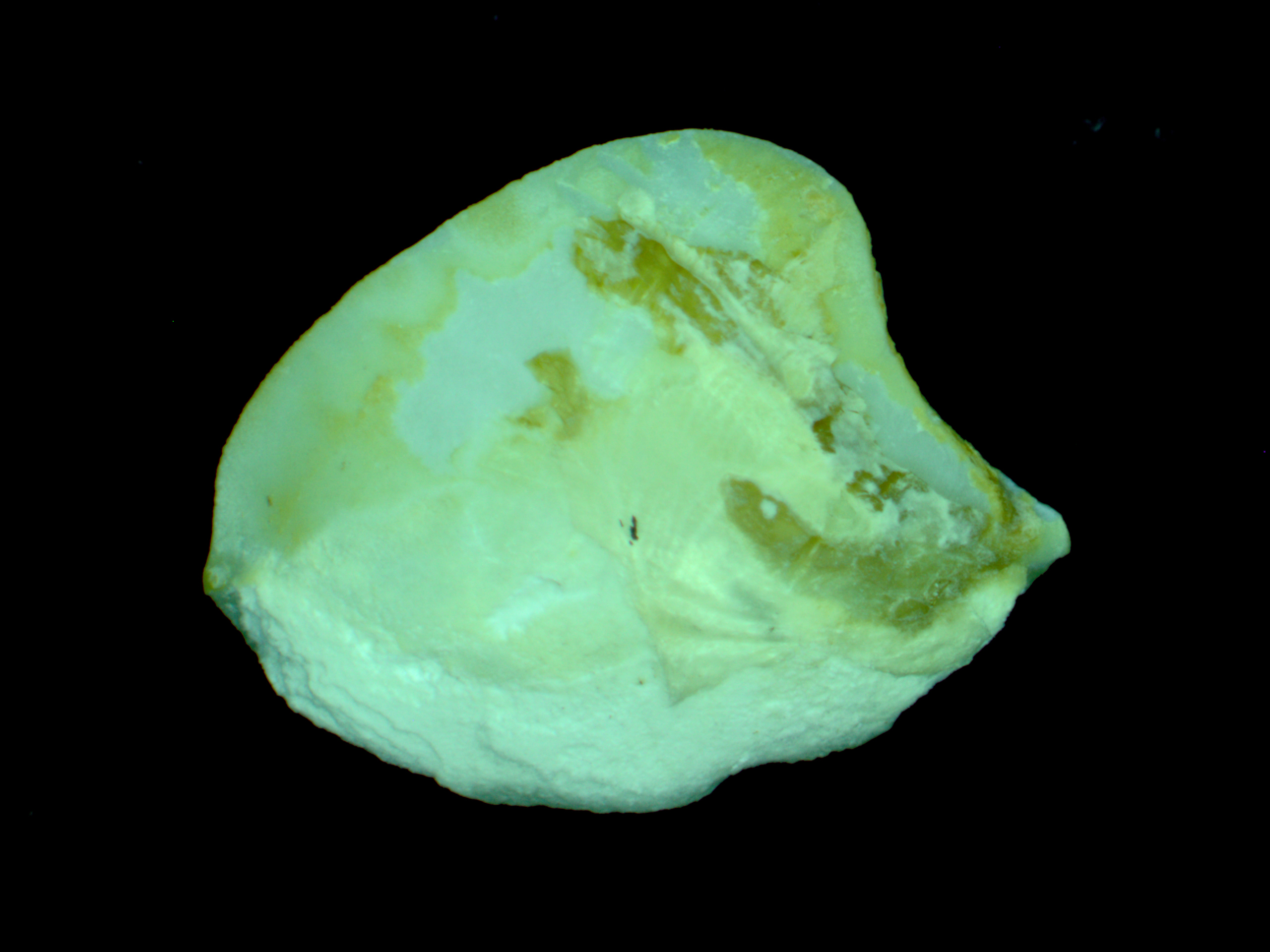

Supplement: Supplemental Information 3 [file peerj-04-1664-s003.zip › CryTru/training/ARI656_R1.jpg]

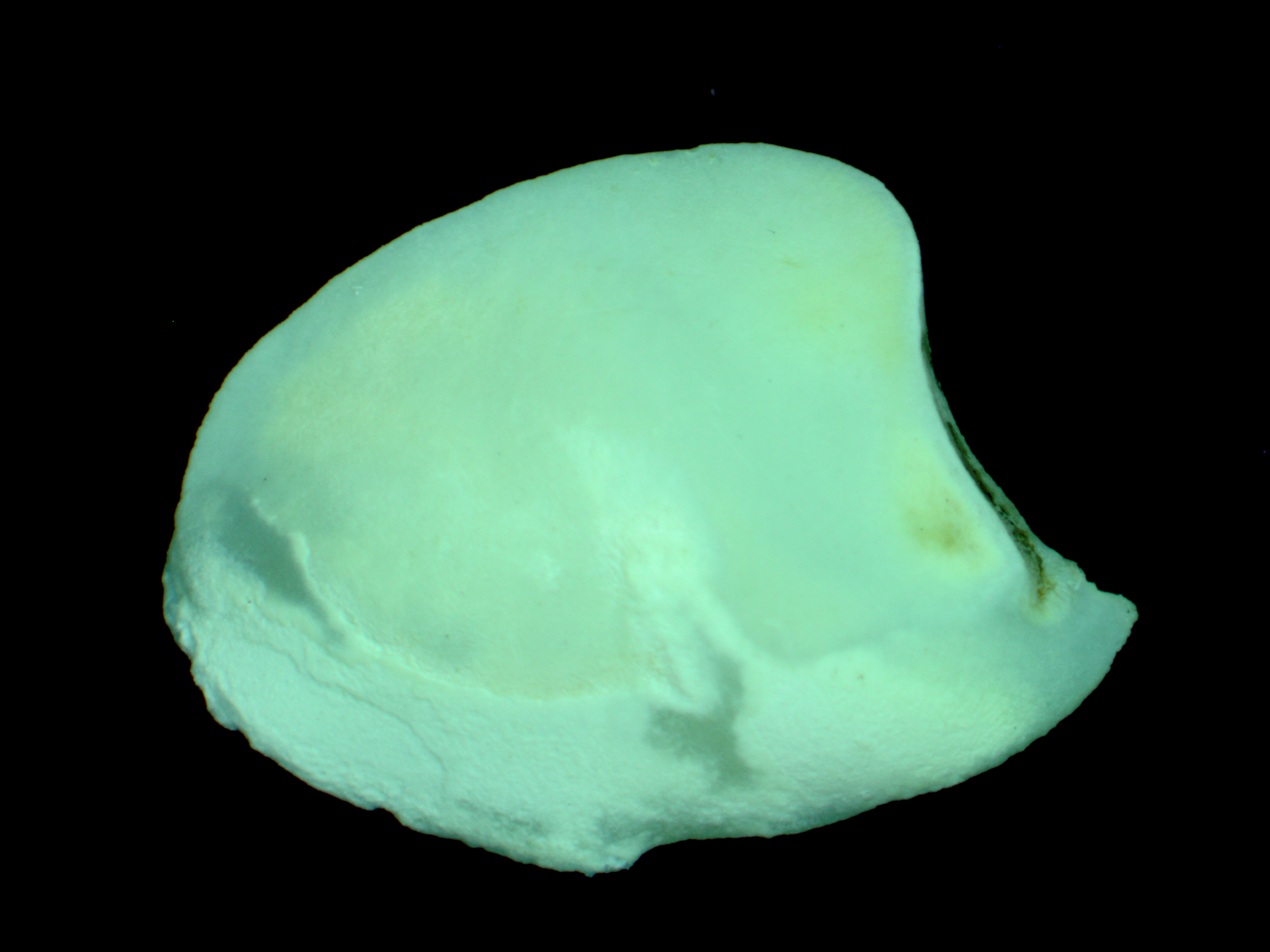

Supplement: Supplemental Information 3 [file peerj-04-1664-s003.zip › CryTru/training/ARI657_R1.jpg]

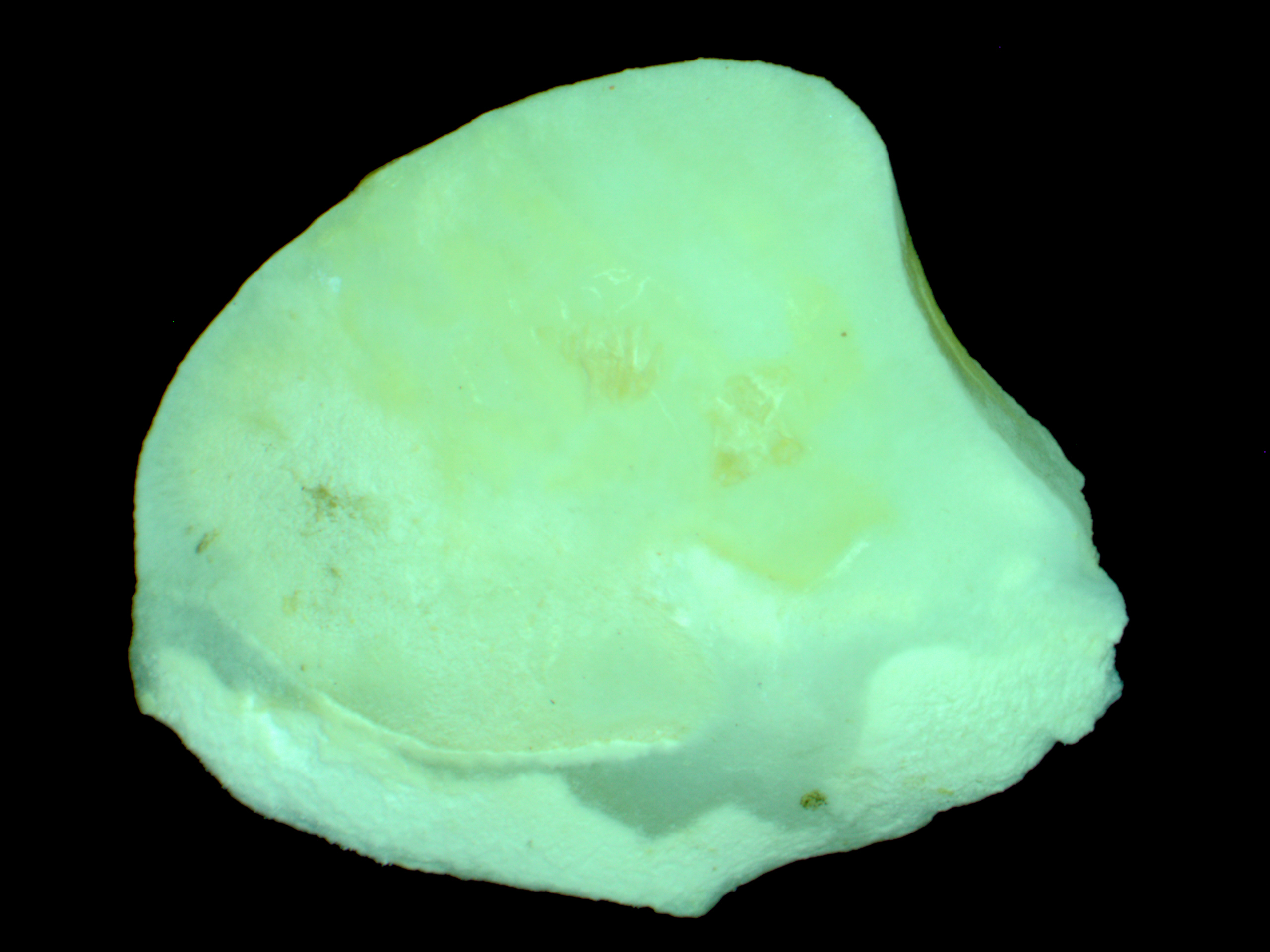

Supplement: Supplemental Information 3 [file peerj-04-1664-s003.zip › CryTru/training/ARI660_R1.jpg]

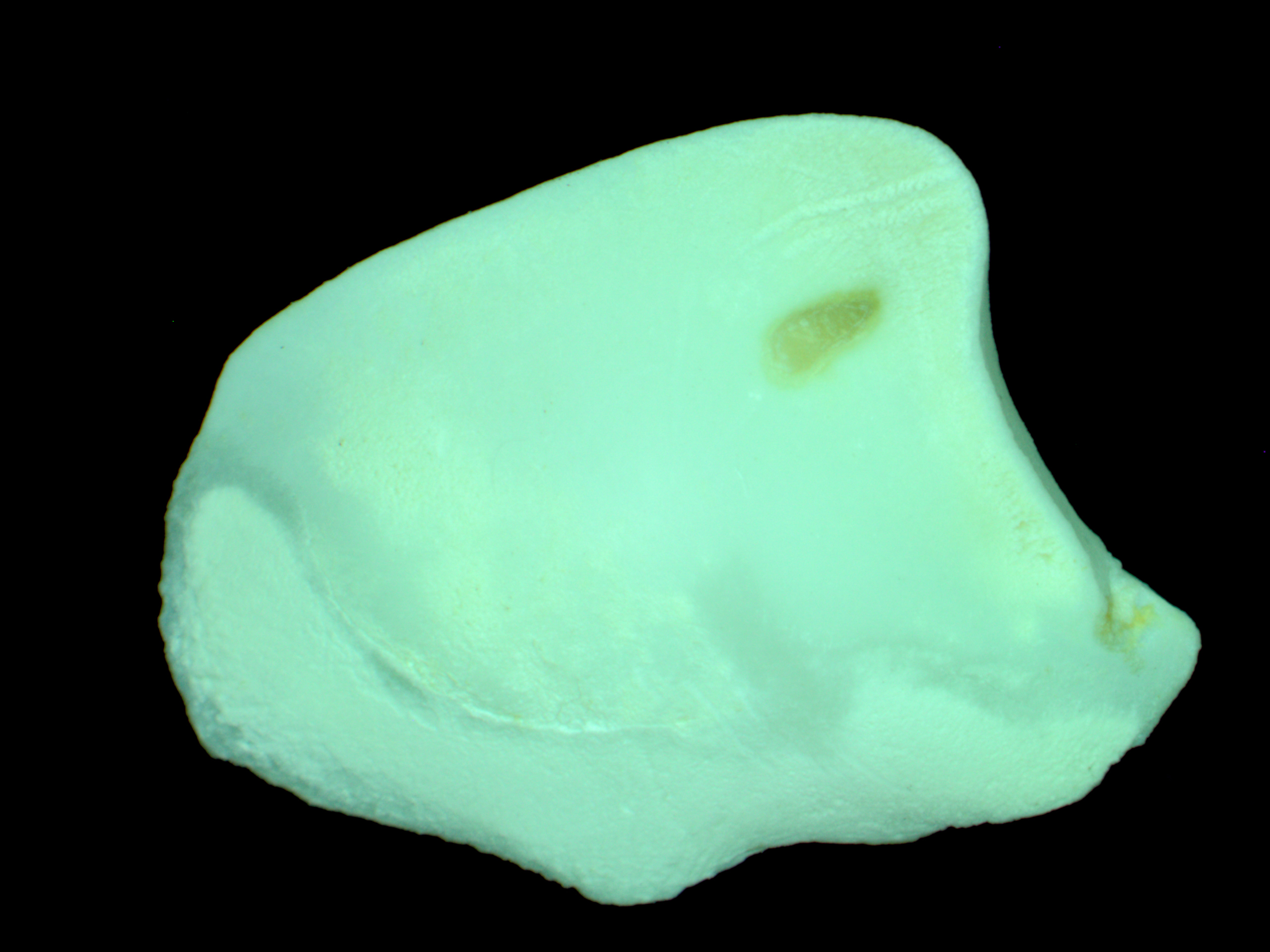

Supplement: Supplemental Information 3 [file peerj-04-1664-s003.zip › CryTru/training/ARI661_R1.jpg]

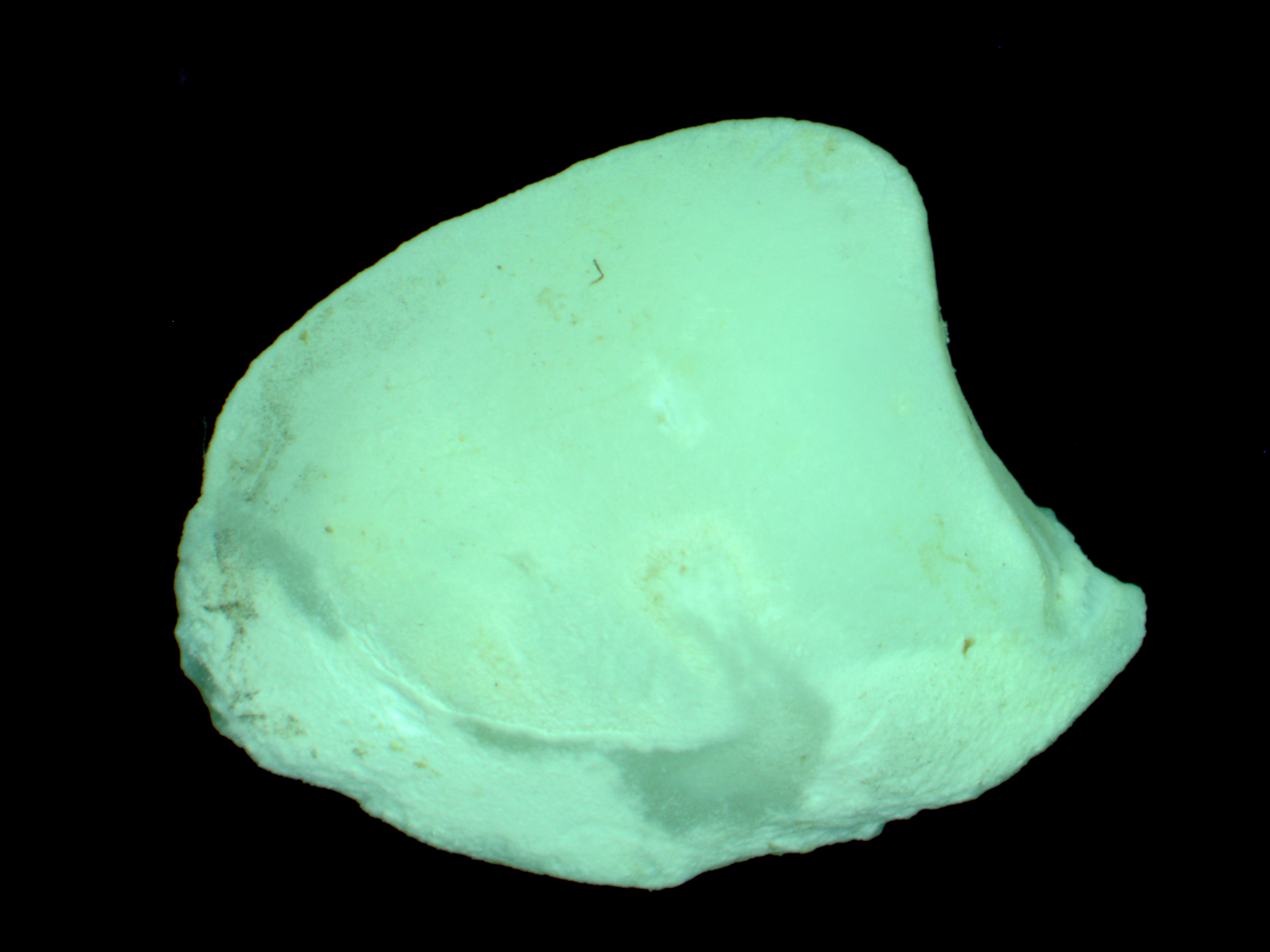

Supplement: Supplemental Information 3 [file peerj-04-1664-s003.zip › CryTru/training/ARI662_R1.jpg]

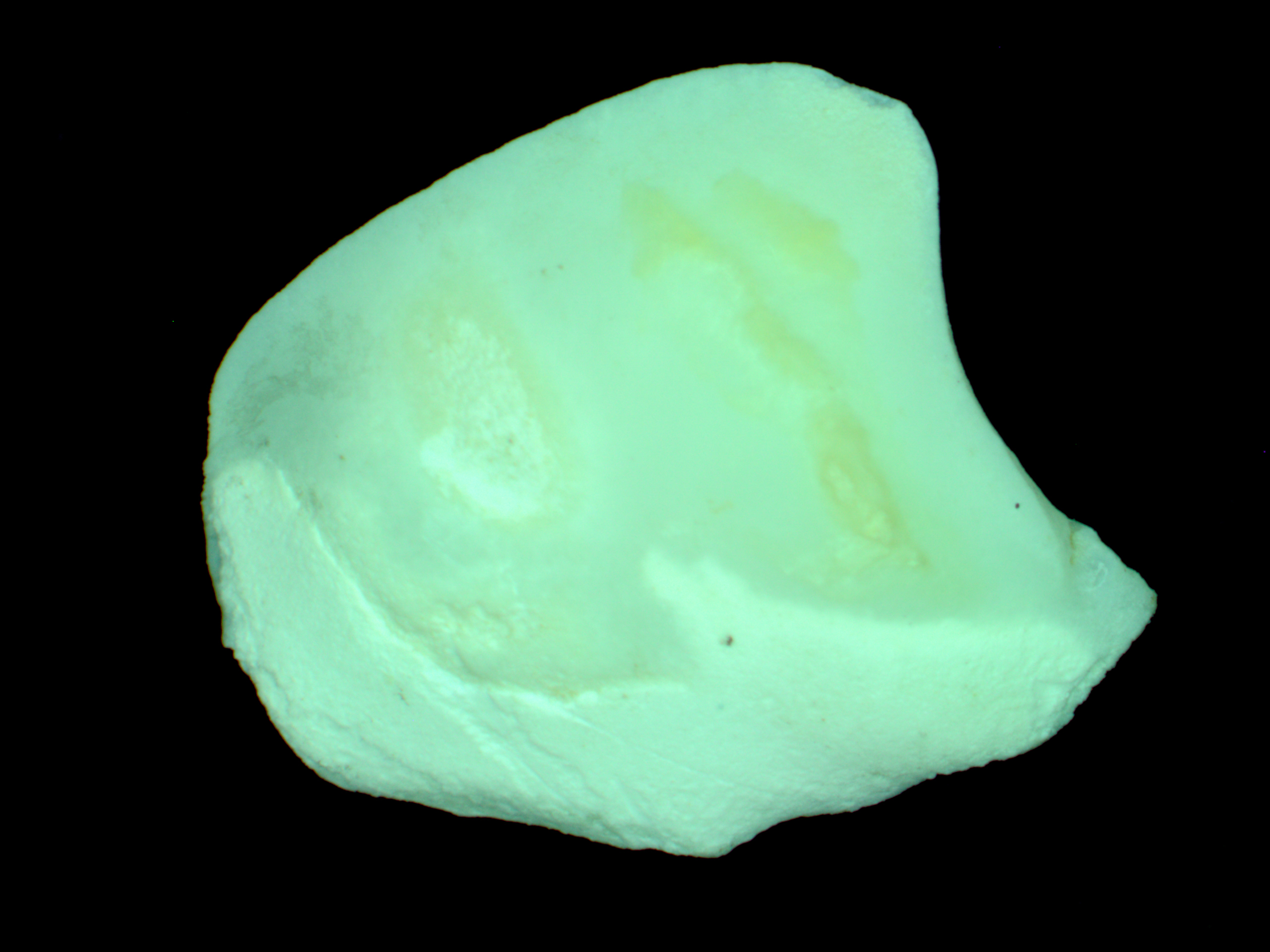

Supplement: Supplemental Information 3 [file peerj-04-1664-s003.zip › CryTru/training/ARI664_R1.jpg]

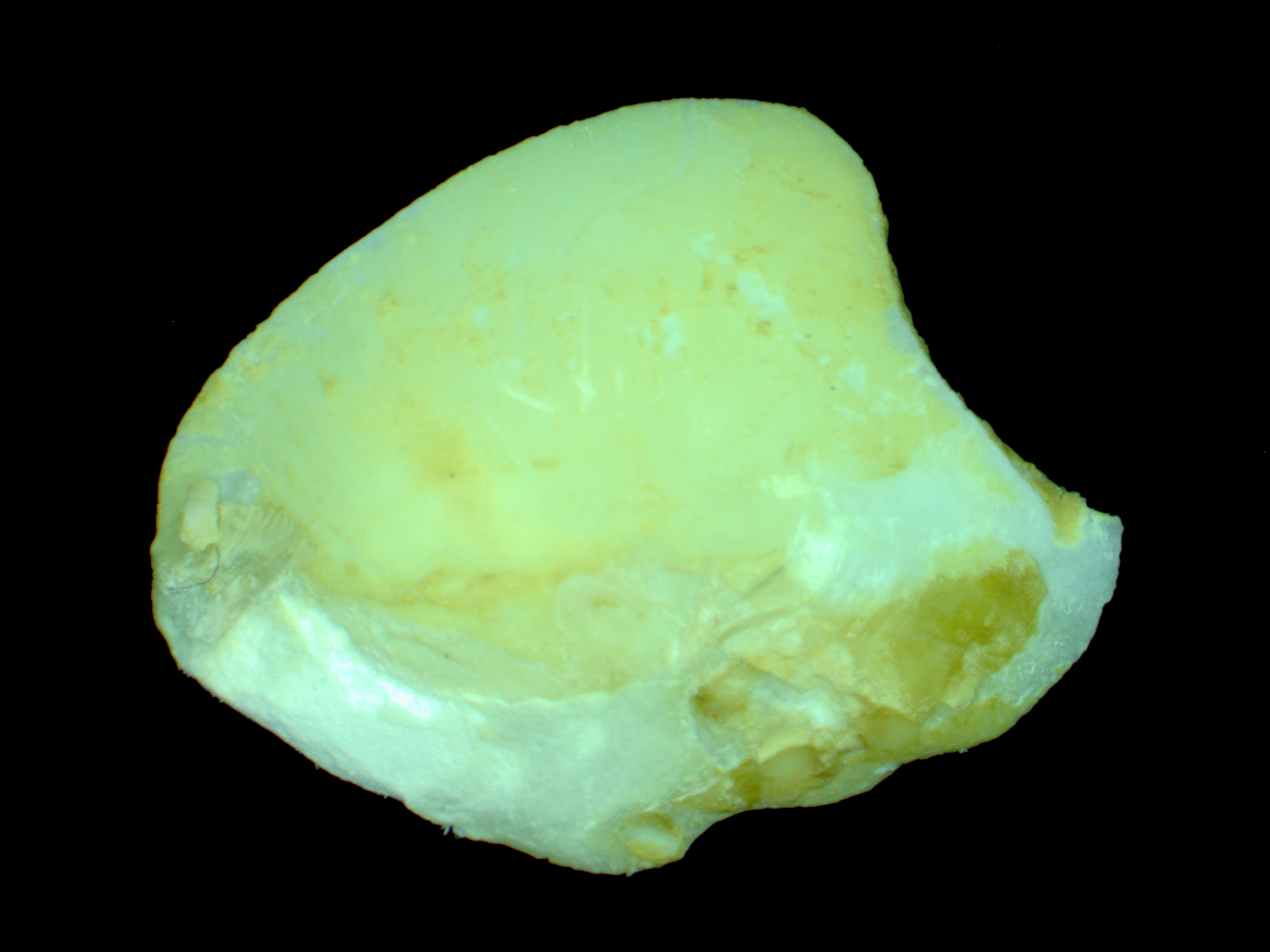

Supplement: Supplemental Information 3 [file peerj-04-1664-s003.zip › CryTru/training/ARI665_R1.jpg]

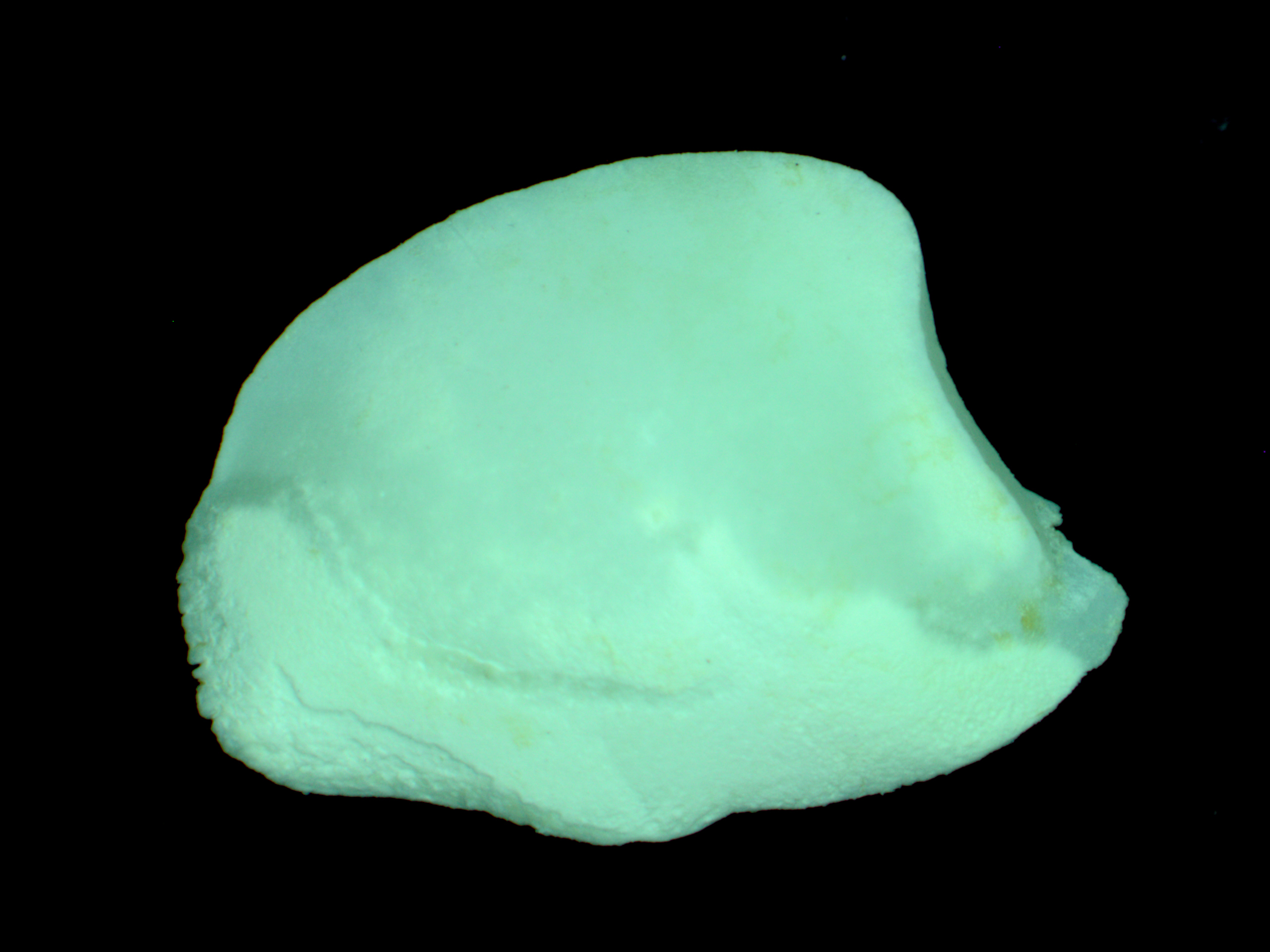

Supplement: Supplemental Information 3 [file peerj-04-1664-s003.zip › CryTru/training/ARI667_R1.jpg]

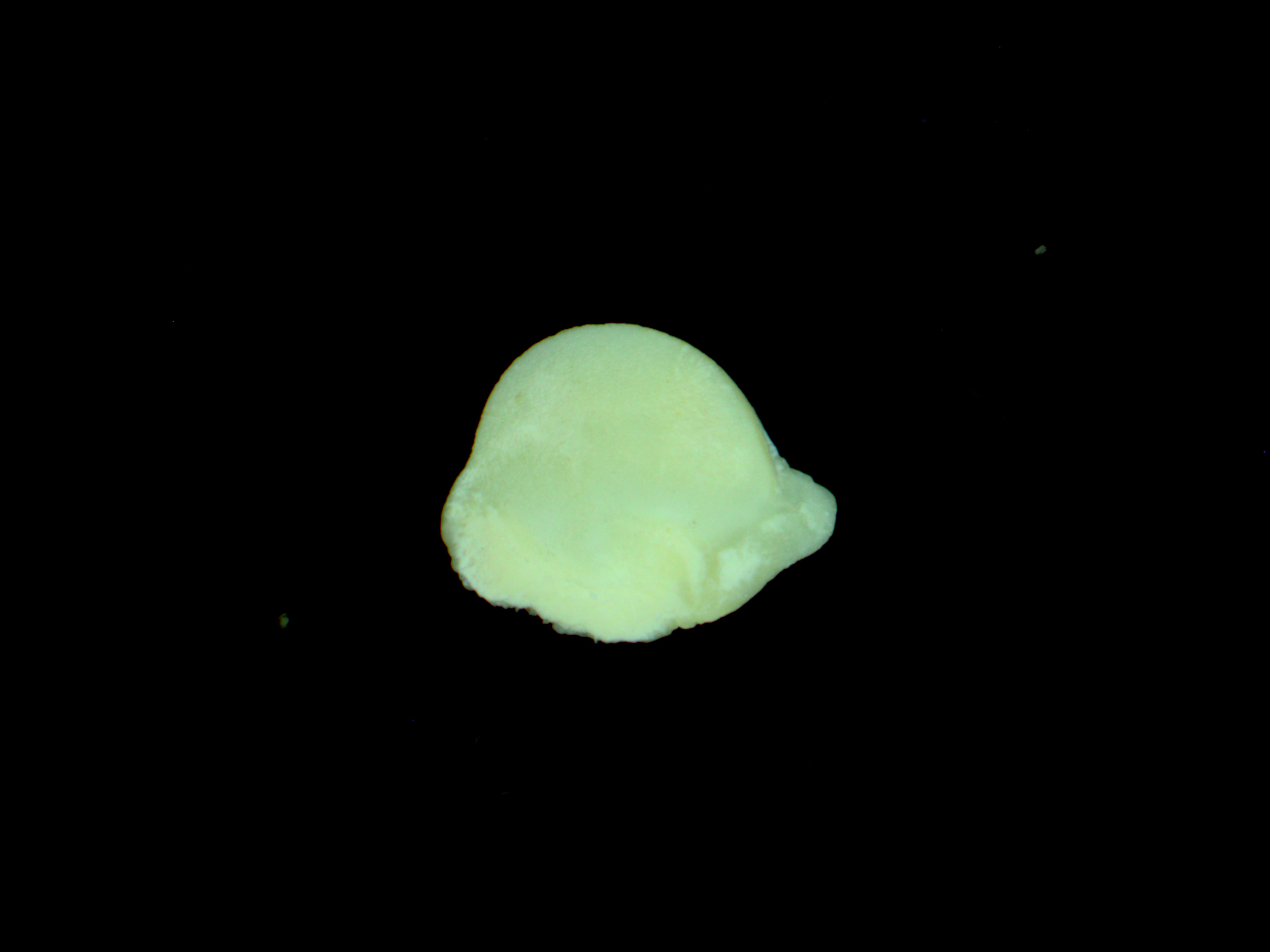

Supplement: Supplemental Information 4 [file peerj-04-1664-s004.zip › HexSag/testing/ARI1003_R1.jpg]

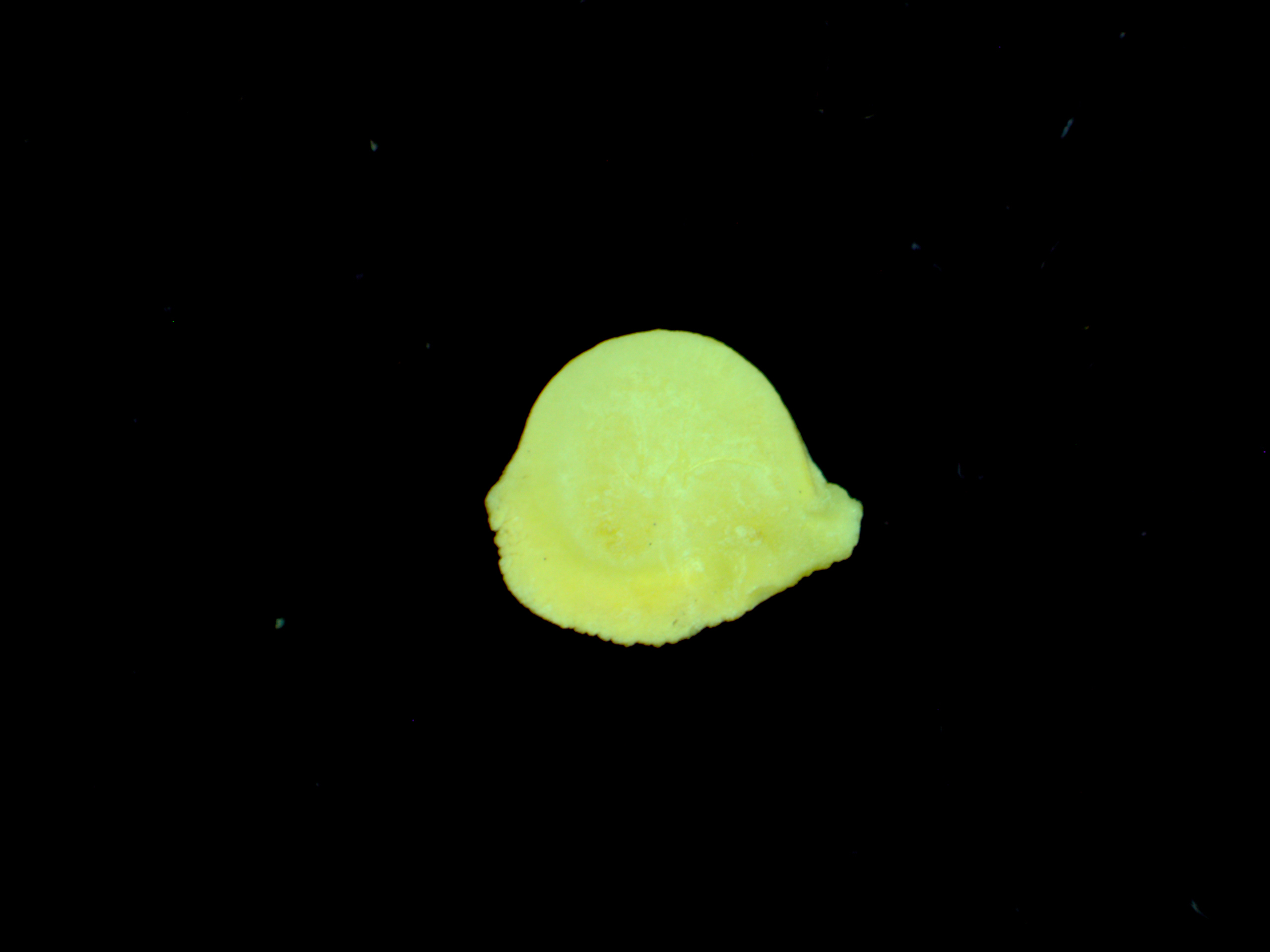

Supplement: Supplemental Information 4 [file peerj-04-1664-s004.zip › HexSag/testing/ARI774_R1.jpg]

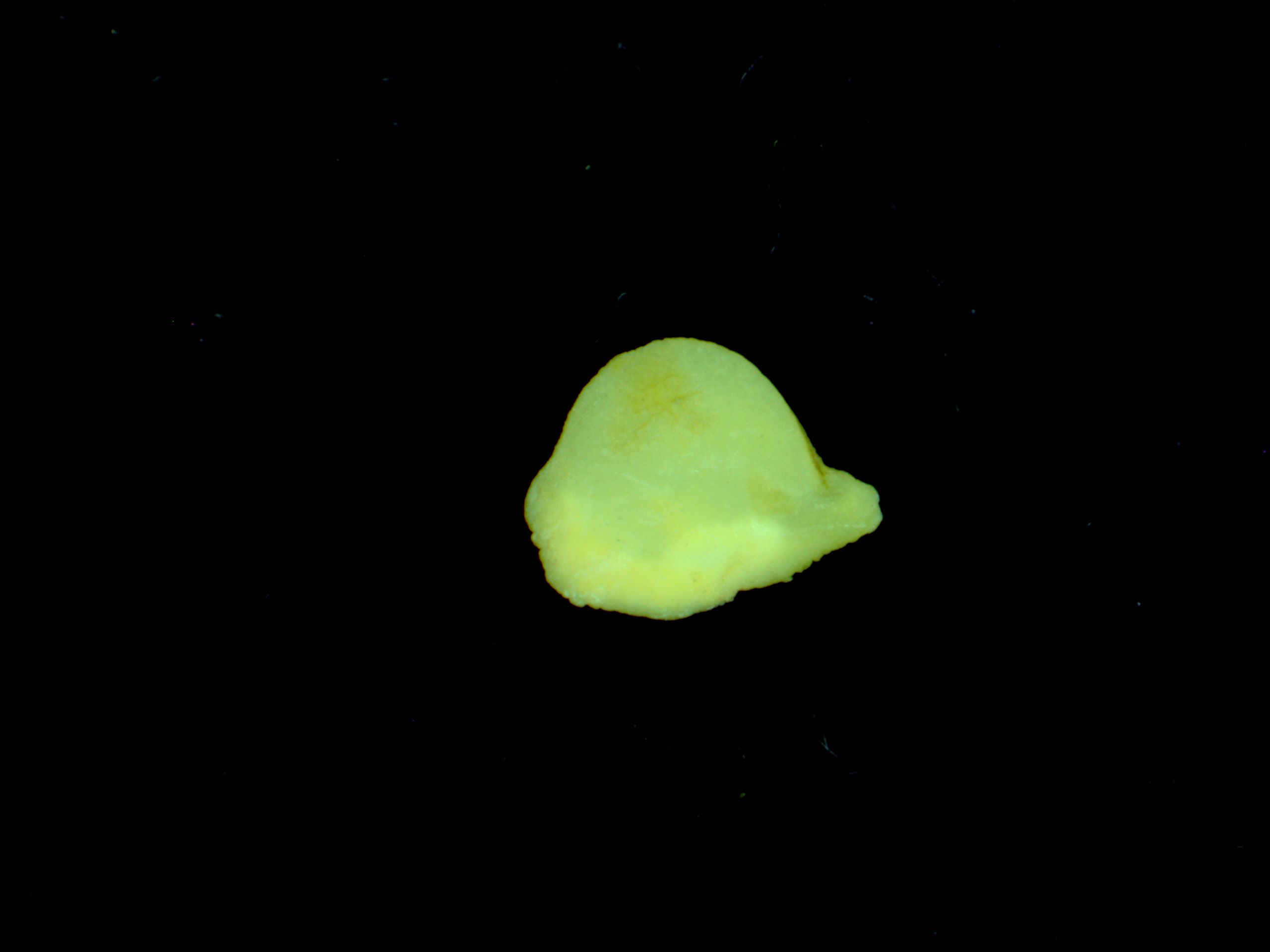

Supplement: Supplemental Information 4 [file peerj-04-1664-s004.zip › HexSag/testing/ARI775_R1.jpg]

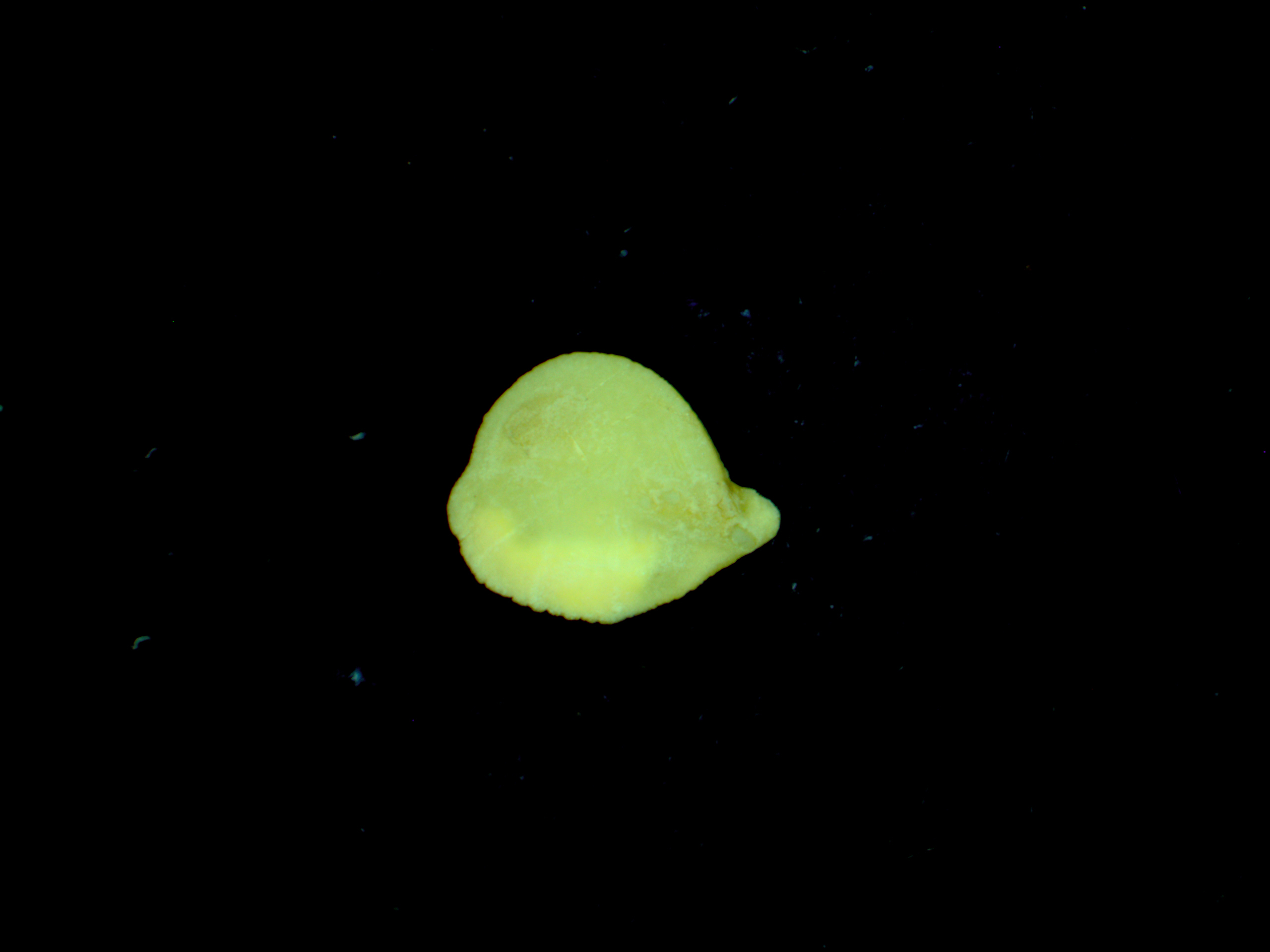

Supplement: Supplemental Information 4 [file peerj-04-1664-s004.zip › HexSag/testing/ARI776_R1.jpg]

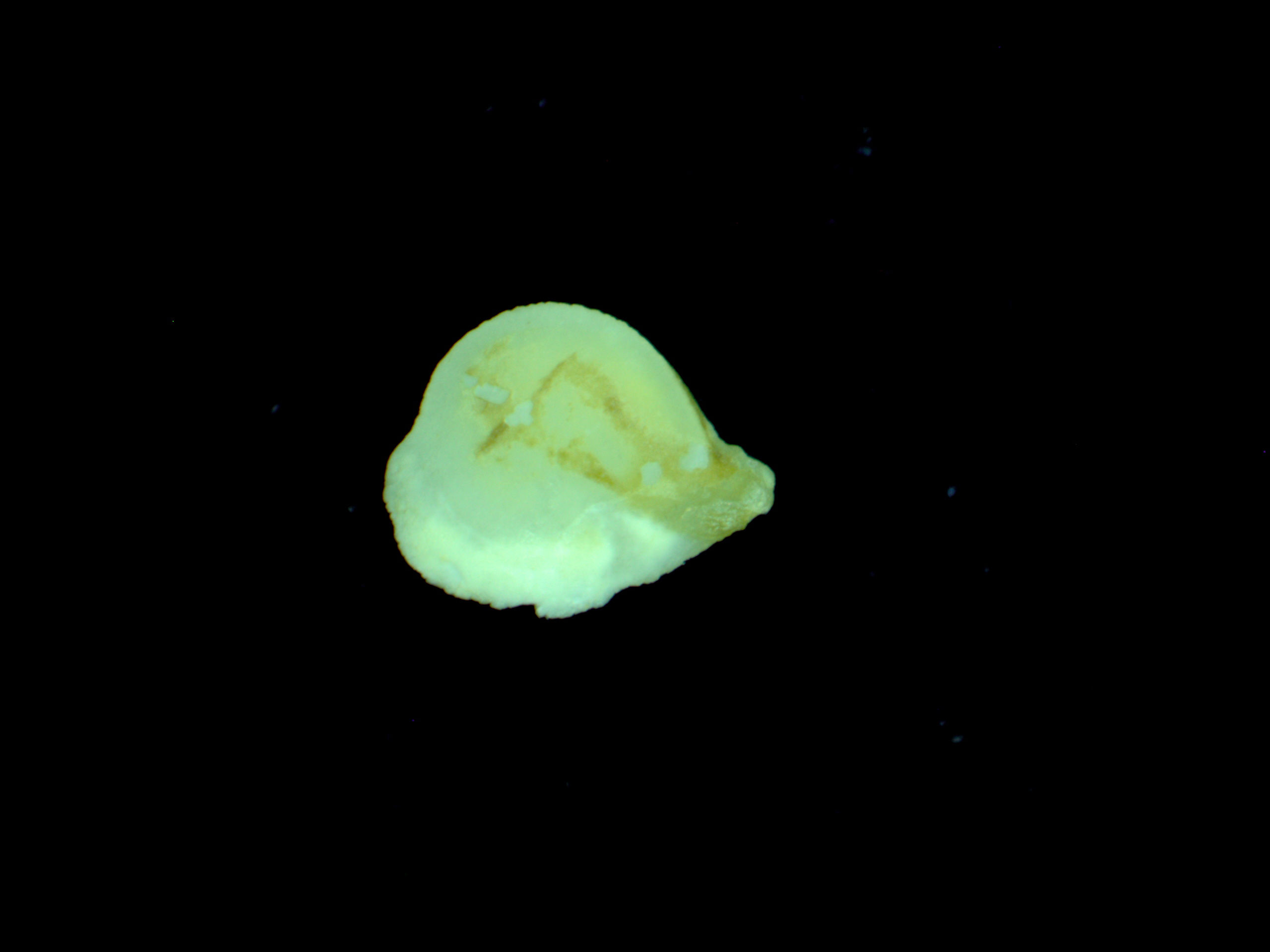

Supplement: Supplemental Information 4 [file peerj-04-1664-s004.zip › HexSag/testing/ARI959_R1.jpg]

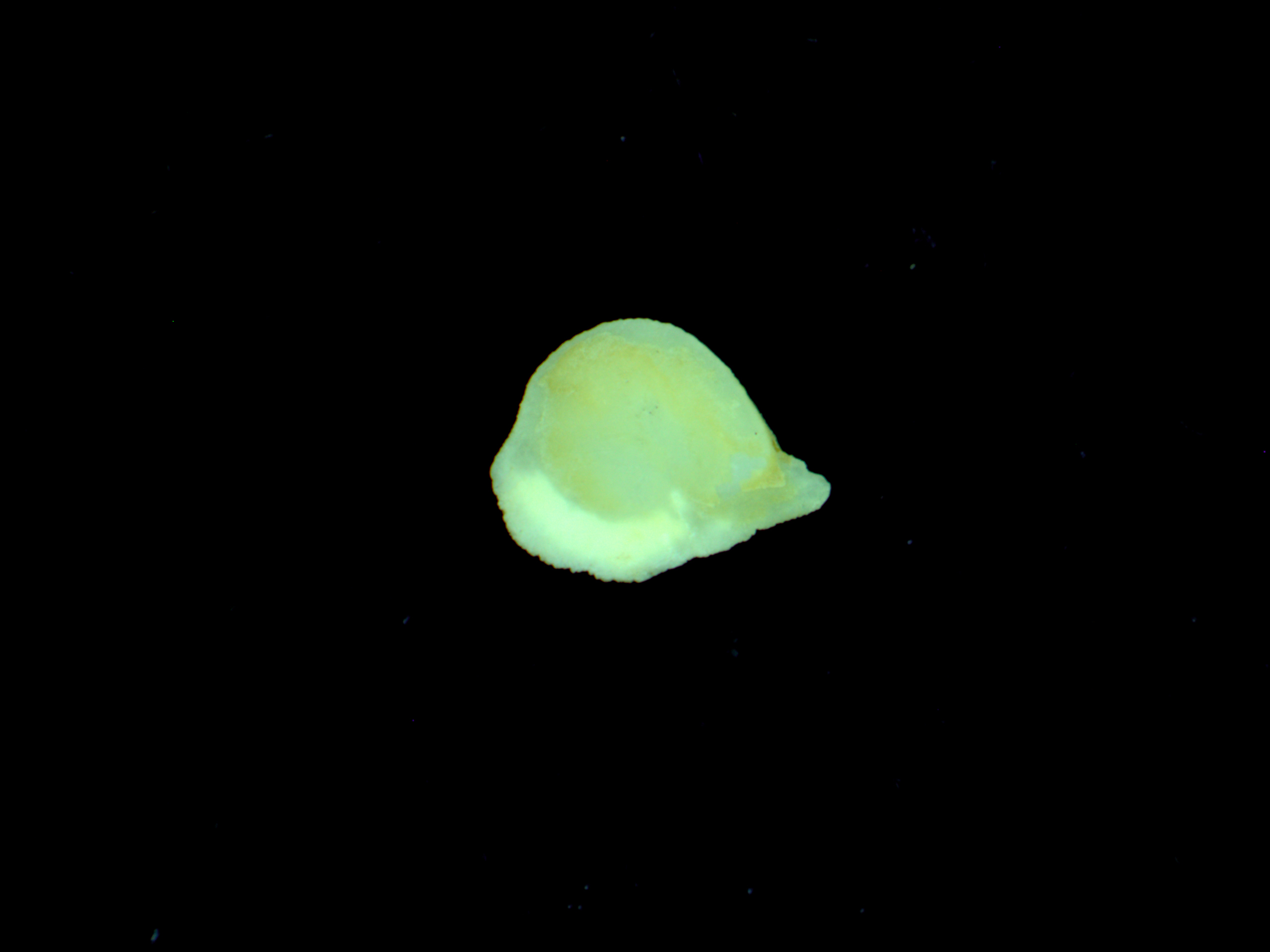

Supplement: Supplemental Information 4 [file peerj-04-1664-s004.zip › HexSag/testing/ARI960_R1.jpg]

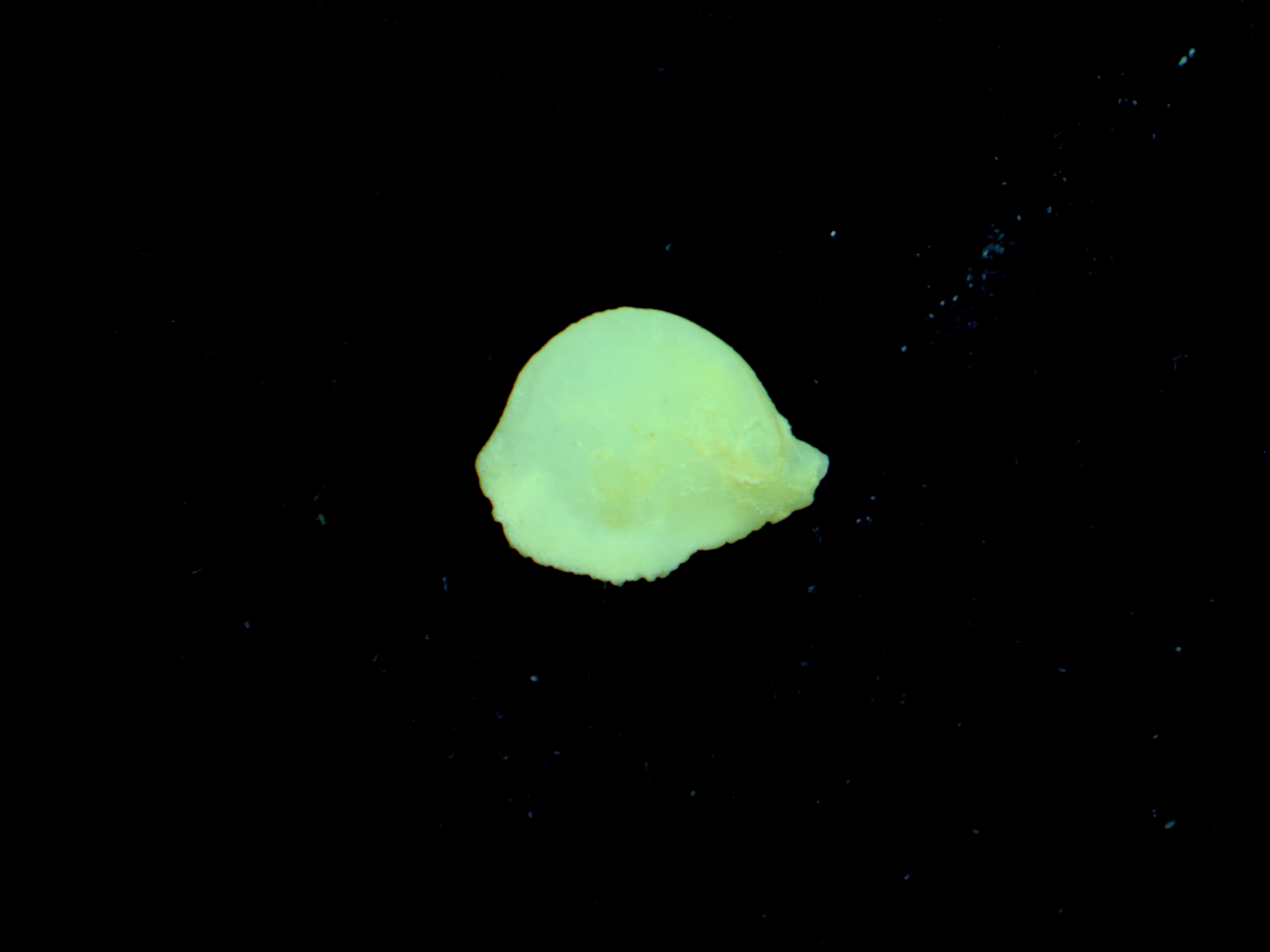

Supplement: Supplemental Information 4 [file peerj-04-1664-s004.zip › HexSag/testing/ARI963_R1.jpg]

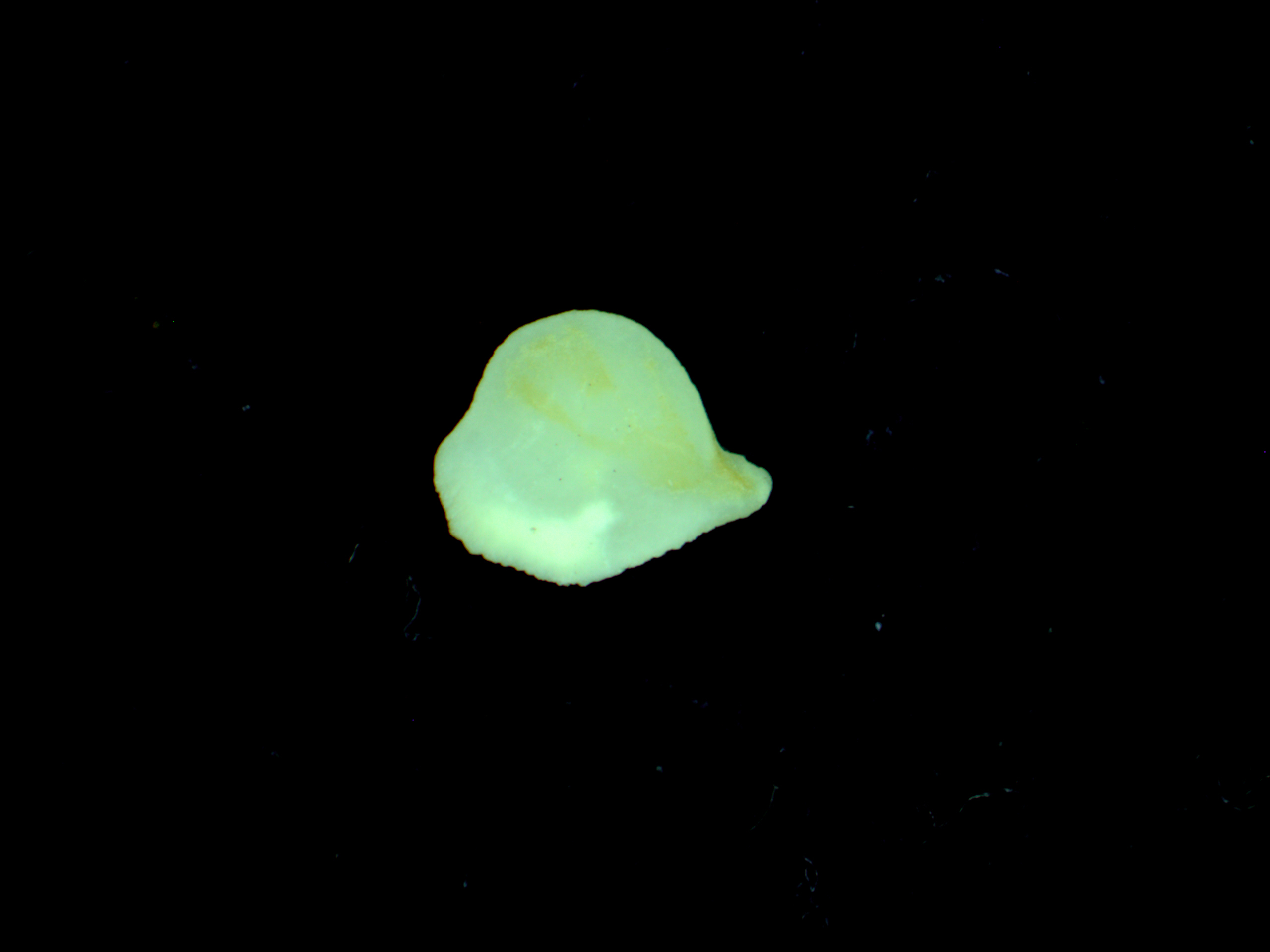

Supplement: Supplemental Information 4 [file peerj-04-1664-s004.zip › HexSag/testing/ARI964_R1.jpg]

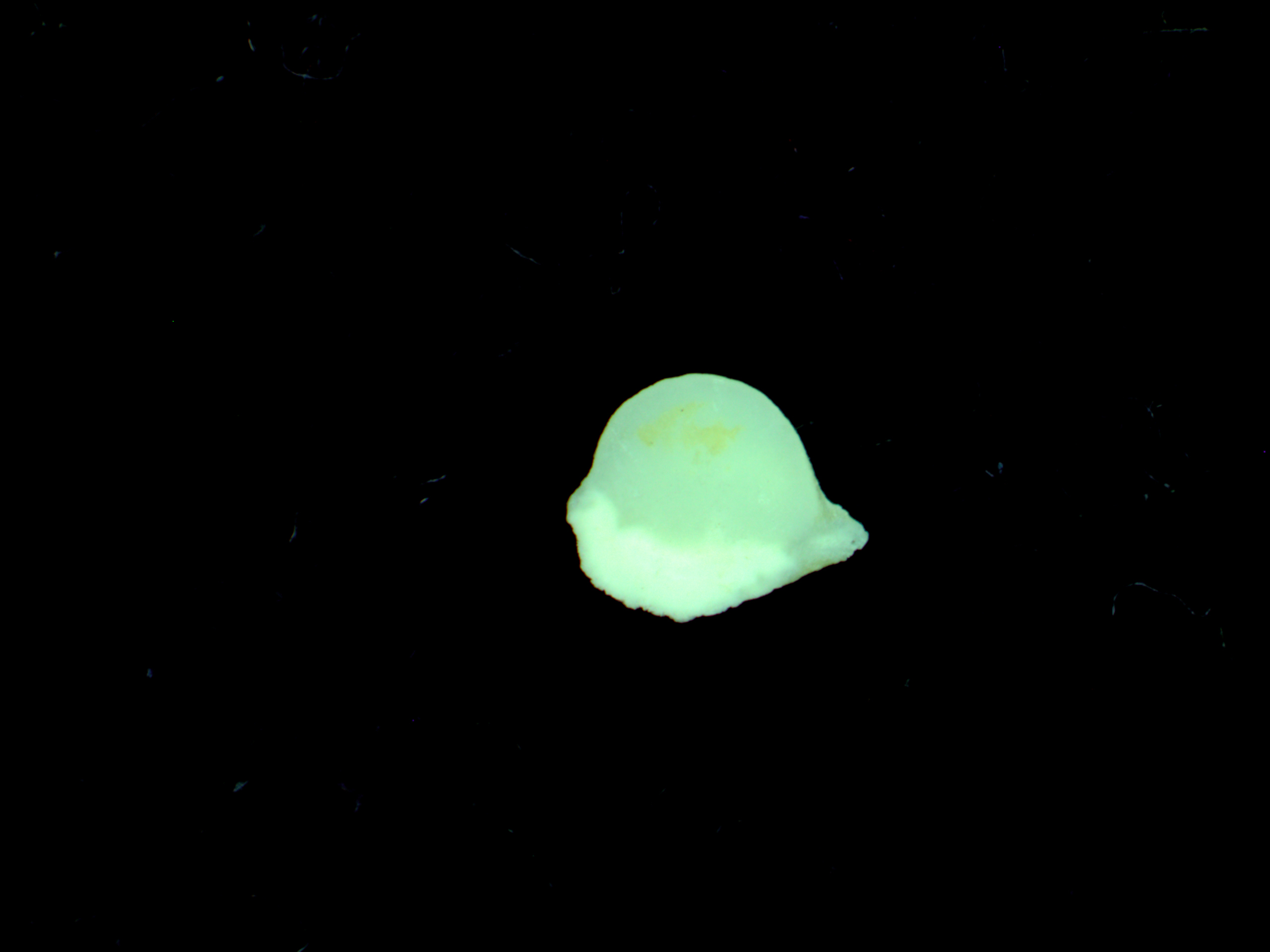

Supplement: Supplemental Information 4 [file peerj-04-1664-s004.zip › HexSag/testing/ARI966_R1.jpg]

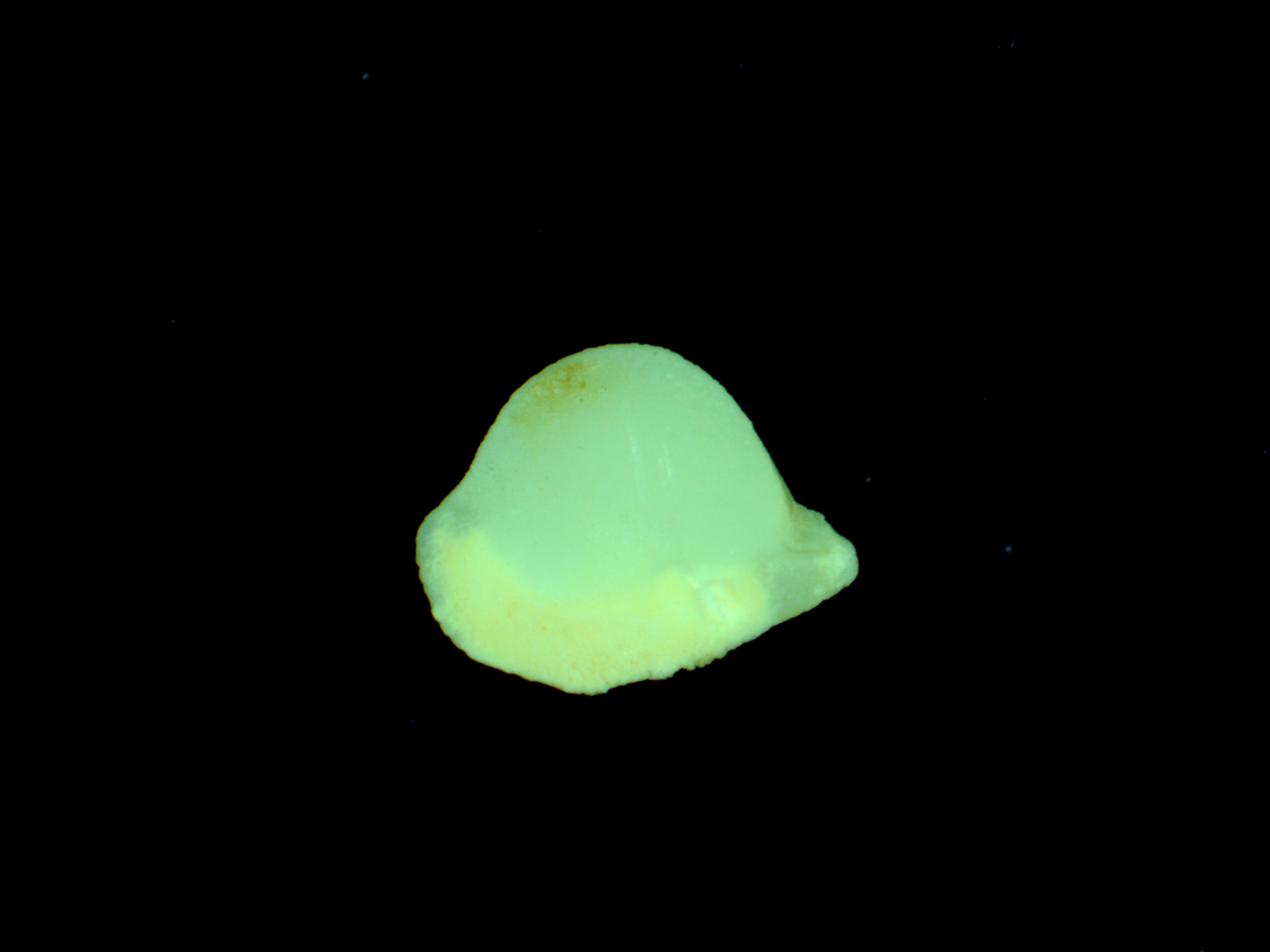

Supplement: Supplemental Information 4 [file peerj-04-1664-s004.zip › HexSag/testing/ARI991_R1.jpg]

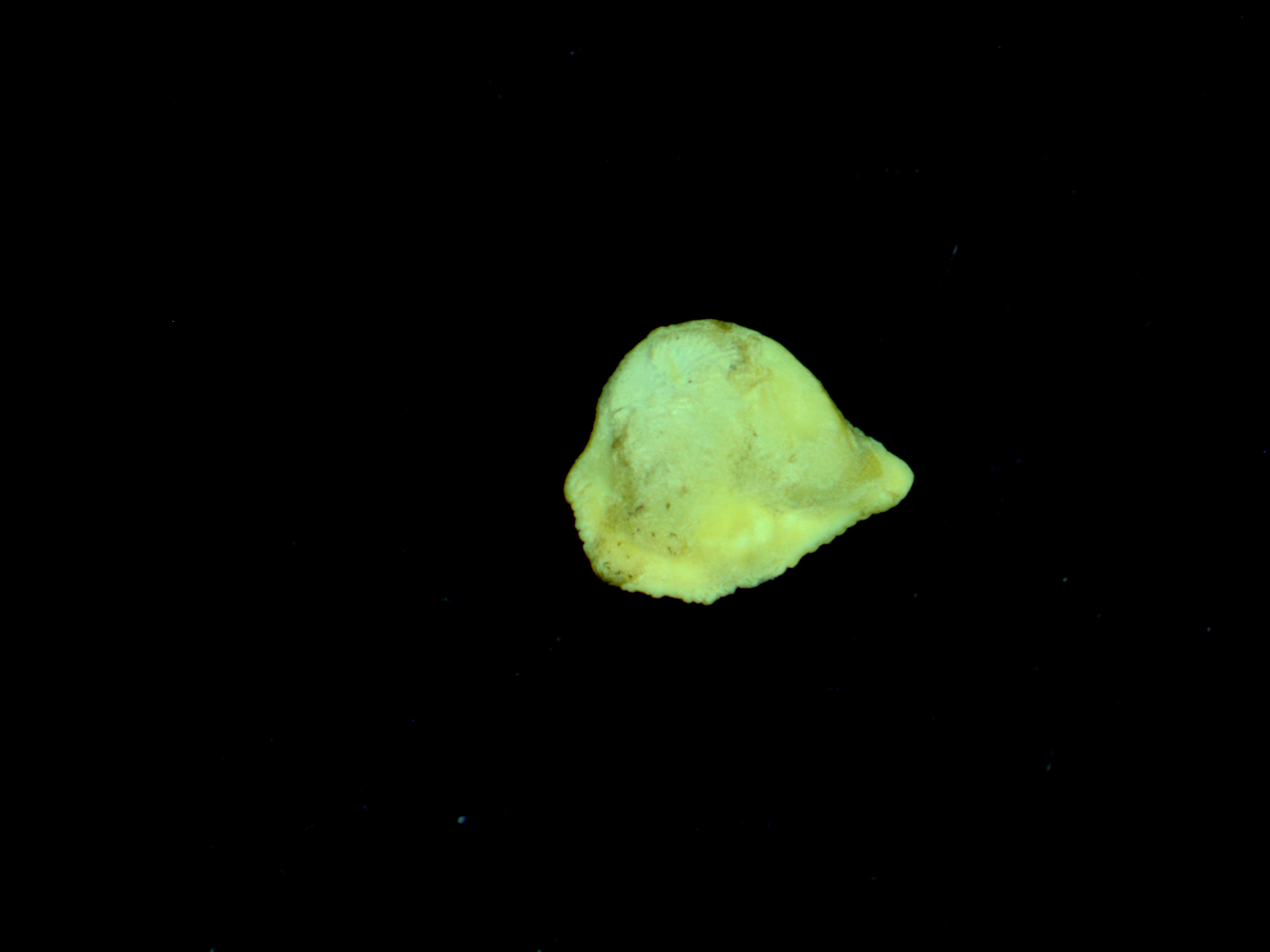

Supplement: Supplemental Information 4 [file peerj-04-1664-s004.zip › HexSag/training/ARI100_R1.jpg]

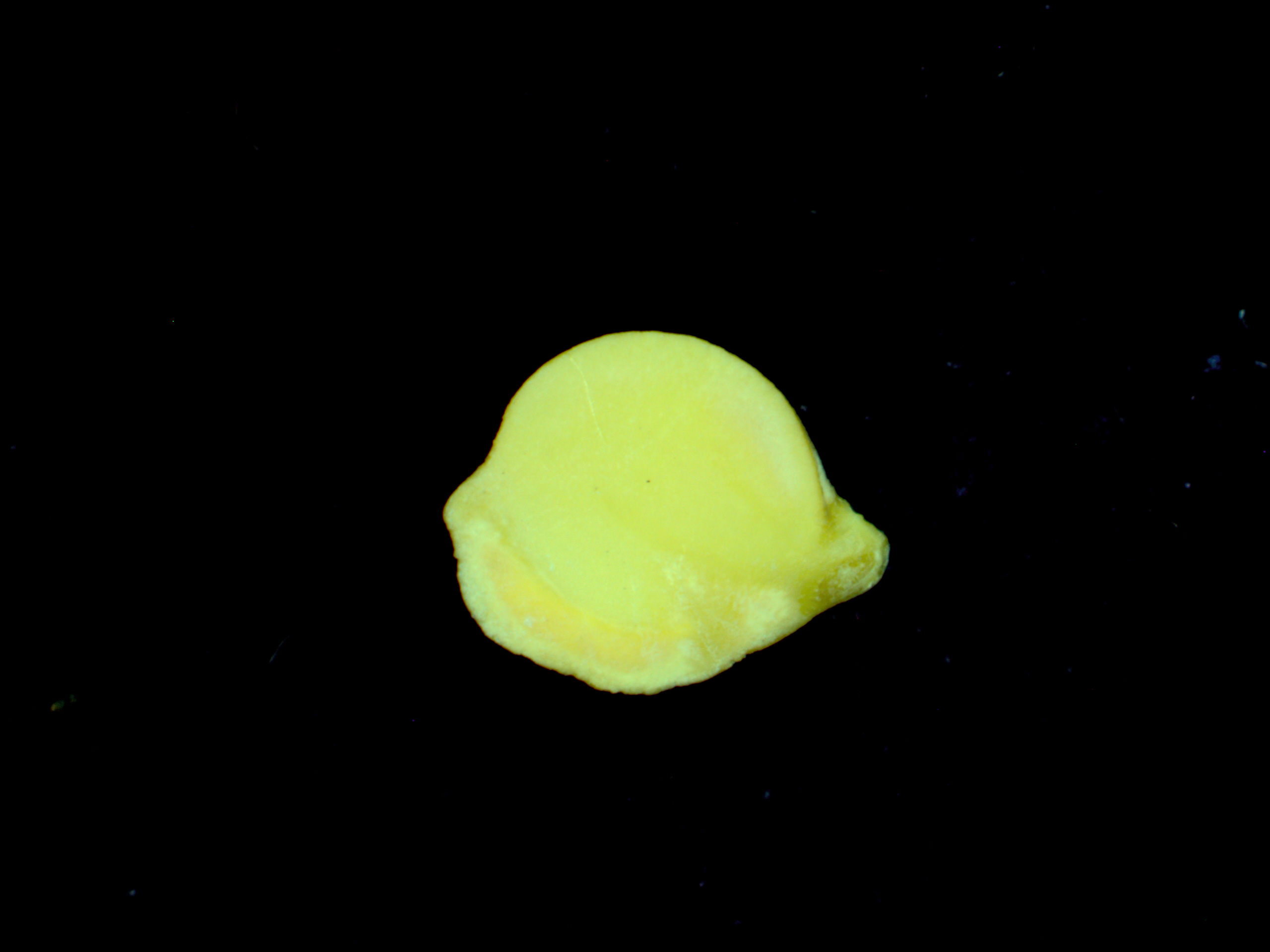

Supplement: Supplemental Information 4 [file peerj-04-1664-s004.zip › HexSag/training/ARI153_R1.jpg]

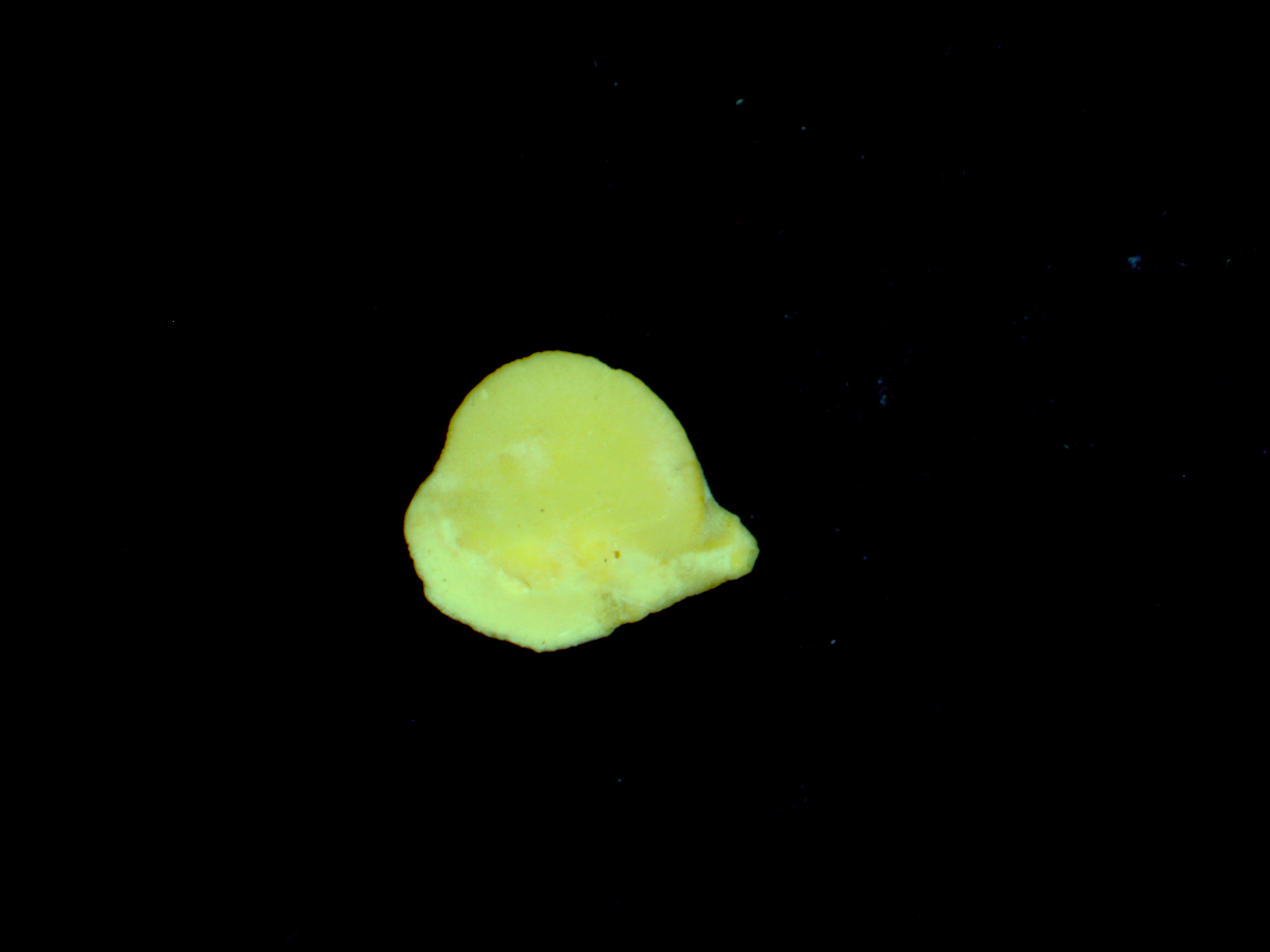

Supplement: Supplemental Information 4 [file peerj-04-1664-s004.zip › HexSag/training/ARI160_R1.jpg]

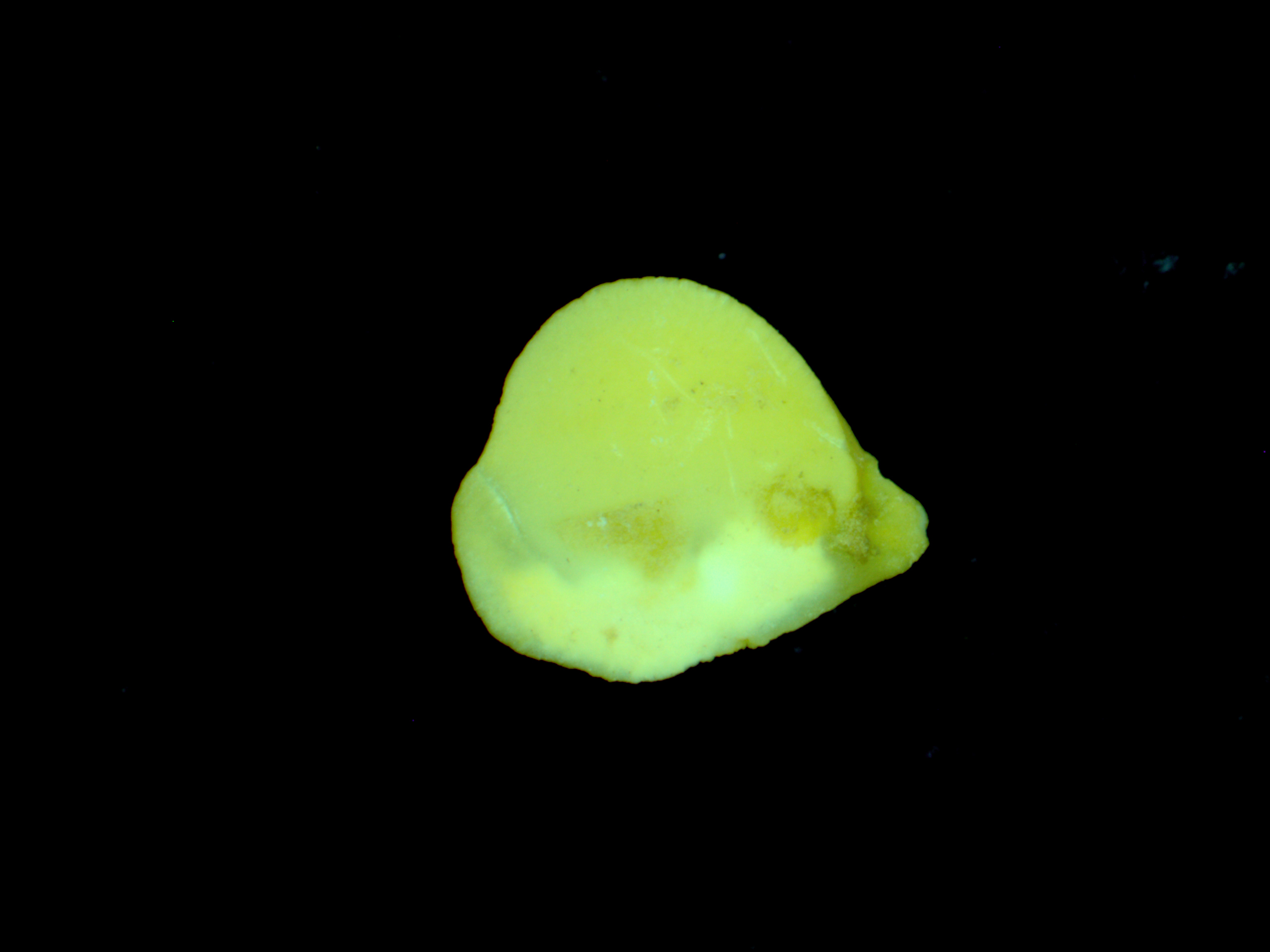

Supplement: Supplemental Information 4 [file peerj-04-1664-s004.zip › HexSag/training/ARI257_R1.jpg]

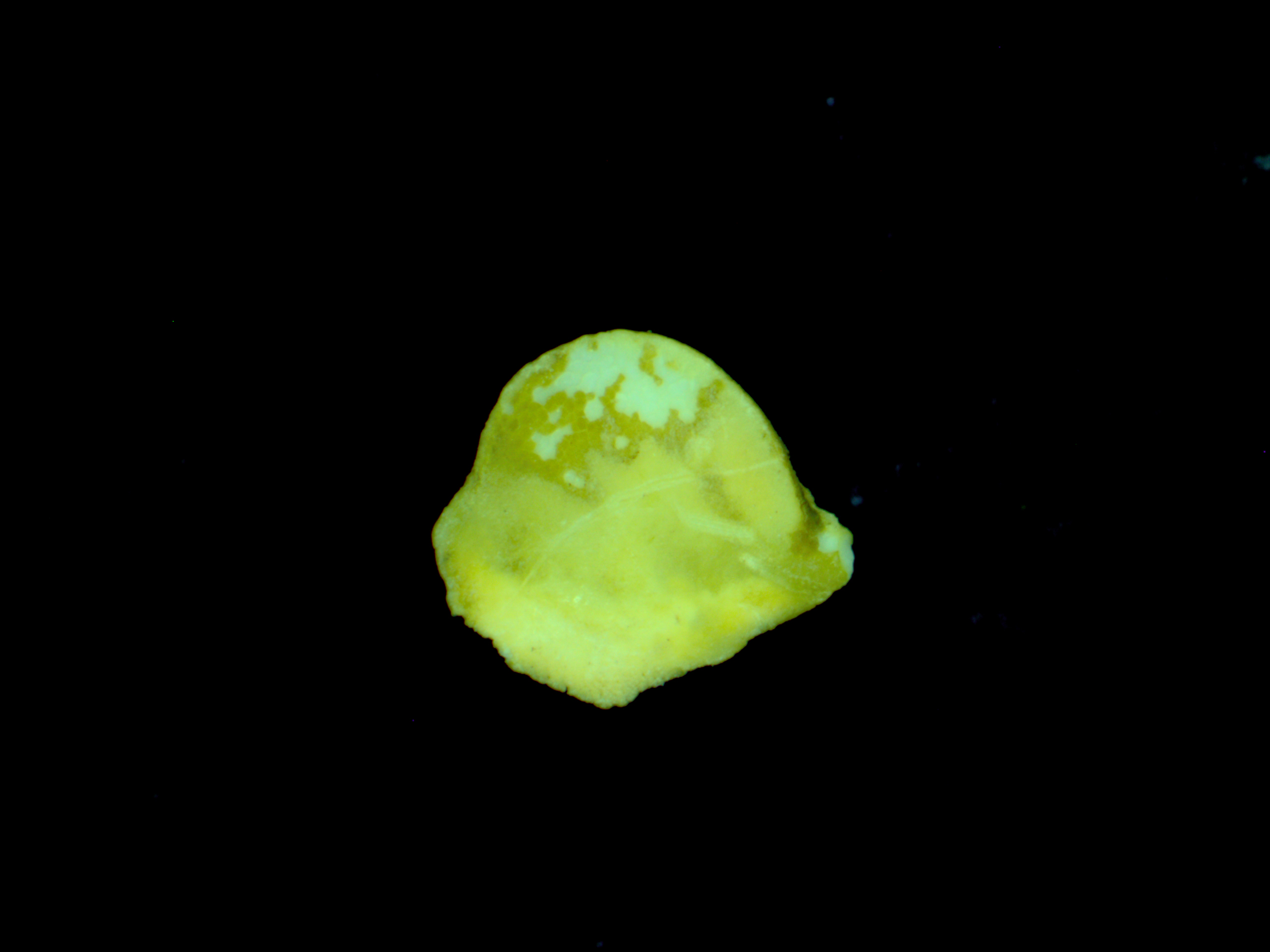

Supplement: Supplemental Information 4 [file peerj-04-1664-s004.zip › HexSag/training/ARI259_R1.jpg]

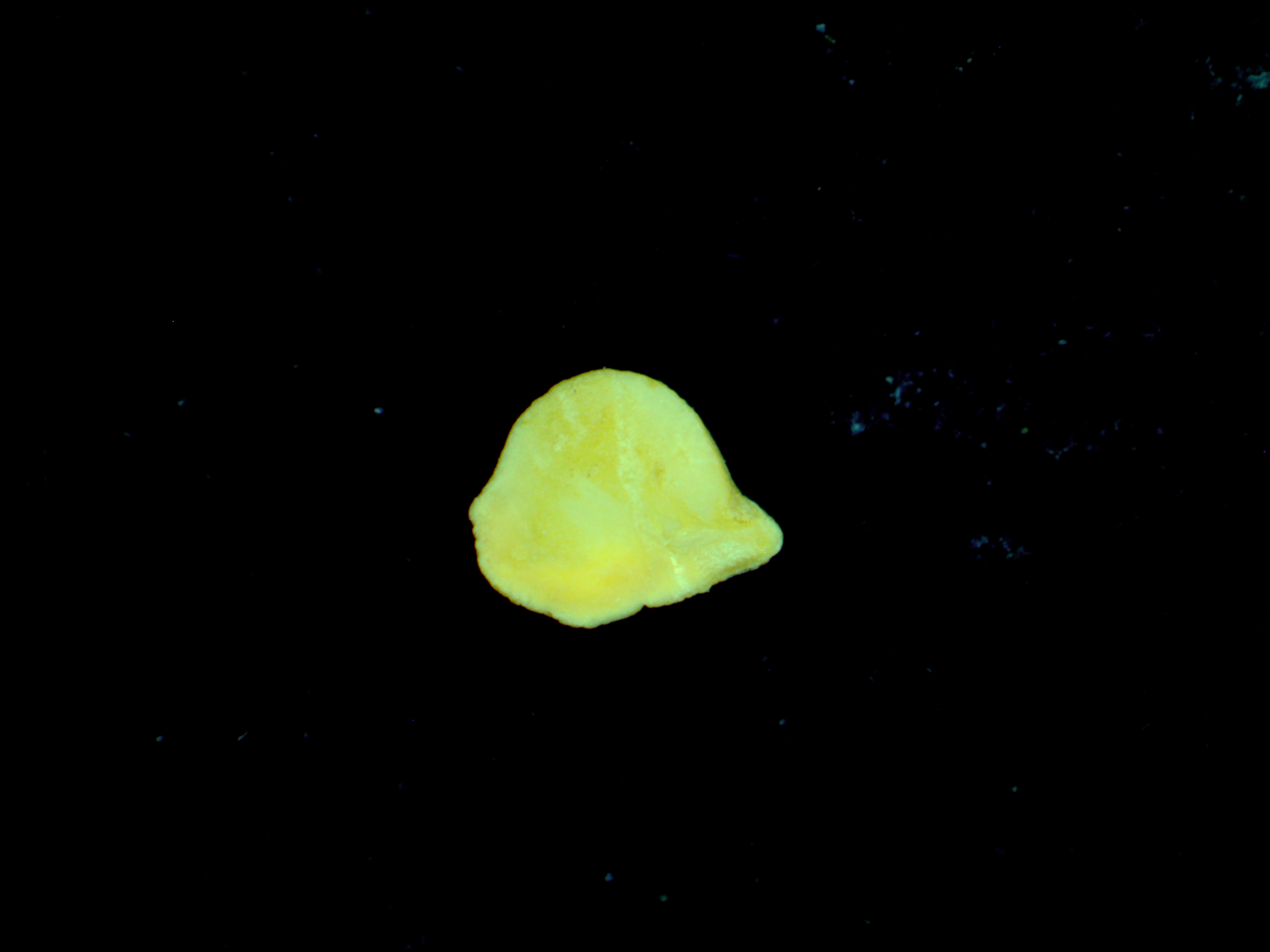

Supplement: Supplemental Information 4 [file peerj-04-1664-s004.zip › HexSag/training/ARI268_R1.jpg]

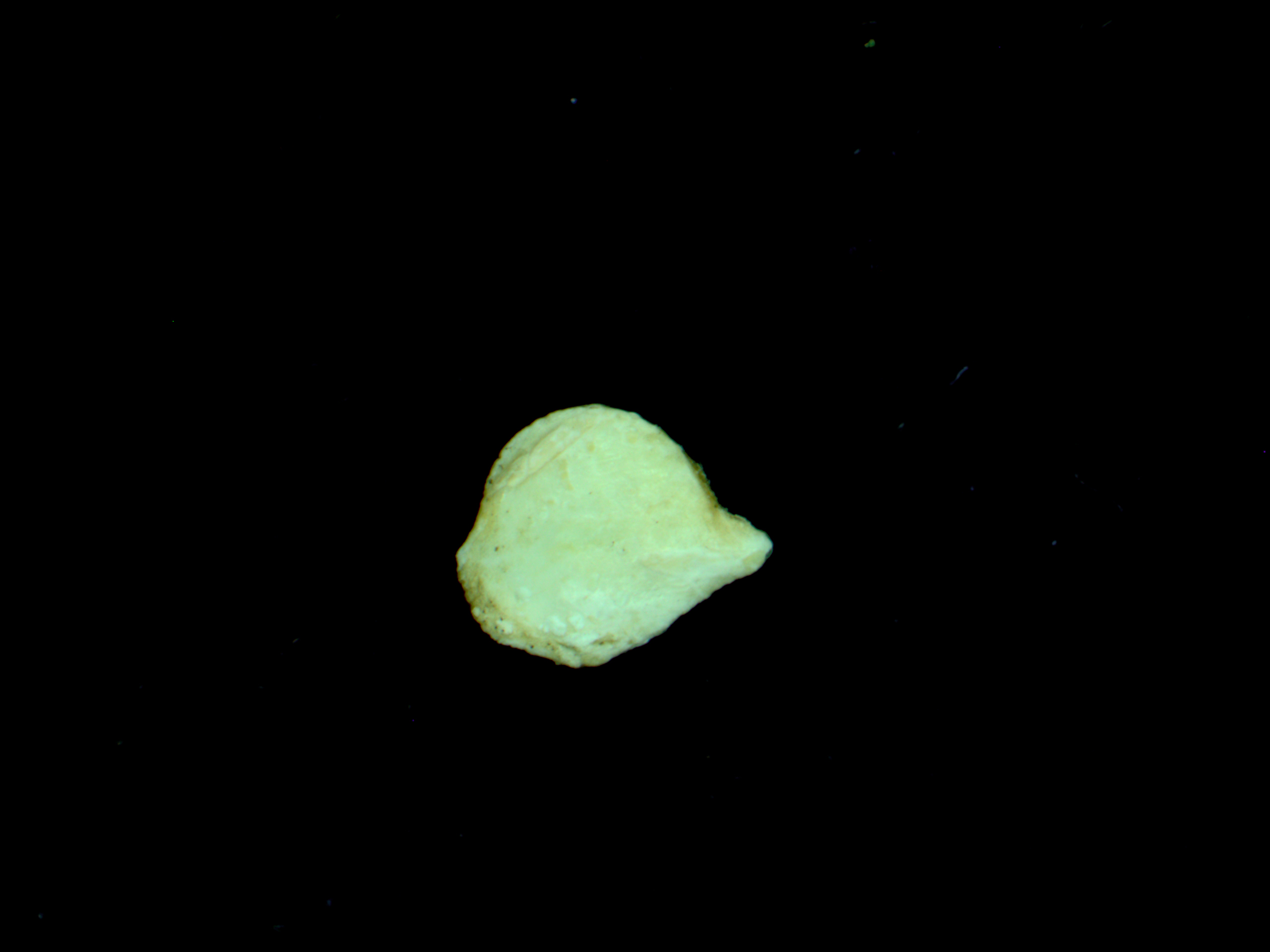

Supplement: Supplemental Information 4 [file peerj-04-1664-s004.zip › HexSag/training/ARI304_R1.jpg]

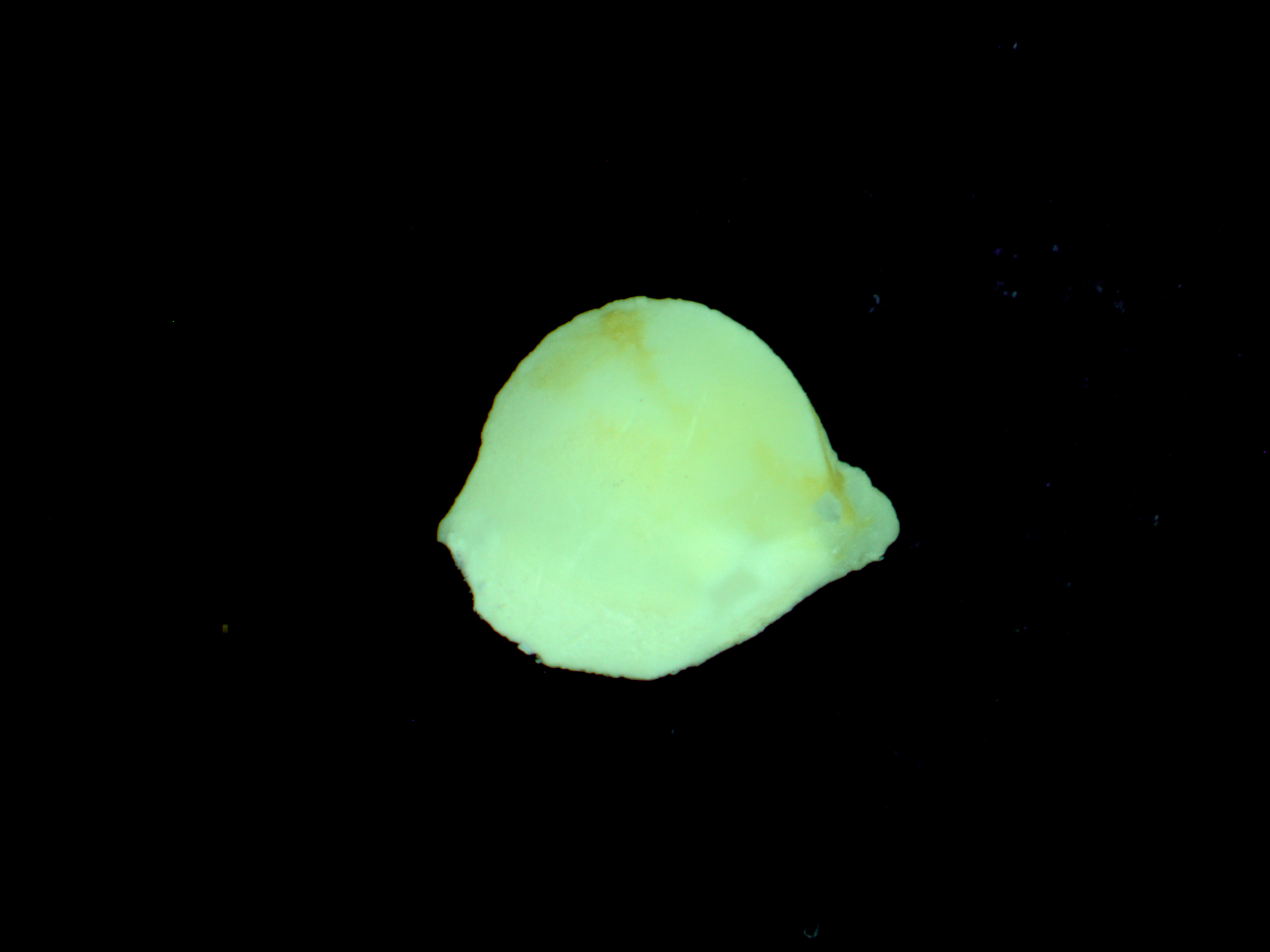

Supplement: Supplemental Information 4 [file peerj-04-1664-s004.zip › HexSag/training/ARI451_R1.jpg]

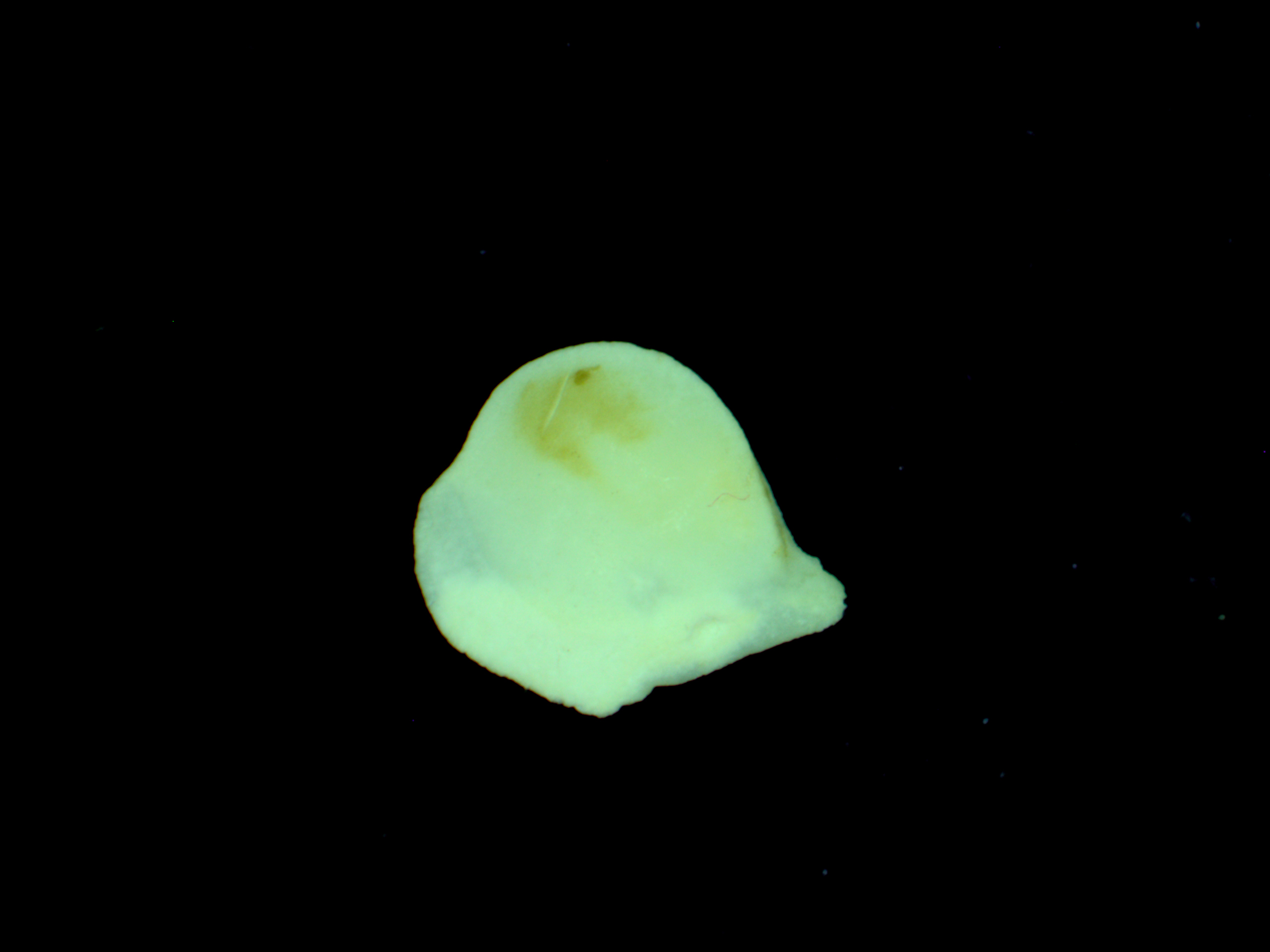

Supplement: Supplemental Information 4 [file peerj-04-1664-s004.zip › HexSag/training/ARI587_R1.jpg]

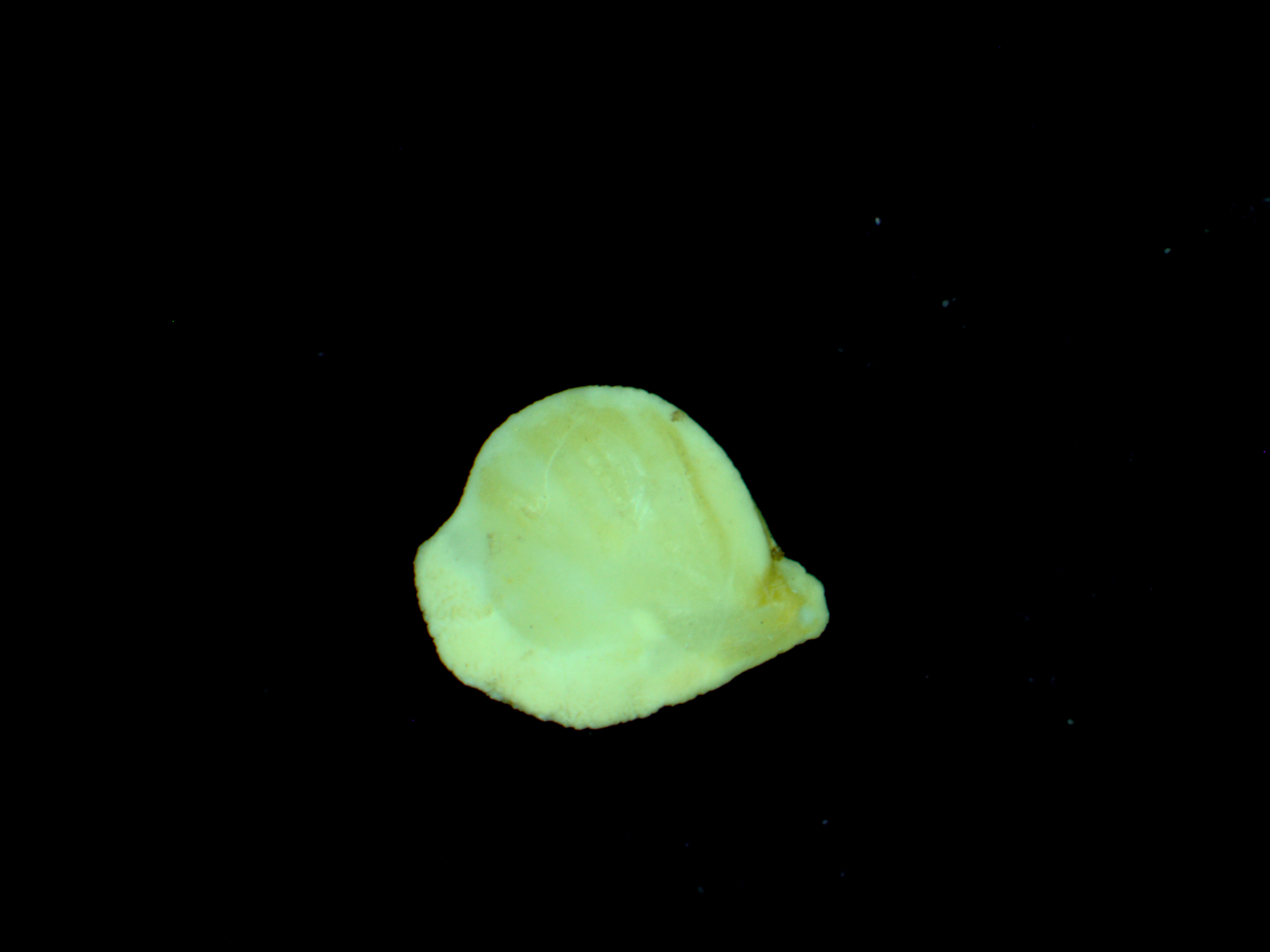

Supplement: Supplemental Information 4 [file peerj-04-1664-s004.zip › HexSag/training/ARI588_R1.jpg]

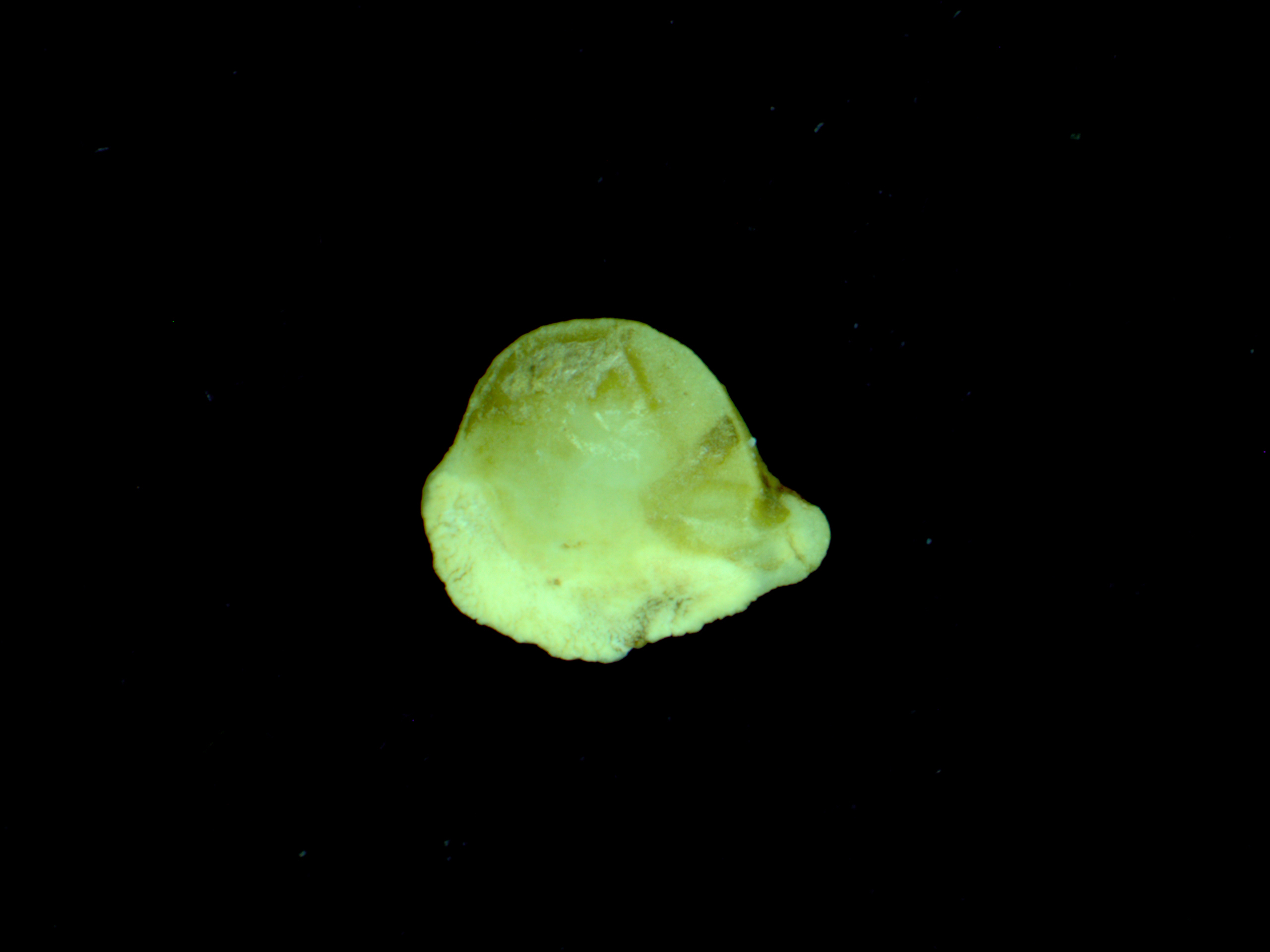

Supplement: Supplemental Information 4 [file peerj-04-1664-s004.zip › HexSag/training/ARI67_R1.jpg]

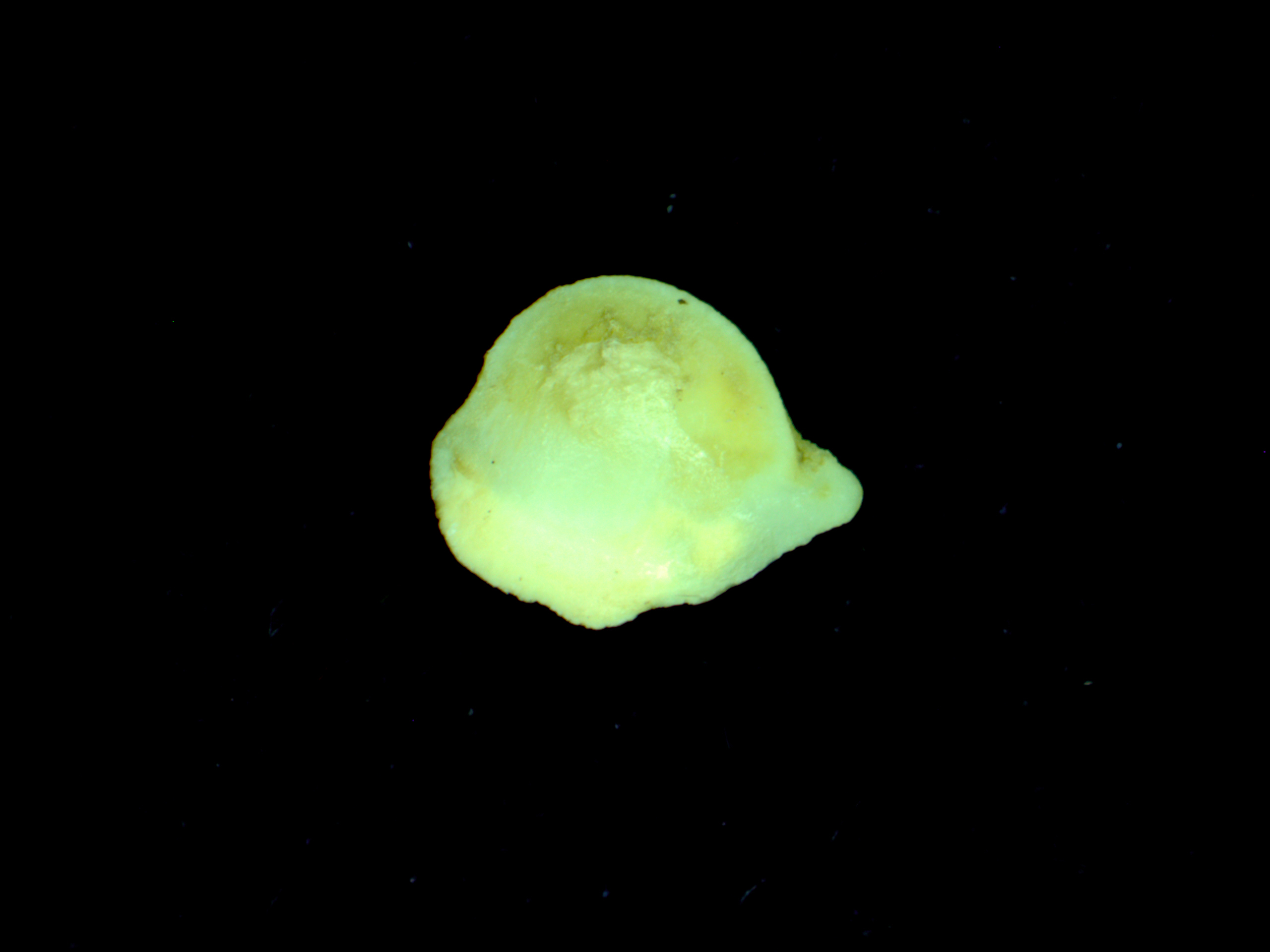

Supplement: Supplemental Information 4 [file peerj-04-1664-s004.zip › HexSag/training/ARI70_R1.jpg]

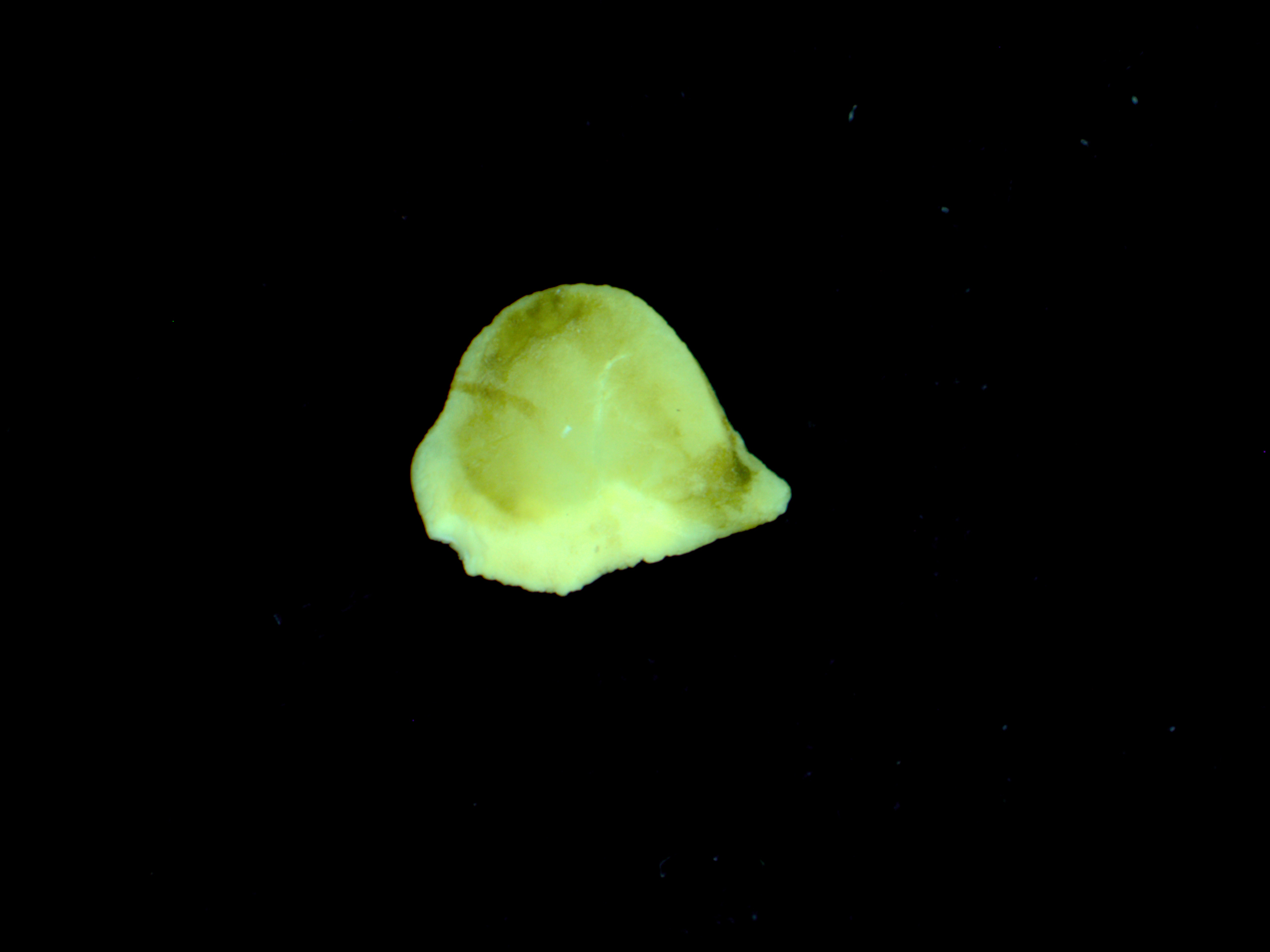

Supplement: Supplemental Information 4 [file peerj-04-1664-s004.zip › HexSag/training/ARI74_R1.jpg]

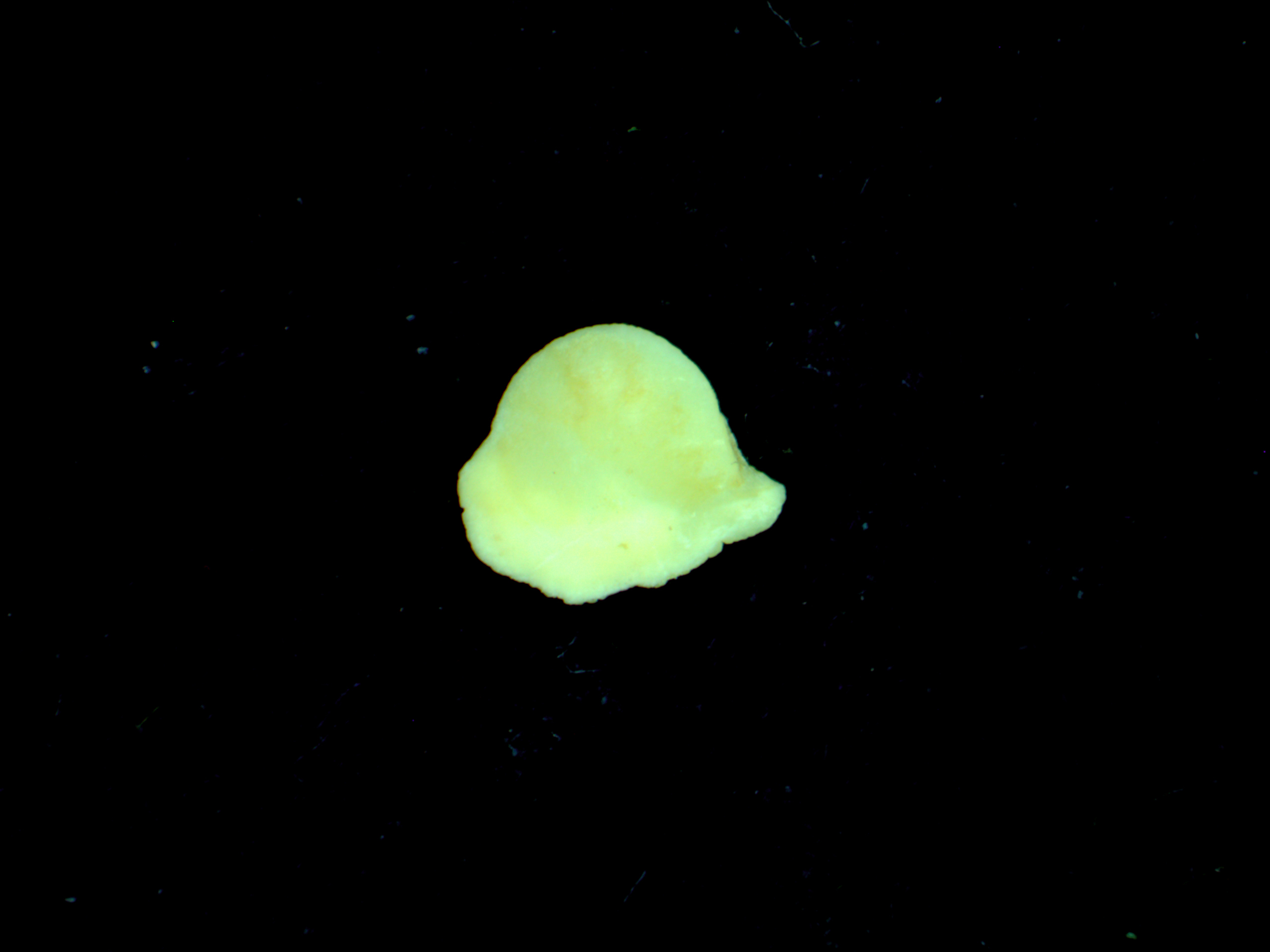

Supplement: Supplemental Information 4 [file peerj-04-1664-s004.zip › HexSag/training/ARI76_R1.jpg]

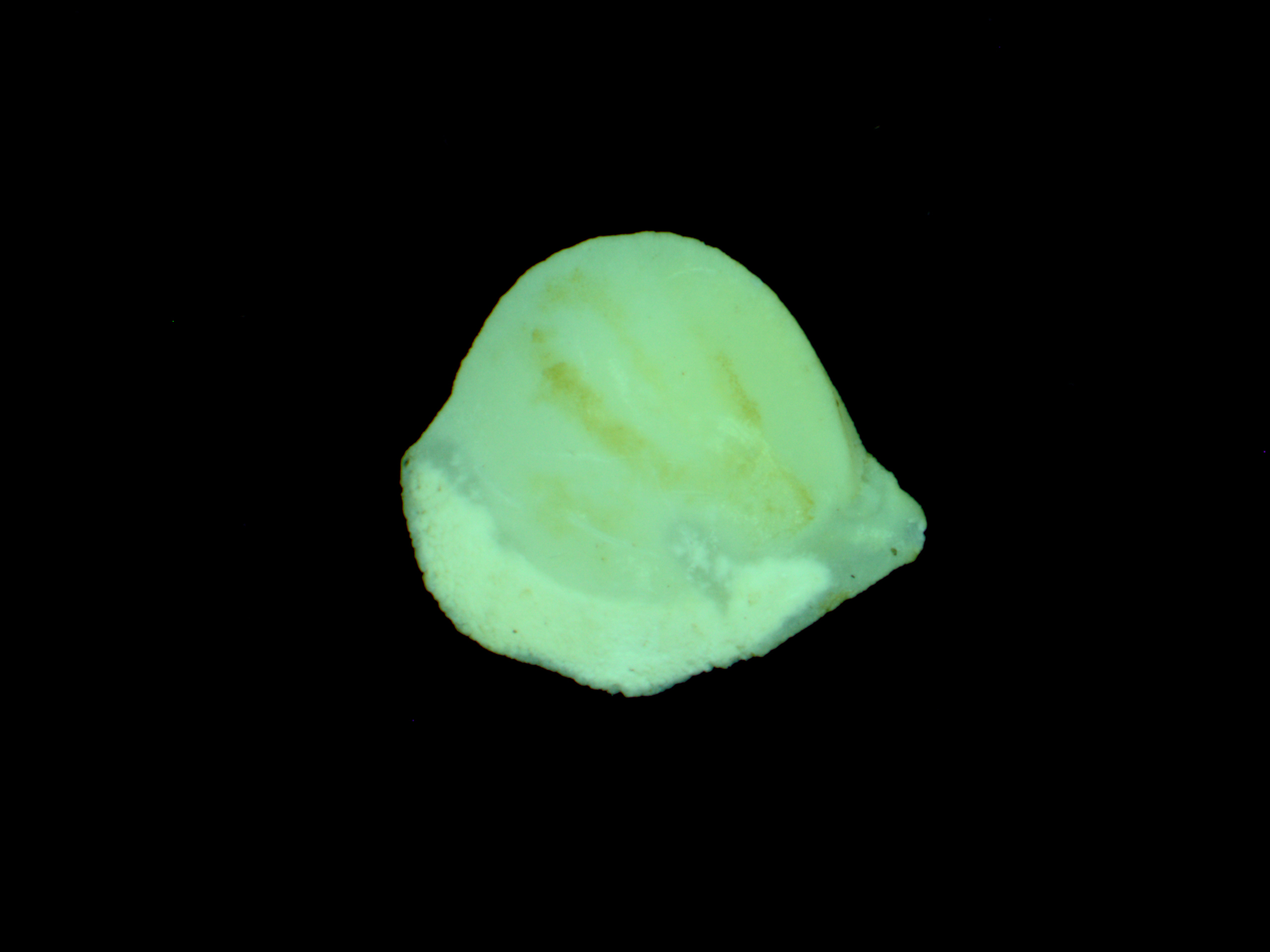

Supplement: Supplemental Information 4 [file peerj-04-1664-s004.zip › HexSag/training/ARI773_R1.jpg]

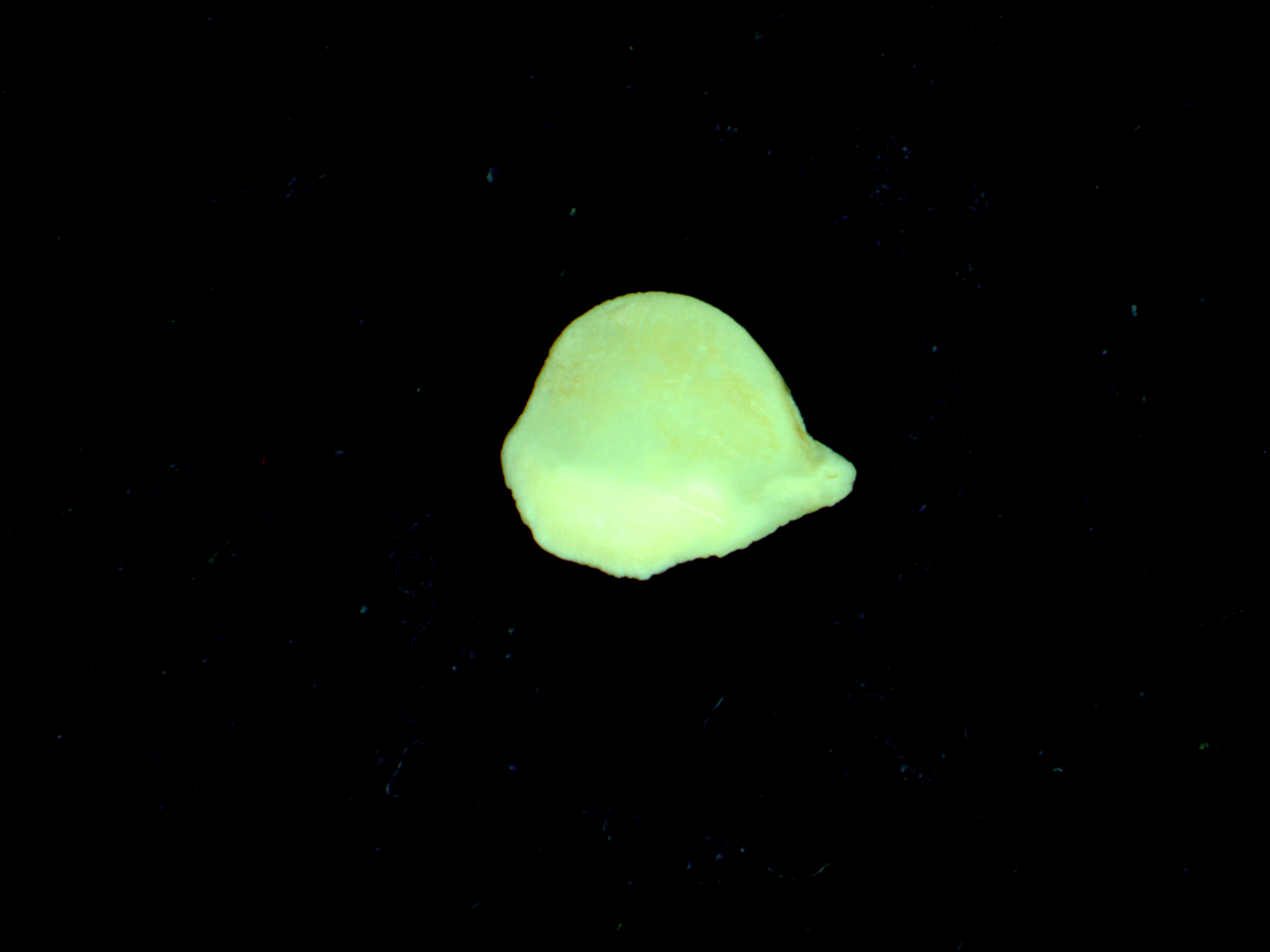

Supplement: Supplemental Information 4 [file peerj-04-1664-s004.zip › HexSag/training/ARI77_R1.jpg]

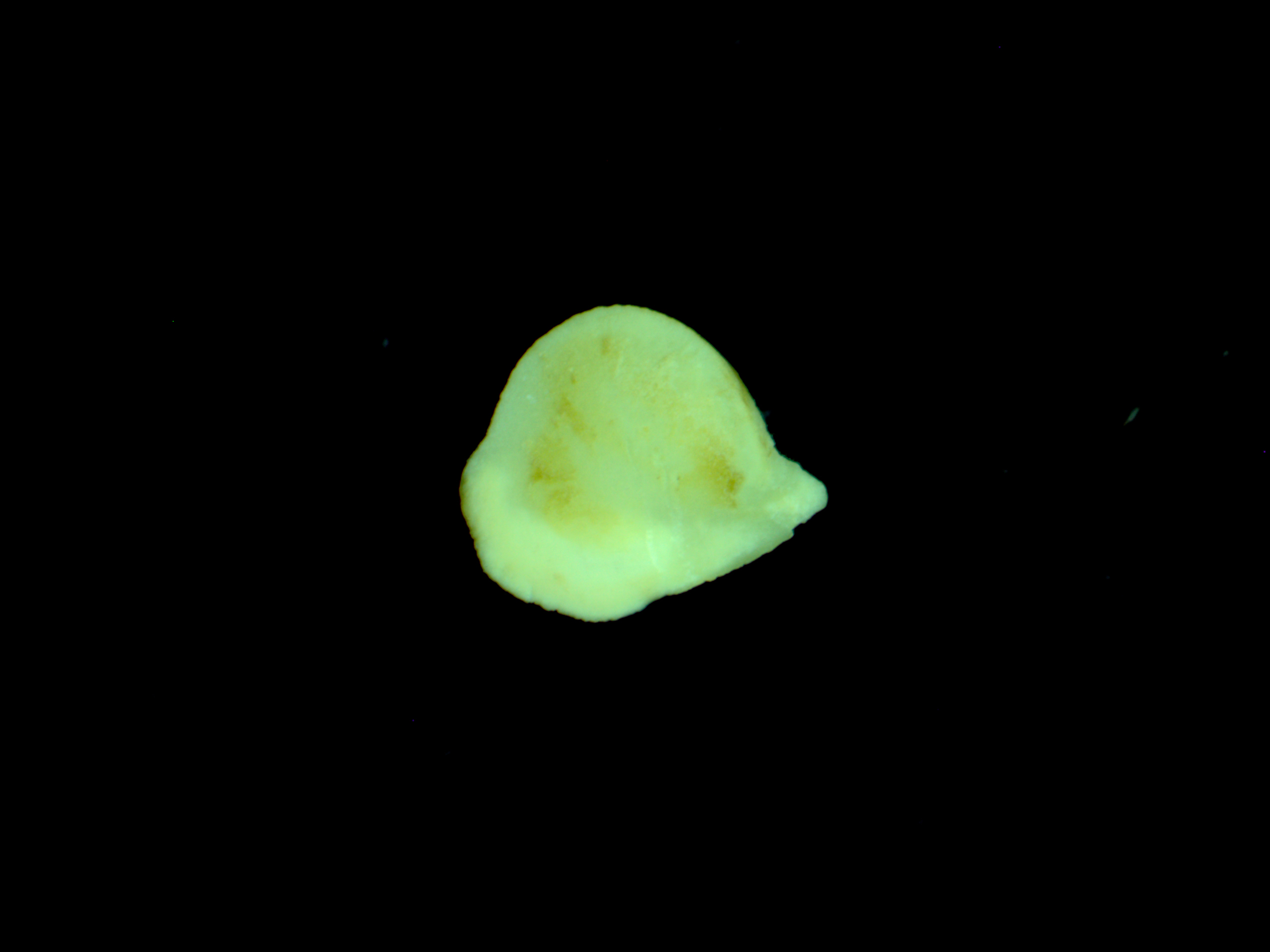

Supplement: Supplemental Information 4 [file peerj-04-1664-s004.zip › HexSag/training/ARI95_R1.jpg]

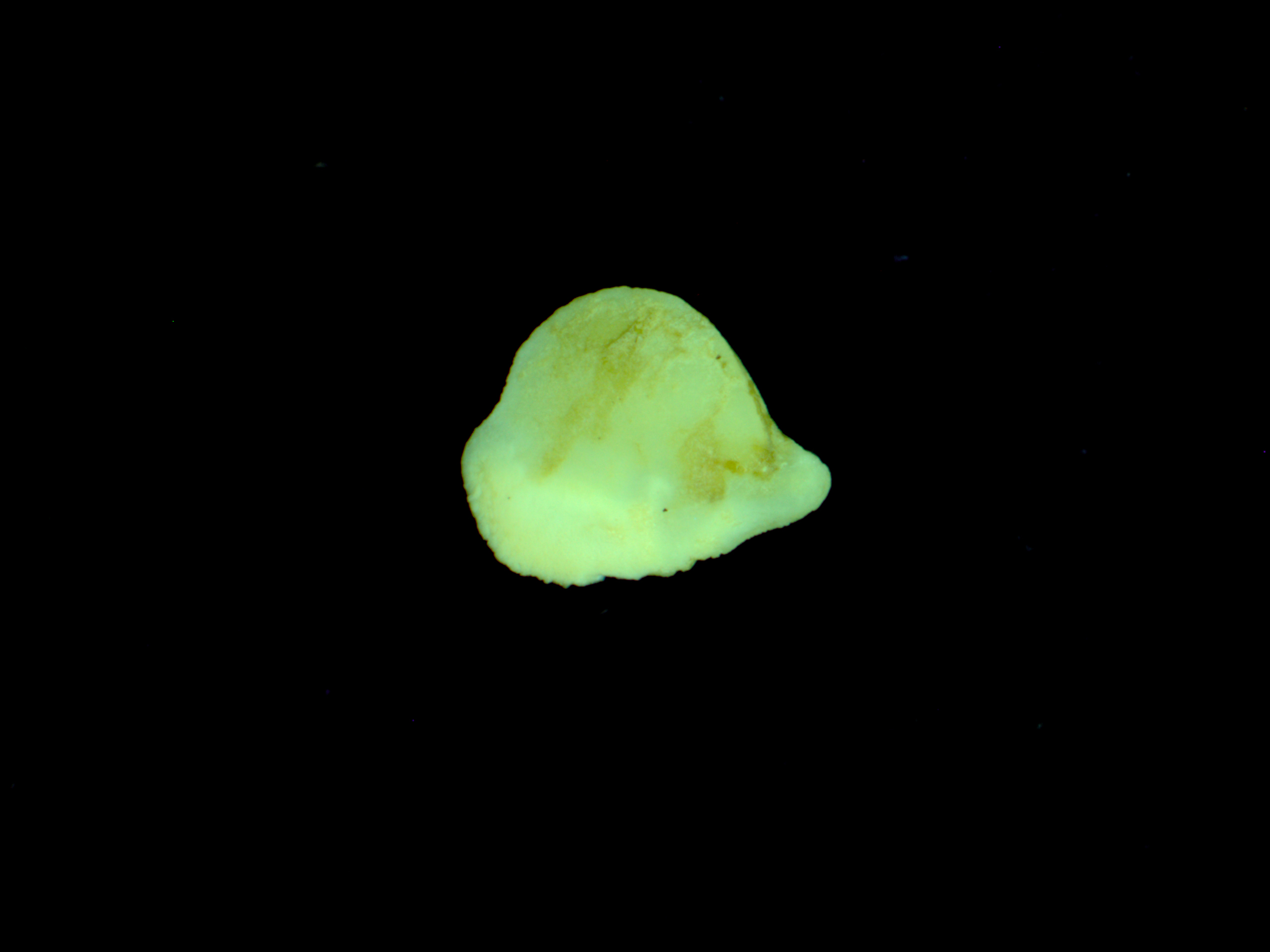

Supplement: Supplemental Information 4 [file peerj-04-1664-s004.zip › HexSag/training/ARI96_R1.jpg]

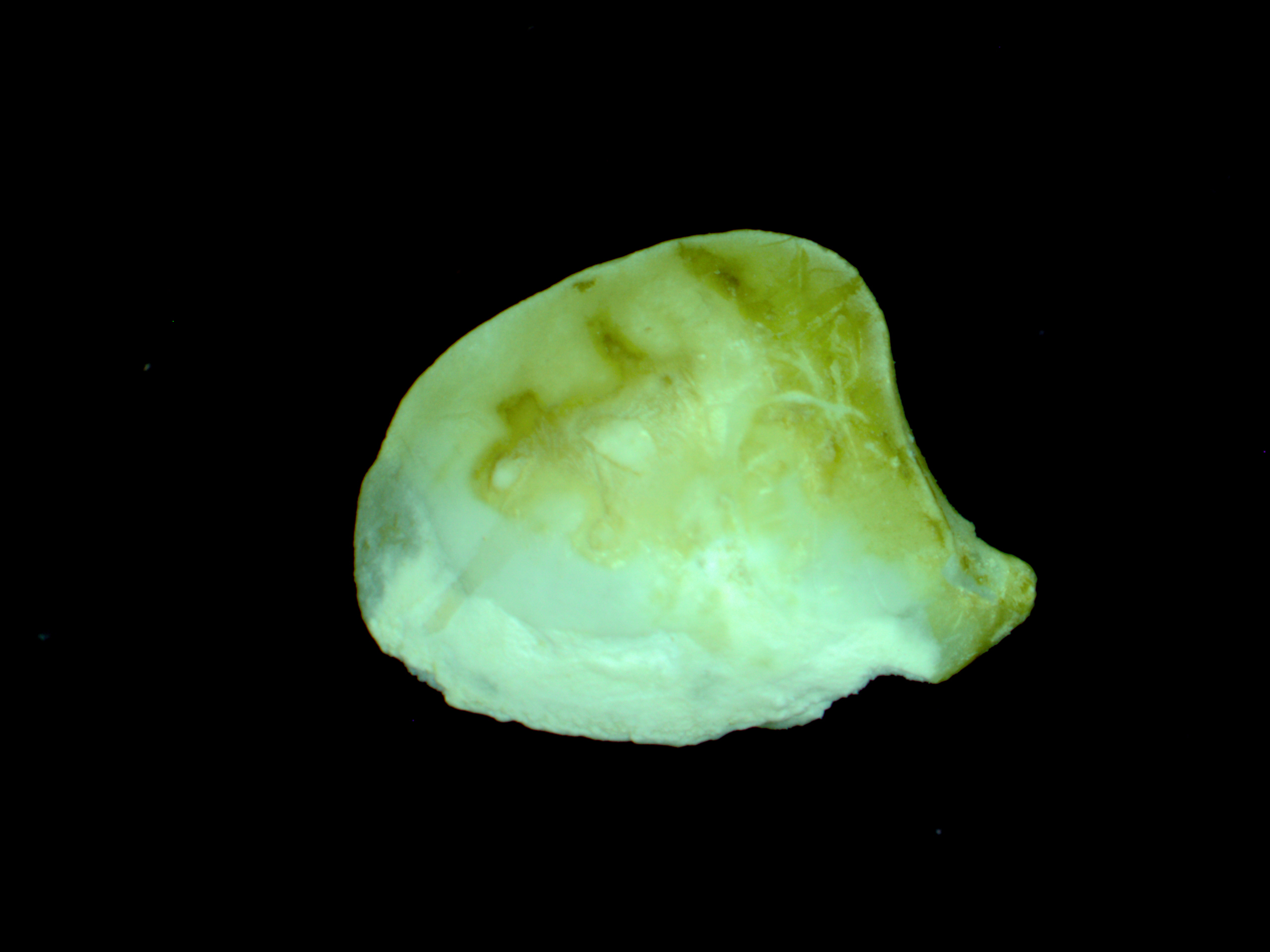

Supplement: Supplemental Information 5 [file peerj-04-1664-s005.zip › Nemcae/testing/ARI1012_R1.jpg]

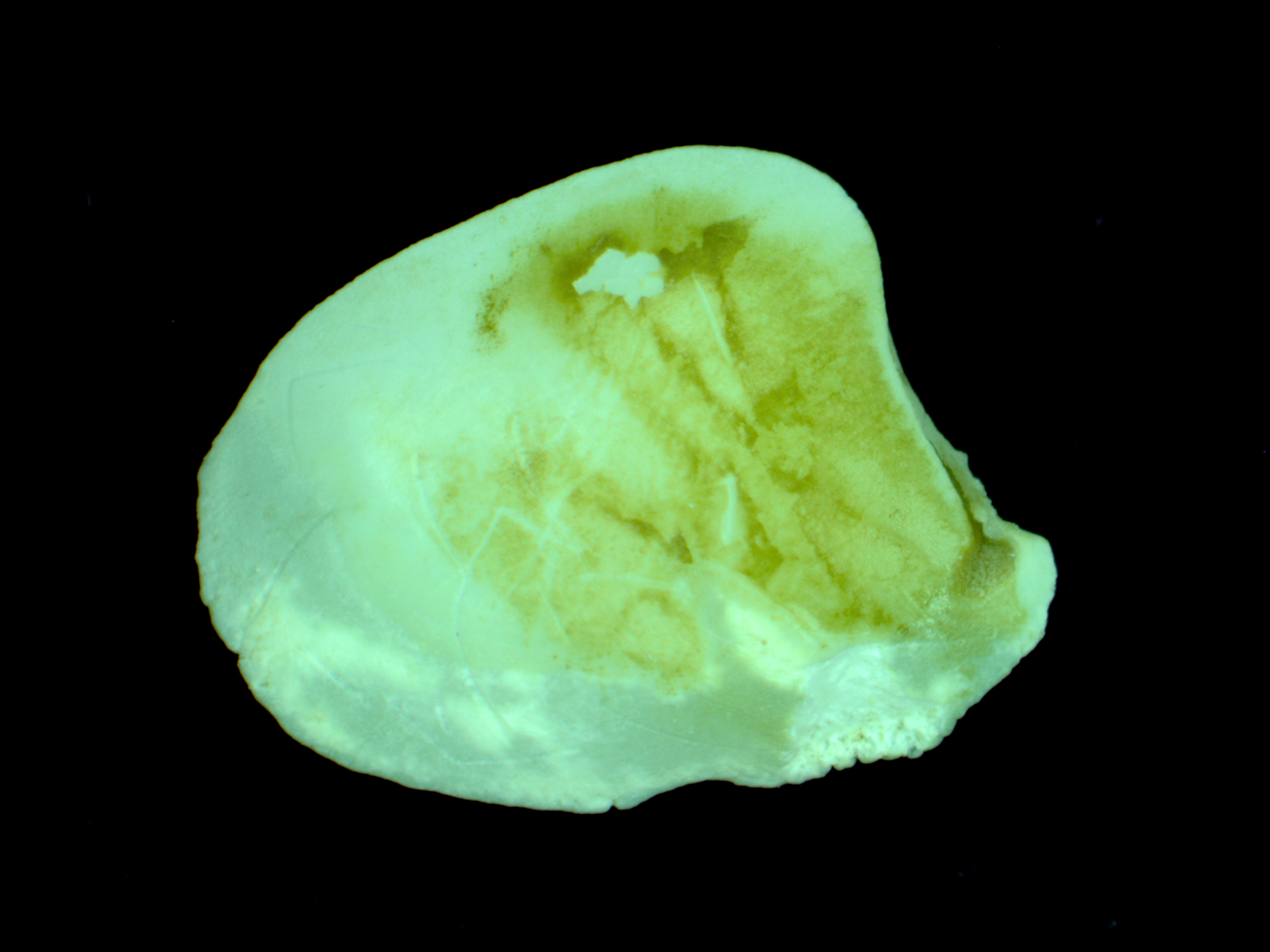

Supplement: Supplemental Information 5 [file peerj-04-1664-s005.zip › Nemcae/testing/ARI928_R1.jpg]

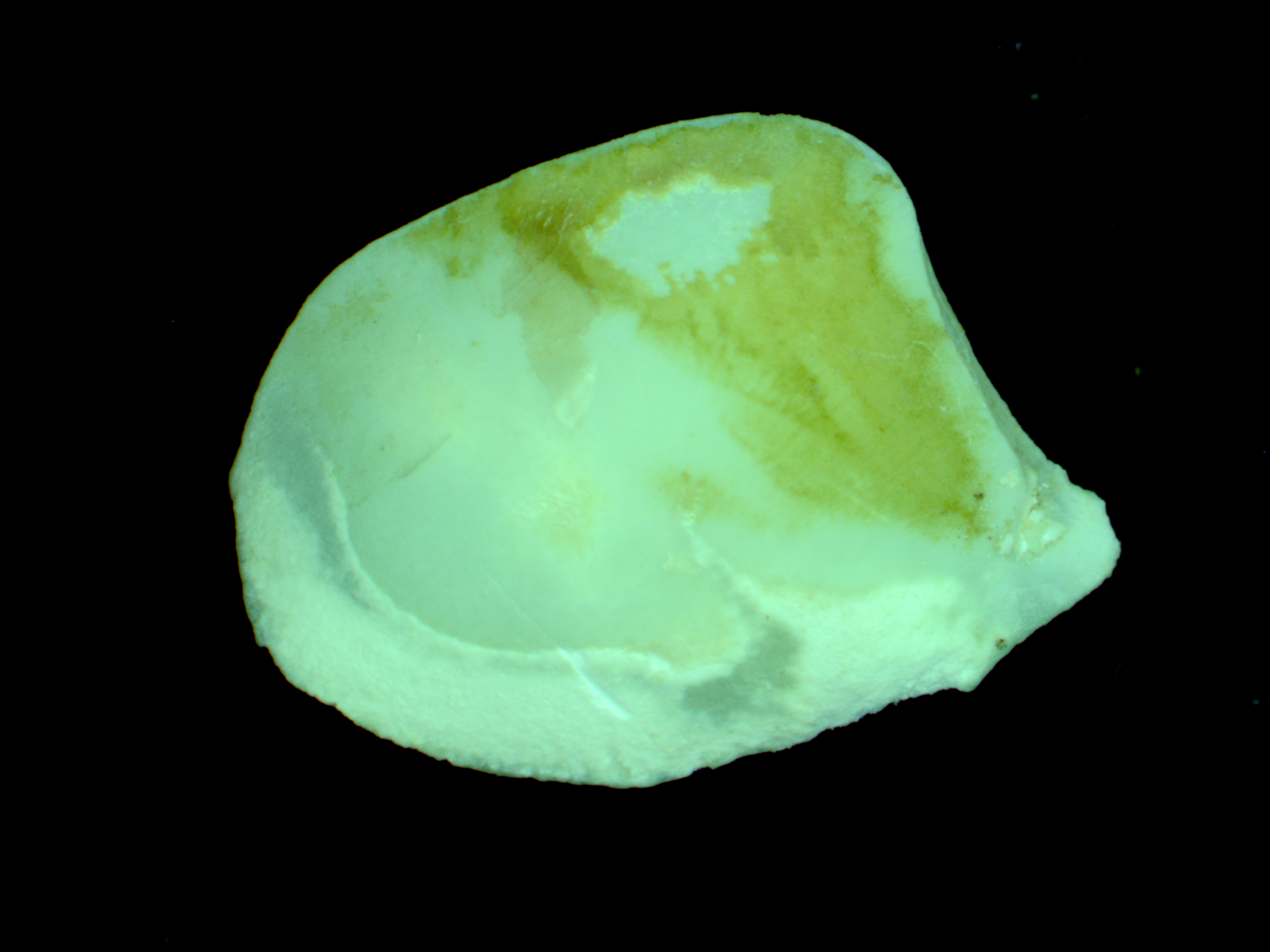

Supplement: Supplemental Information 5 [file peerj-04-1664-s005.zip › Nemcae/testing/ARI929_R1.jpg]

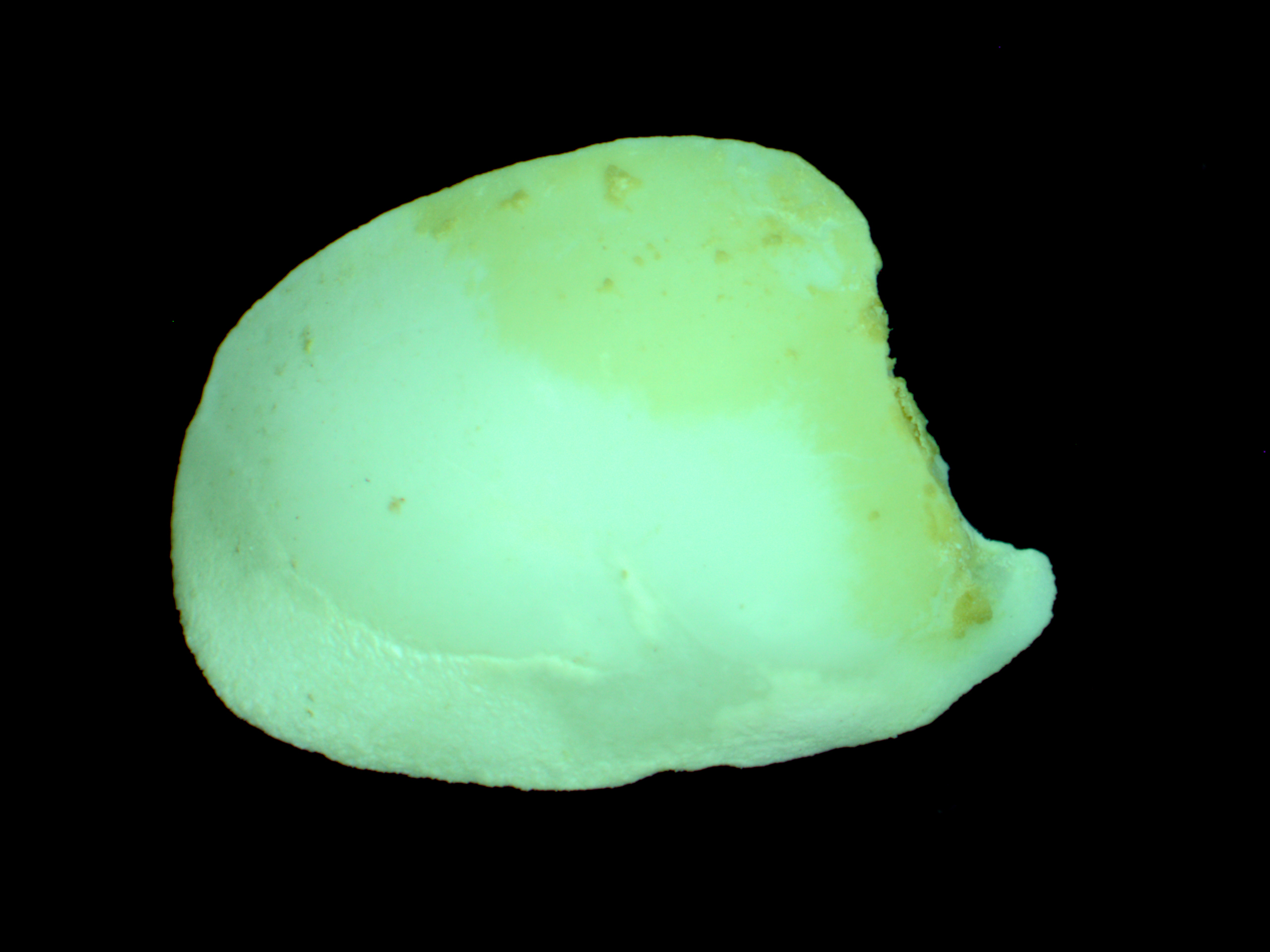

Supplement: Supplemental Information 5 [file peerj-04-1664-s005.zip › Nemcae/testing/ARI930_R1.jpg]

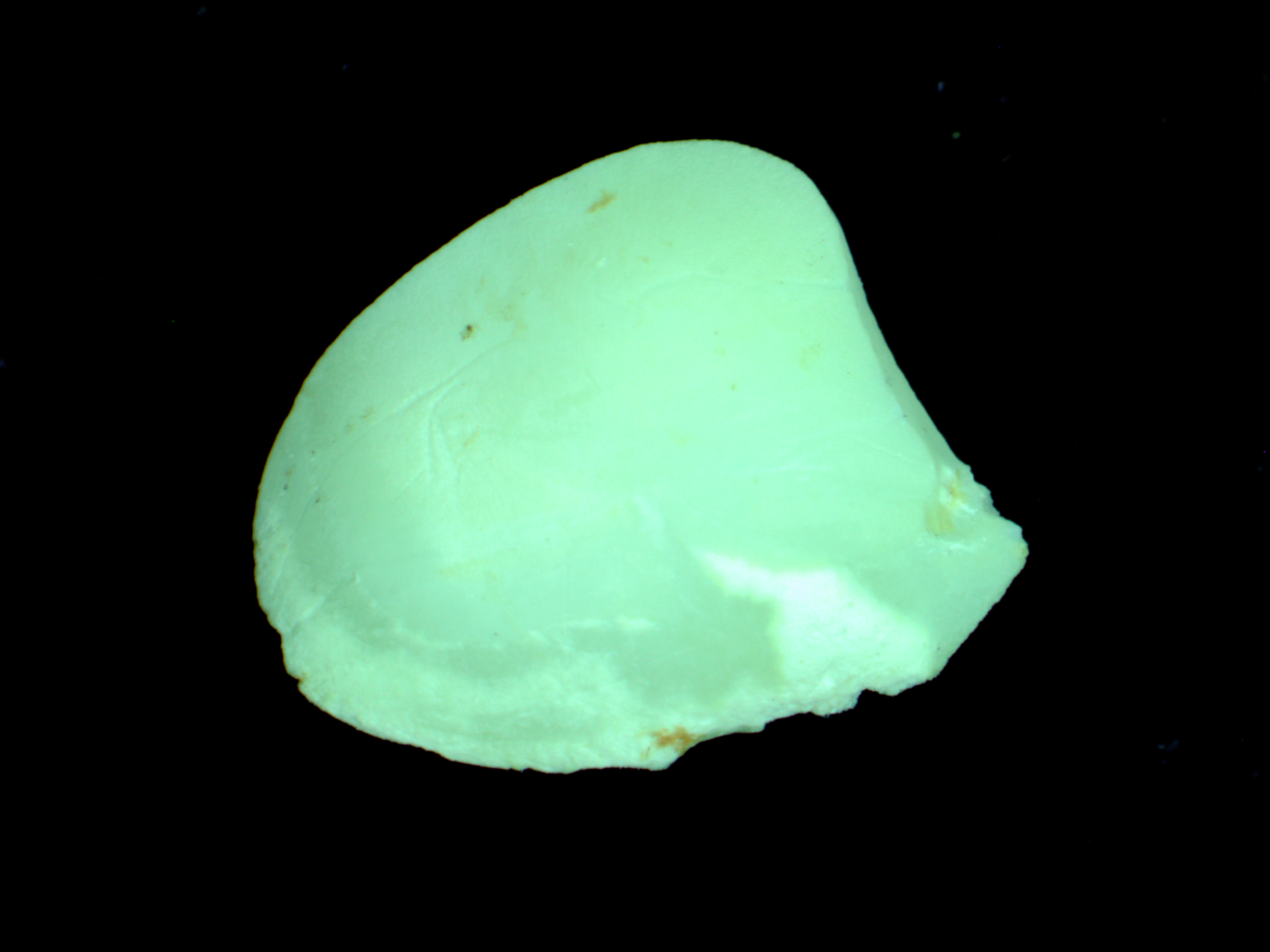

Supplement: Supplemental Information 5 [file peerj-04-1664-s005.zip › Nemcae/testing/ARI932_R1.jpg]

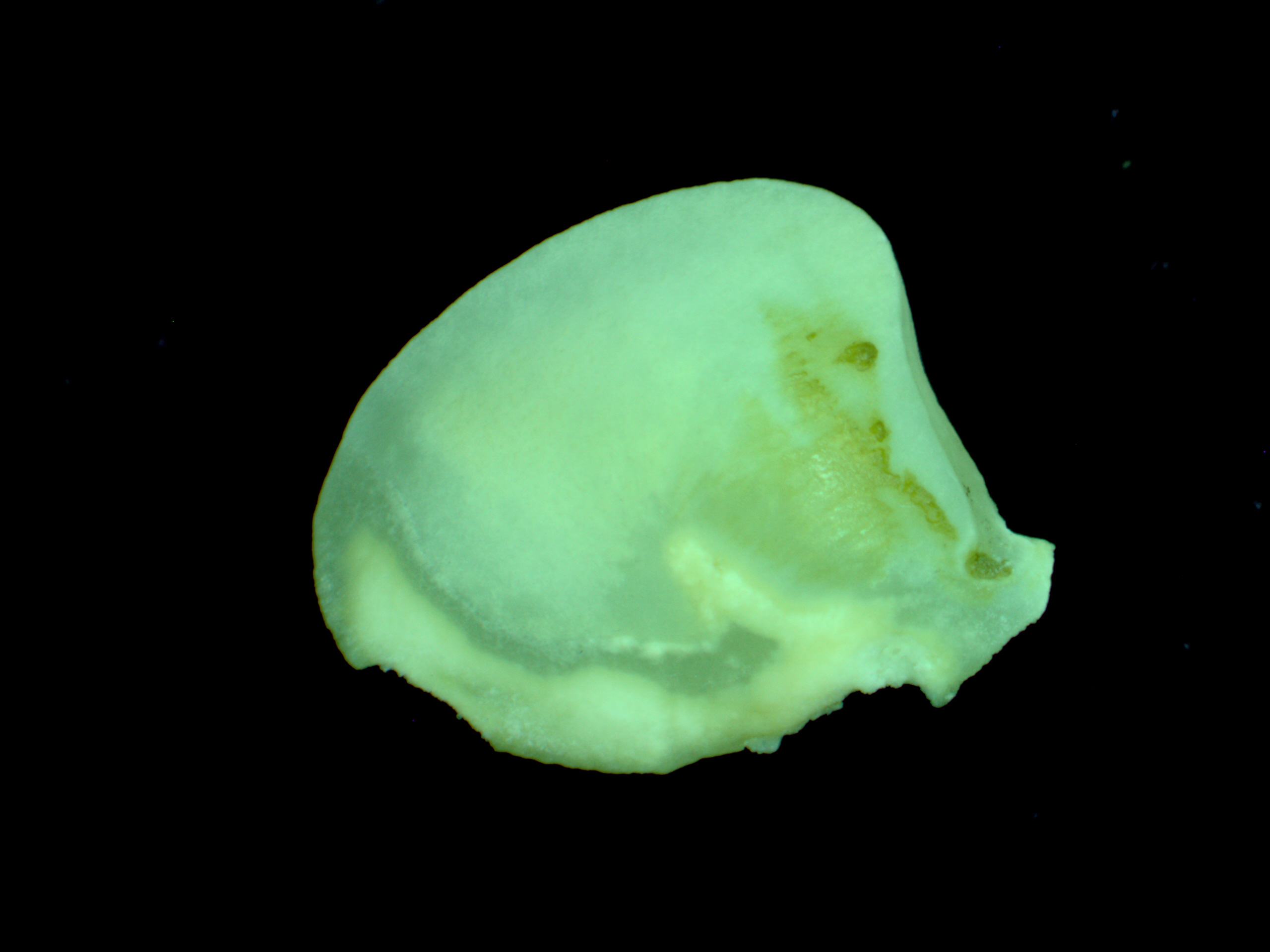

Supplement: Supplemental Information 5 [file peerj-04-1664-s005.zip › Nemcae/testing/ARI933_R1.jpg]

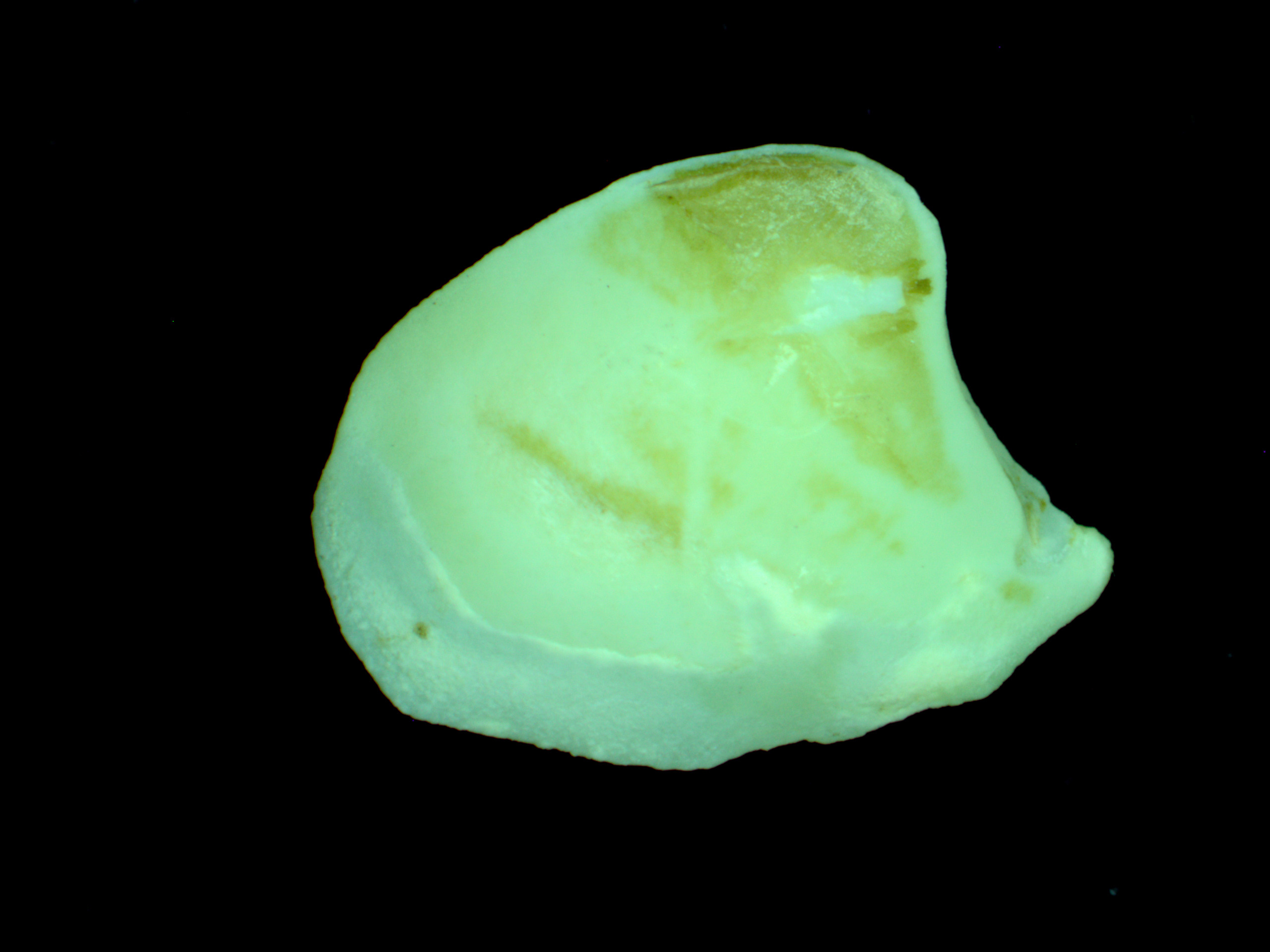

Supplement: Supplemental Information 5 [file peerj-04-1664-s005.zip › Nemcae/testing/ARI935_R1.jpg]

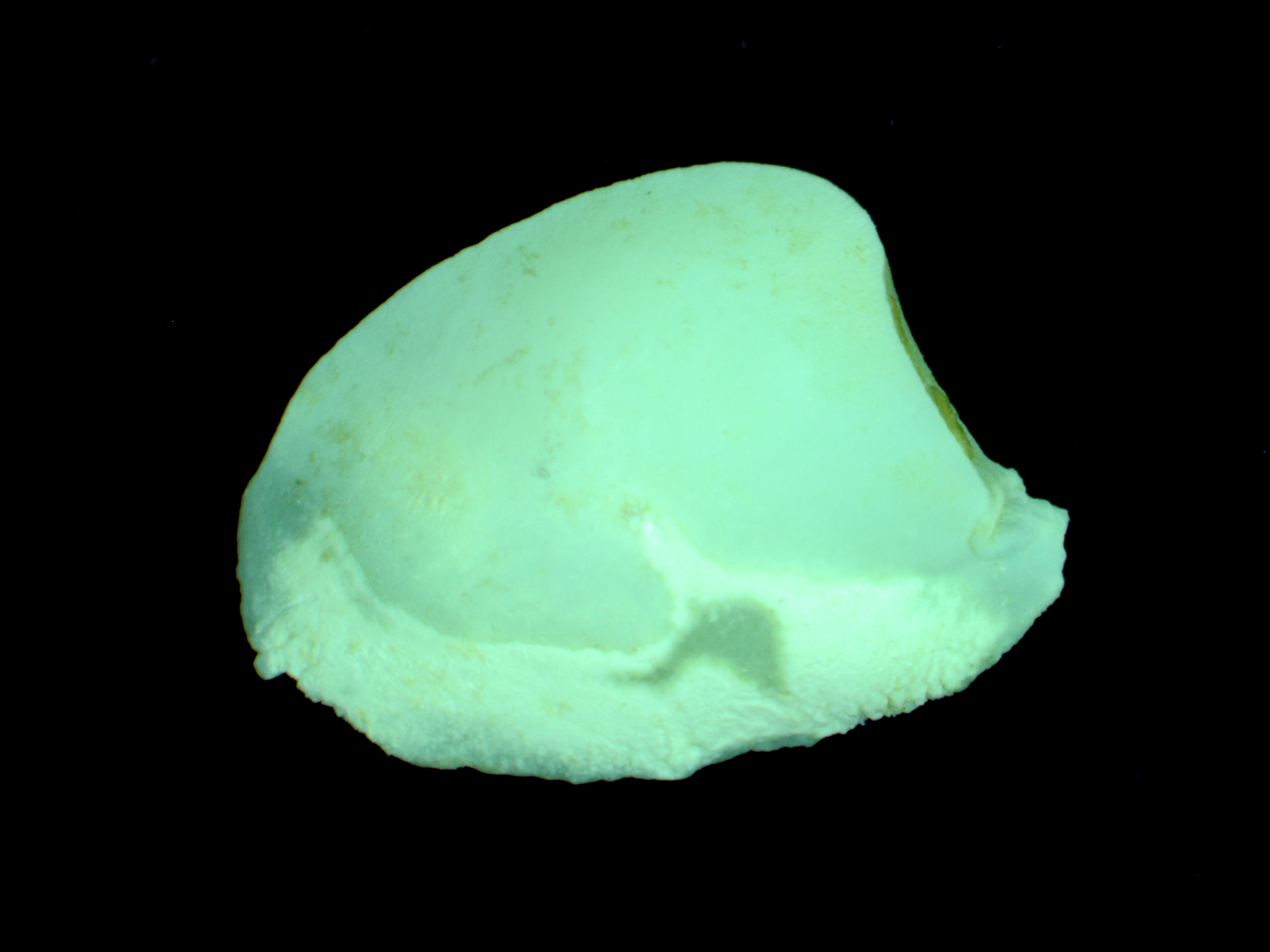

Supplement: Supplemental Information 5 [file peerj-04-1664-s005.zip › Nemcae/testing/ARI936_R1.jpg]

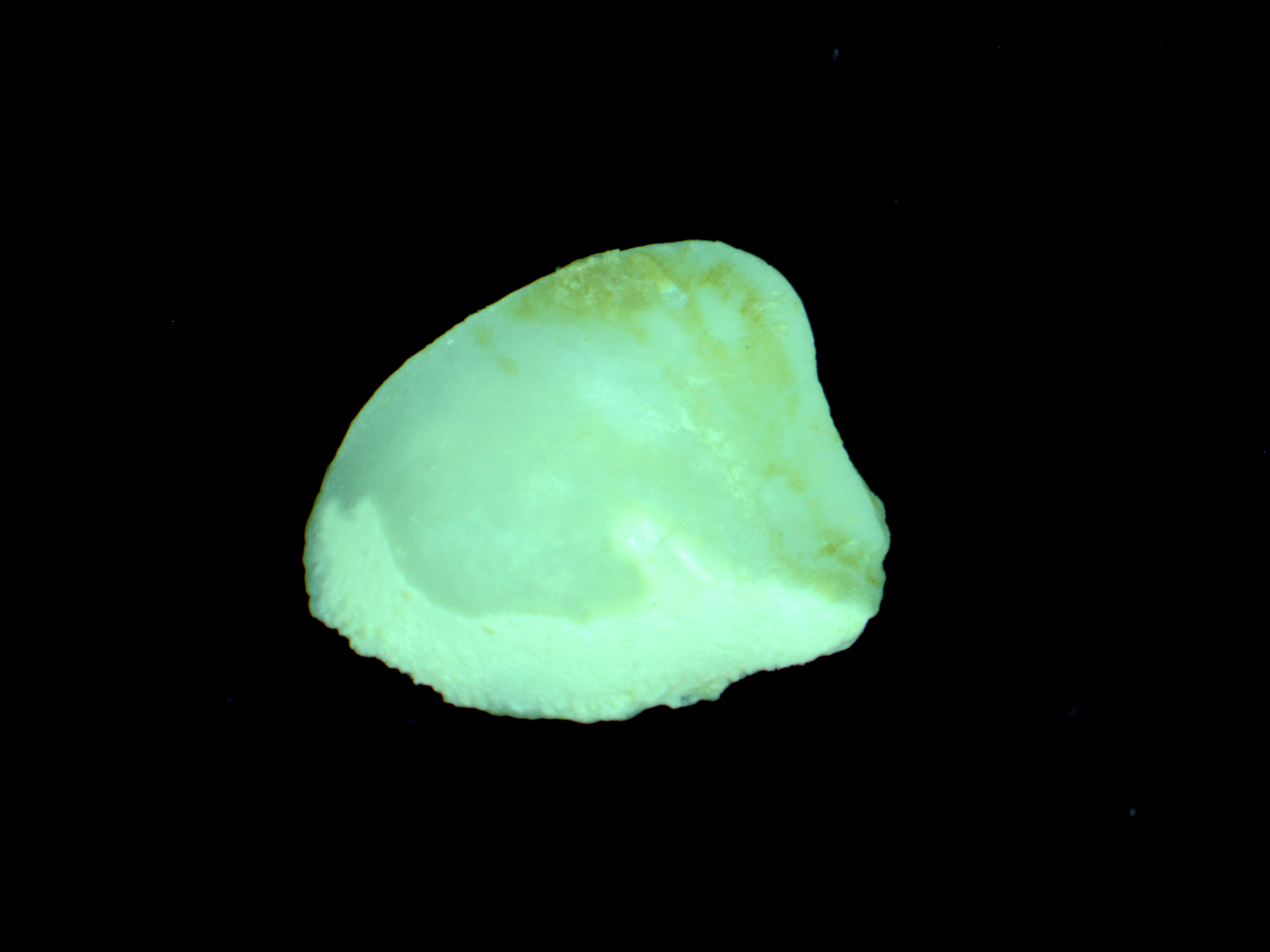

Supplement: Supplemental Information 5 [file peerj-04-1664-s005.zip › Nemcae/testing/ARI937_R1.jpg]

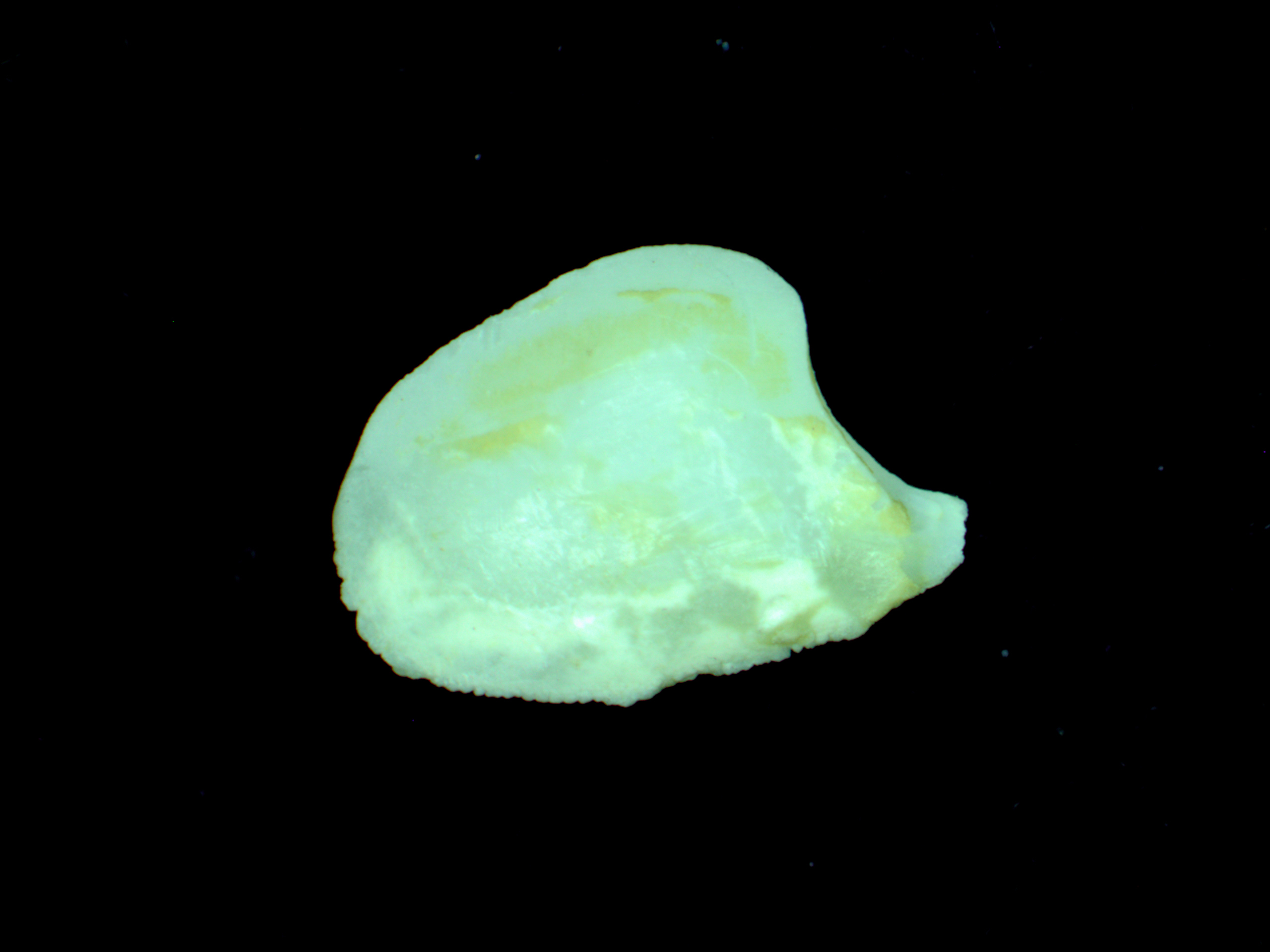

Supplement: Supplemental Information 5 [file peerj-04-1664-s005.zip › Nemcae/testing/ARI975_R1.jpg]

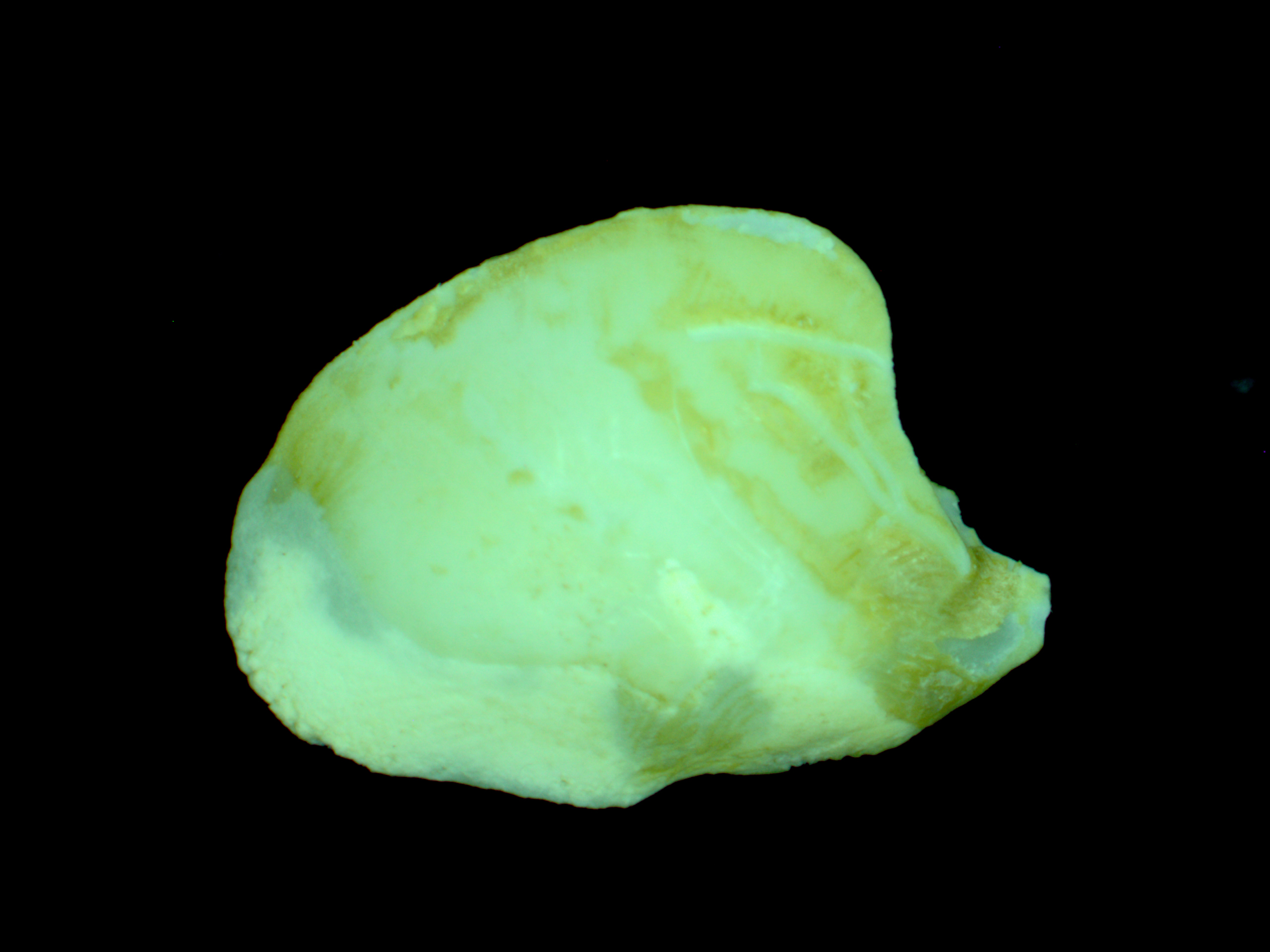

Supplement: Supplemental Information 5 [file peerj-04-1664-s005.zip › Nemcae/training/ARI145_R1.jpg]

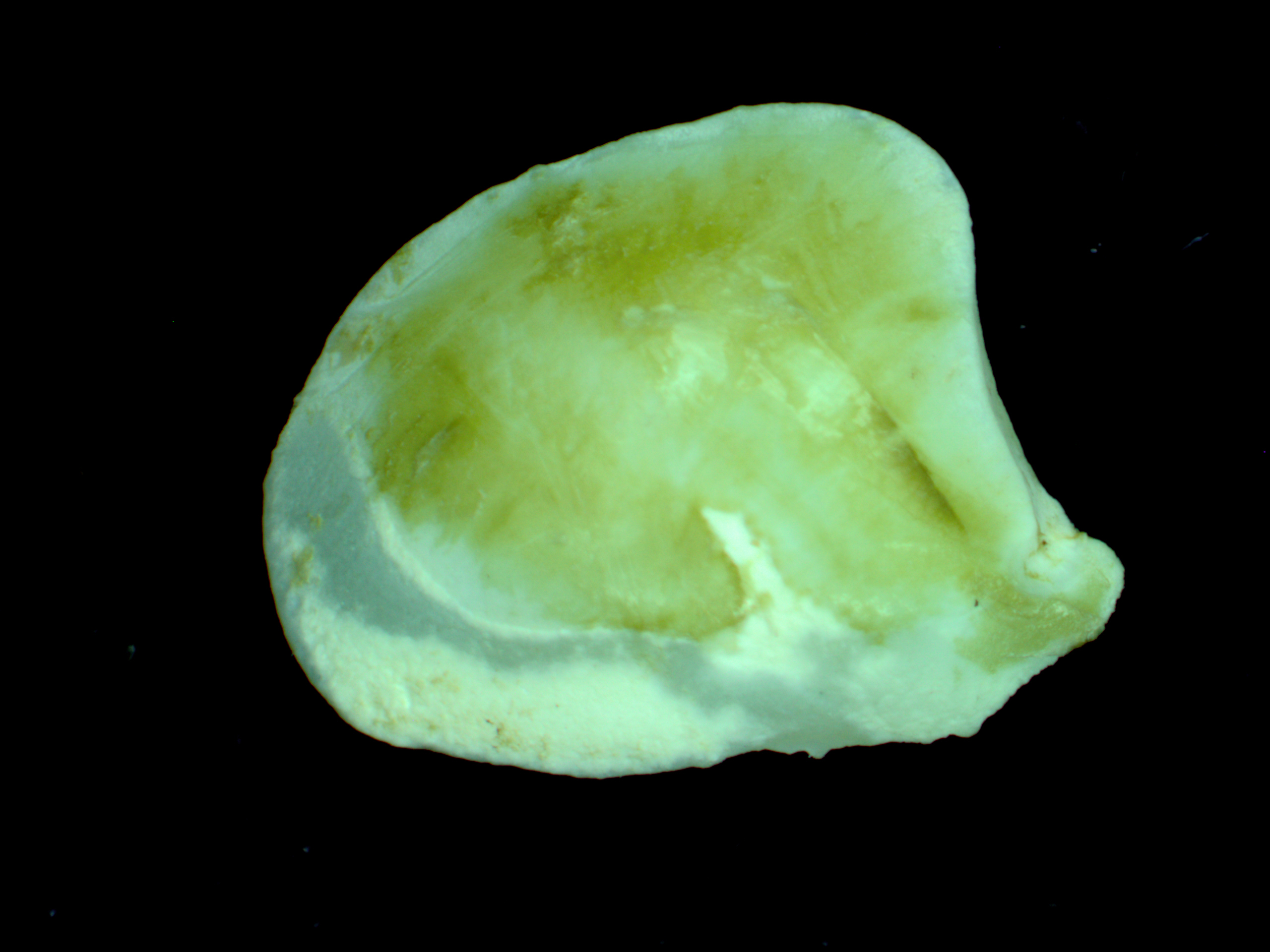

Supplement: Supplemental Information 5 [file peerj-04-1664-s005.zip › Nemcae/training/ARI229_R1.jpg]

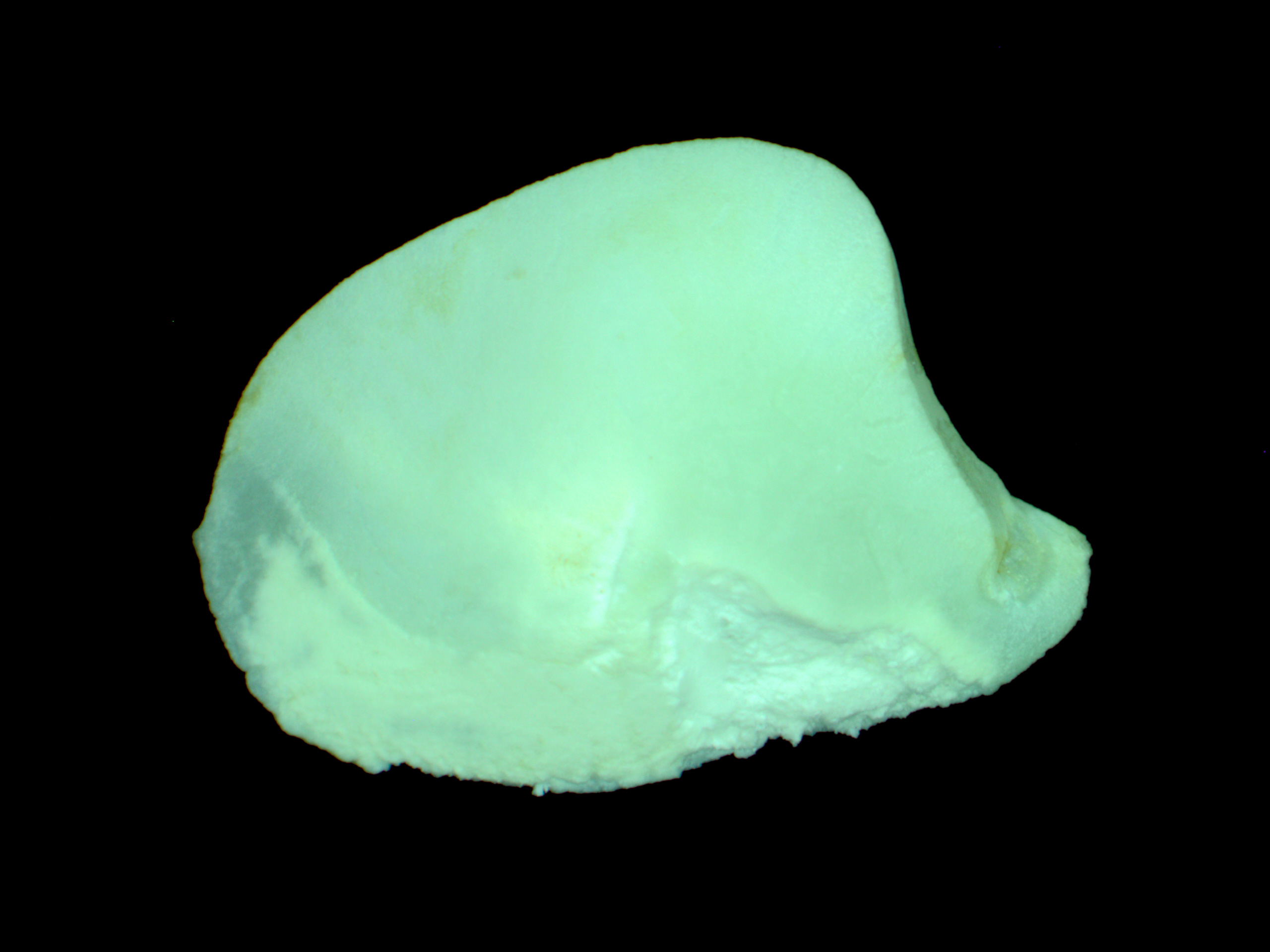

Supplement: Supplemental Information 5 [file peerj-04-1664-s005.zip › Nemcae/training/ARI45_R1.jpg]

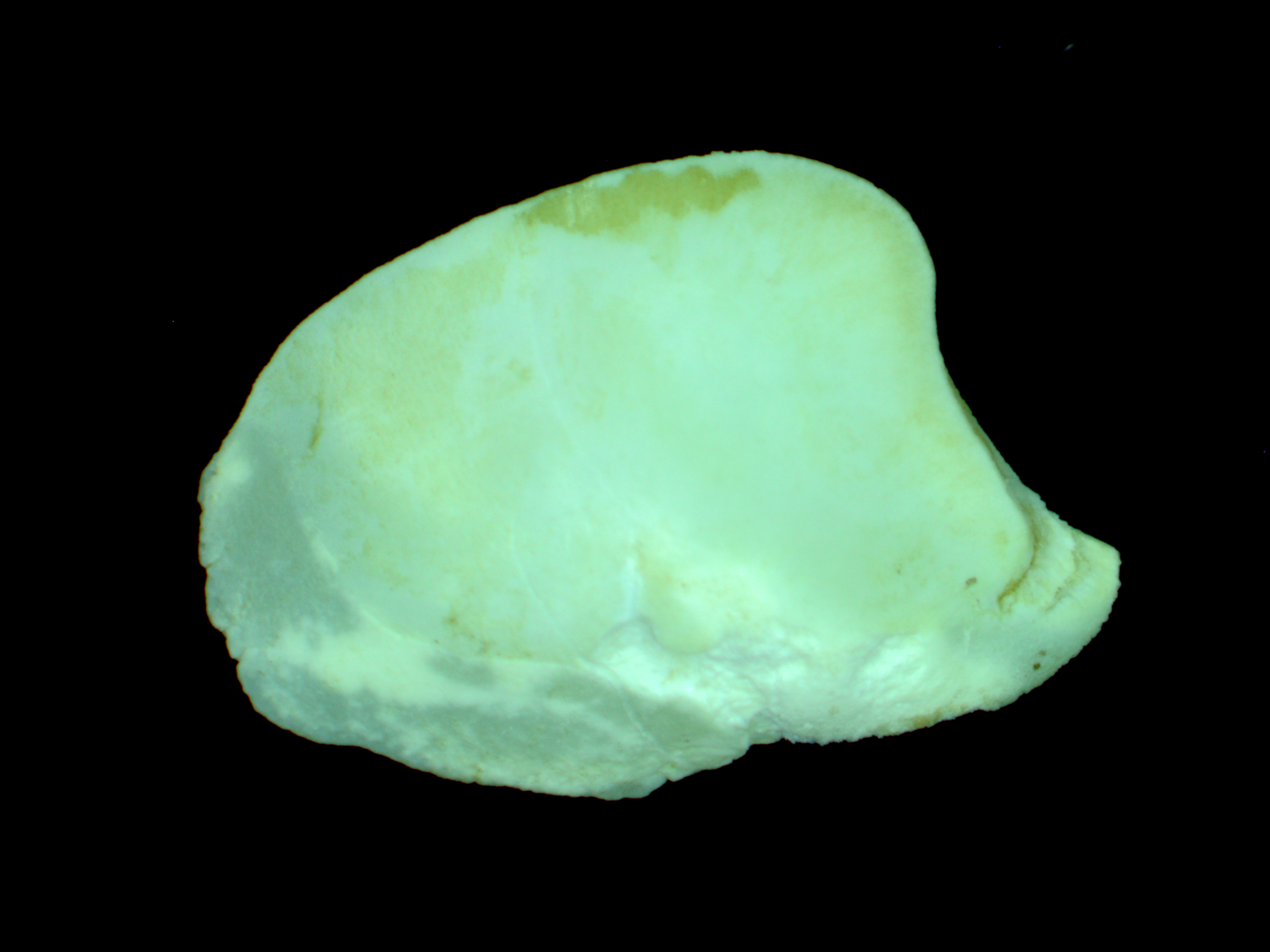

Supplement: Supplemental Information 5 [file peerj-04-1664-s005.zip › Nemcae/training/ARI46_R1.jpg]

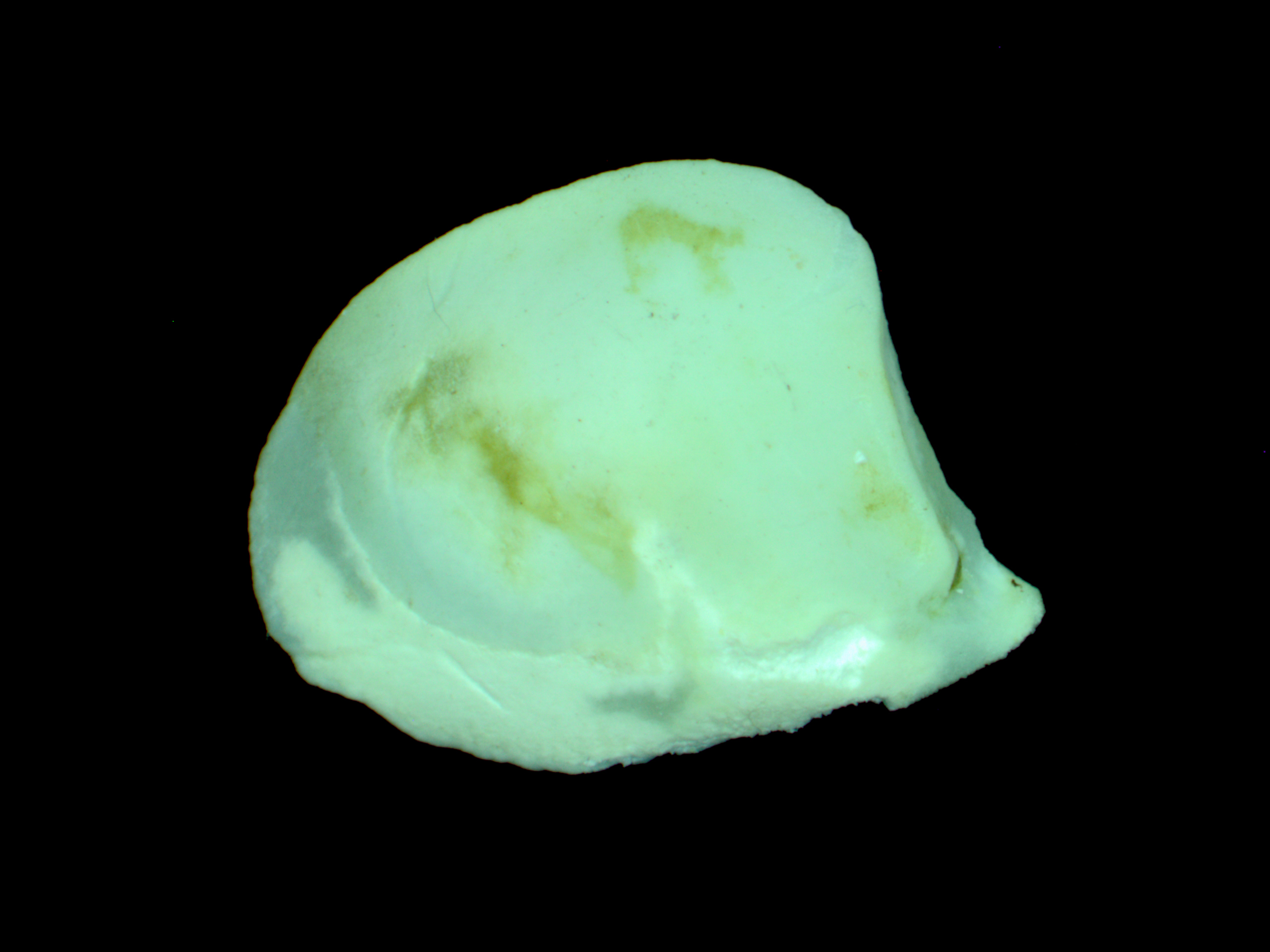

Supplement: Supplemental Information 5 [file peerj-04-1664-s005.zip › Nemcae/training/ARI47_R1.jpg]

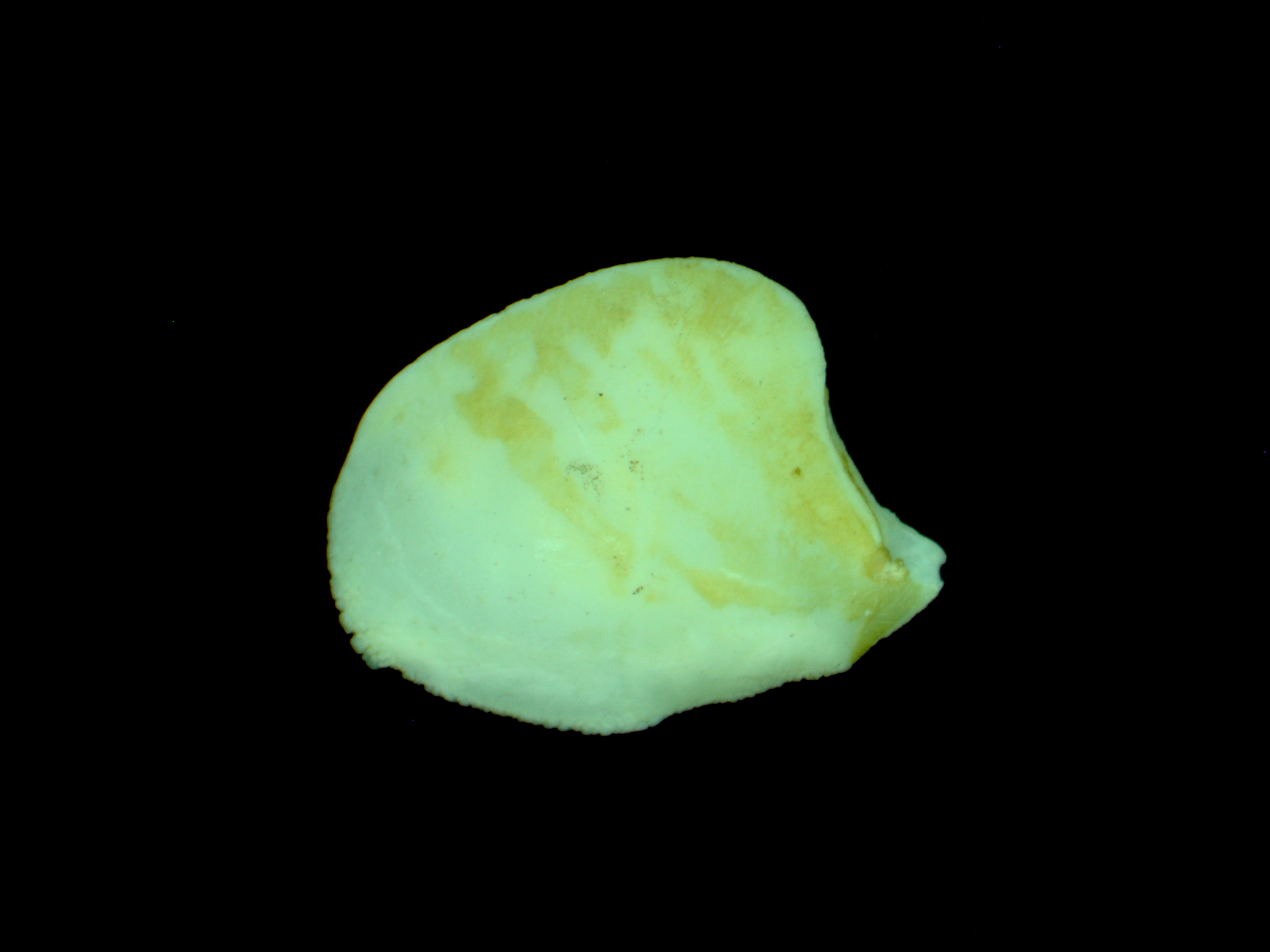

Supplement: Supplemental Information 5 [file peerj-04-1664-s005.zip › Nemcae/training/ARI49_R1.jpg]
