# Supplementary figures and images for: Fully-automated identification of fish species based on otolith contour: using short-time Fourier transform and discriminant analysis (STFT-DA) (part 2 of 5)
Source: PeerJ. 2016 Feb 22;4:e1664. doi: 10.7717/peerj.1664 (PMC4768690; doi:10.7717/peerj.1664)

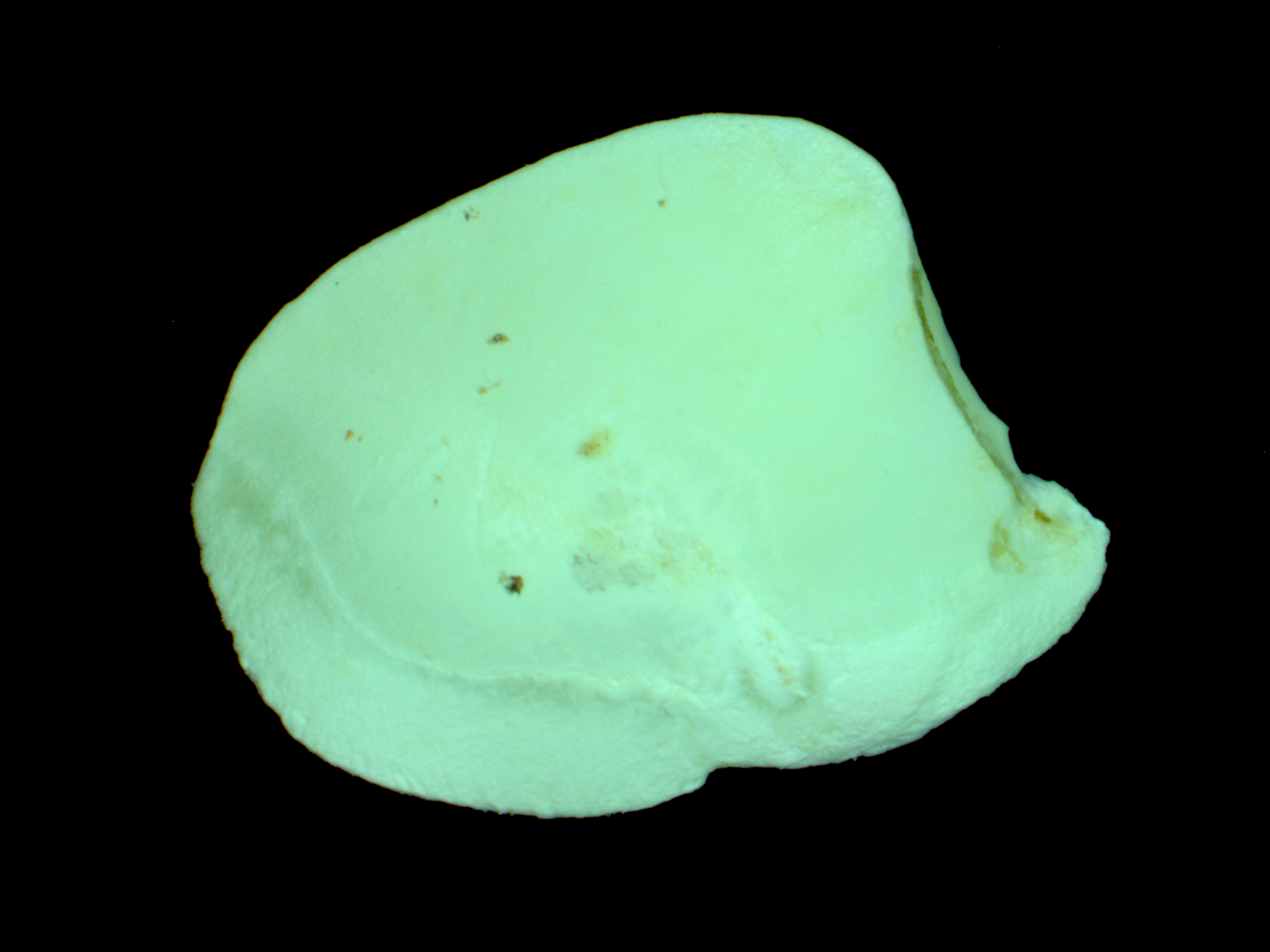

Supplement: Supplemental Information 5 [file peerj-04-1664-s005.zip › Nemcae/training/ARI912_R1.jpg]

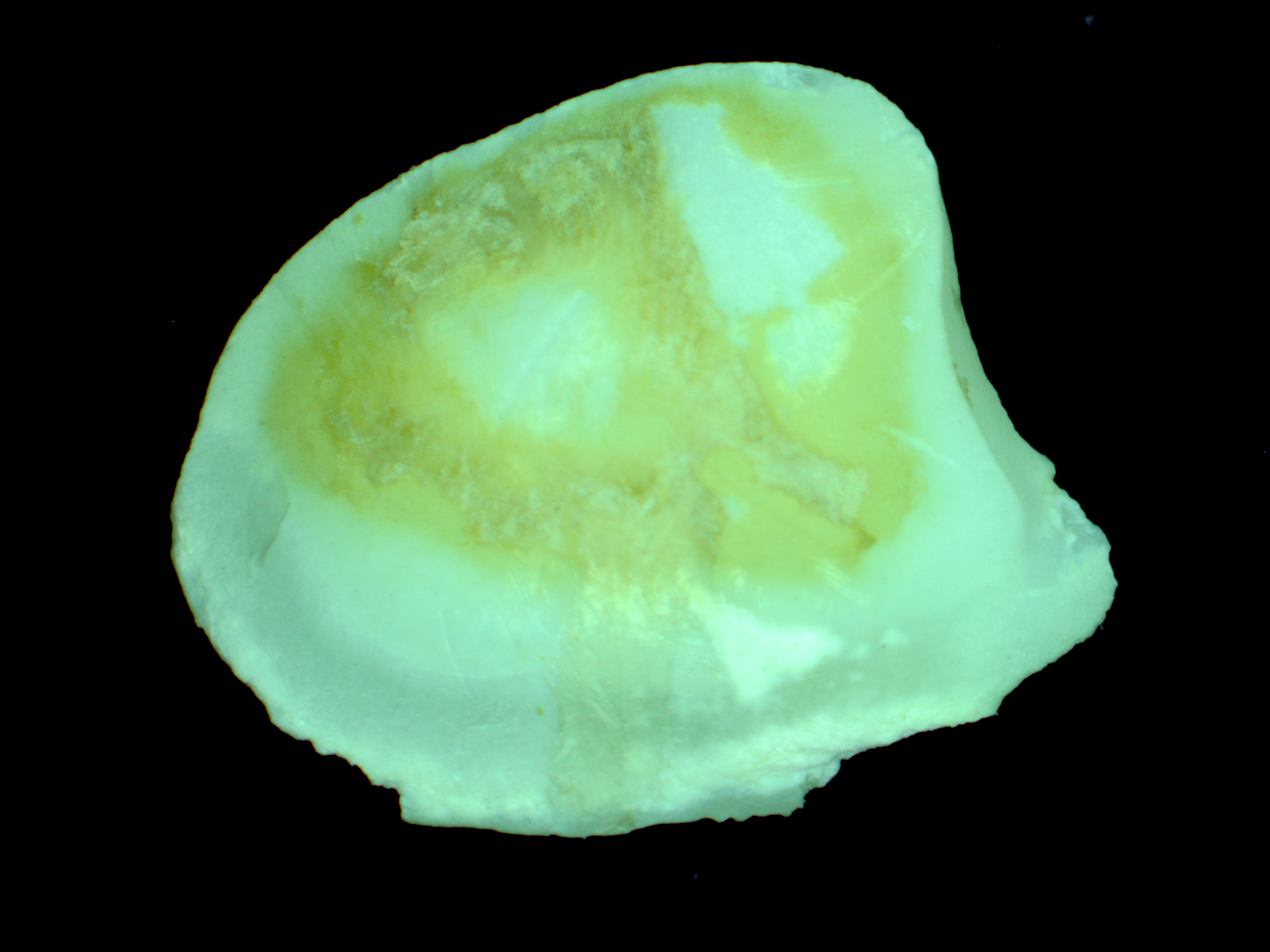

Supplement: Supplemental Information 5 [file peerj-04-1664-s005.zip › Nemcae/training/ARI913_R1.jpg]

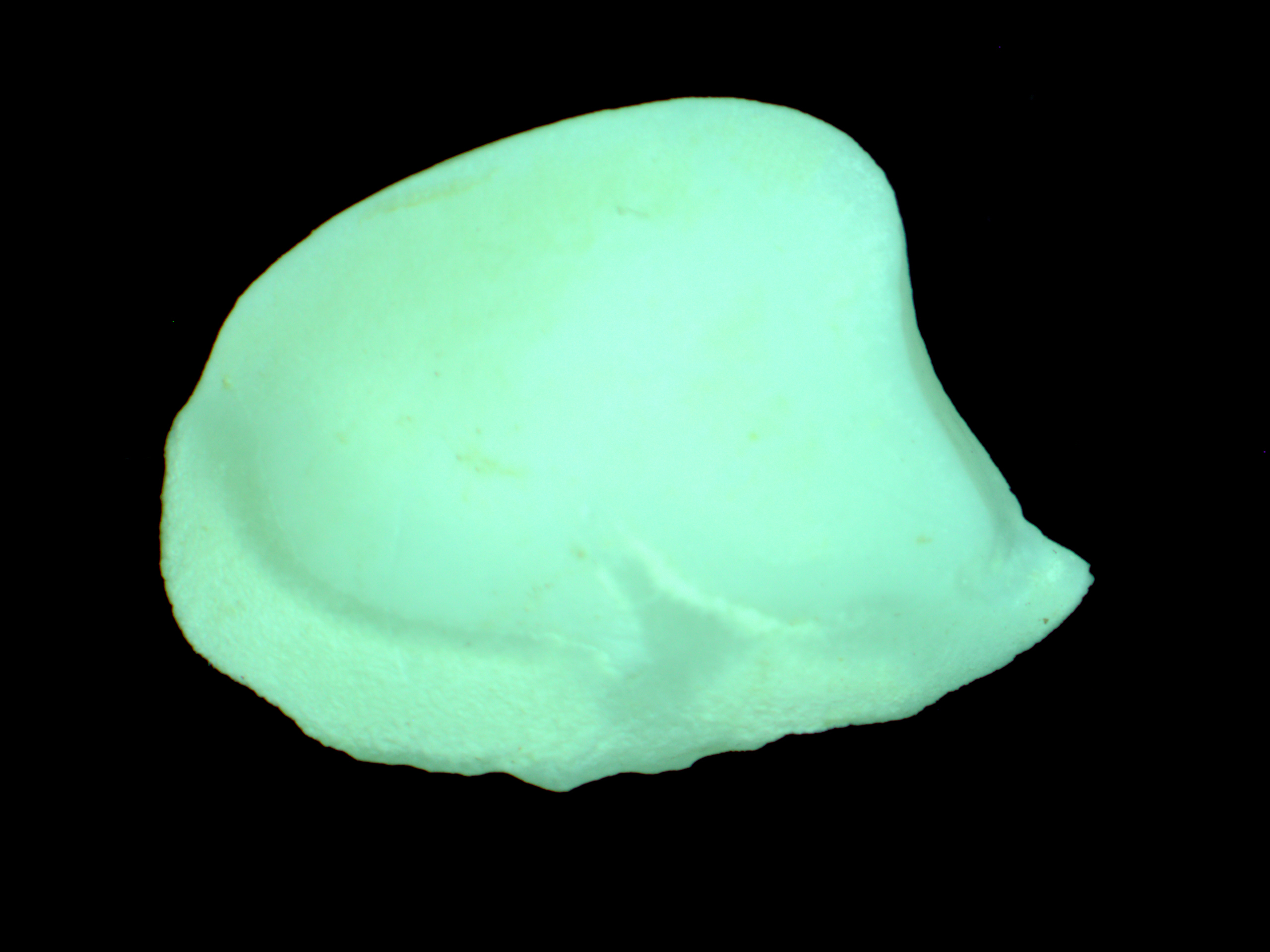

Supplement: Supplemental Information 5 [file peerj-04-1664-s005.zip › Nemcae/training/ARI914_R1.jpg]

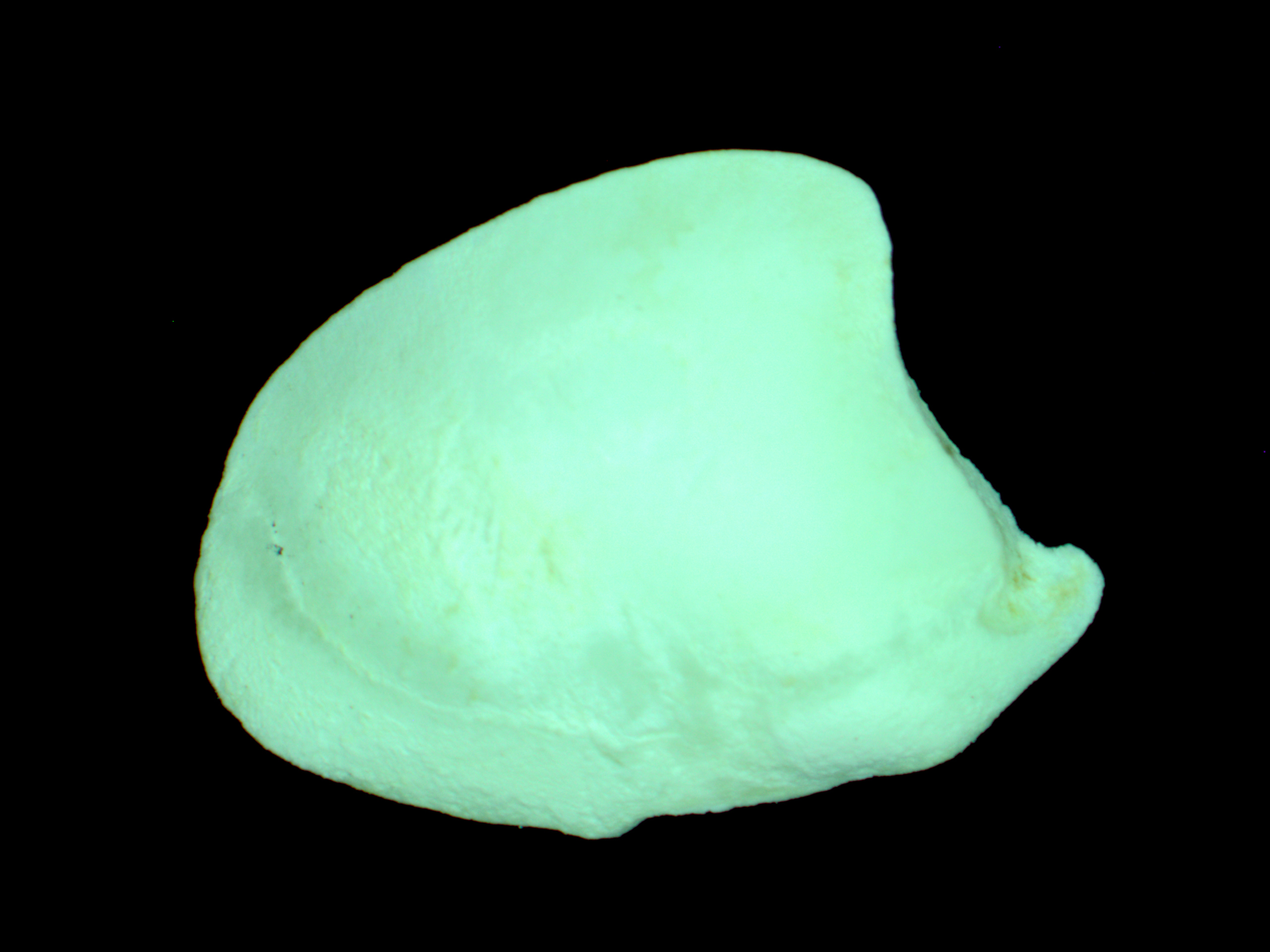

Supplement: Supplemental Information 5 [file peerj-04-1664-s005.zip › Nemcae/training/ARI915_R1.jpg]

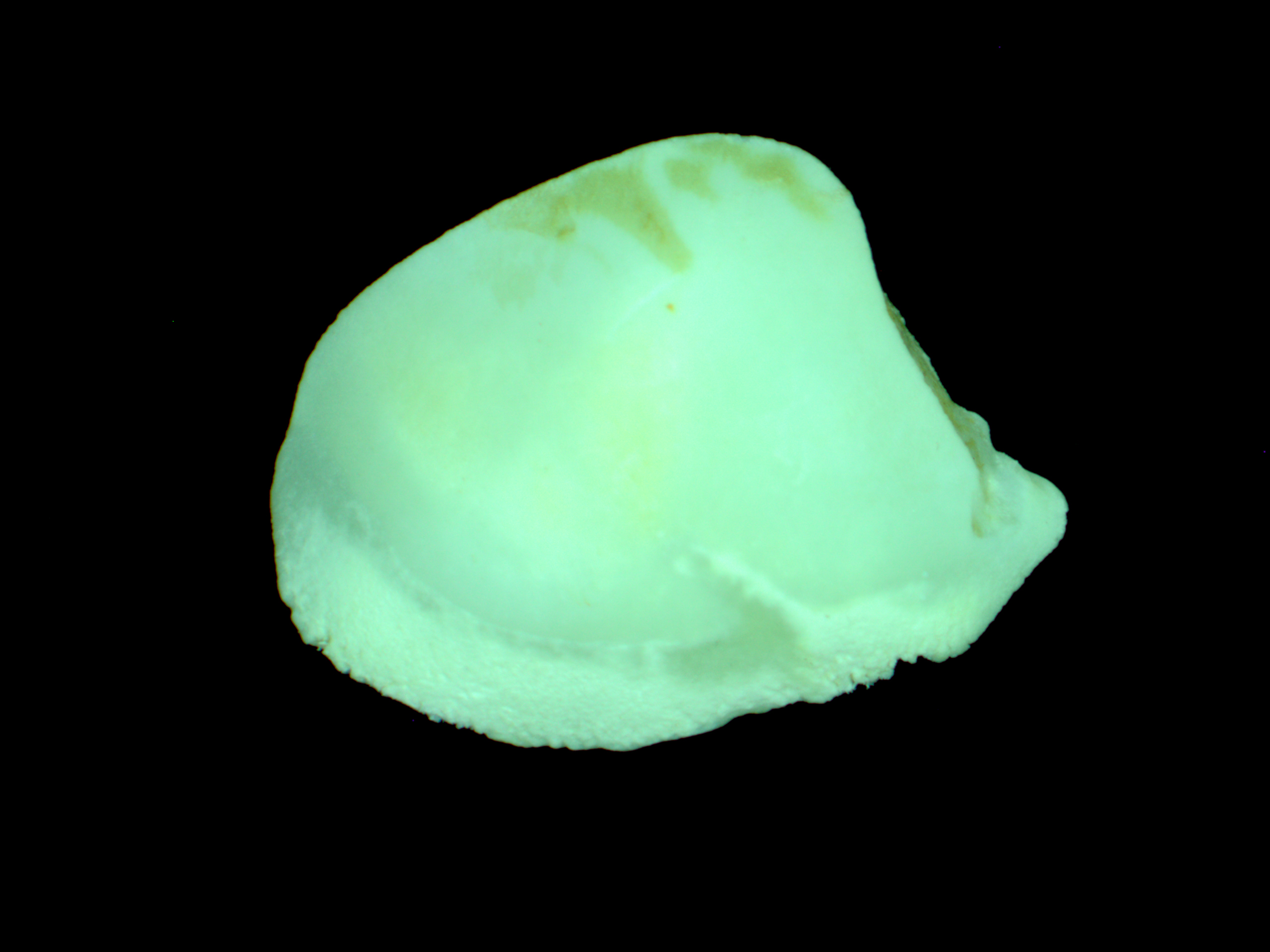

Supplement: Supplemental Information 5 [file peerj-04-1664-s005.zip › Nemcae/training/ARI916_R1.jpg]

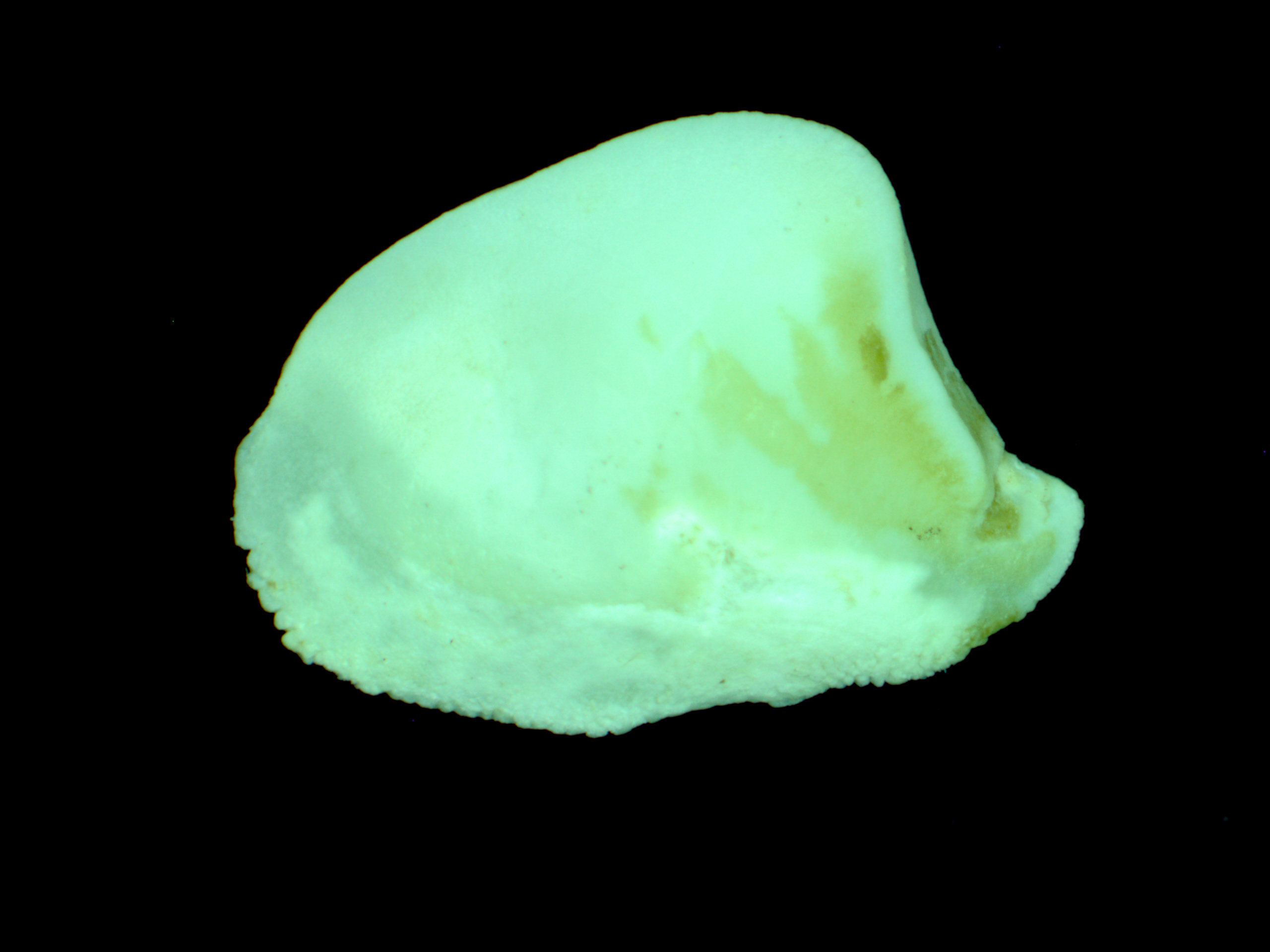

Supplement: Supplemental Information 5 [file peerj-04-1664-s005.zip › Nemcae/training/ARI917_R1.jpg]

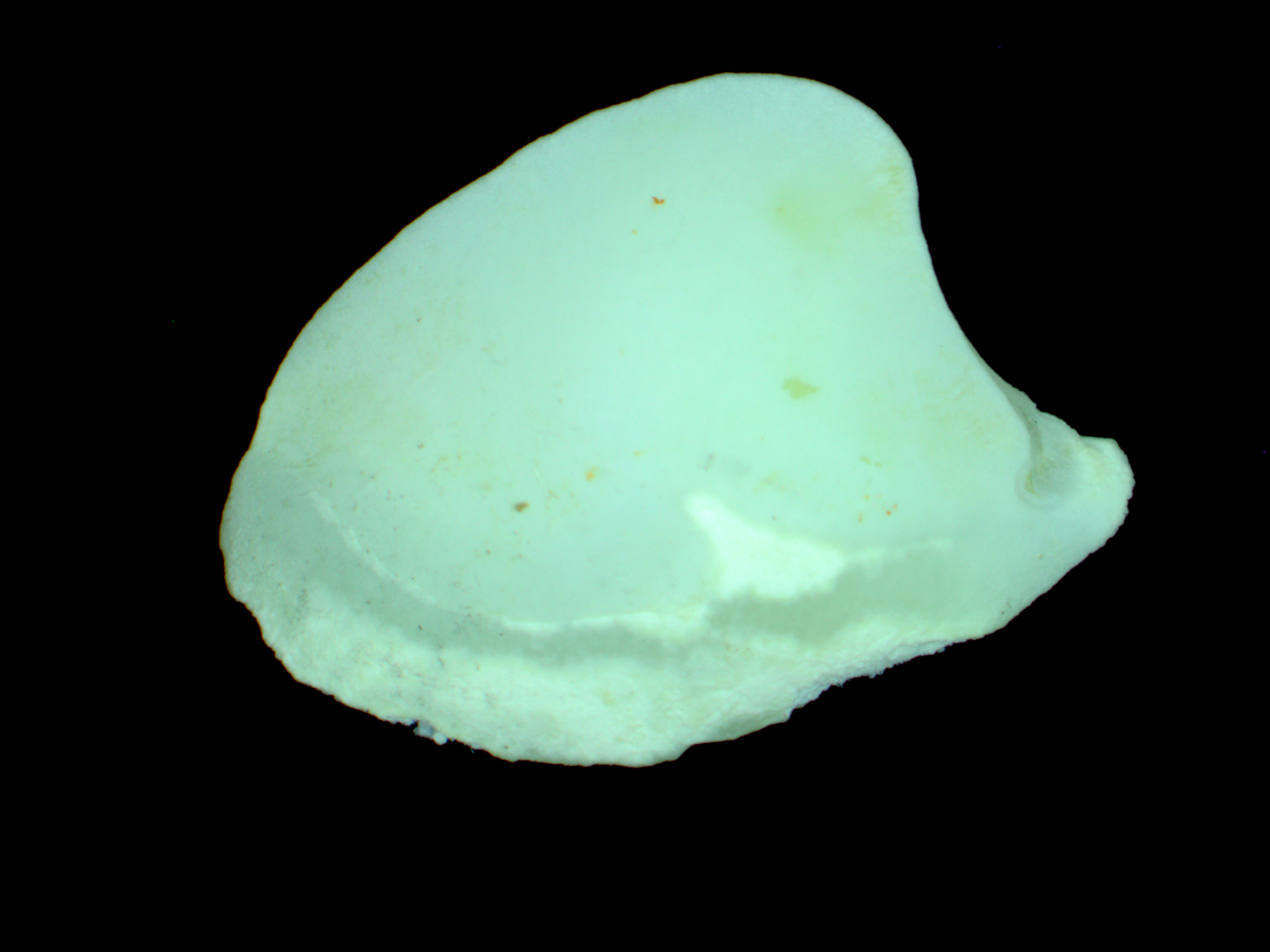

Supplement: Supplemental Information 5 [file peerj-04-1664-s005.zip › Nemcae/training/ARI918_R1.jpg]

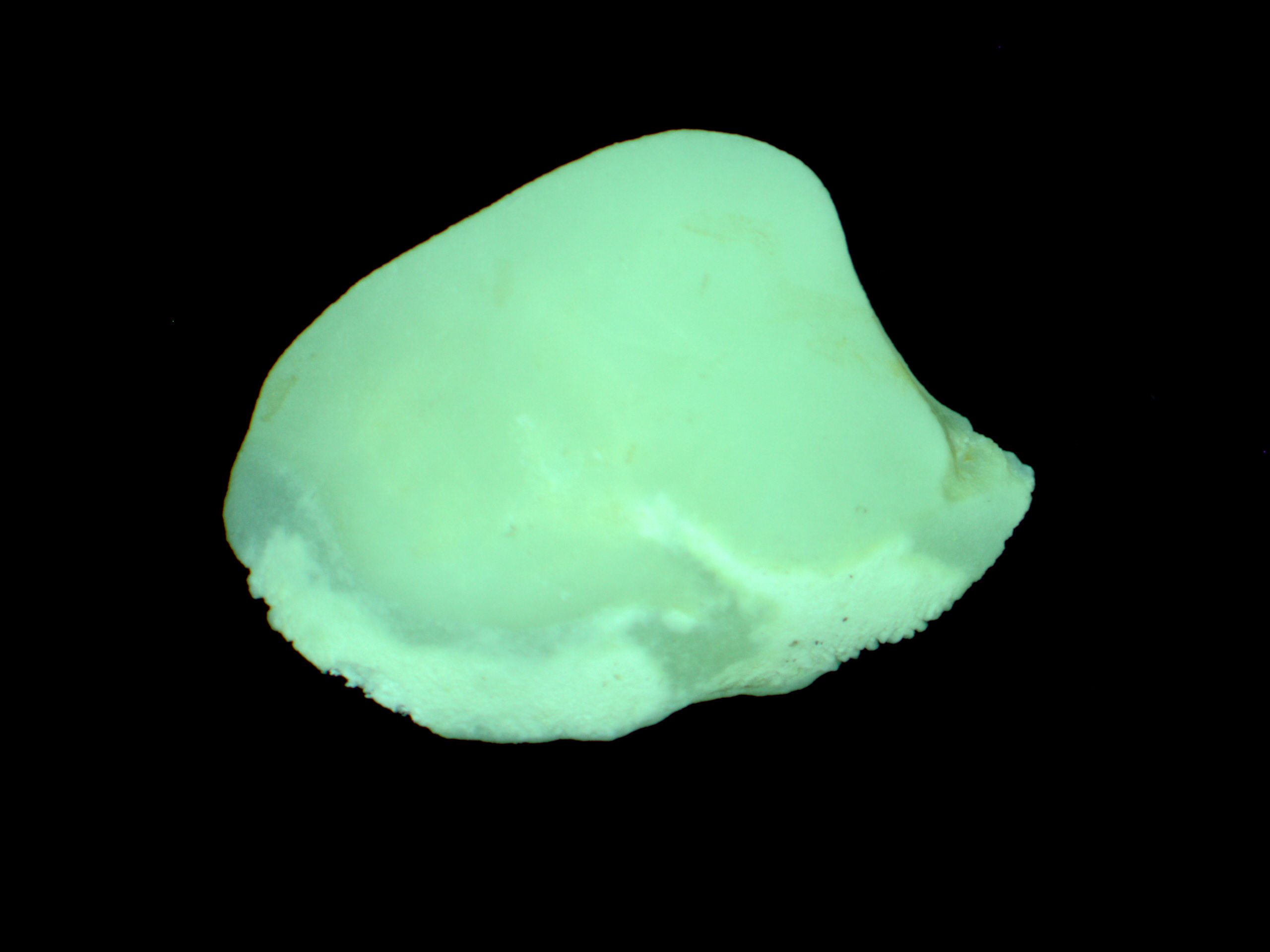

Supplement: Supplemental Information 5 [file peerj-04-1664-s005.zip › Nemcae/training/ARI919_R1.jpg]

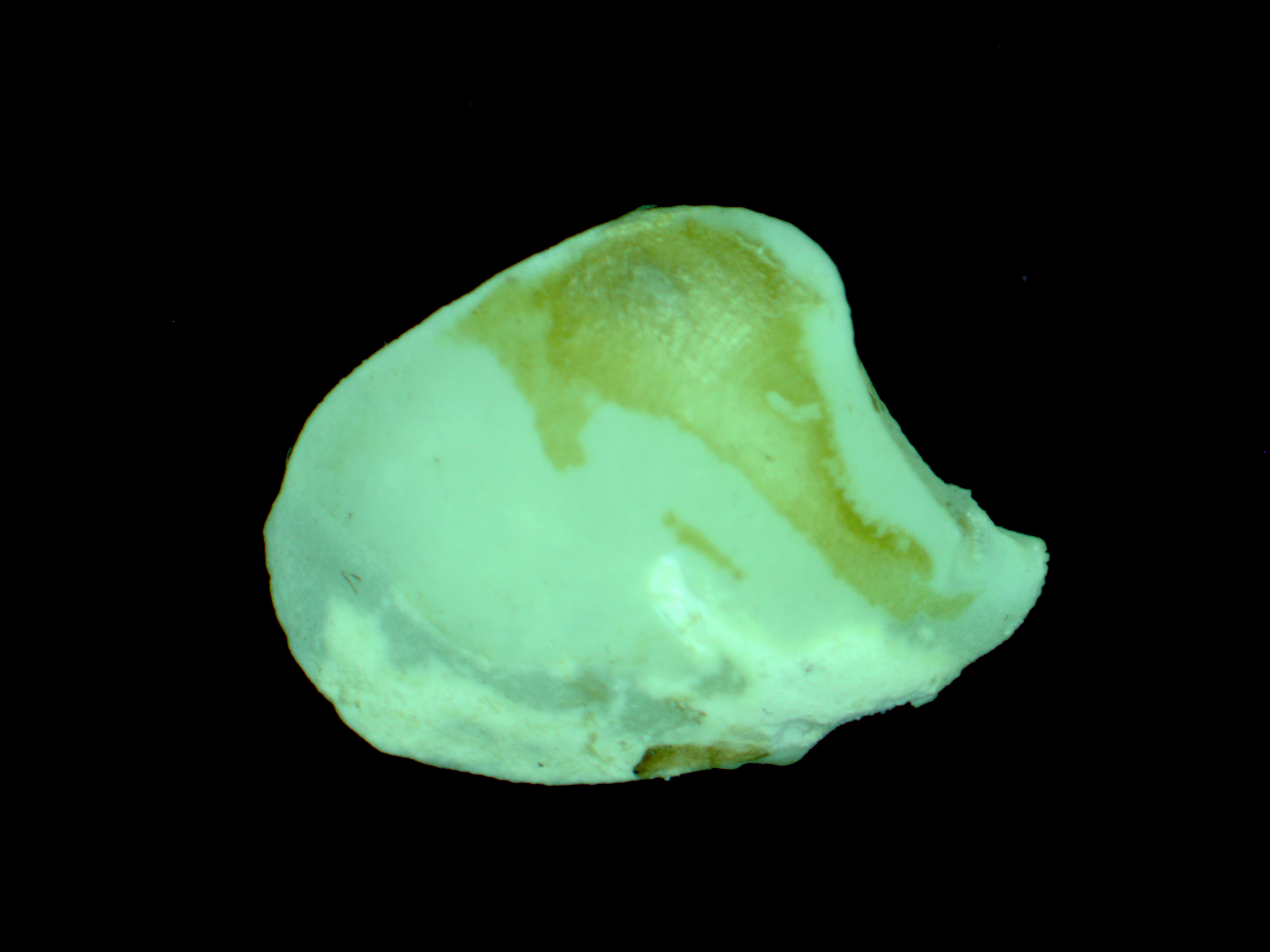

Supplement: Supplemental Information 5 [file peerj-04-1664-s005.zip › Nemcae/training/ARI921_R1.jpg]

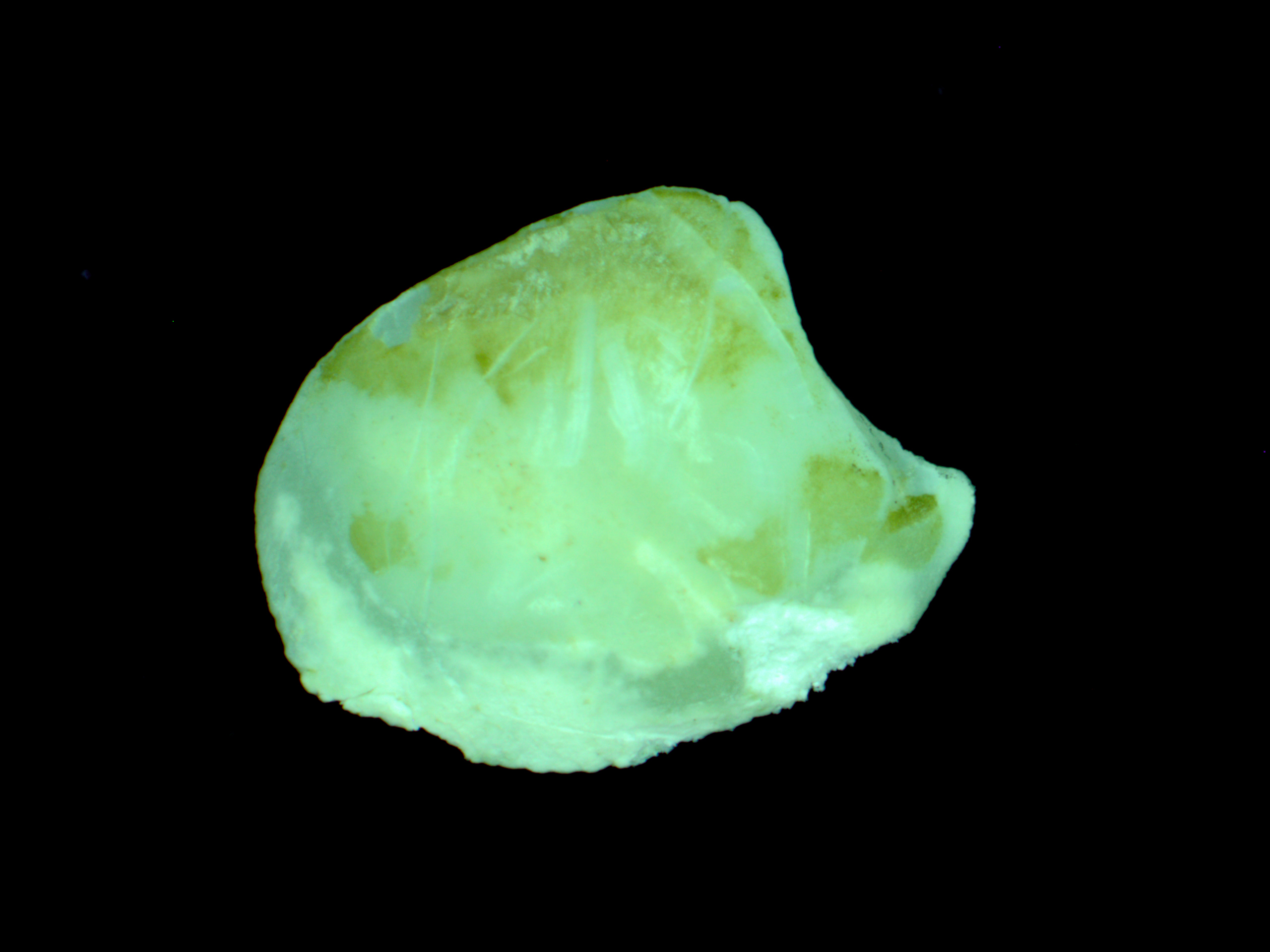

Supplement: Supplemental Information 5 [file peerj-04-1664-s005.zip › Nemcae/training/ARI922_R1.jpg]

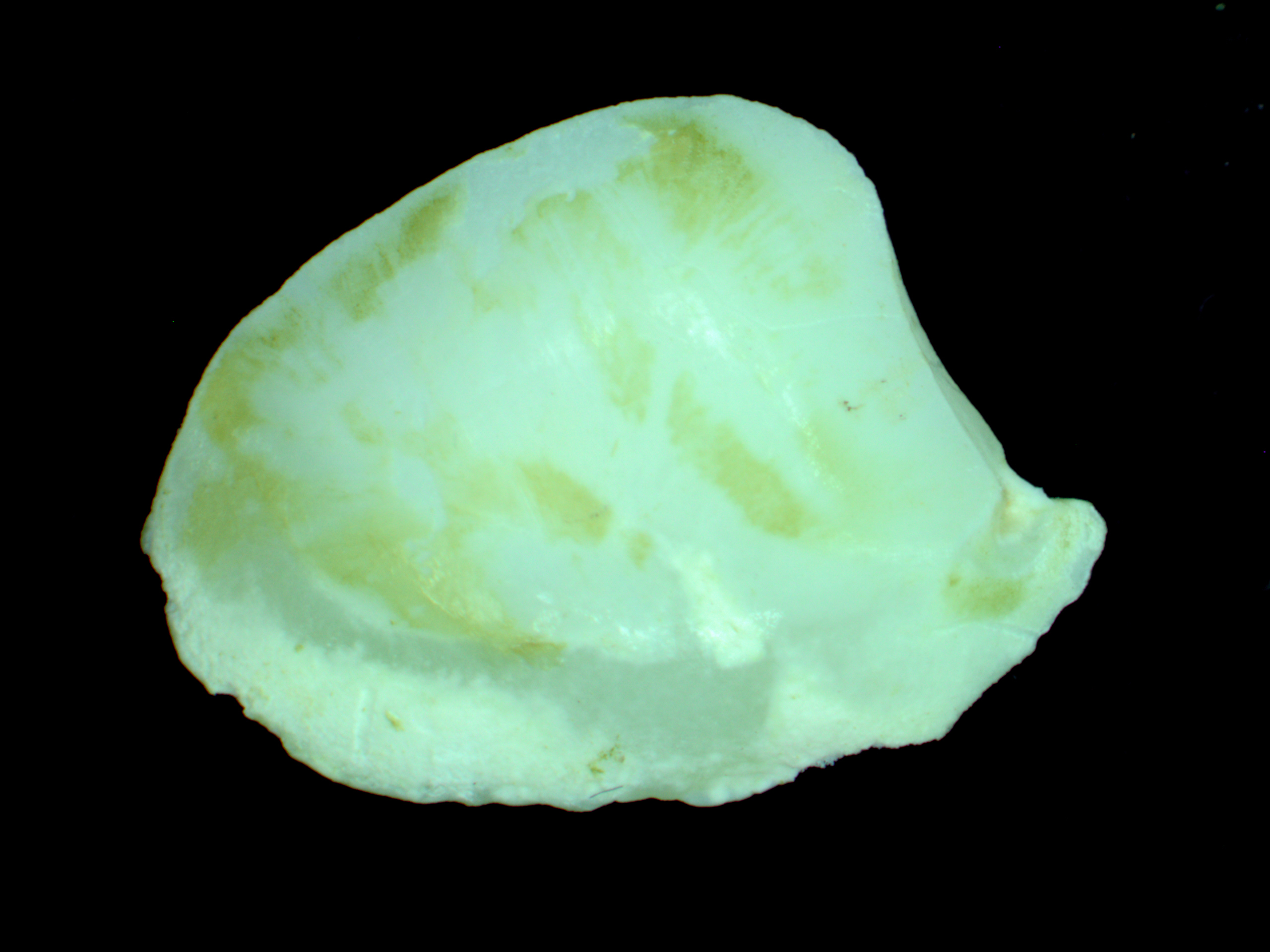

Supplement: Supplemental Information 5 [file peerj-04-1664-s005.zip › Nemcae/training/ARI926_R1.jpg]

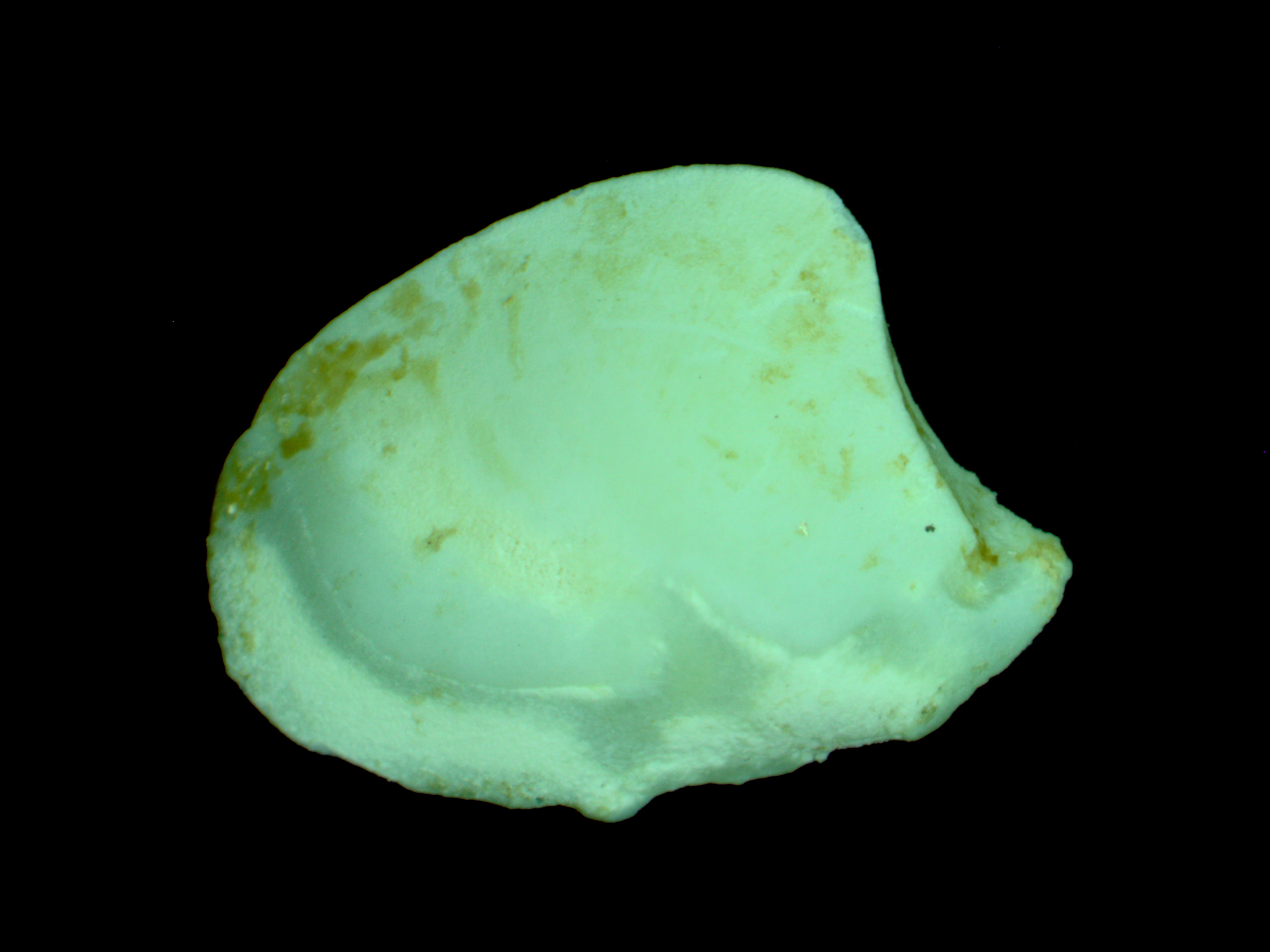

Supplement: Supplemental Information 5 [file peerj-04-1664-s005.zip › Nemcae/training/ARI927_R1.jpg]

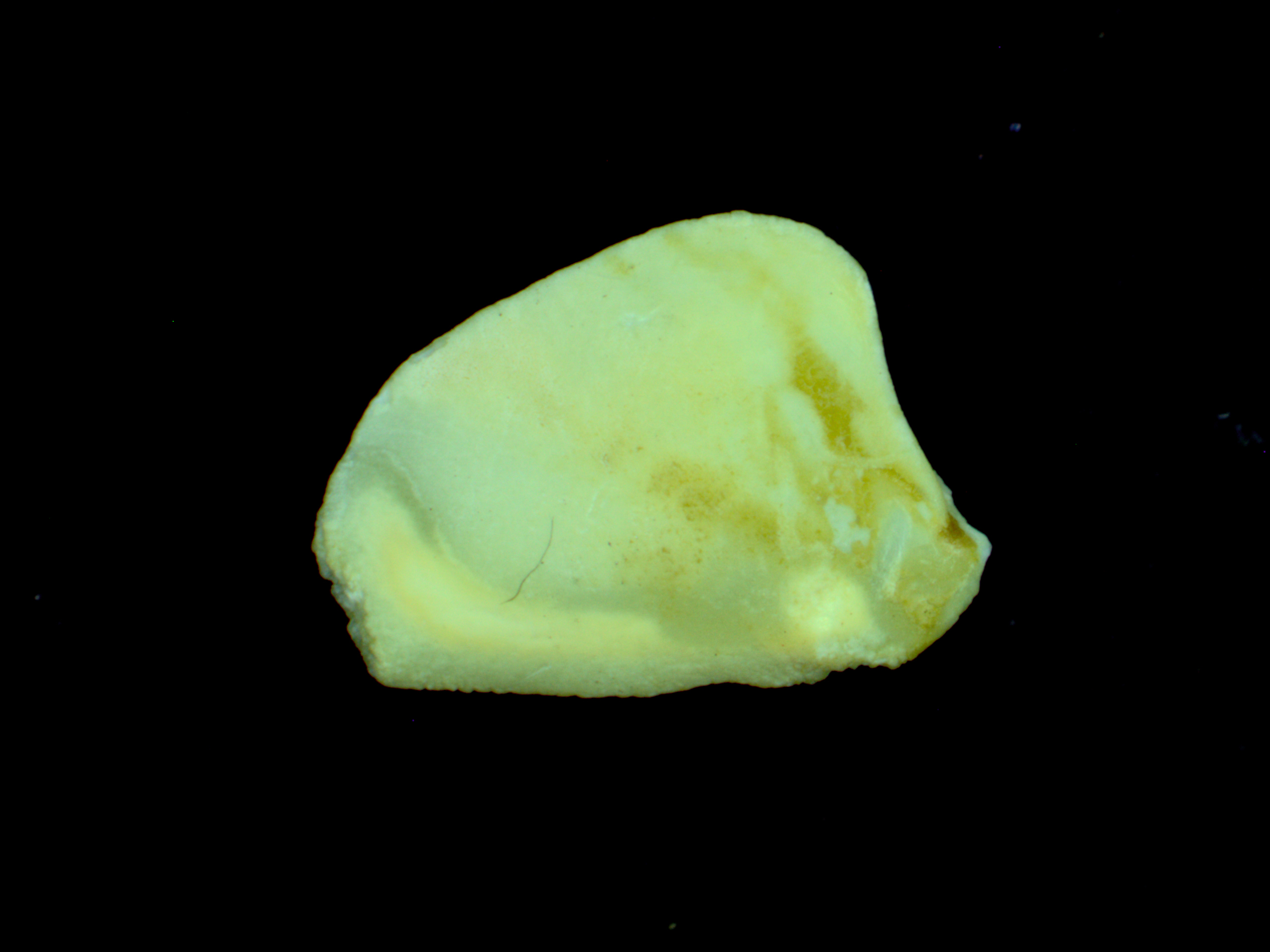

Supplement: Supplemental Information 6 [file peerj-04-1664-s006.zip › OstMil/testing/ARI1068_R1.jpg]

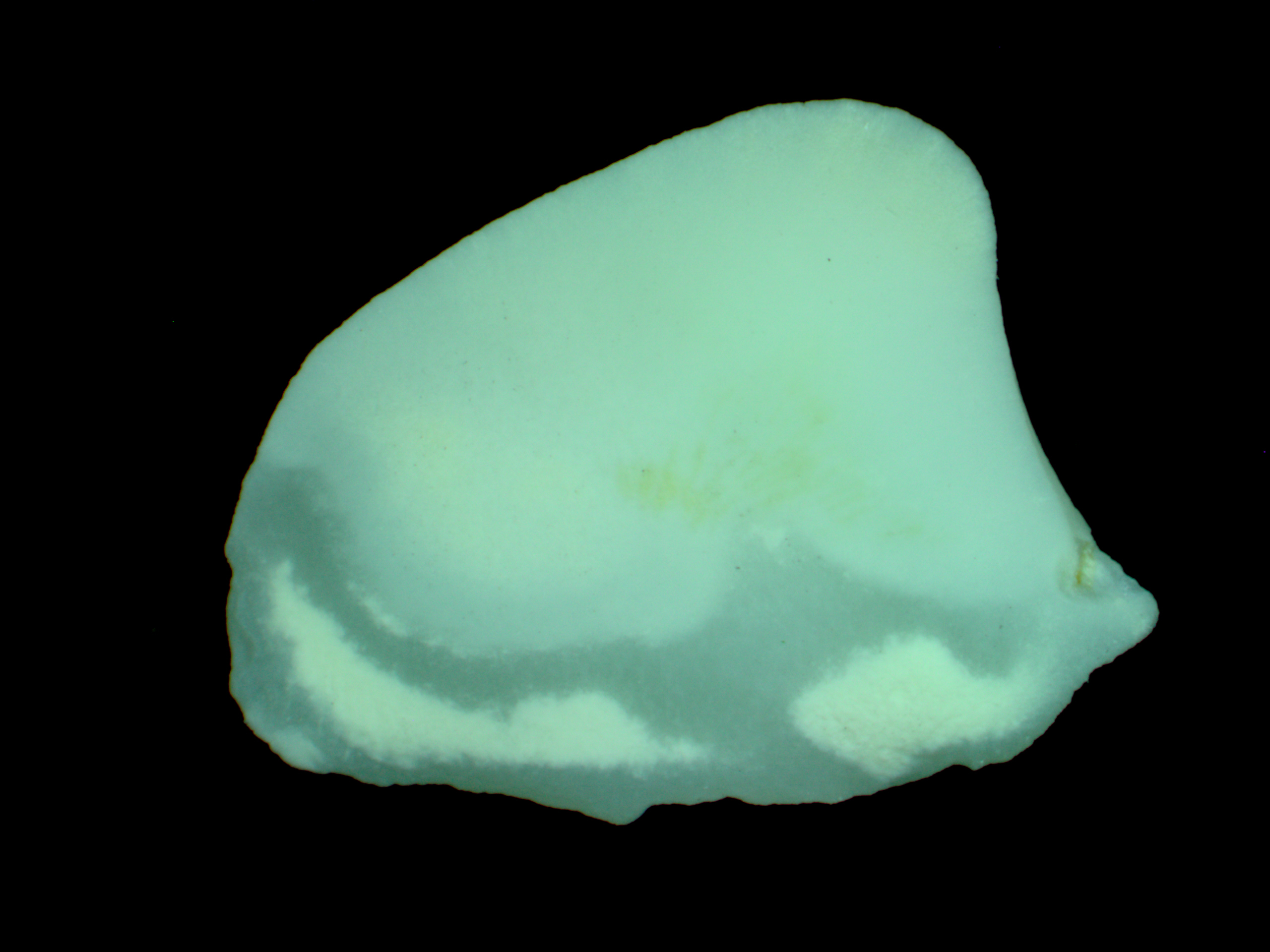

Supplement: Supplemental Information 6 [file peerj-04-1664-s006.zip › OstMil/testing/ARI1083_R1.jpg]

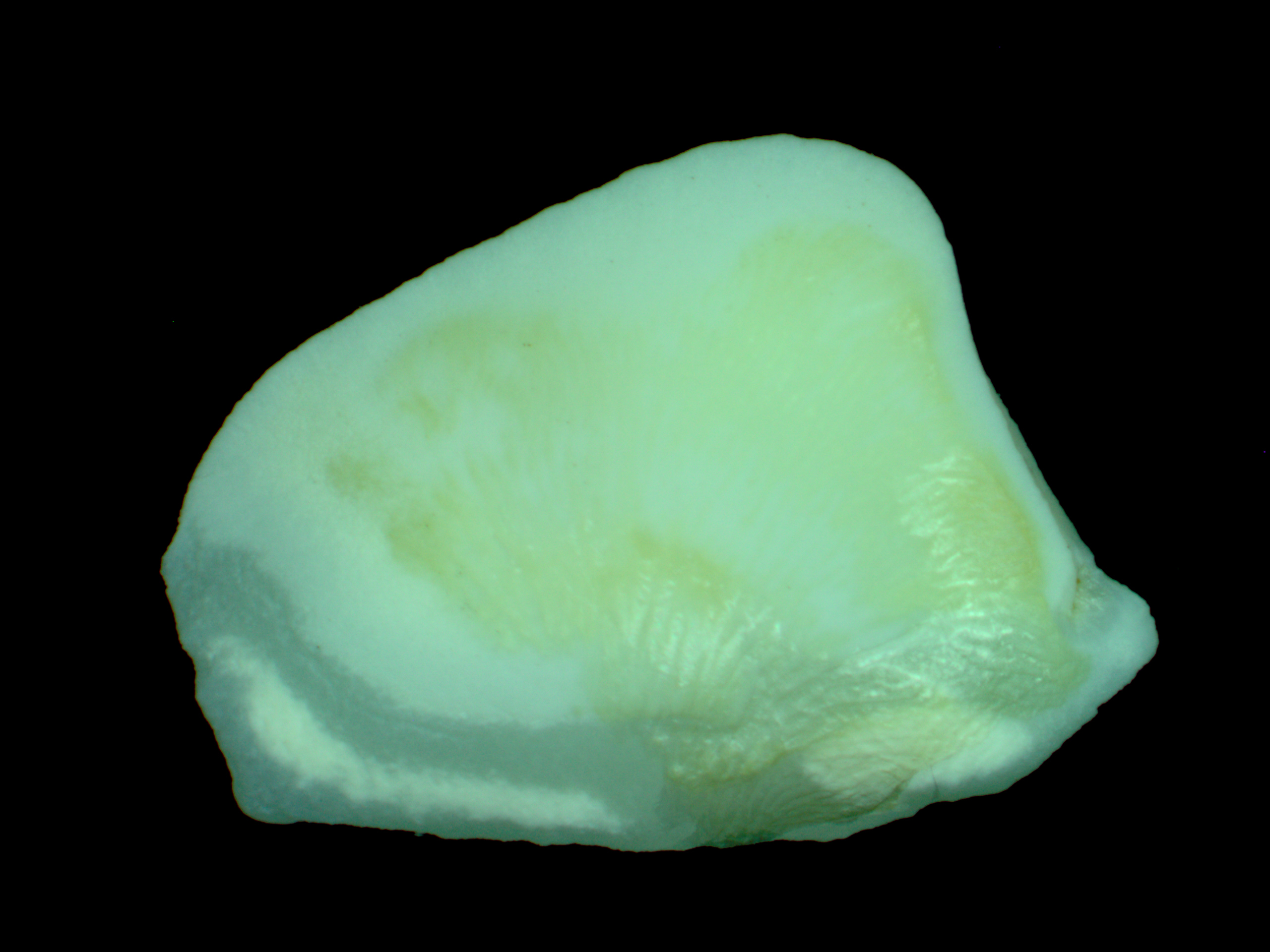

Supplement: Supplemental Information 6 [file peerj-04-1664-s006.zip › OstMil/testing/ARI1084_R1.jpg]

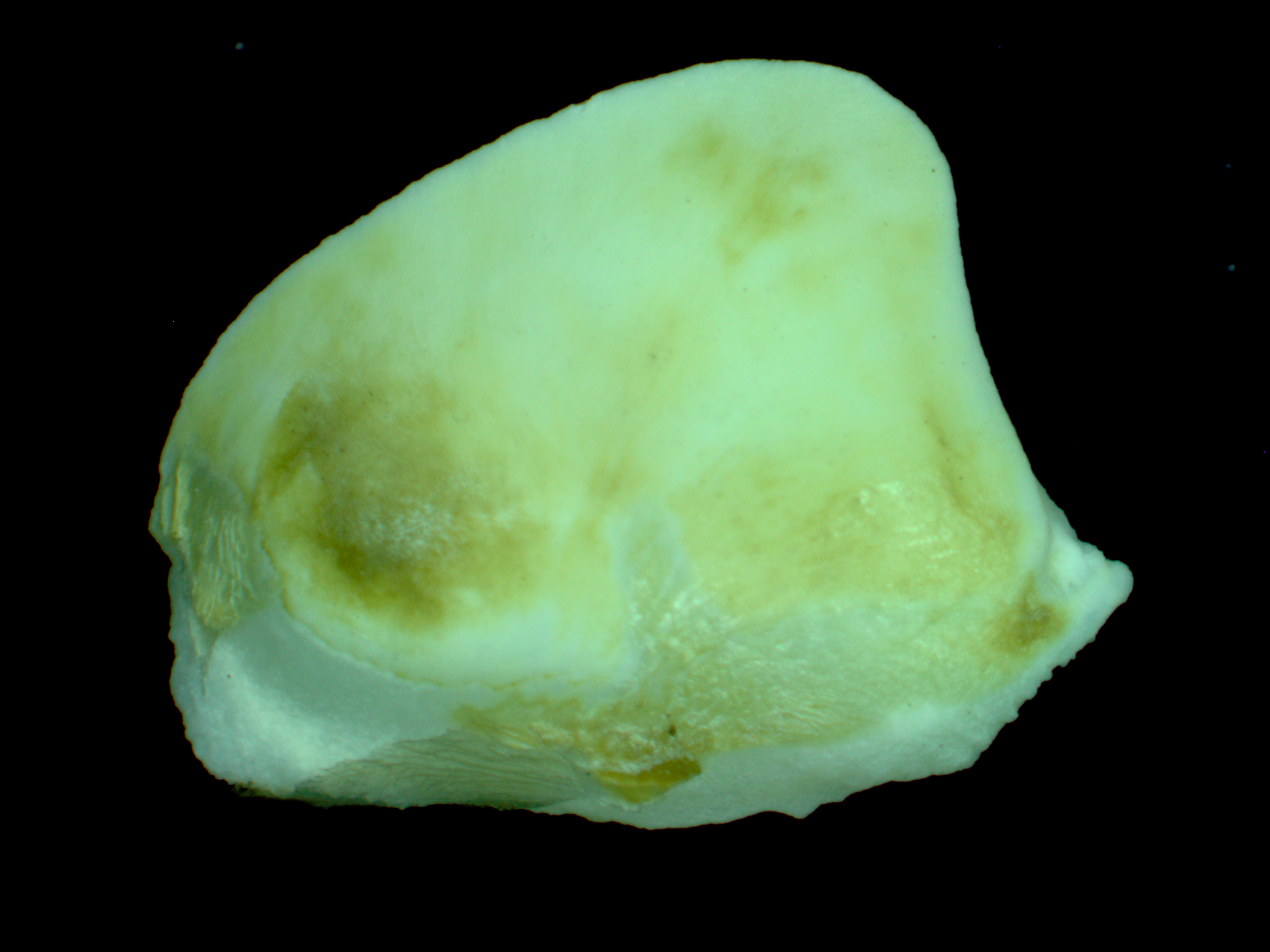

Supplement: Supplemental Information 6 [file peerj-04-1664-s006.zip › OstMil/testing/ARI1085_R1.jpg]

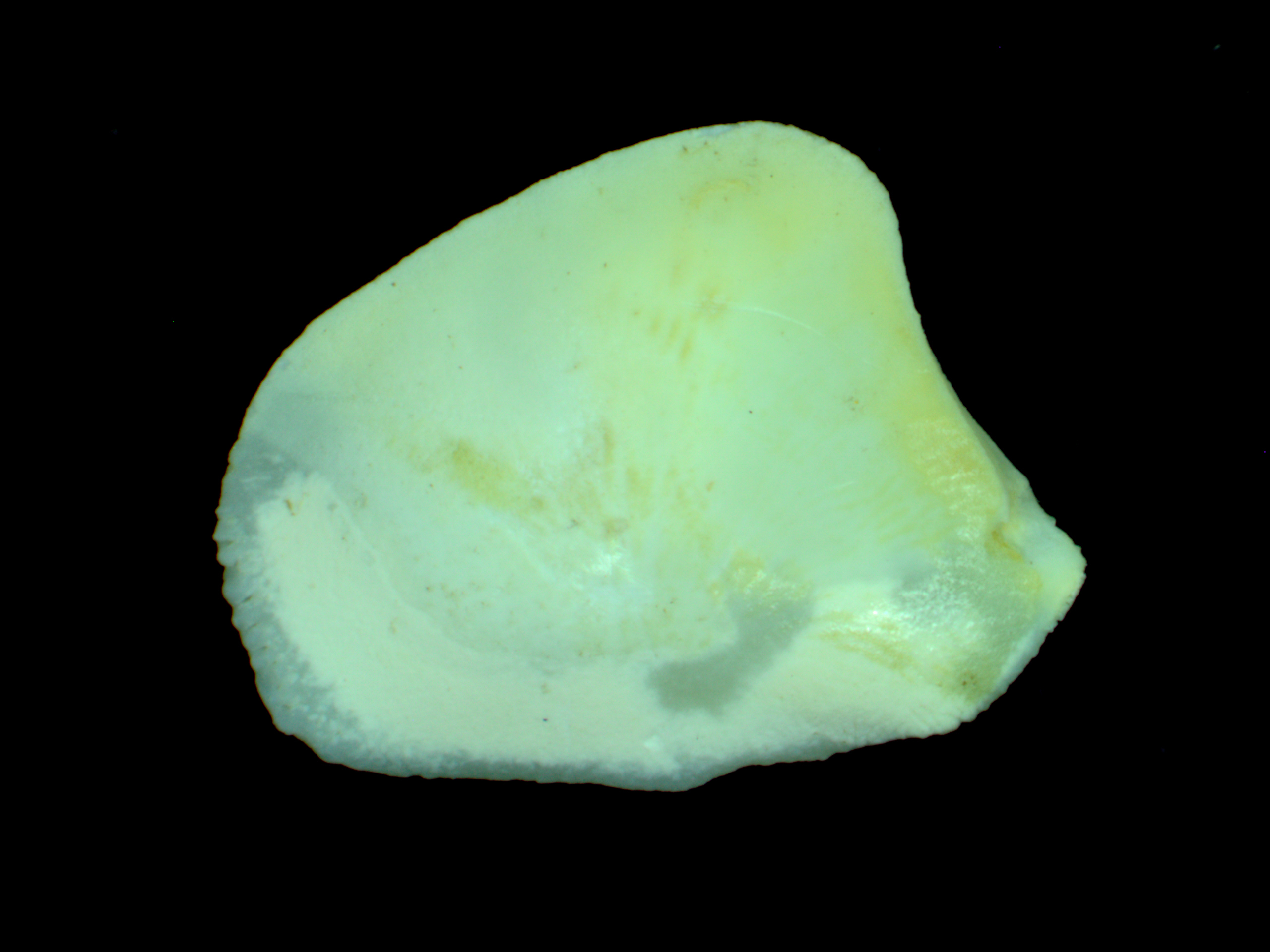

Supplement: Supplemental Information 6 [file peerj-04-1664-s006.zip › OstMil/testing/ARI977_R1.jpg]

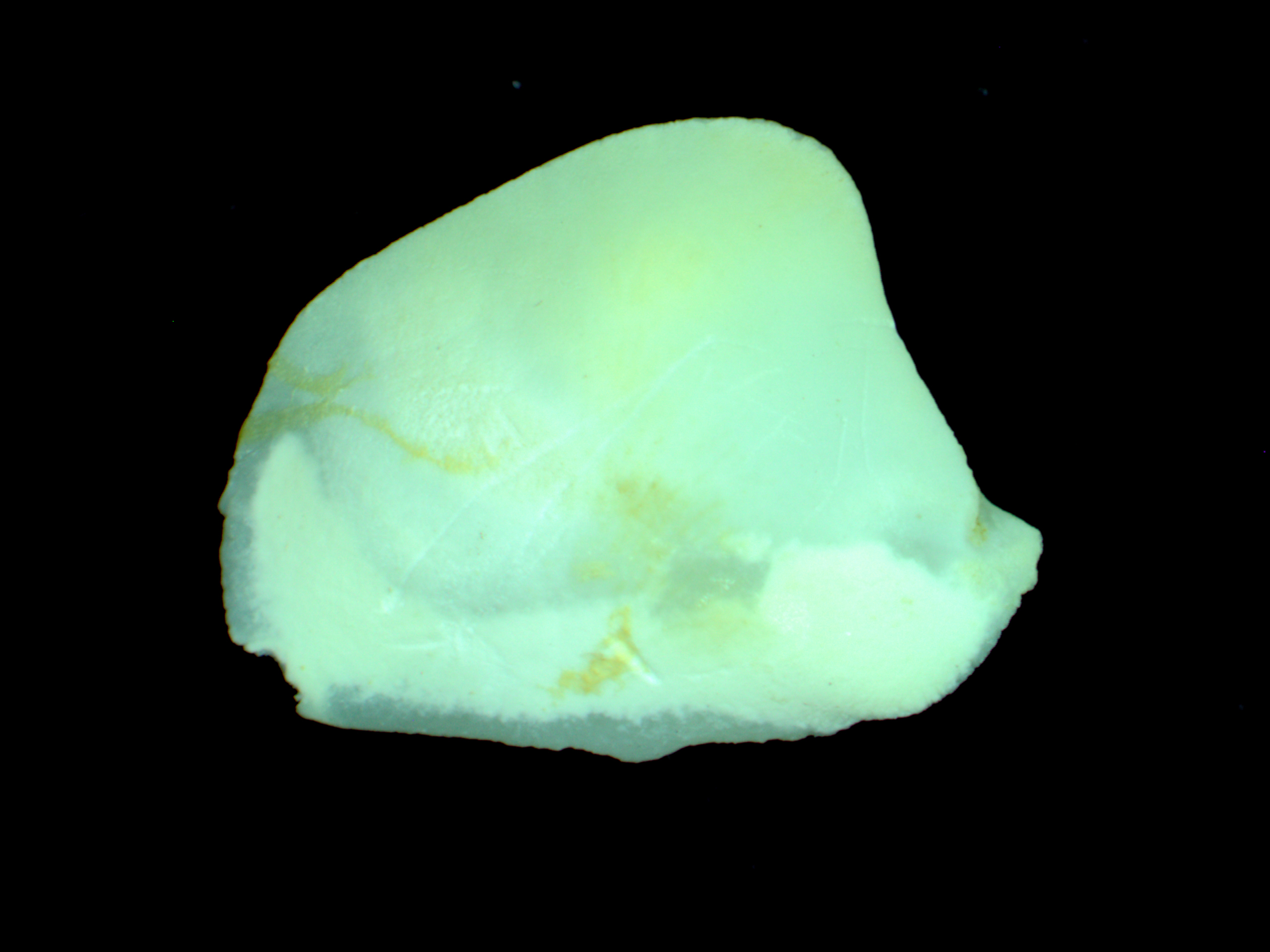

Supplement: Supplemental Information 6 [file peerj-04-1664-s006.zip › OstMil/testing/ARI979_R1.jpg]

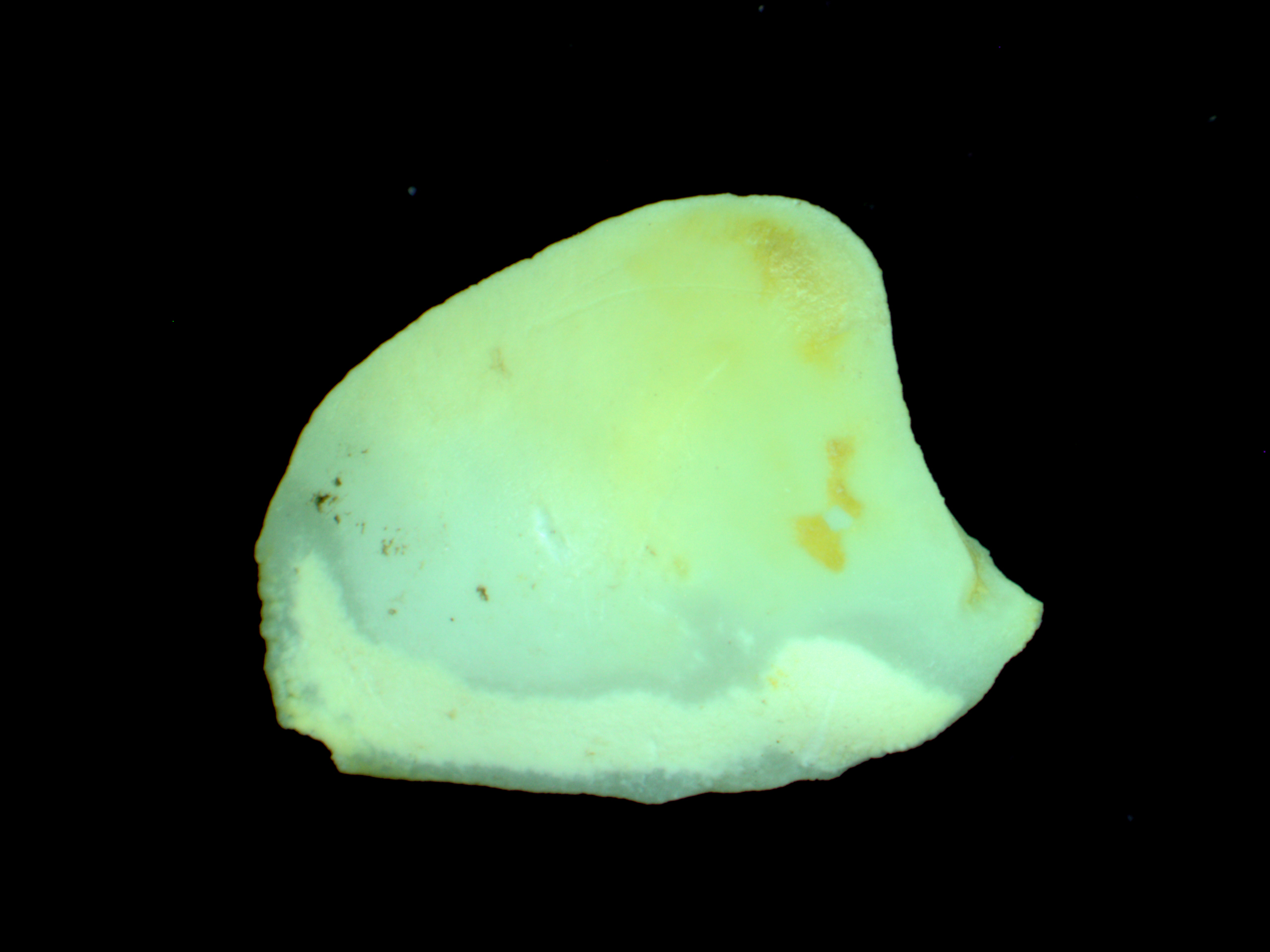

Supplement: Supplemental Information 6 [file peerj-04-1664-s006.zip › OstMil/testing/ARI980_R1.jpg]

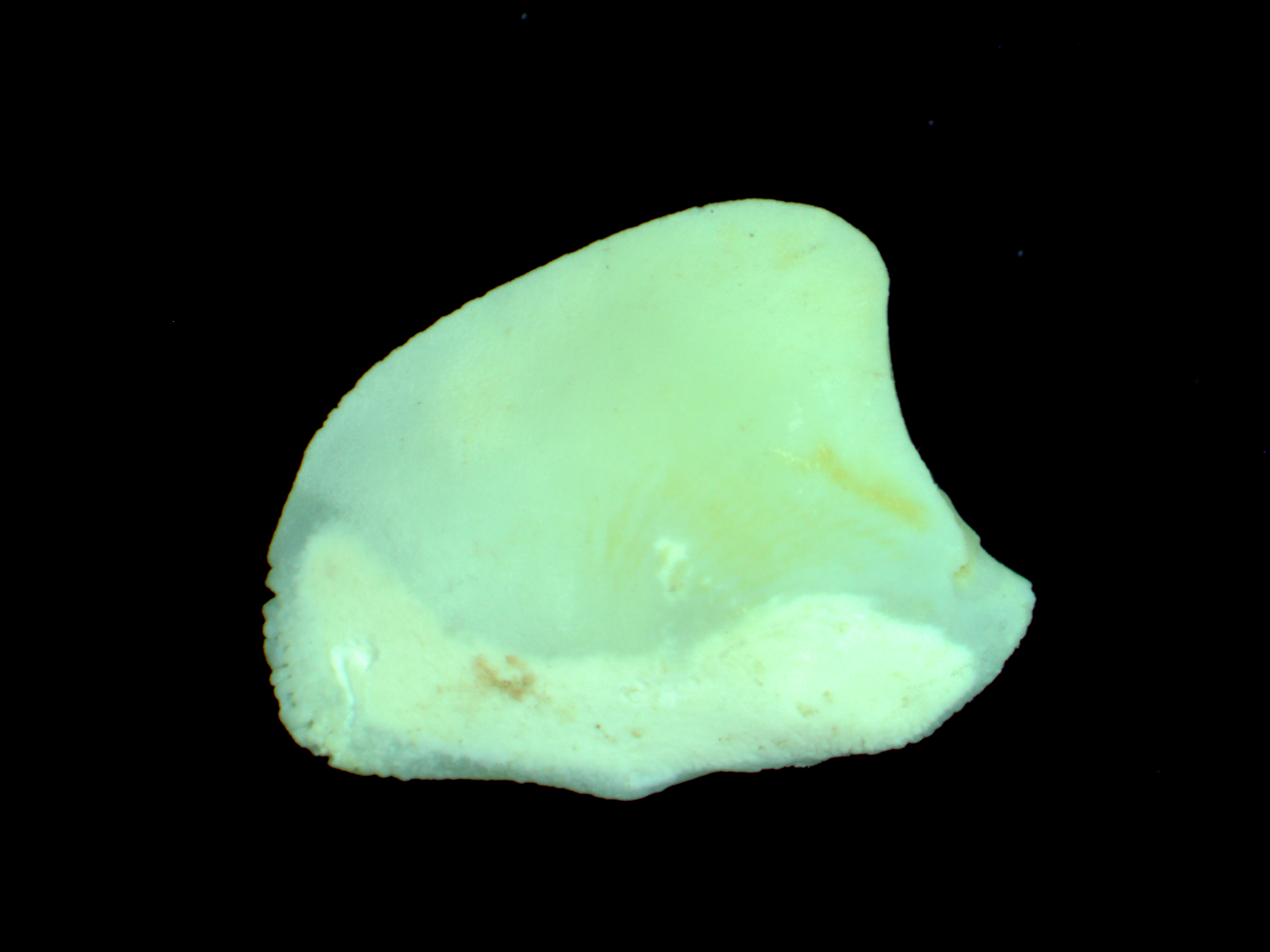

Supplement: Supplemental Information 6 [file peerj-04-1664-s006.zip › OstMil/testing/ARI981_R1.jpg]

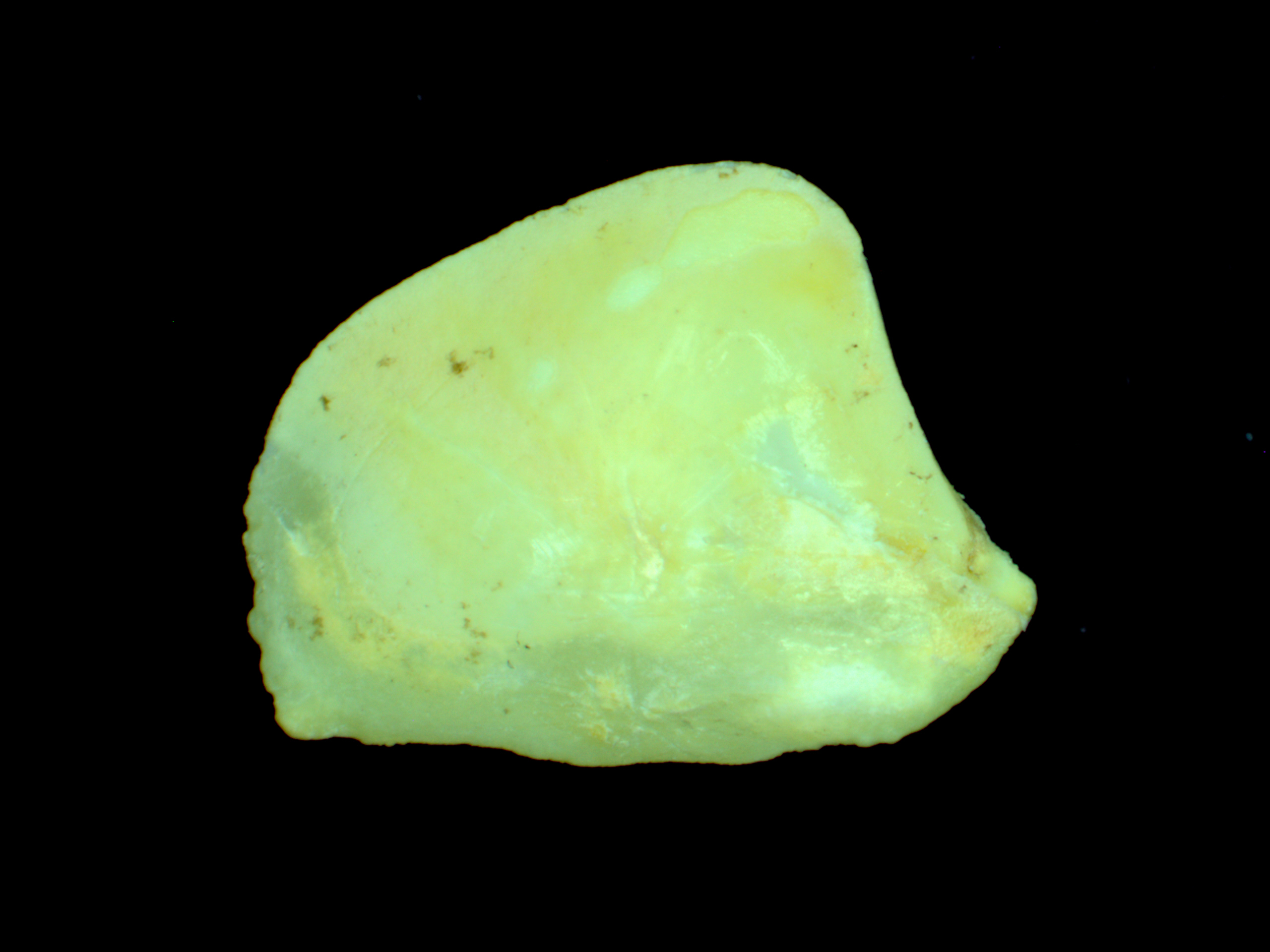

Supplement: Supplemental Information 6 [file peerj-04-1664-s006.zip › OstMil/testing/ARI983_R1.jpg]

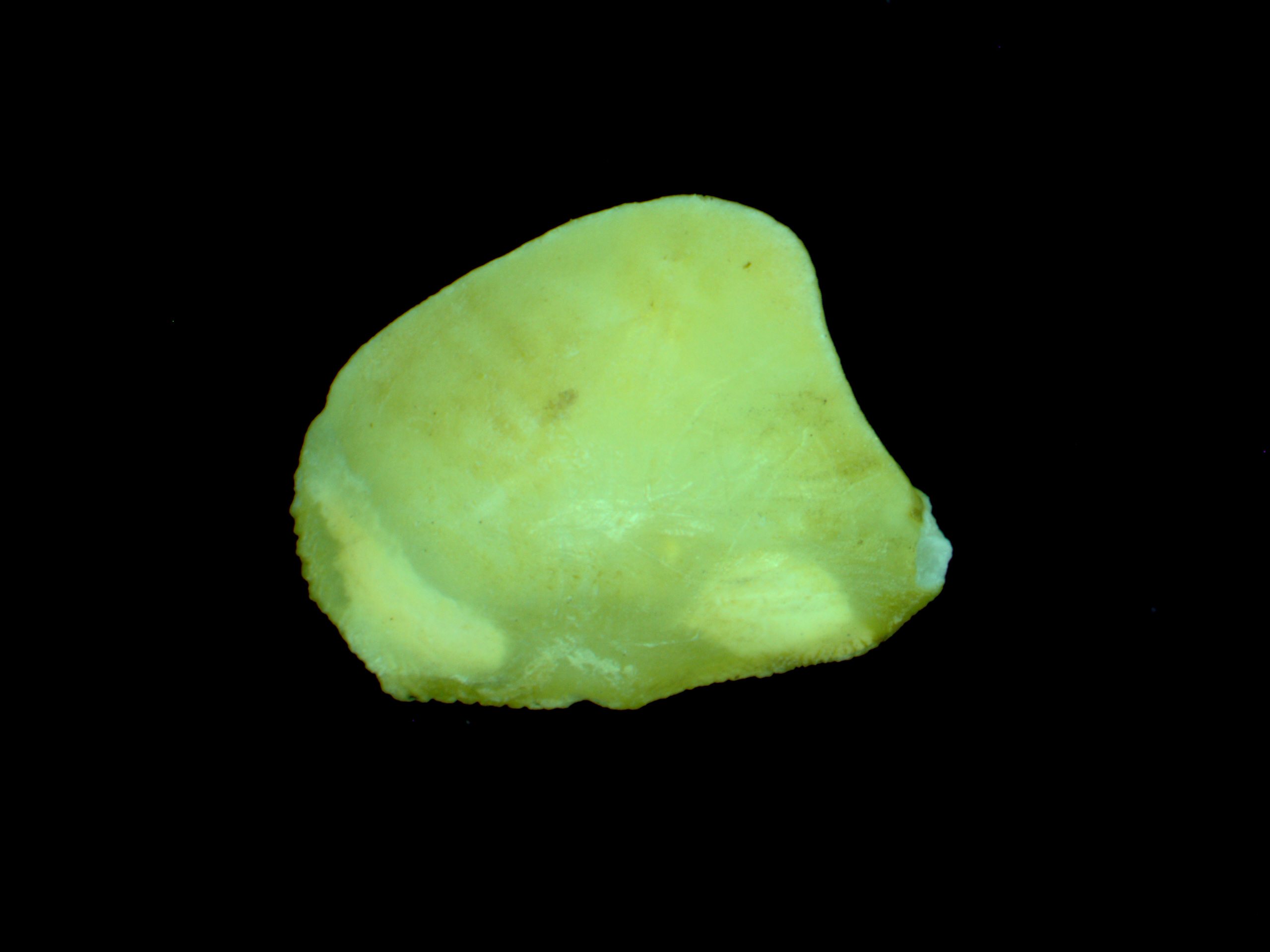

Supplement: Supplemental Information 6 [file peerj-04-1664-s006.zip › OstMil/testing/ARI984_R1.jpg]

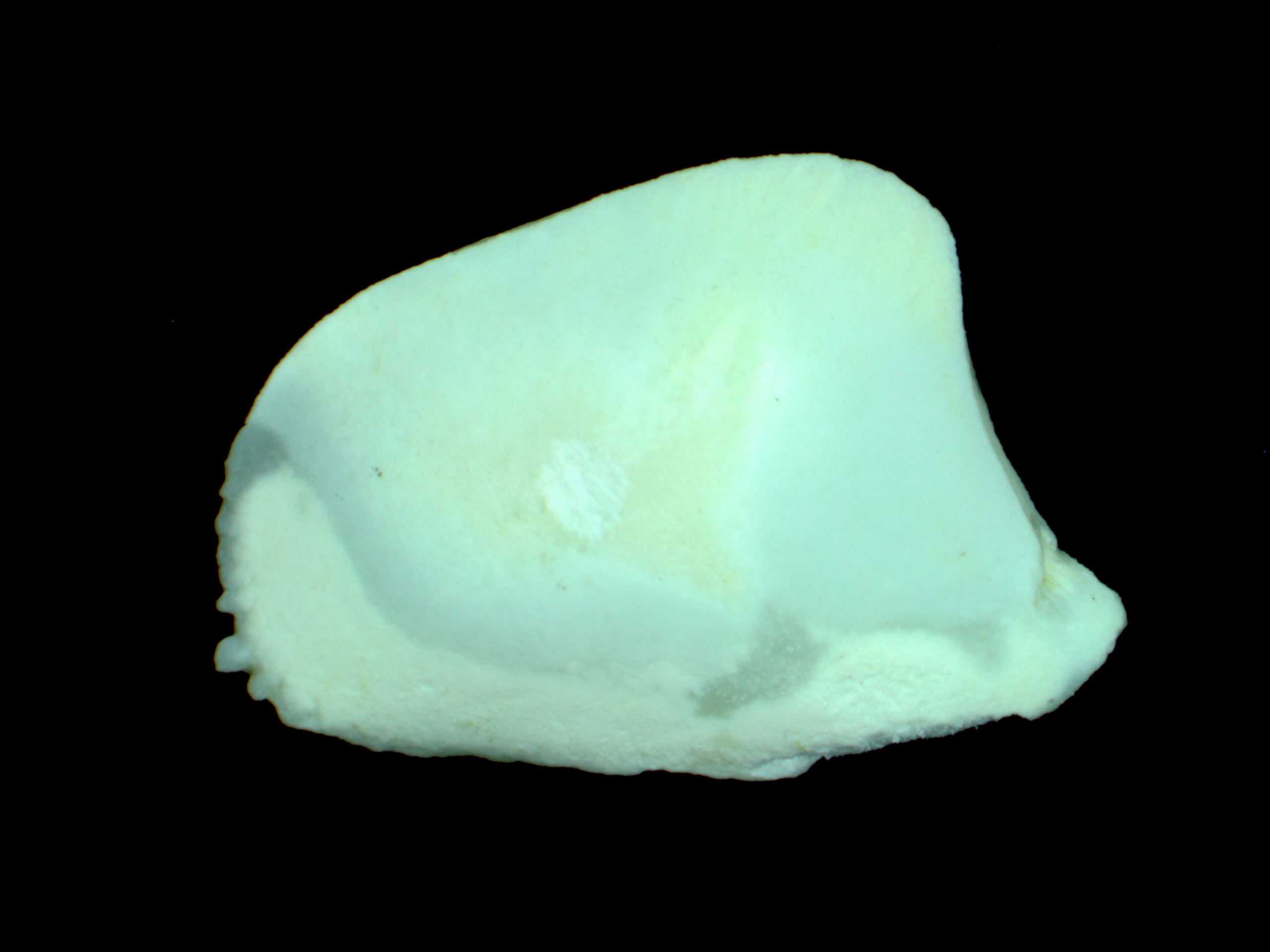

Supplement: Supplemental Information 6 [file peerj-04-1664-s006.zip › OstMil/training/ARI13_R1.jpg]

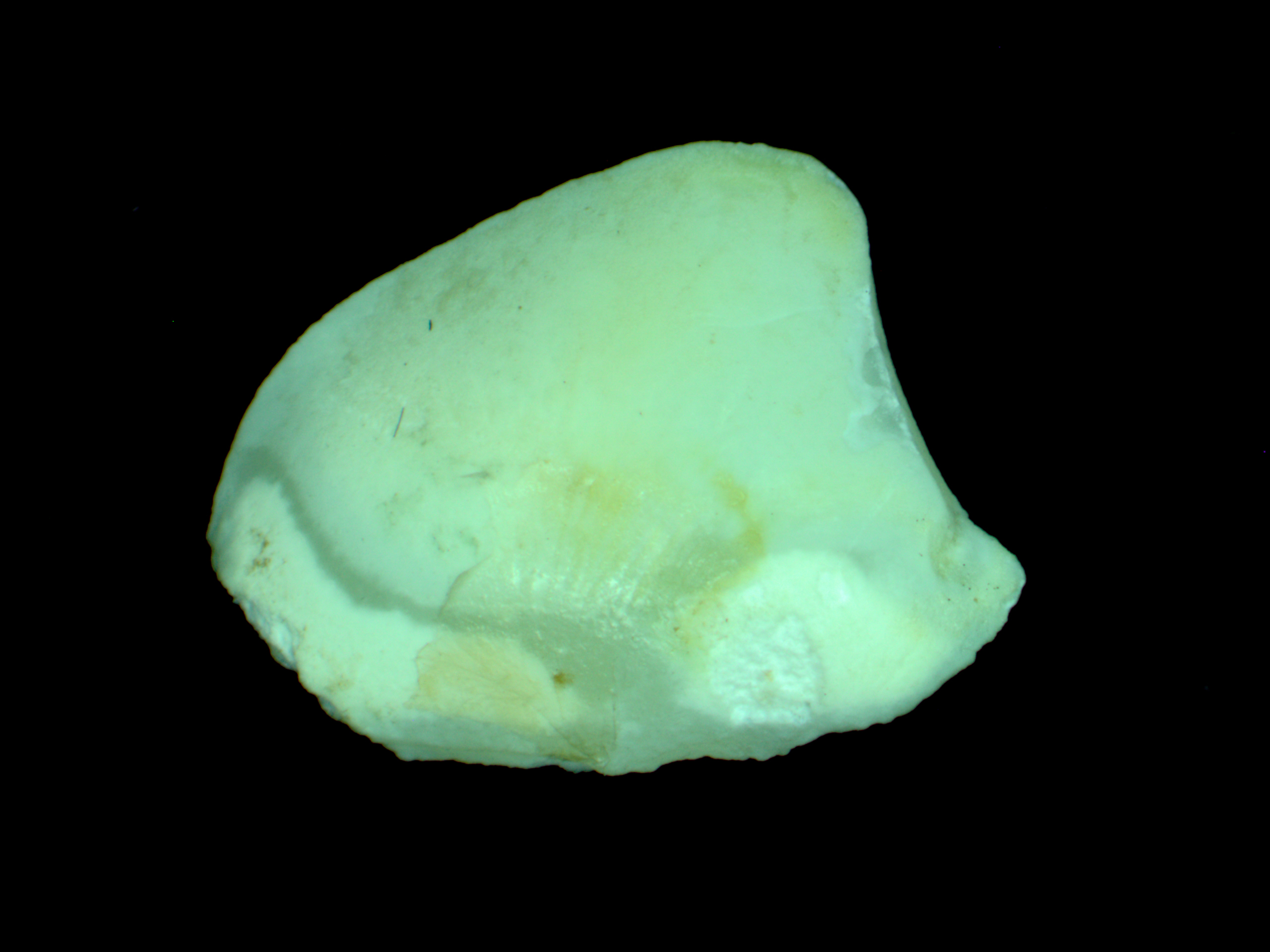

Supplement: Supplemental Information 6 [file peerj-04-1664-s006.zip › OstMil/training/ARI14_R1.jpg]

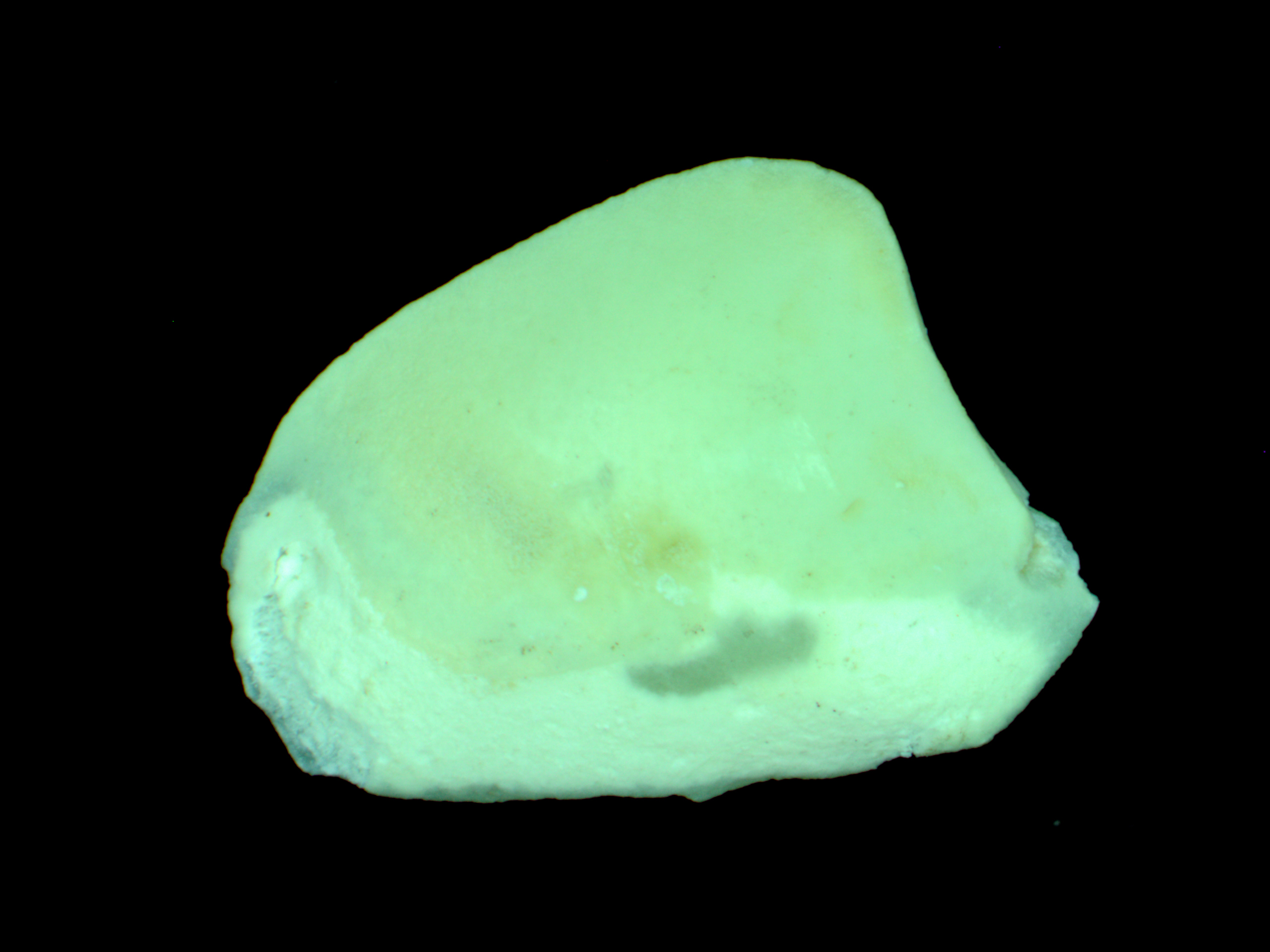

Supplement: Supplemental Information 6 [file peerj-04-1664-s006.zip › OstMil/training/ARI30_R1.jpg]

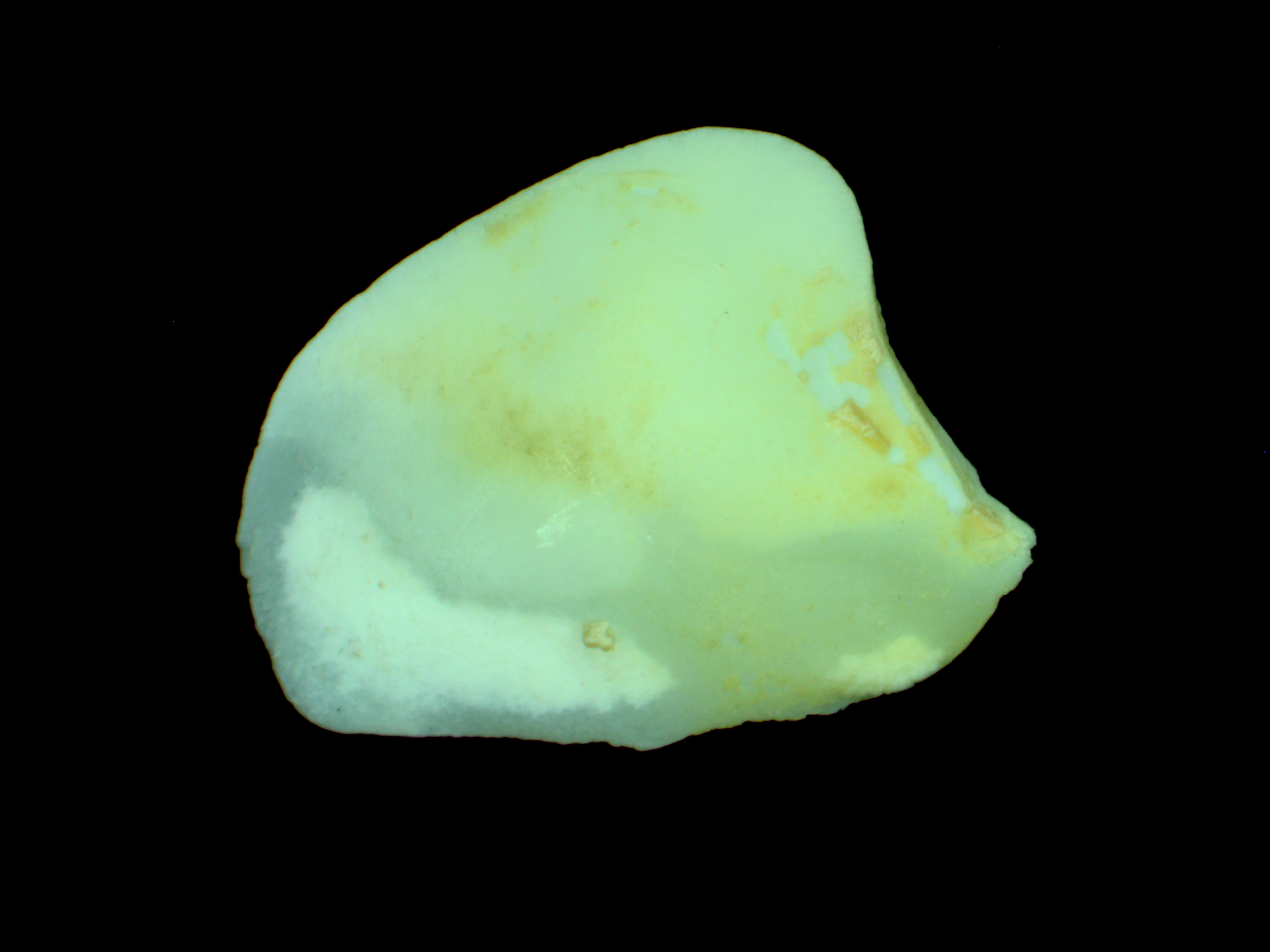

Supplement: Supplemental Information 6 [file peerj-04-1664-s006.zip › OstMil/training/ARI32_R1.jpg]

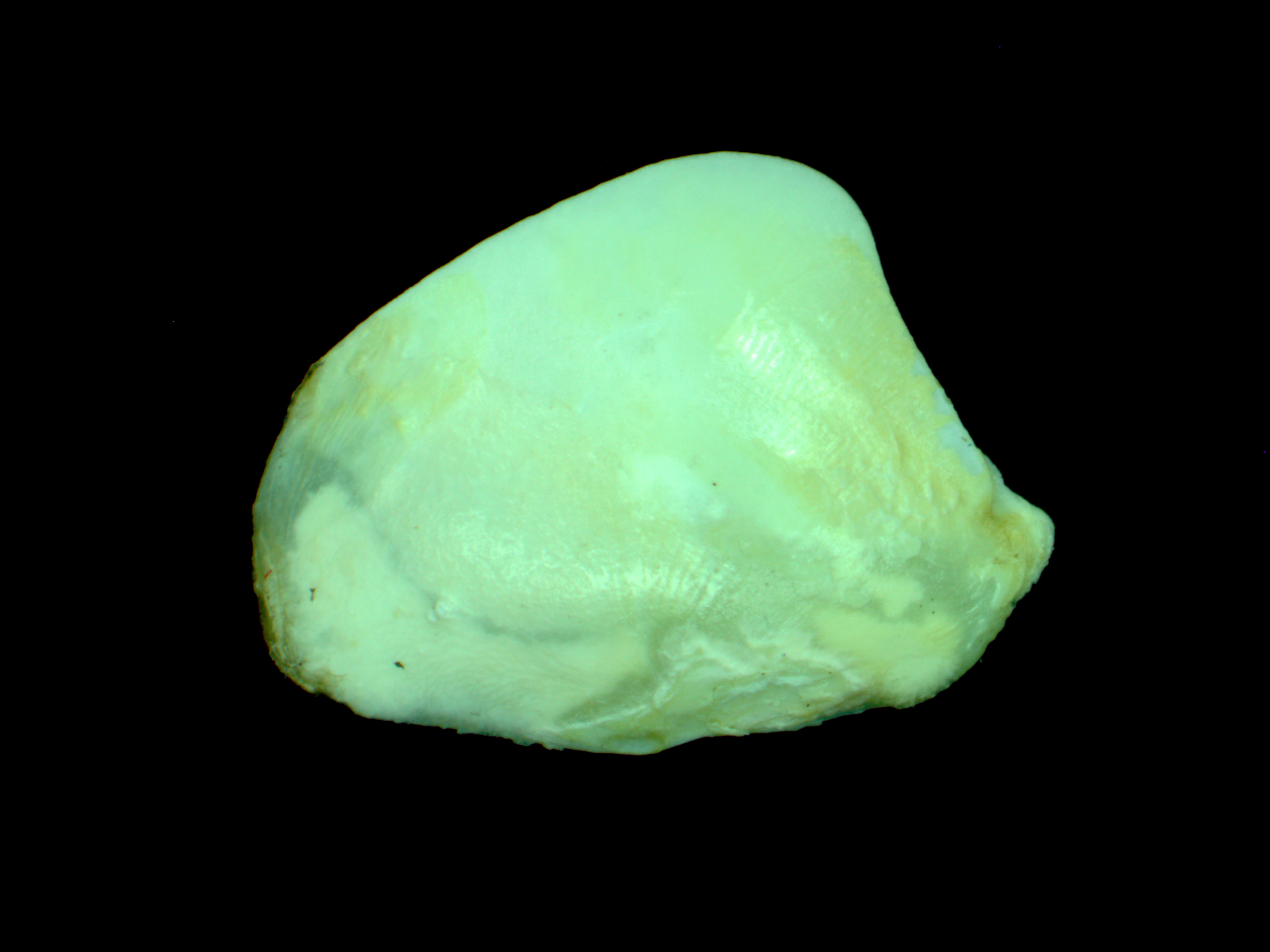

Supplement: Supplemental Information 6 [file peerj-04-1664-s006.zip › OstMil/training/ARI33_R1.jpg]

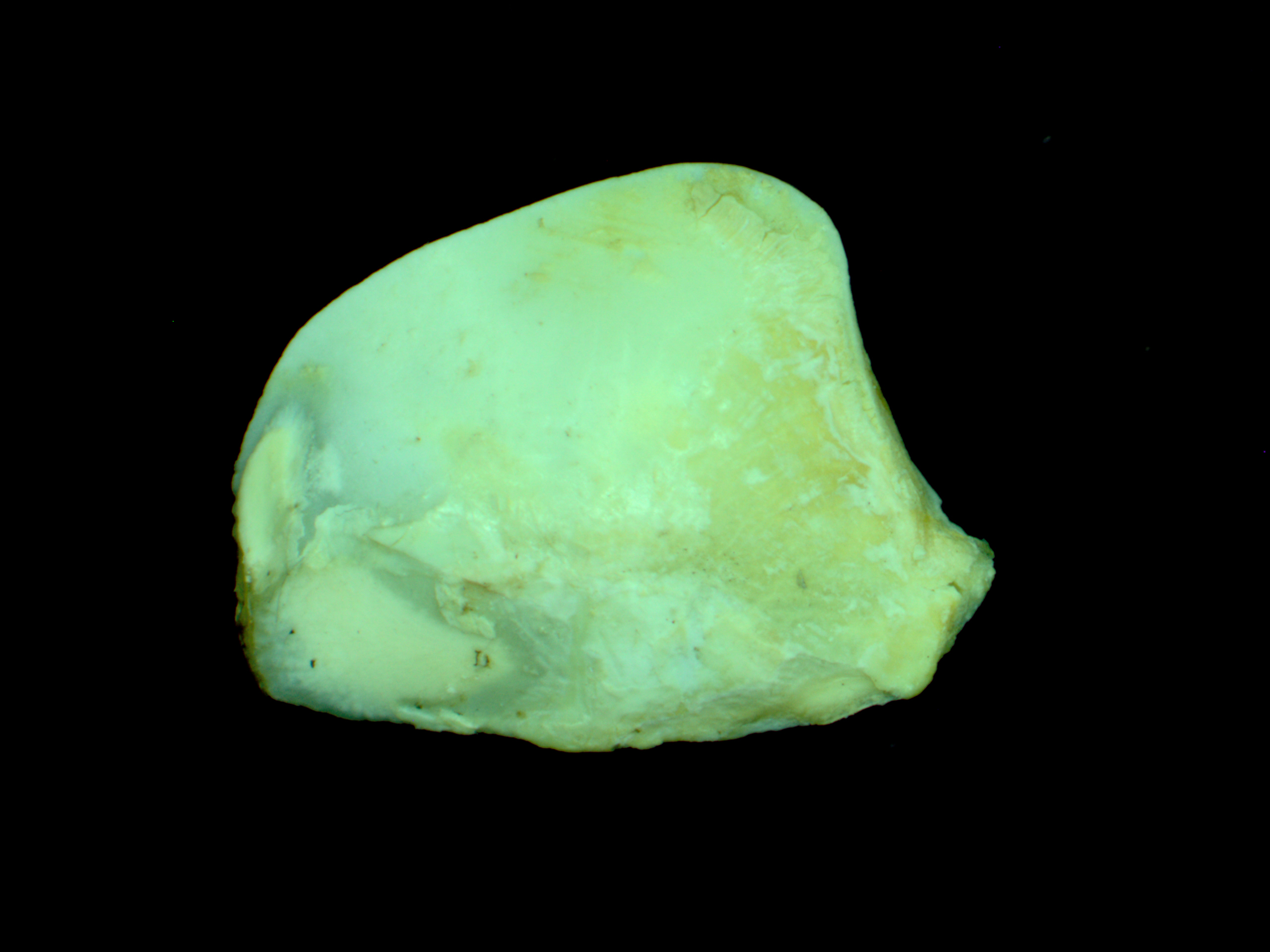

Supplement: Supplemental Information 6 [file peerj-04-1664-s006.zip › OstMil/training/ARI34_R1.jpg]

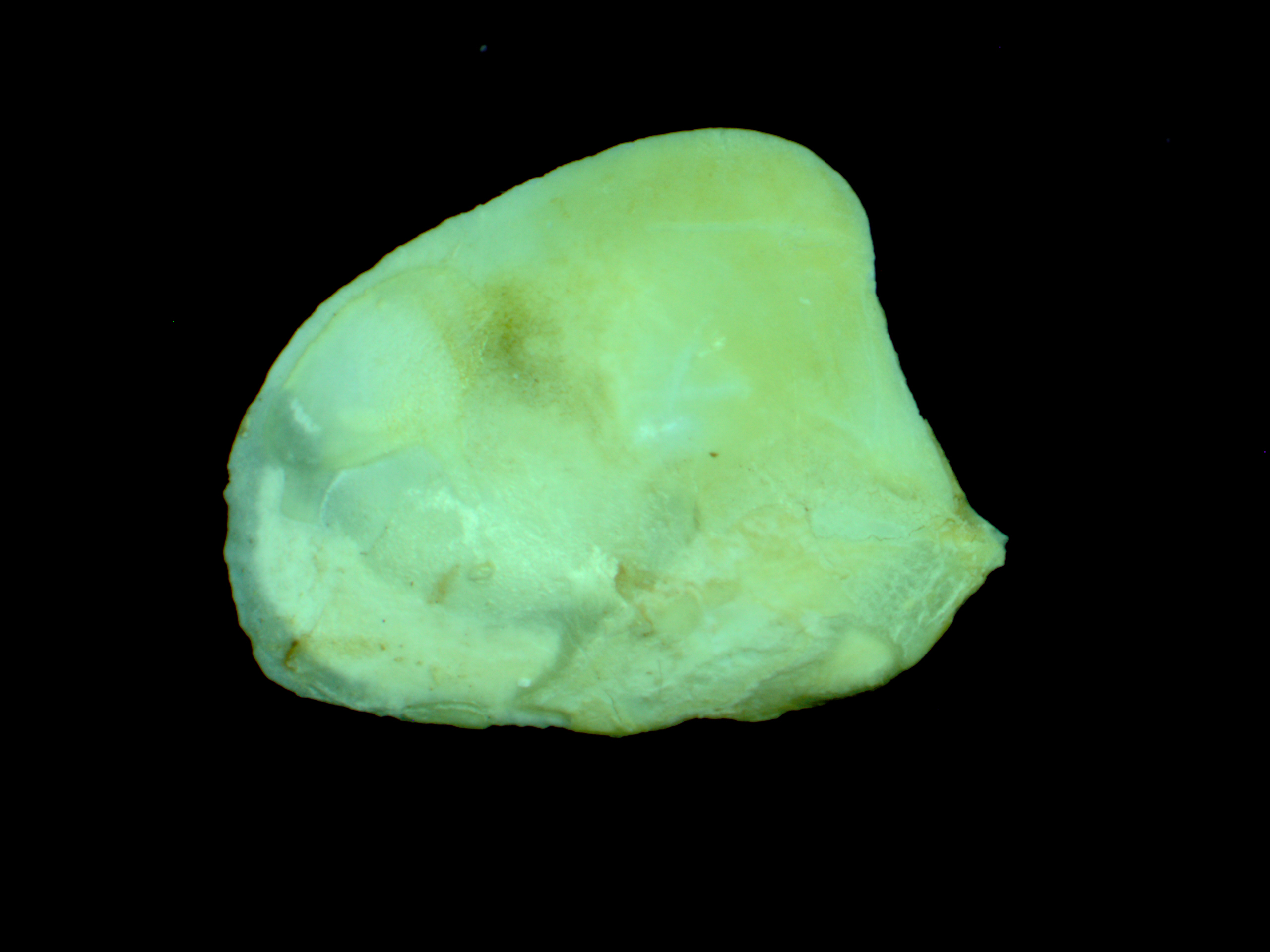

Supplement: Supplemental Information 6 [file peerj-04-1664-s006.zip › OstMil/training/ARI35_R1.jpg]

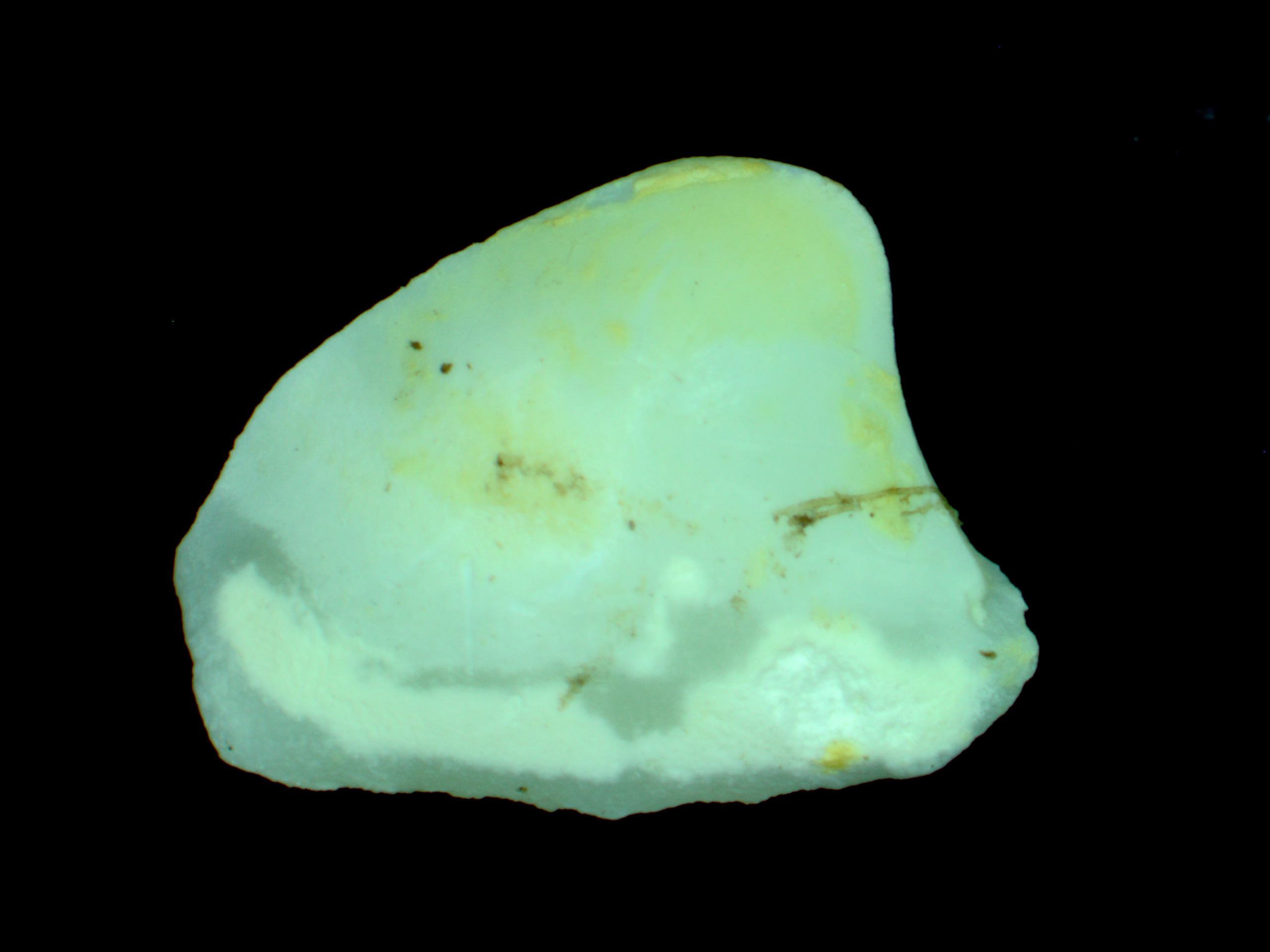

Supplement: Supplemental Information 6 [file peerj-04-1664-s006.zip › OstMil/training/ARI567_R1.jpg]

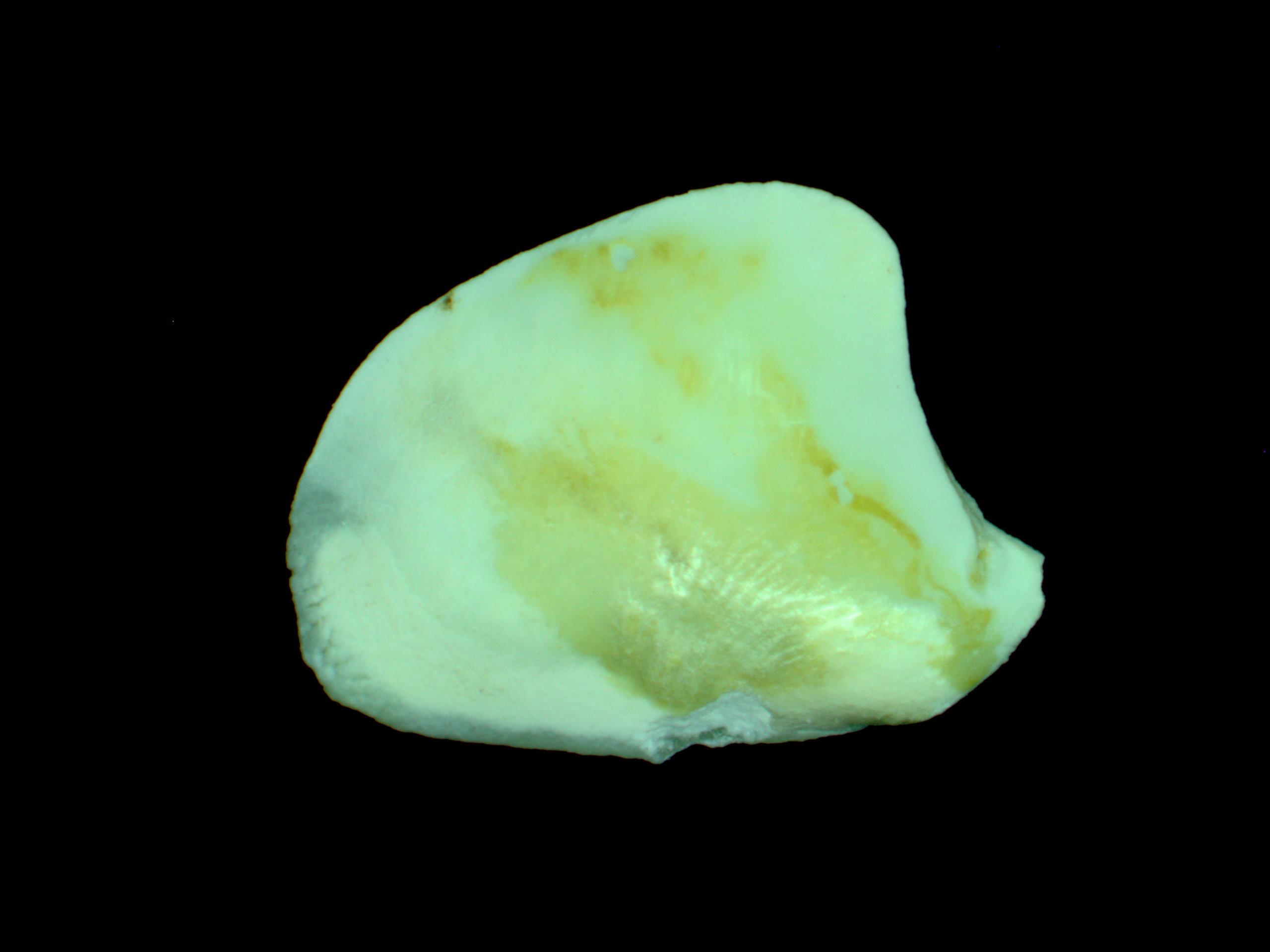

Supplement: Supplemental Information 6 [file peerj-04-1664-s006.zip › OstMil/training/ARI570_R1.jpg]

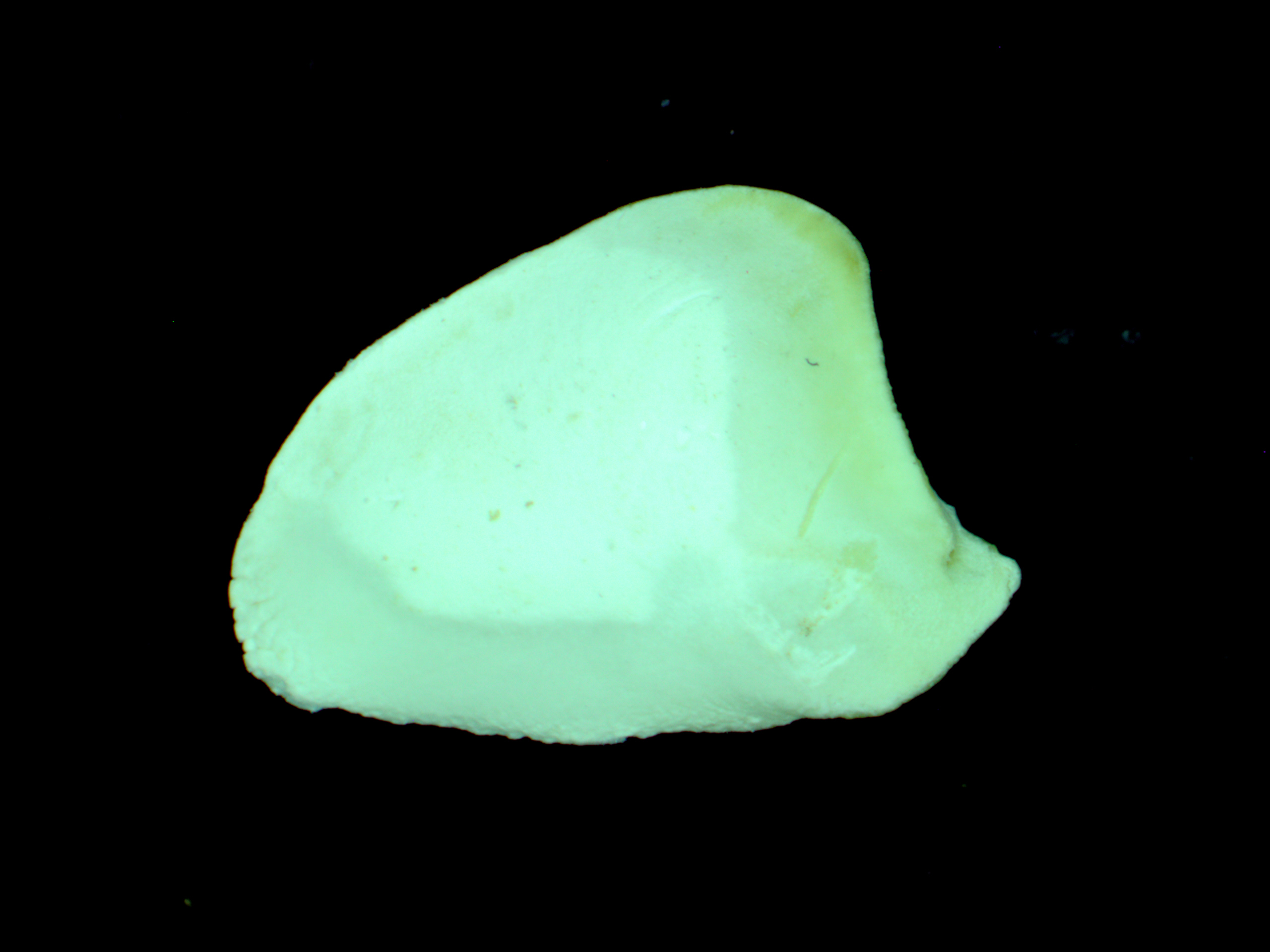

Supplement: Supplemental Information 6 [file peerj-04-1664-s006.zip › OstMil/training/ARI591_R1.jpg]

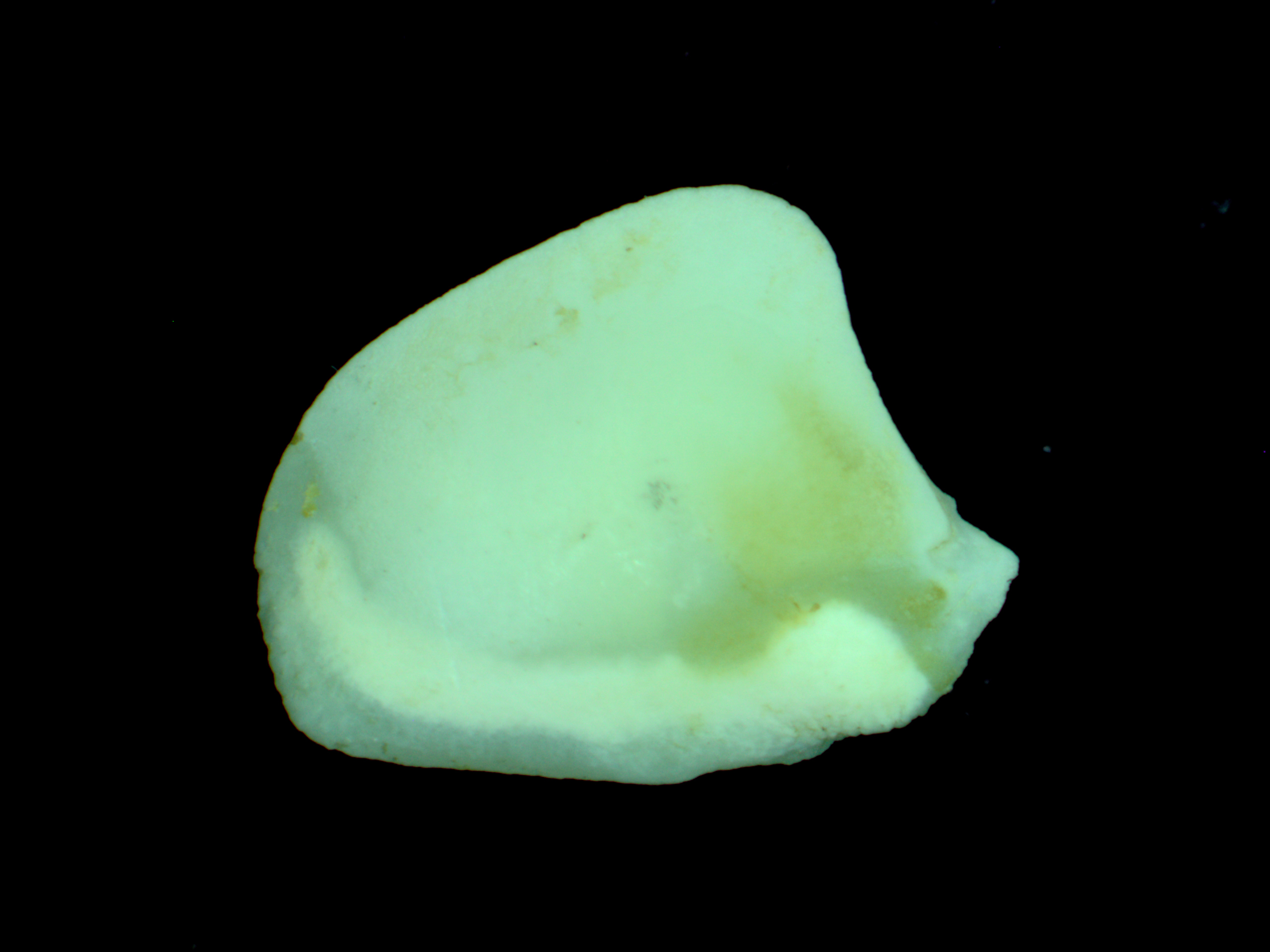

Supplement: Supplemental Information 6 [file peerj-04-1664-s006.zip › OstMil/training/ARI627_R1.jpg]

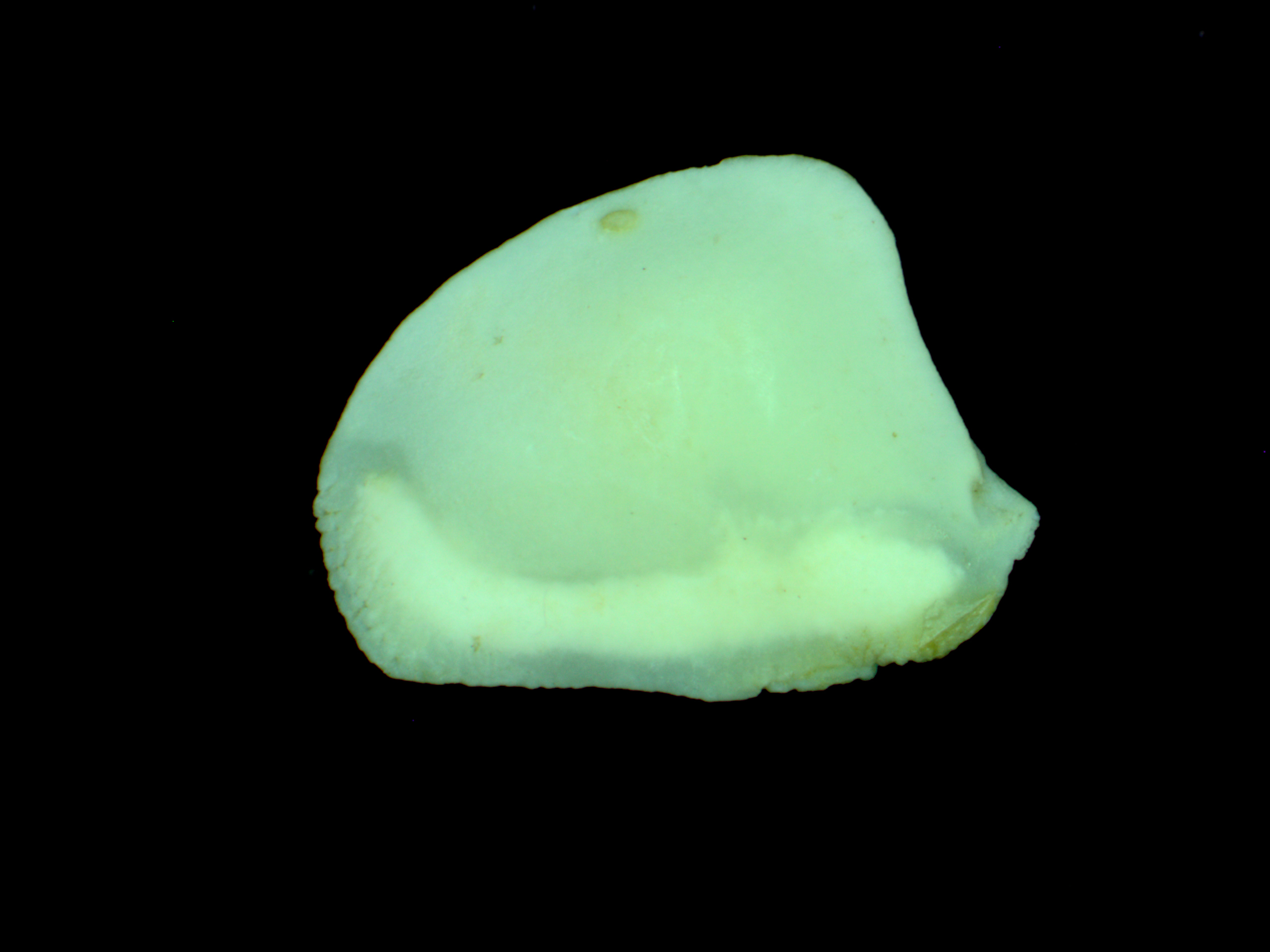

Supplement: Supplemental Information 6 [file peerj-04-1664-s006.zip › OstMil/training/ARI628_R1.jpg]

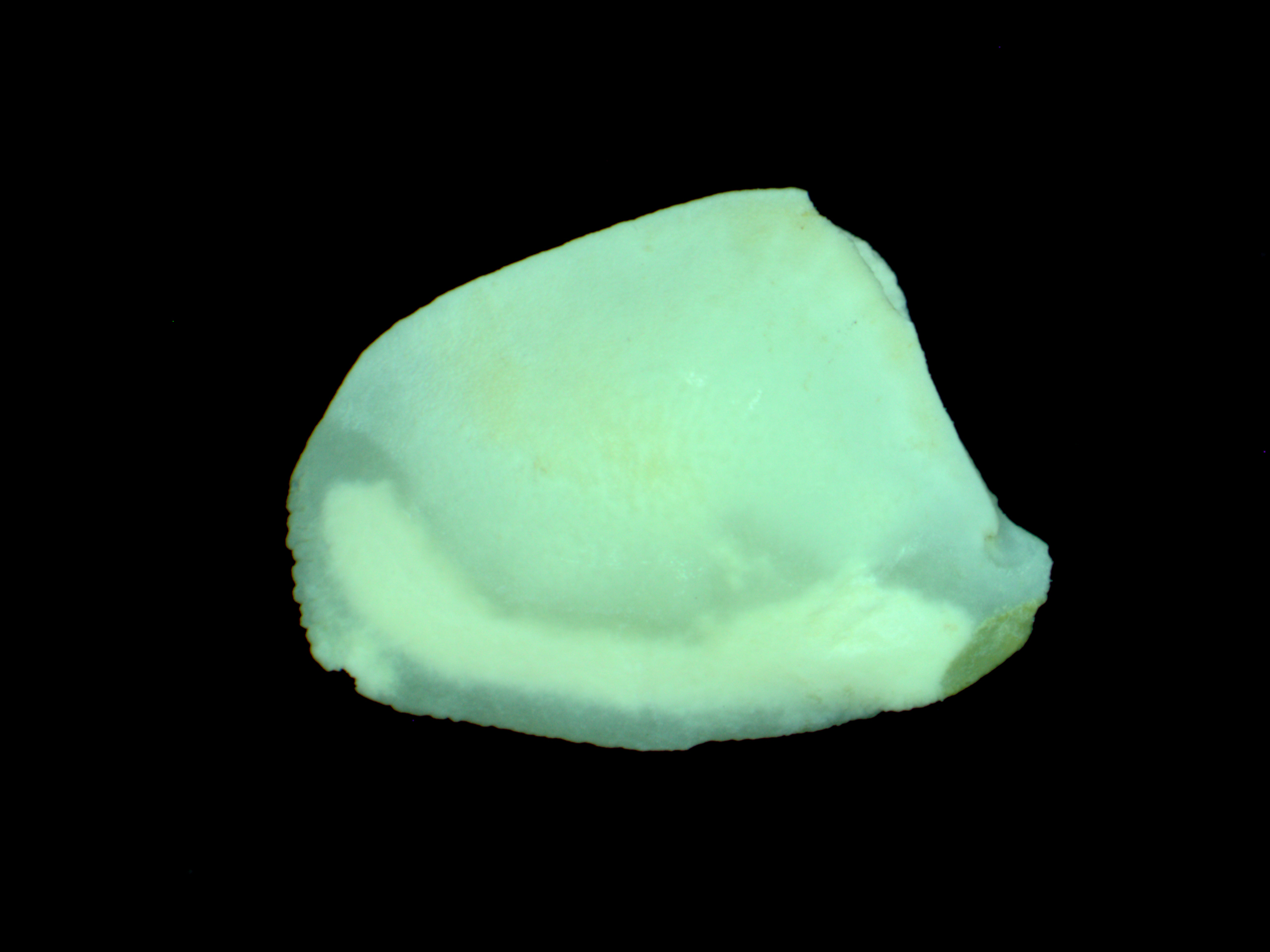

Supplement: Supplemental Information 6 [file peerj-04-1664-s006.zip › OstMil/training/ARI631_R1.jpg]

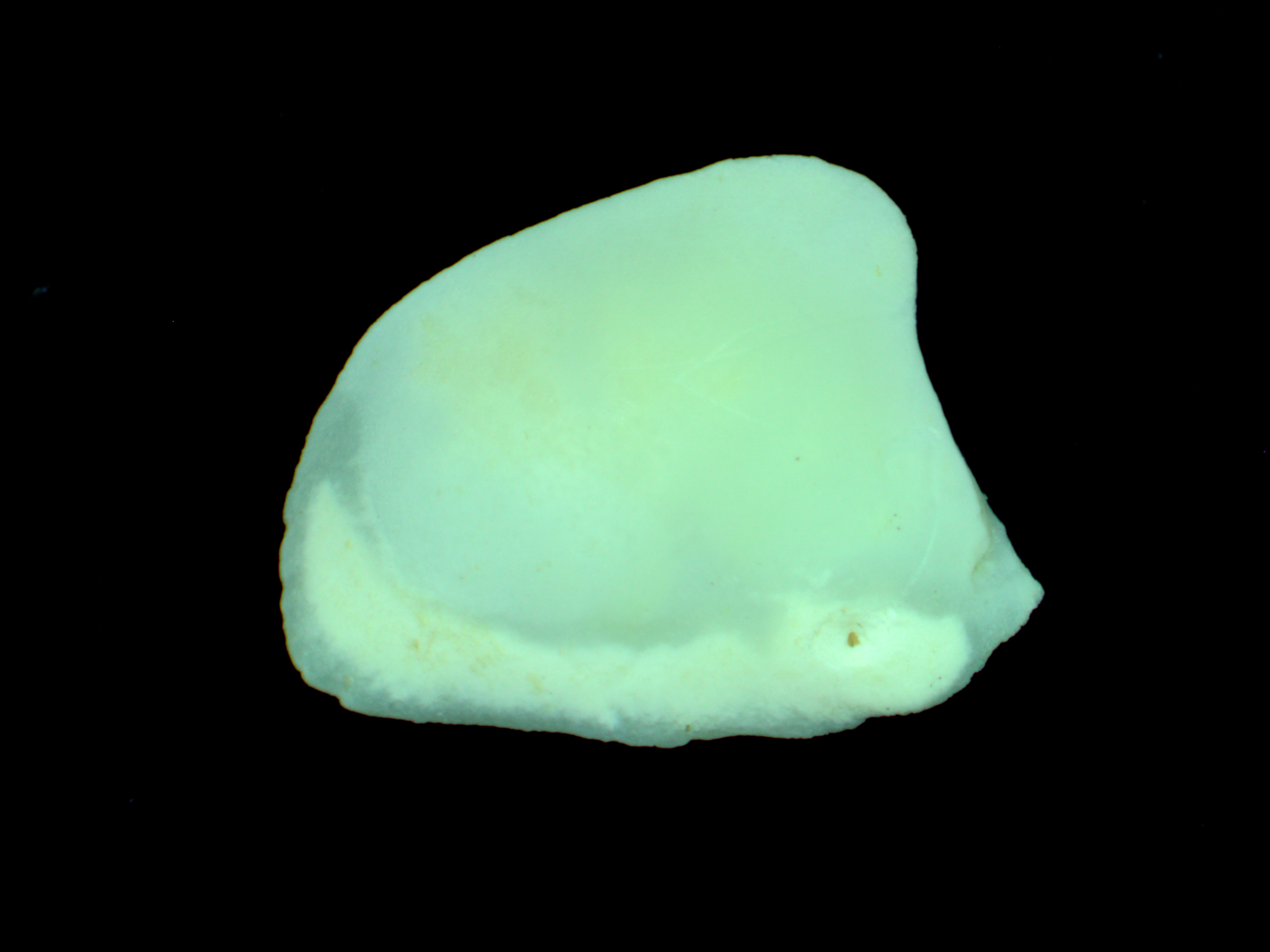

Supplement: Supplemental Information 6 [file peerj-04-1664-s006.zip › OstMil/training/ARI636_R1.jpg]

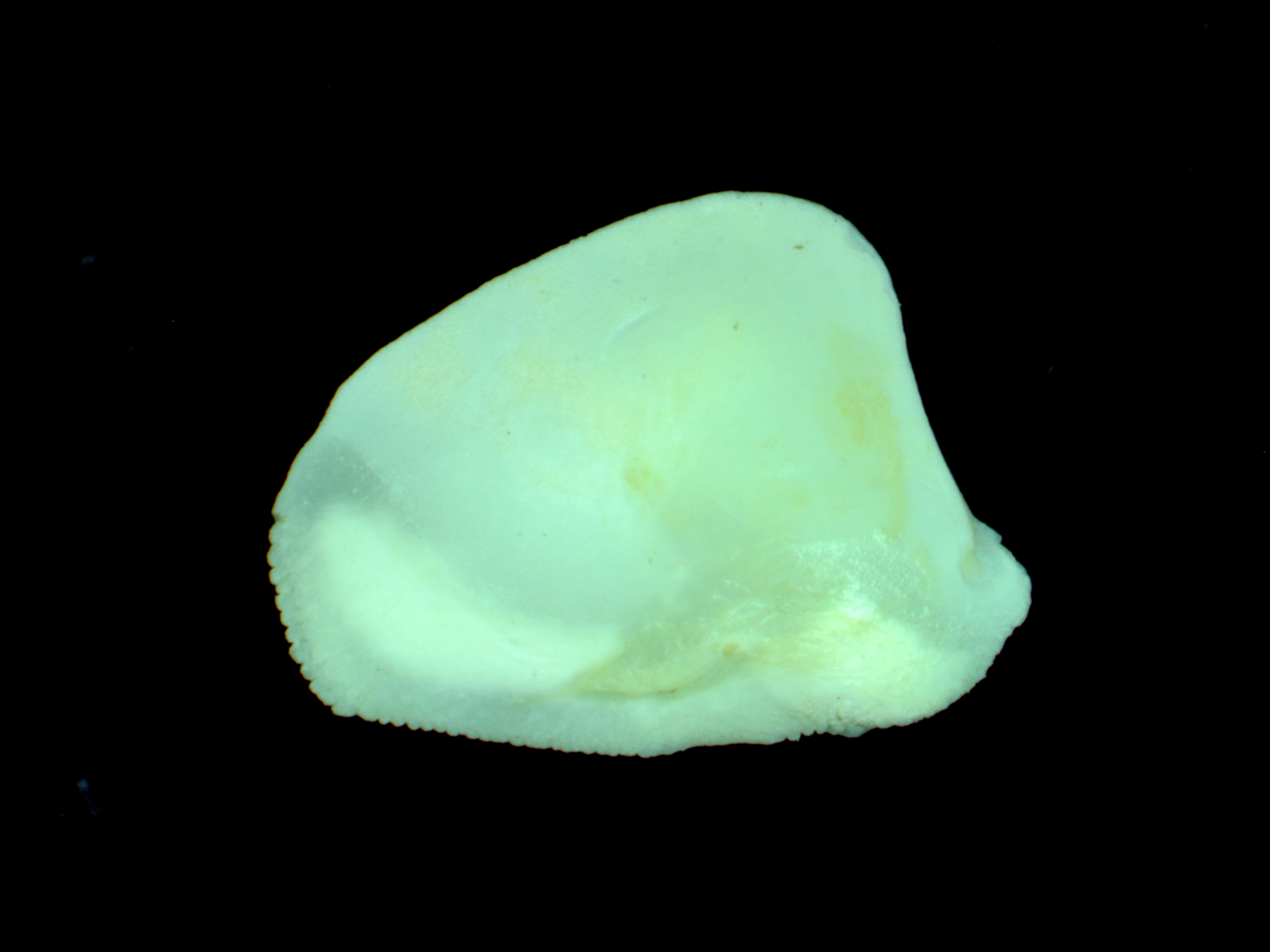

Supplement: Supplemental Information 6 [file peerj-04-1664-s006.zip › OstMil/training/ARI637_R1.jpg]

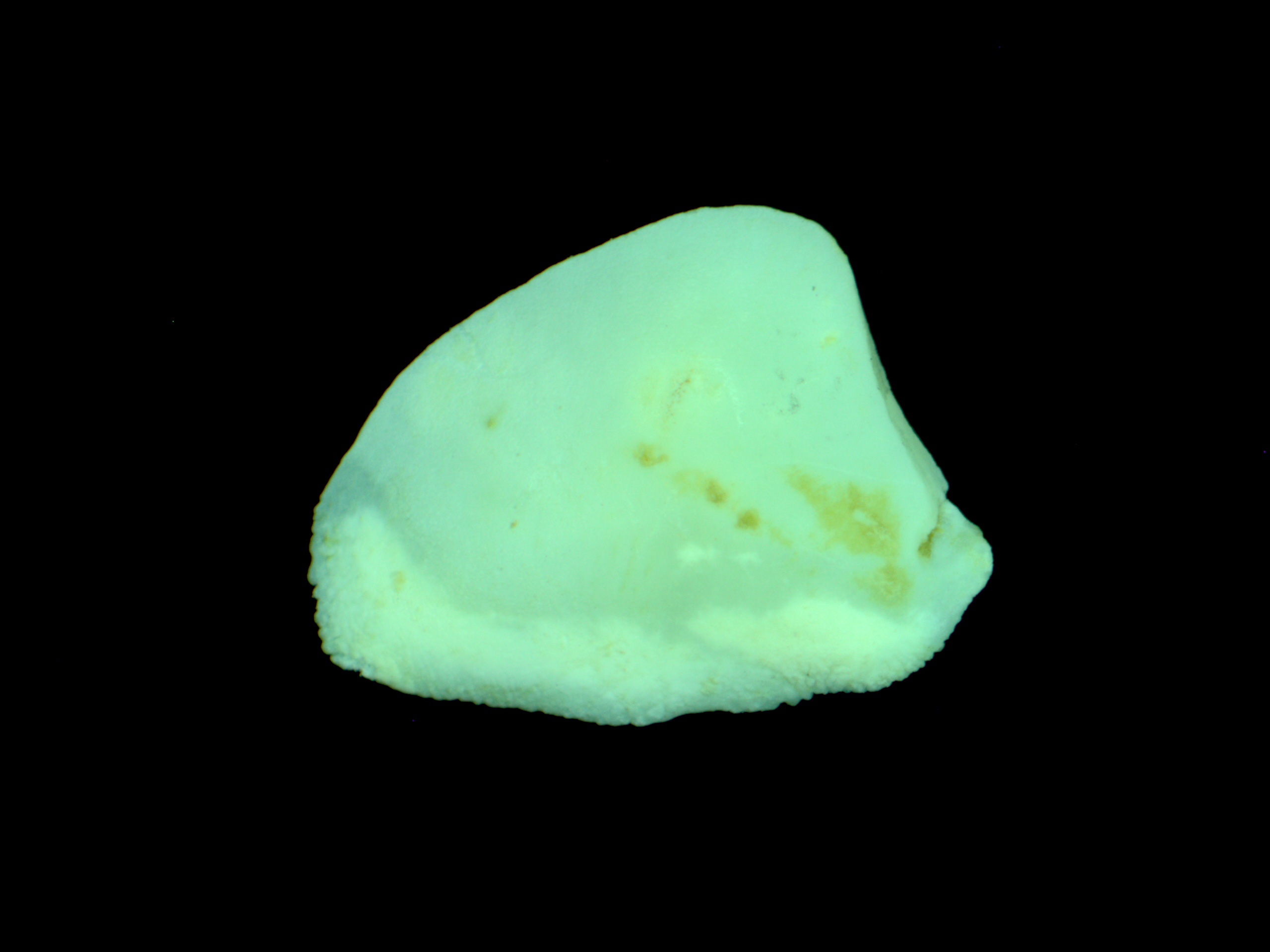

Supplement: Supplemental Information 6 [file peerj-04-1664-s006.zip › OstMil/training/ARI756_R1.jpg]

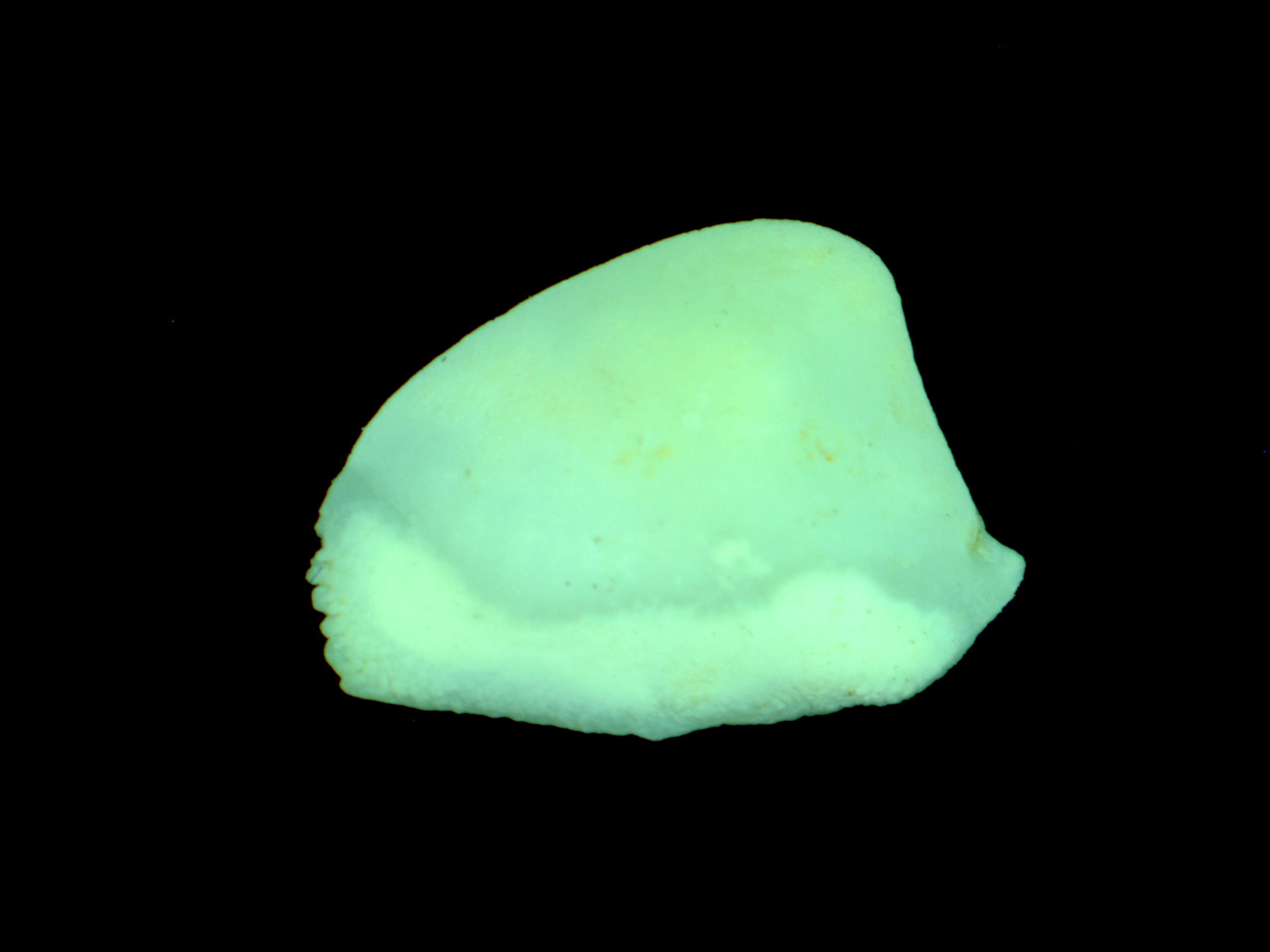

Supplement: Supplemental Information 6 [file peerj-04-1664-s006.zip › OstMil/training/ARI758_R1.jpg]

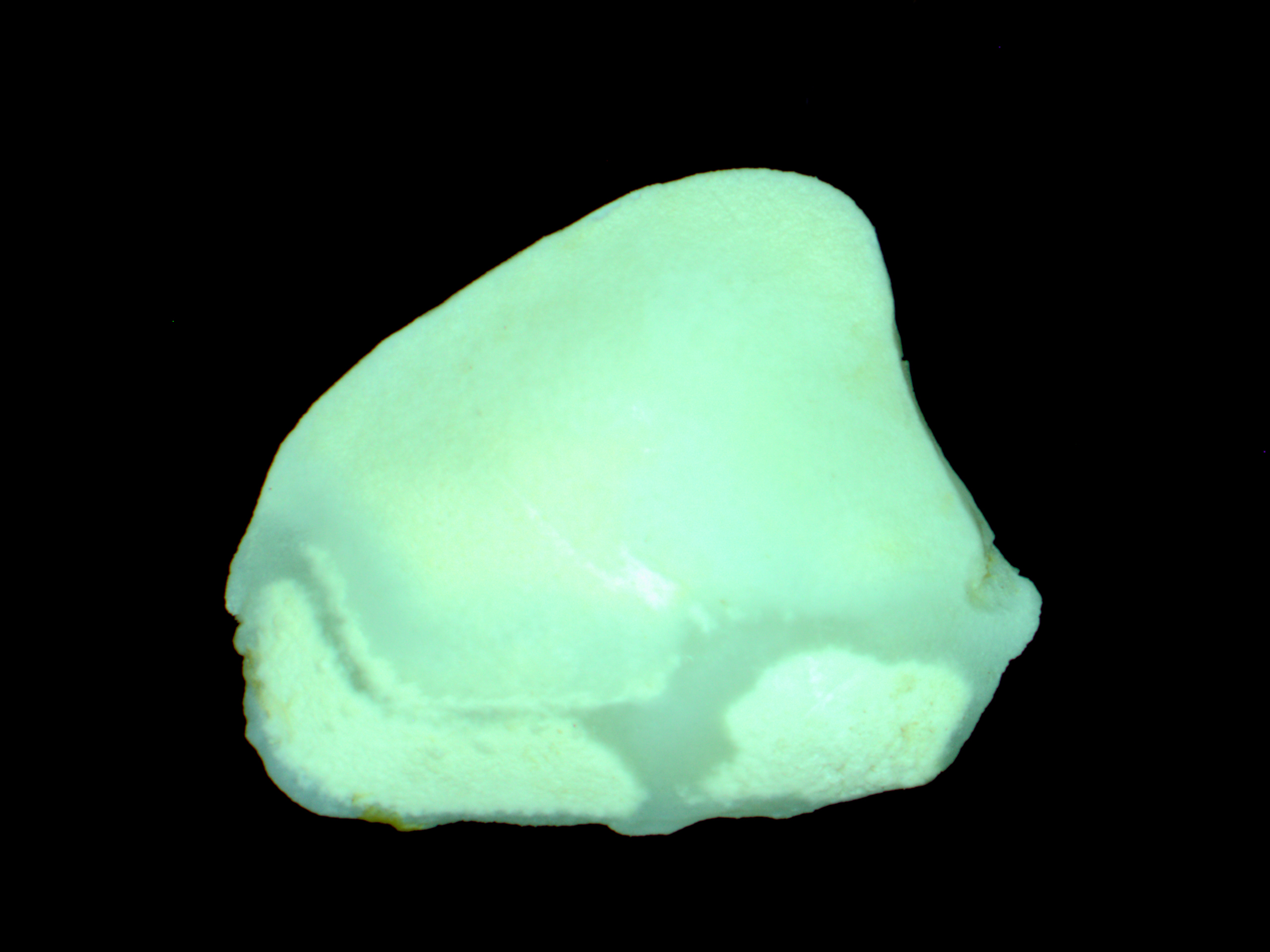

Supplement: Supplemental Information 6 [file peerj-04-1664-s006.zip › OstMil/training/ARI944_R1.jpg]

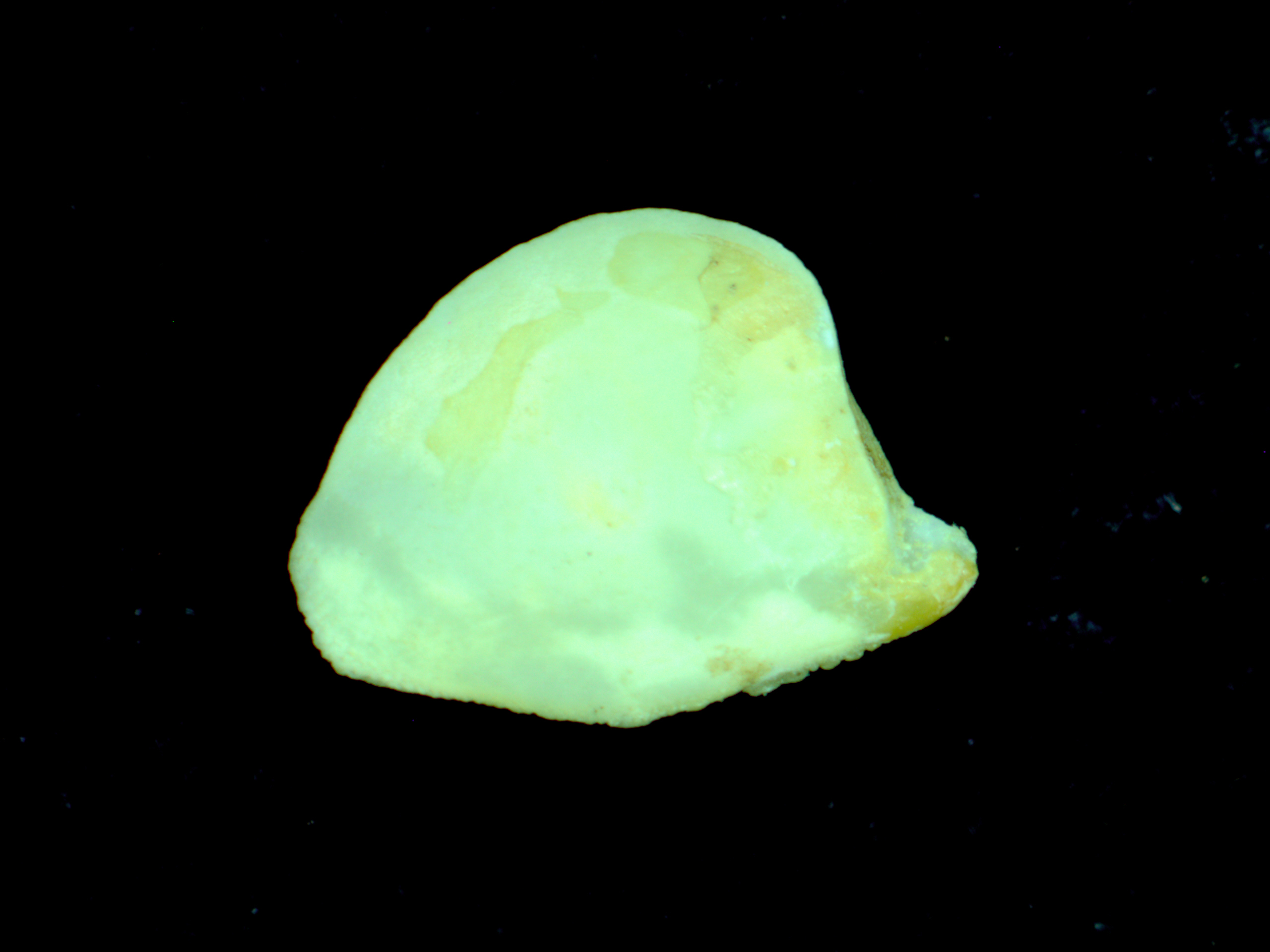

Supplement: Supplemental Information 7 [file peerj-04-1664-s007.zip › PliArg/testing/ARI203_R1.jpg]

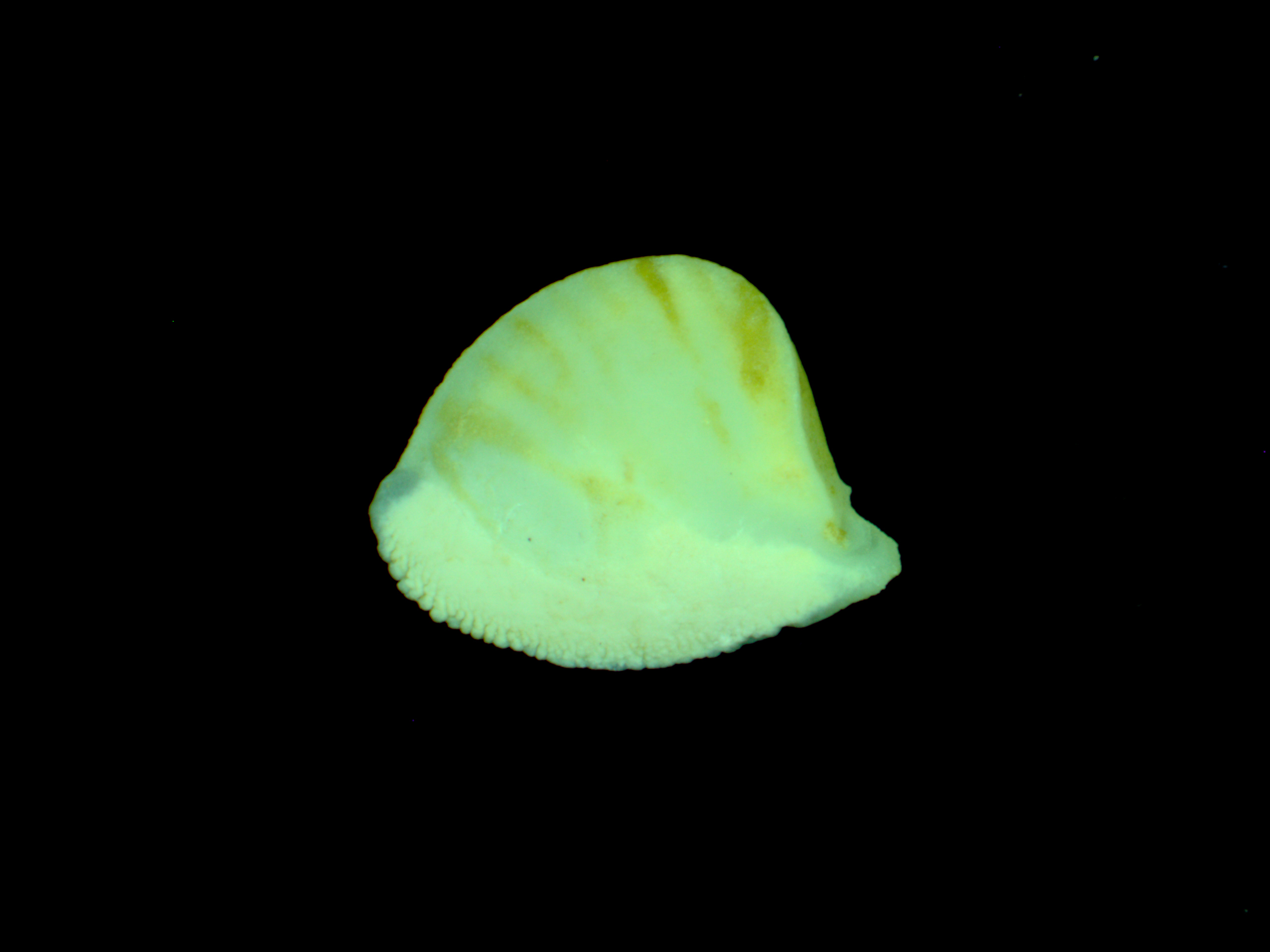

Supplement: Supplemental Information 7 [file peerj-04-1664-s007.zip › PliArg/testing/ARI214_R1.jpg]

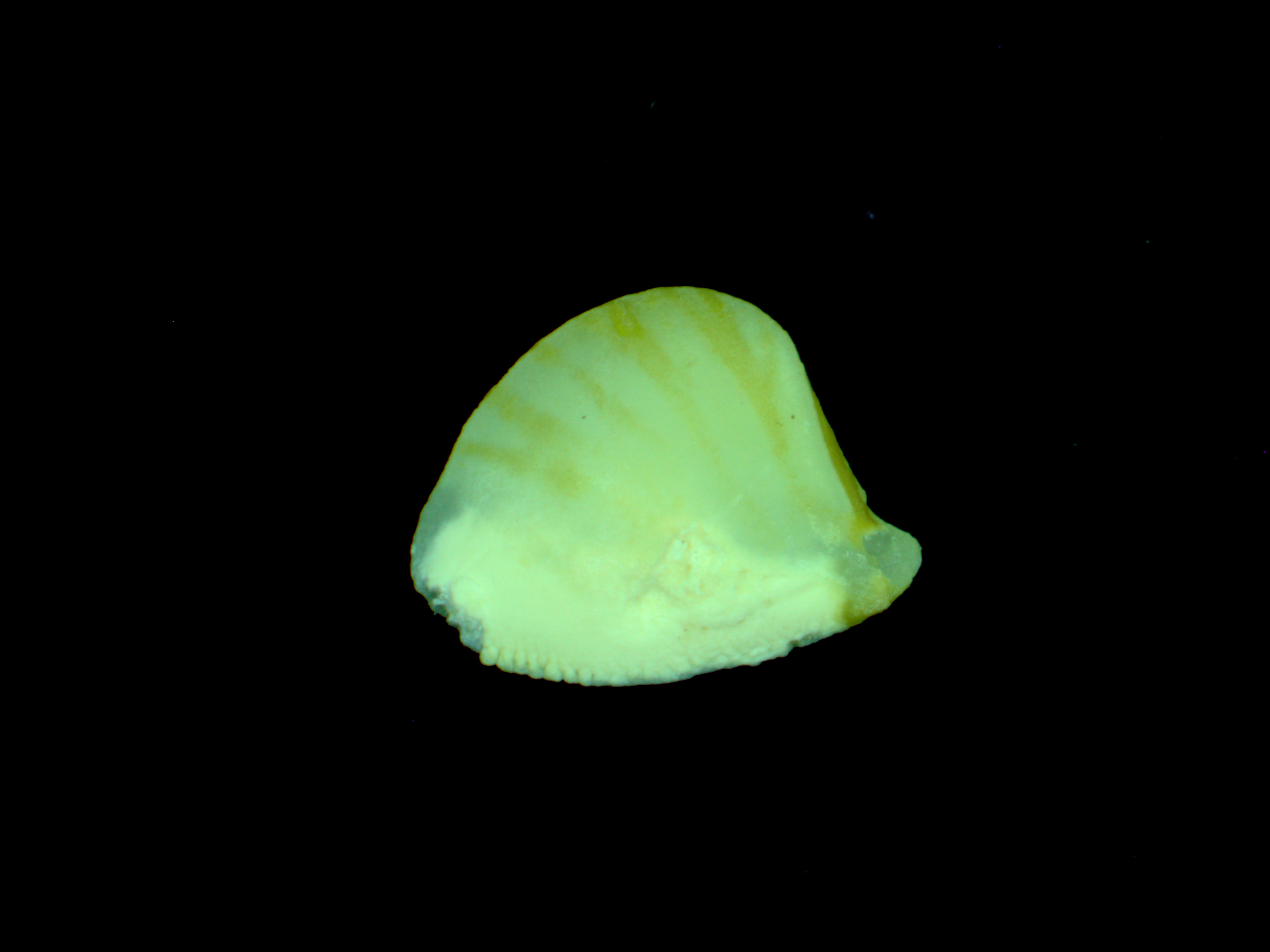

Supplement: Supplemental Information 7 [file peerj-04-1664-s007.zip › PliArg/testing/ARI215_R1.jpg]

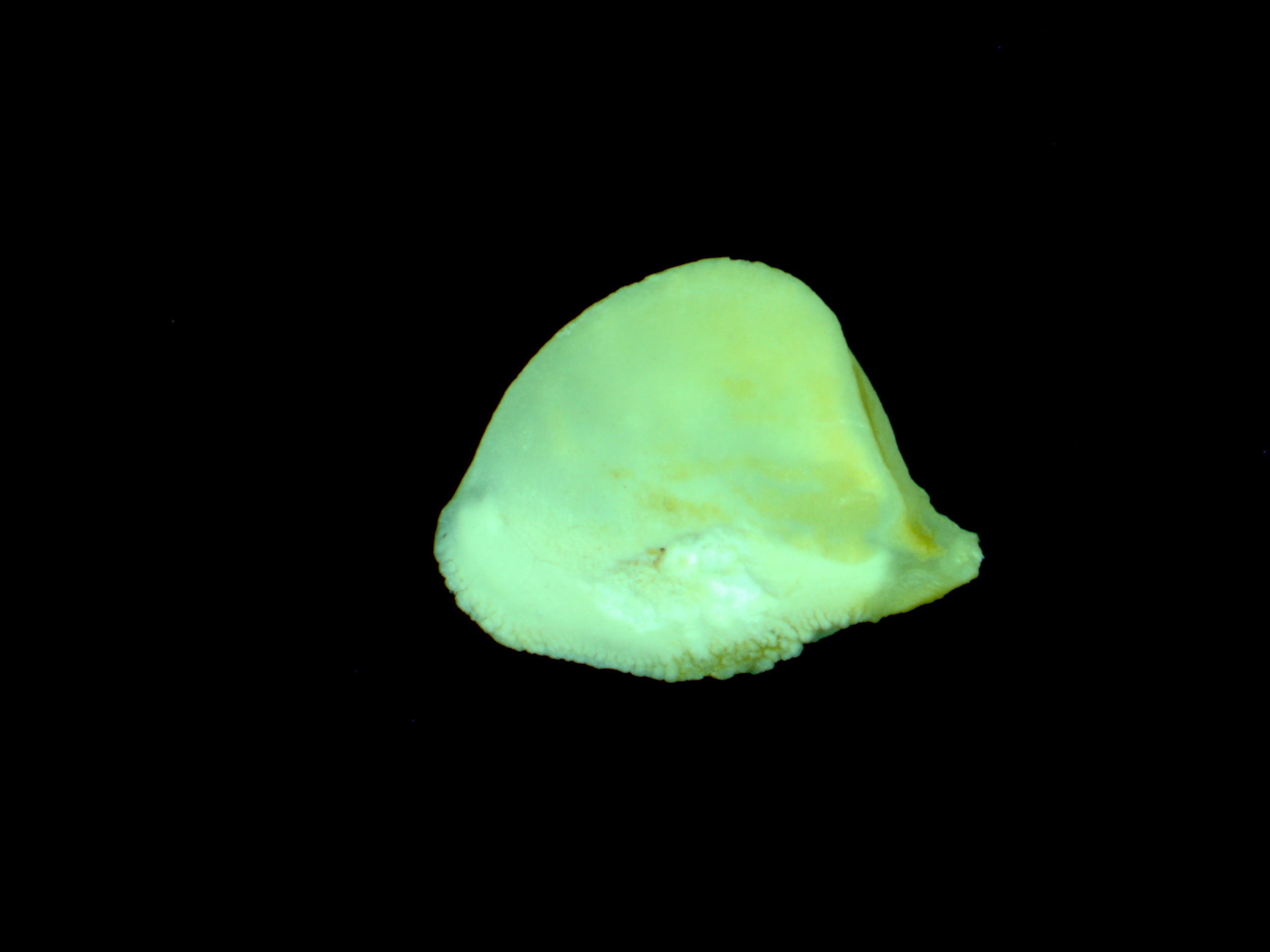

Supplement: Supplemental Information 7 [file peerj-04-1664-s007.zip › PliArg/testing/ARI216_R1.jpg]

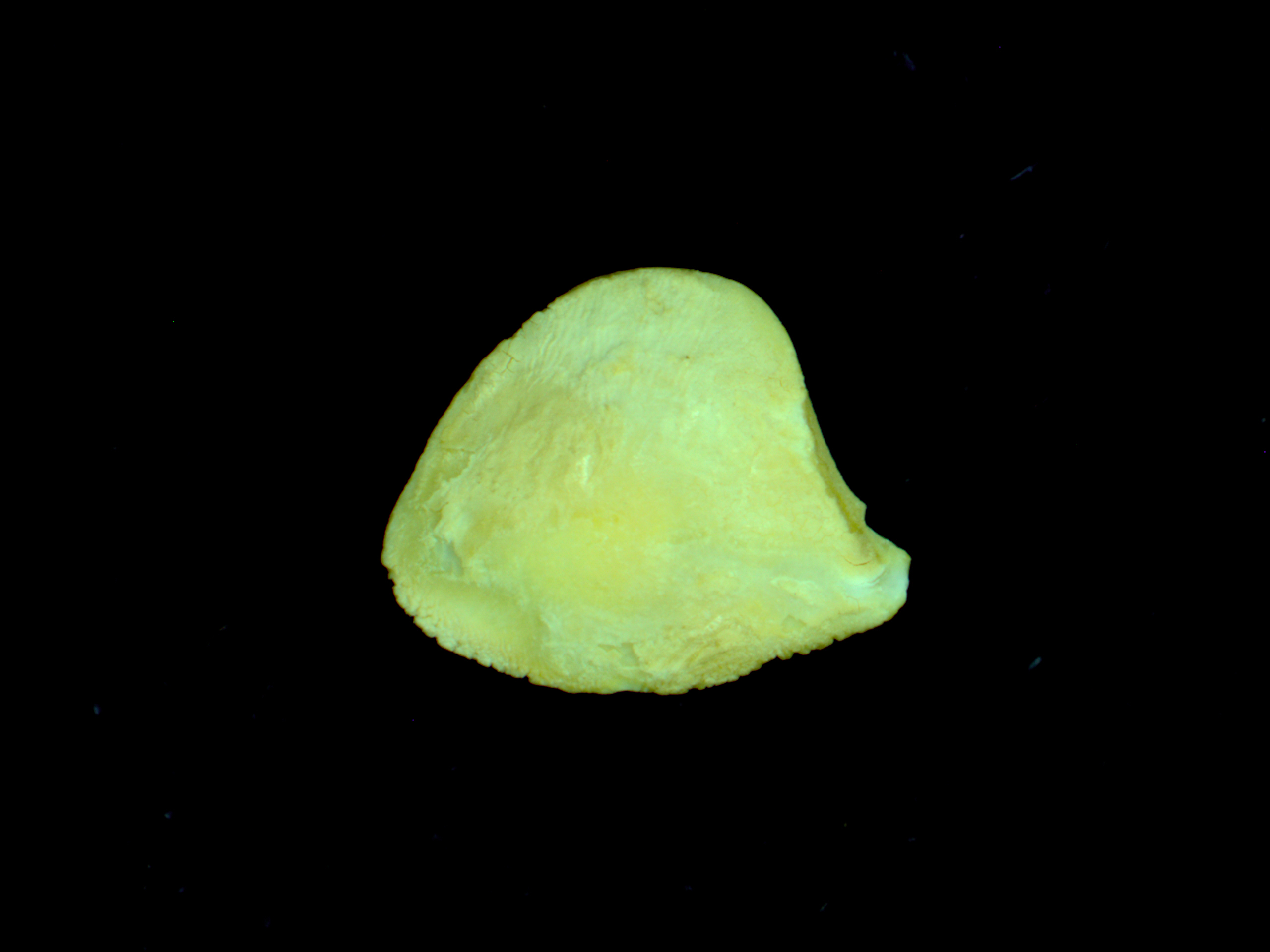

Supplement: Supplemental Information 7 [file peerj-04-1664-s007.zip › PliArg/testing/ARI221_R1.jpg]

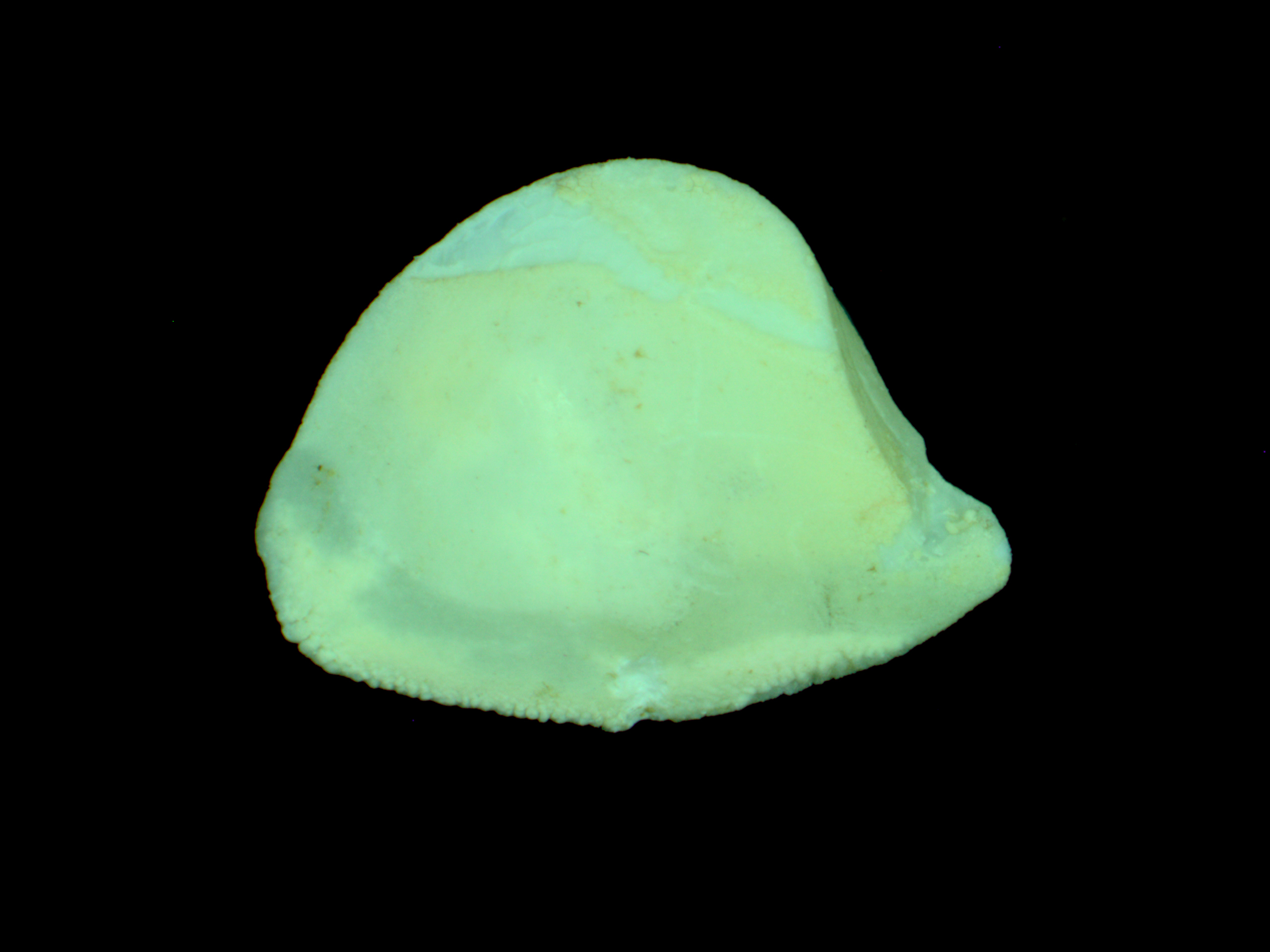

Supplement: Supplemental Information 7 [file peerj-04-1664-s007.zip › PliArg/testing/ARI272_R1.jpg]

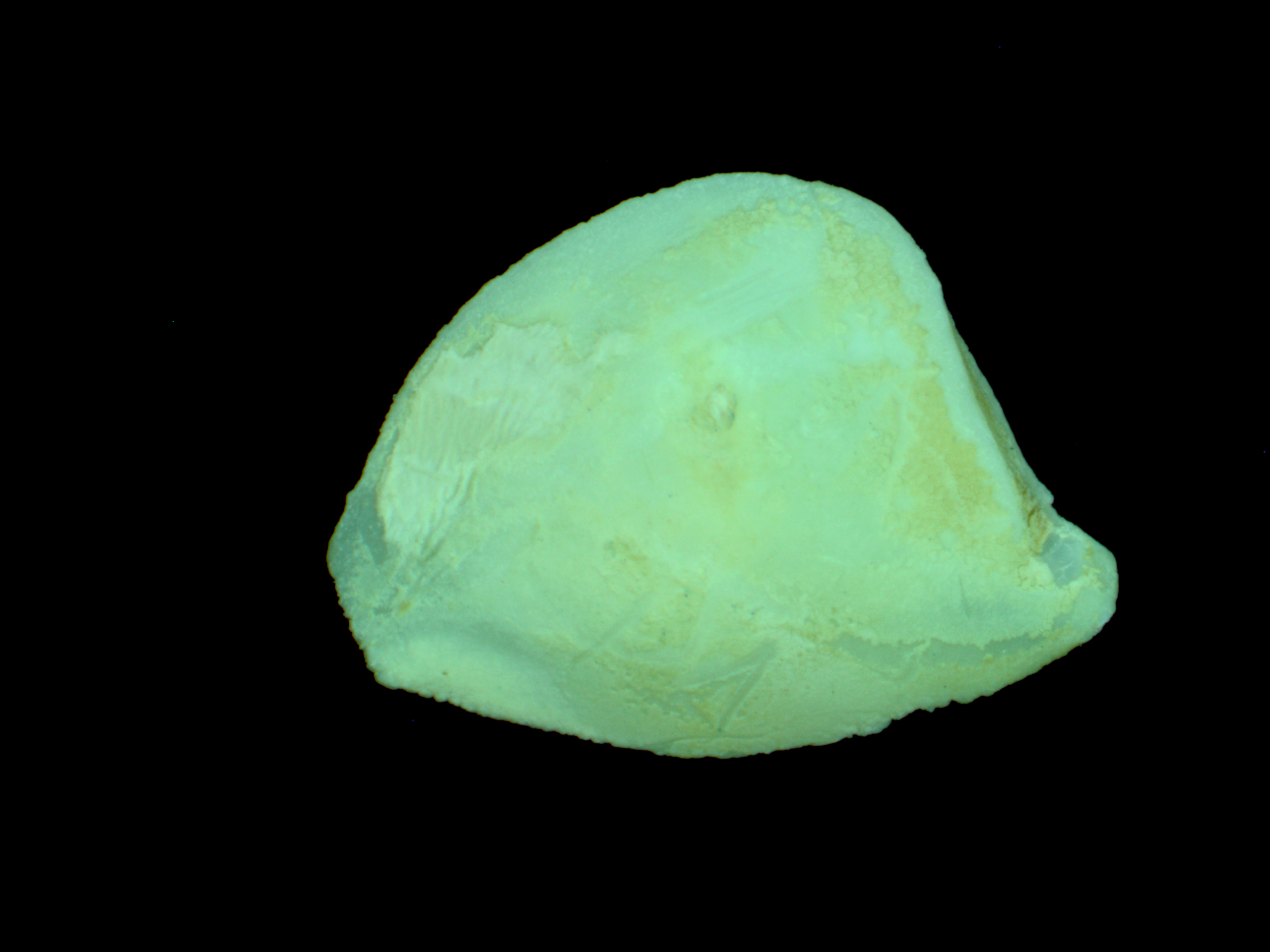

Supplement: Supplemental Information 7 [file peerj-04-1664-s007.zip › PliArg/testing/ARI273_R1.jpg]

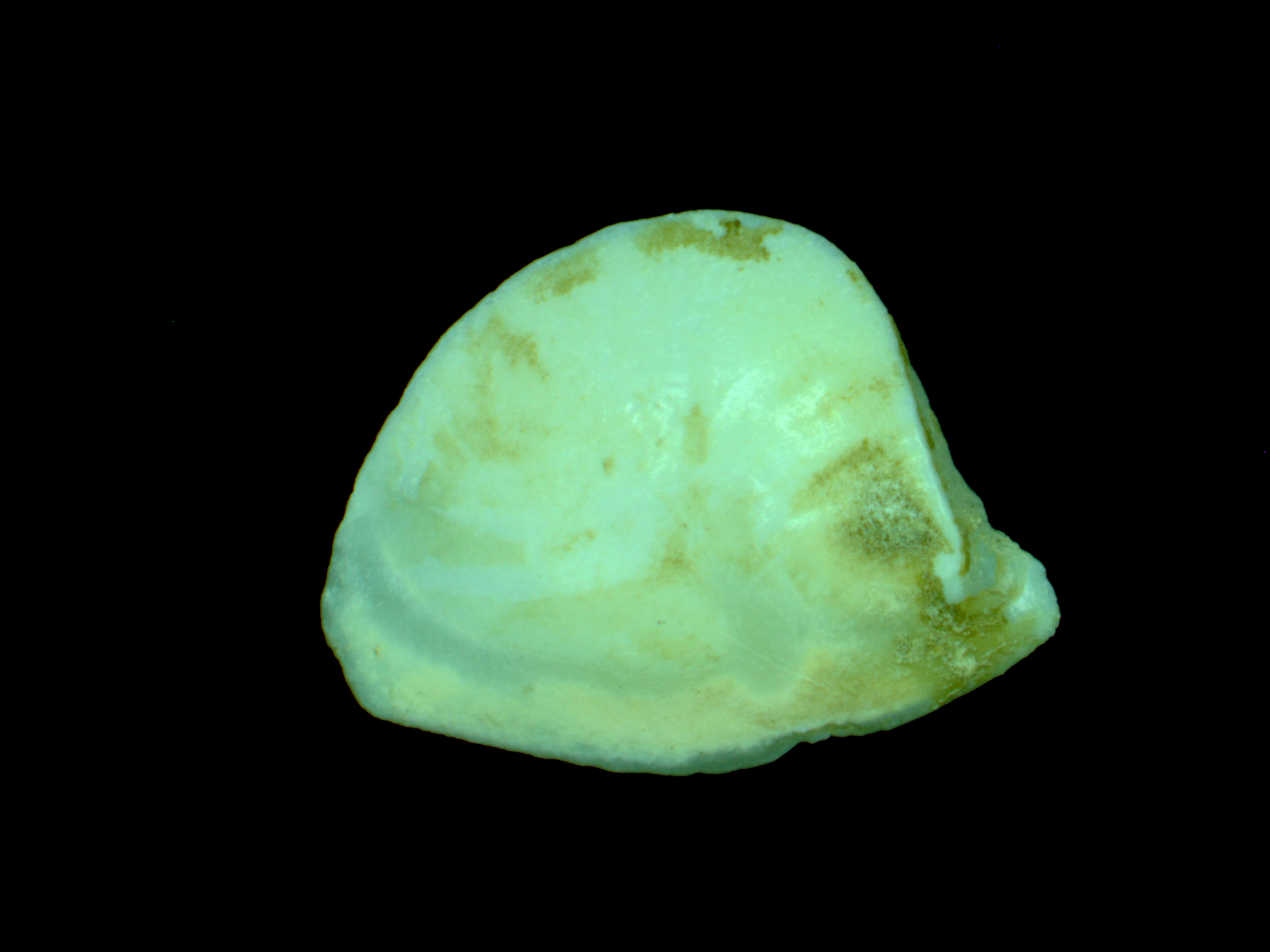

Supplement: Supplemental Information 7 [file peerj-04-1664-s007.zip › PliArg/testing/ARI275_R1.jpg]

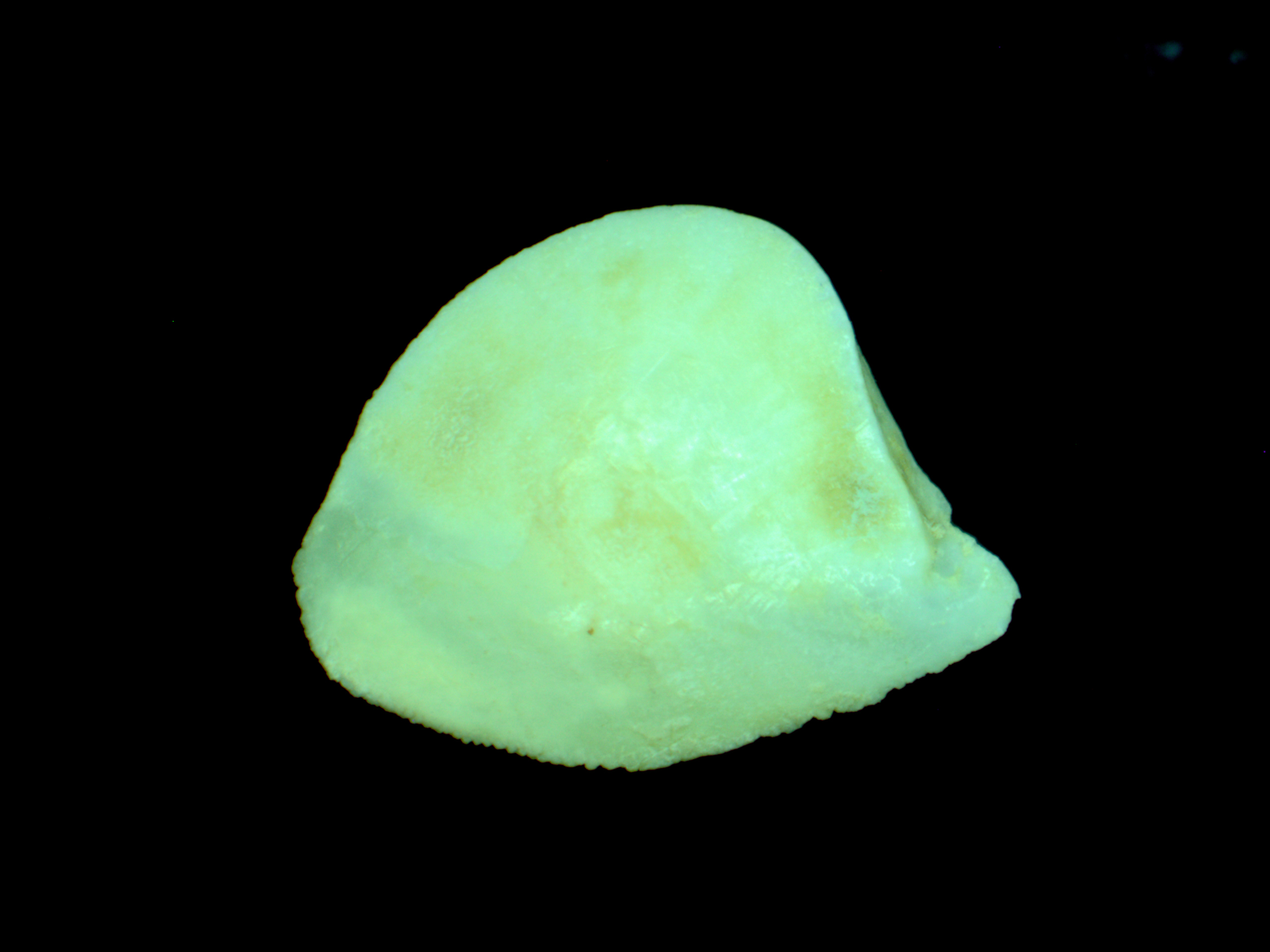

Supplement: Supplemental Information 7 [file peerj-04-1664-s007.zip › PliArg/testing/ARI277_R1.jpg]

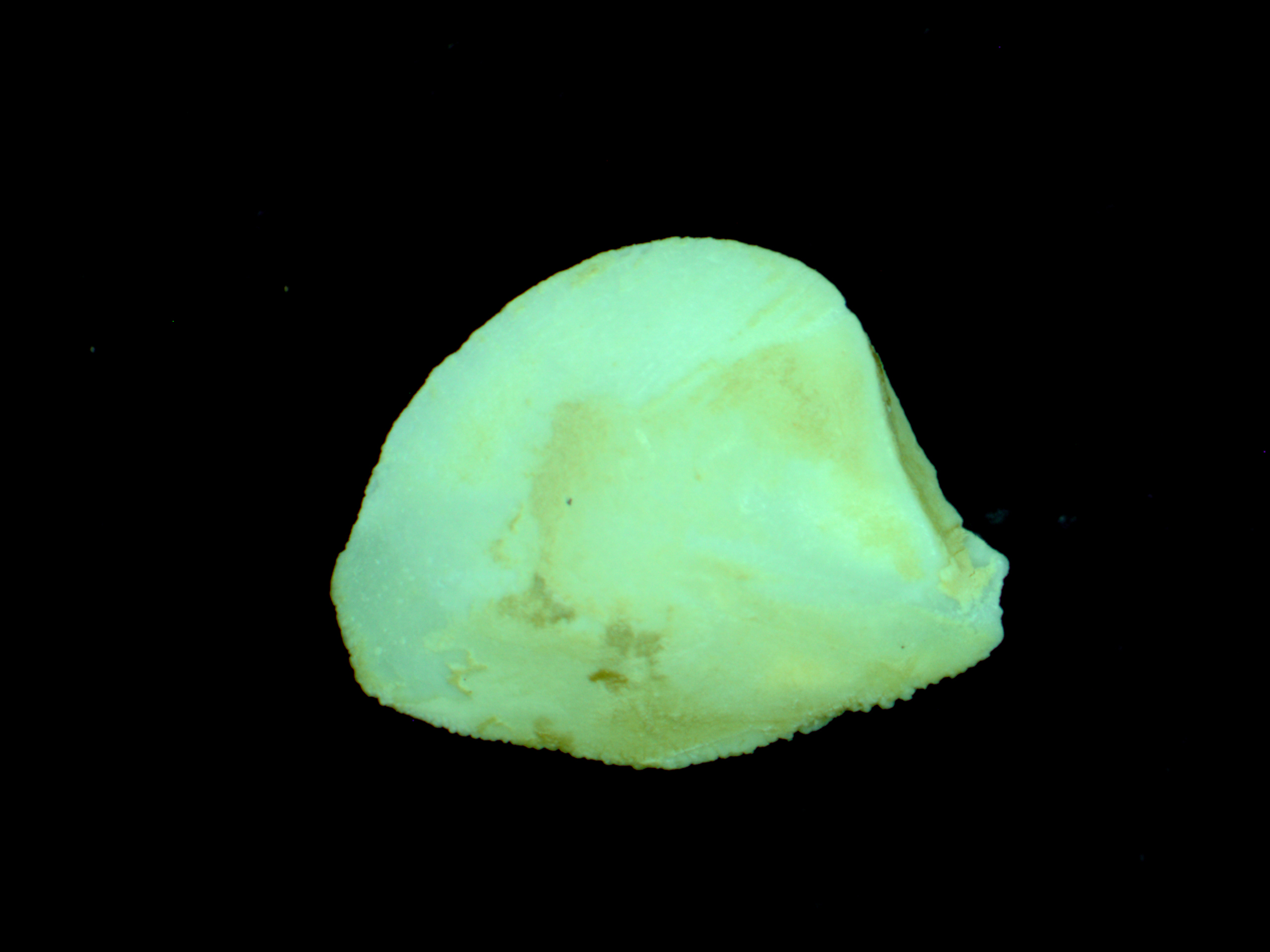

Supplement: Supplemental Information 7 [file peerj-04-1664-s007.zip › PliArg/testing/ARI278_R1.jpg]

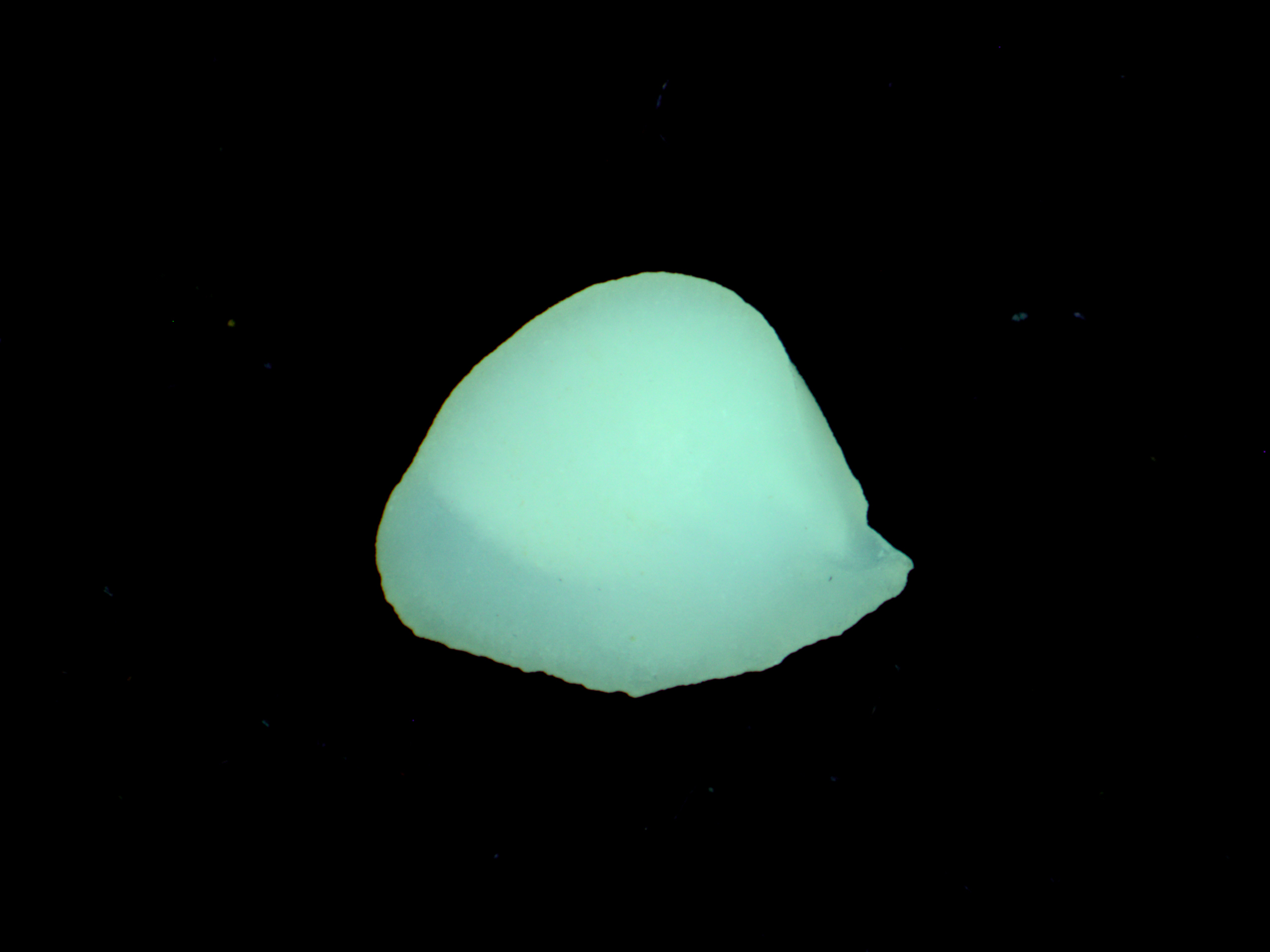

Supplement: Supplemental Information 7 [file peerj-04-1664-s007.zip › PliArg/training/ARI10_R1.jpg]

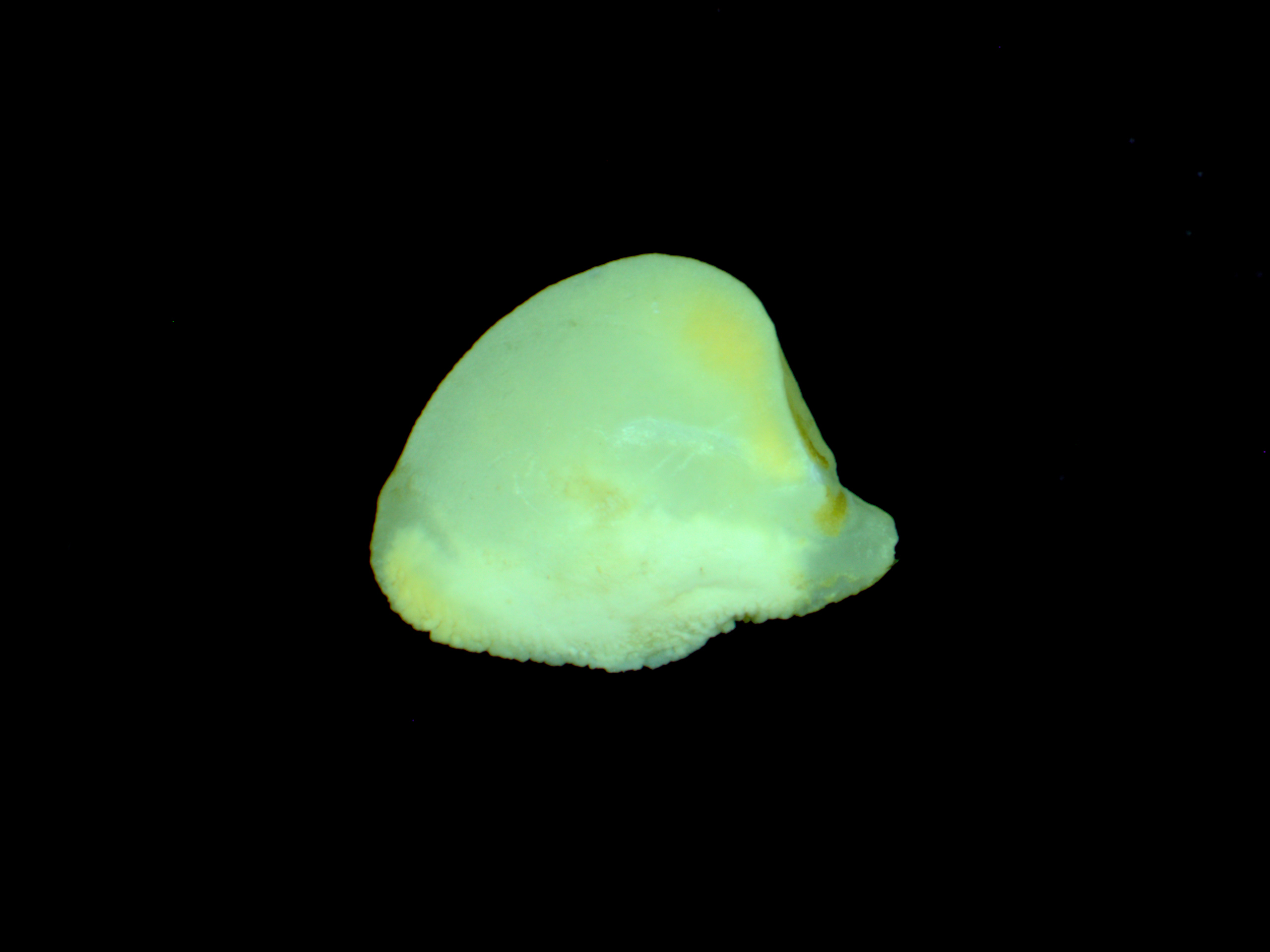

Supplement: Supplemental Information 7 [file peerj-04-1664-s007.zip › PliArg/training/ARI115_R1.jpg]

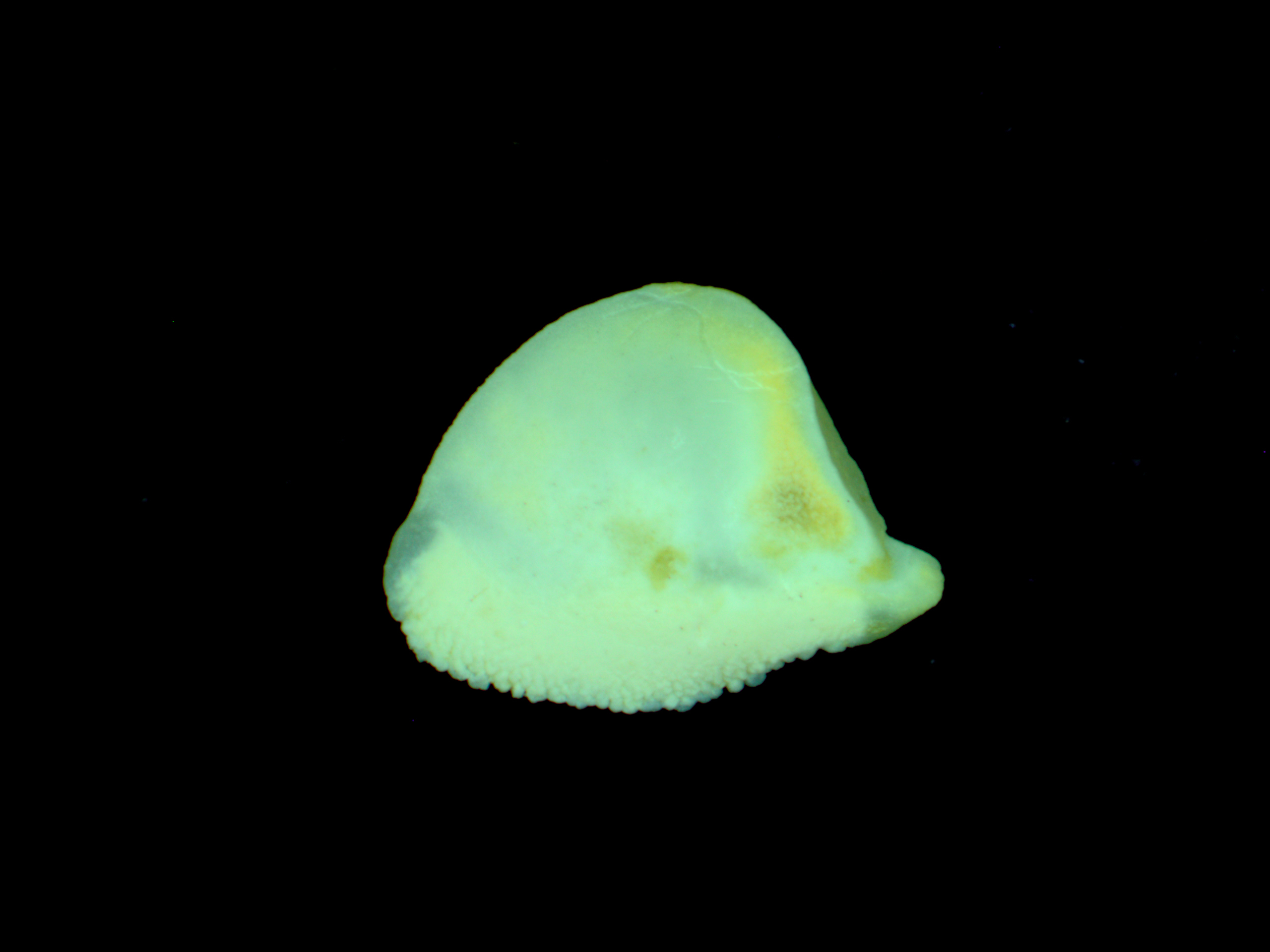

Supplement: Supplemental Information 7 [file peerj-04-1664-s007.zip › PliArg/training/ARI117_R1.jpg]

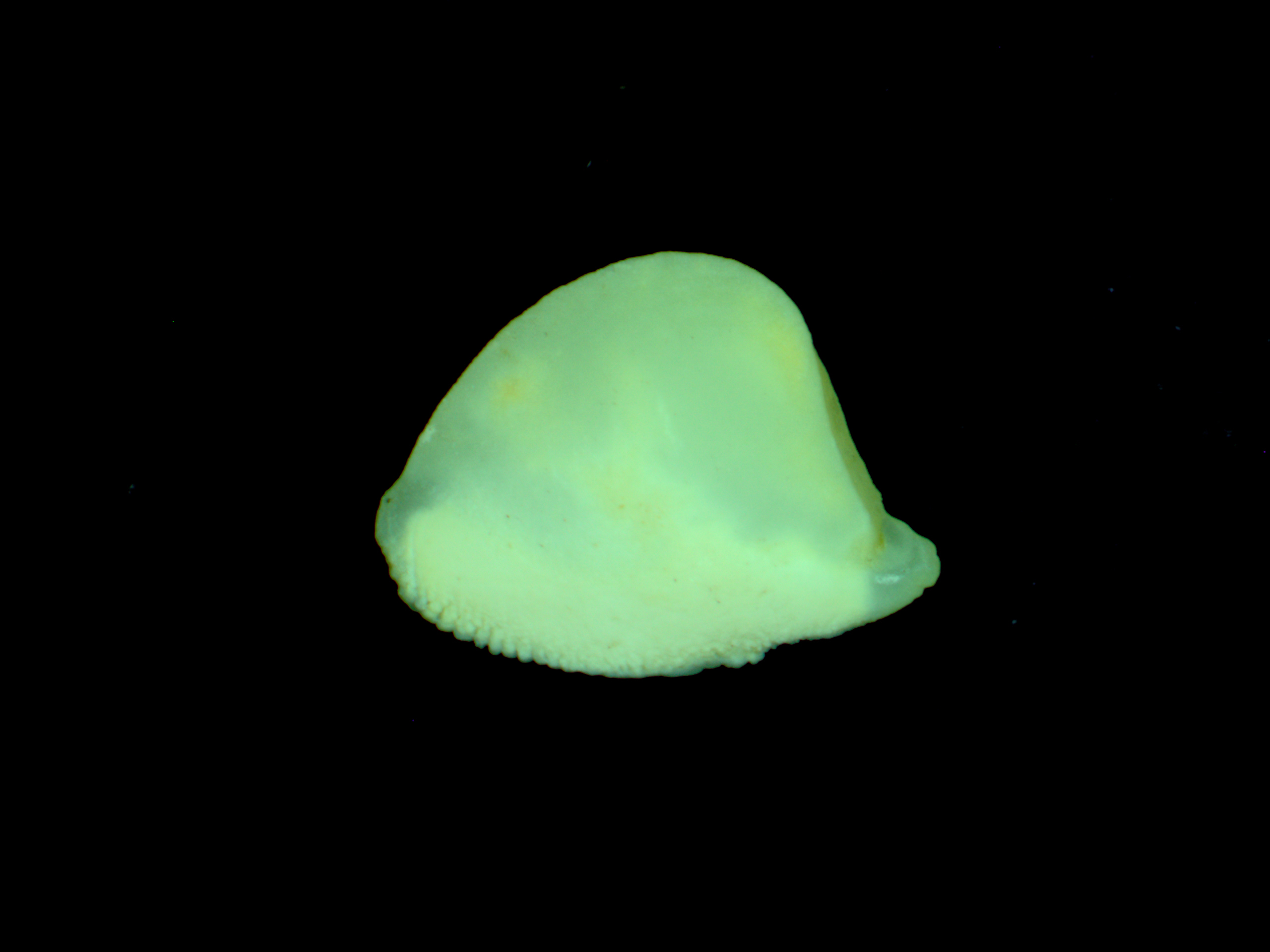

Supplement: Supplemental Information 7 [file peerj-04-1664-s007.zip › PliArg/training/ARI118_R1.jpg]

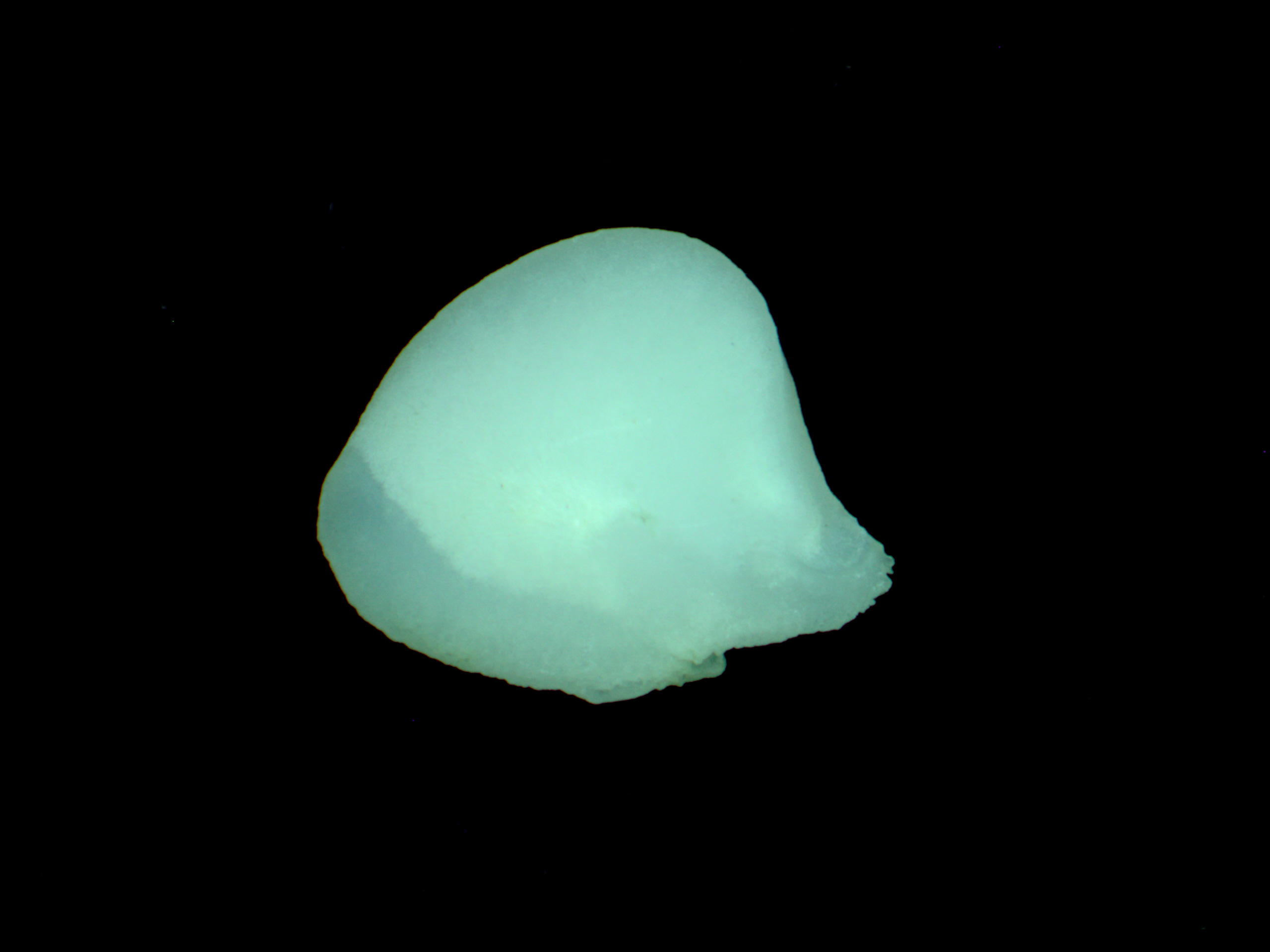

Supplement: Supplemental Information 7 [file peerj-04-1664-s007.zip › PliArg/training/ARI11_R1.jpg]

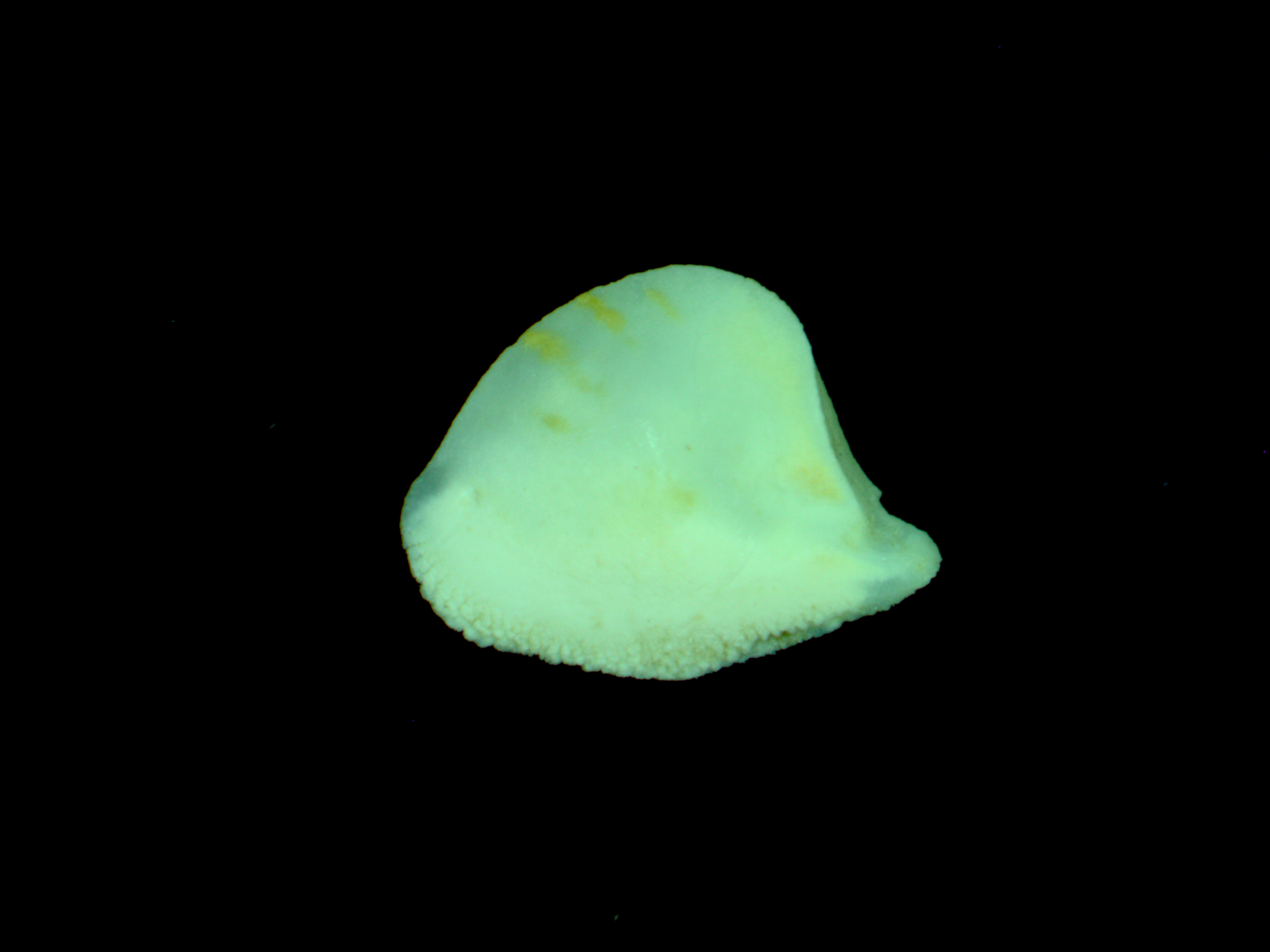

Supplement: Supplemental Information 7 [file peerj-04-1664-s007.zip › PliArg/training/ARI121_R1.jpg]

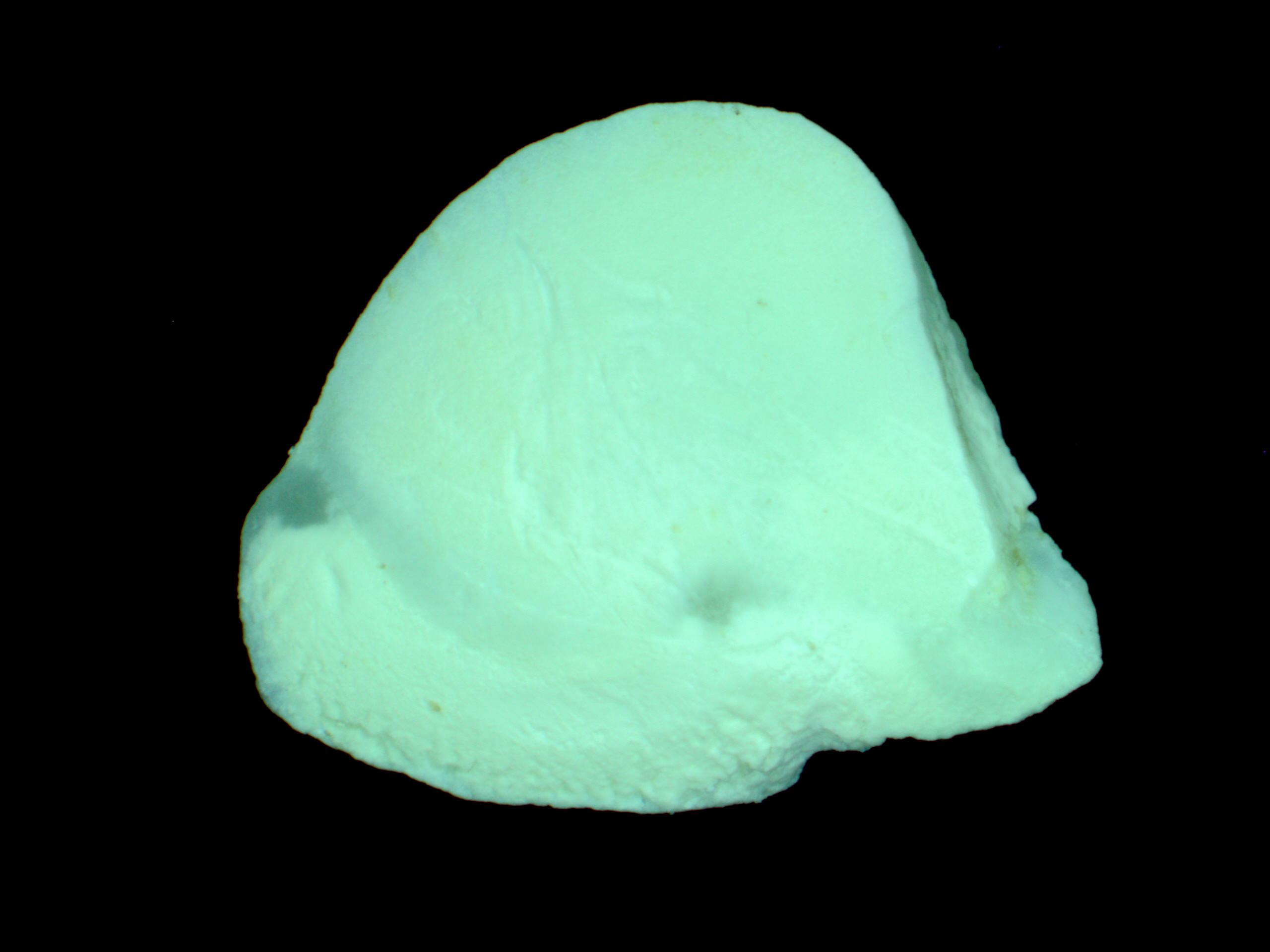

Supplement: Supplemental Information 7 [file peerj-04-1664-s007.zip › PliArg/training/ARI122_R1.jpg]

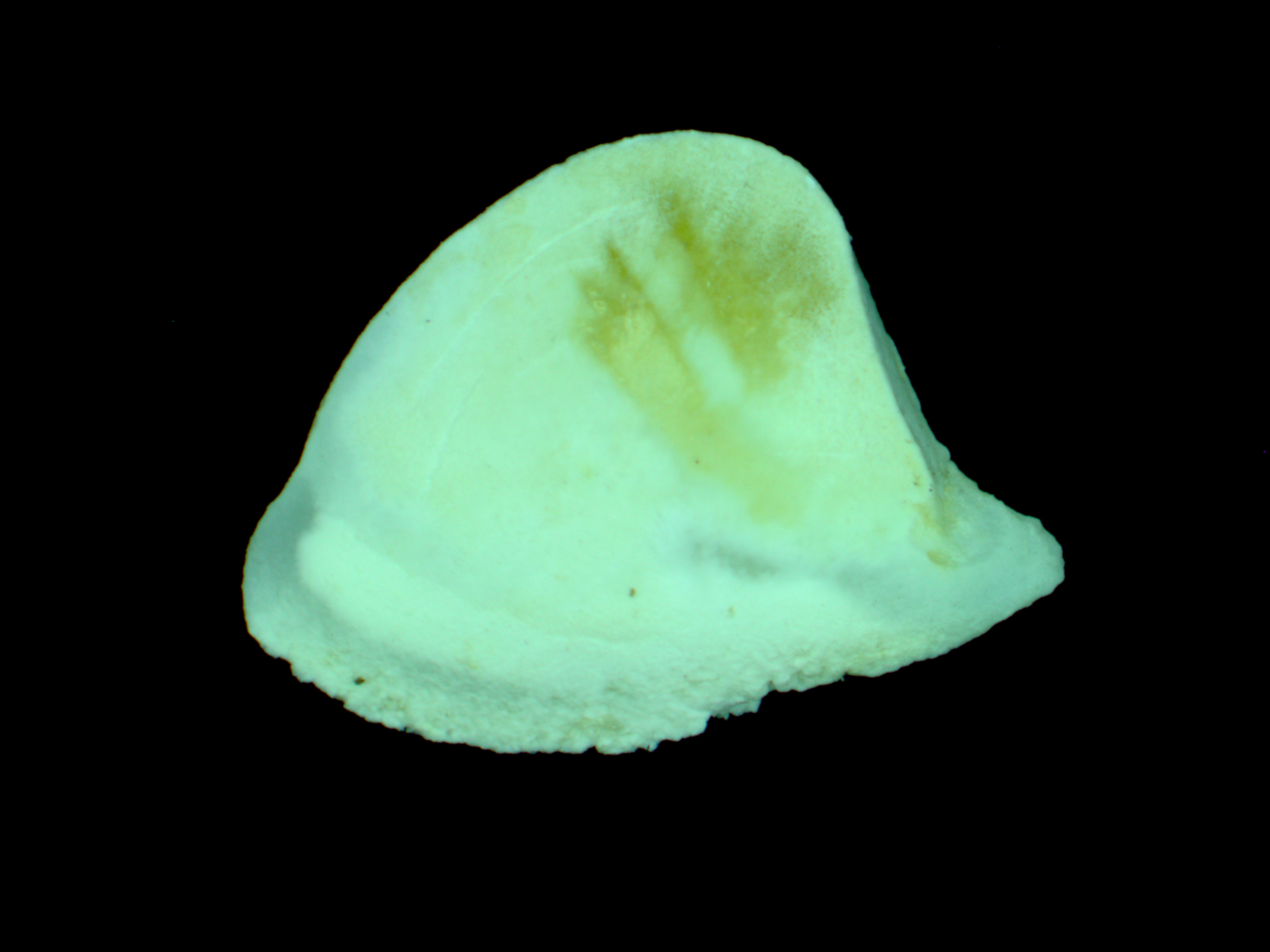

Supplement: Supplemental Information 7 [file peerj-04-1664-s007.zip › PliArg/training/ARI123_R1.jpg]

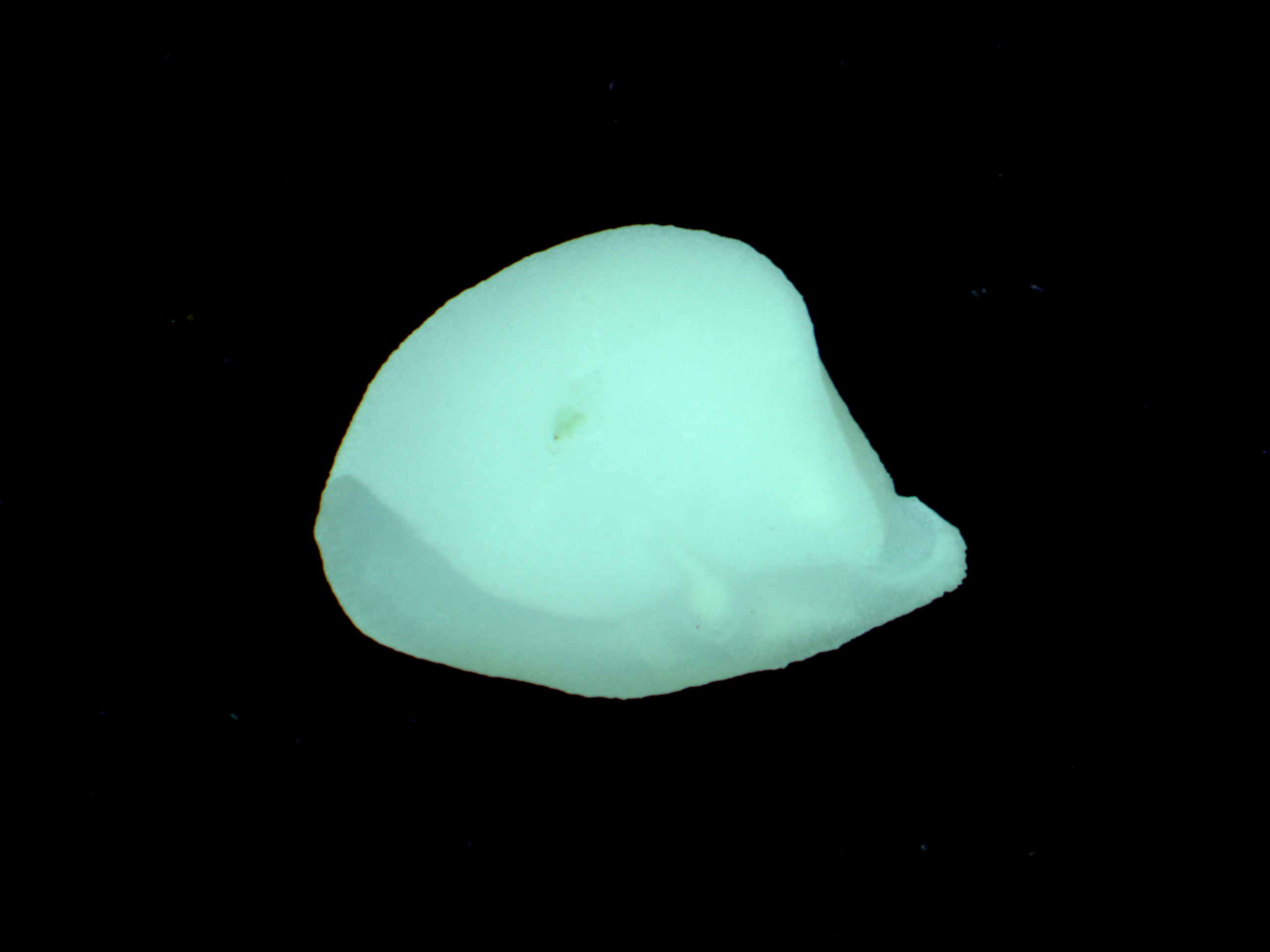

Supplement: Supplemental Information 7 [file peerj-04-1664-s007.zip › PliArg/training/ARI12_R1.jpg]

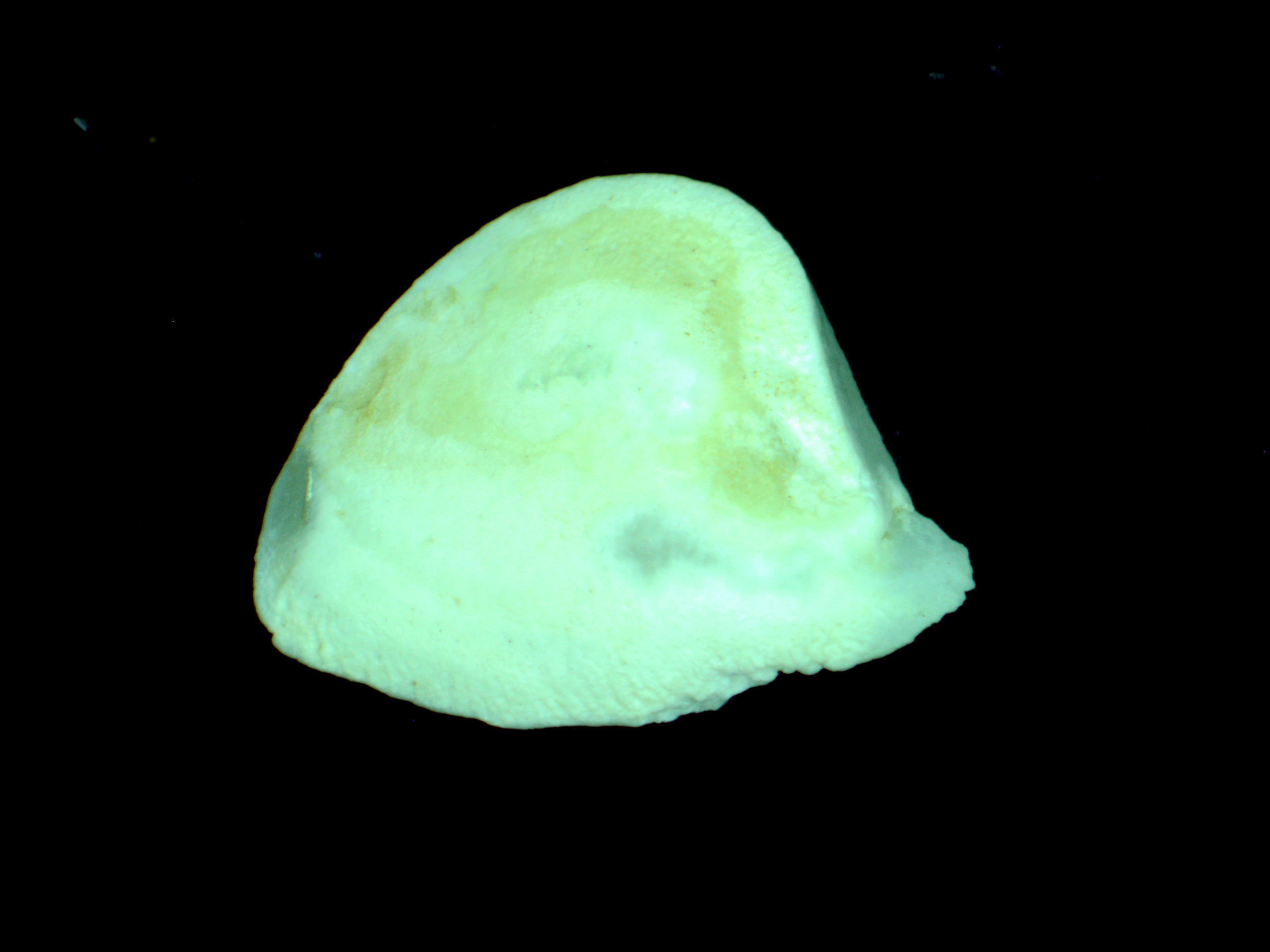

Supplement: Supplemental Information 7 [file peerj-04-1664-s007.zip › PliArg/training/ARI15_R1.jpg]

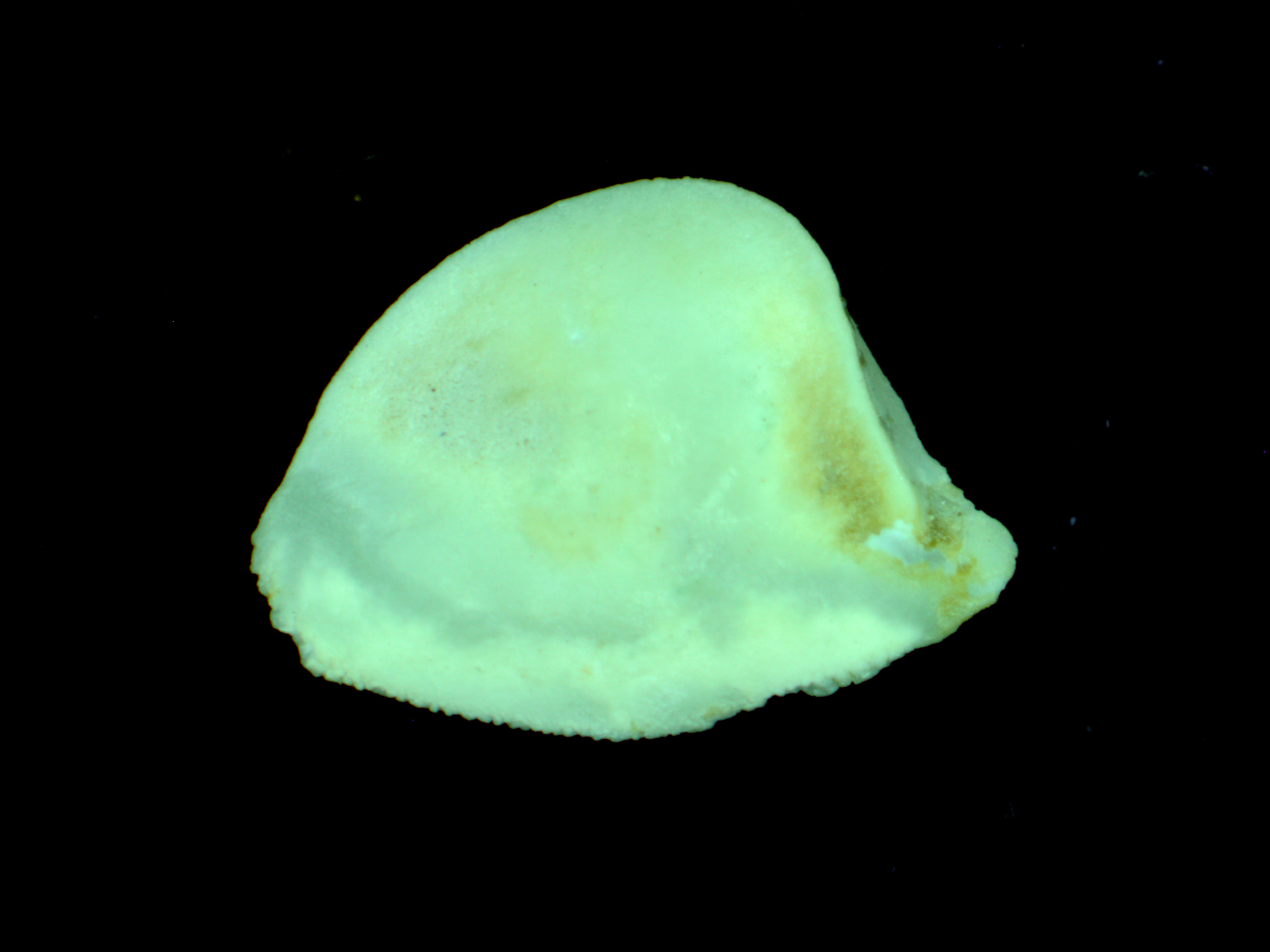

Supplement: Supplemental Information 7 [file peerj-04-1664-s007.zip › PliArg/training/ARI17_R1.jpg]

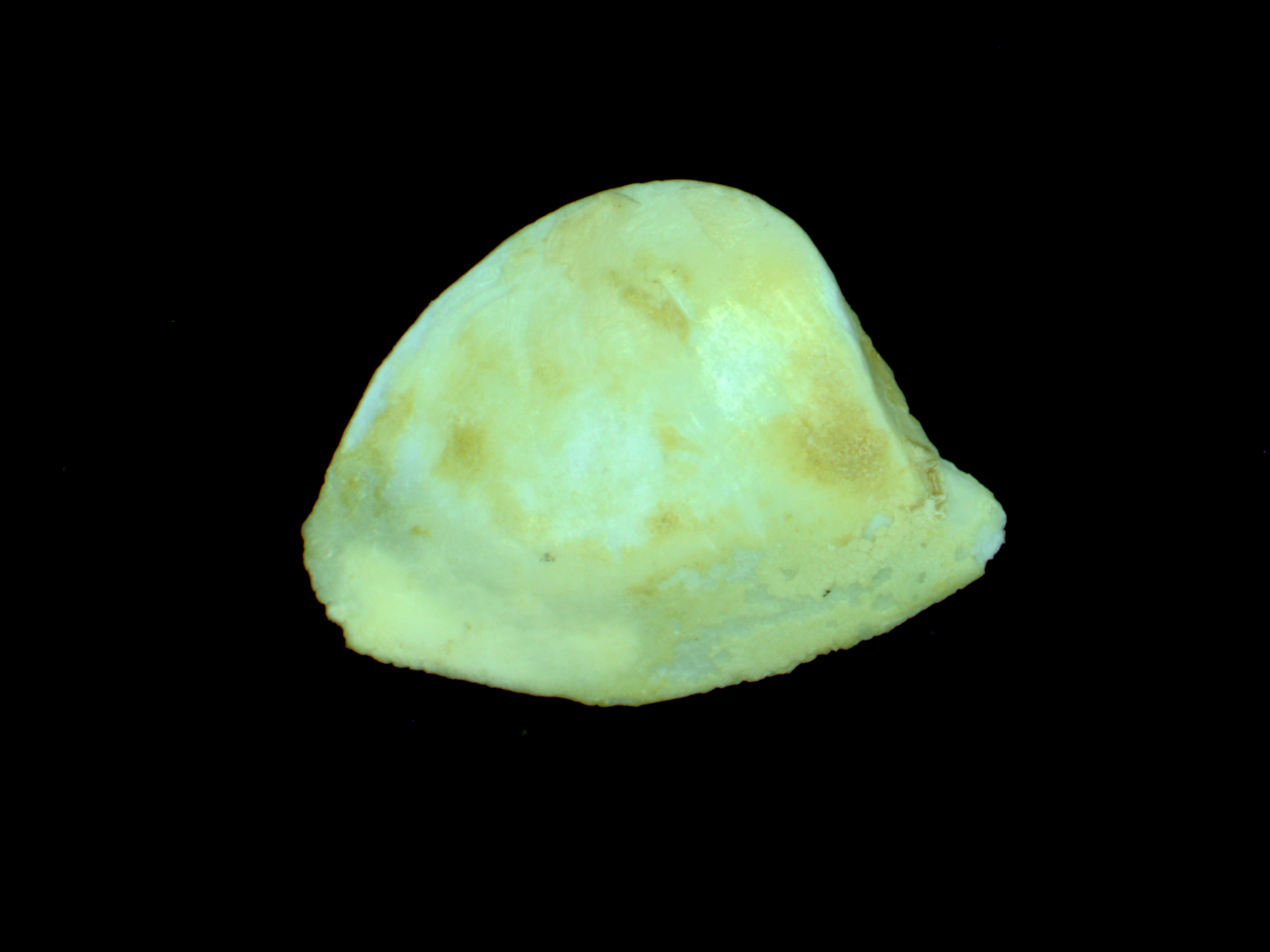

Supplement: Supplemental Information 7 [file peerj-04-1664-s007.zip › PliArg/training/ARI19_R1.jpg]

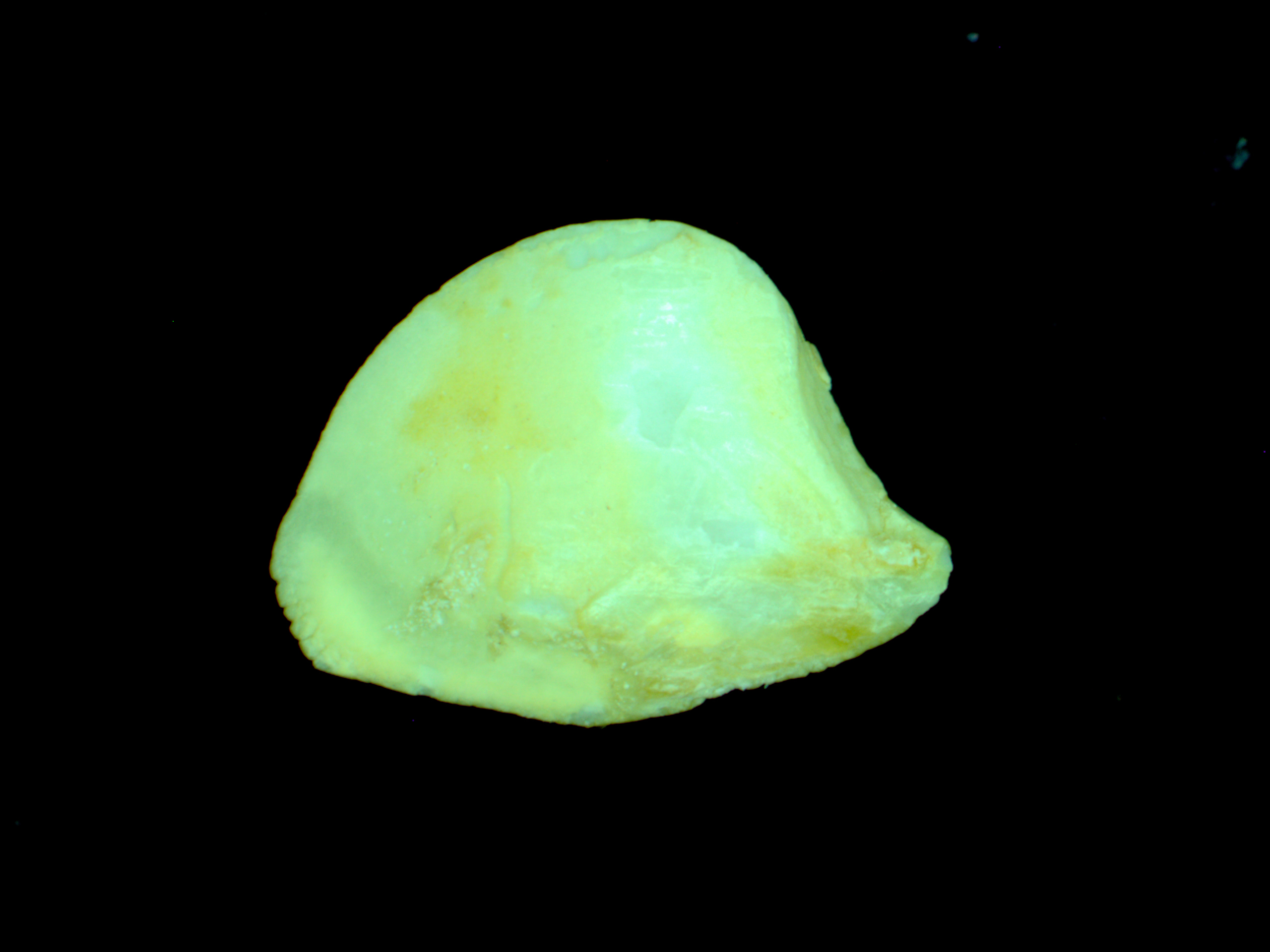

Supplement: Supplemental Information 7 [file peerj-04-1664-s007.zip › PliArg/training/ARI23_R1.jpg]

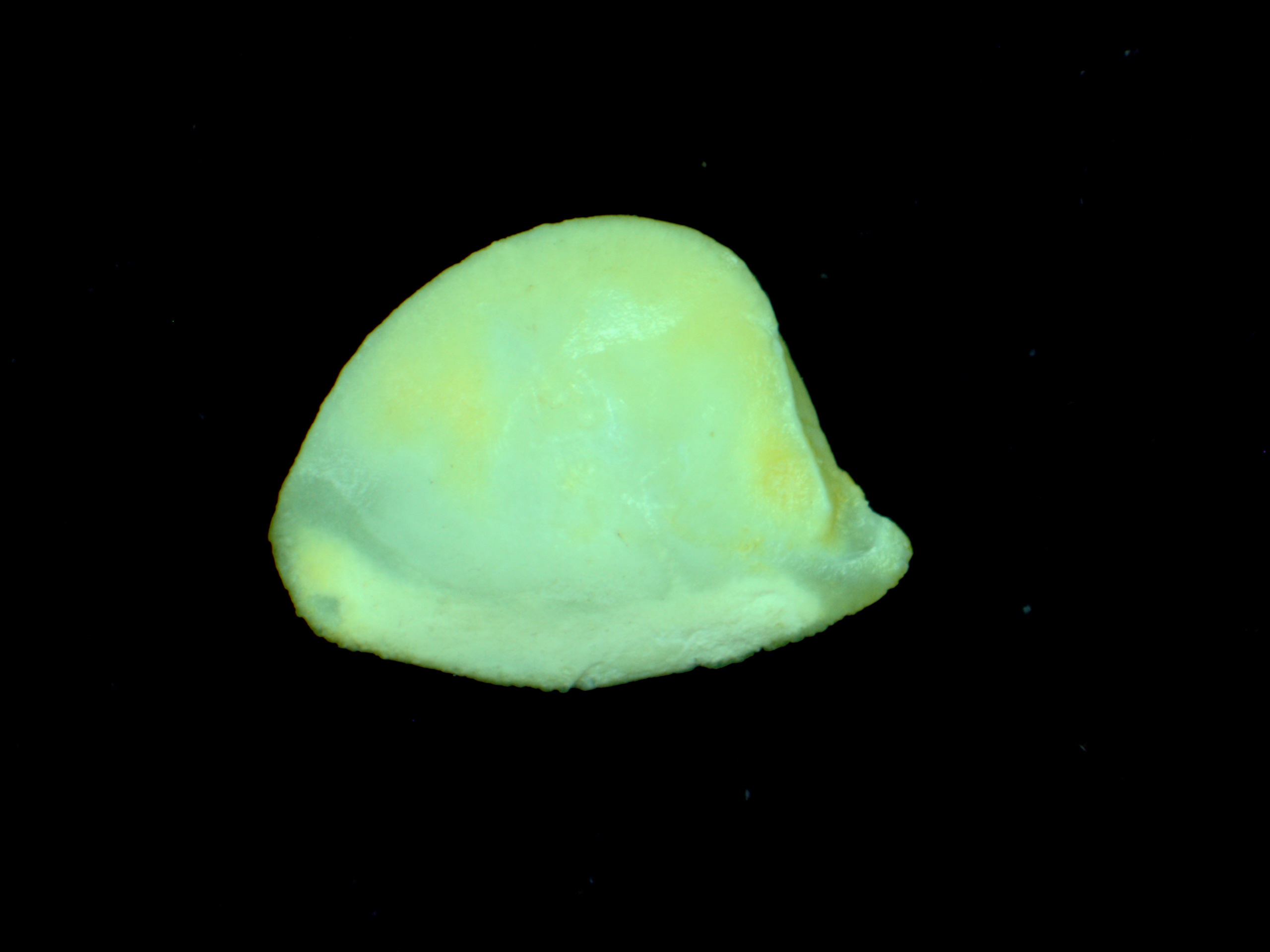

Supplement: Supplemental Information 7 [file peerj-04-1664-s007.zip › PliArg/training/ARI25_R1.jpg]

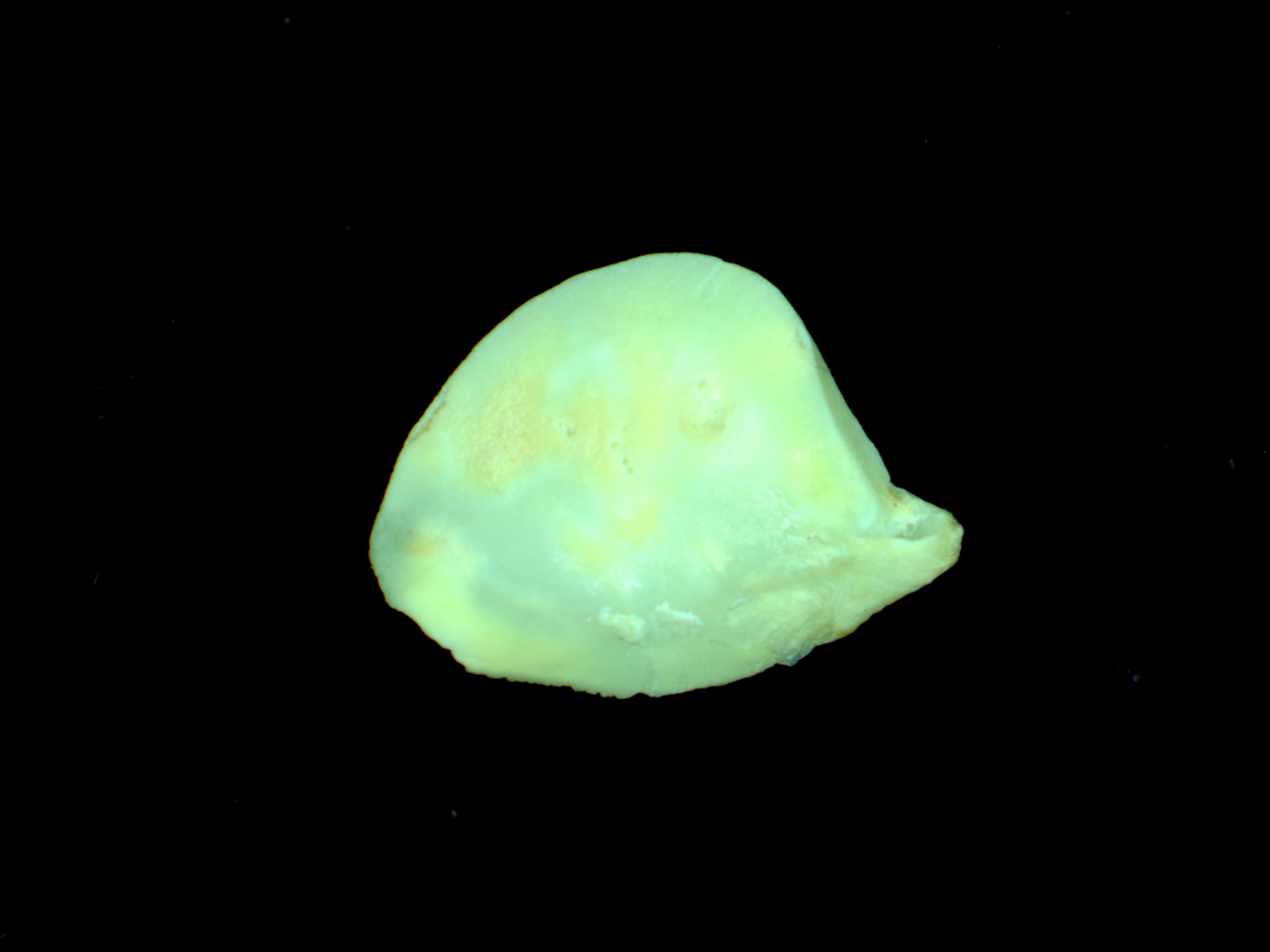

Supplement: Supplemental Information 7 [file peerj-04-1664-s007.zip › PliArg/training/ARI29_R1.jpg]

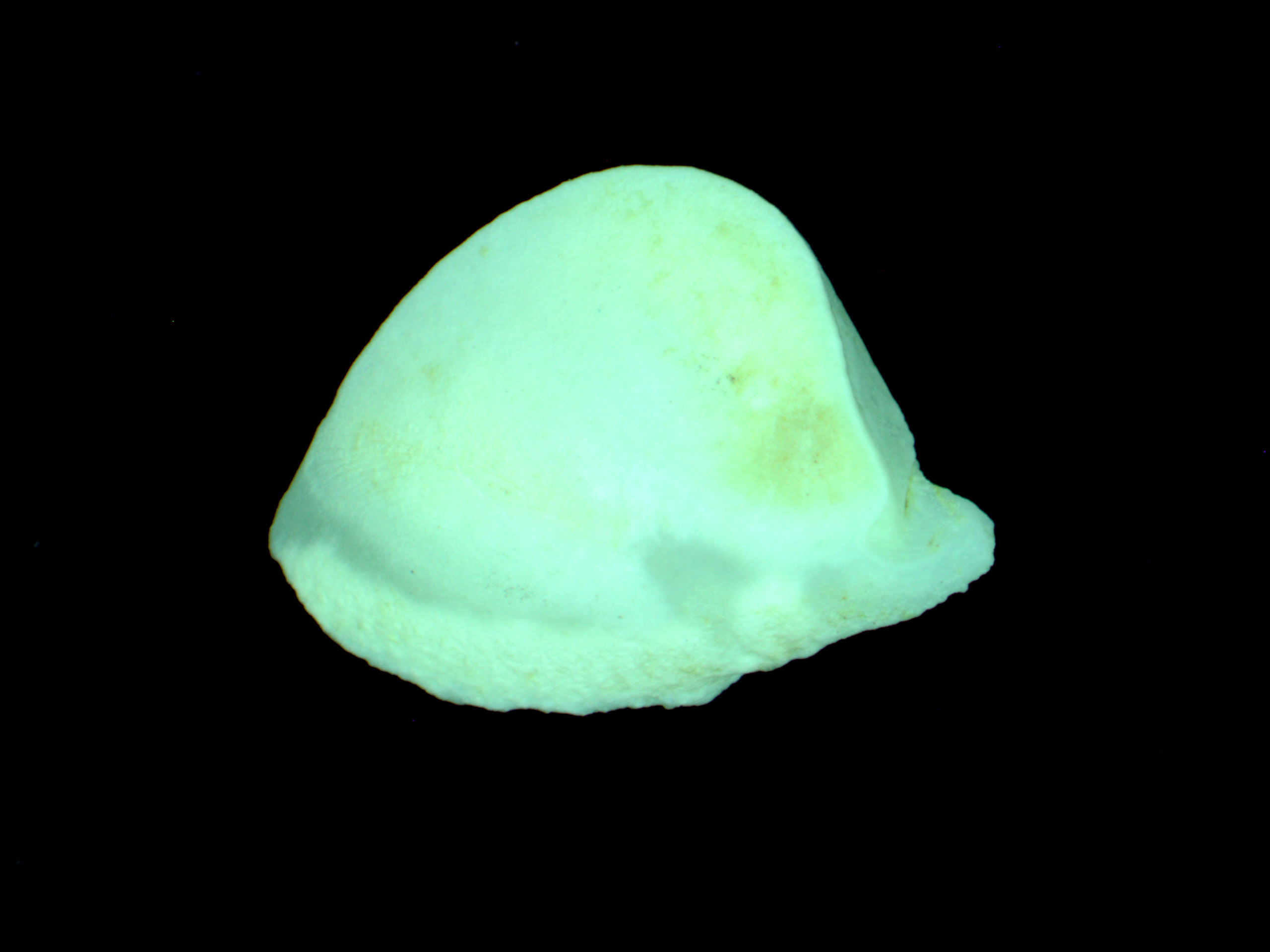

Supplement: Supplemental Information 7 [file peerj-04-1664-s007.zip › PliArg/training/ARI61_R1.jpg]

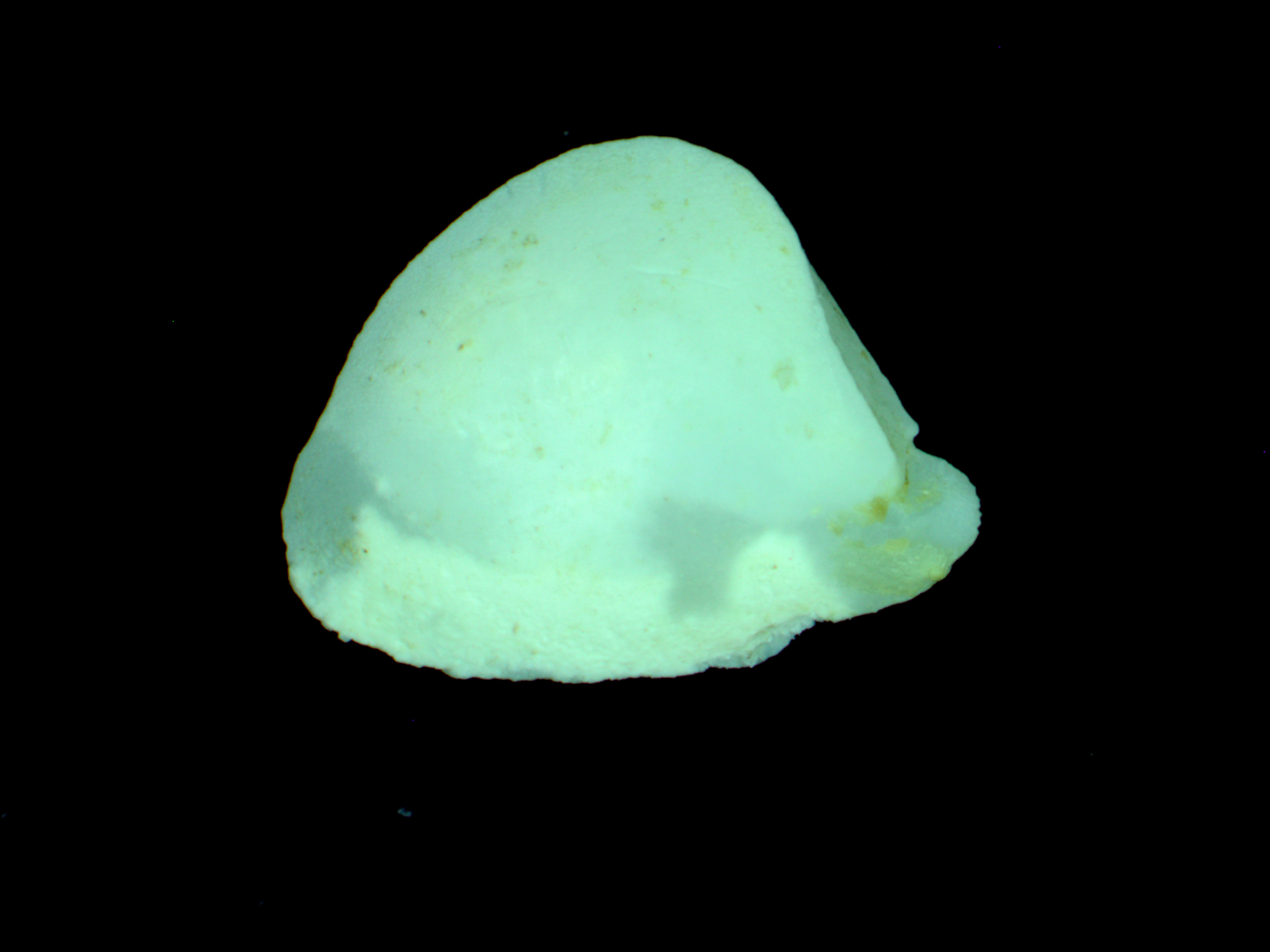

Supplement: Supplemental Information 7 [file peerj-04-1664-s007.zip › PliArg/training/ARI63_R1.jpg]

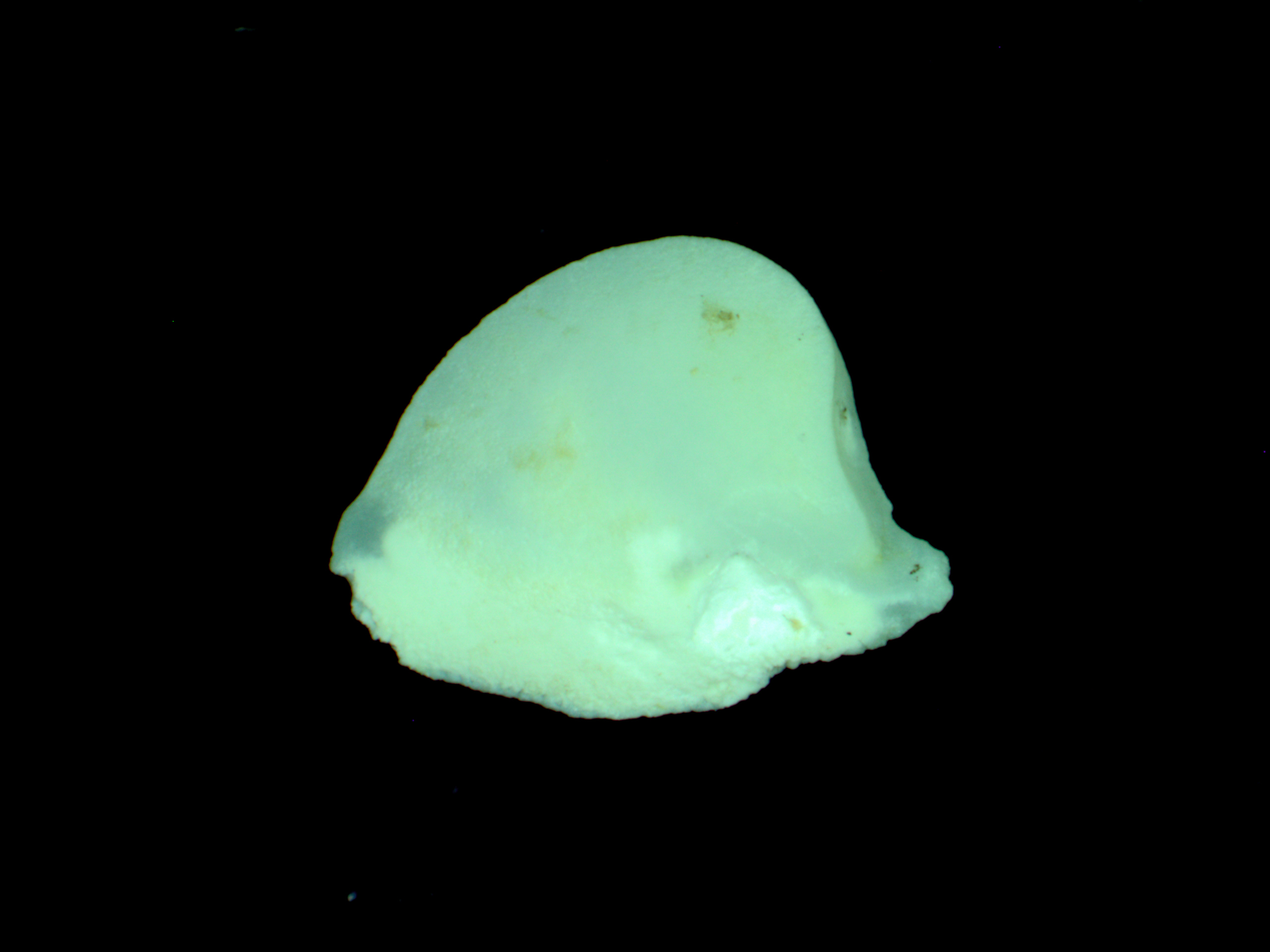

Supplement: Supplemental Information 7 [file peerj-04-1664-s007.zip › PliArg/training/ARI65_R1.jpg]

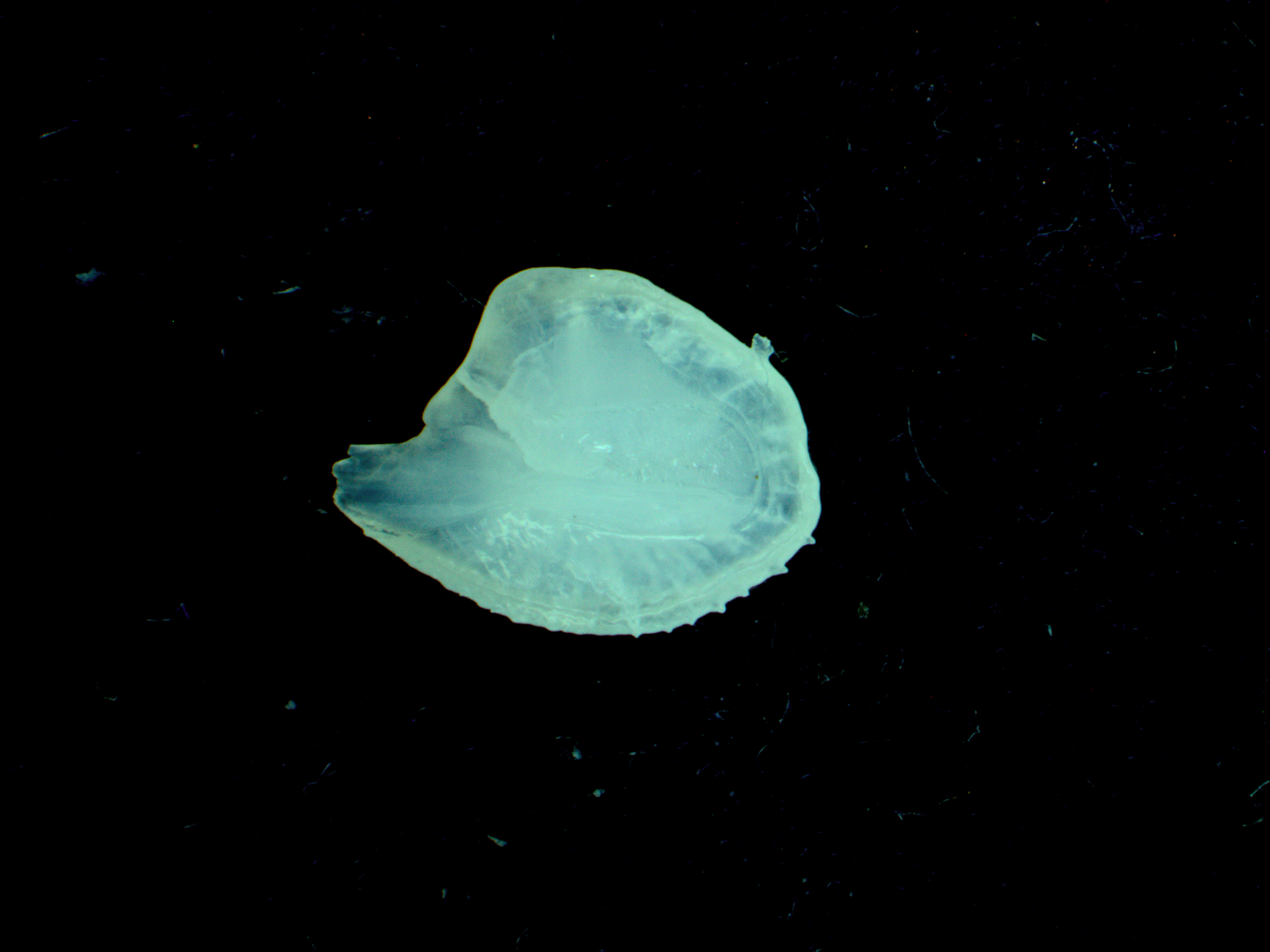

Supplement: Supplemental Information 8 [file peerj-04-1664-s008.zip › Coilia/testing/Eng185R1.jpg]

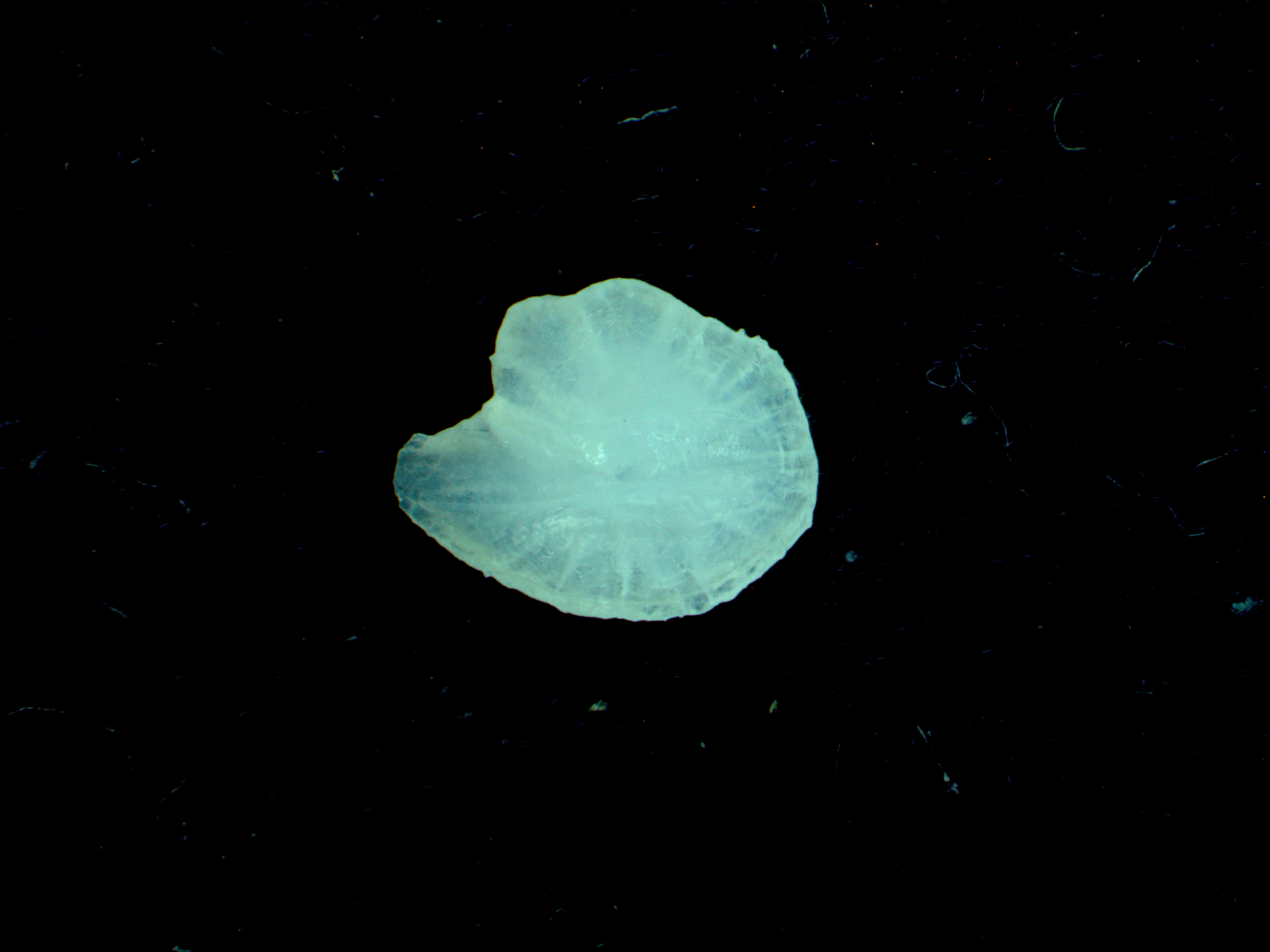

Supplement: Supplemental Information 8 [file peerj-04-1664-s008.zip › Coilia/testing/Eng186R1.jpg]

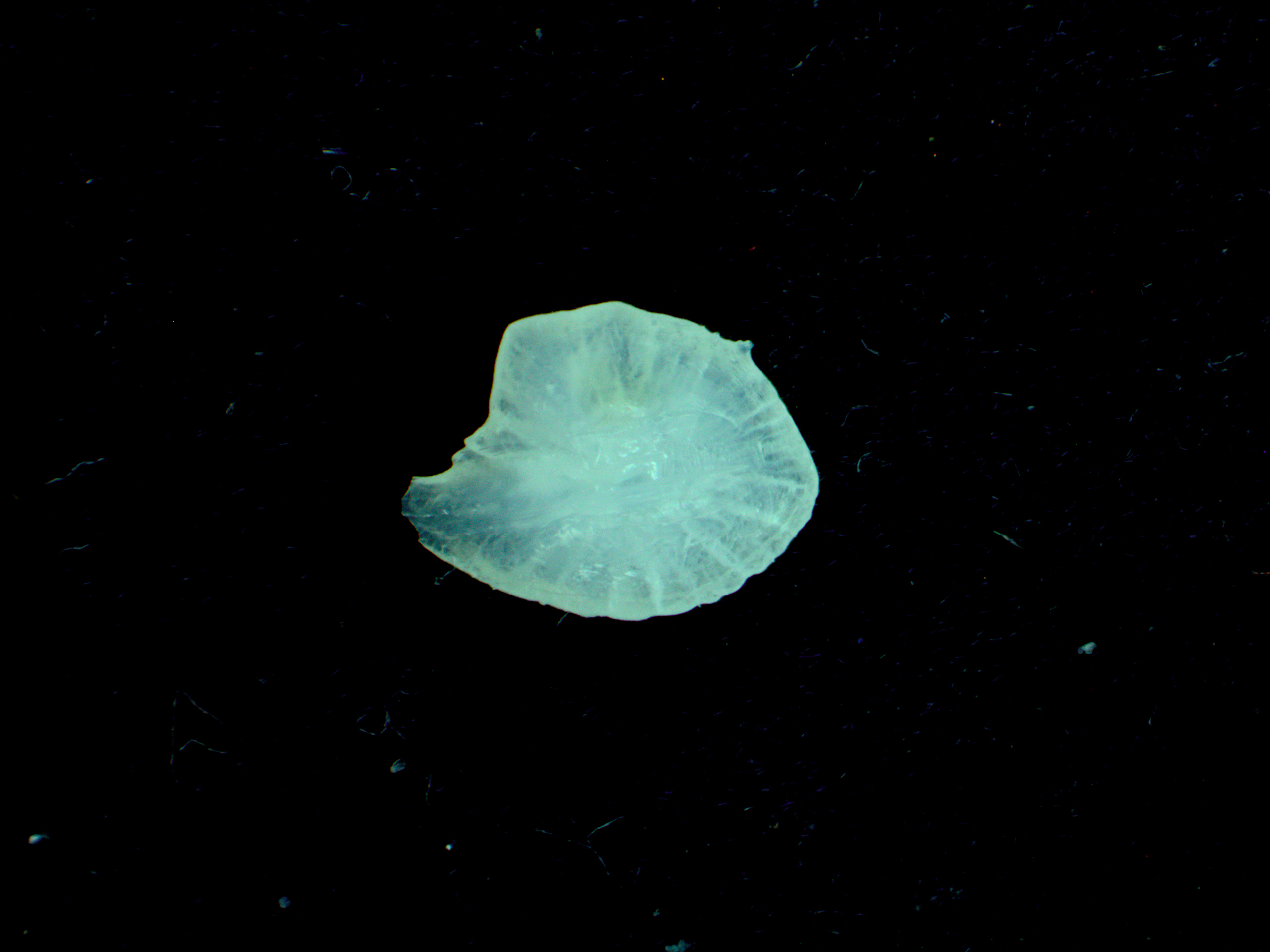

Supplement: Supplemental Information 8 [file peerj-04-1664-s008.zip › Coilia/testing/Eng187R1.jpg]

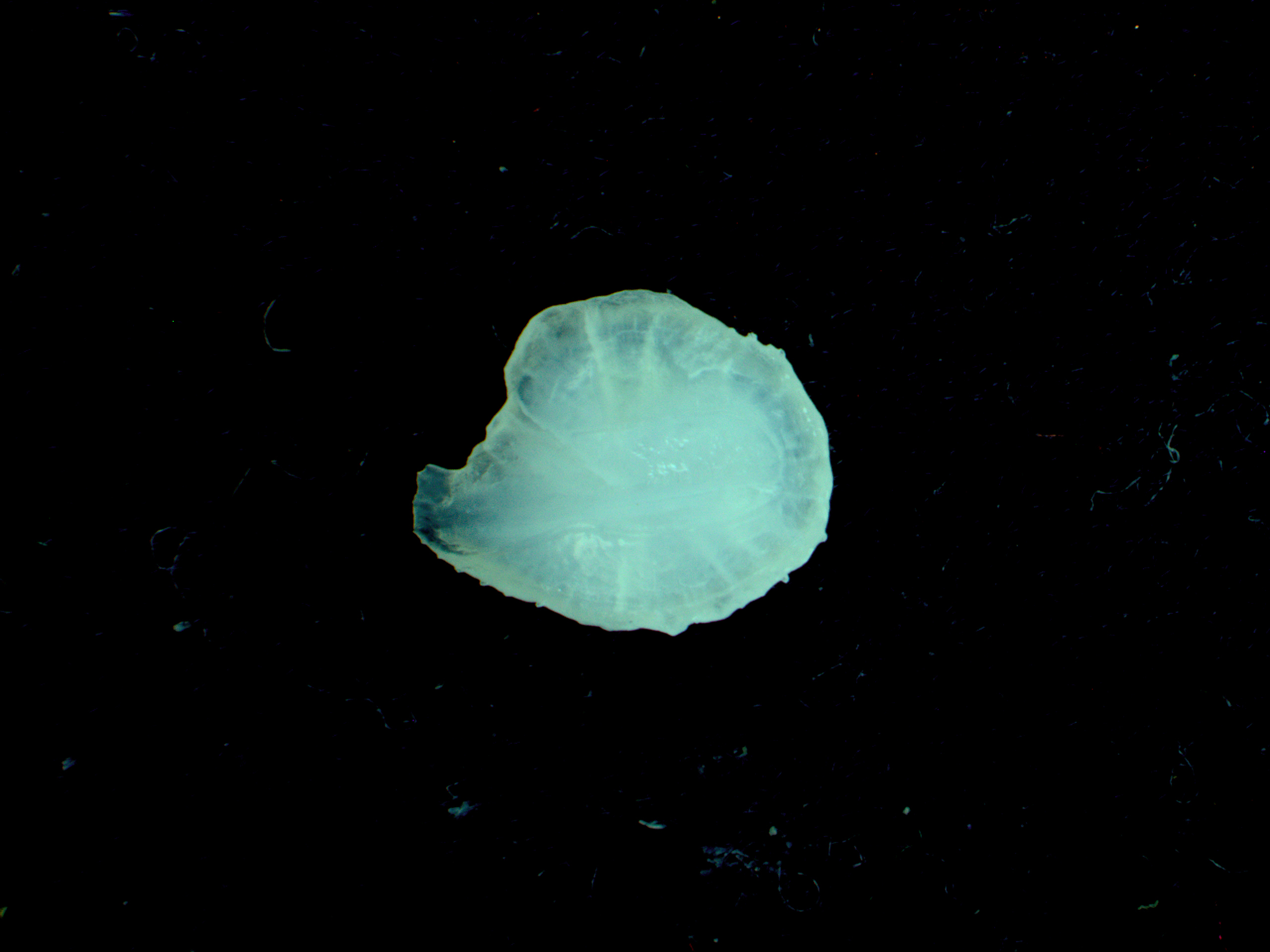

Supplement: Supplemental Information 8 [file peerj-04-1664-s008.zip › Coilia/testing/Eng188R1.jpg]

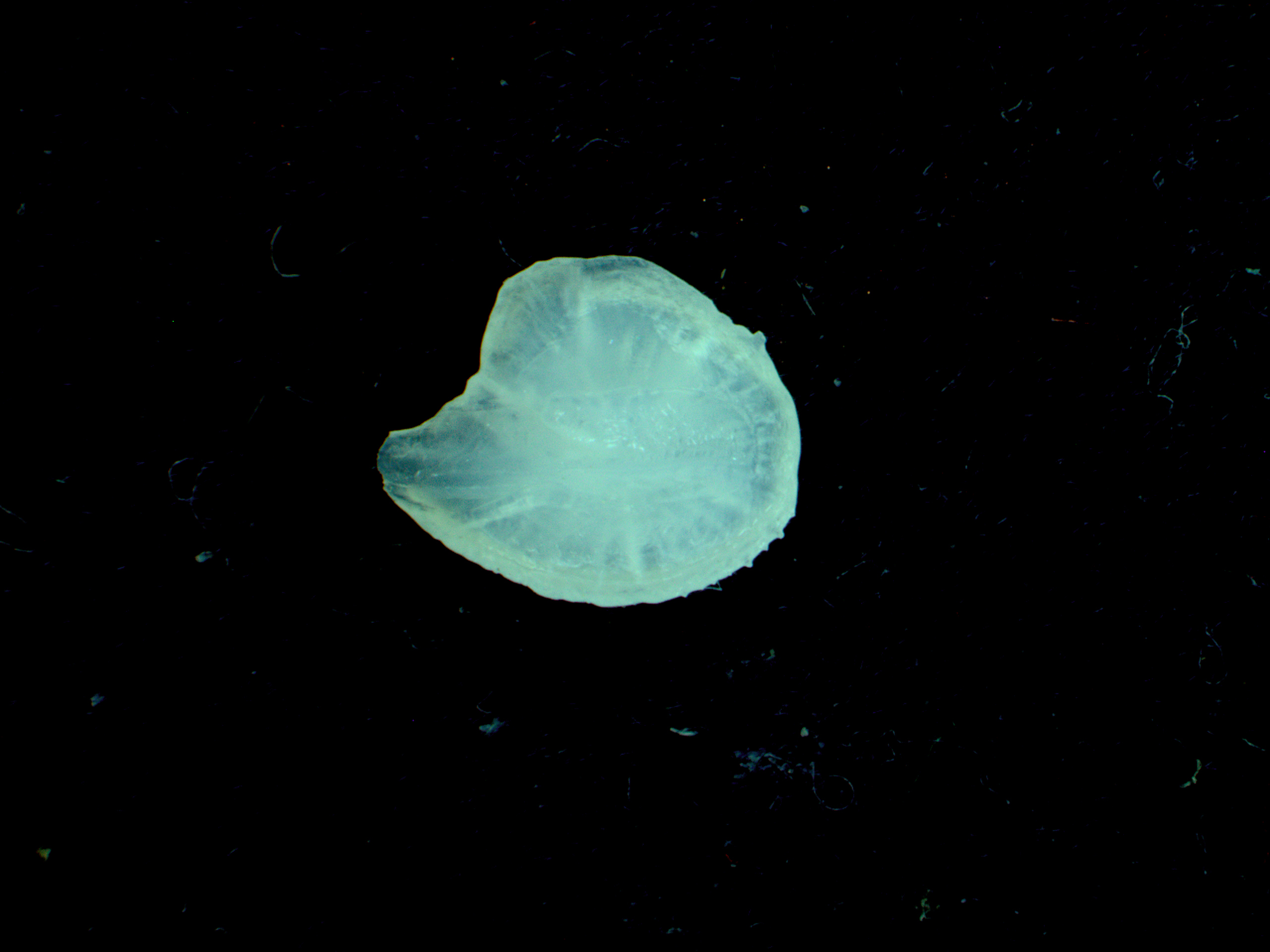

Supplement: Supplemental Information 8 [file peerj-04-1664-s008.zip › Coilia/testing/Eng189R1.jpg]

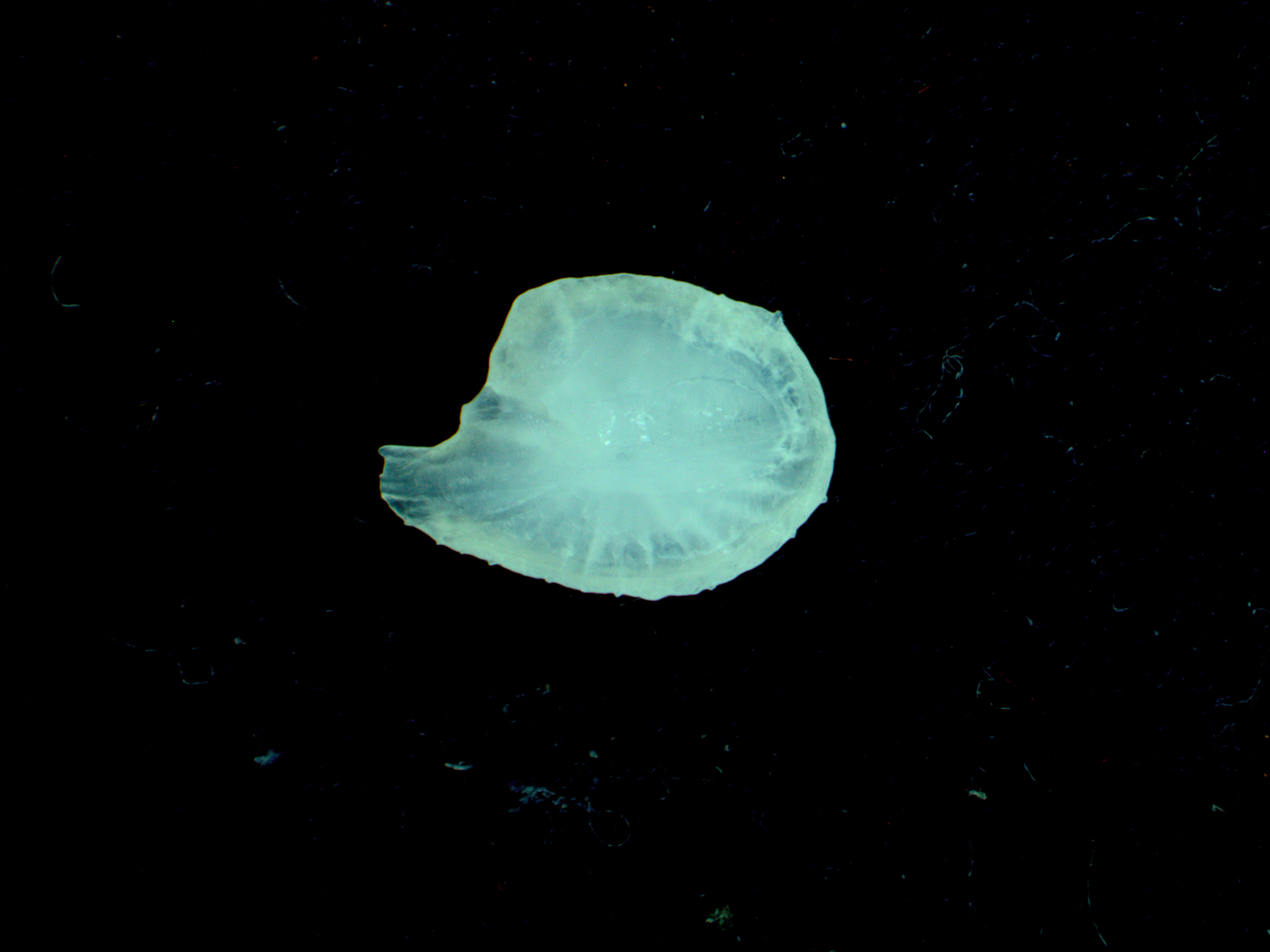

Supplement: Supplemental Information 8 [file peerj-04-1664-s008.zip › Coilia/testing/Eng190R1.jpg]

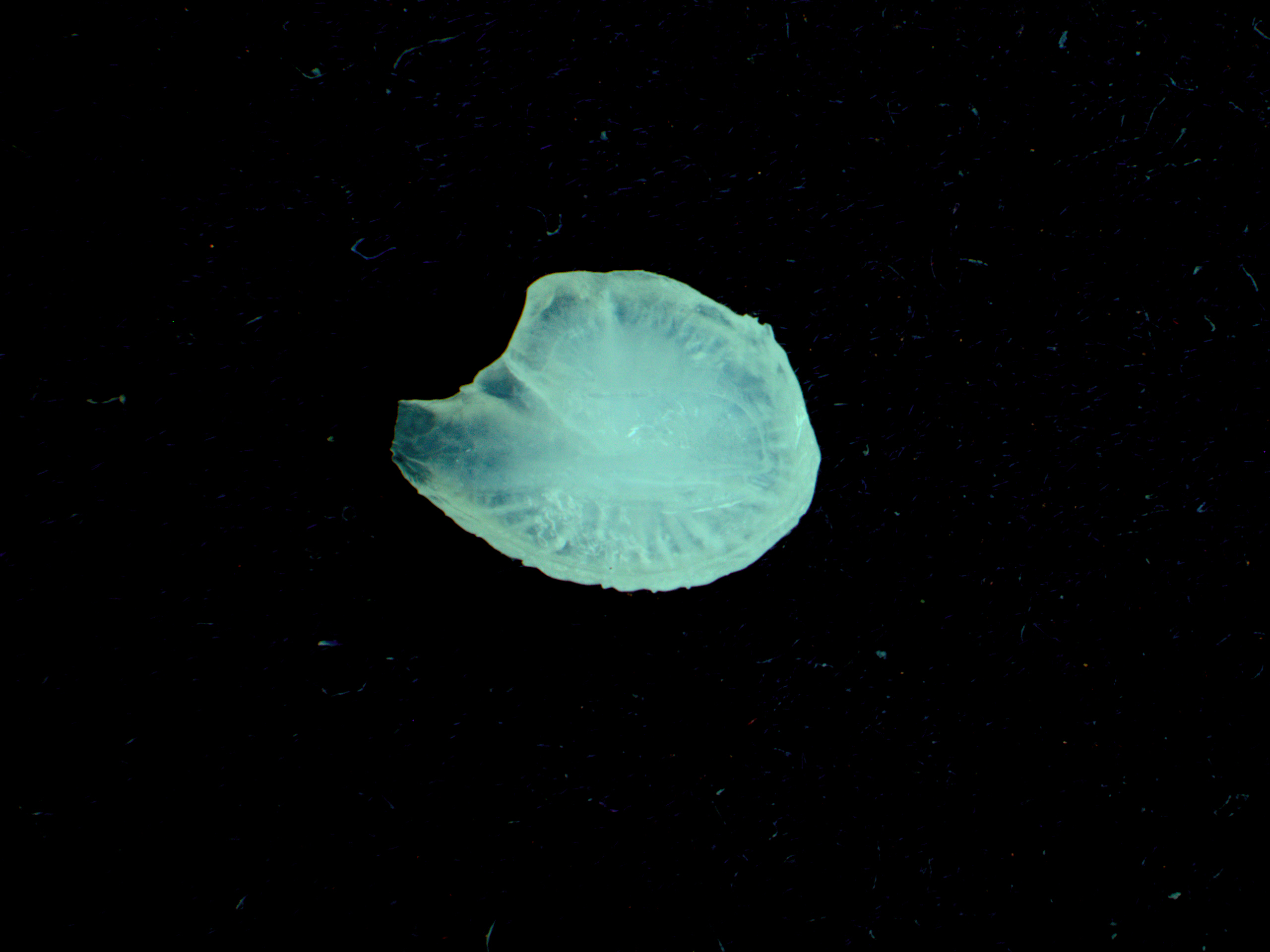

Supplement: Supplemental Information 8 [file peerj-04-1664-s008.zip › Coilia/testing/Eng191R1.jpg]

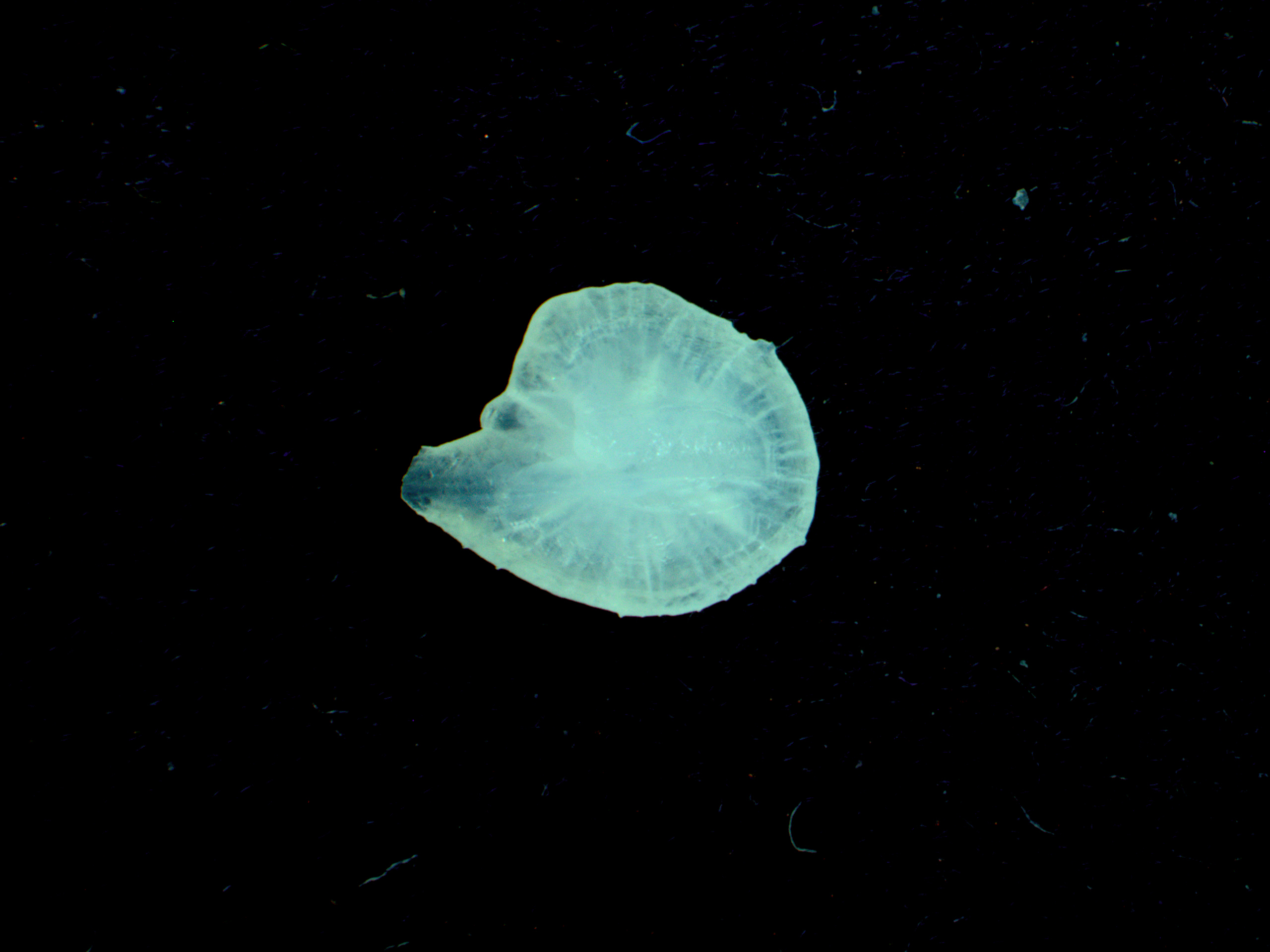

Supplement: Supplemental Information 8 [file peerj-04-1664-s008.zip › Coilia/testing/Eng192R1.jpg]

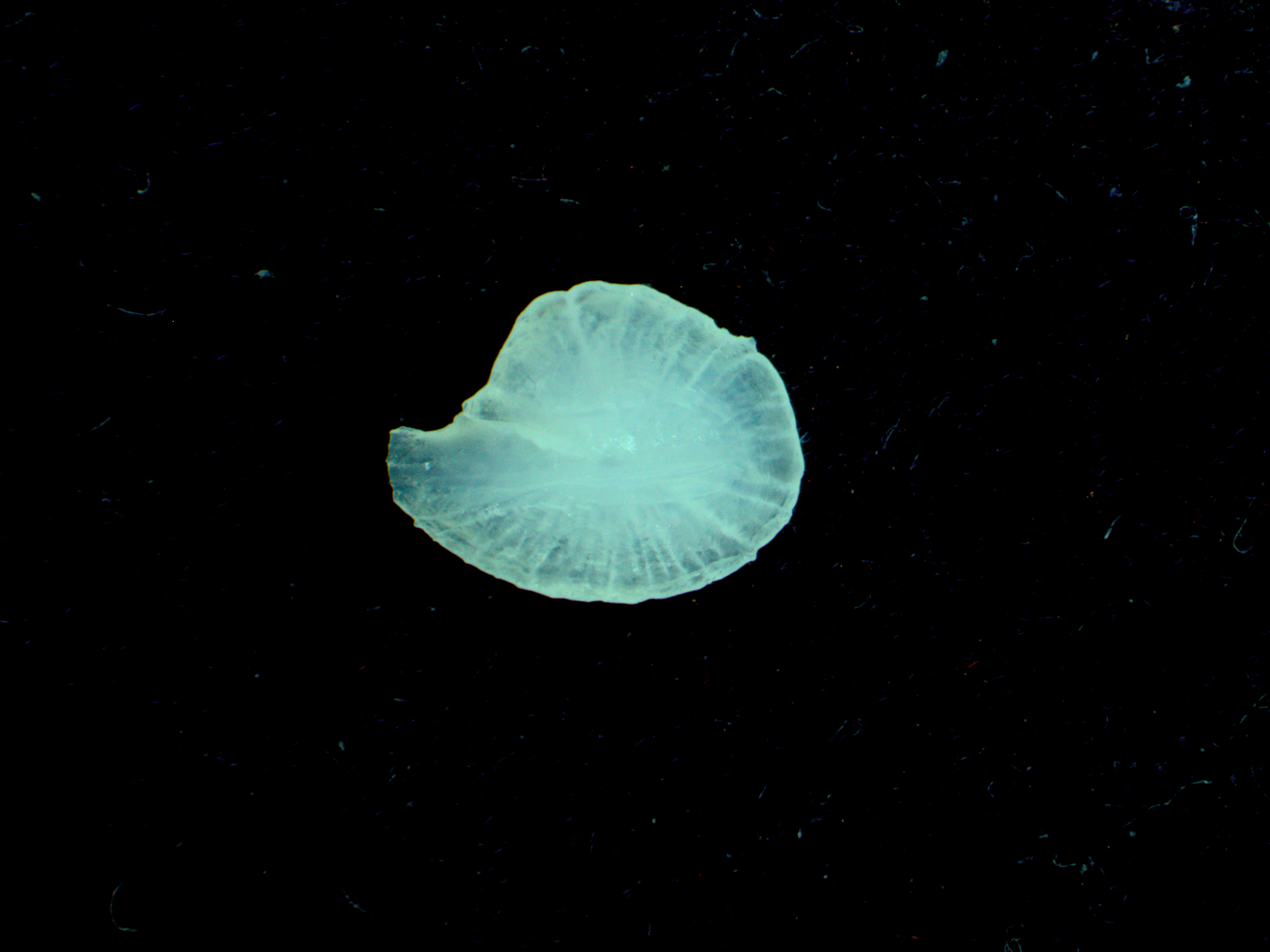

Supplement: Supplemental Information 8 [file peerj-04-1664-s008.zip › Coilia/testing/Eng193R1.jpg]

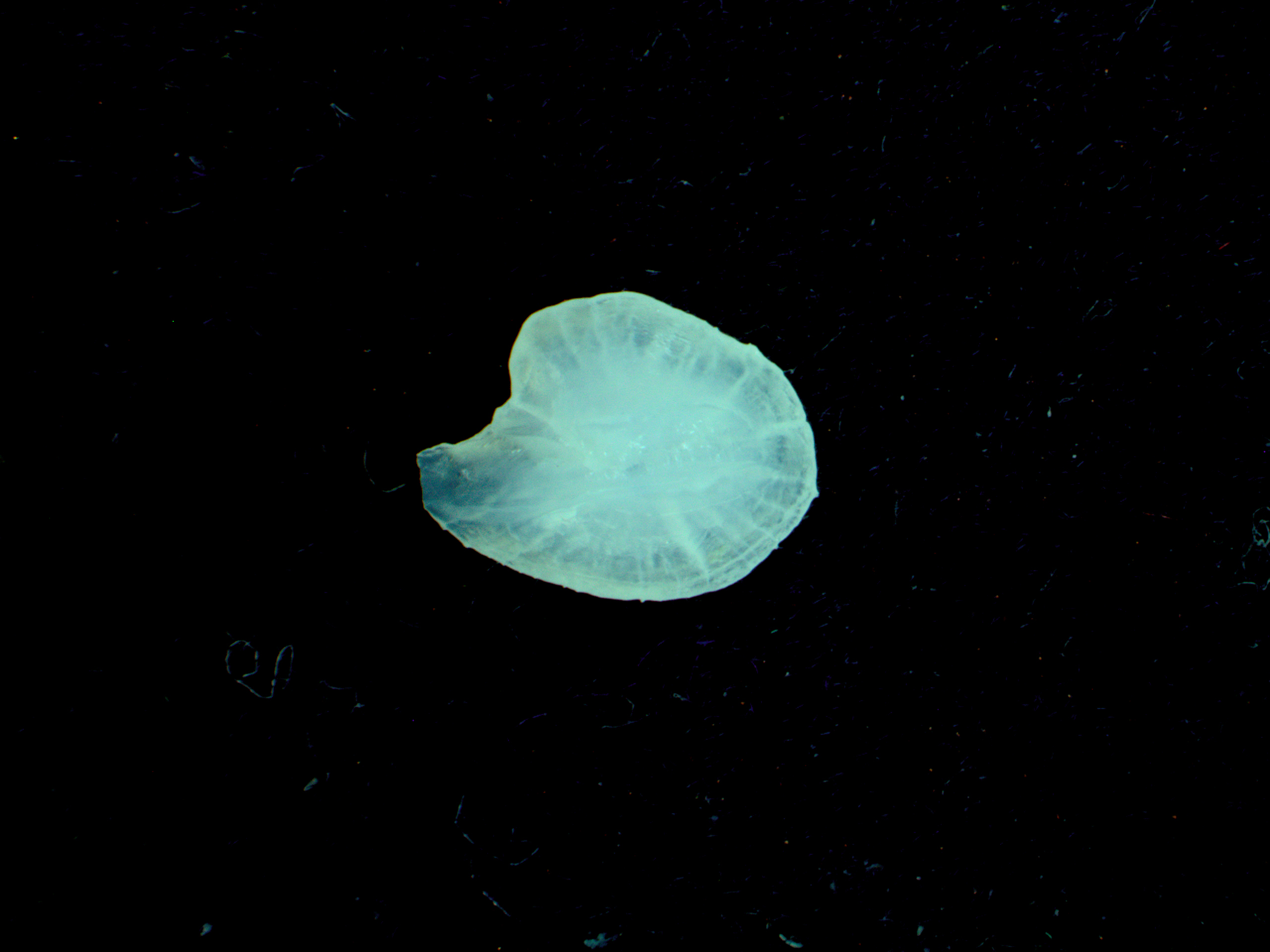

Supplement: Supplemental Information 8 [file peerj-04-1664-s008.zip › Coilia/testing/Eng194R1.jpg]

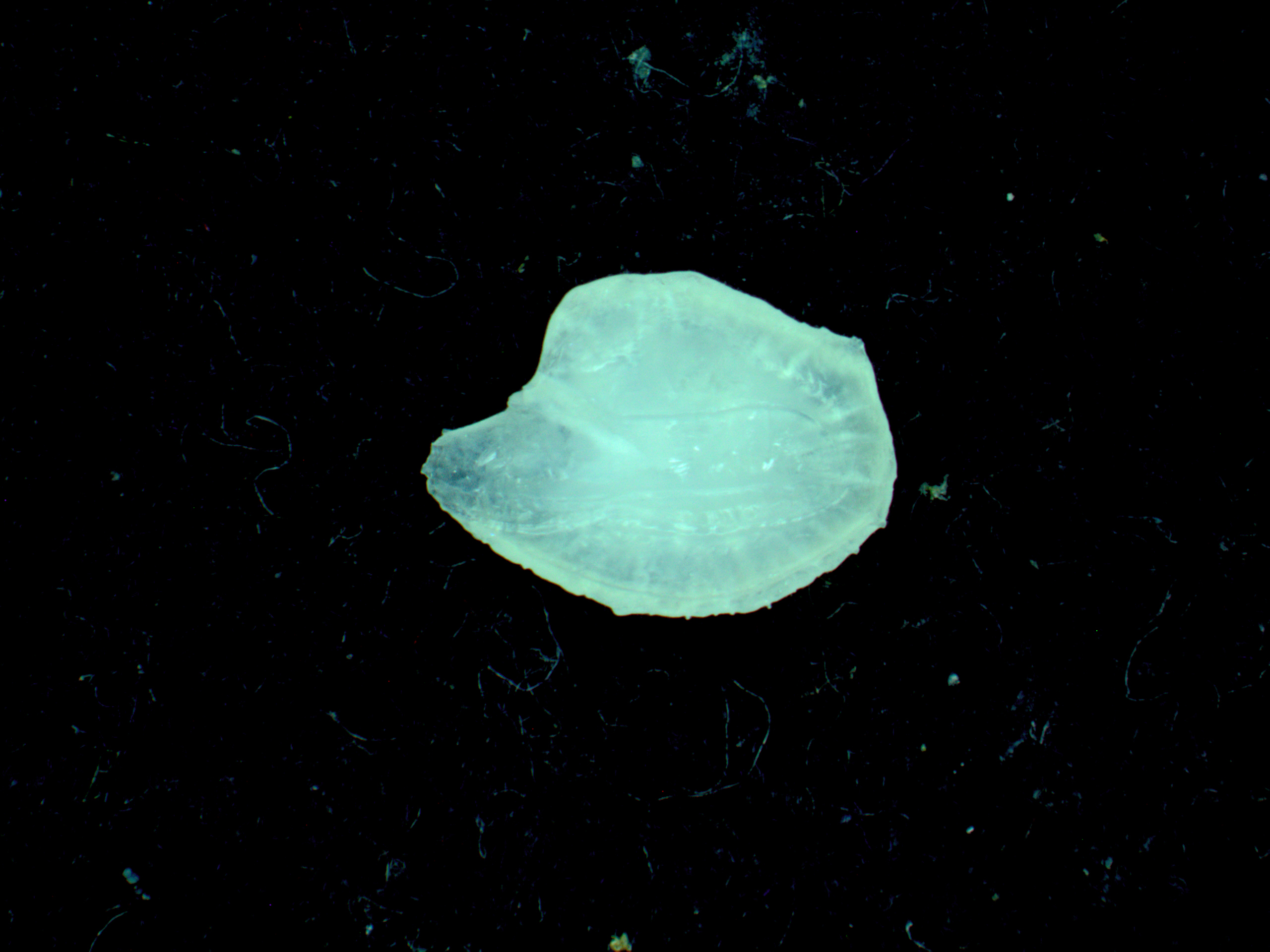

Supplement: Supplemental Information 8 [file peerj-04-1664-s008.zip › Coilia/training/Eng164R1.jpg]

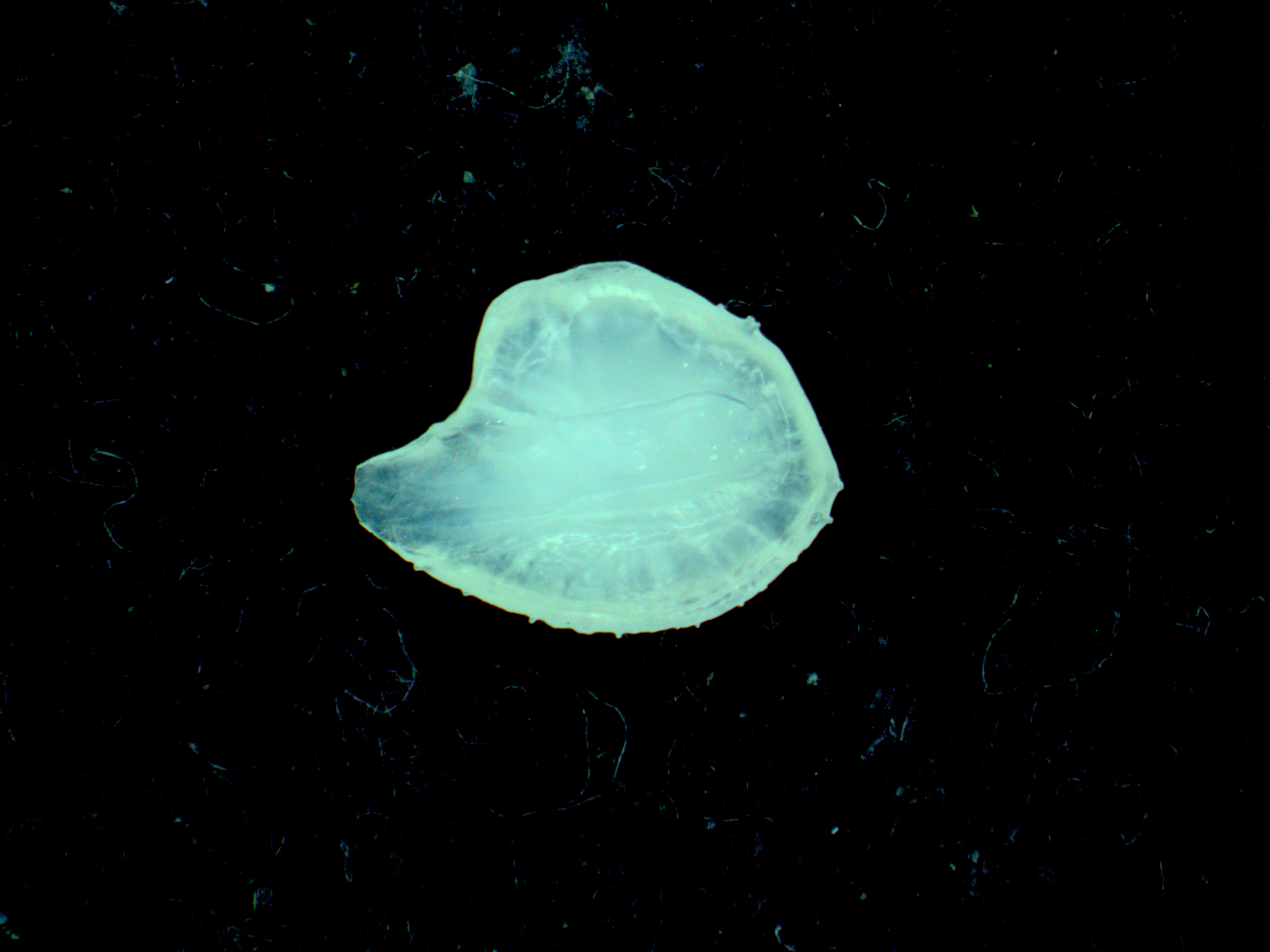

Supplement: Supplemental Information 8 [file peerj-04-1664-s008.zip › Coilia/training/Eng165R1.jpg]

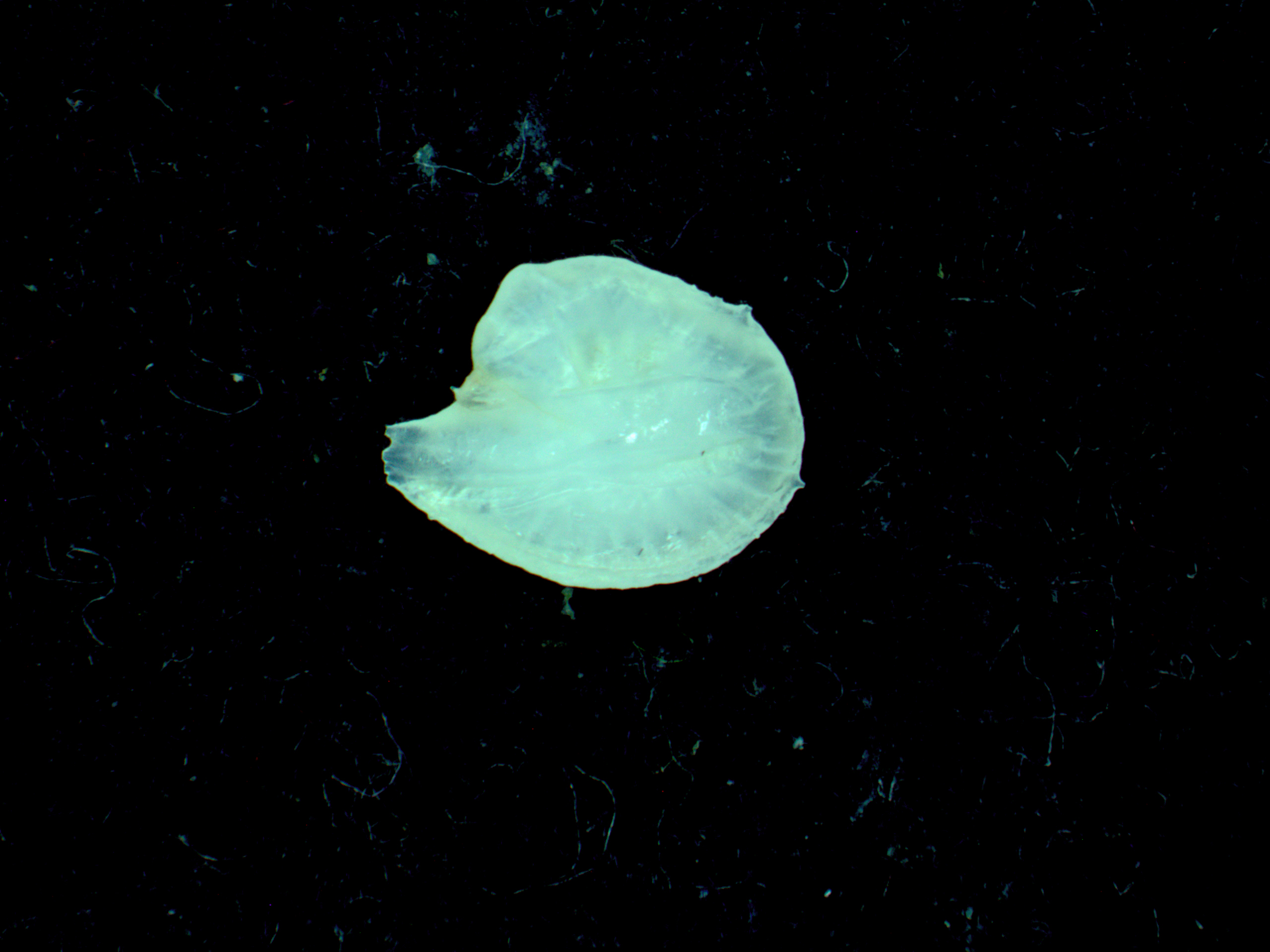

Supplement: Supplemental Information 8 [file peerj-04-1664-s008.zip › Coilia/training/Eng167R1.jpg]

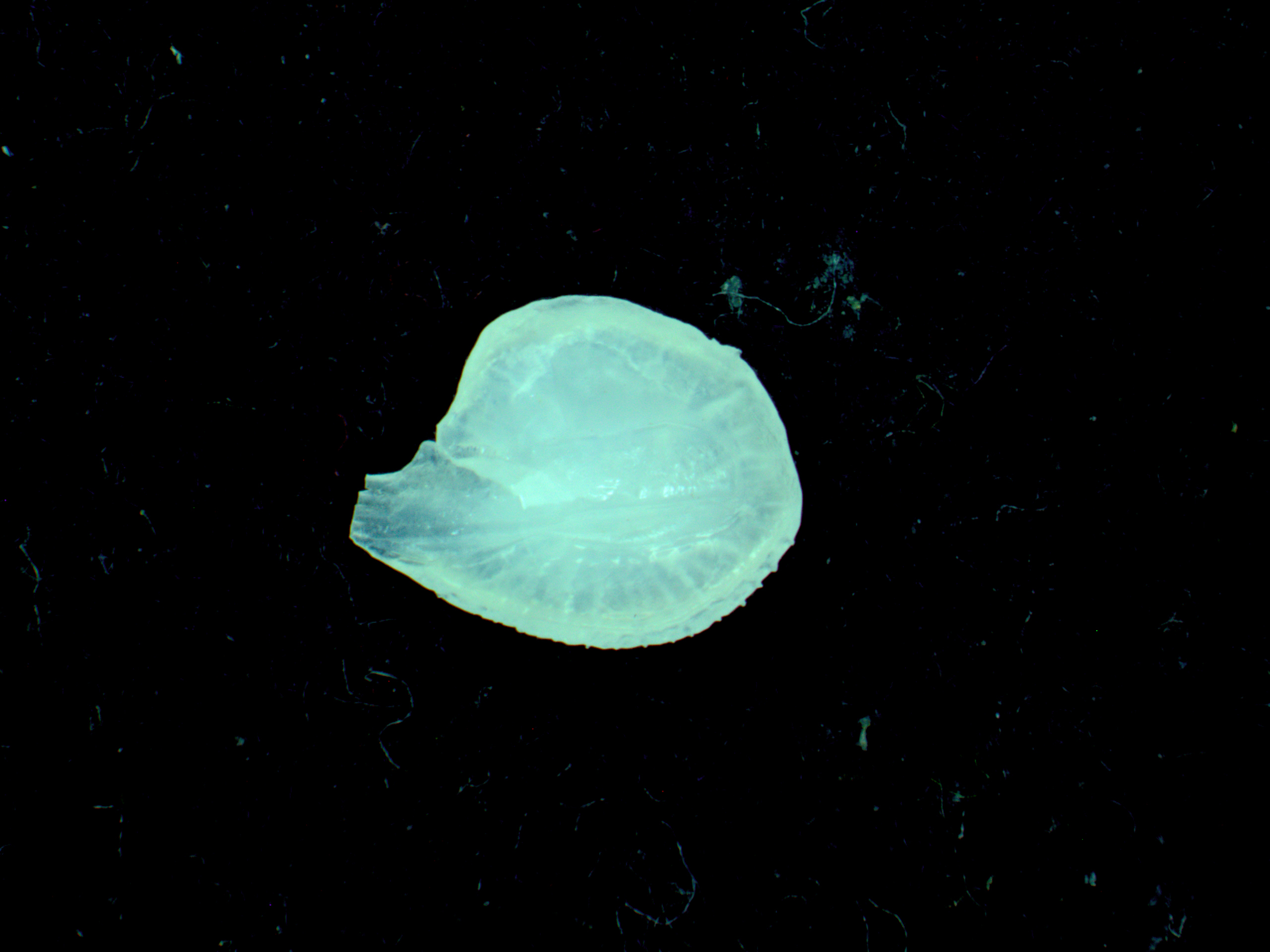

Supplement: Supplemental Information 8 [file peerj-04-1664-s008.zip › Coilia/training/Eng168R1.jpg]

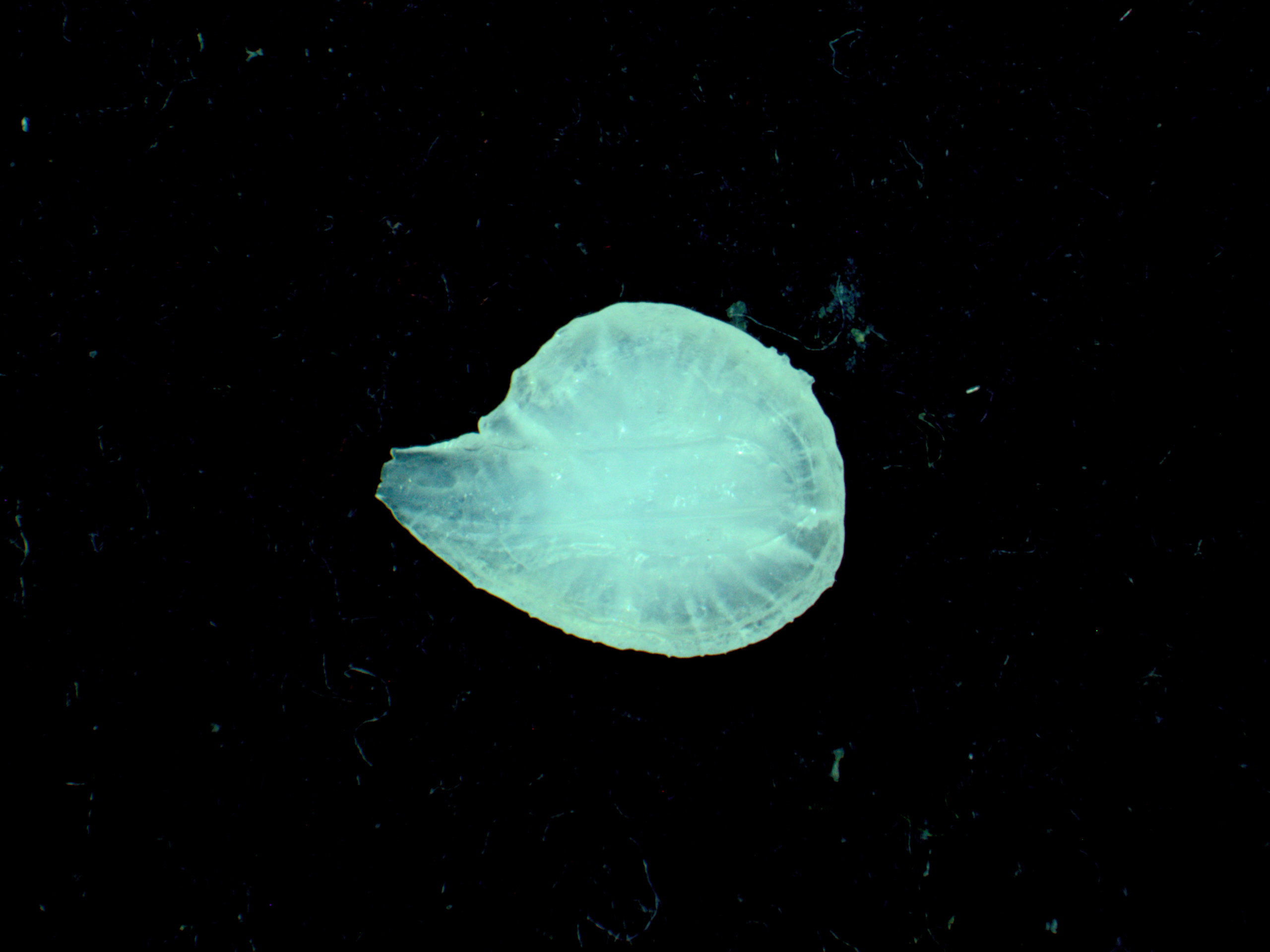

Supplement: Supplemental Information 8 [file peerj-04-1664-s008.zip › Coilia/training/Eng169R1.jpg]

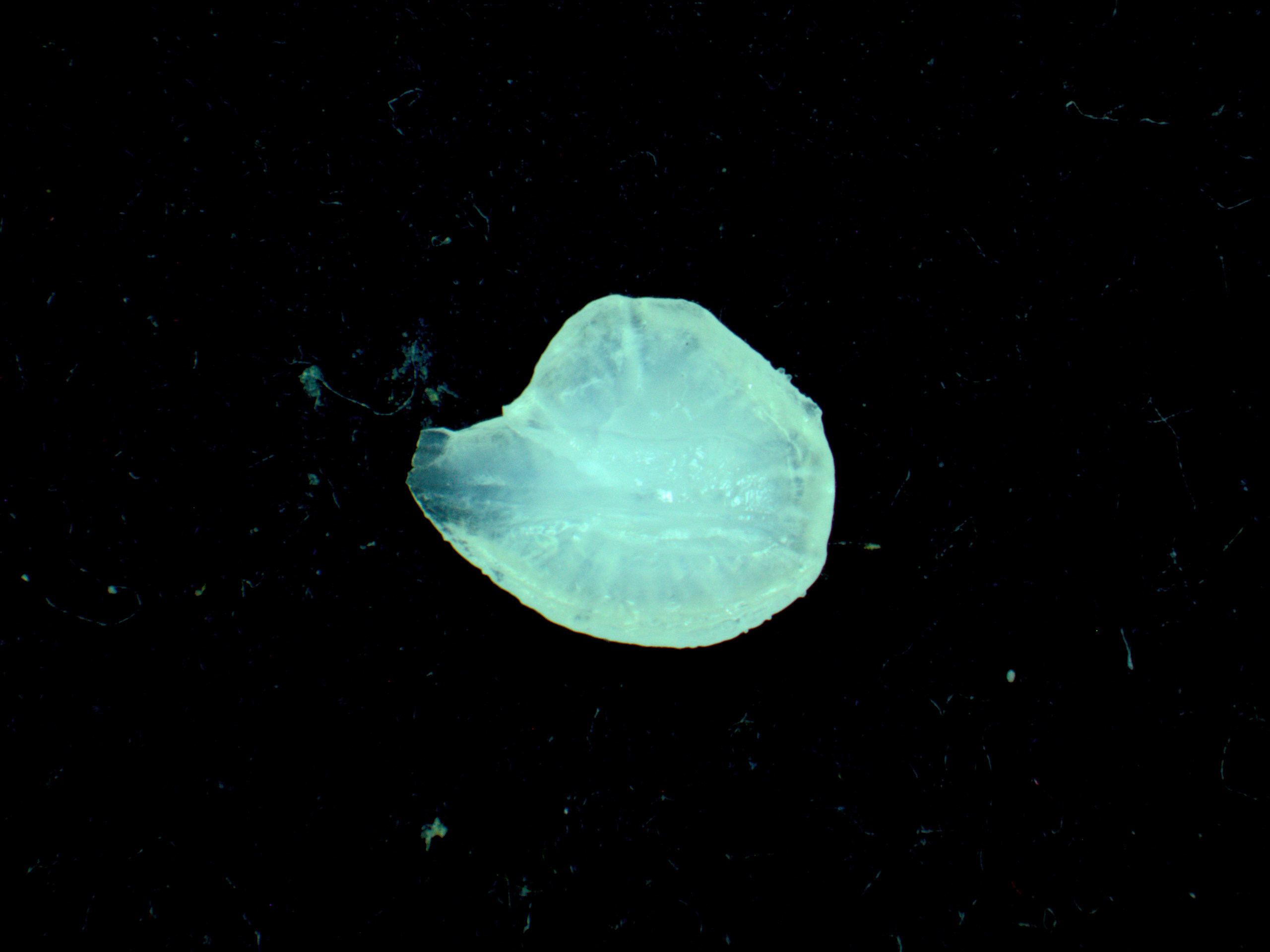

Supplement: Supplemental Information 8 [file peerj-04-1664-s008.zip › Coilia/training/Eng170R1.jpg]

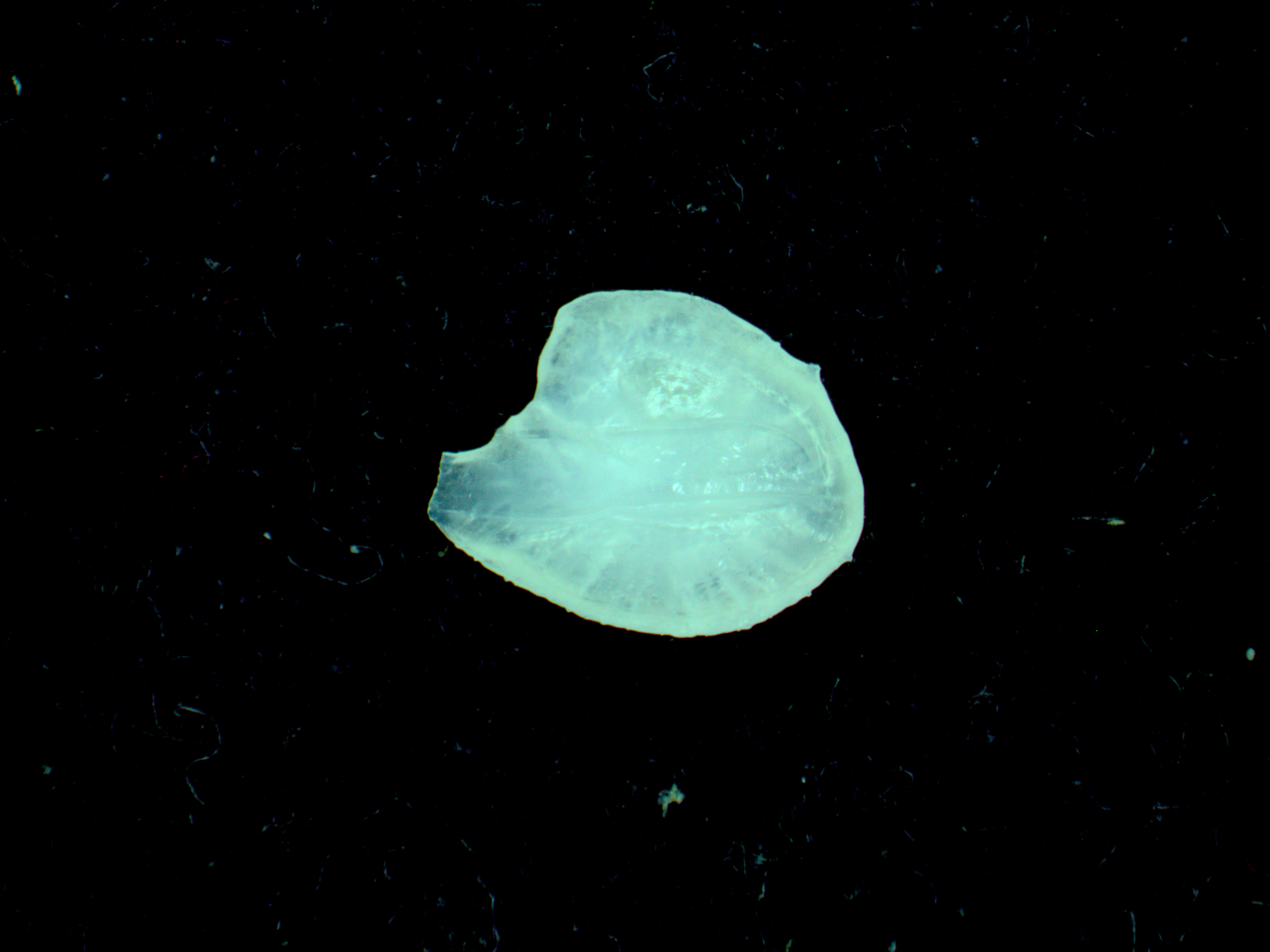

Supplement: Supplemental Information 8 [file peerj-04-1664-s008.zip › Coilia/training/Eng171R1.jpg]

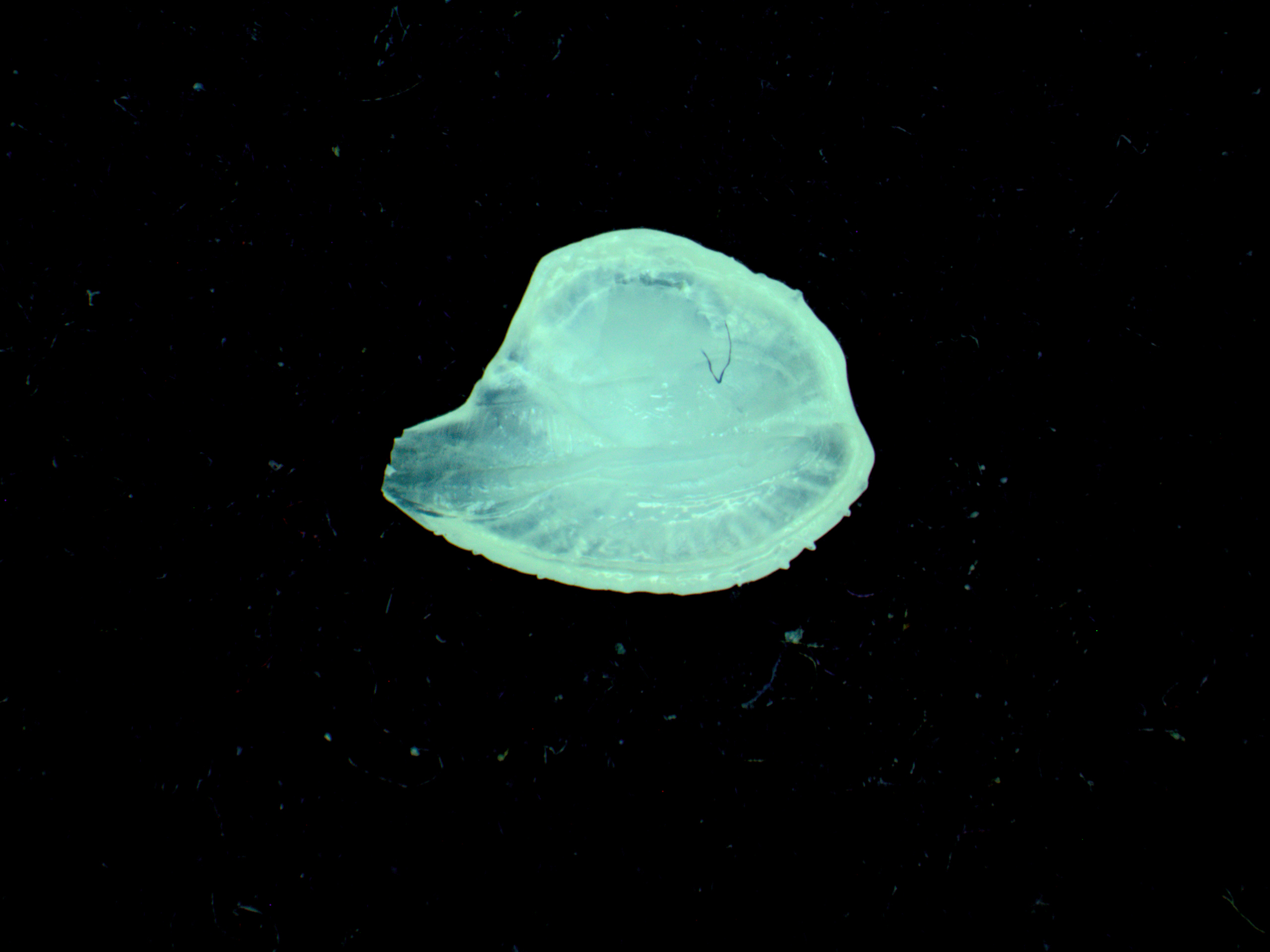

Supplement: Supplemental Information 8 [file peerj-04-1664-s008.zip › Coilia/training/Eng172R1.jpg]

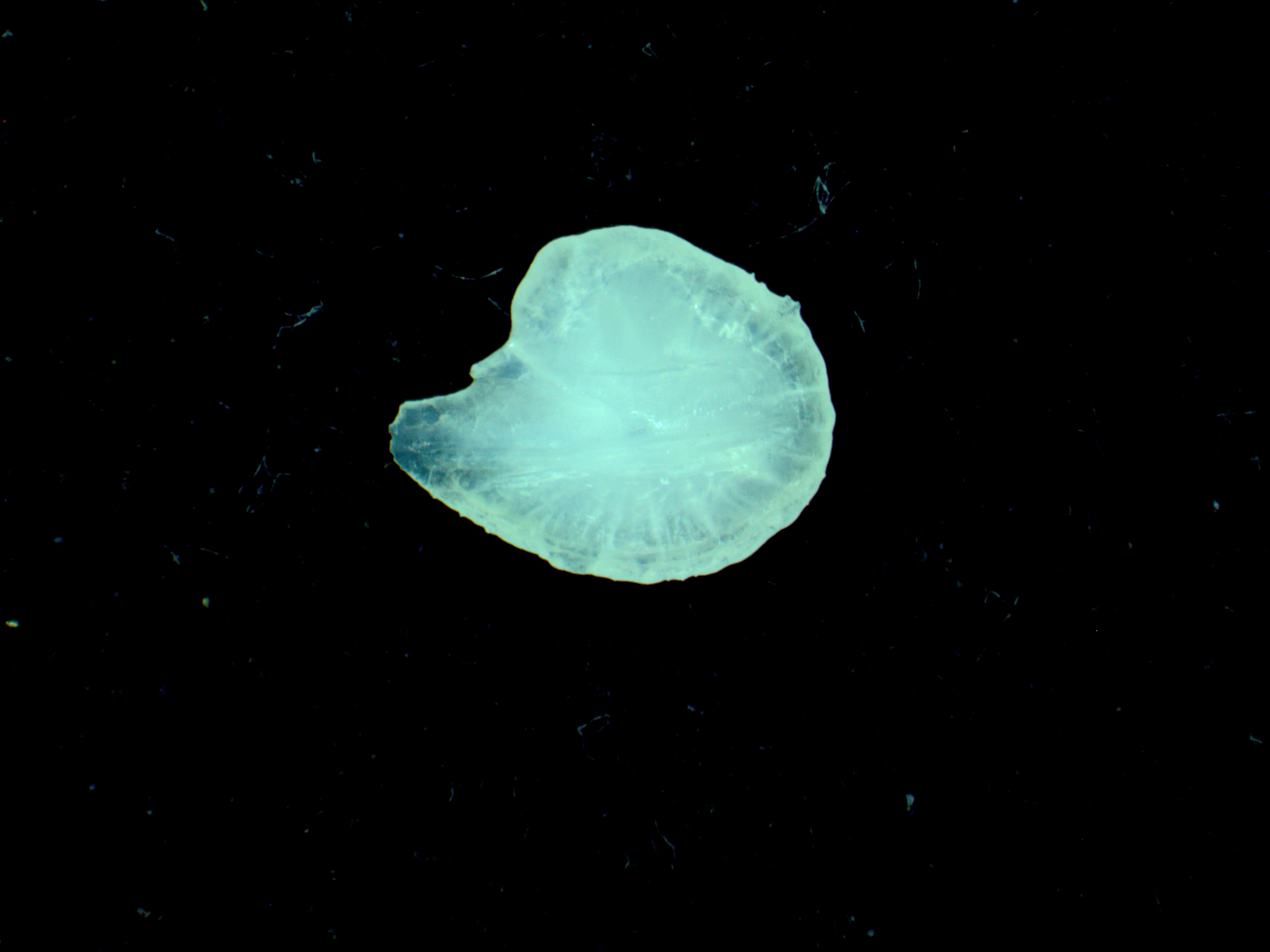

Supplement: Supplemental Information 8 [file peerj-04-1664-s008.zip › Coilia/training/Eng173R1.jpg]

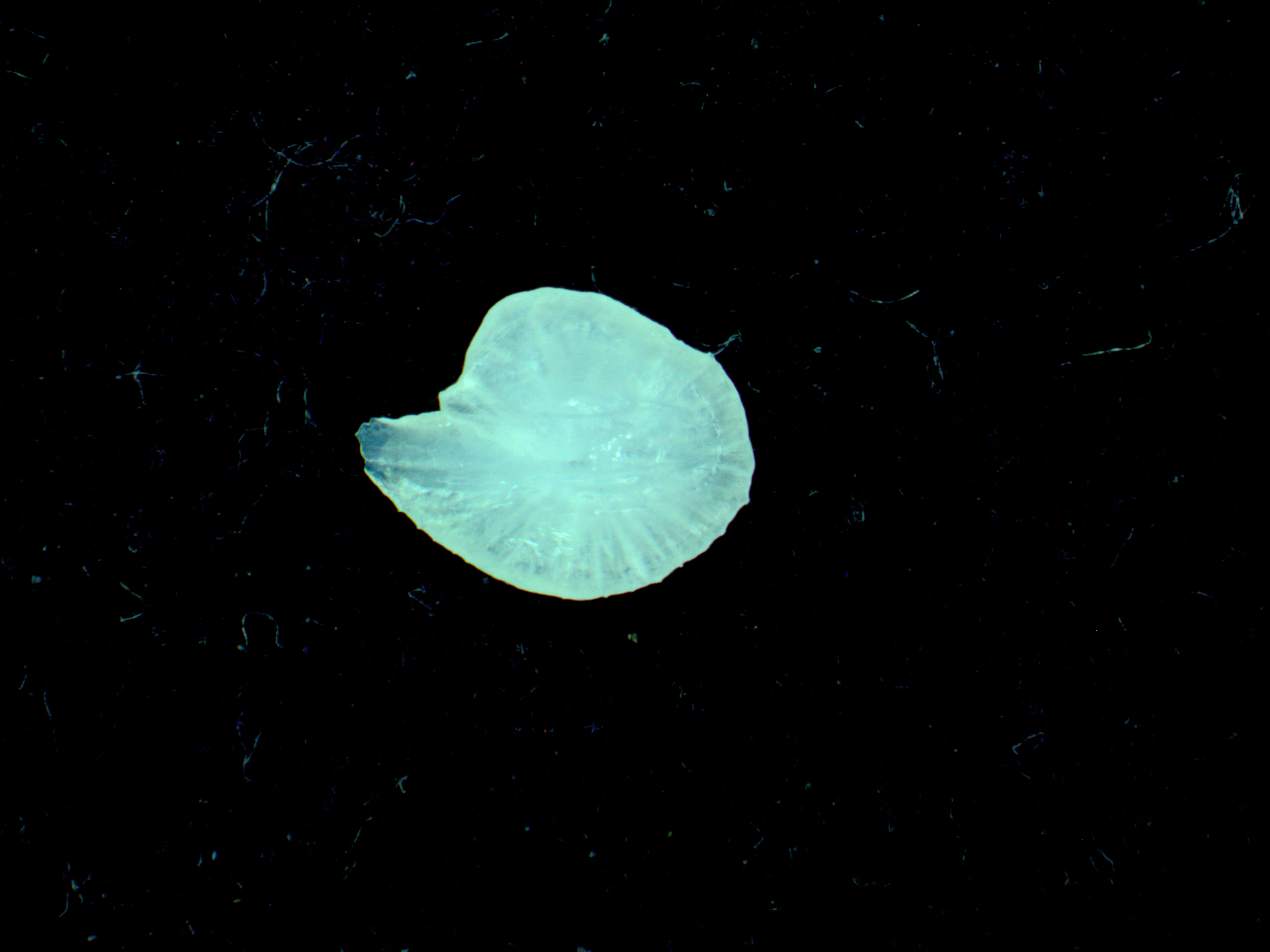

Supplement: Supplemental Information 8 [file peerj-04-1664-s008.zip › Coilia/training/Eng174R1.jpg]

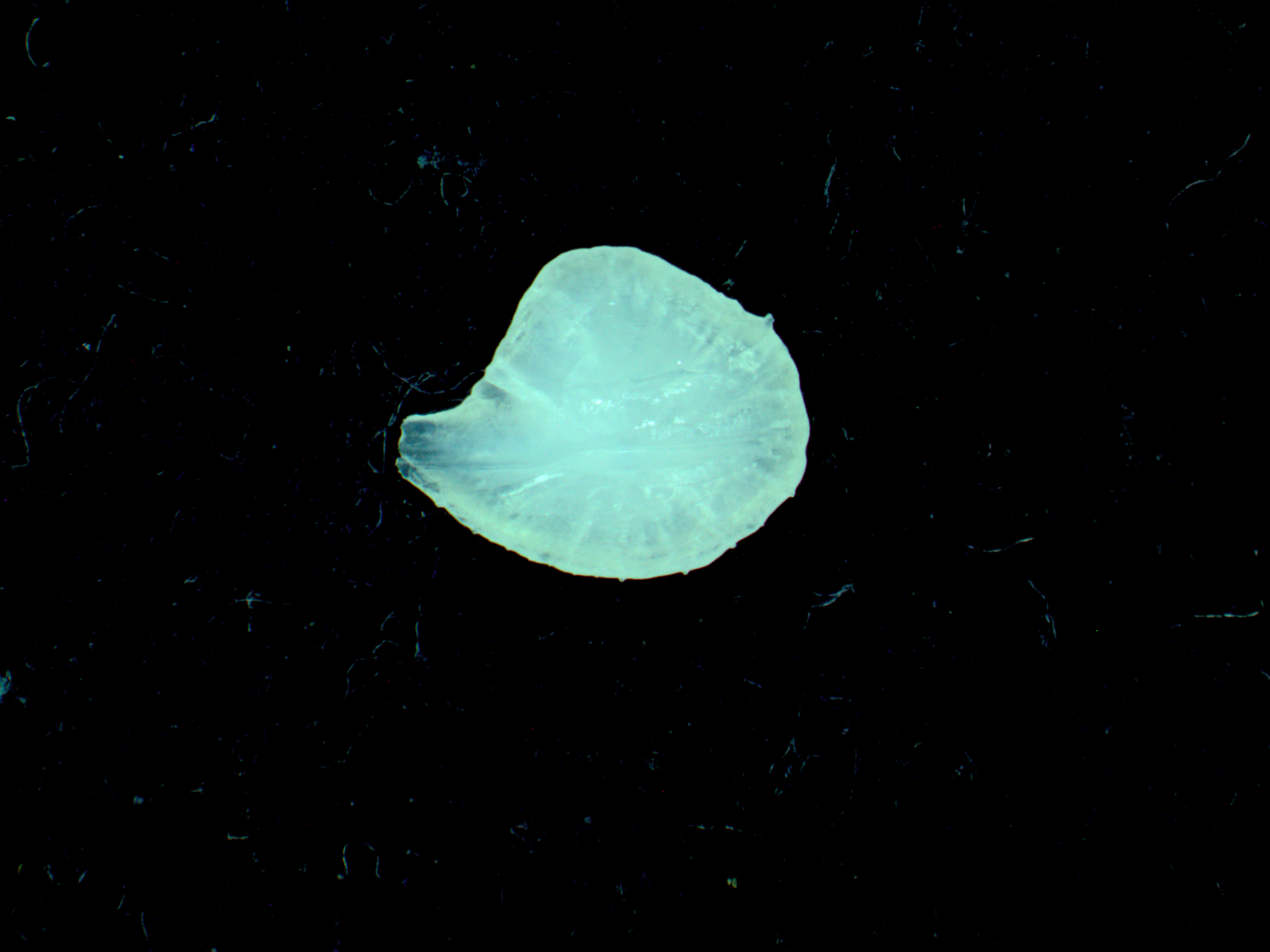

Supplement: Supplemental Information 8 [file peerj-04-1664-s008.zip › Coilia/training/Eng175R1.jpg]

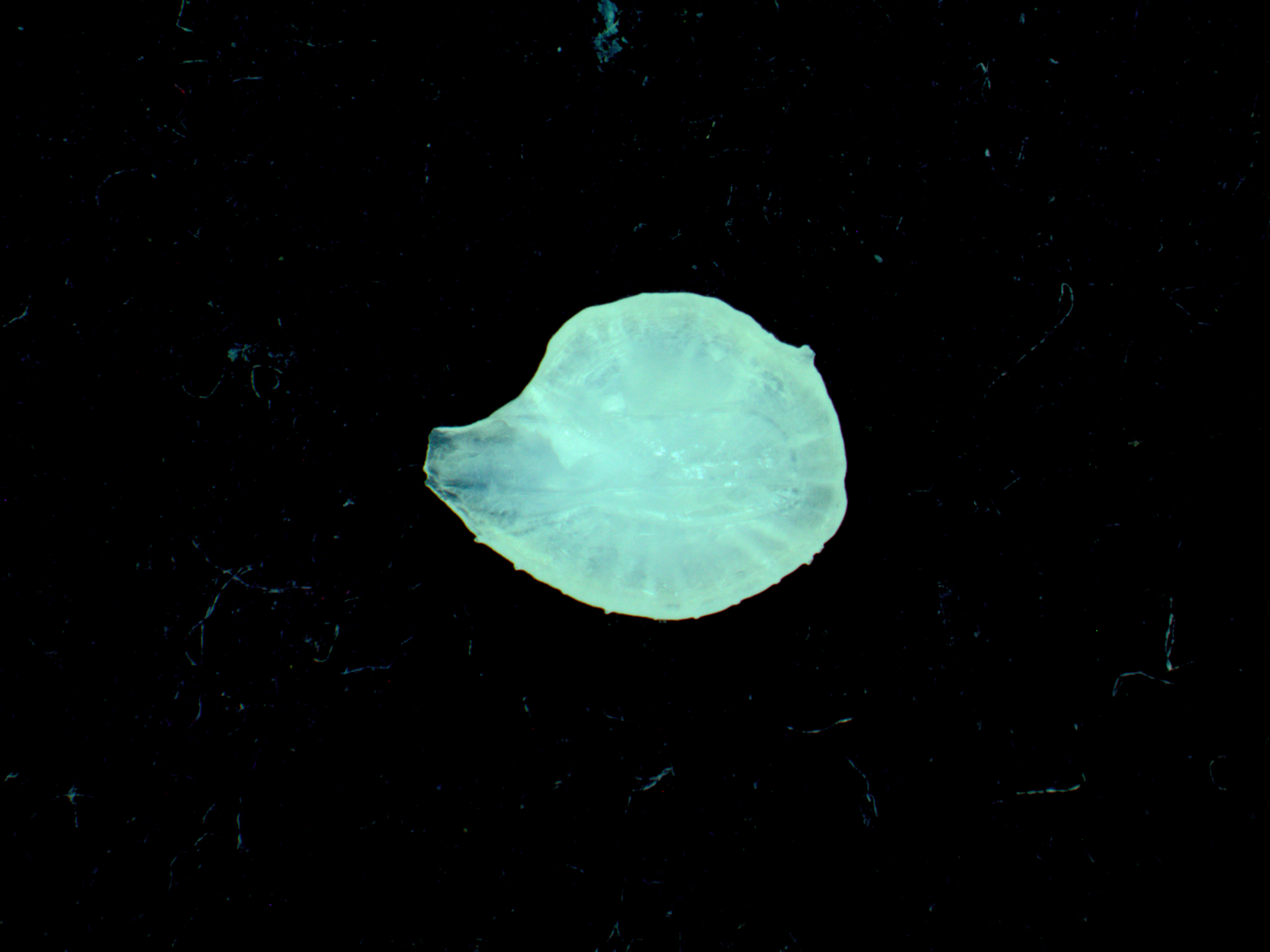

Supplement: Supplemental Information 8 [file peerj-04-1664-s008.zip › Coilia/training/Eng176R1.jpg]

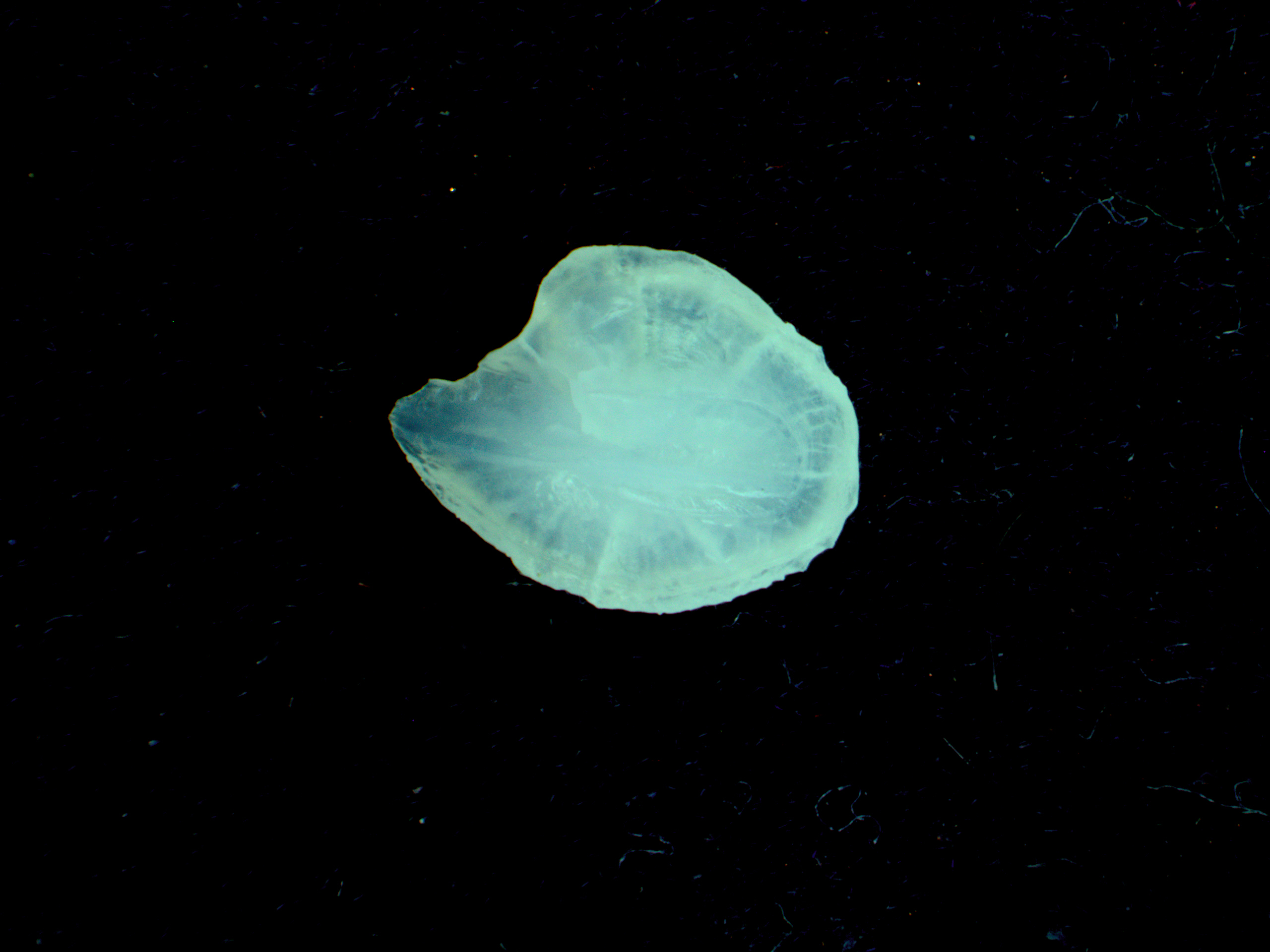

Supplement: Supplemental Information 8 [file peerj-04-1664-s008.zip › Coilia/training/Eng177R1.jpg]

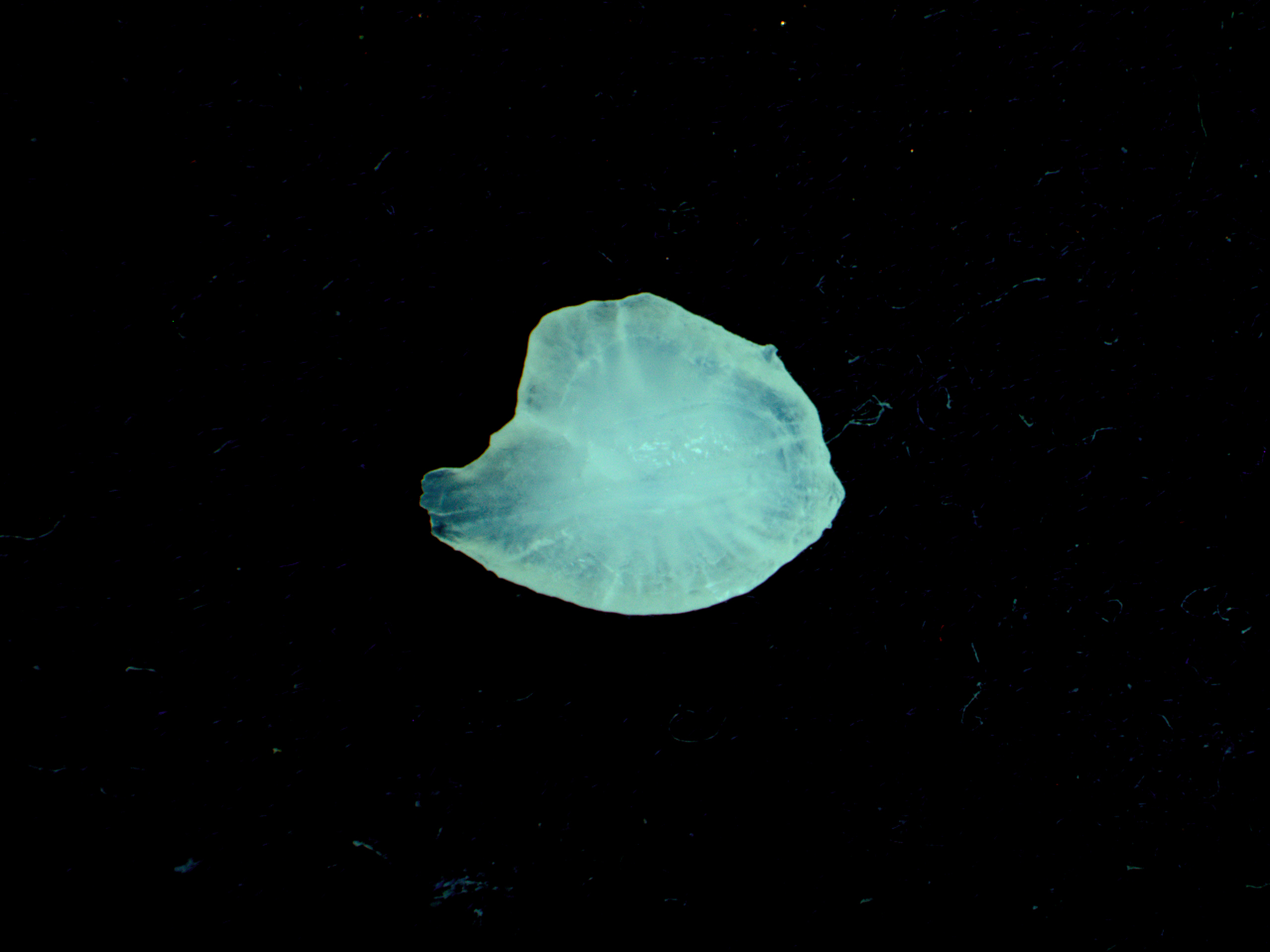

Supplement: Supplemental Information 8 [file peerj-04-1664-s008.zip › Coilia/training/Eng178R1.jpg]

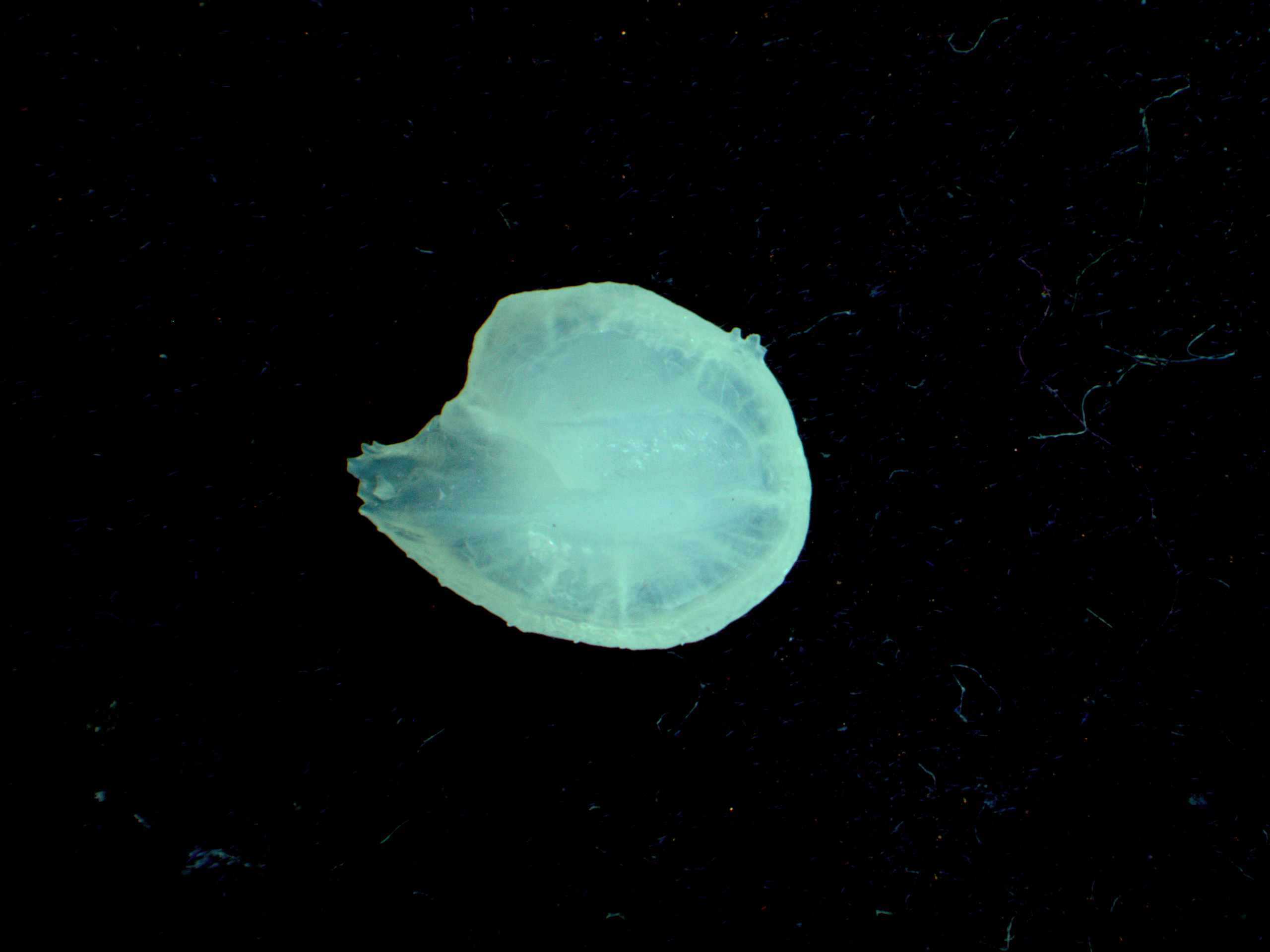

Supplement: Supplemental Information 8 [file peerj-04-1664-s008.zip › Coilia/training/Eng179R1.jpg]

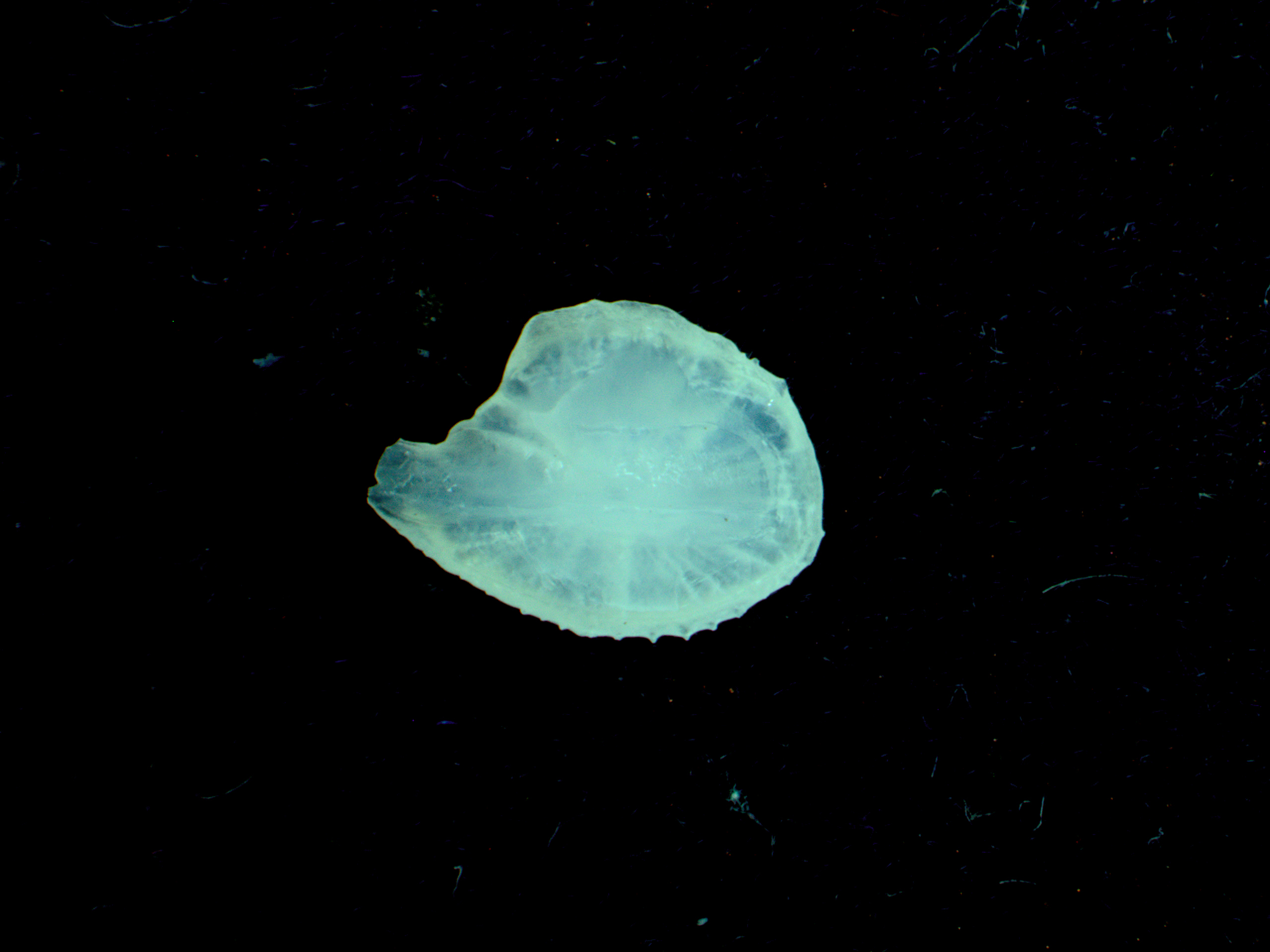

Supplement: Supplemental Information 8 [file peerj-04-1664-s008.zip › Coilia/training/Eng180R1.jpg]

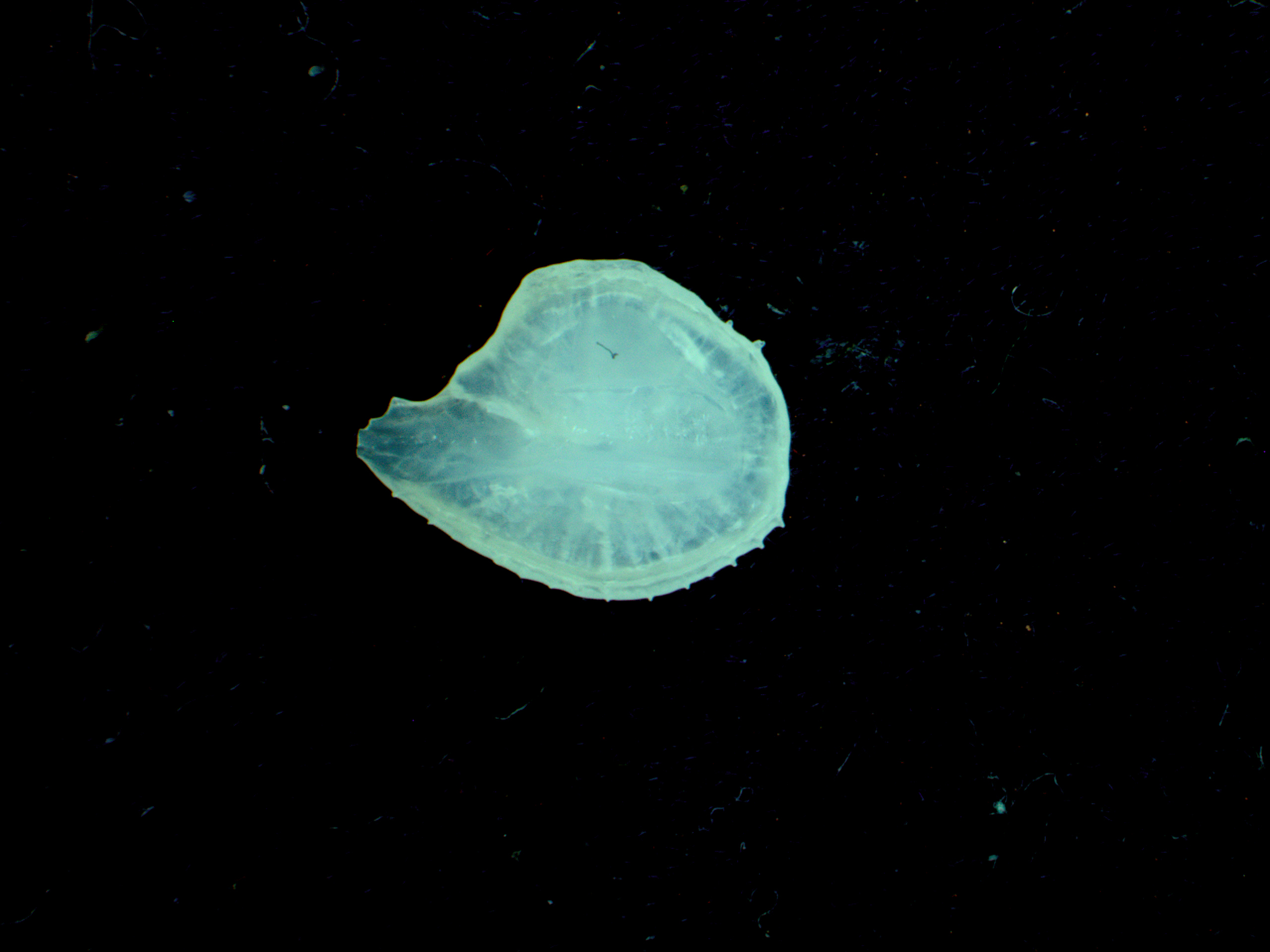

Supplement: Supplemental Information 8 [file peerj-04-1664-s008.zip › Coilia/training/Eng181R1.jpg]

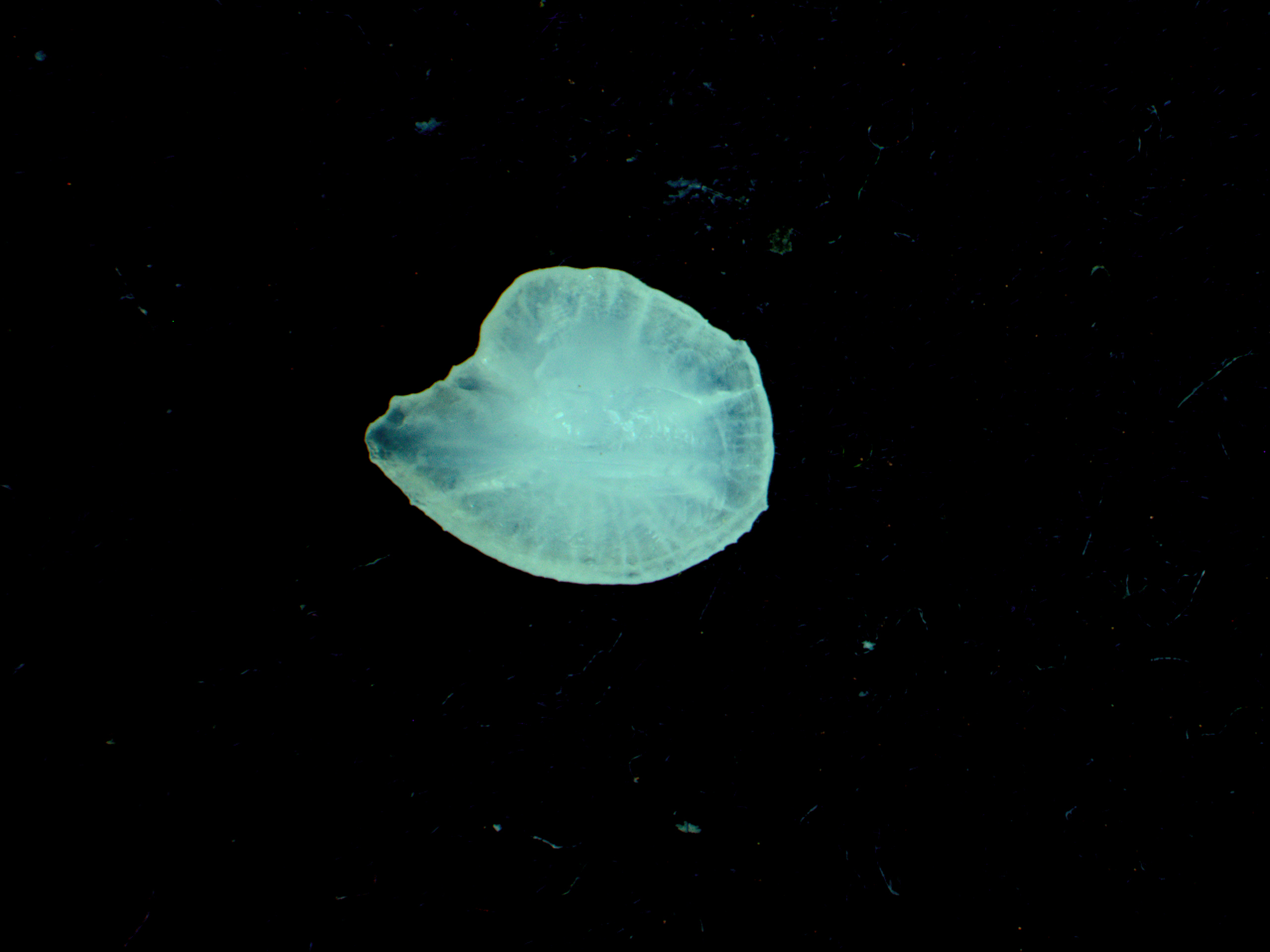

Supplement: Supplemental Information 8 [file peerj-04-1664-s008.zip › Coilia/training/Eng182R1.jpg]

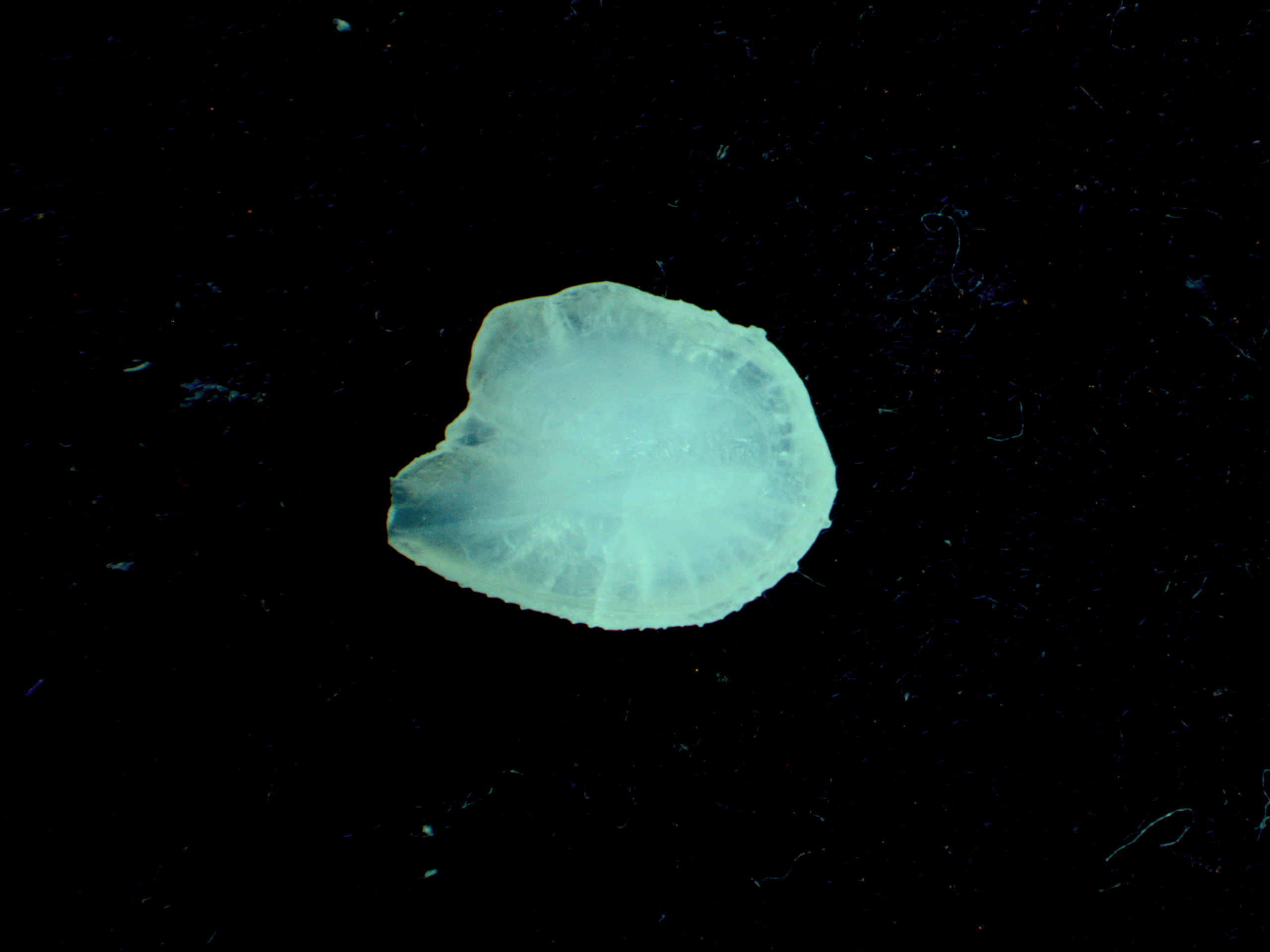

Supplement: Supplemental Information 8 [file peerj-04-1664-s008.zip › Coilia/training/Eng183R1.jpg]

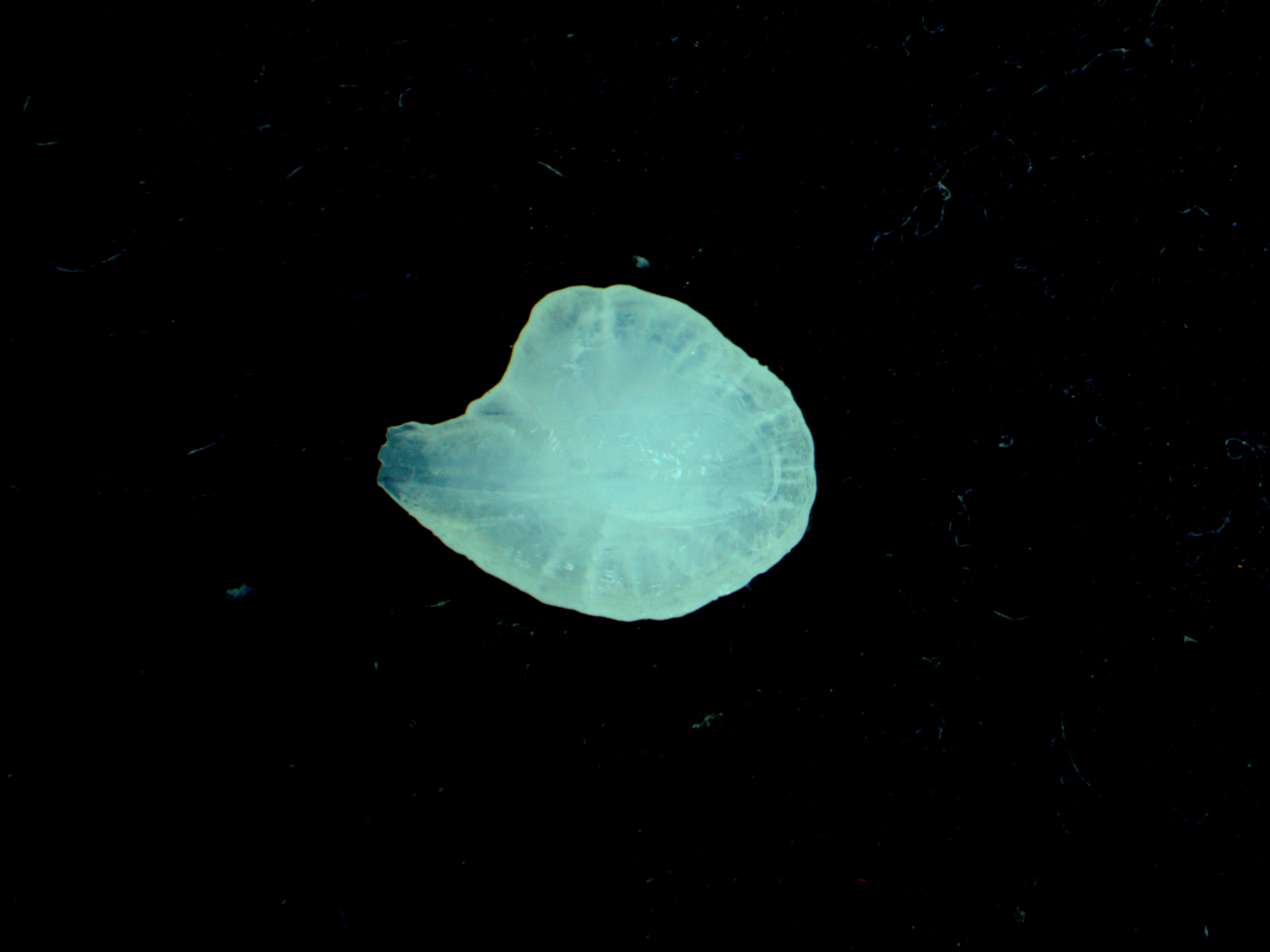

Supplement: Supplemental Information 8 [file peerj-04-1664-s008.zip › Coilia/training/Eng184R1.jpg]

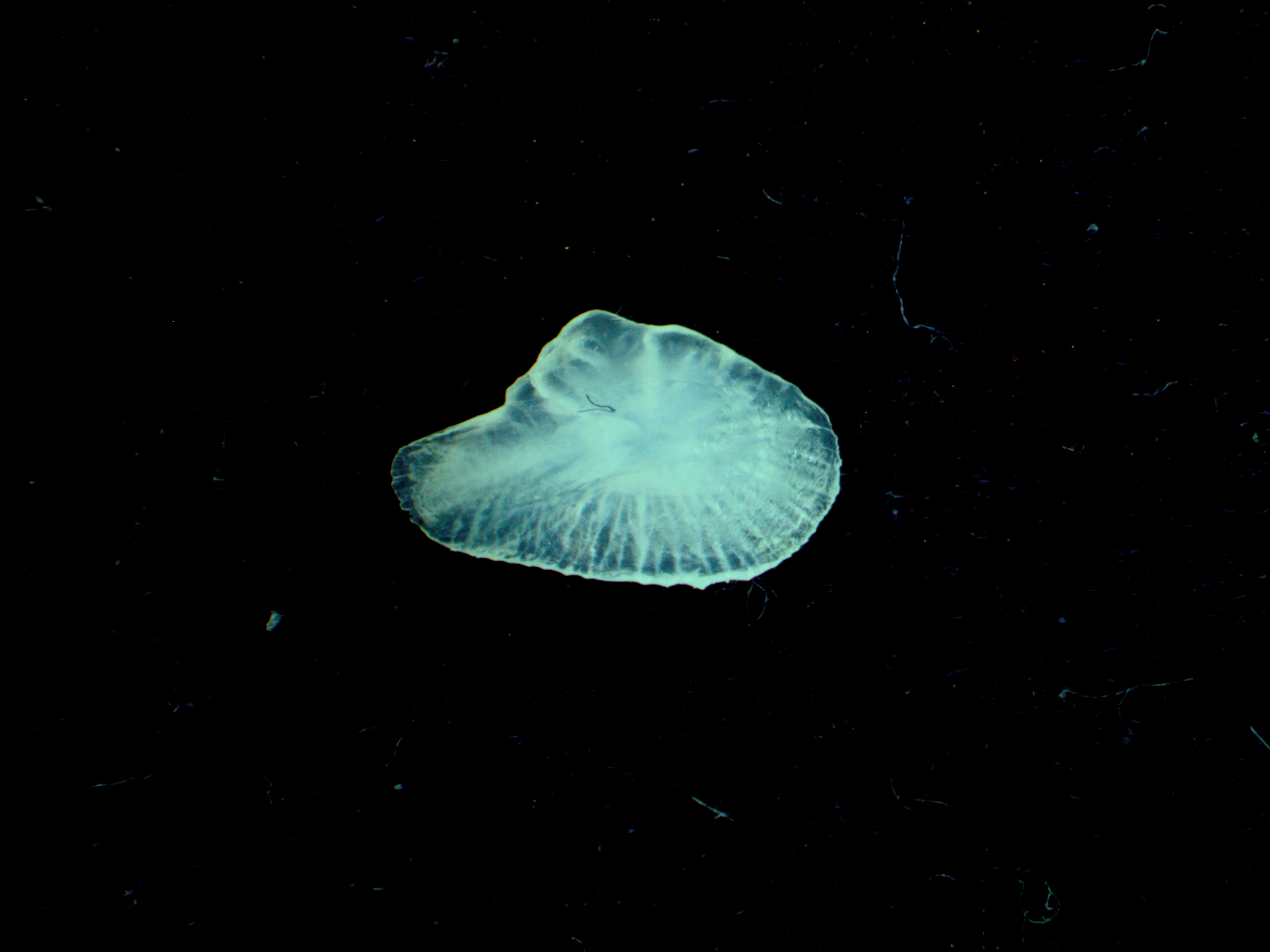

Supplement: Supplemental Information 9 [file peerj-04-1664-s009.zip › Setipinna/testing/Eng217R1.jpg]

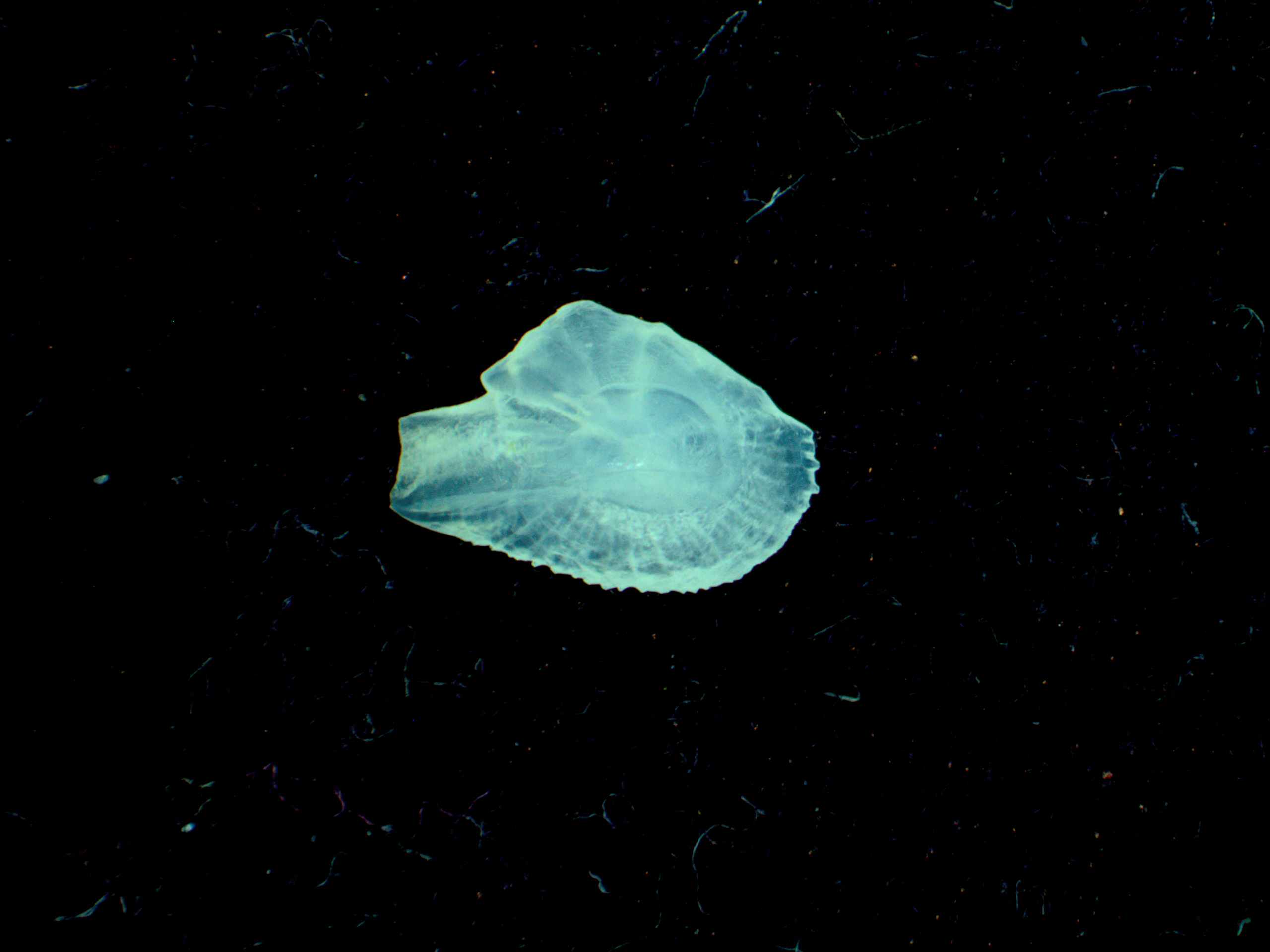

Supplement: Supplemental Information 9 [file peerj-04-1664-s009.zip › Setipinna/testing/Eng250R1.jpg]
