# Supplementary figures and images for: Fully-automated identification of fish species based on otolith contour: using short-time Fourier transform and discriminant analysis (STFT-DA) (part 3 of 5)
Source: PeerJ. 2016 Feb 22;4:e1664. doi: 10.7717/peerj.1664 (PMC4768690; doi:10.7717/peerj.1664)

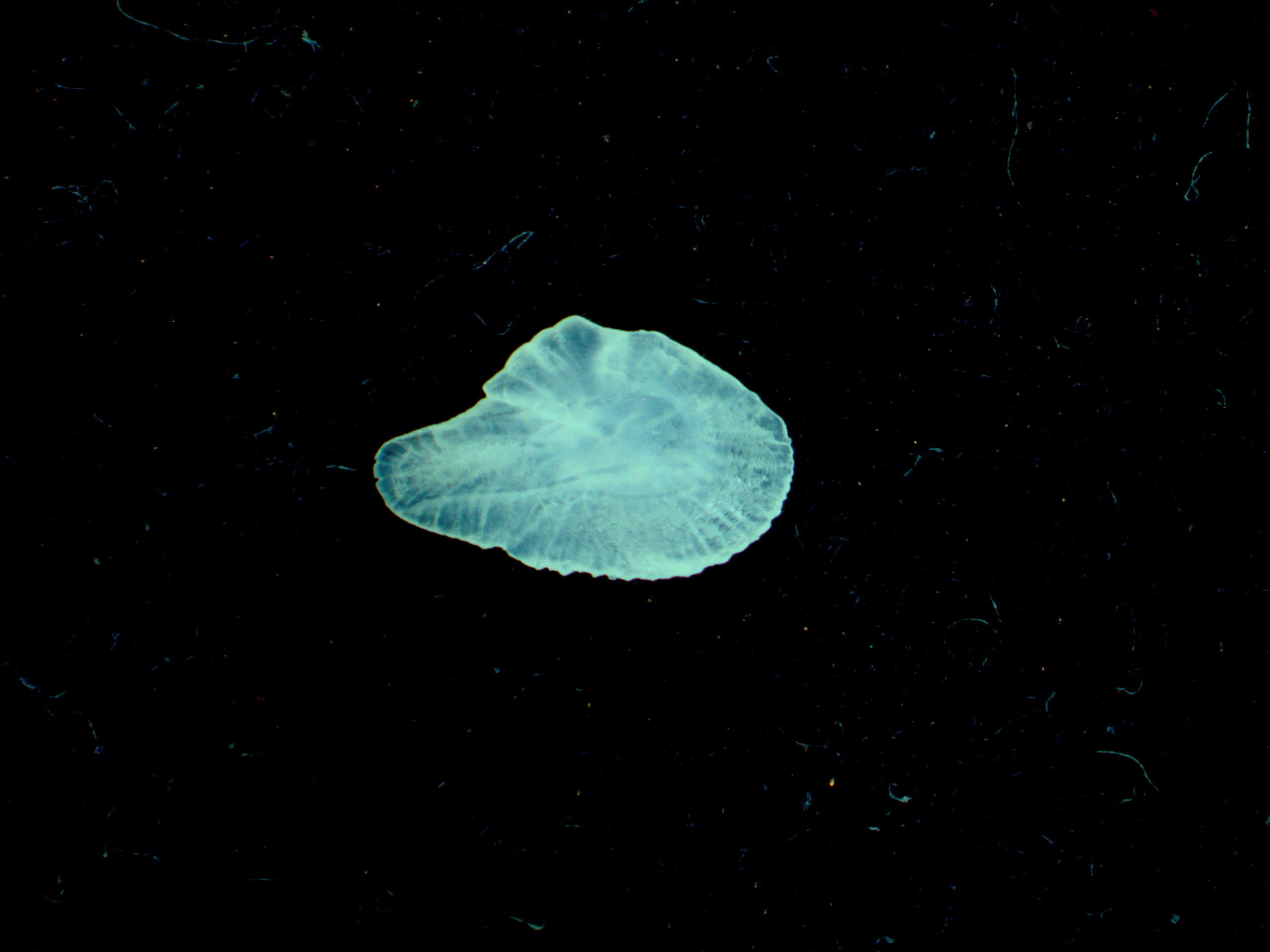

Supplement: Supplemental Information 9 [file peerj-04-1664-s009.zip › Setipinna/testing/Eng251R1.jpg]

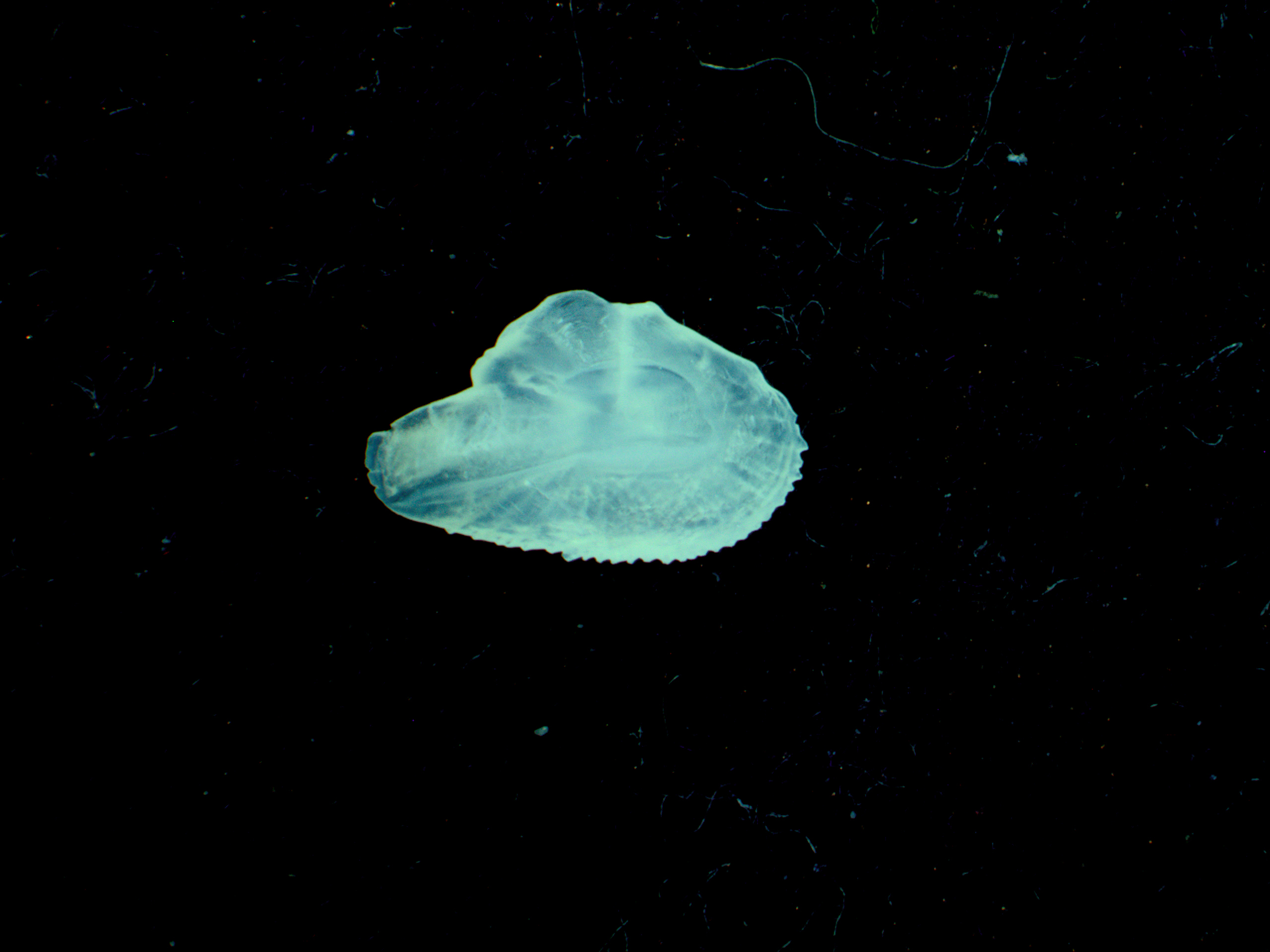

Supplement: Supplemental Information 9 [file peerj-04-1664-s009.zip › Setipinna/testing/Eng252R1.jpg]

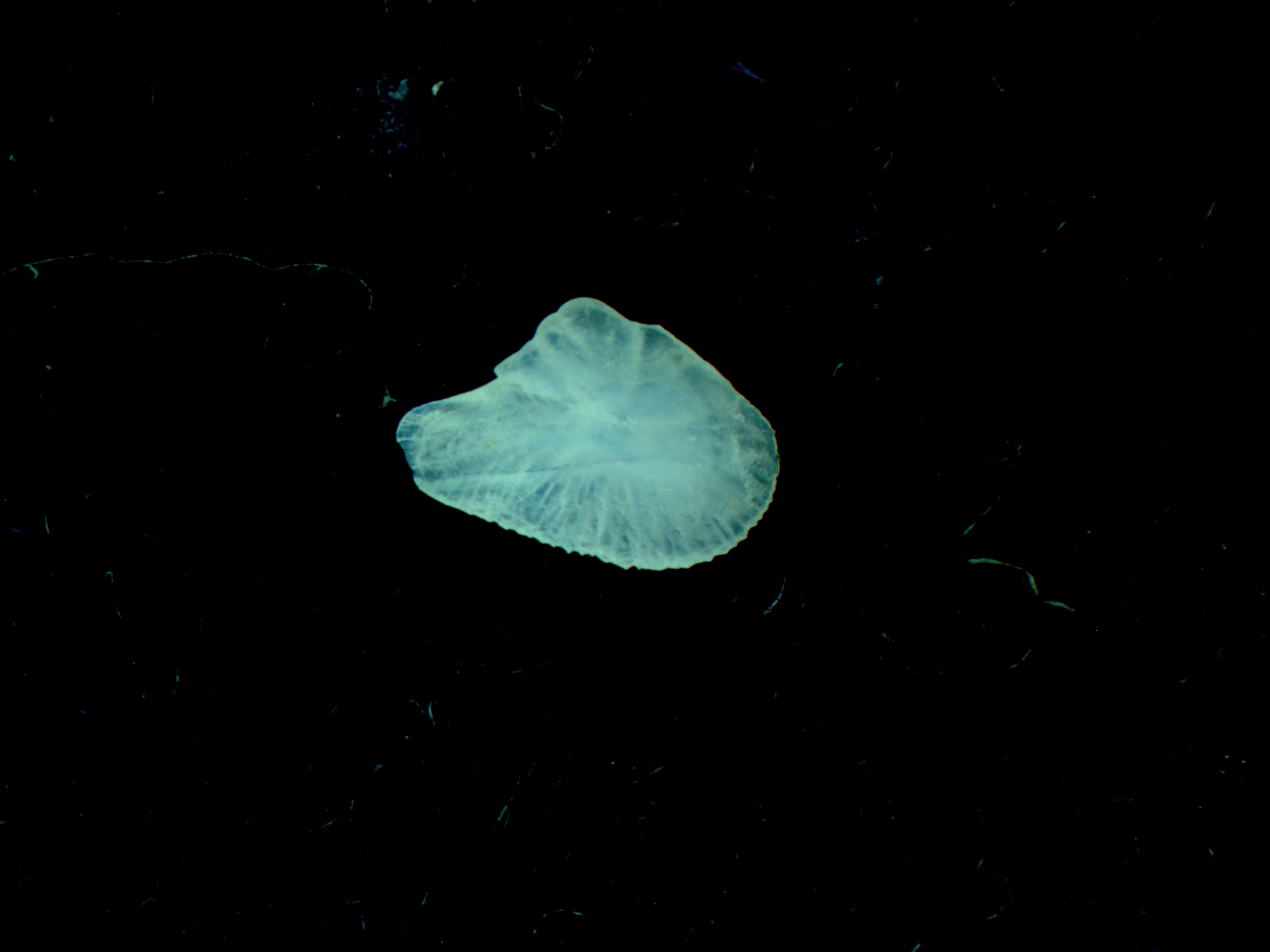

Supplement: Supplemental Information 9 [file peerj-04-1664-s009.zip › Setipinna/testing/Eng253R1.jpg]

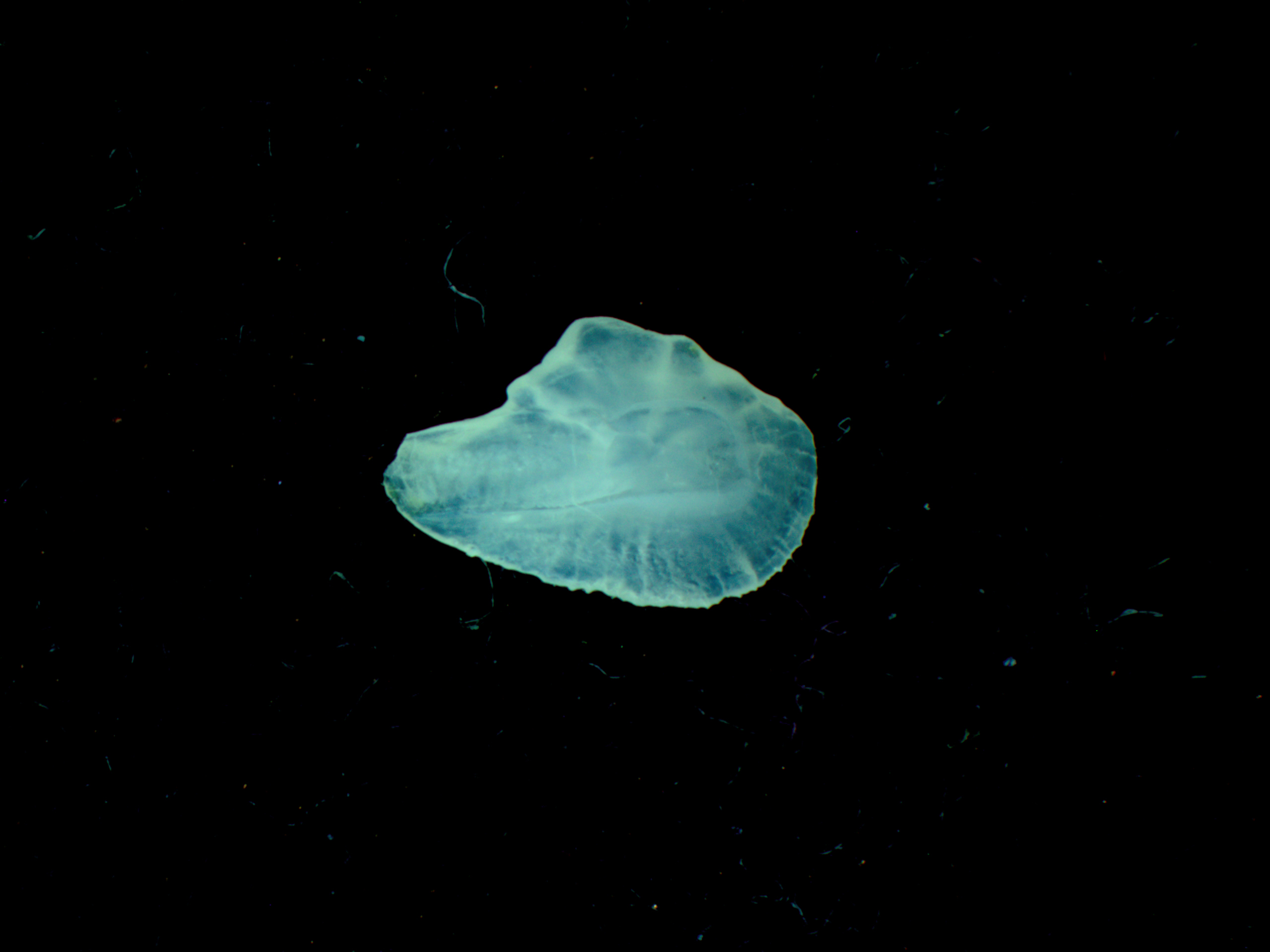

Supplement: Supplemental Information 9 [file peerj-04-1664-s009.zip › Setipinna/testing/Eng254R1.jpg]

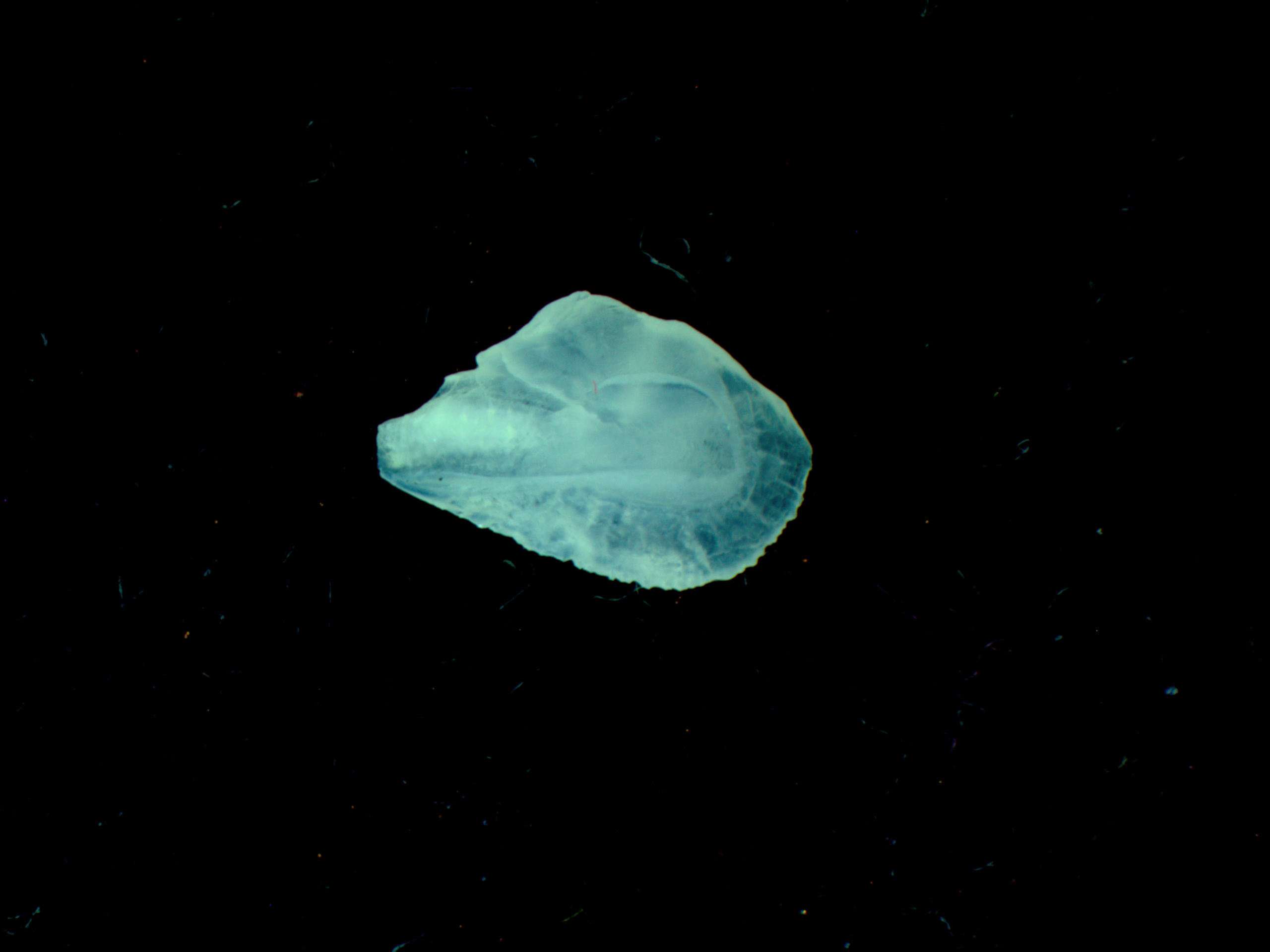

Supplement: Supplemental Information 9 [file peerj-04-1664-s009.zip › Setipinna/testing/Eng255R1.jpg]

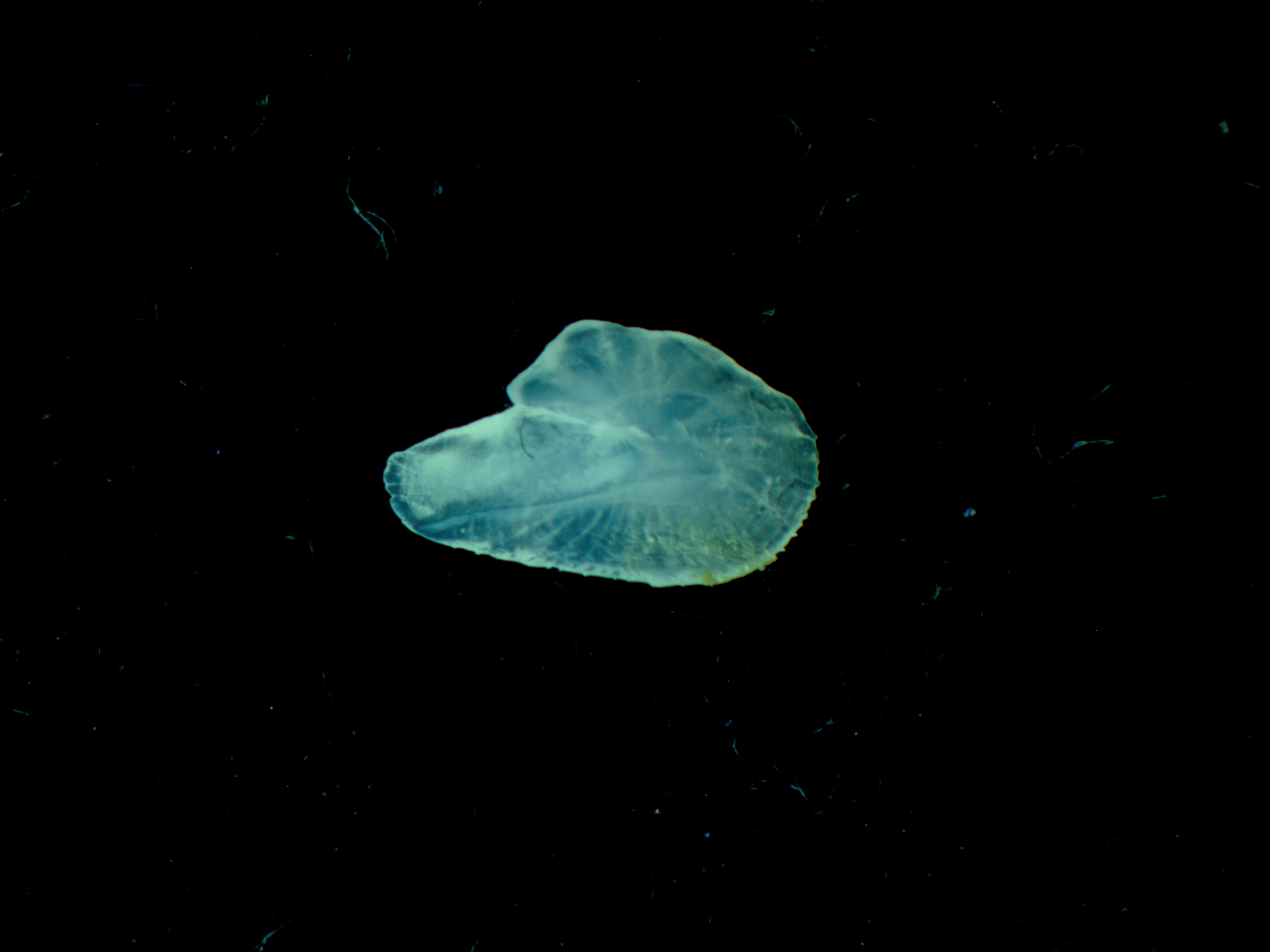

Supplement: Supplemental Information 9 [file peerj-04-1664-s009.zip › Setipinna/testing/Eng256R1.jpg]

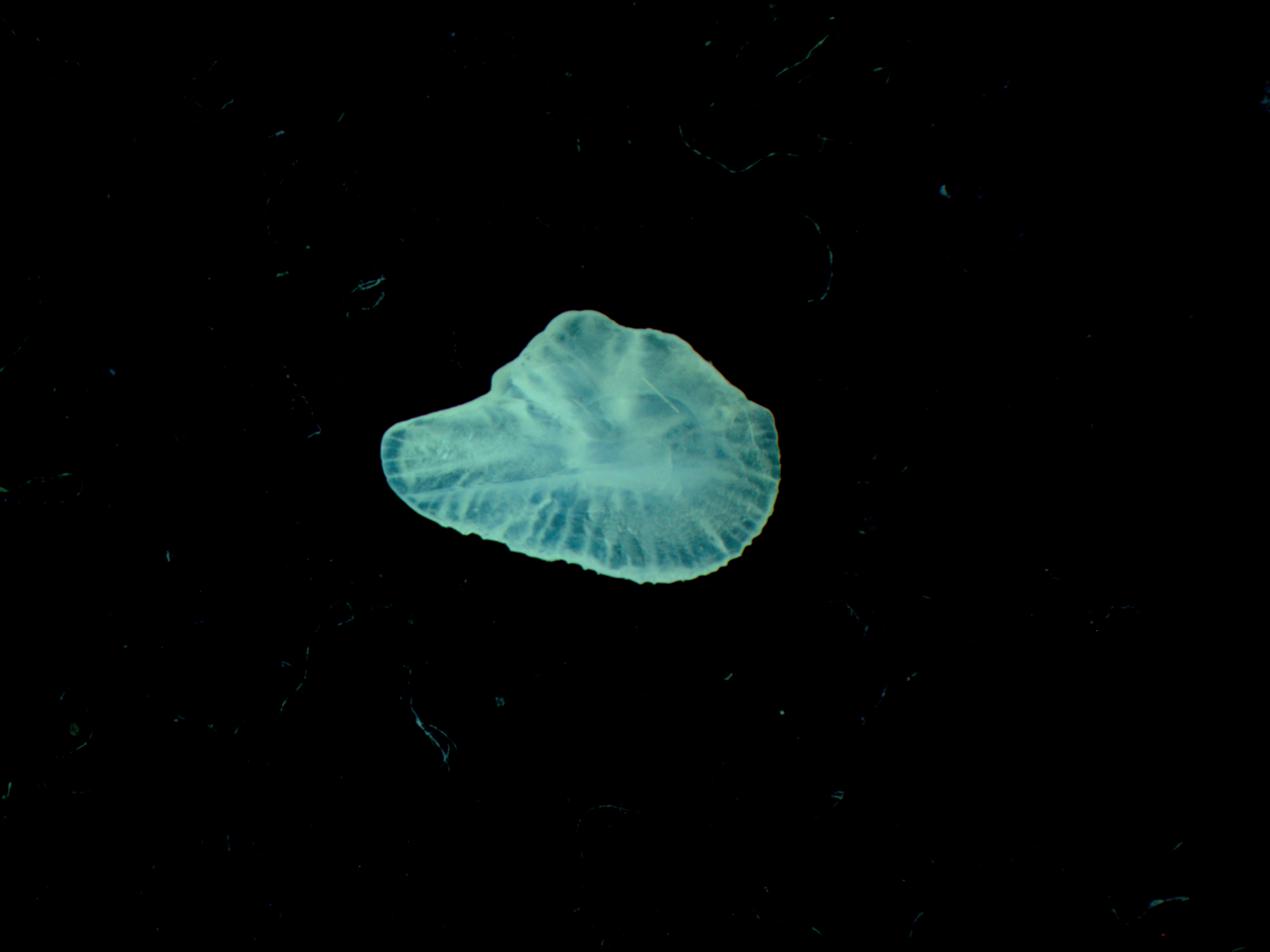

Supplement: Supplemental Information 9 [file peerj-04-1664-s009.zip › Setipinna/testing/Eng257R1.jpg]

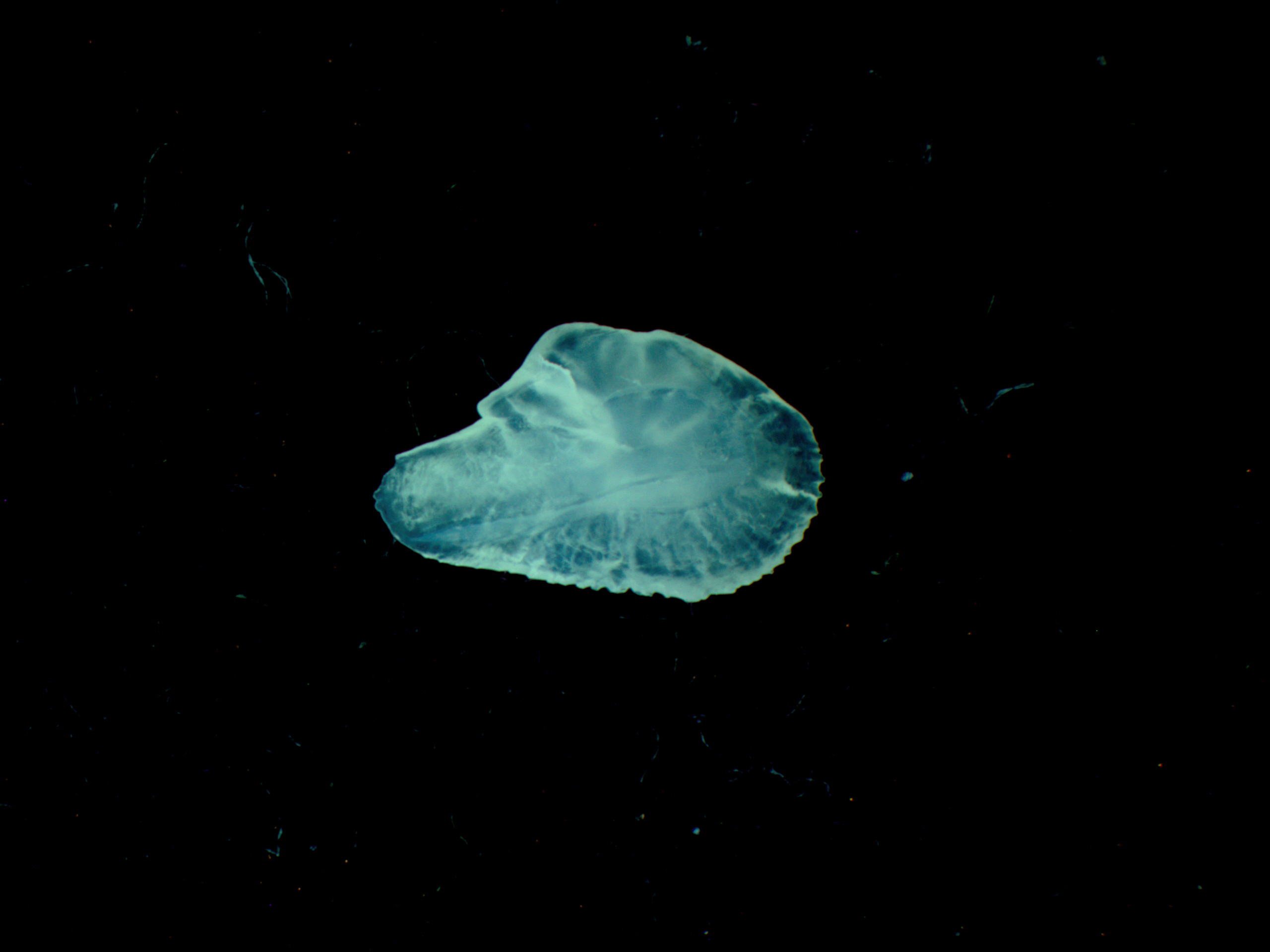

Supplement: Supplemental Information 9 [file peerj-04-1664-s009.zip › Setipinna/testing/Eng258R1.jpg]

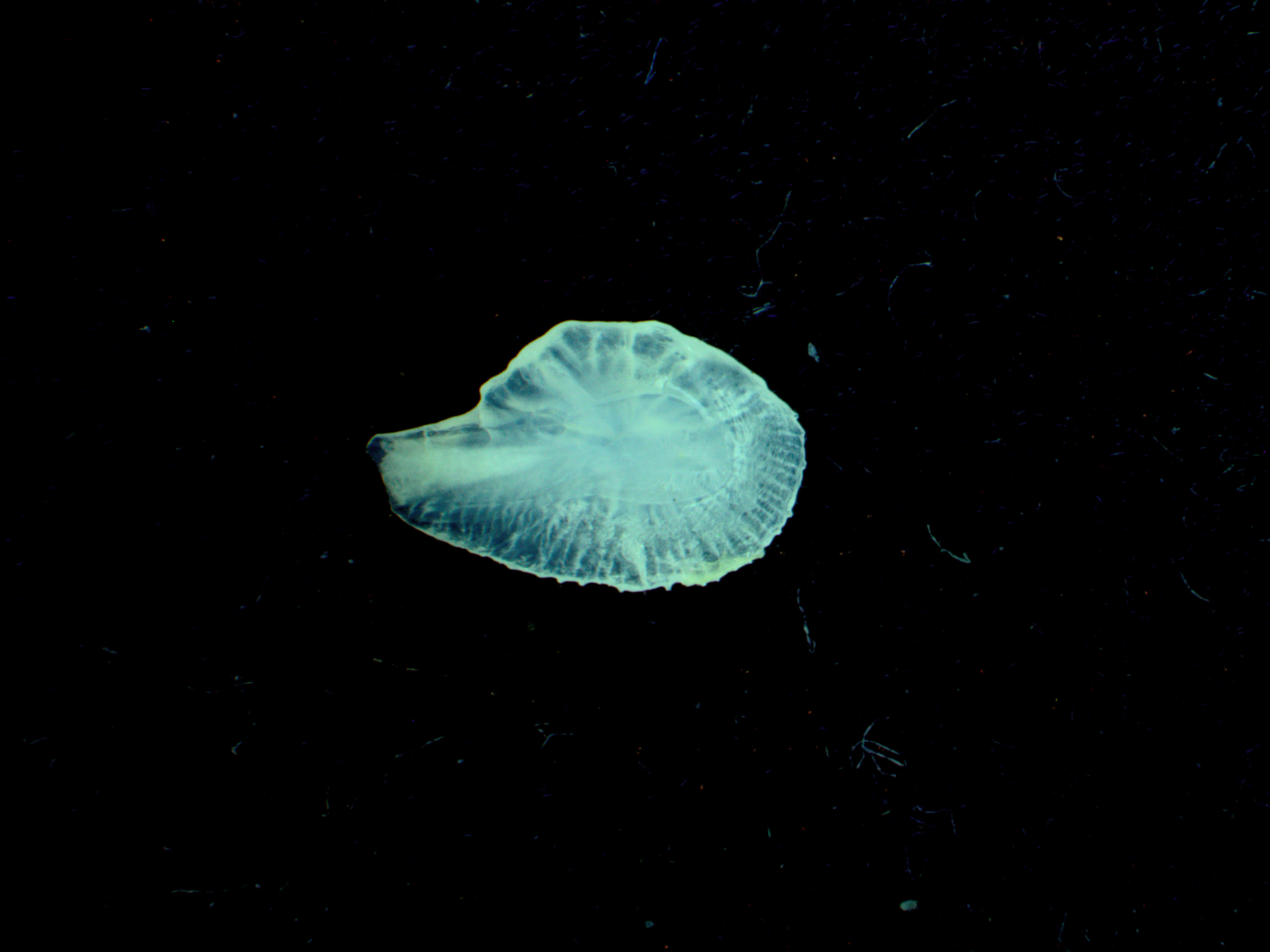

Supplement: Supplemental Information 9 [file peerj-04-1664-s009.zip › Setipinna/training/Eng197R1.jpg]

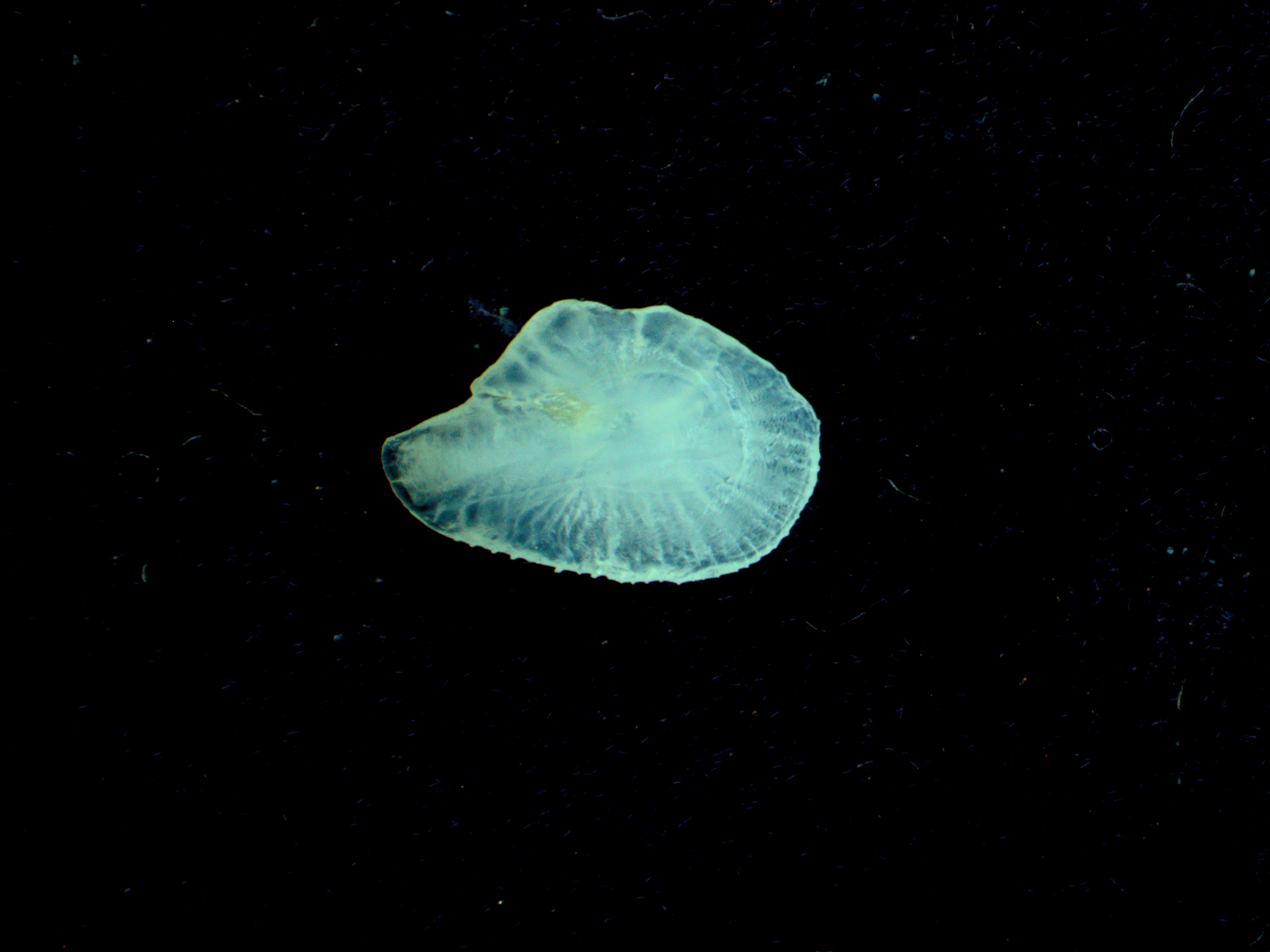

Supplement: Supplemental Information 9 [file peerj-04-1664-s009.zip › Setipinna/training/Eng198R1.jpg]

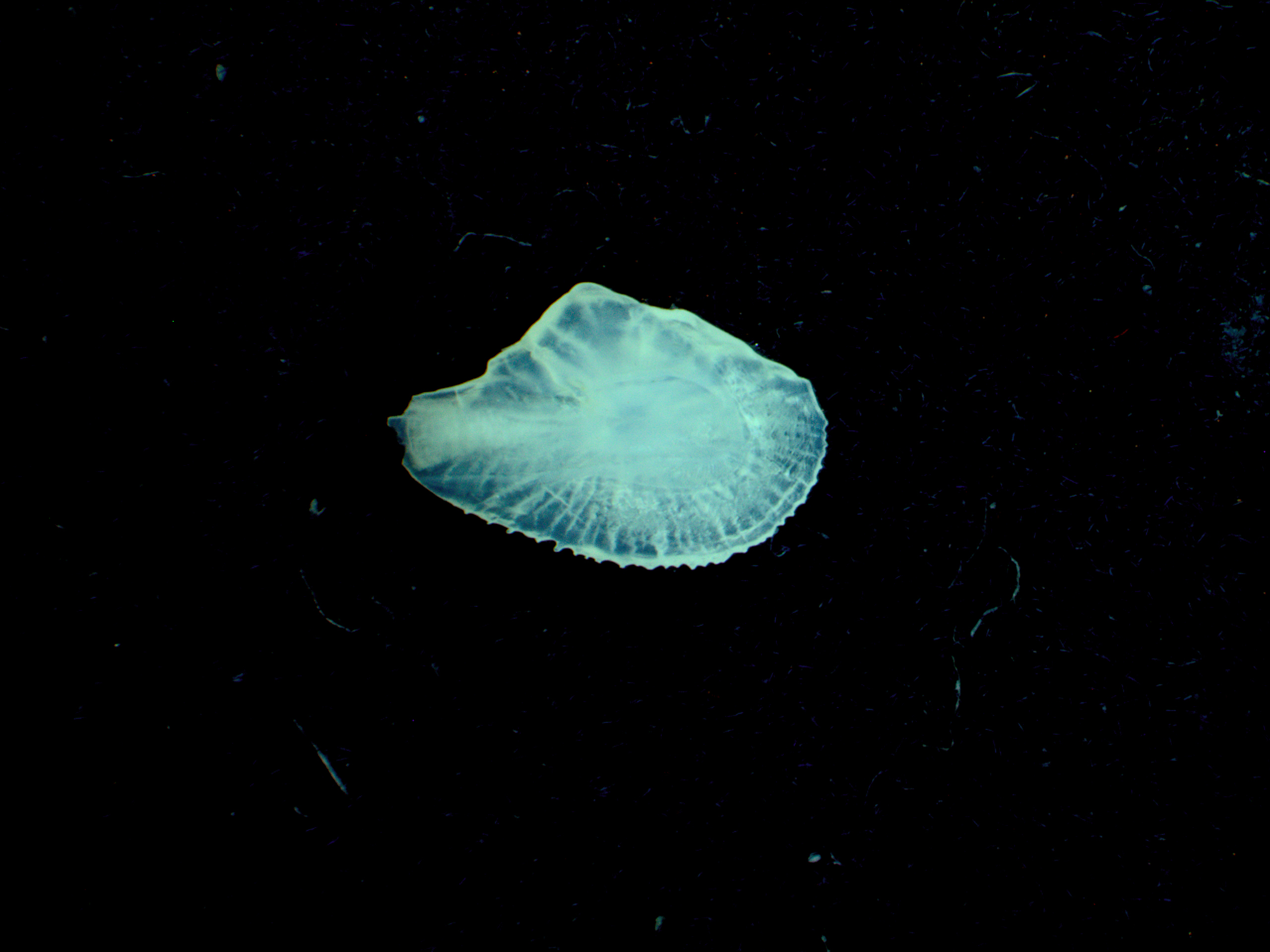

Supplement: Supplemental Information 9 [file peerj-04-1664-s009.zip › Setipinna/training/Eng199R1.jpg]

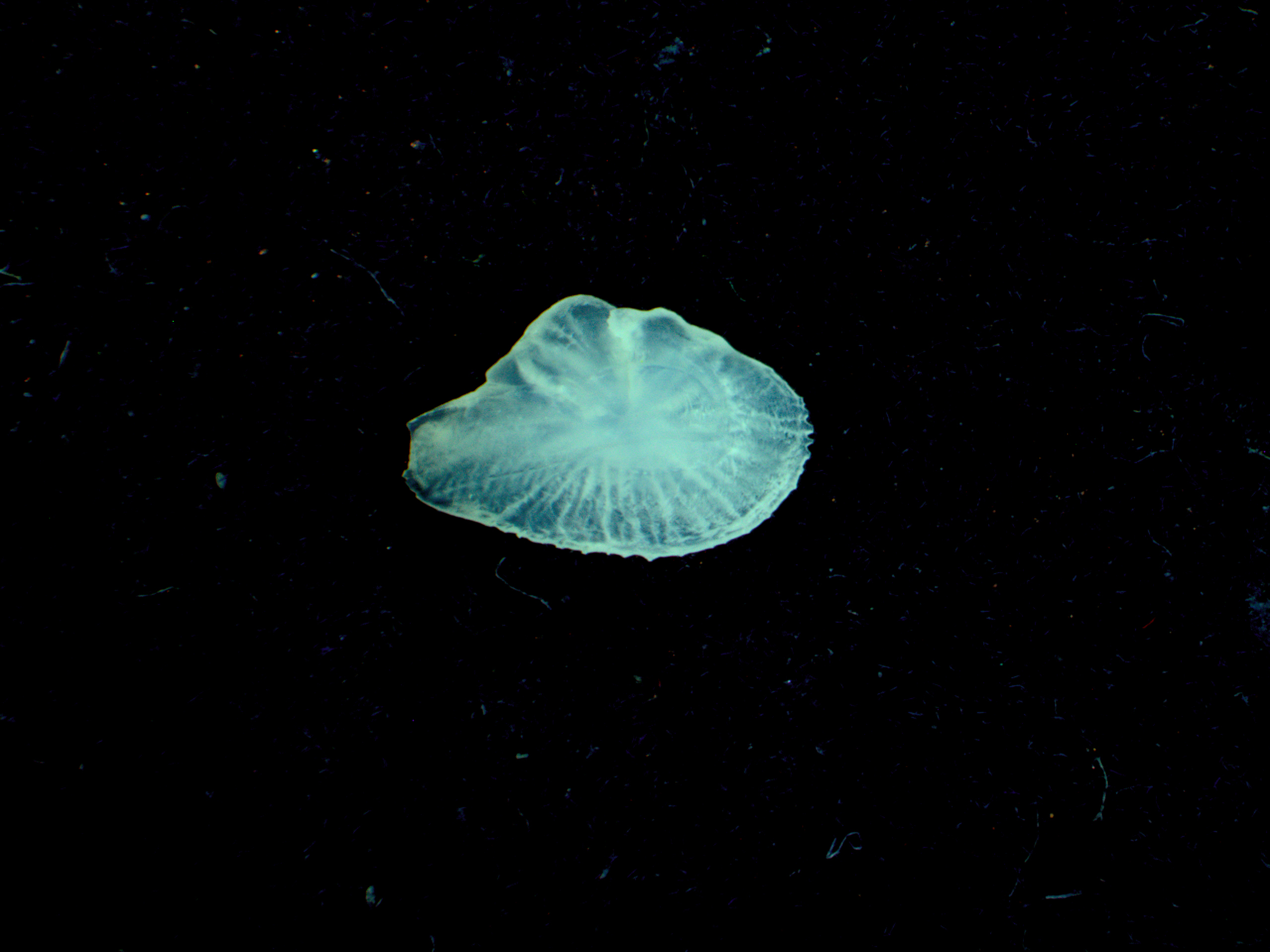

Supplement: Supplemental Information 9 [file peerj-04-1664-s009.zip › Setipinna/training/Eng200R1.jpg]

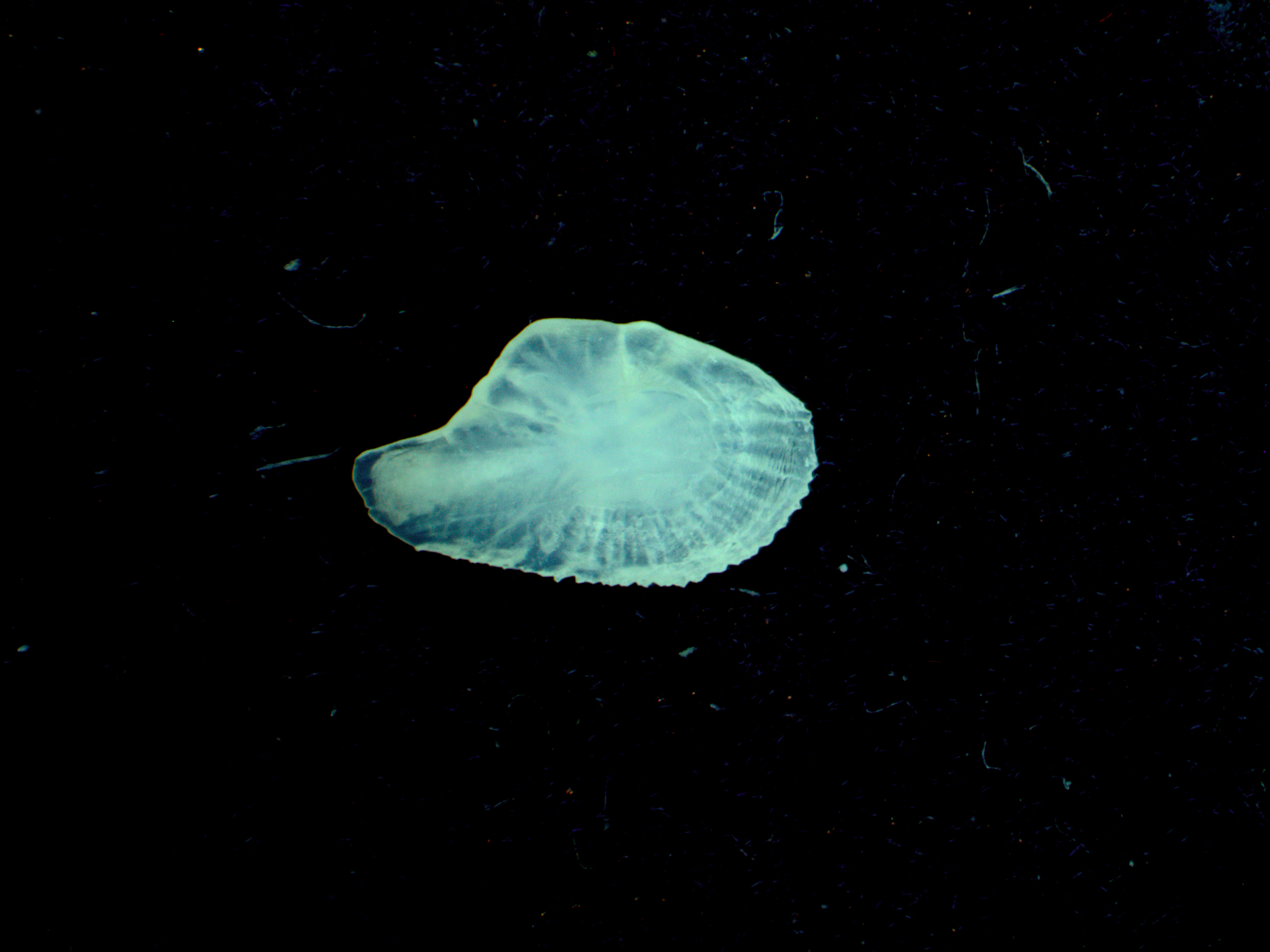

Supplement: Supplemental Information 9 [file peerj-04-1664-s009.zip › Setipinna/training/Eng201R1.jpg]

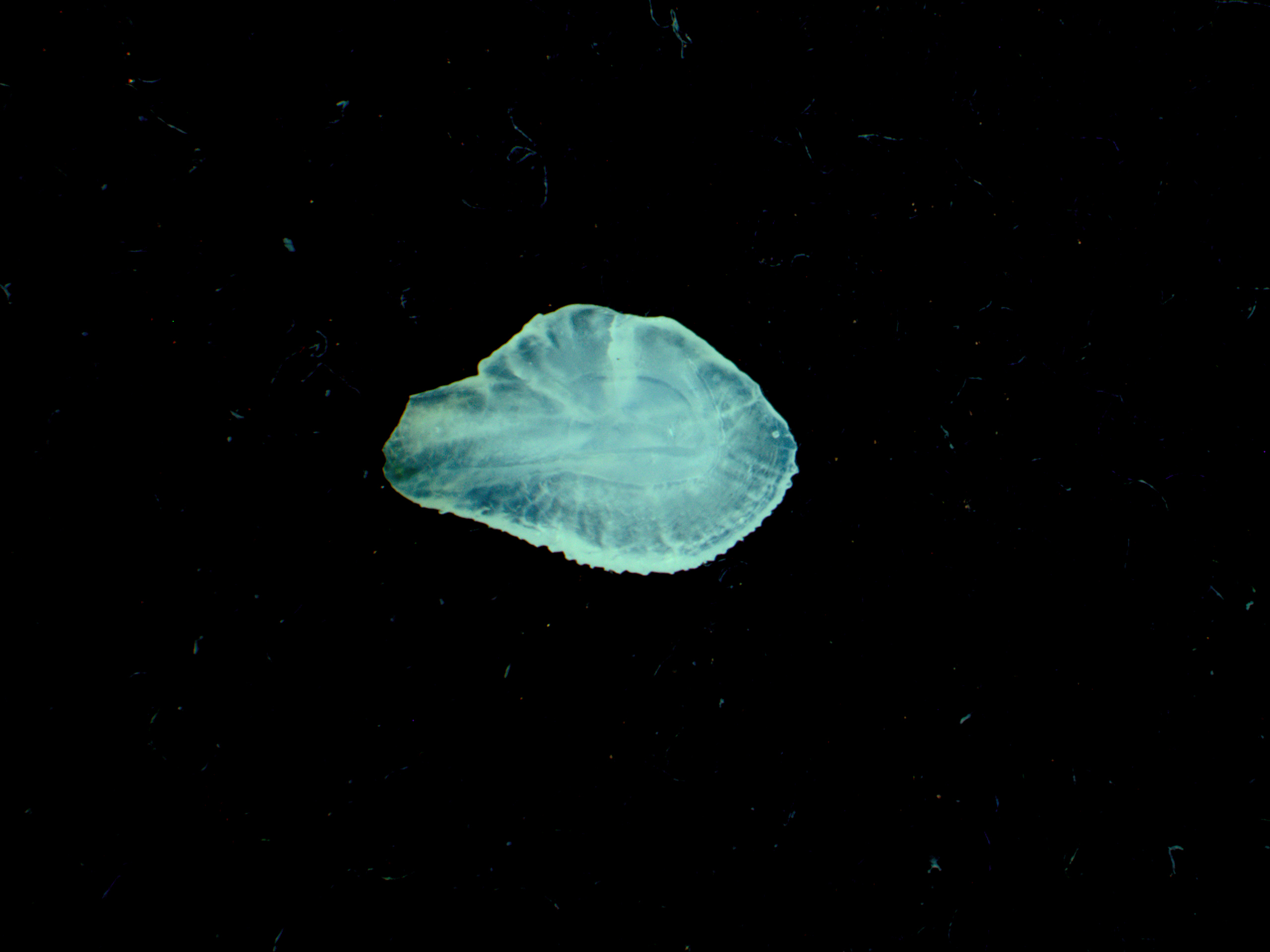

Supplement: Supplemental Information 9 [file peerj-04-1664-s009.zip › Setipinna/training/Eng202R1.jpg]

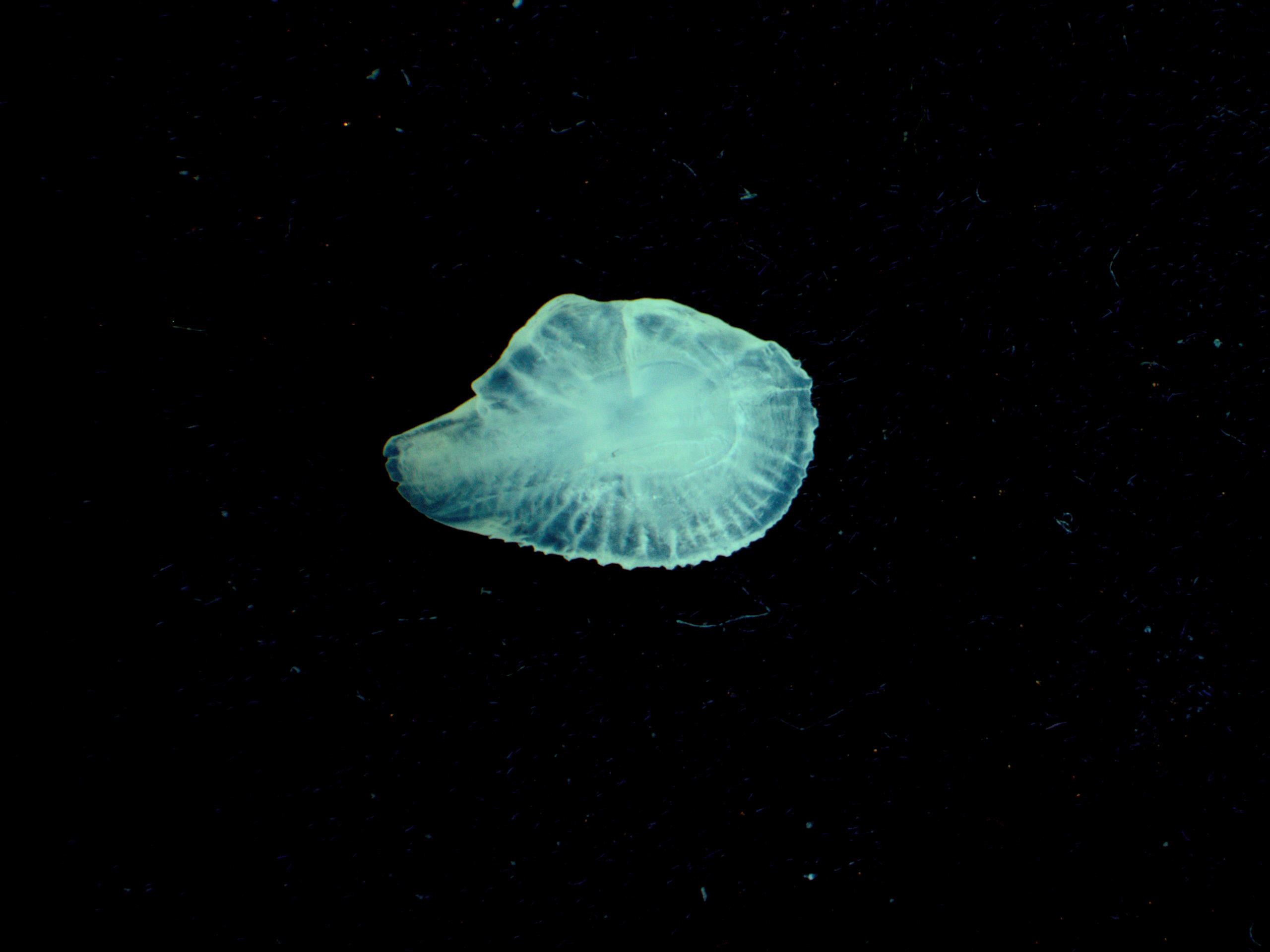

Supplement: Supplemental Information 9 [file peerj-04-1664-s009.zip › Setipinna/training/Eng203R1.jpg]

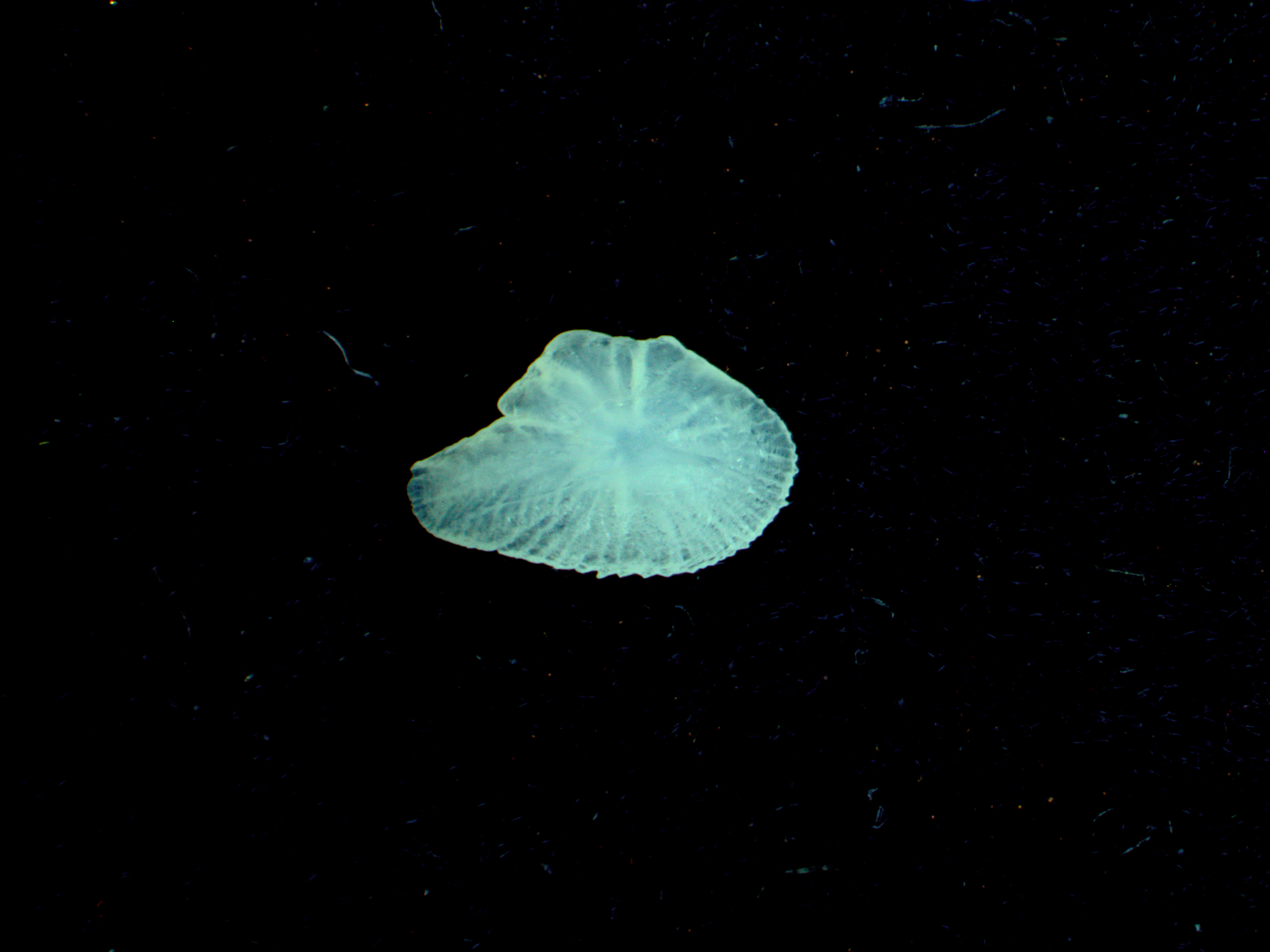

Supplement: Supplemental Information 9 [file peerj-04-1664-s009.zip › Setipinna/training/Eng204R1.jpg]

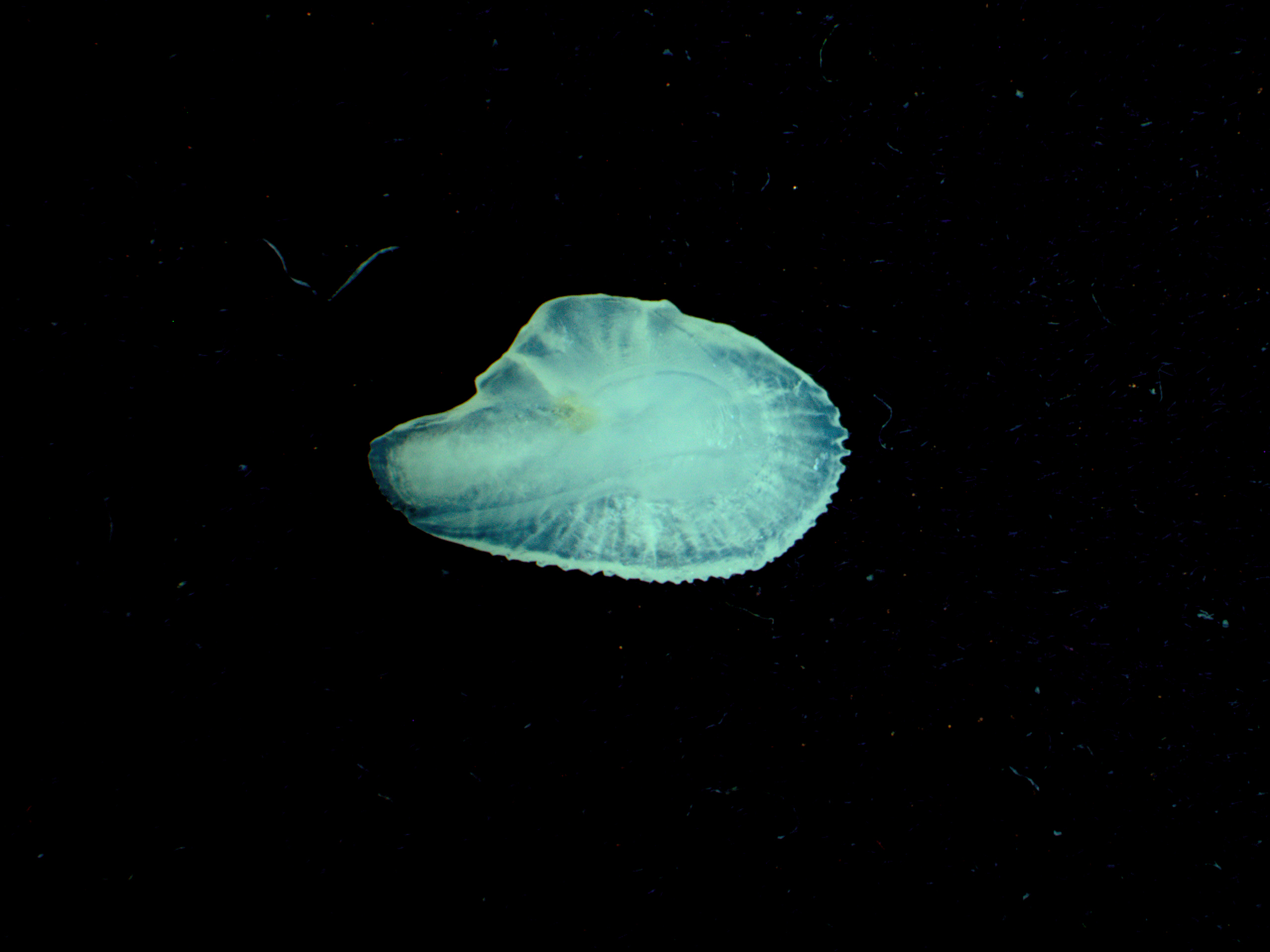

Supplement: Supplemental Information 9 [file peerj-04-1664-s009.zip › Setipinna/training/Eng205R1.jpg]

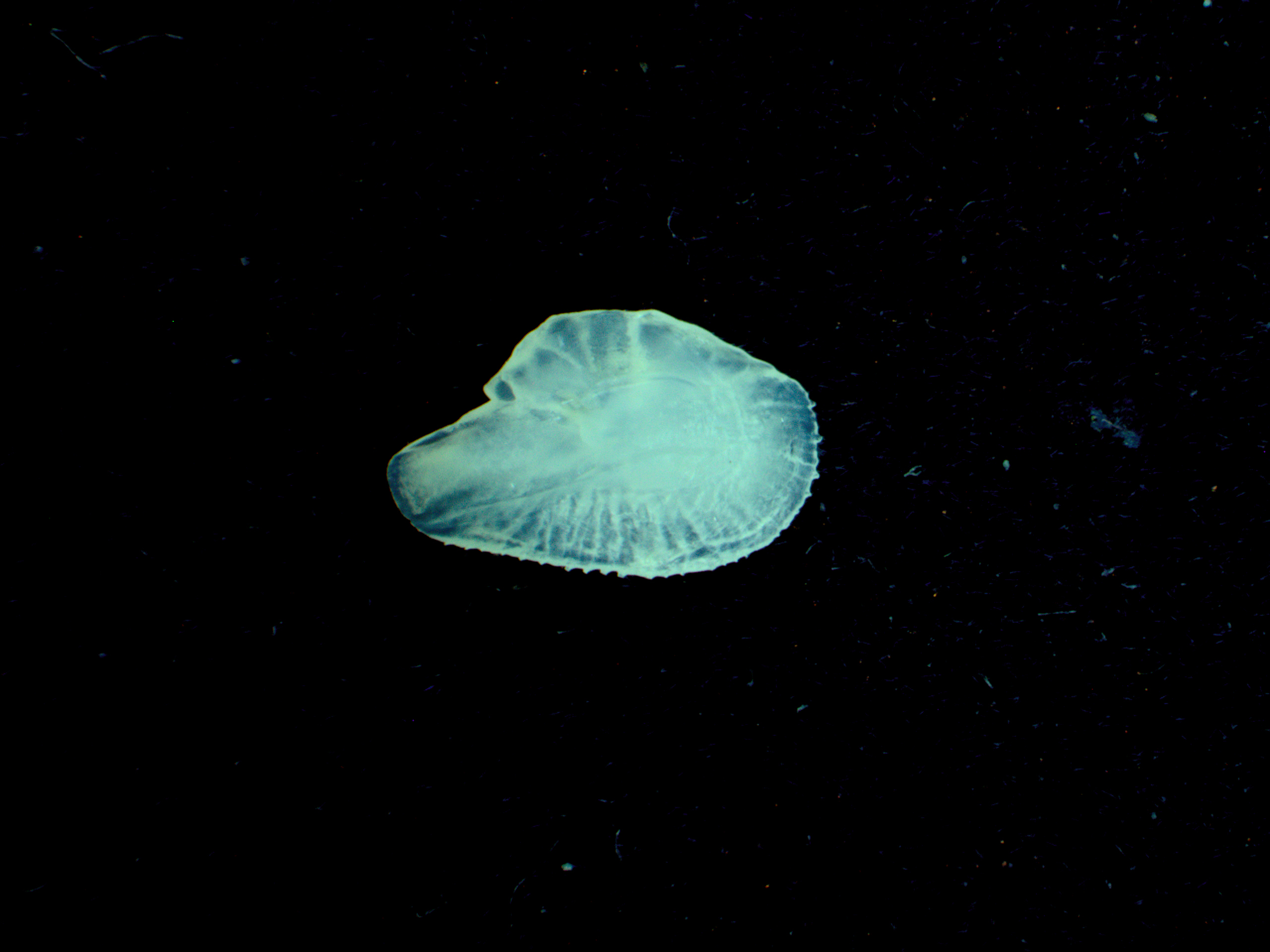

Supplement: Supplemental Information 9 [file peerj-04-1664-s009.zip › Setipinna/training/Eng206R1.jpg]

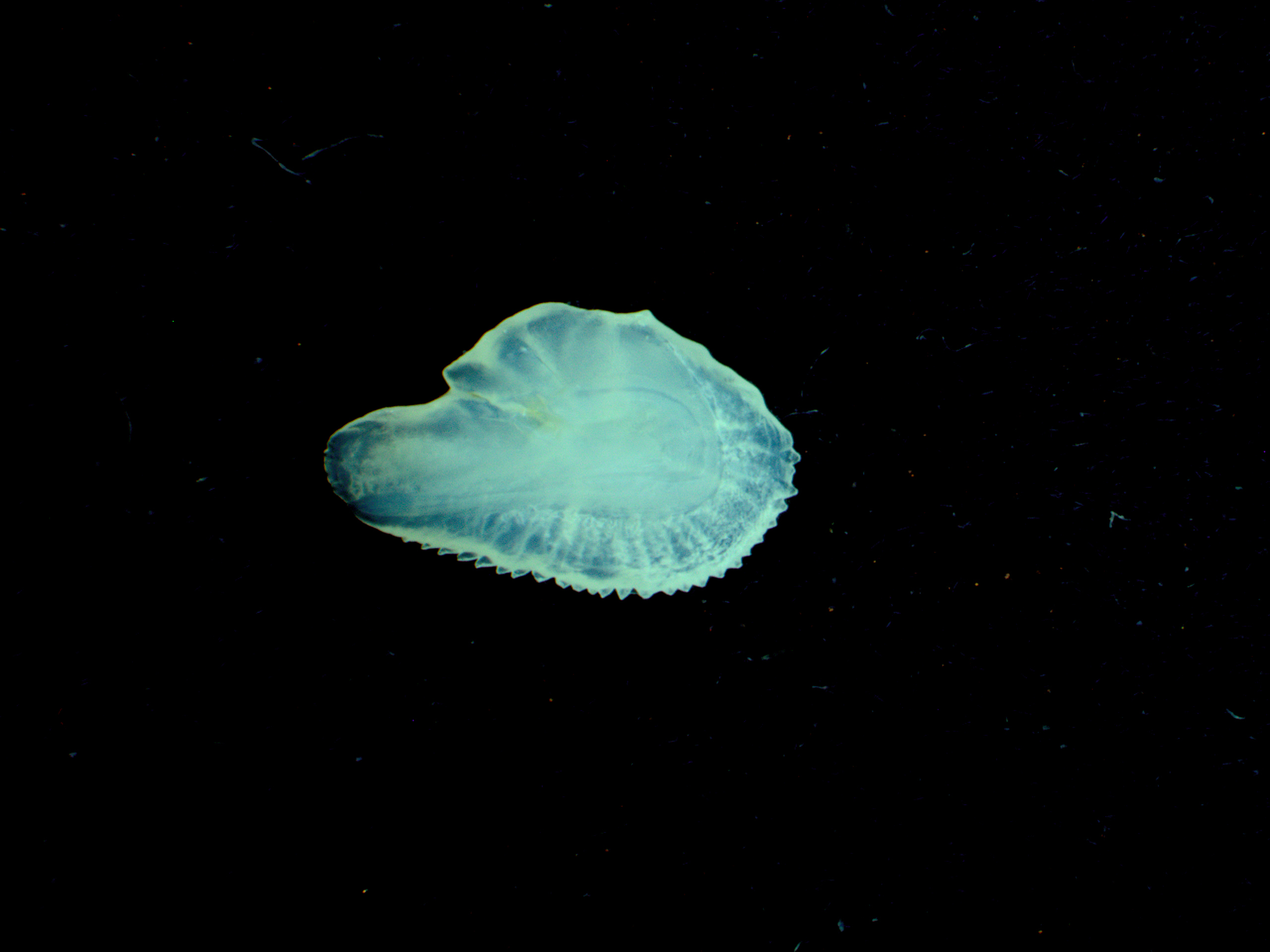

Supplement: Supplemental Information 9 [file peerj-04-1664-s009.zip › Setipinna/training/Eng207R1.jpg]

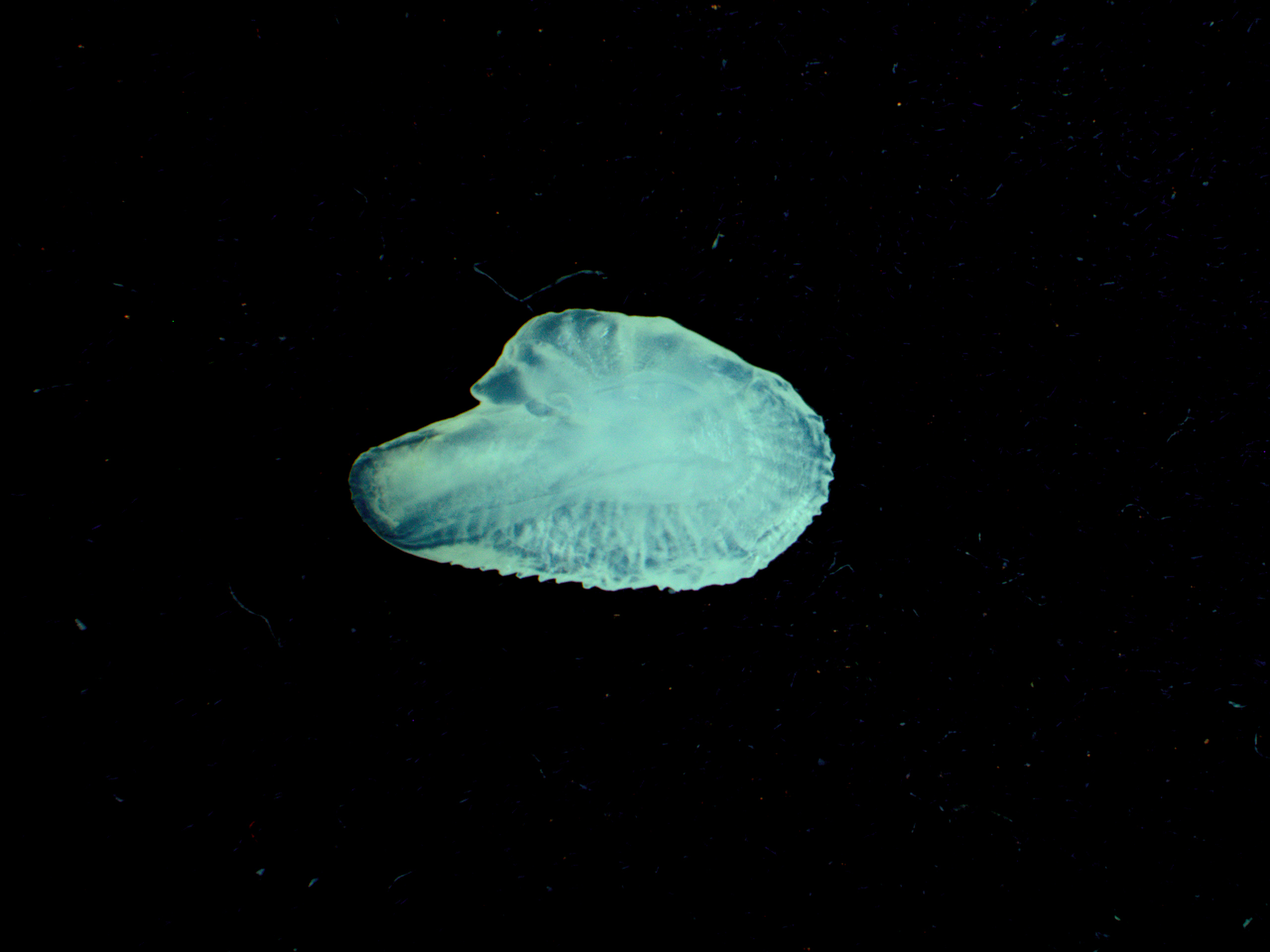

Supplement: Supplemental Information 9 [file peerj-04-1664-s009.zip › Setipinna/training/Eng208R1.jpg]

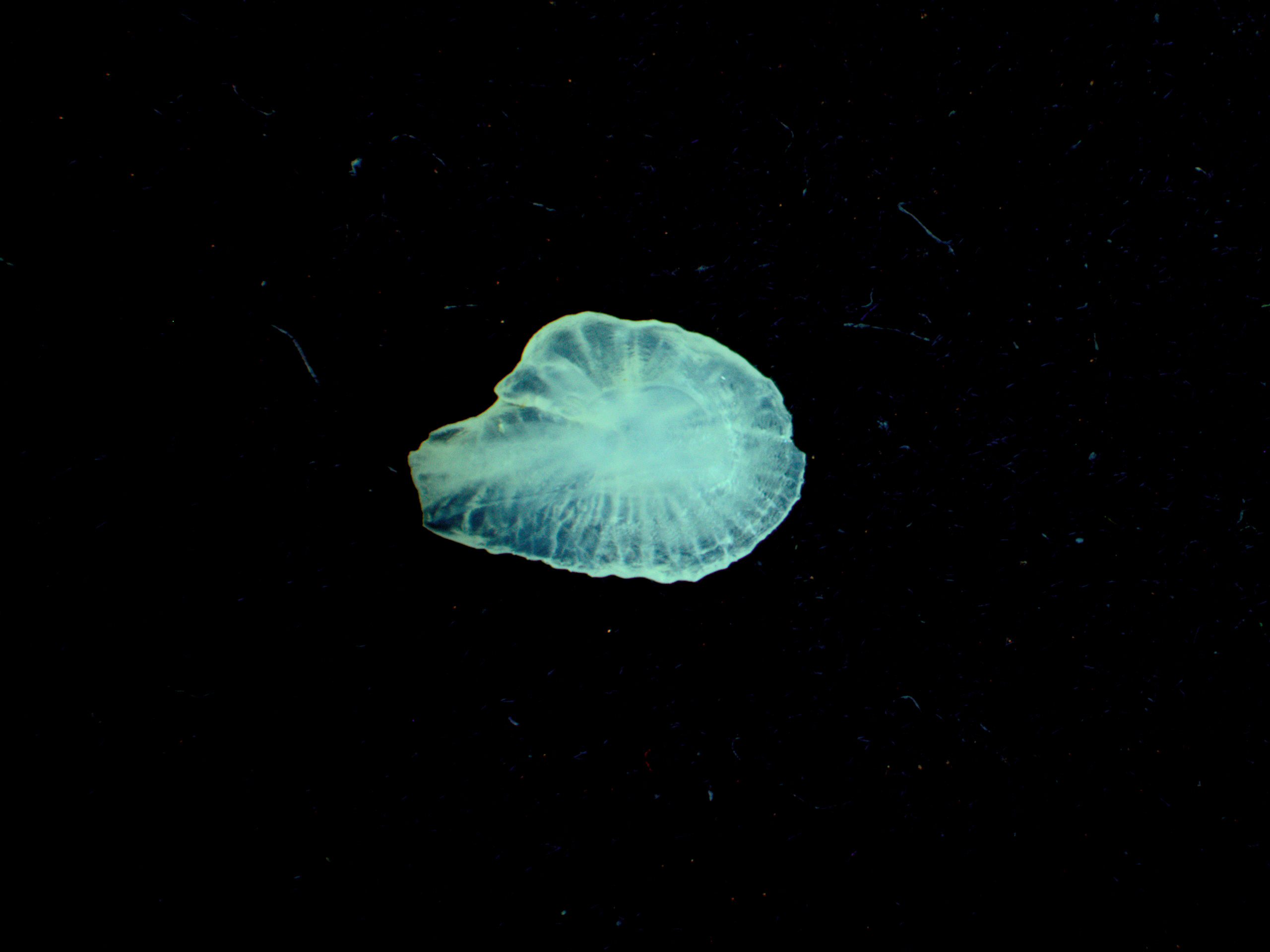

Supplement: Supplemental Information 9 [file peerj-04-1664-s009.zip › Setipinna/training/Eng209R1.jpg]

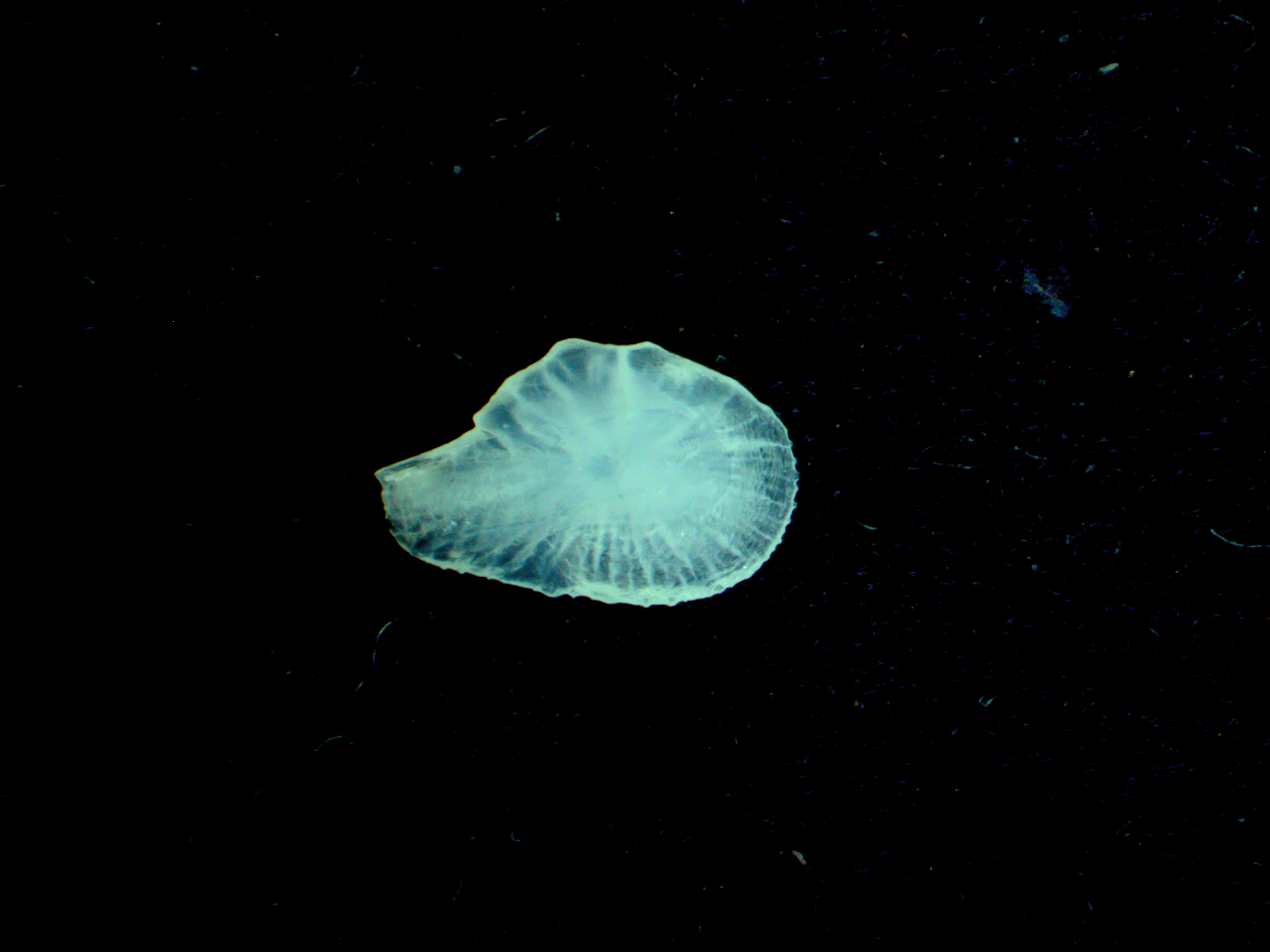

Supplement: Supplemental Information 9 [file peerj-04-1664-s009.zip › Setipinna/training/Eng210R1.jpg]

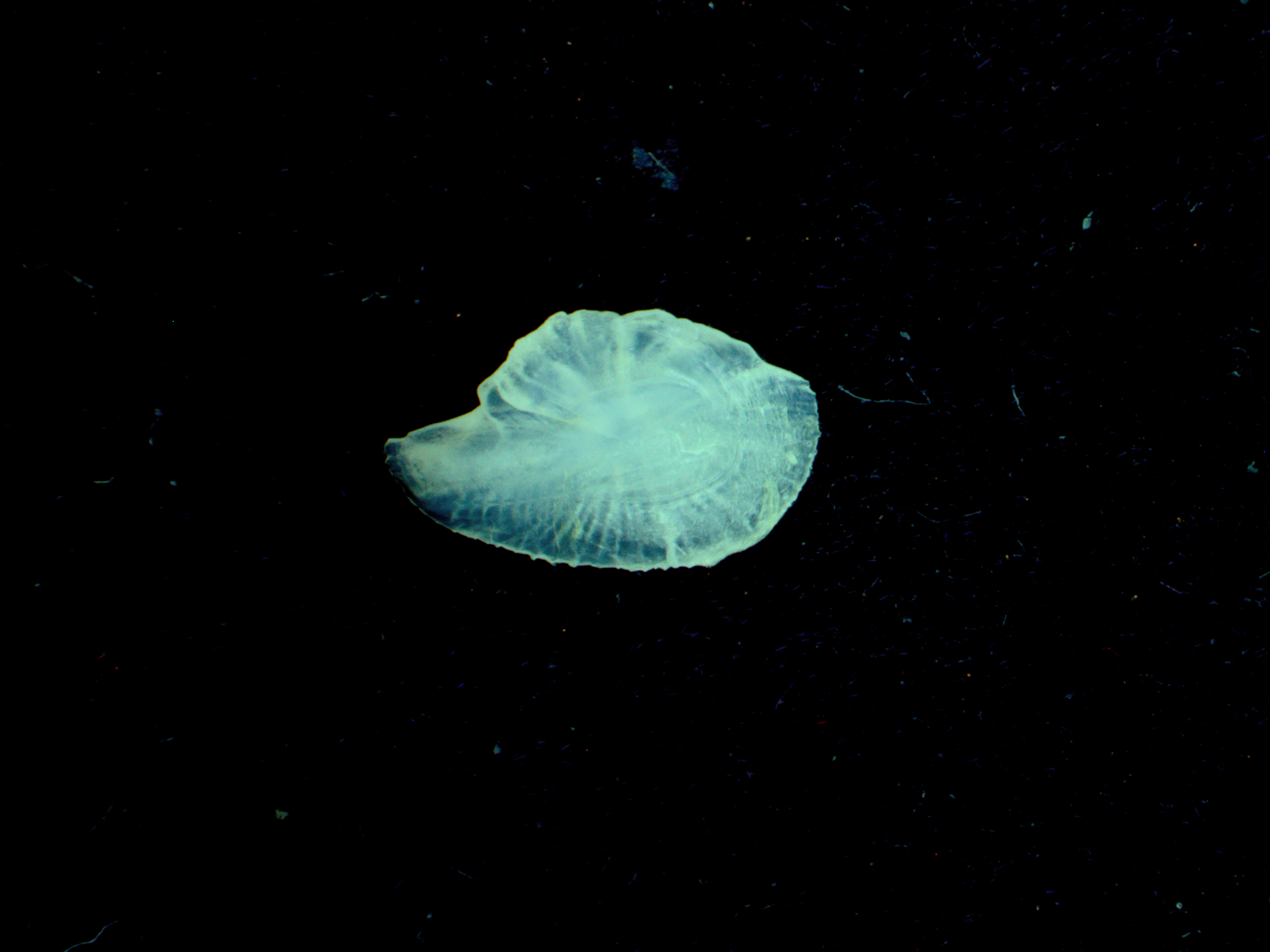

Supplement: Supplemental Information 9 [file peerj-04-1664-s009.zip › Setipinna/training/Eng211R1.jpg]

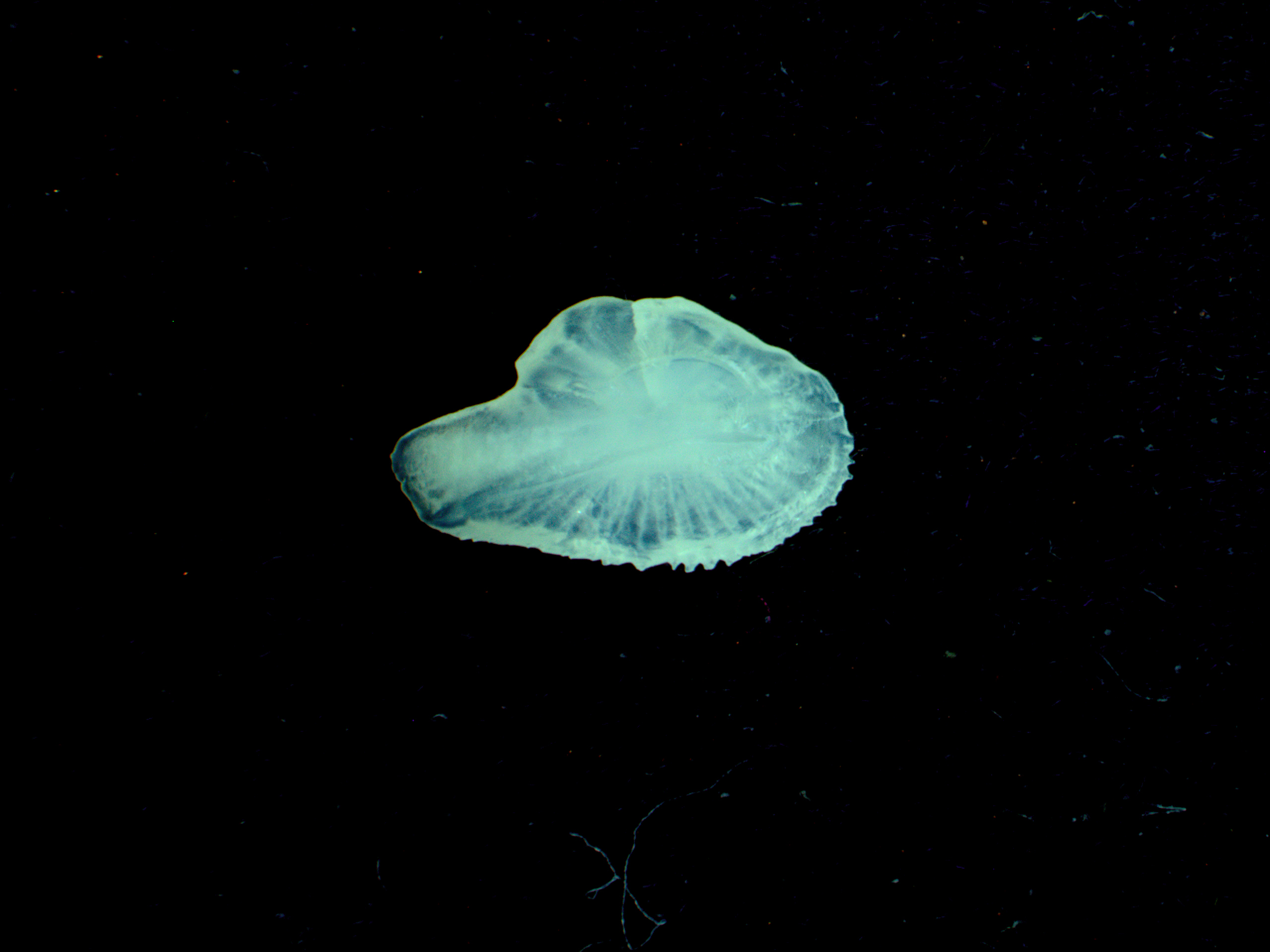

Supplement: Supplemental Information 9 [file peerj-04-1664-s009.zip › Setipinna/training/Eng212R1.jpg]

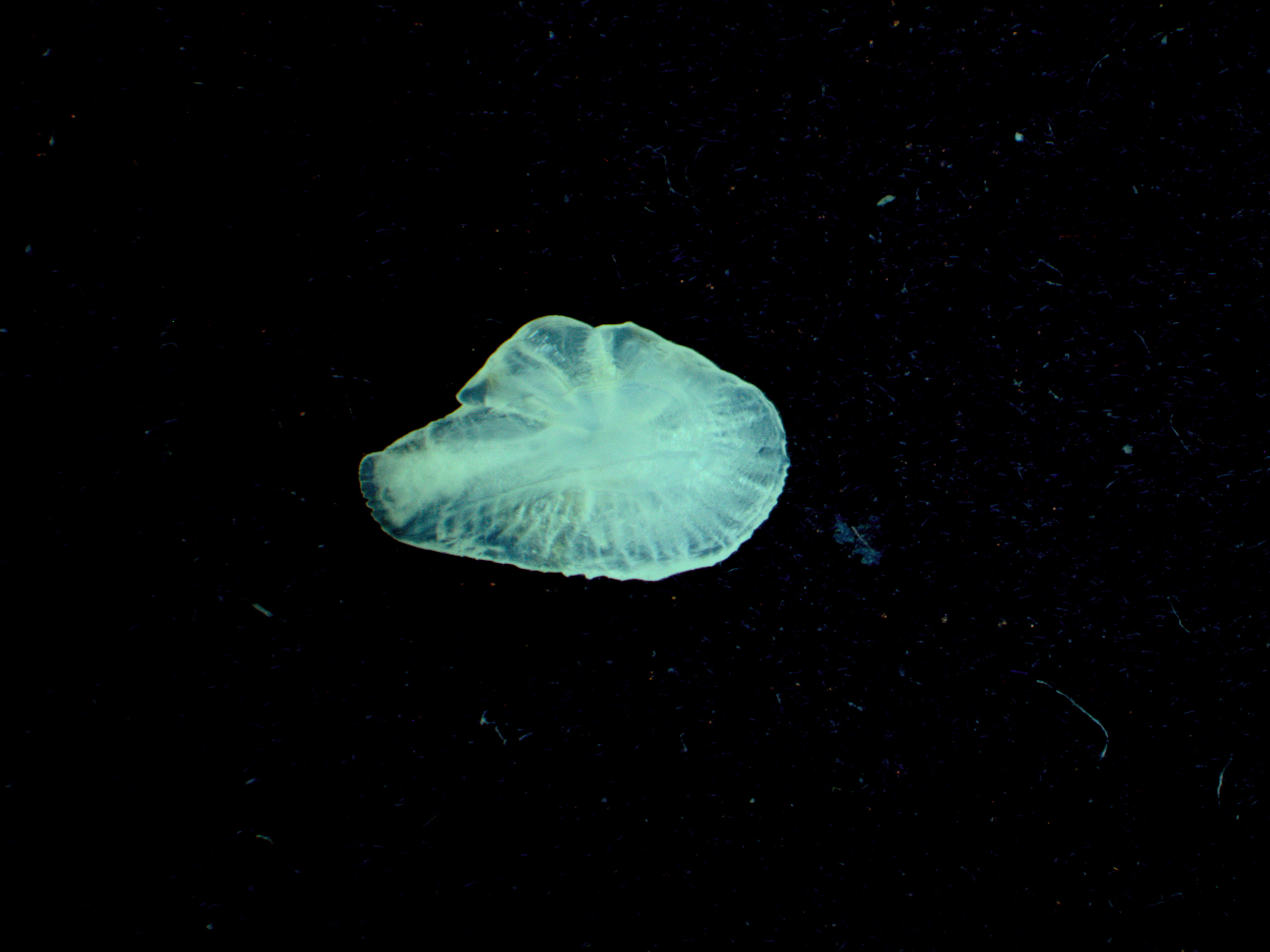

Supplement: Supplemental Information 9 [file peerj-04-1664-s009.zip › Setipinna/training/Eng213R1.jpg]

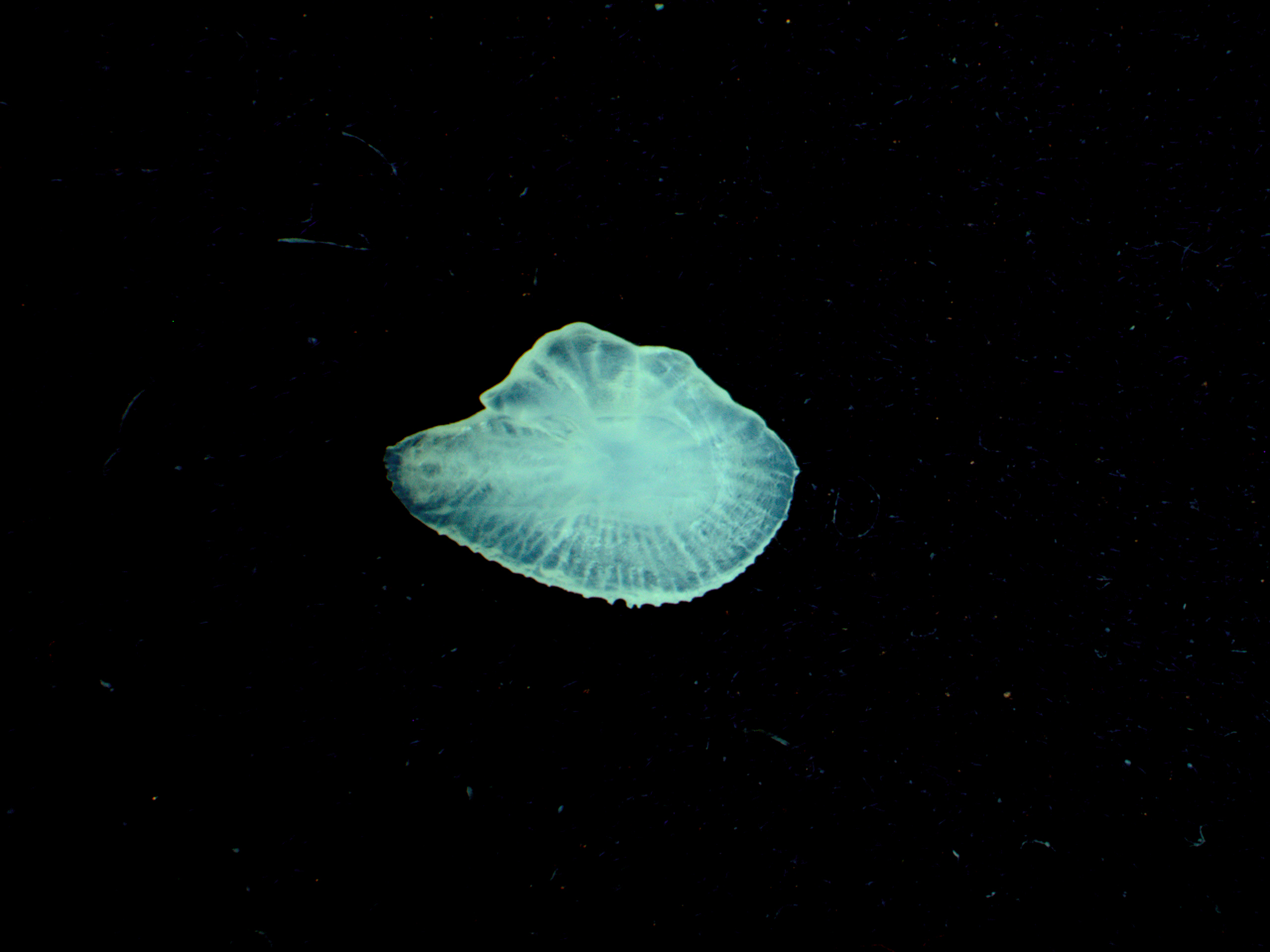

Supplement: Supplemental Information 9 [file peerj-04-1664-s009.zip › Setipinna/training/Eng214R1.jpg]

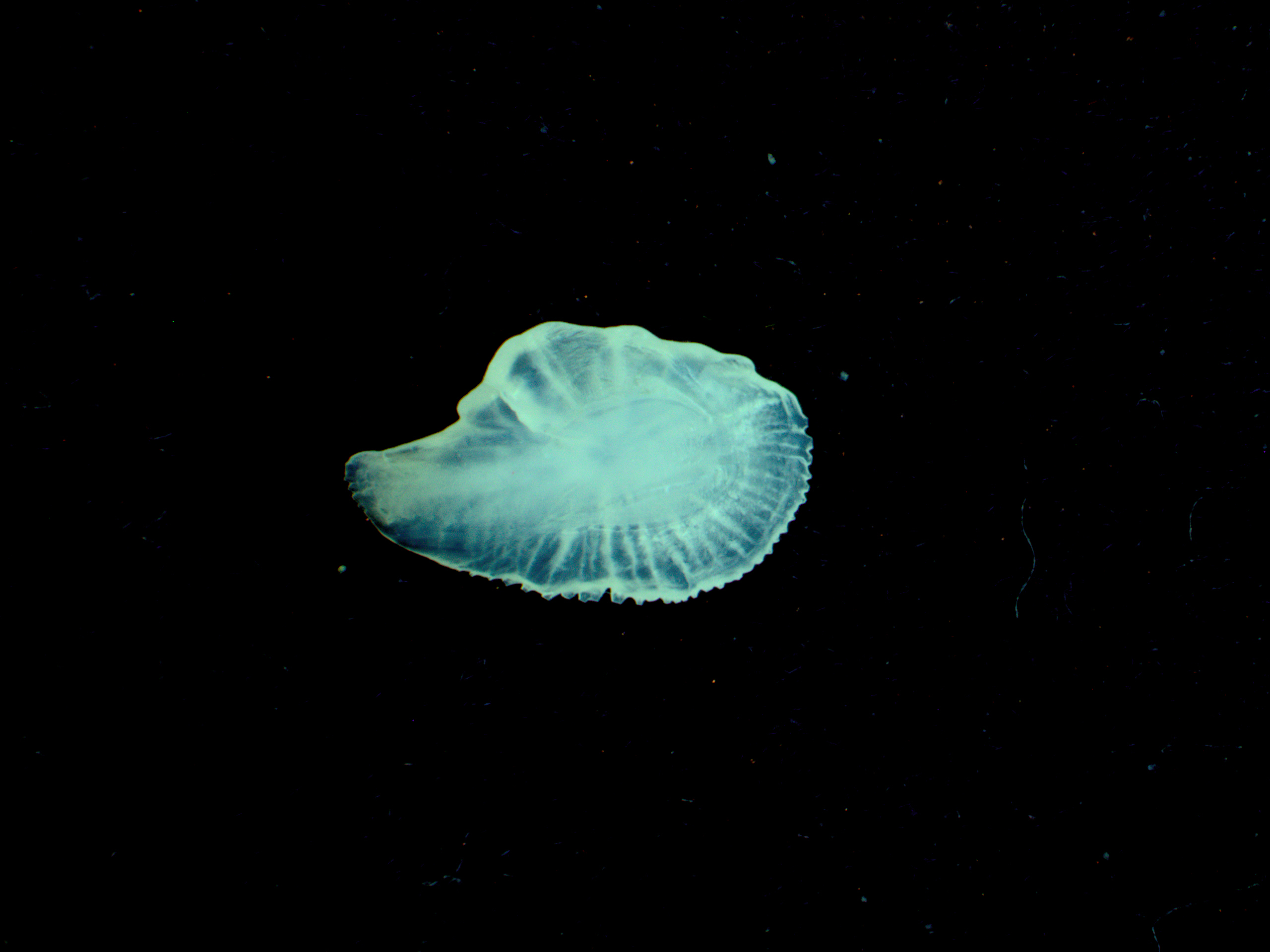

Supplement: Supplemental Information 9 [file peerj-04-1664-s009.zip › Setipinna/training/Eng215R1.jpg]

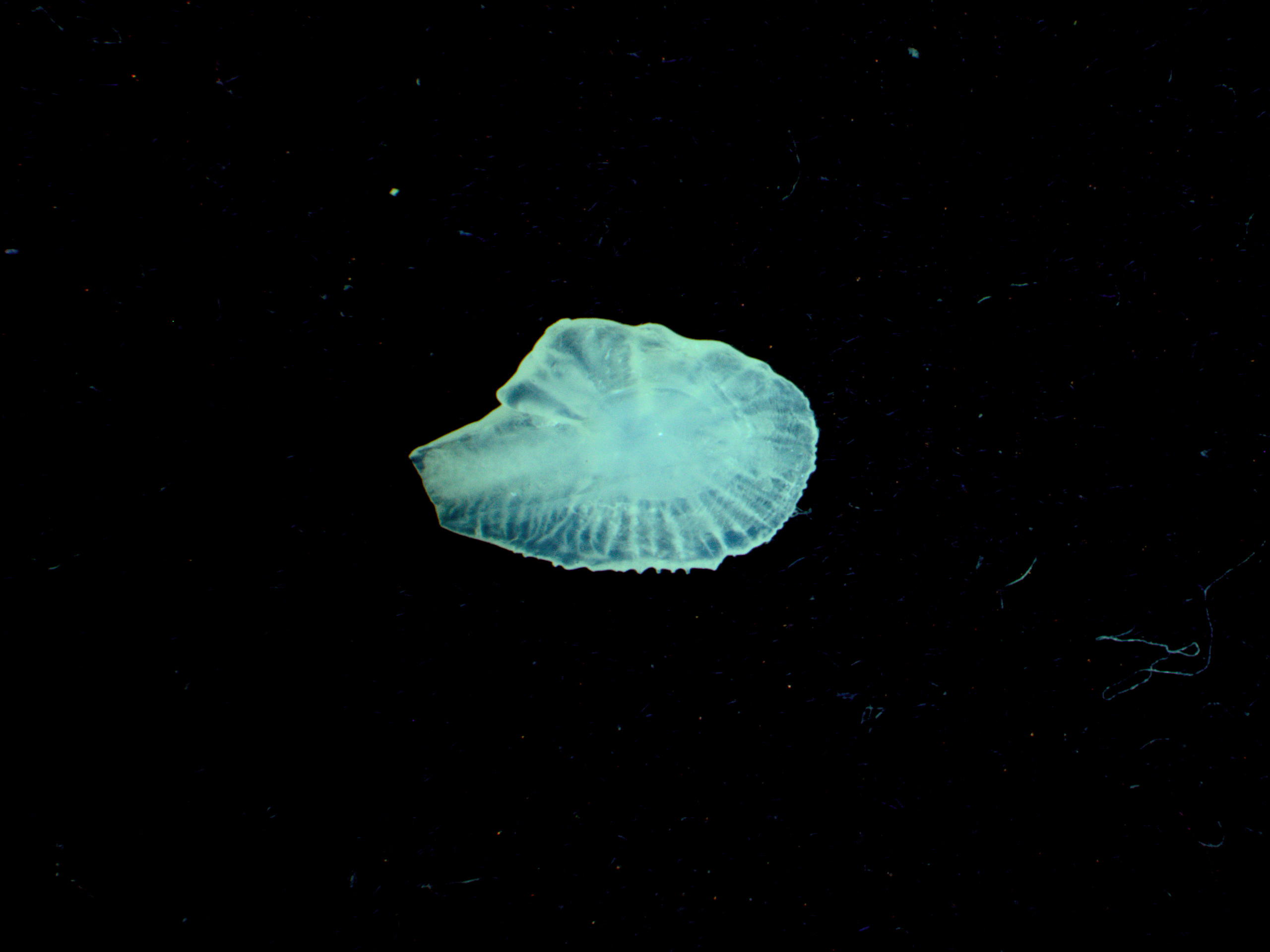

Supplement: Supplemental Information 9 [file peerj-04-1664-s009.zip › Setipinna/training/Eng216R1.jpg]

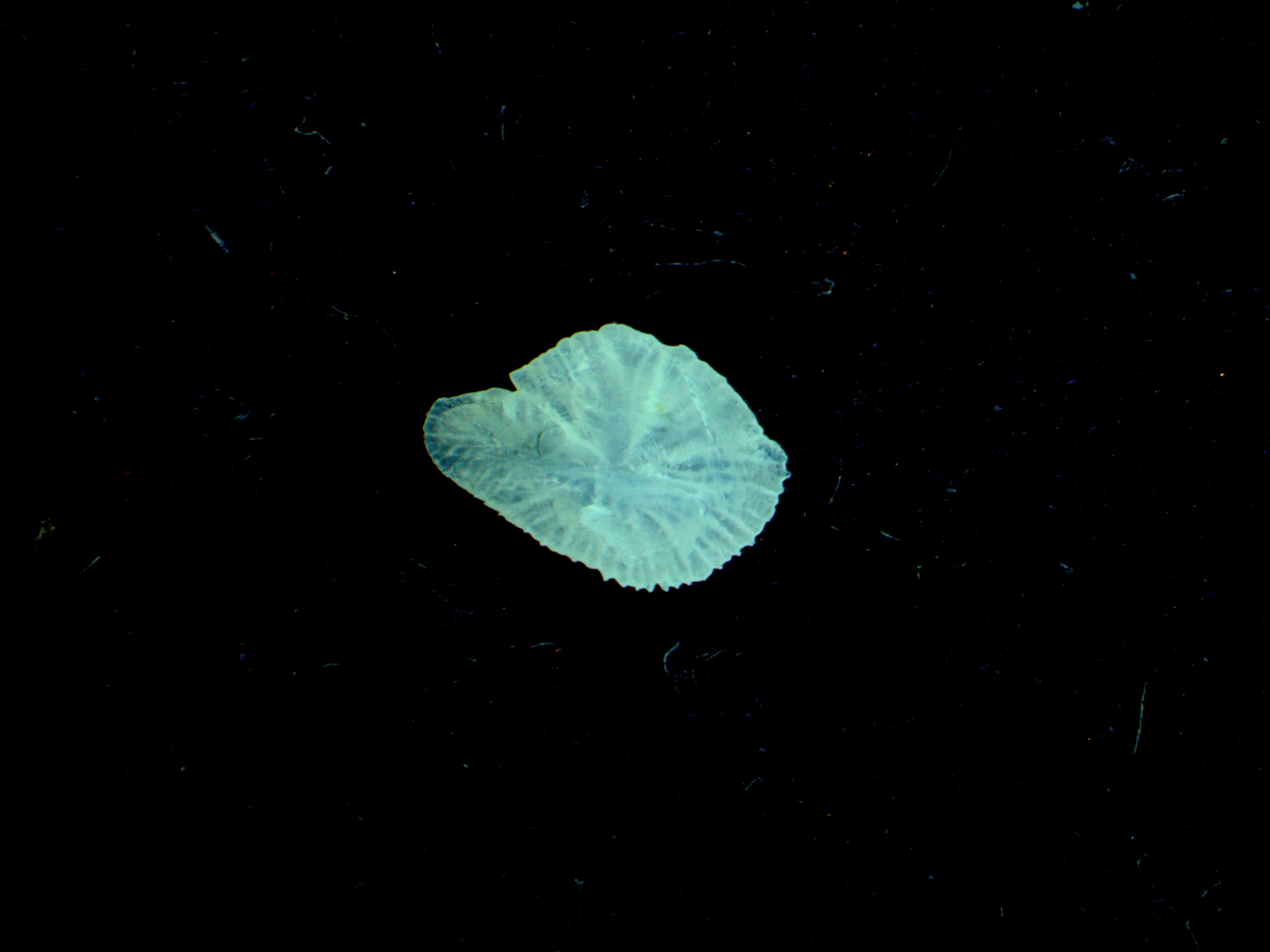

Supplement: Supplemental Information 10 [file peerj-04-1664-s010.zip › Thryssa/testing/Eng238R1.jpg]

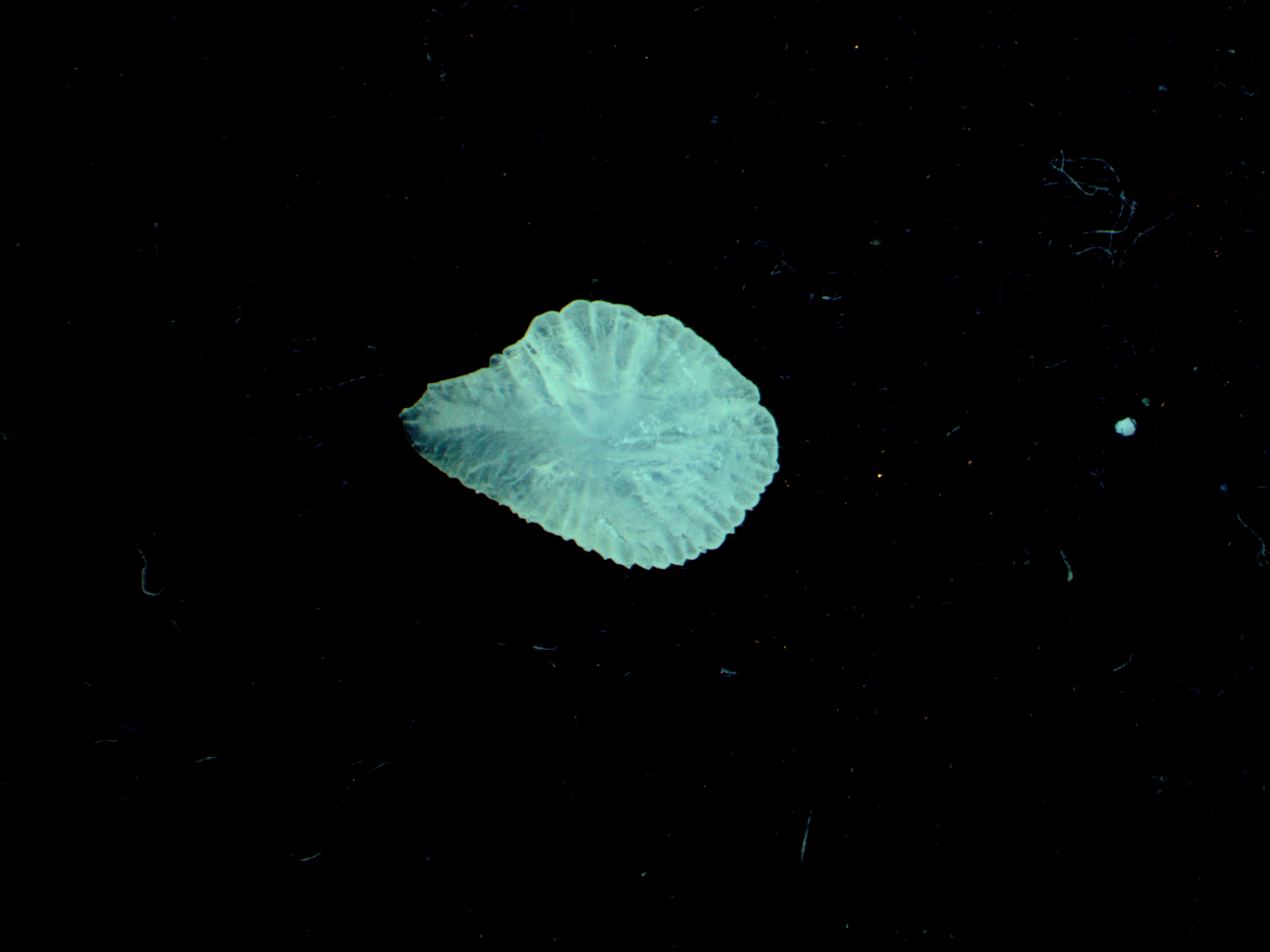

Supplement: Supplemental Information 10 [file peerj-04-1664-s010.zip › Thryssa/testing/Eng239R1.jpg]

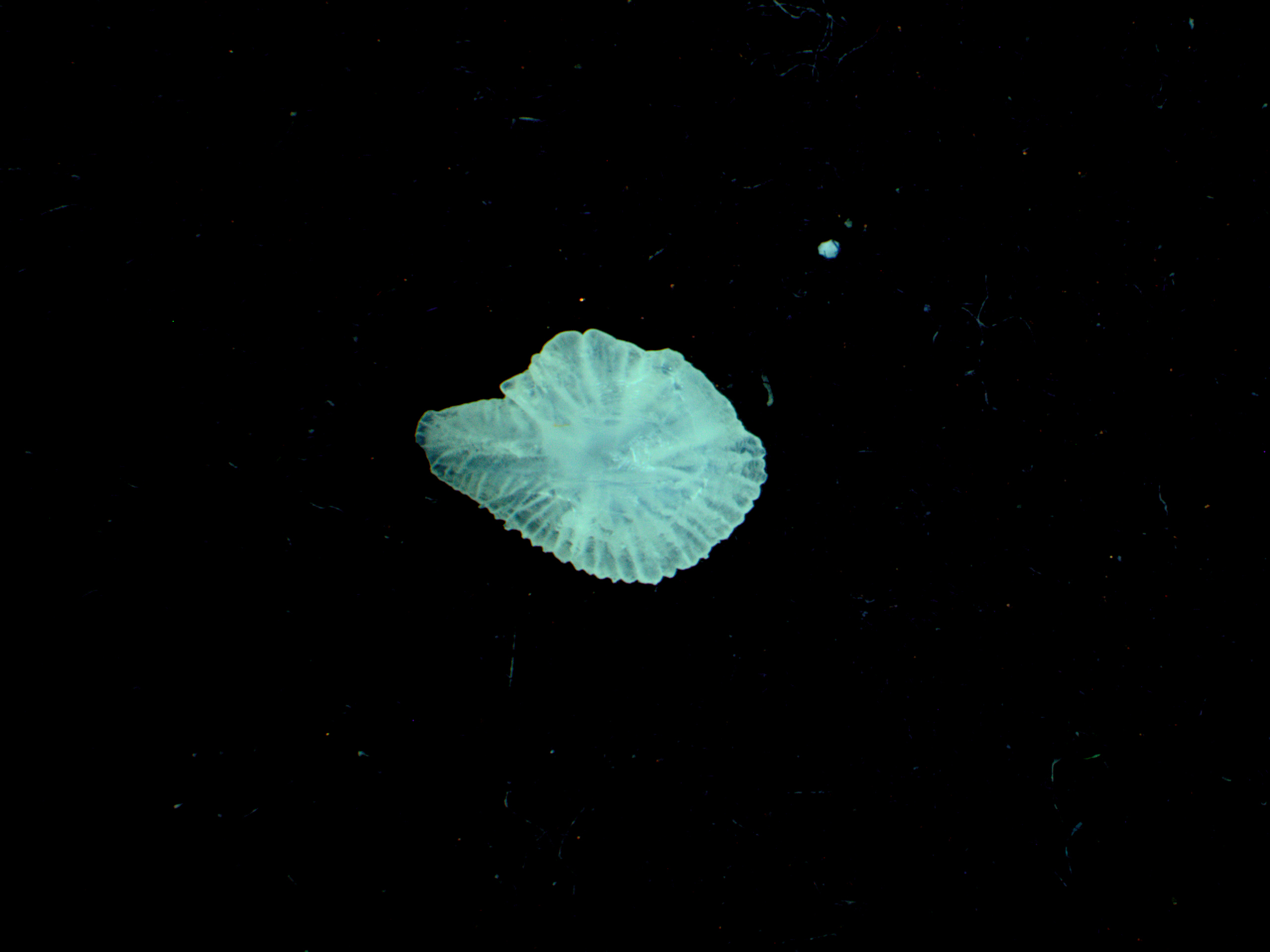

Supplement: Supplemental Information 10 [file peerj-04-1664-s010.zip › Thryssa/testing/Eng240R1.jpg]

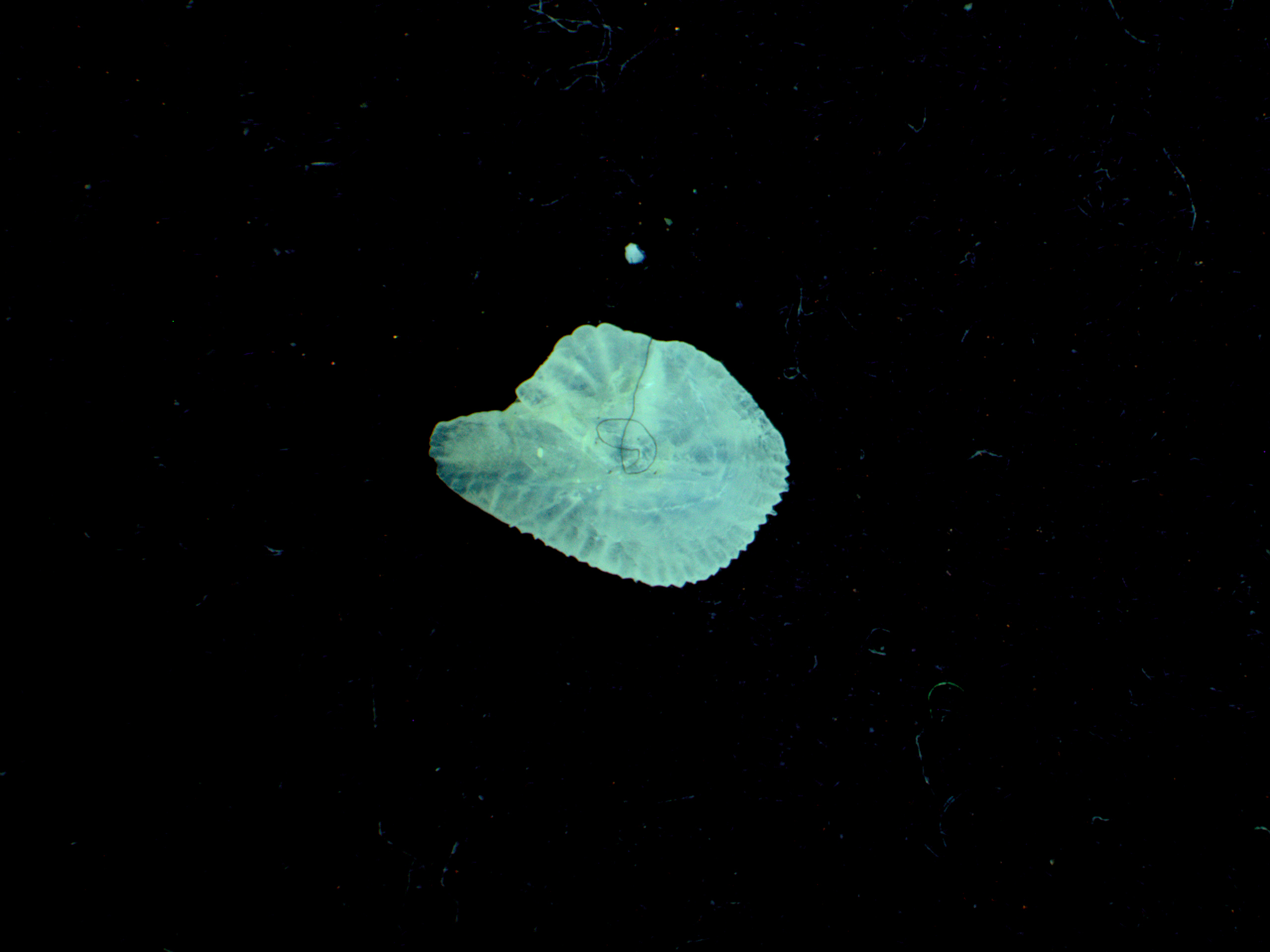

Supplement: Supplemental Information 10 [file peerj-04-1664-s010.zip › Thryssa/testing/Eng241R1.jpg]

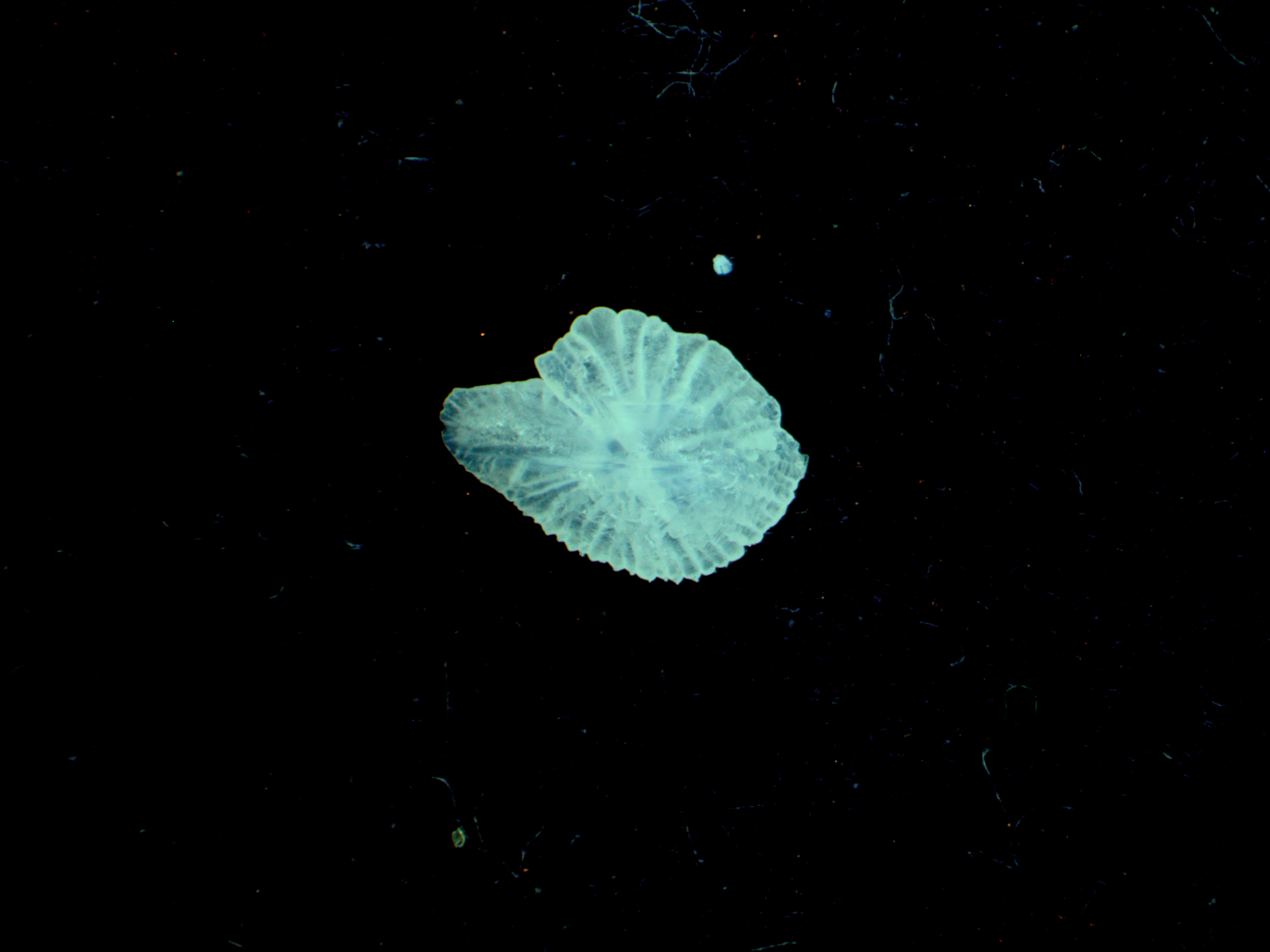

Supplement: Supplemental Information 10 [file peerj-04-1664-s010.zip › Thryssa/testing/Eng242R1.jpg]

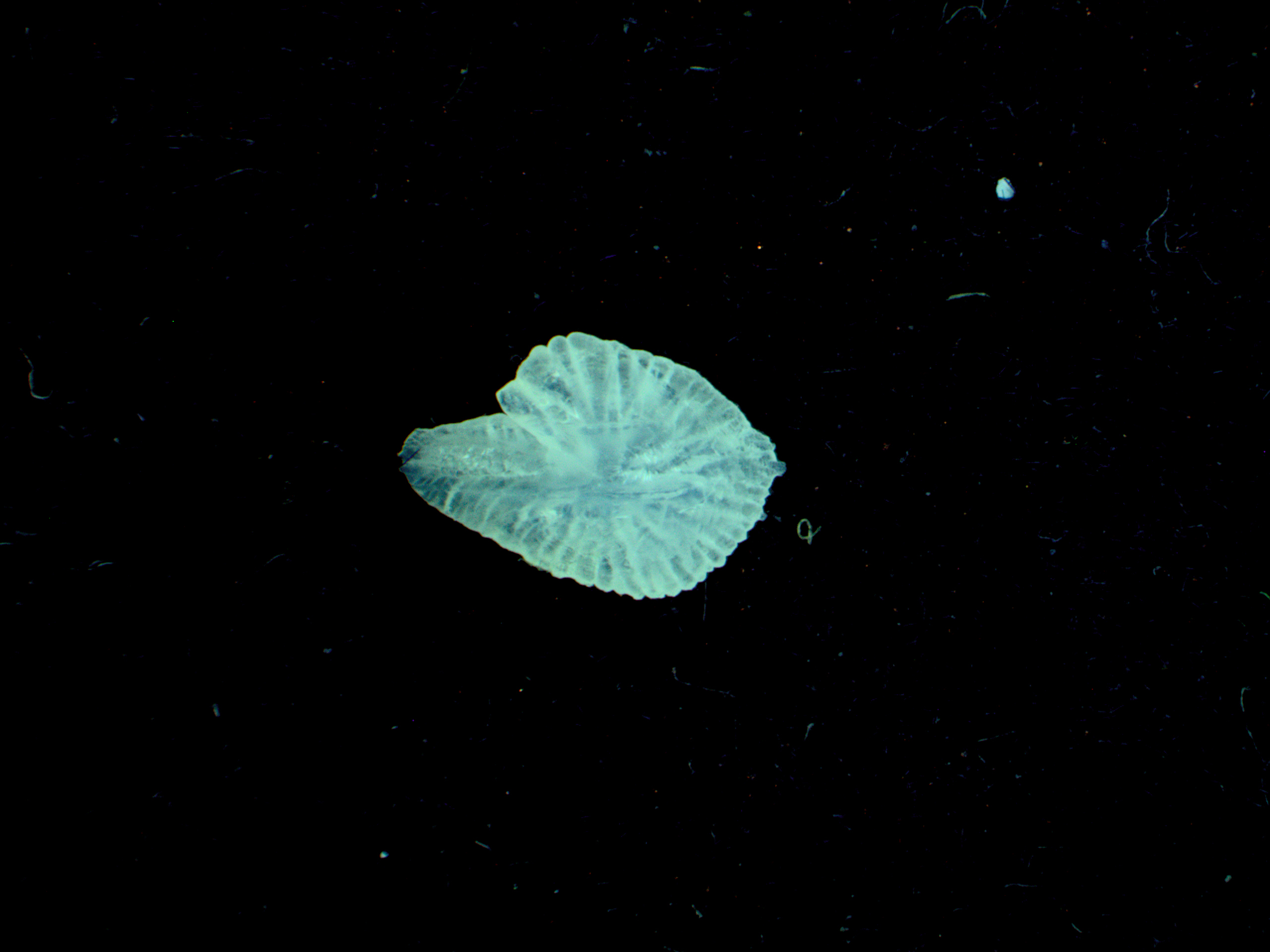

Supplement: Supplemental Information 10 [file peerj-04-1664-s010.zip › Thryssa/testing/Eng243R1.jpg]

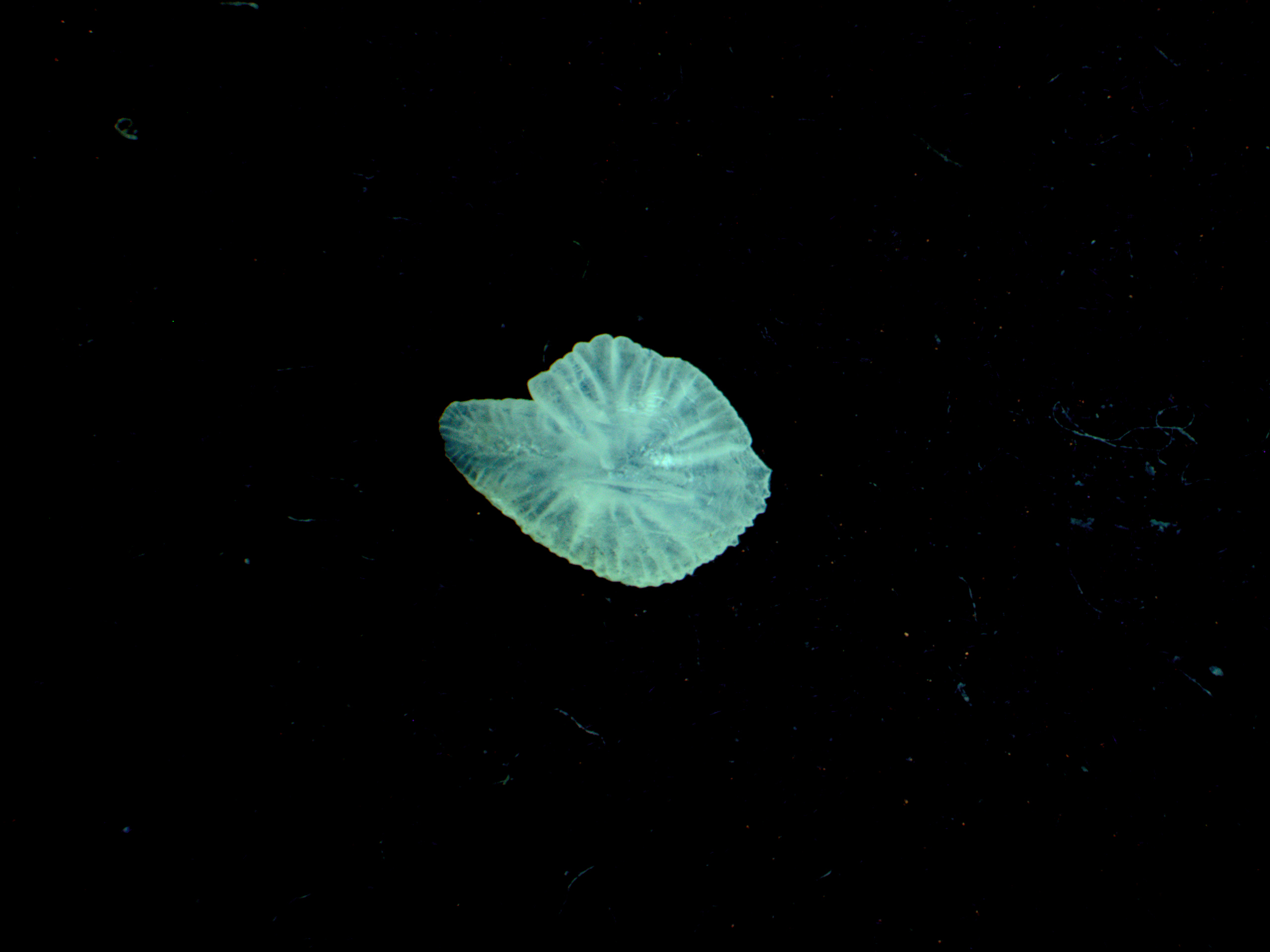

Supplement: Supplemental Information 10 [file peerj-04-1664-s010.zip › Thryssa/testing/Eng244R1.jpg]

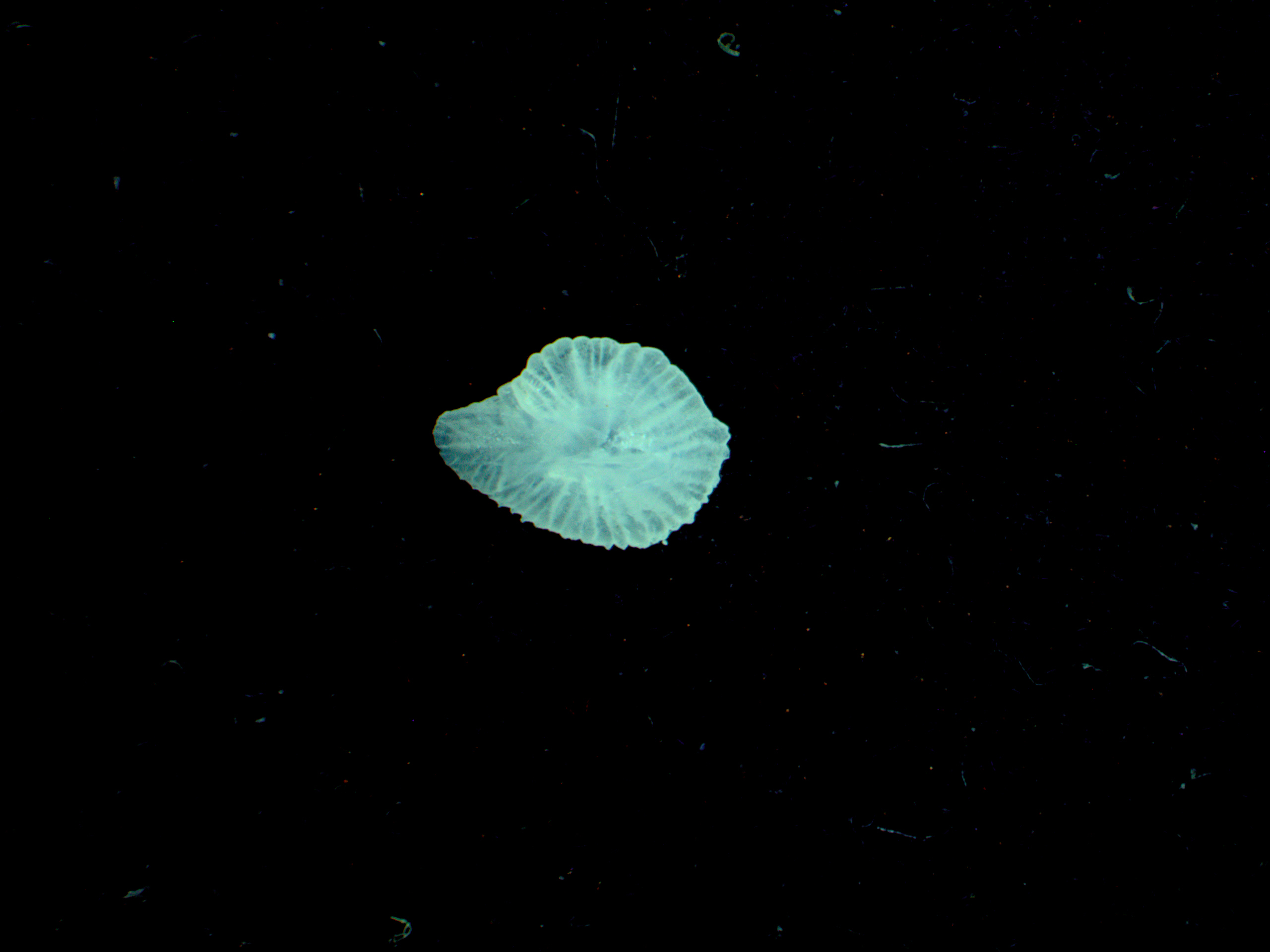

Supplement: Supplemental Information 10 [file peerj-04-1664-s010.zip › Thryssa/testing/Eng245R1.jpg]

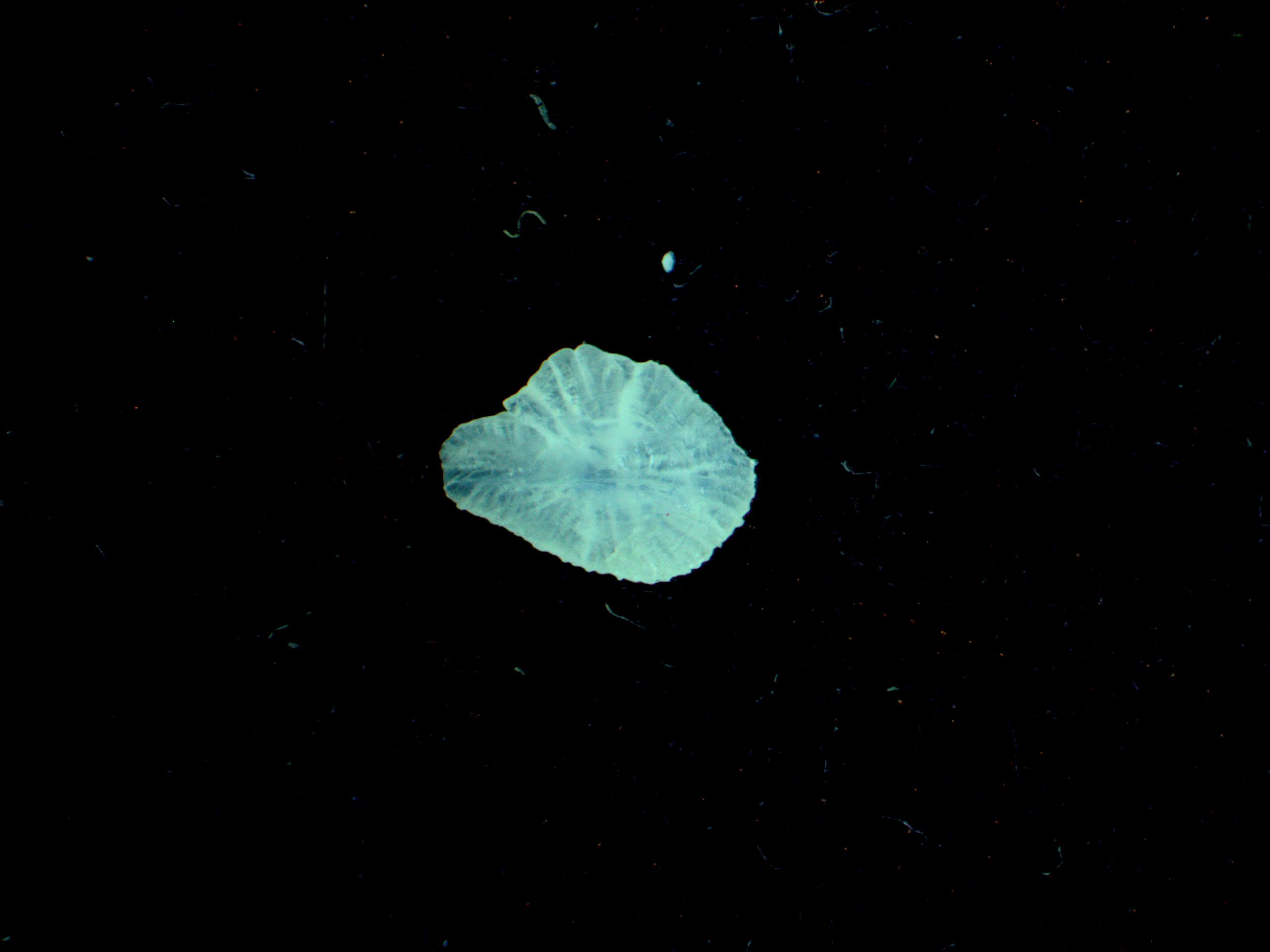

Supplement: Supplemental Information 10 [file peerj-04-1664-s010.zip › Thryssa/testing/Eng246R1.jpg]

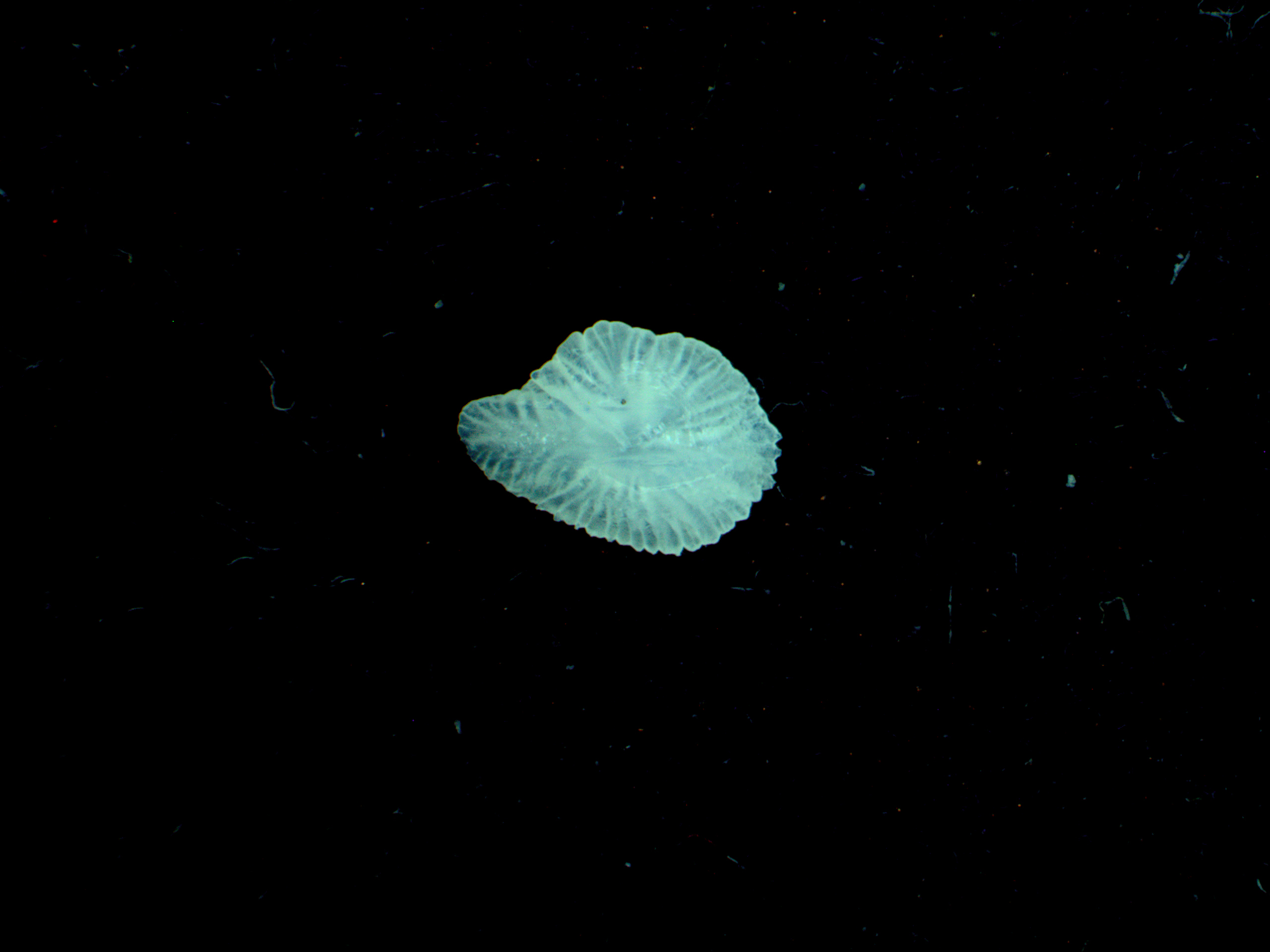

Supplement: Supplemental Information 10 [file peerj-04-1664-s010.zip › Thryssa/testing/Eng247R1.jpg]

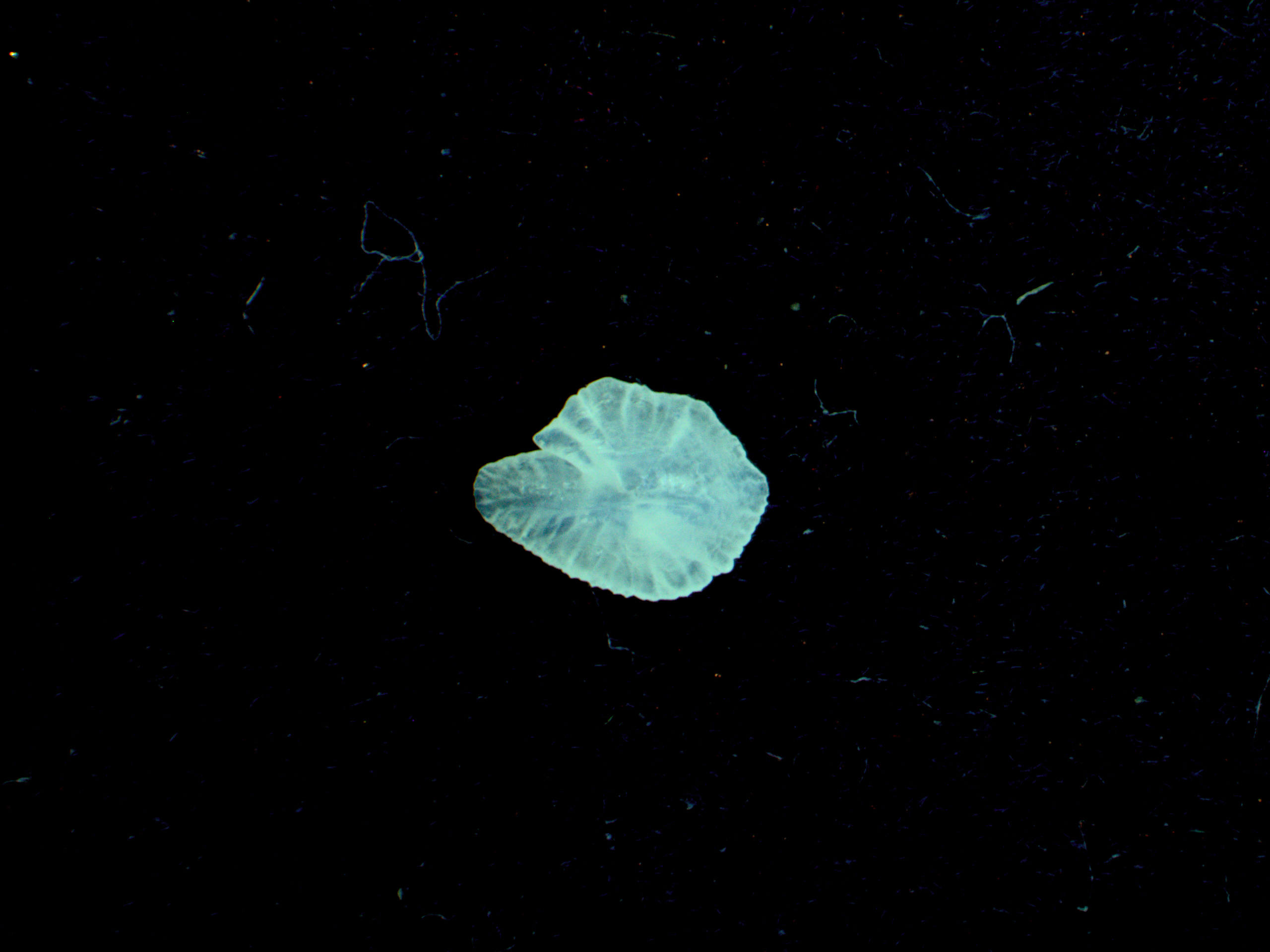

Supplement: Supplemental Information 10 [file peerj-04-1664-s010.zip › Thryssa/training/Eng218R1.jpg]

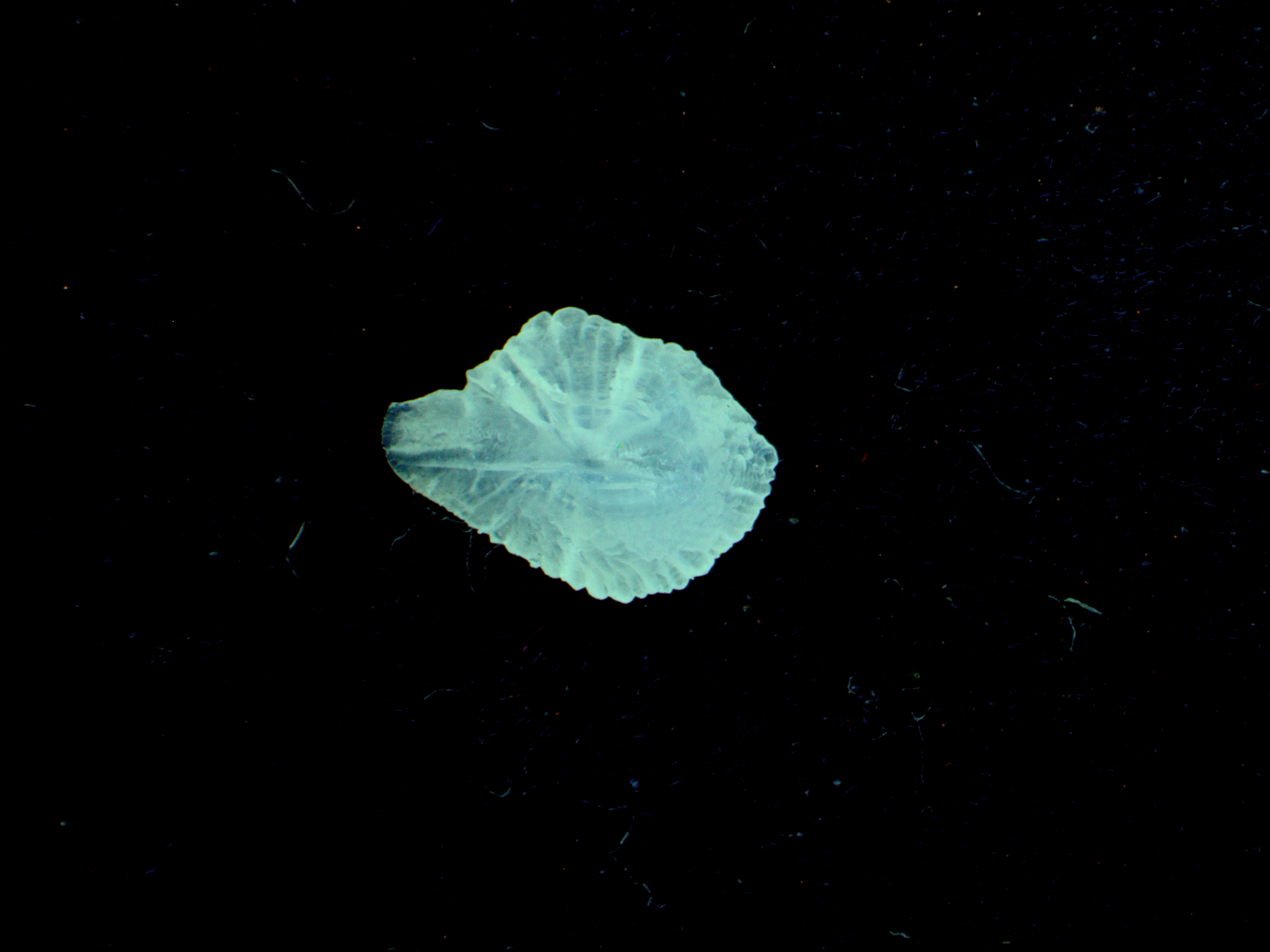

Supplement: Supplemental Information 10 [file peerj-04-1664-s010.zip › Thryssa/training/Eng219R1.jpg]

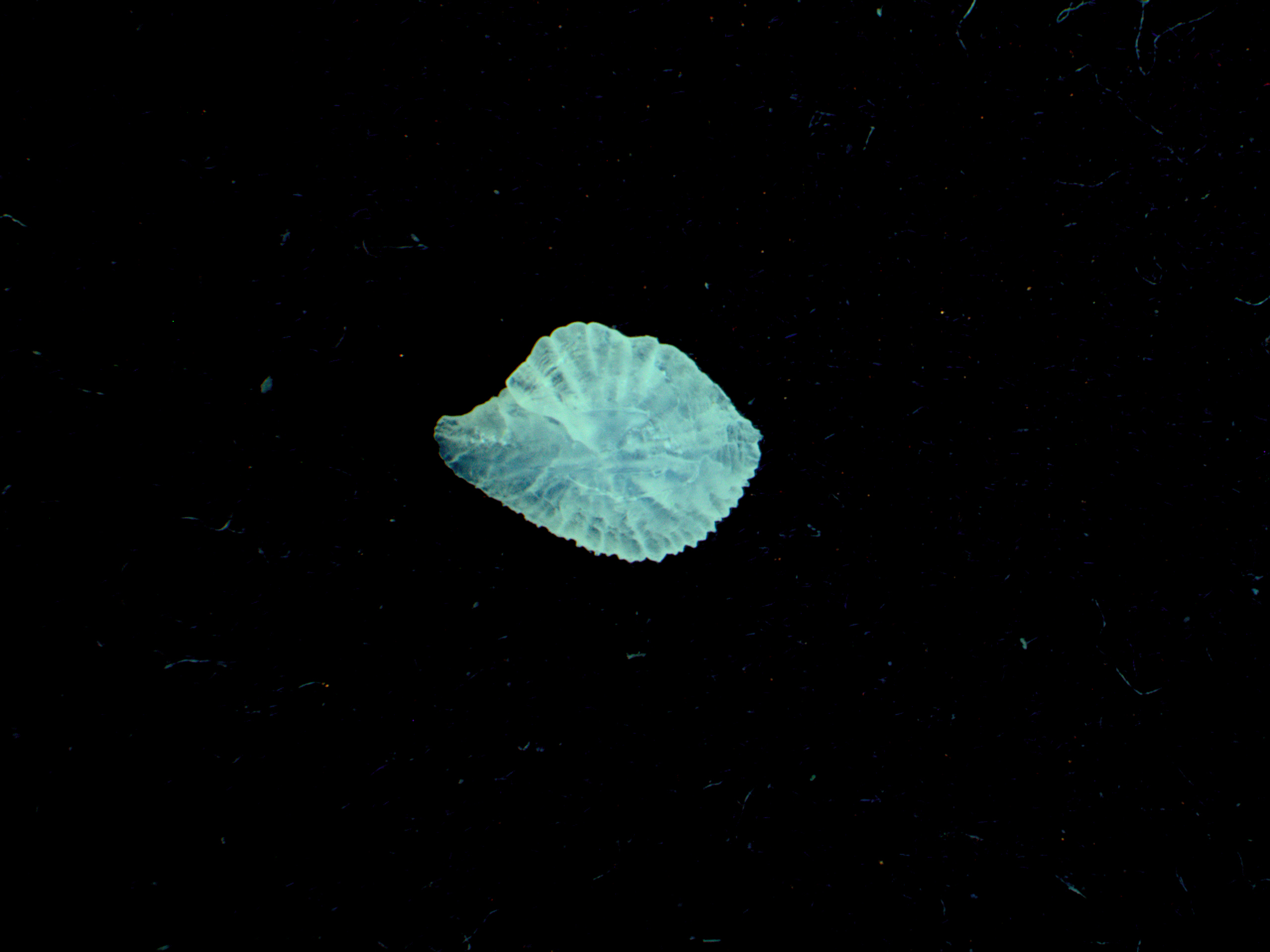

Supplement: Supplemental Information 10 [file peerj-04-1664-s010.zip › Thryssa/training/Eng220R1.jpg]

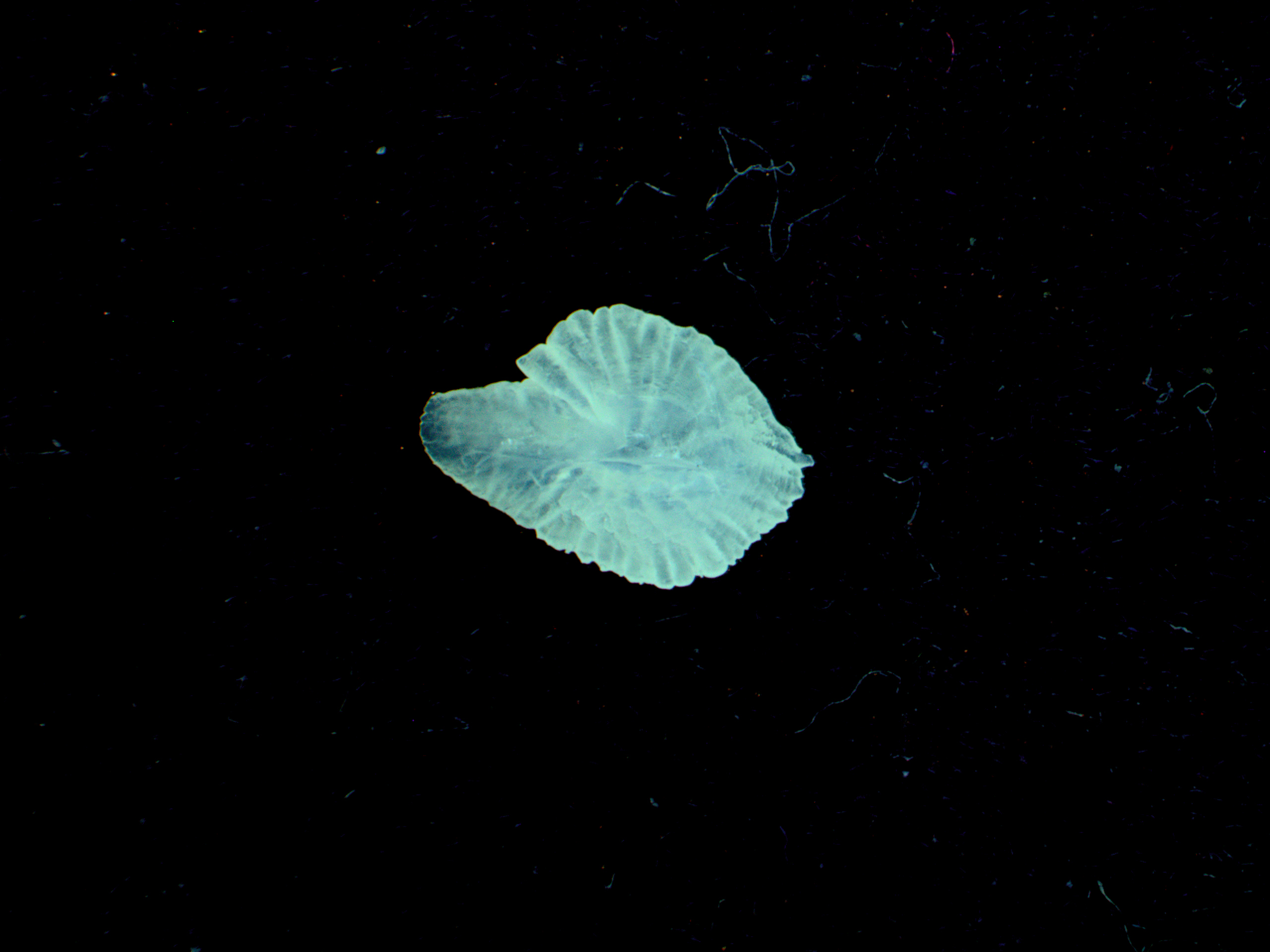

Supplement: Supplemental Information 10 [file peerj-04-1664-s010.zip › Thryssa/training/Eng221R1.jpg]

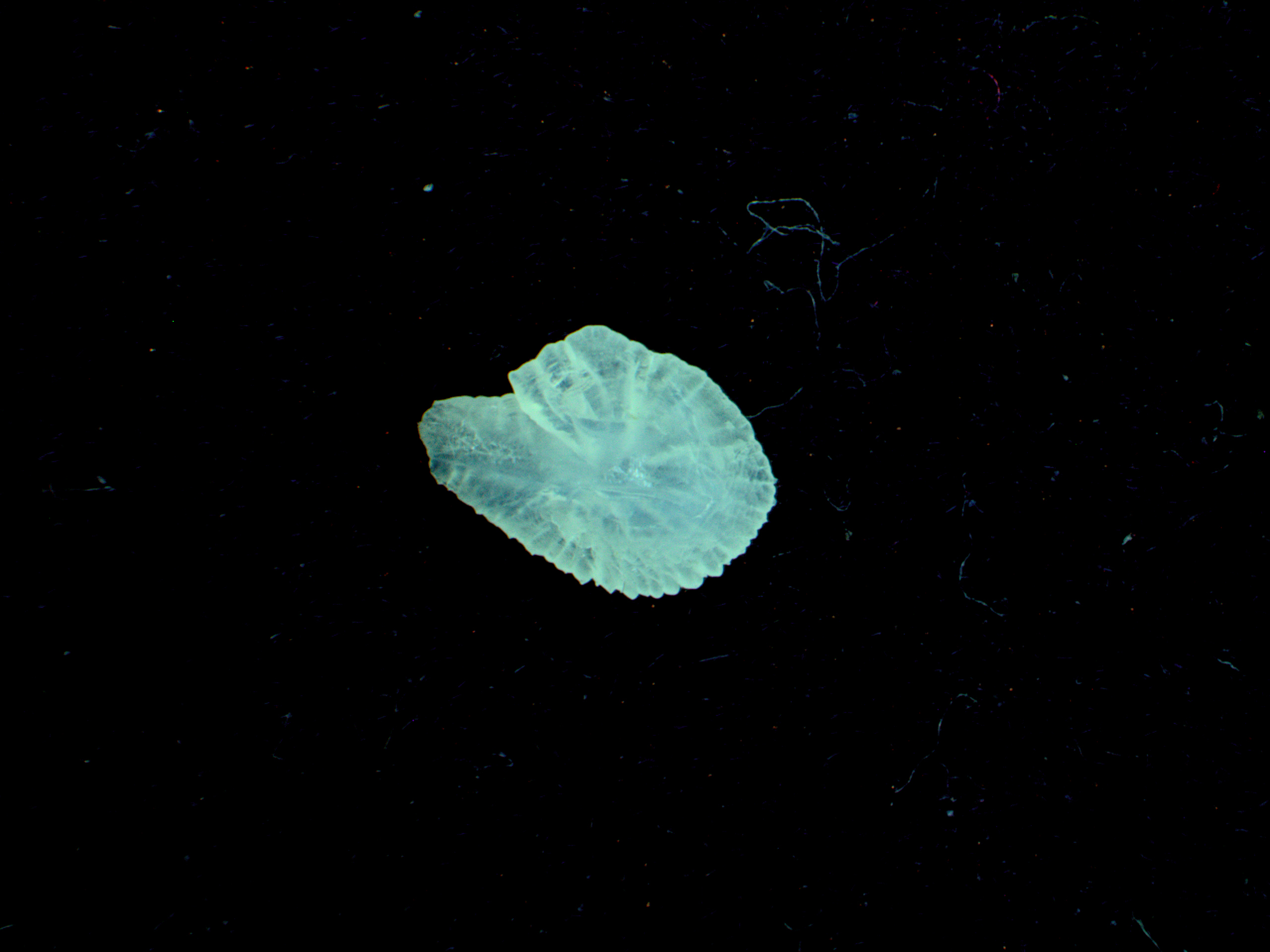

Supplement: Supplemental Information 10 [file peerj-04-1664-s010.zip › Thryssa/training/Eng222R1.jpg]

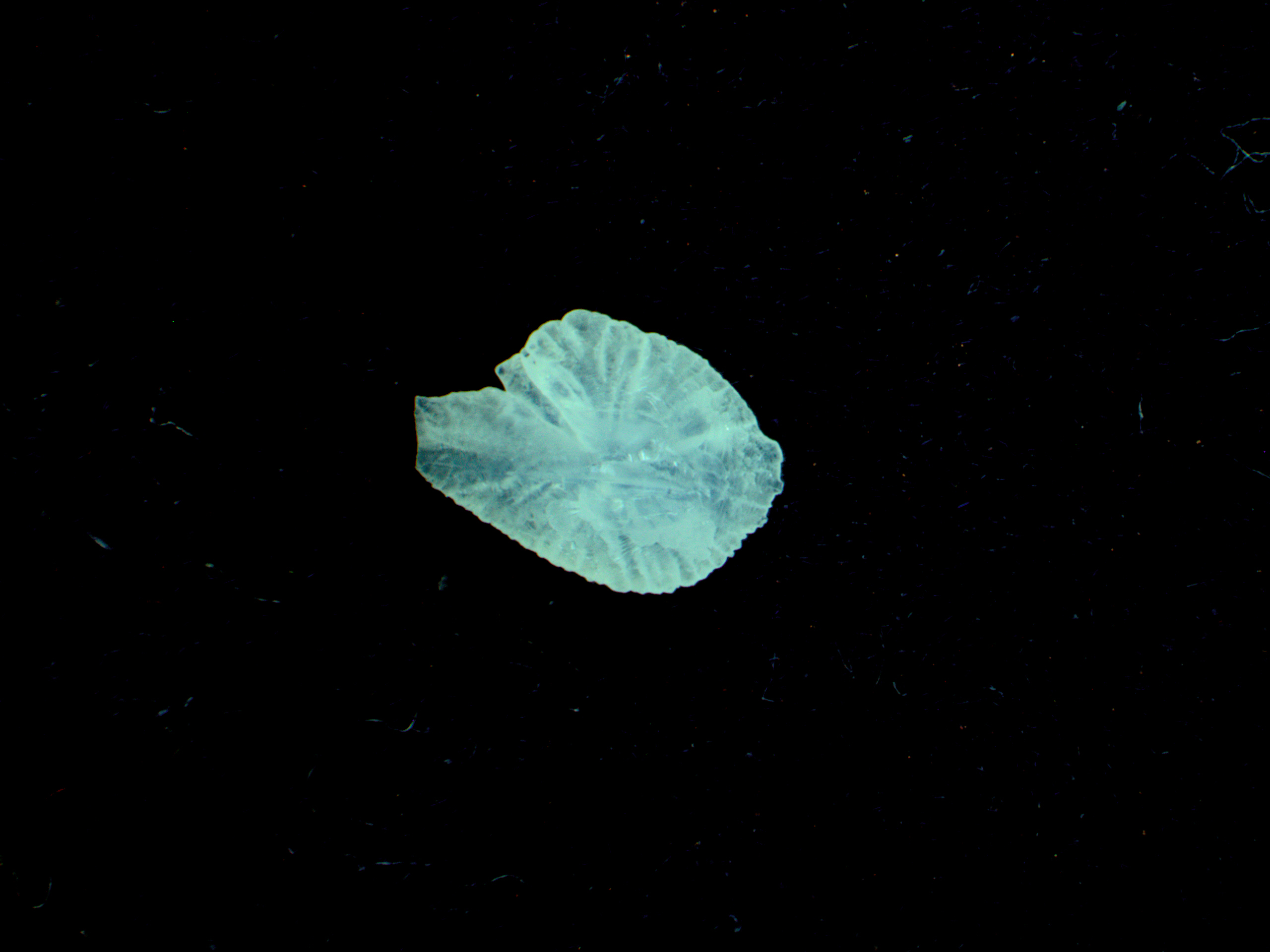

Supplement: Supplemental Information 10 [file peerj-04-1664-s010.zip › Thryssa/training/Eng223R1.jpg]

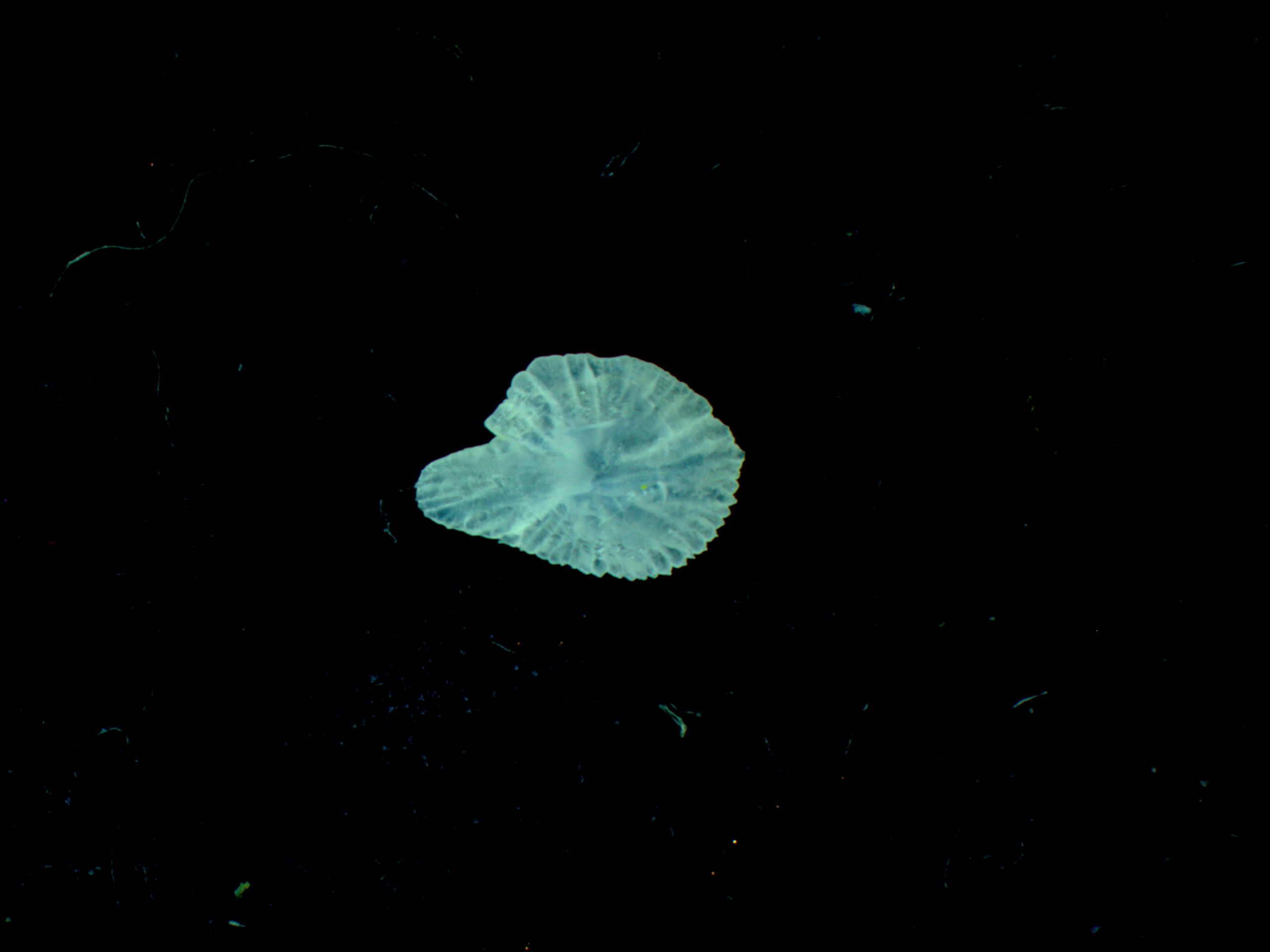

Supplement: Supplemental Information 10 [file peerj-04-1664-s010.zip › Thryssa/training/Eng224R1.jpg]

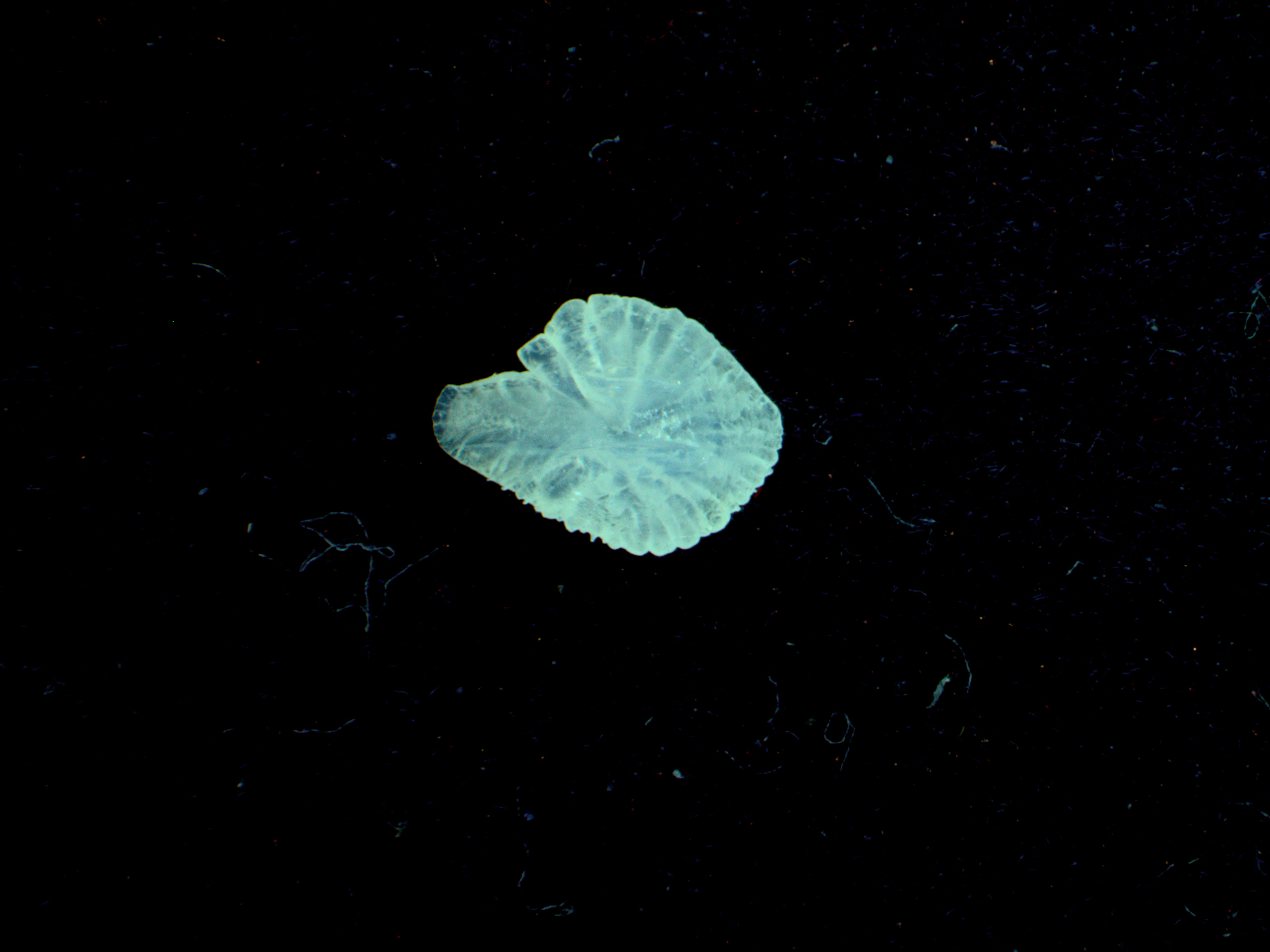

Supplement: Supplemental Information 10 [file peerj-04-1664-s010.zip › Thryssa/training/Eng225R1.jpg]

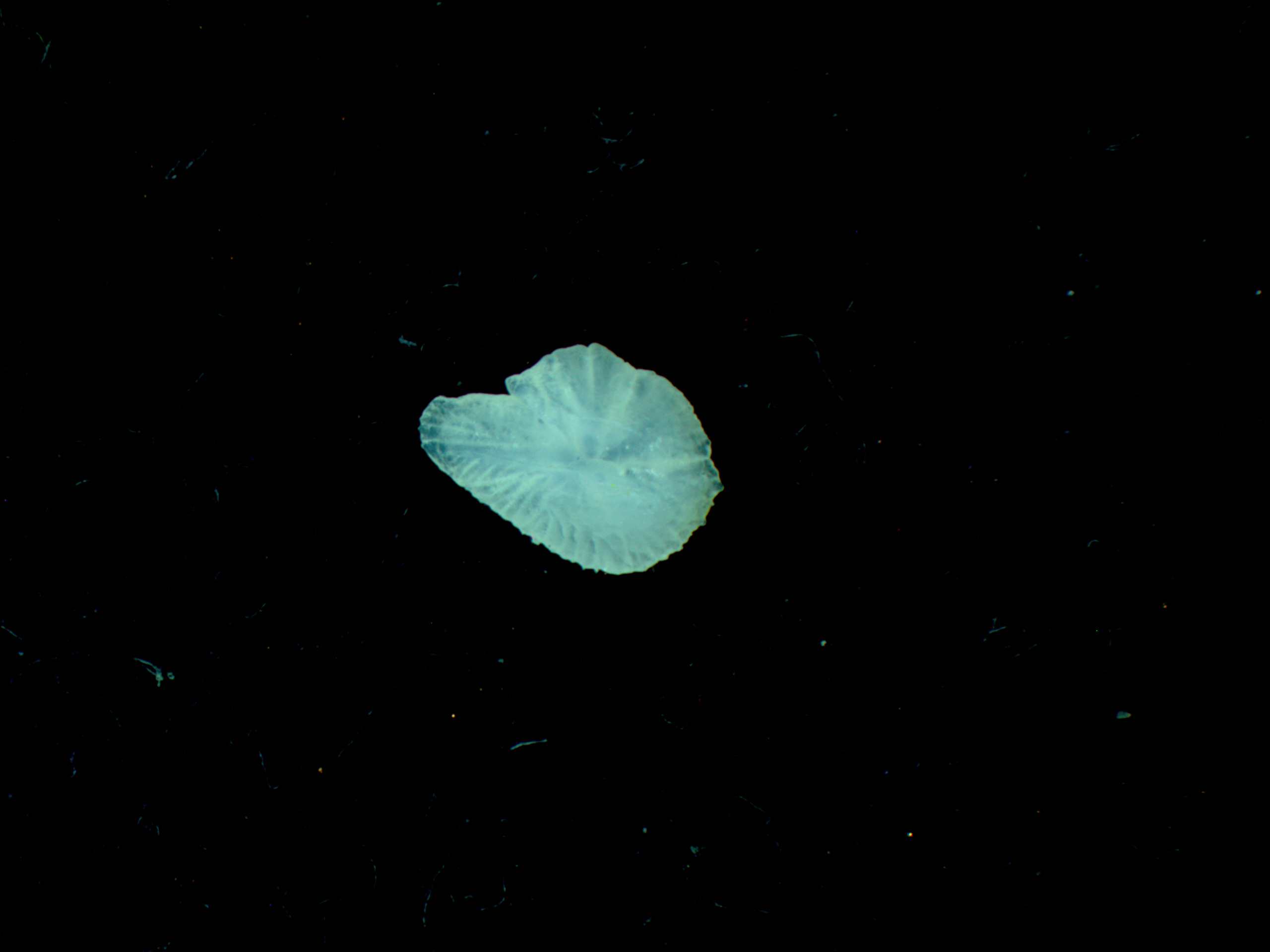

Supplement: Supplemental Information 10 [file peerj-04-1664-s010.zip › Thryssa/training/Eng226R1.jpg]

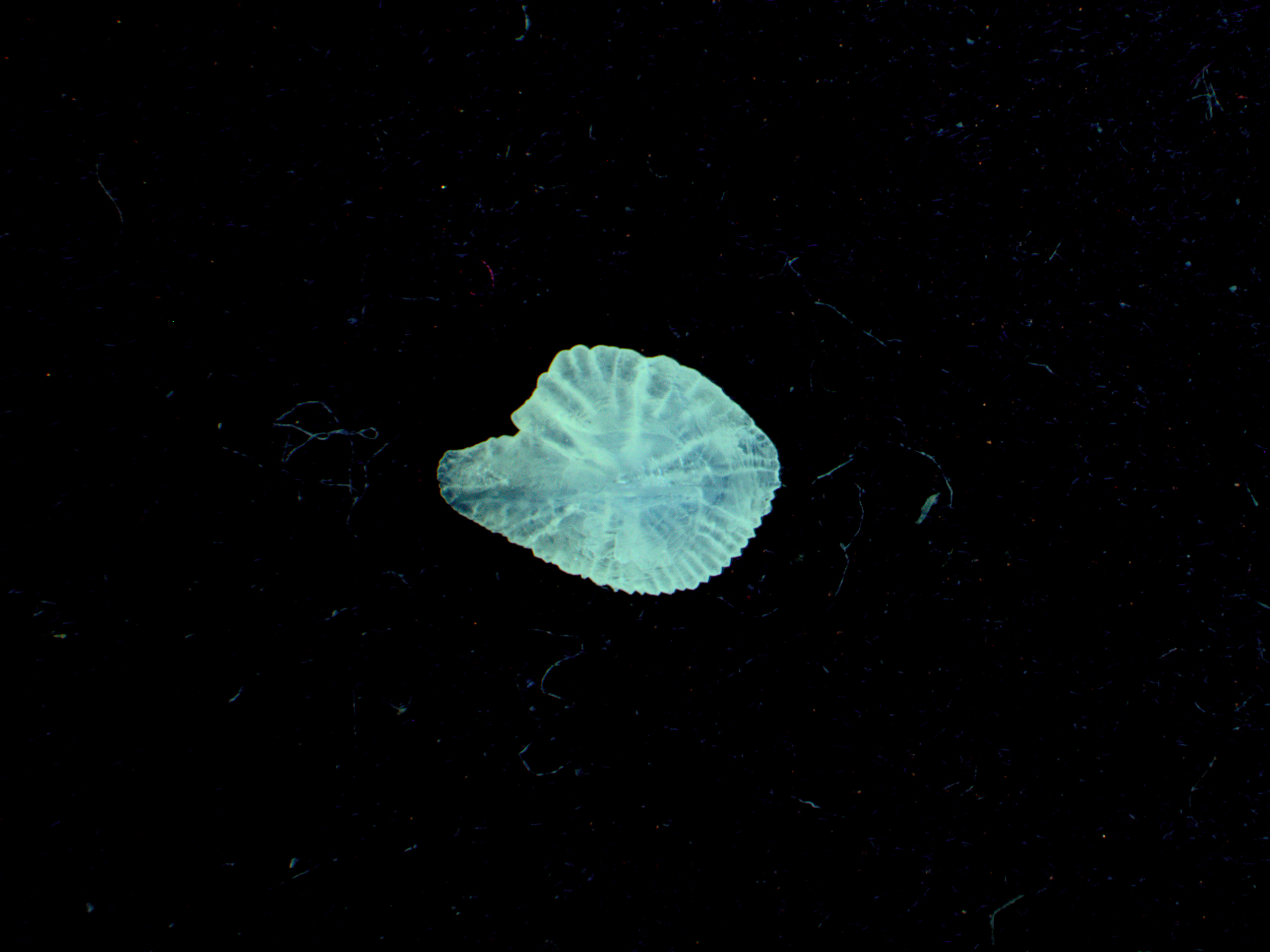

Supplement: Supplemental Information 10 [file peerj-04-1664-s010.zip › Thryssa/training/Eng227R1.jpg]

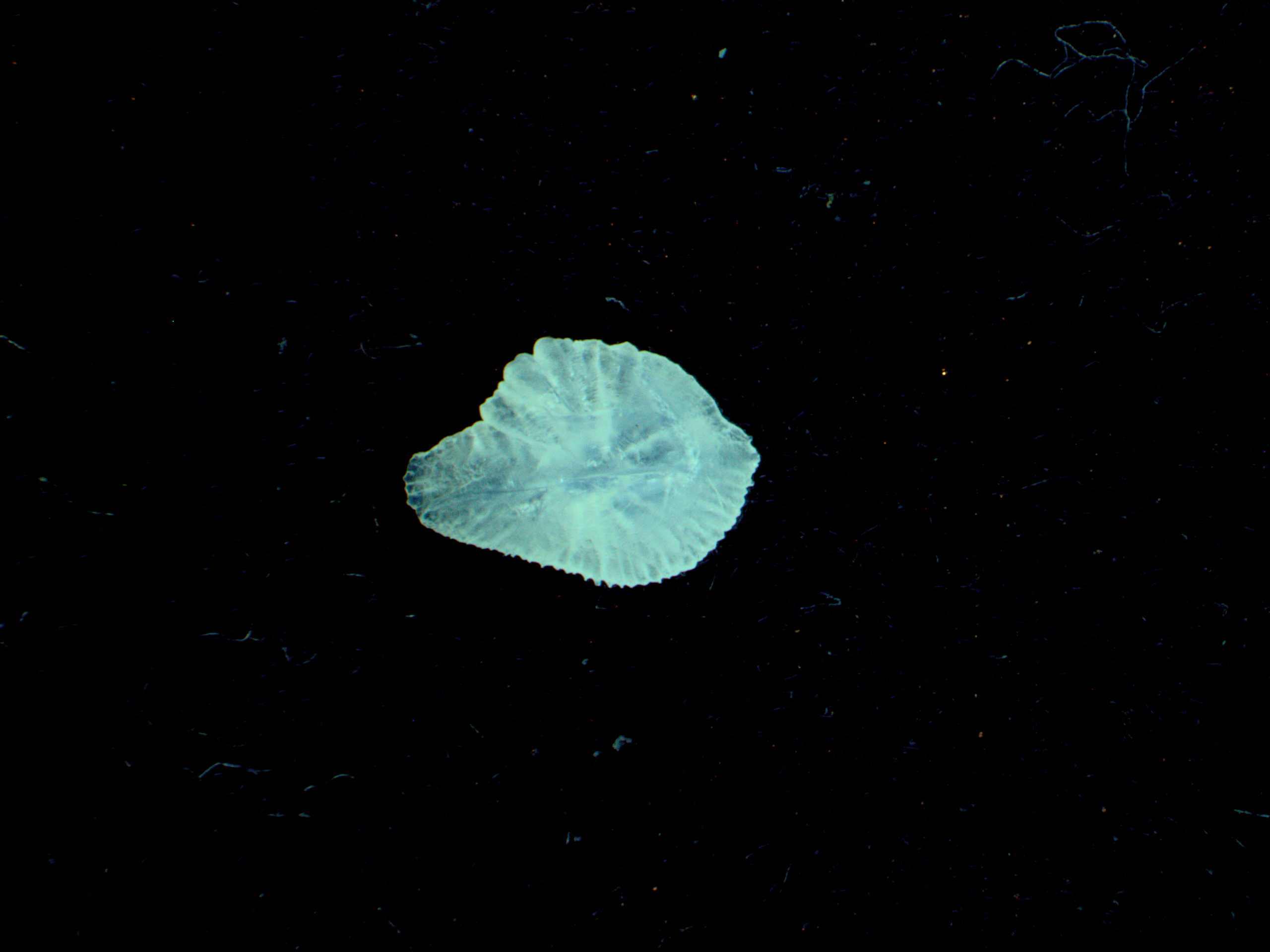

Supplement: Supplemental Information 10 [file peerj-04-1664-s010.zip › Thryssa/training/Eng228R1.jpg]

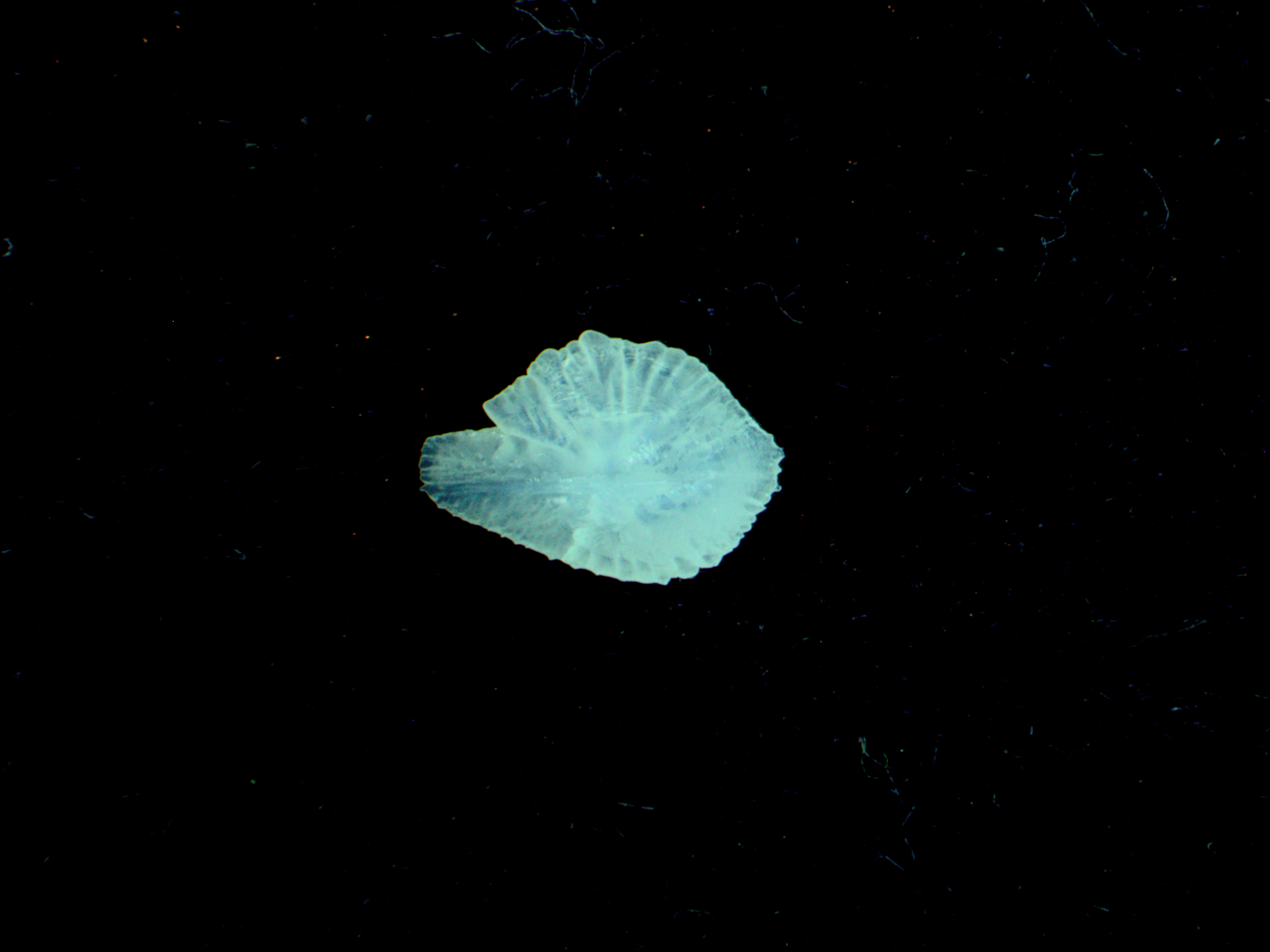

Supplement: Supplemental Information 10 [file peerj-04-1664-s010.zip › Thryssa/training/Eng229R1.jpg]

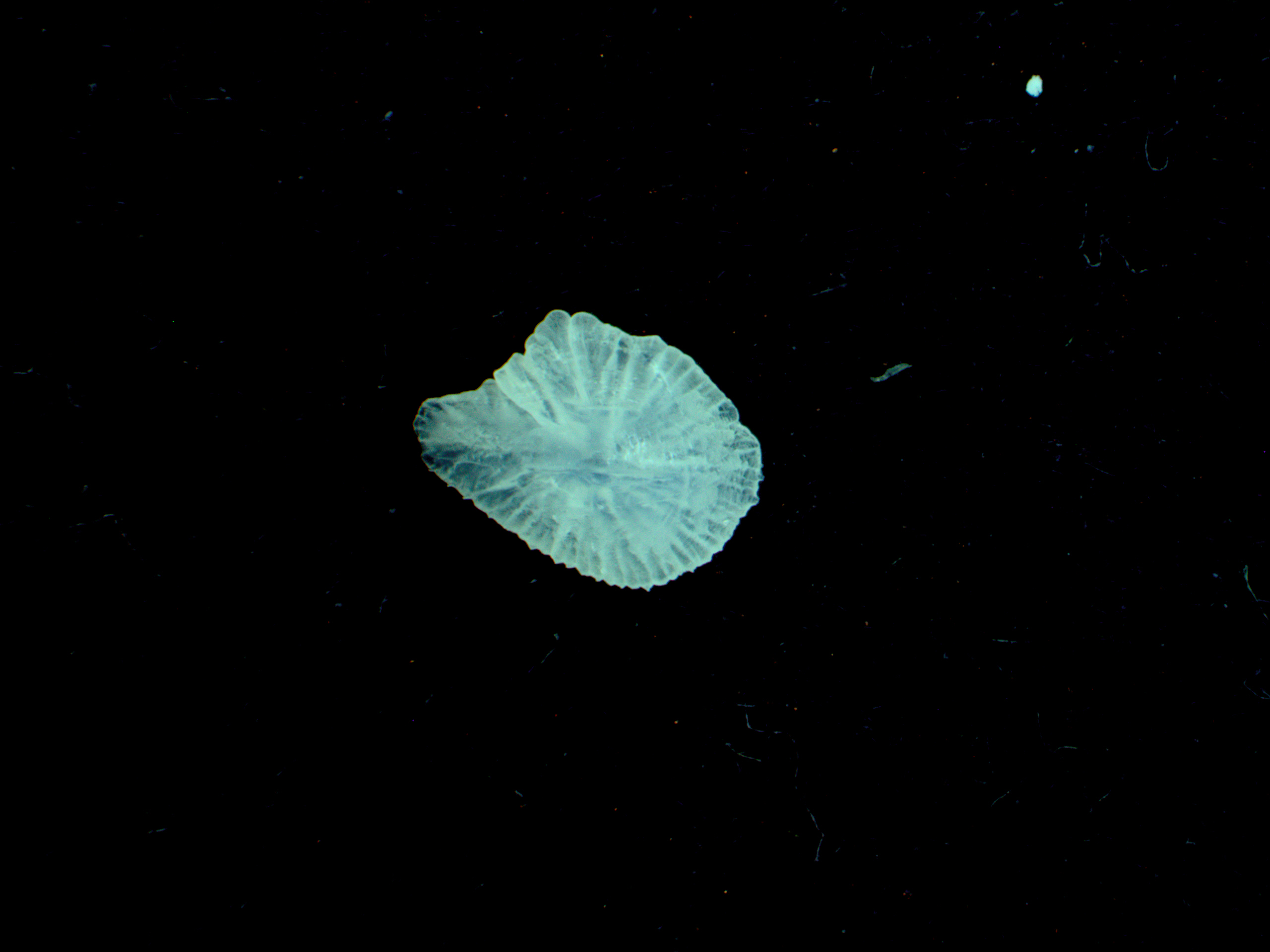

Supplement: Supplemental Information 10 [file peerj-04-1664-s010.zip › Thryssa/training/Eng230R1.jpg]

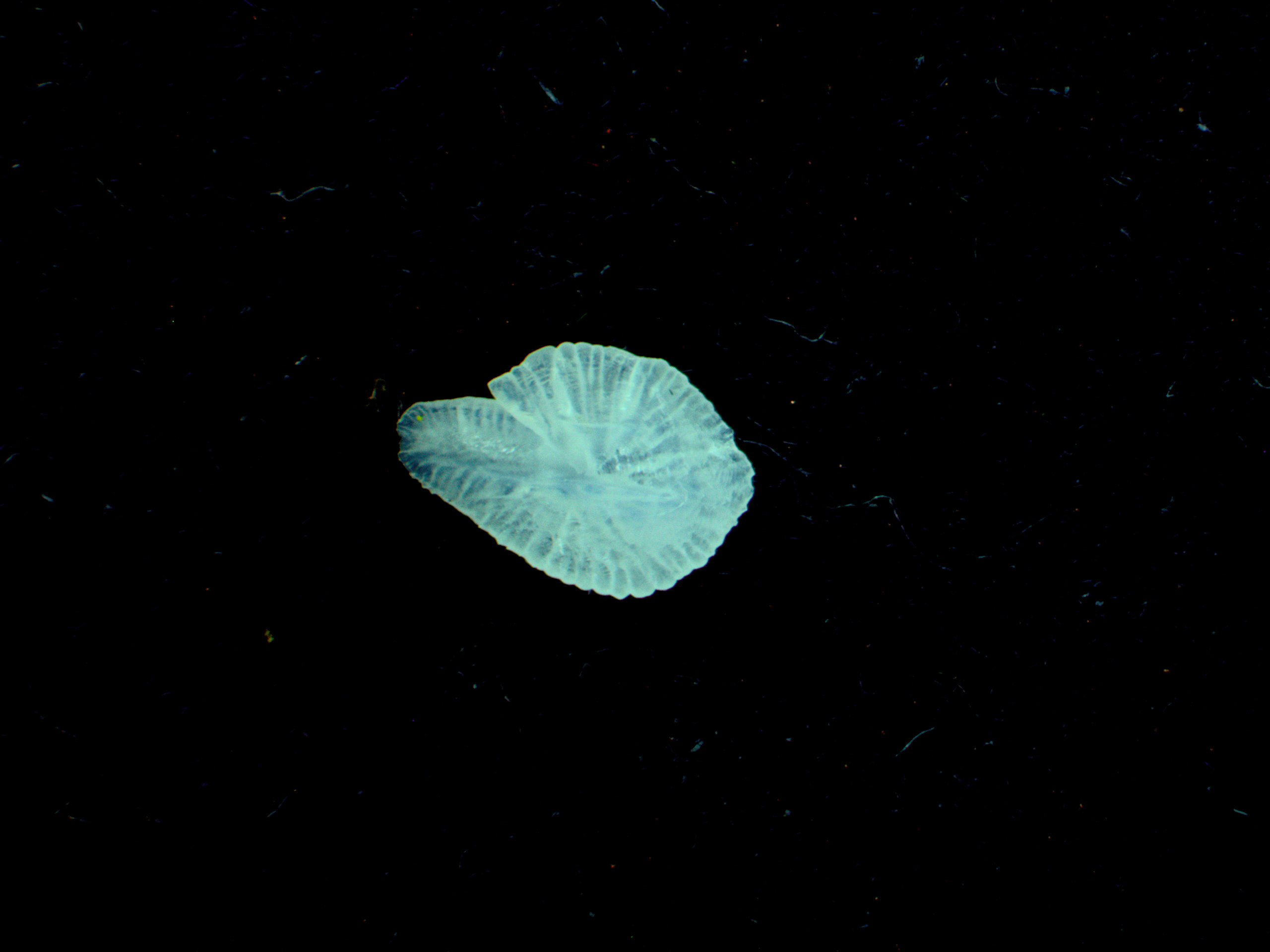

Supplement: Supplemental Information 10 [file peerj-04-1664-s010.zip › Thryssa/training/Eng231R1.jpg]

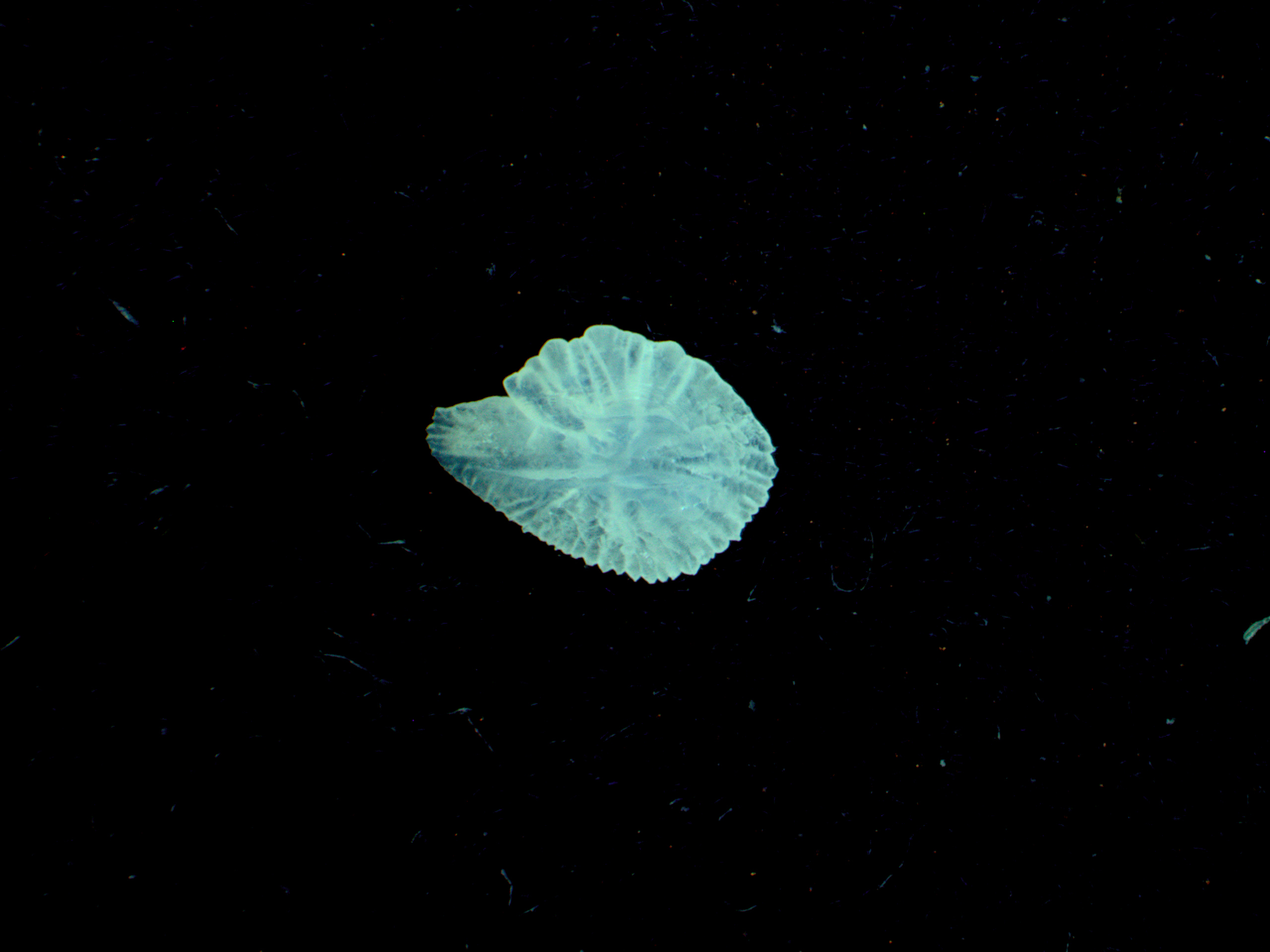

Supplement: Supplemental Information 10 [file peerj-04-1664-s010.zip › Thryssa/training/Eng232R1.jpg]

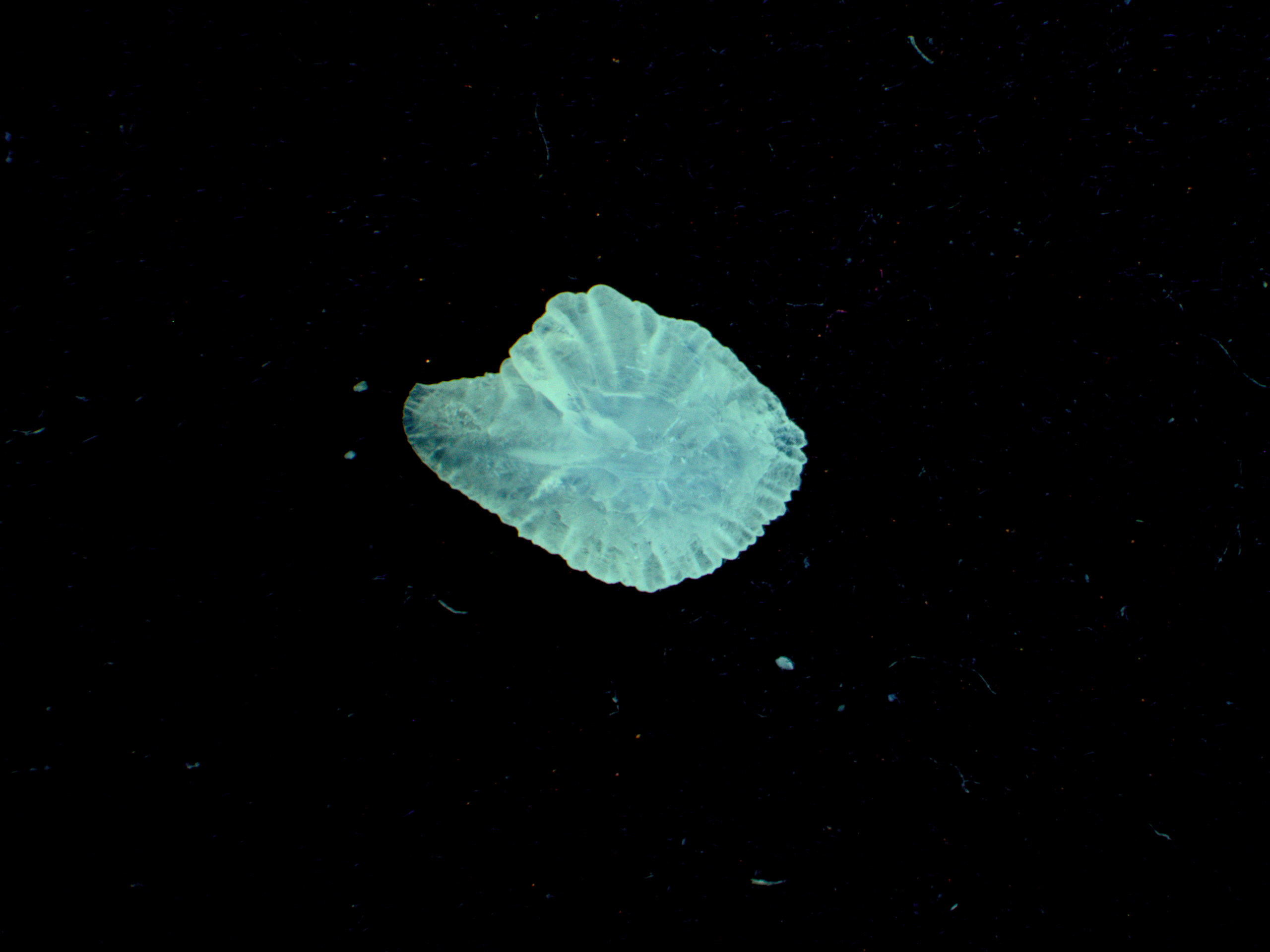

Supplement: Supplemental Information 10 [file peerj-04-1664-s010.zip › Thryssa/training/Eng233R1.jpg]

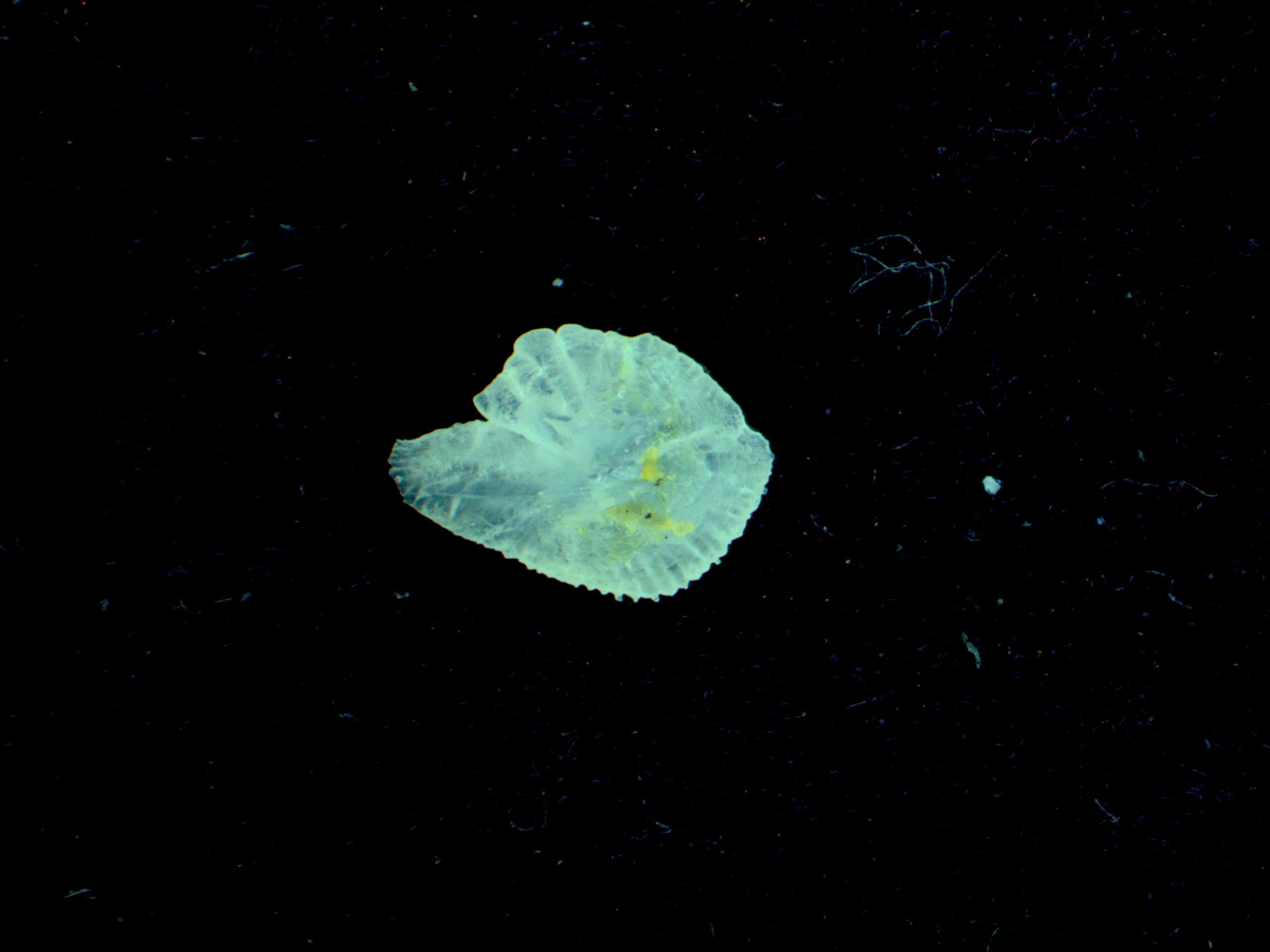

Supplement: Supplemental Information 10 [file peerj-04-1664-s010.zip › Thryssa/training/Eng234R1.jpg]

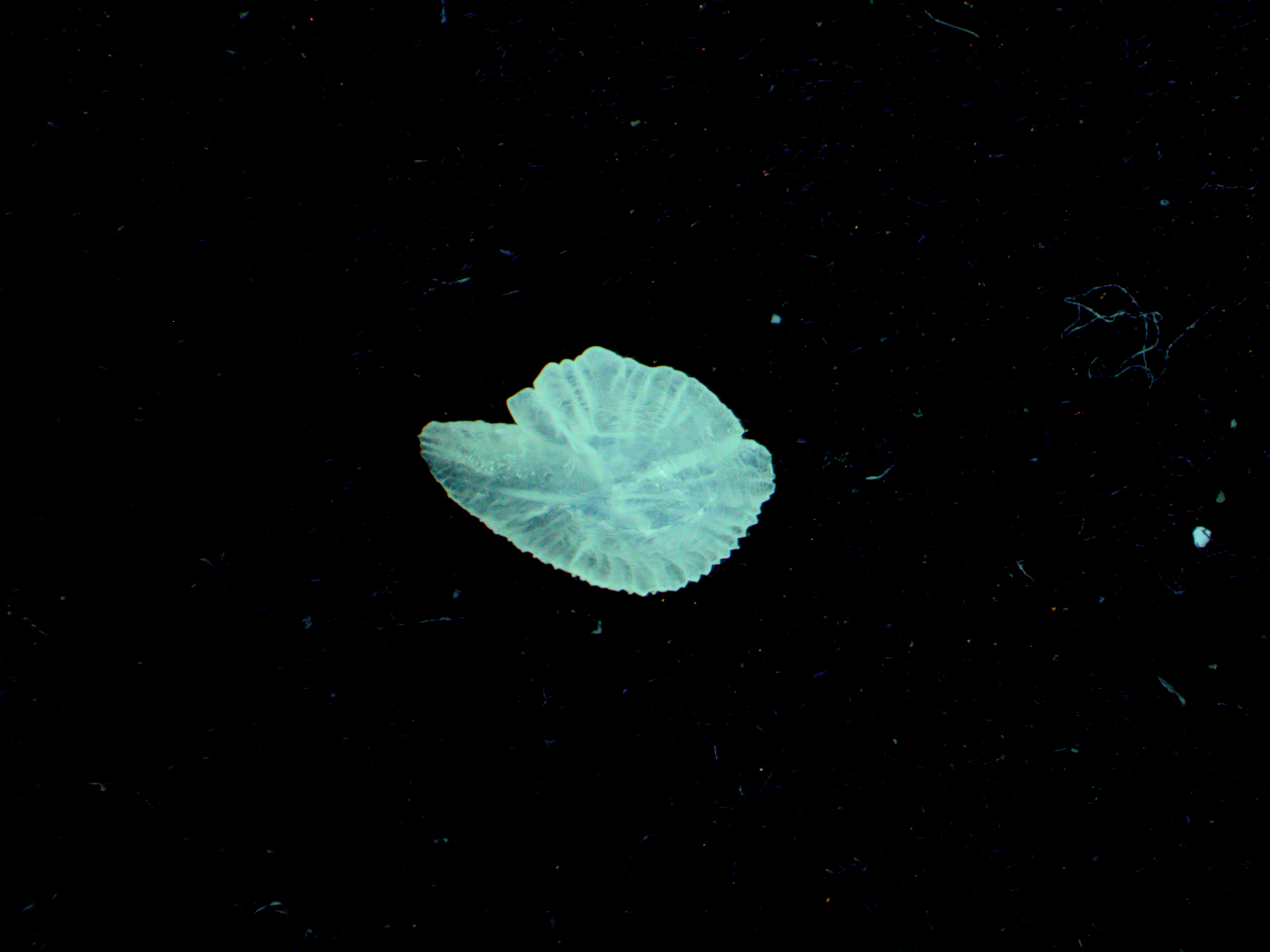

Supplement: Supplemental Information 10 [file peerj-04-1664-s010.zip › Thryssa/training/Eng235R1.jpg]

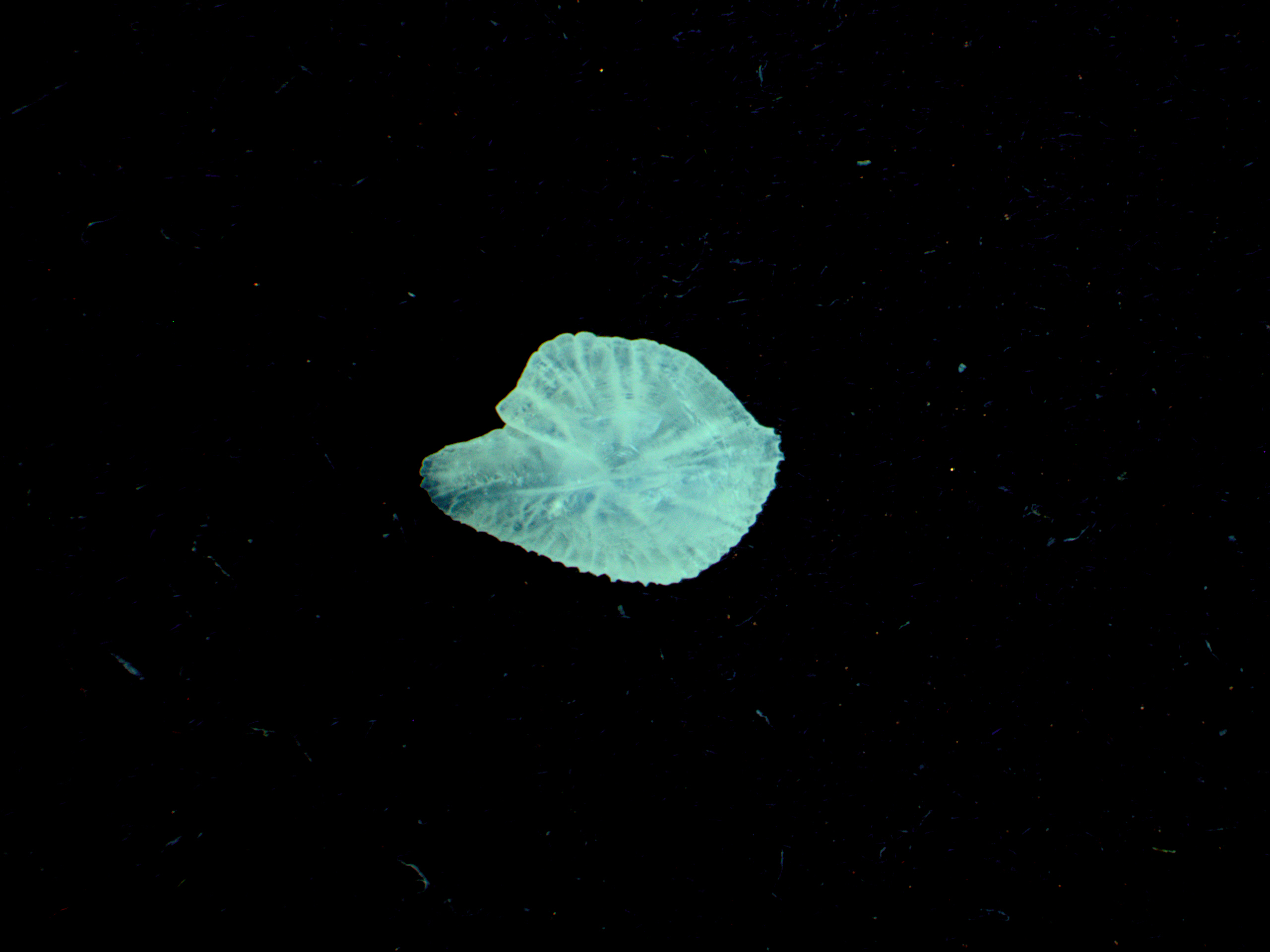

Supplement: Supplemental Information 10 [file peerj-04-1664-s010.zip › Thryssa/training/Eng236R1.jpg]

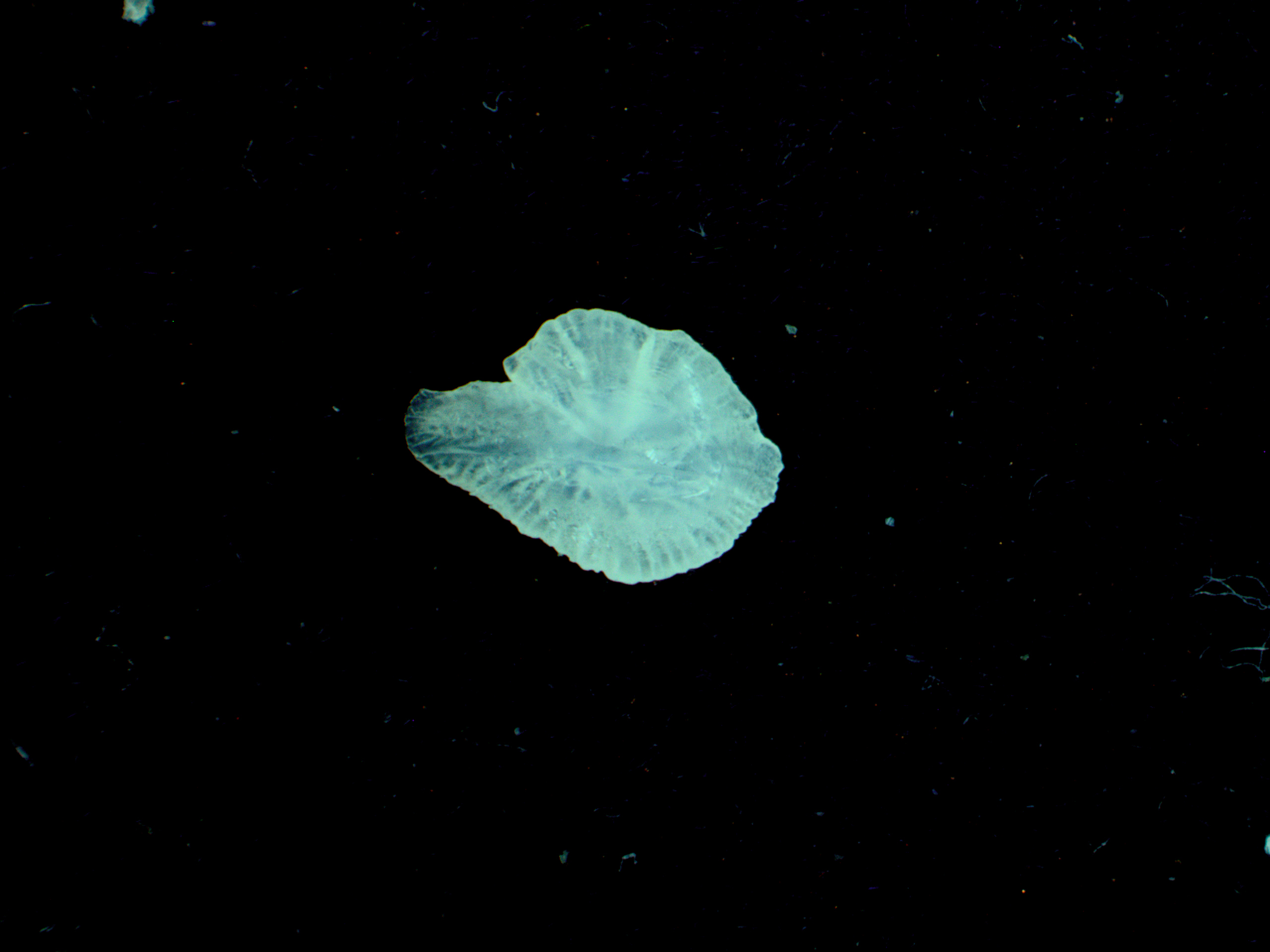

Supplement: Supplemental Information 10 [file peerj-04-1664-s010.zip › Thryssa/training/Eng237R1.jpg]

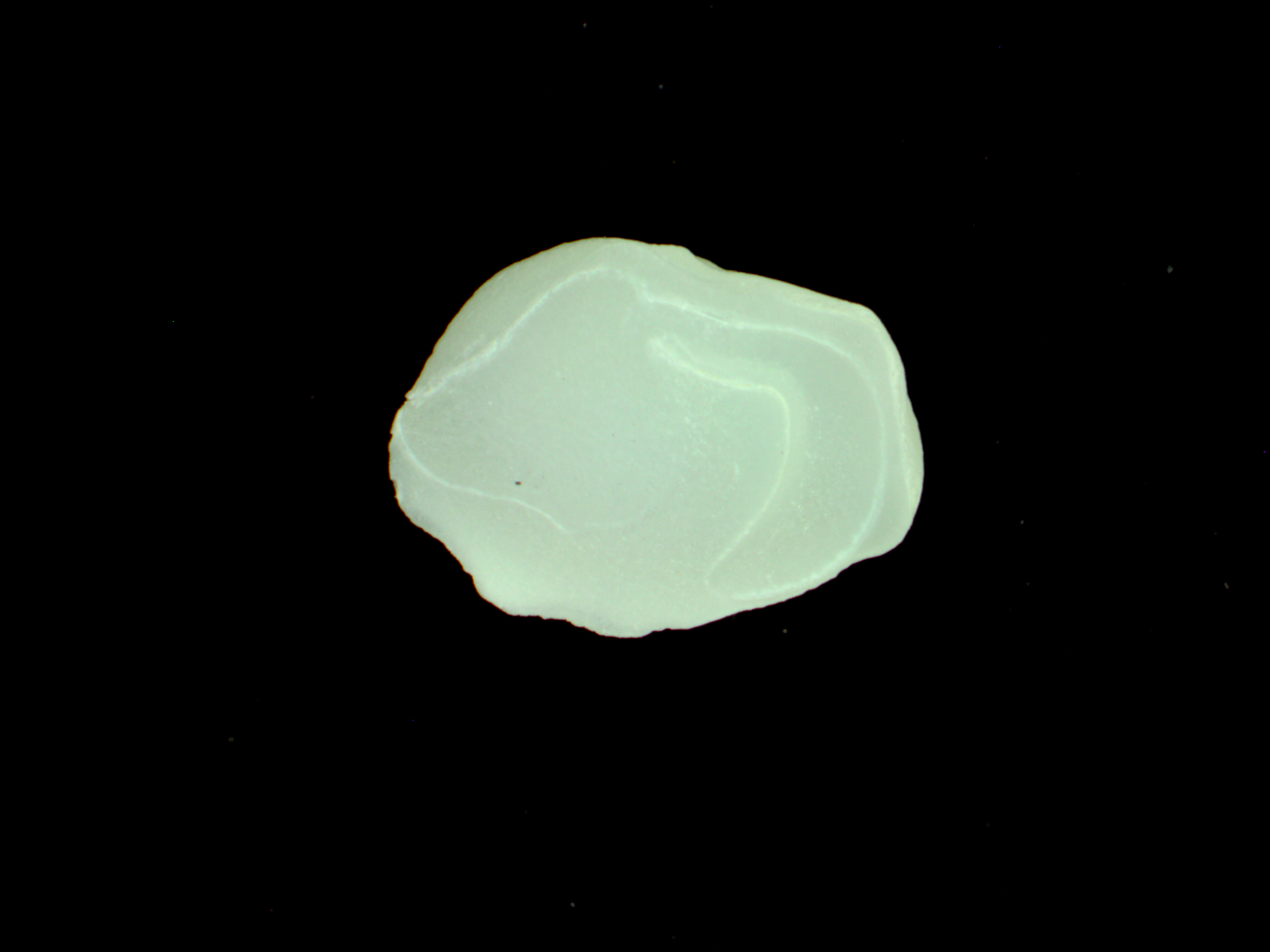

Supplement: Supplemental Information 11 [file peerj-04-1664-s011.zip › DenRus/testing/F72R1.jpg]

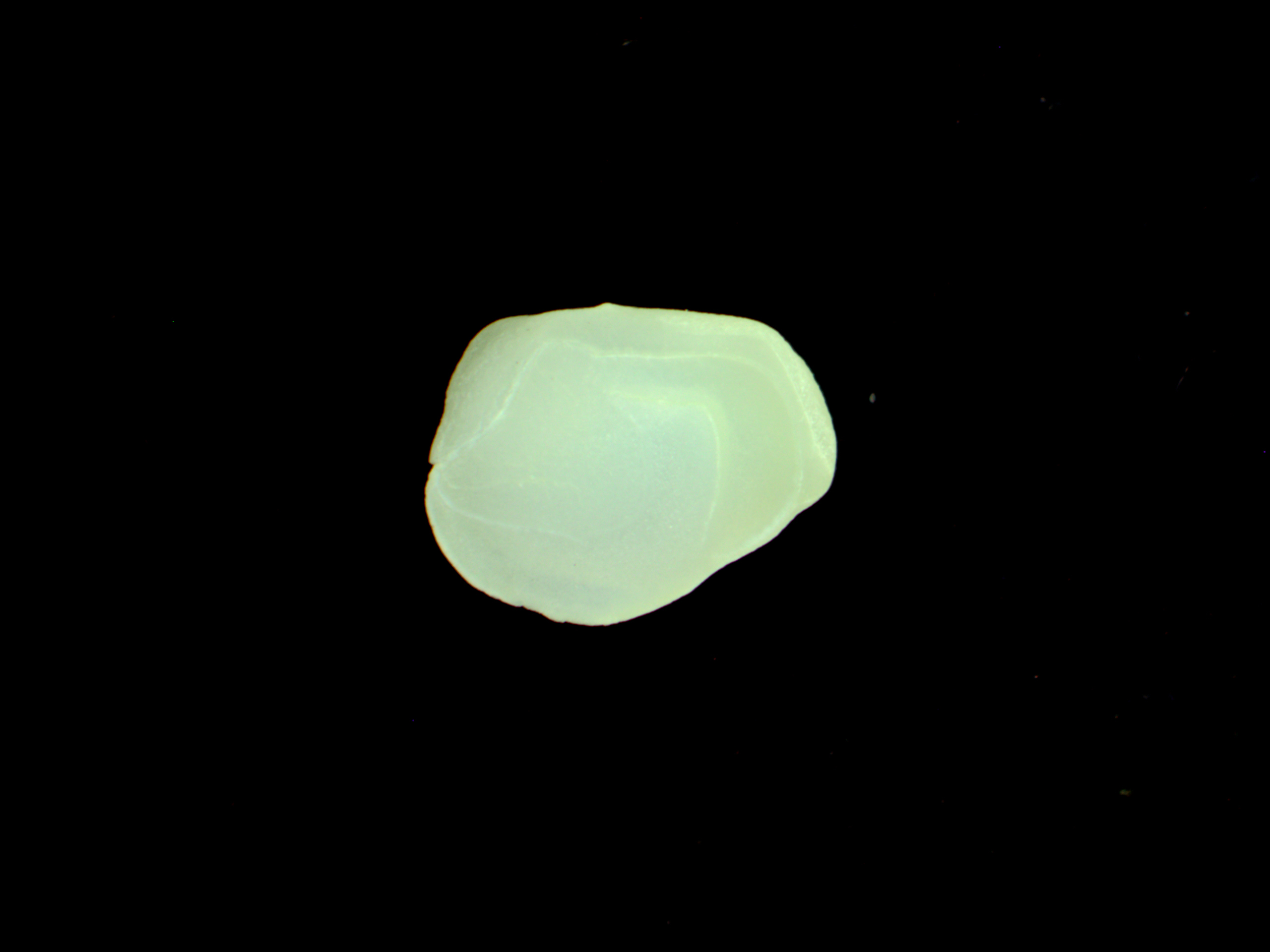

Supplement: Supplemental Information 11 [file peerj-04-1664-s011.zip › DenRus/testing/Q12R1.jpg]

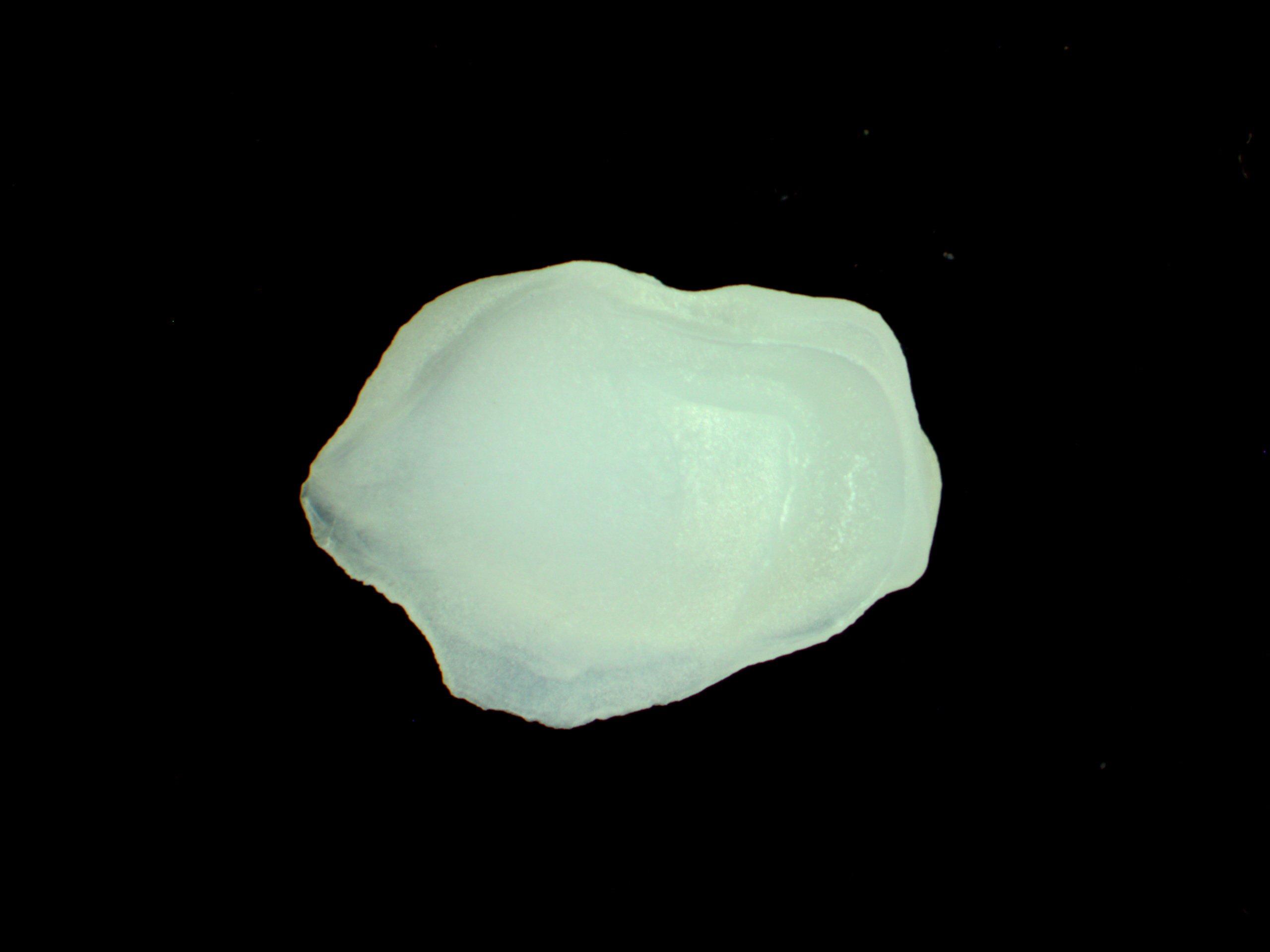

Supplement: Supplemental Information 11 [file peerj-04-1664-s011.zip › DenRus/testing/S26R1.jpg]

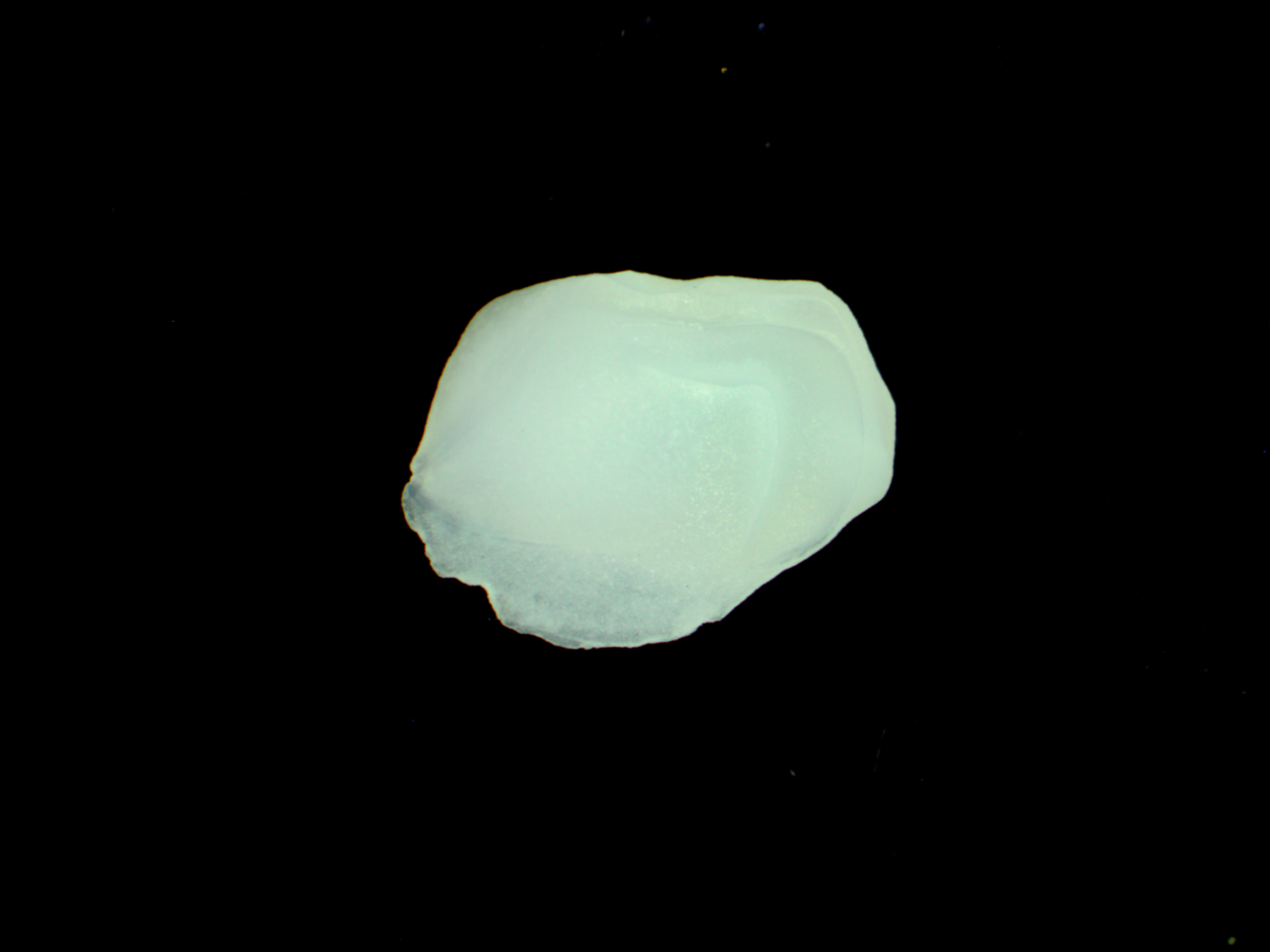

Supplement: Supplemental Information 11 [file peerj-04-1664-s011.zip › DenRus/testing/S27R1.jpg]

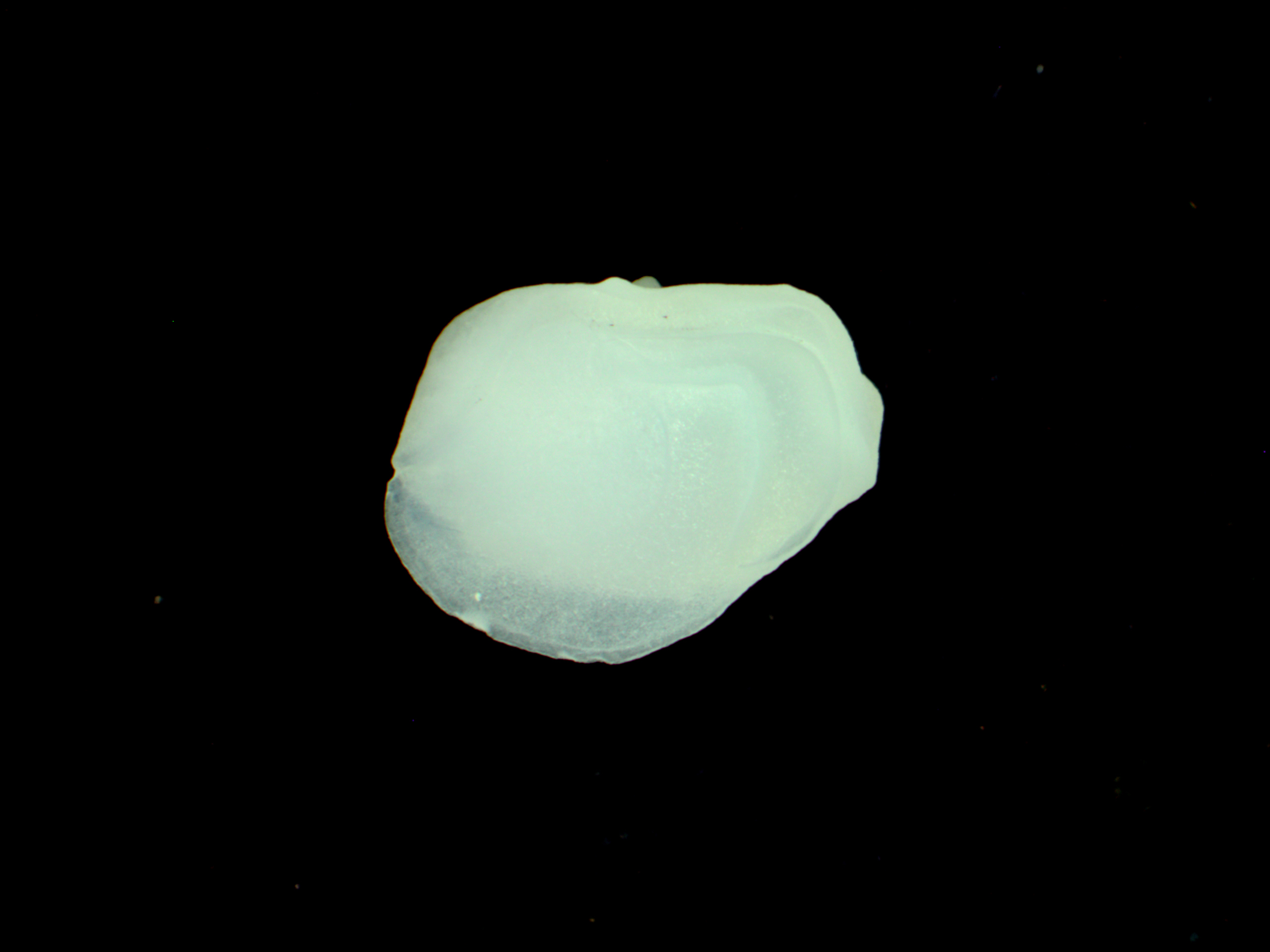

Supplement: Supplemental Information 11 [file peerj-04-1664-s011.zip › DenRus/testing/S28R1.jpg]

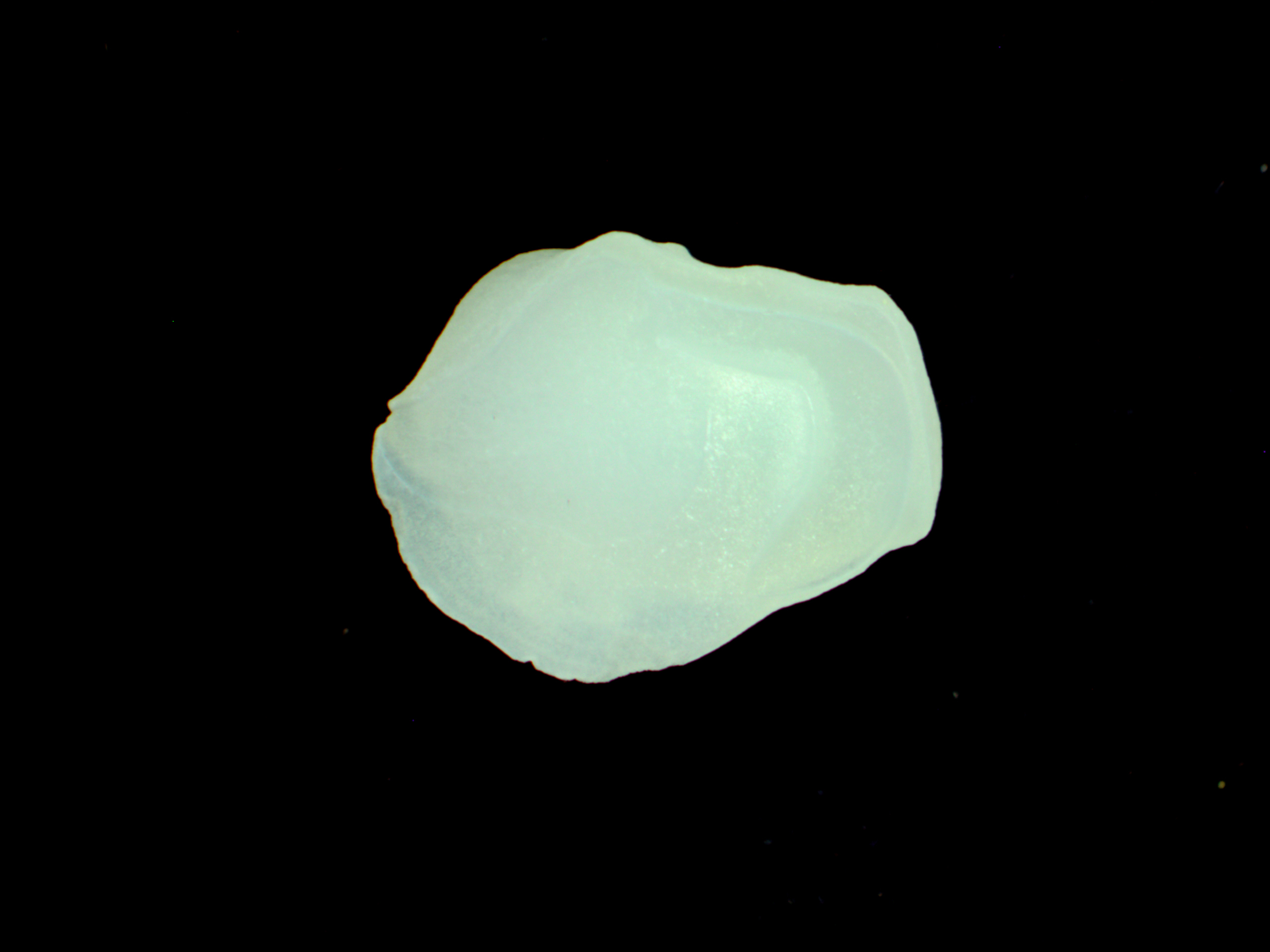

Supplement: Supplemental Information 11 [file peerj-04-1664-s011.zip › DenRus/testing/S37R1.jpg]

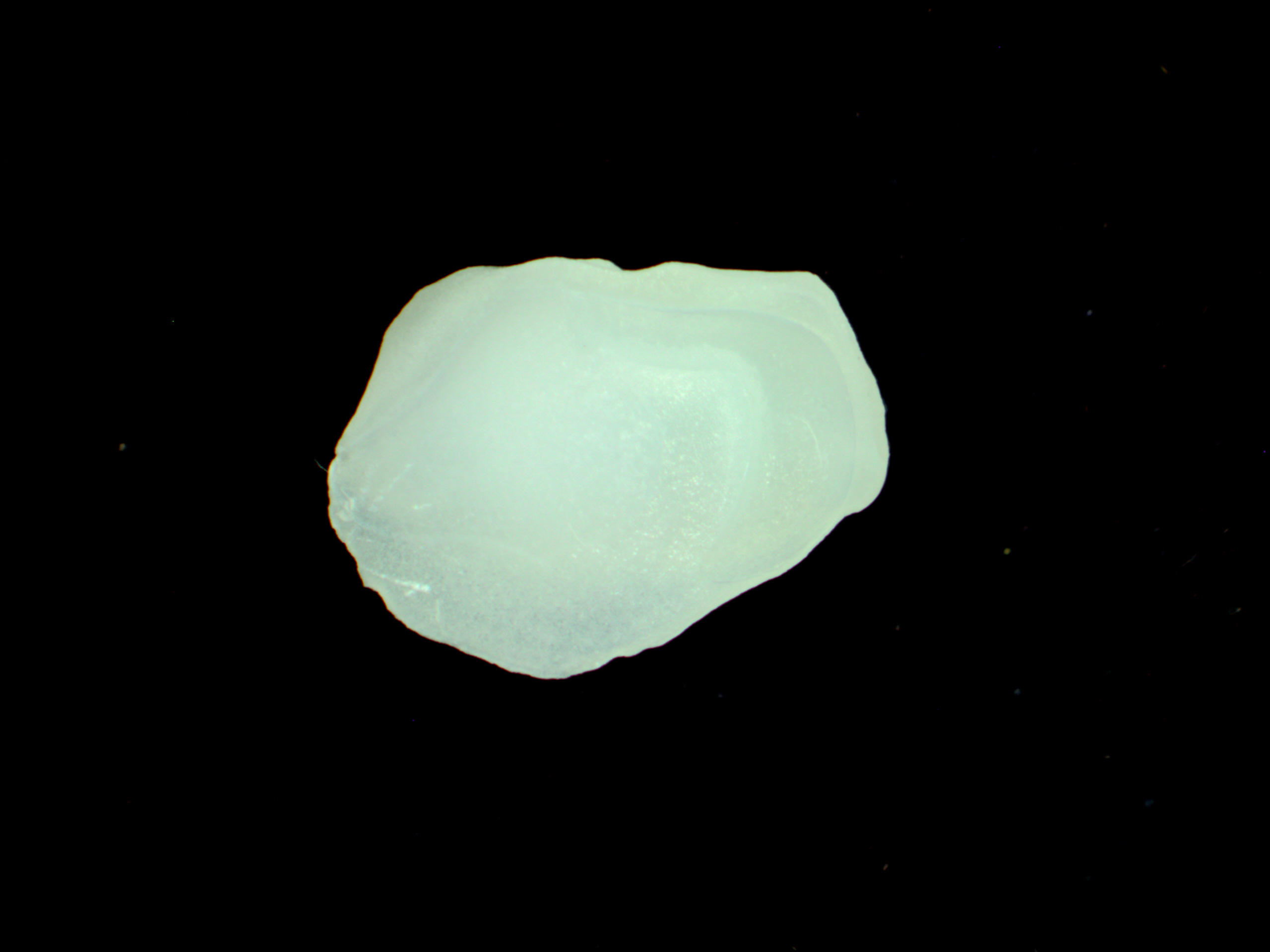

Supplement: Supplemental Information 11 [file peerj-04-1664-s011.zip › DenRus/testing/S38R1.jpg]

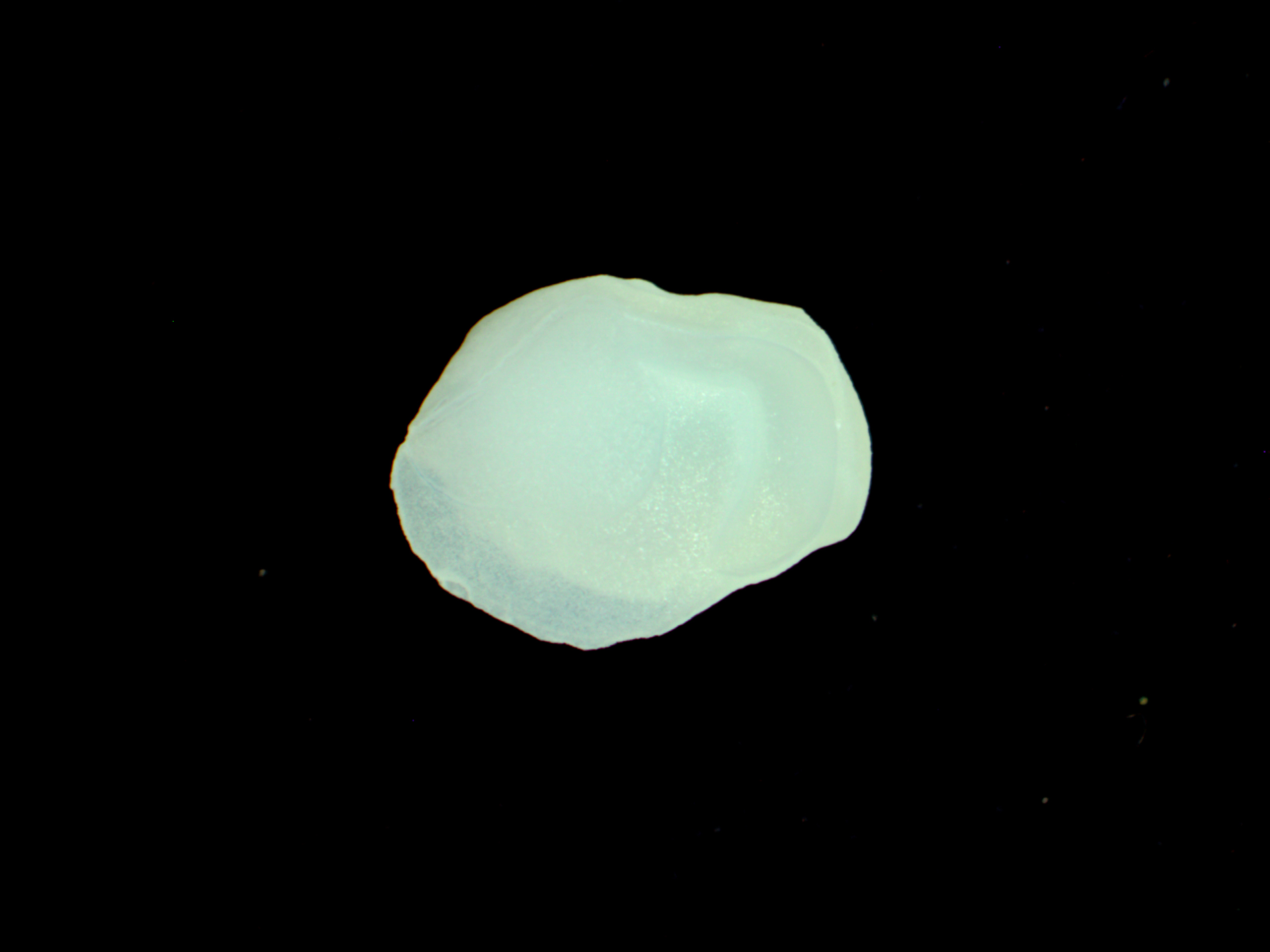

Supplement: Supplemental Information 11 [file peerj-04-1664-s011.zip › DenRus/testing/S39R1.jpg]

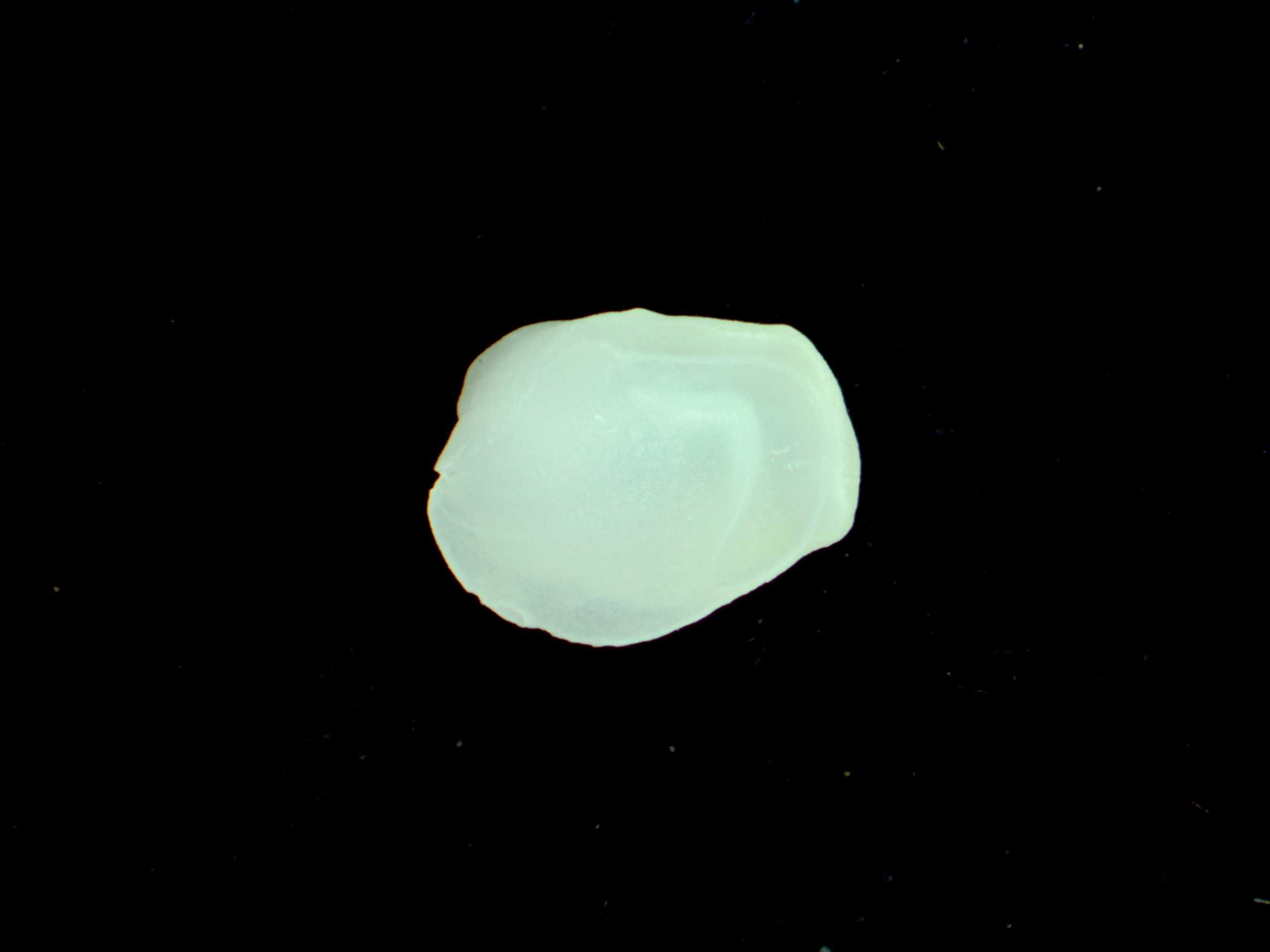

Supplement: Supplemental Information 11 [file peerj-04-1664-s011.zip › DenRus/testing/S40R1.jpg]

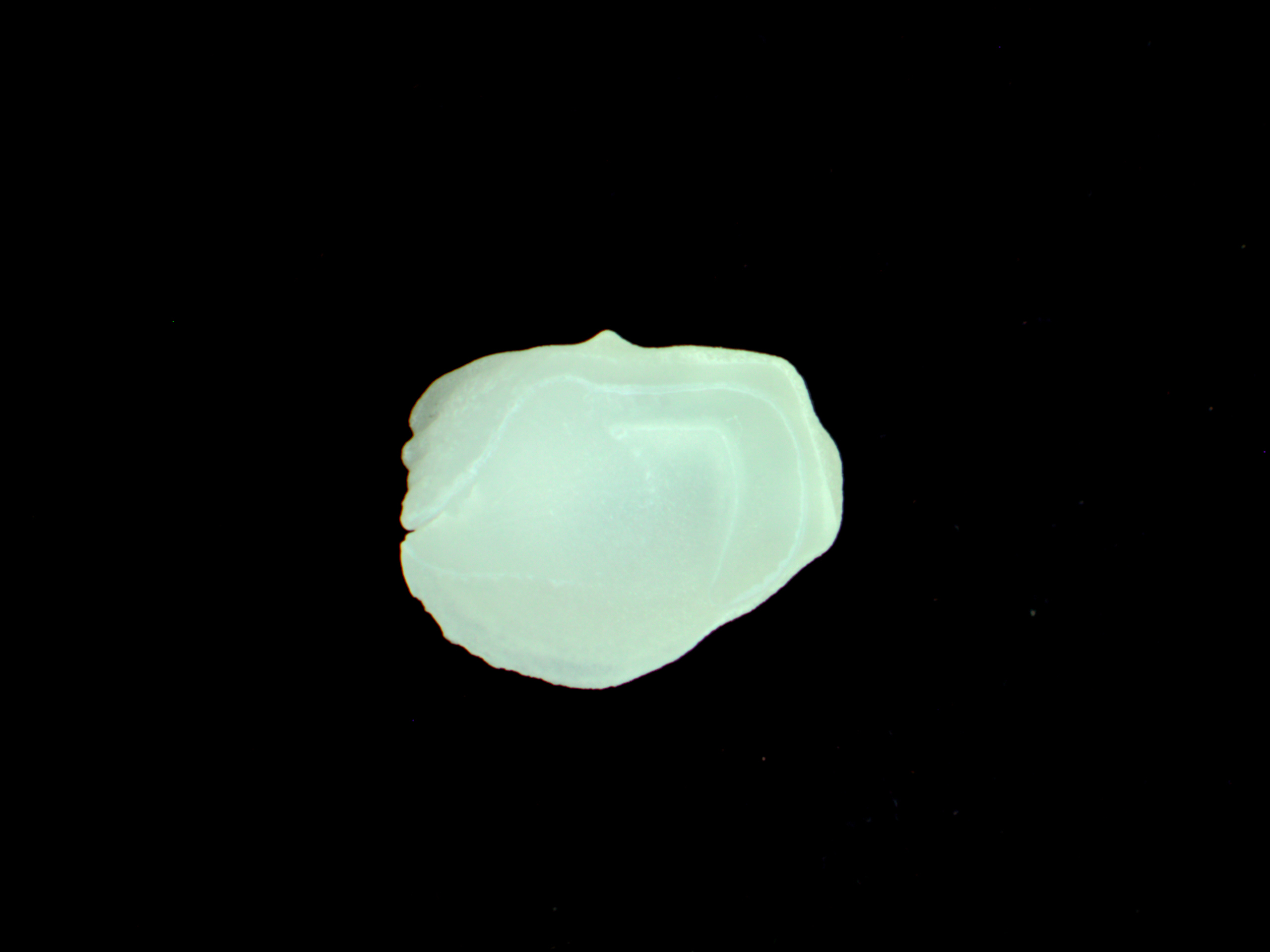

Supplement: Supplemental Information 11 [file peerj-04-1664-s011.zip › DenRus/testing/S55R1.jpg]

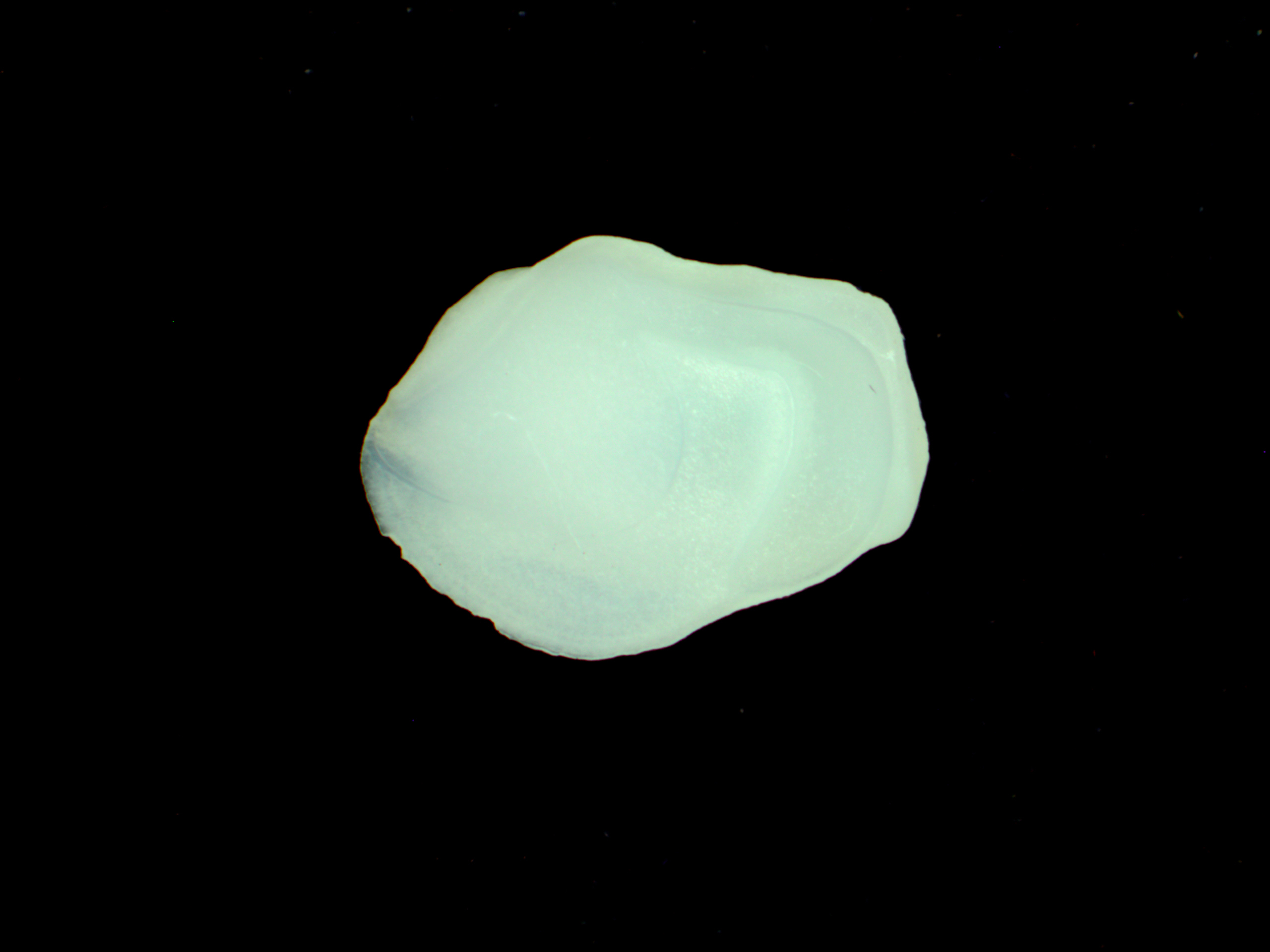

Supplement: Supplemental Information 11 [file peerj-04-1664-s011.zip › DenRus/training/13R1.jpg]

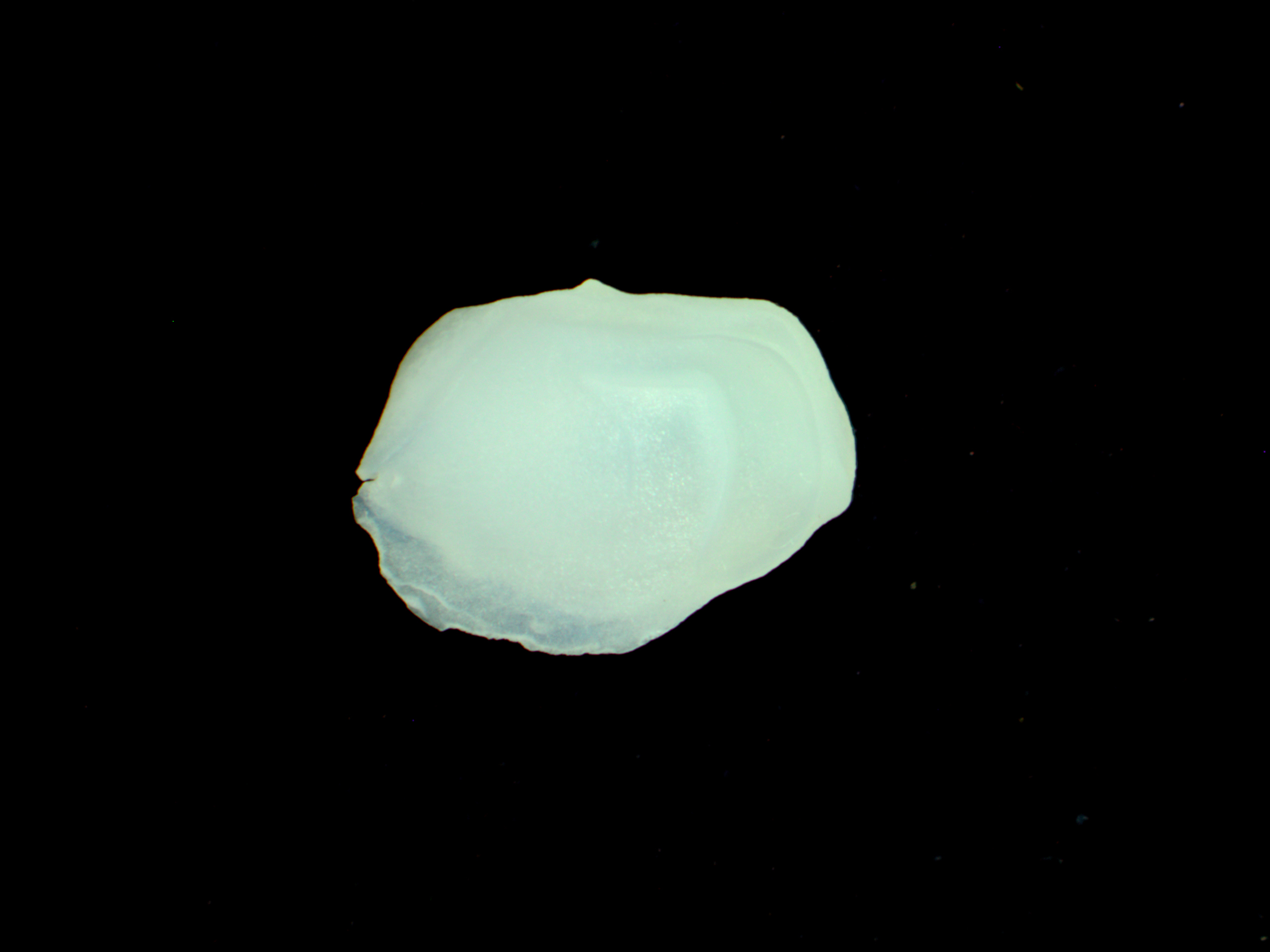

Supplement: Supplemental Information 11 [file peerj-04-1664-s011.zip › DenRus/training/14R1.jpg]

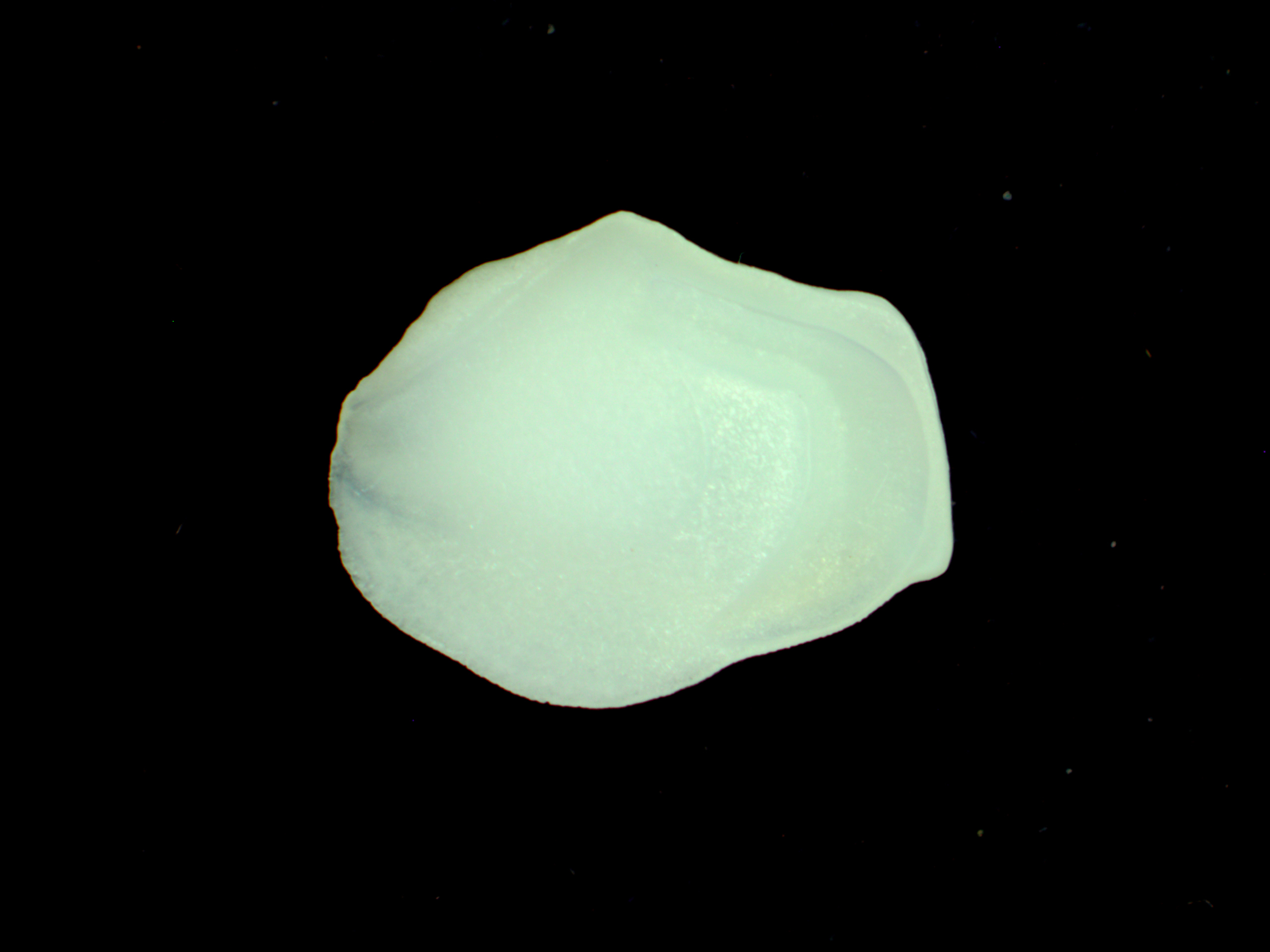

Supplement: Supplemental Information 11 [file peerj-04-1664-s011.zip › DenRus/training/15R1.jpg]

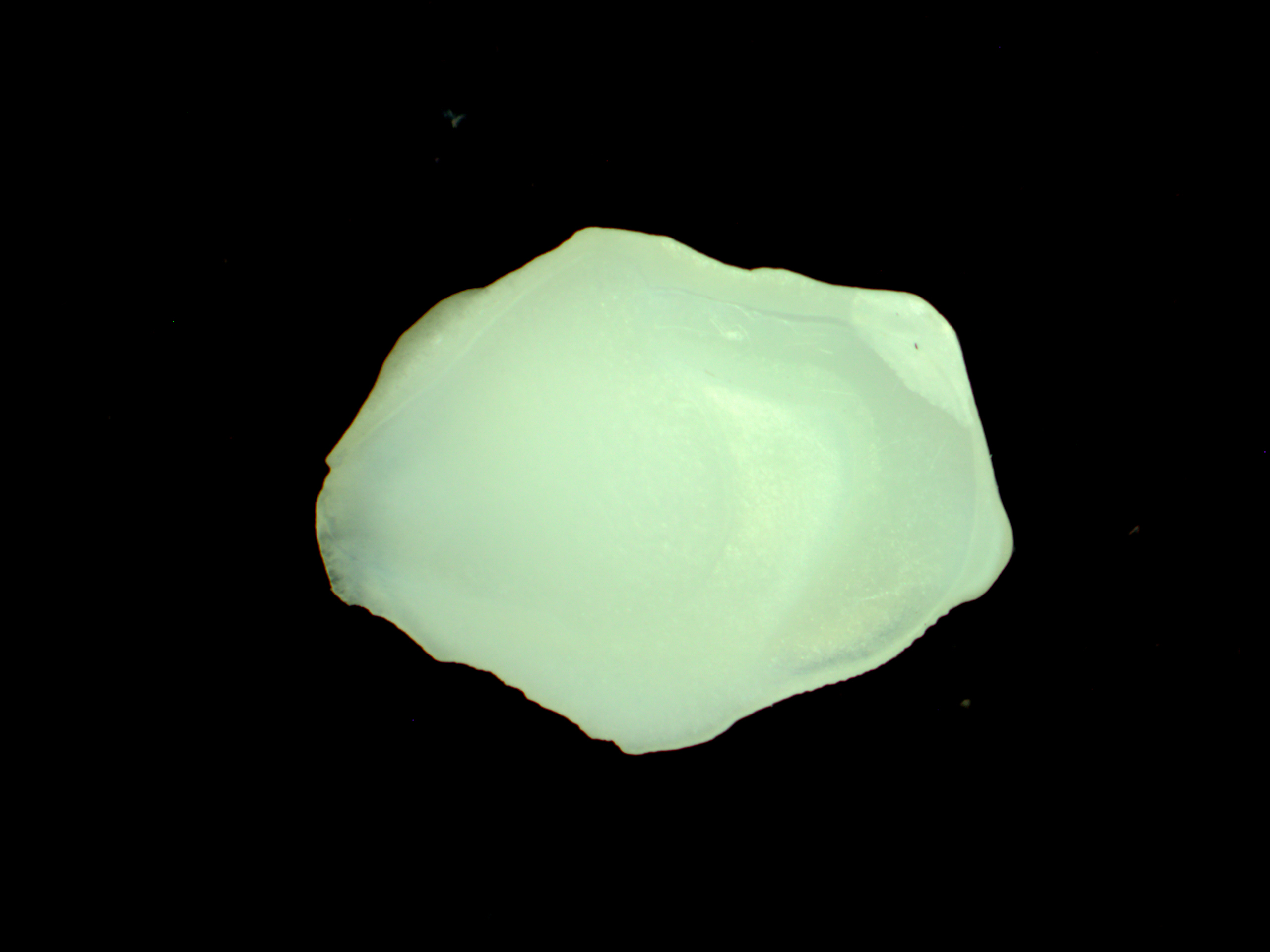

Supplement: Supplemental Information 11 [file peerj-04-1664-s011.zip › DenRus/training/1R1.jpg]

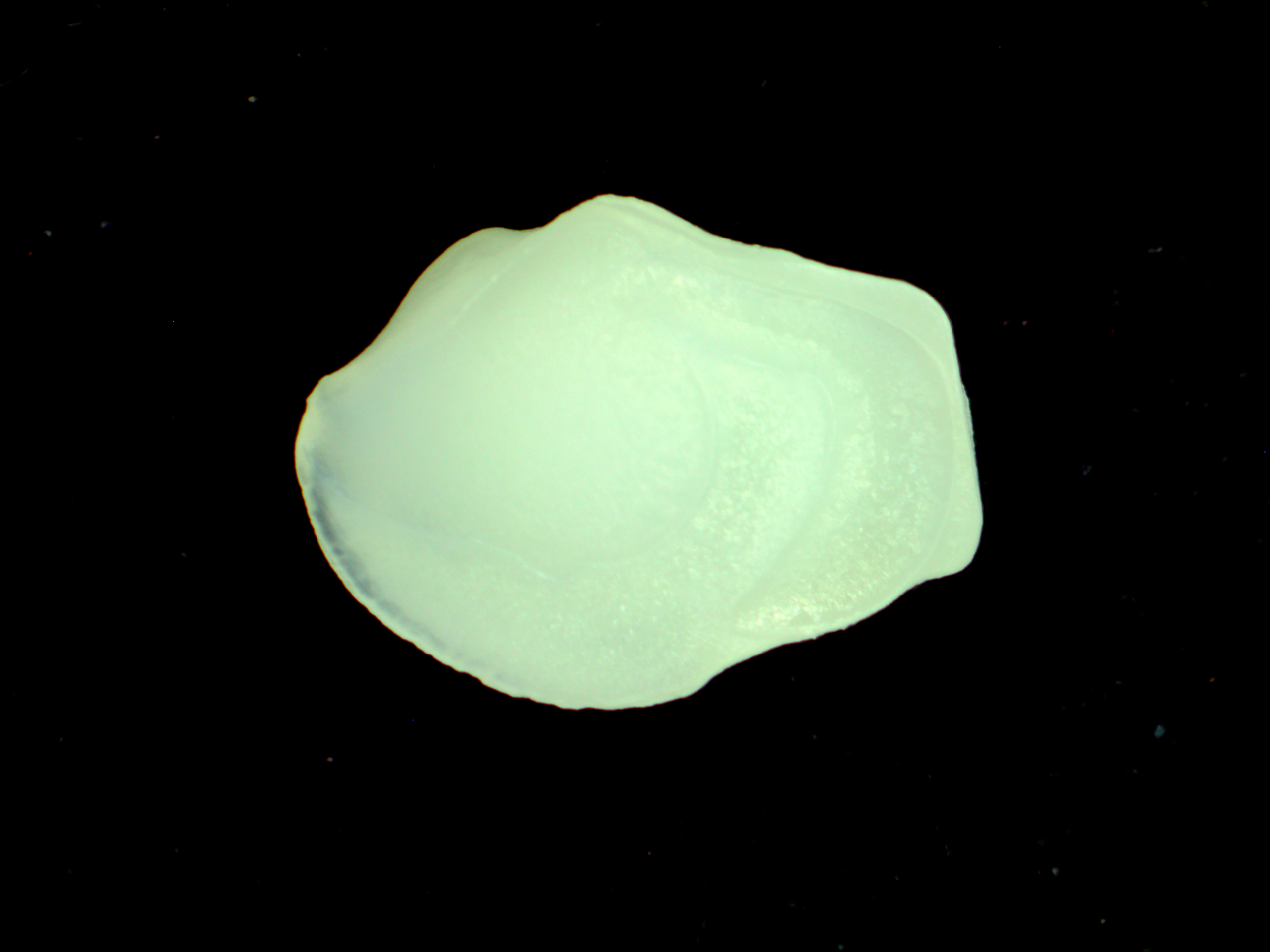

Supplement: Supplemental Information 11 [file peerj-04-1664-s011.zip › DenRus/training/2R1.jpg]

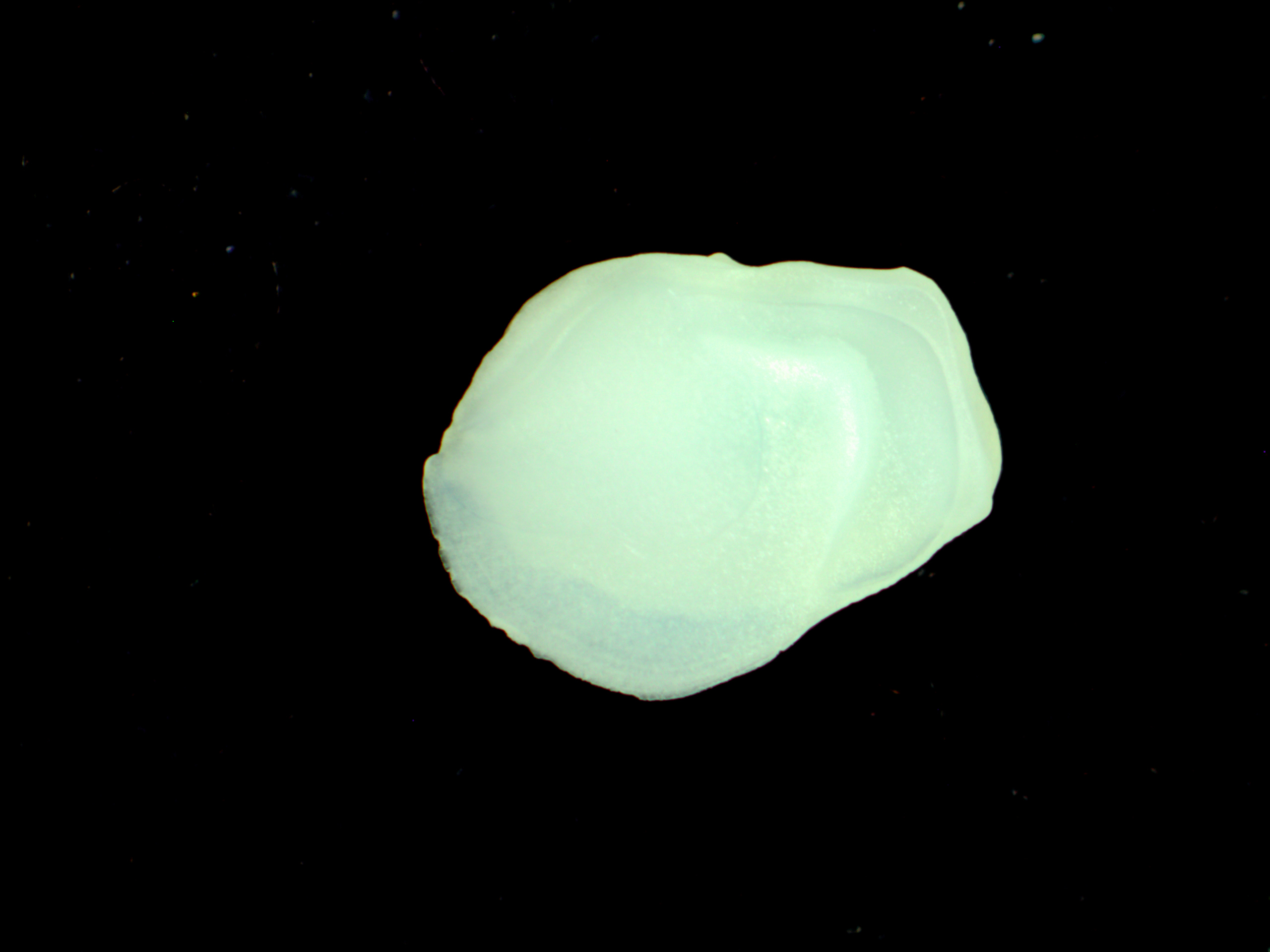

Supplement: Supplemental Information 11 [file peerj-04-1664-s011.zip › DenRus/training/3R1.jpg]

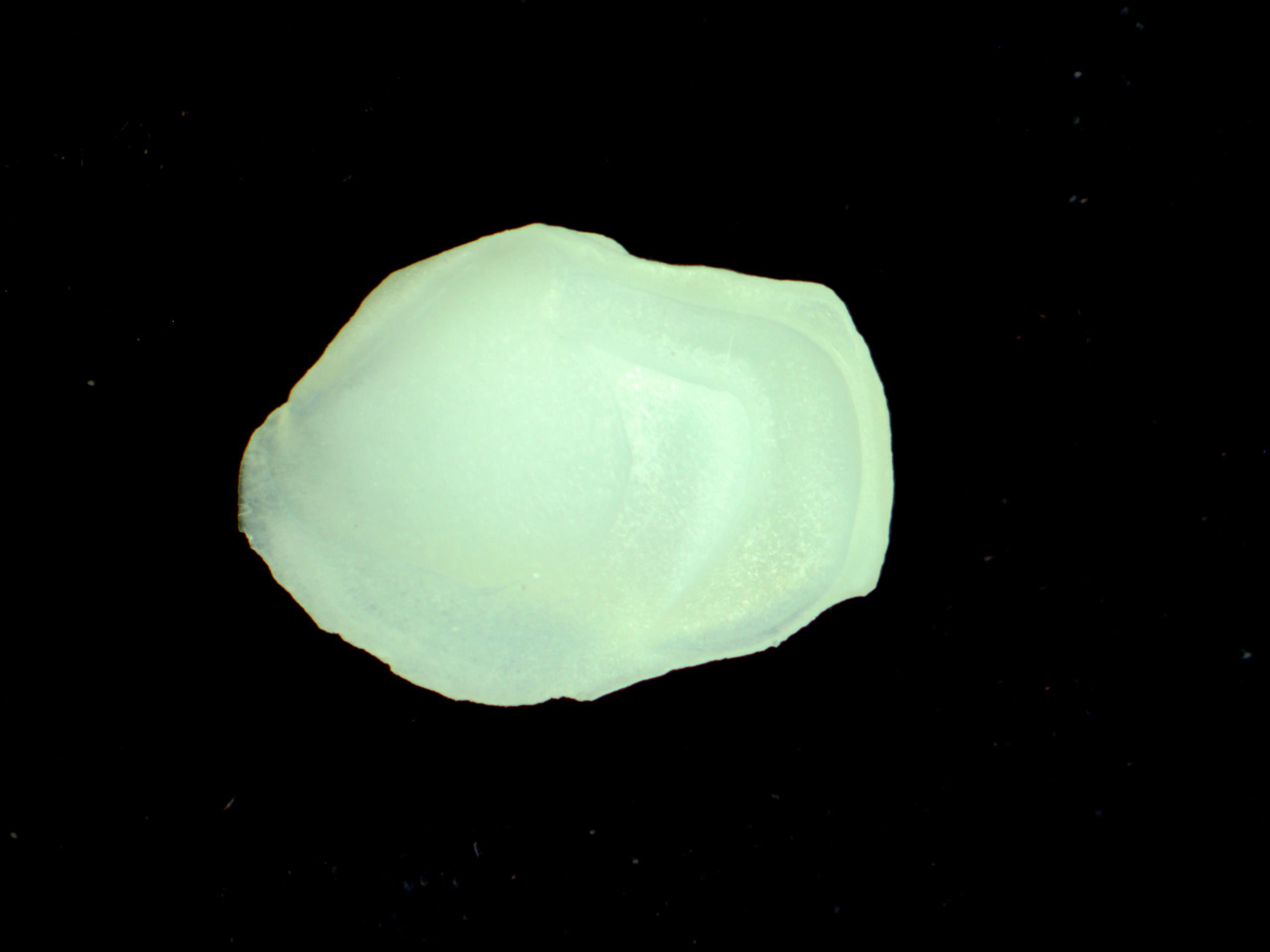

Supplement: Supplemental Information 11 [file peerj-04-1664-s011.zip › DenRus/training/4R1.jpg]

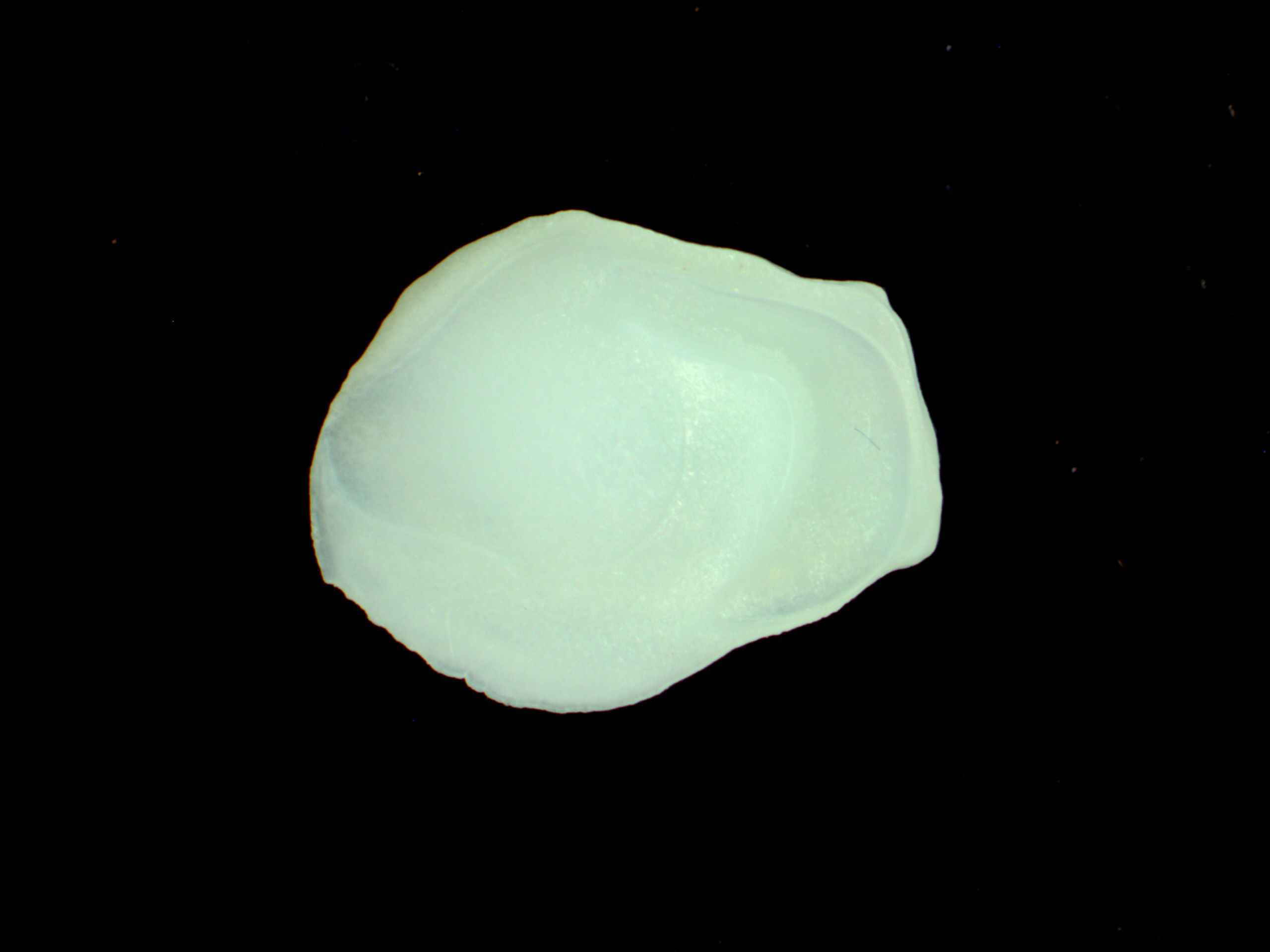

Supplement: Supplemental Information 11 [file peerj-04-1664-s011.zip › DenRus/training/518R1.jpg]

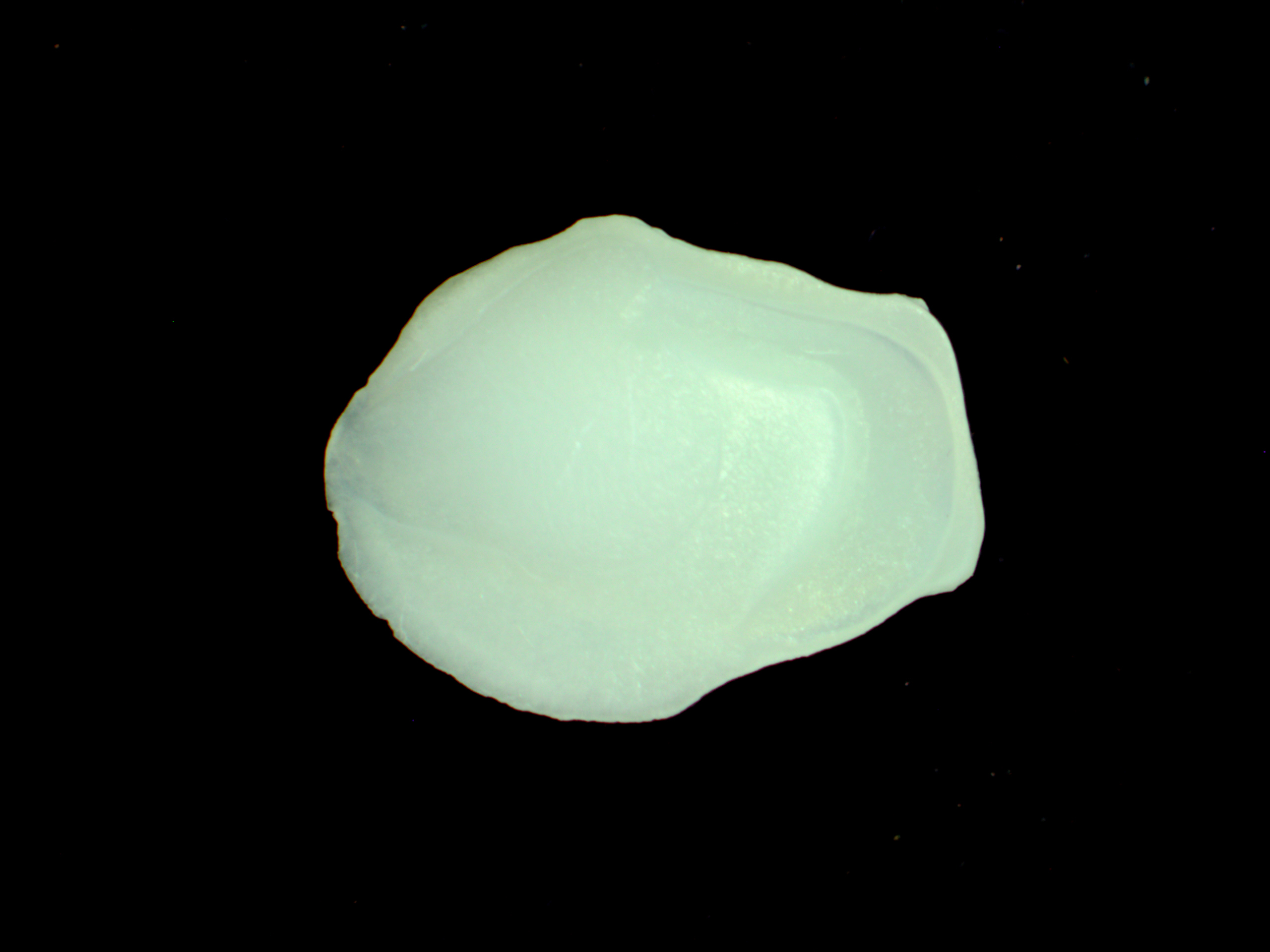

Supplement: Supplemental Information 11 [file peerj-04-1664-s011.zip › DenRus/training/520R1.jpg]

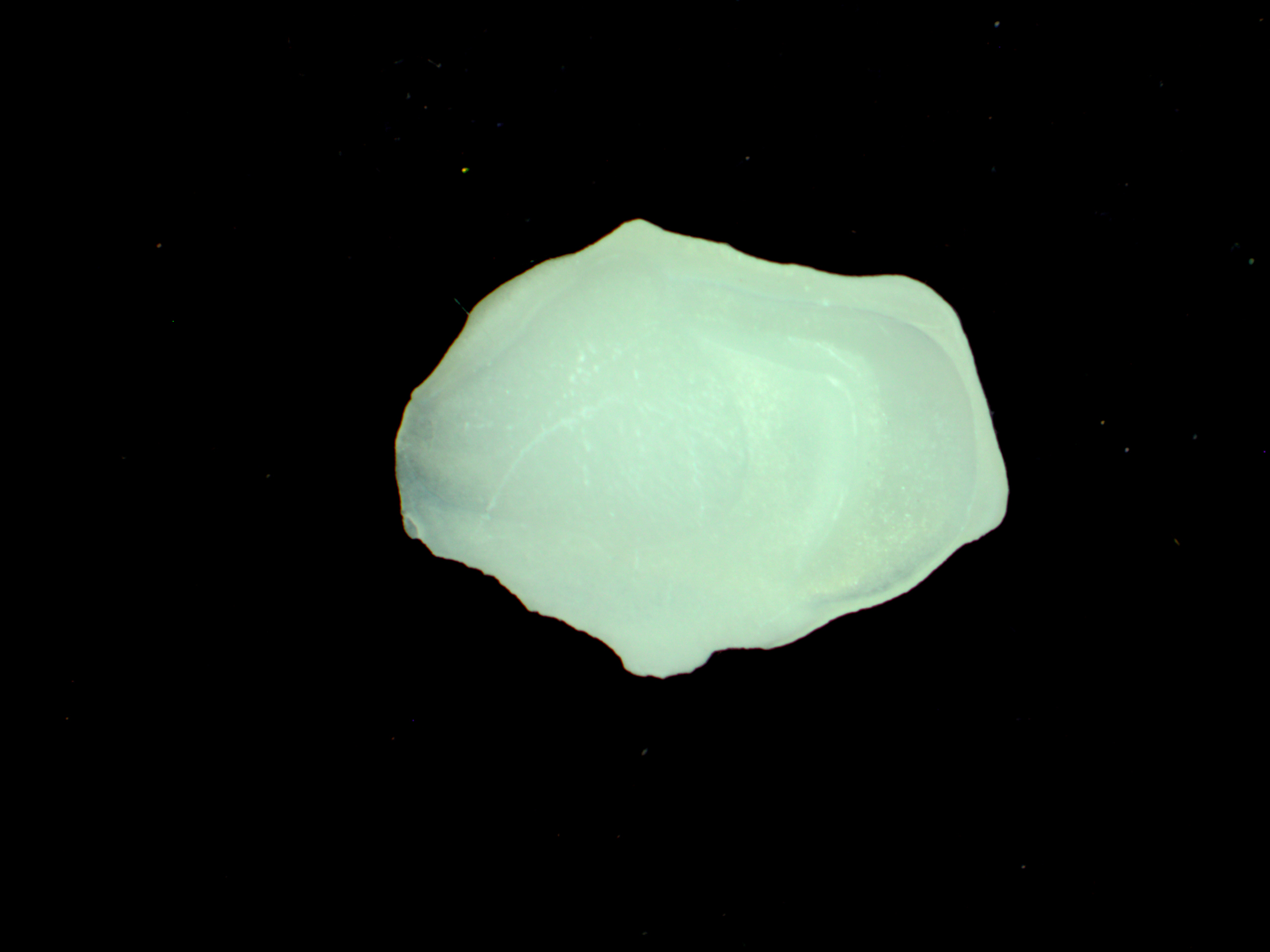

Supplement: Supplemental Information 11 [file peerj-04-1664-s011.zip › DenRus/training/521R1.jpg]

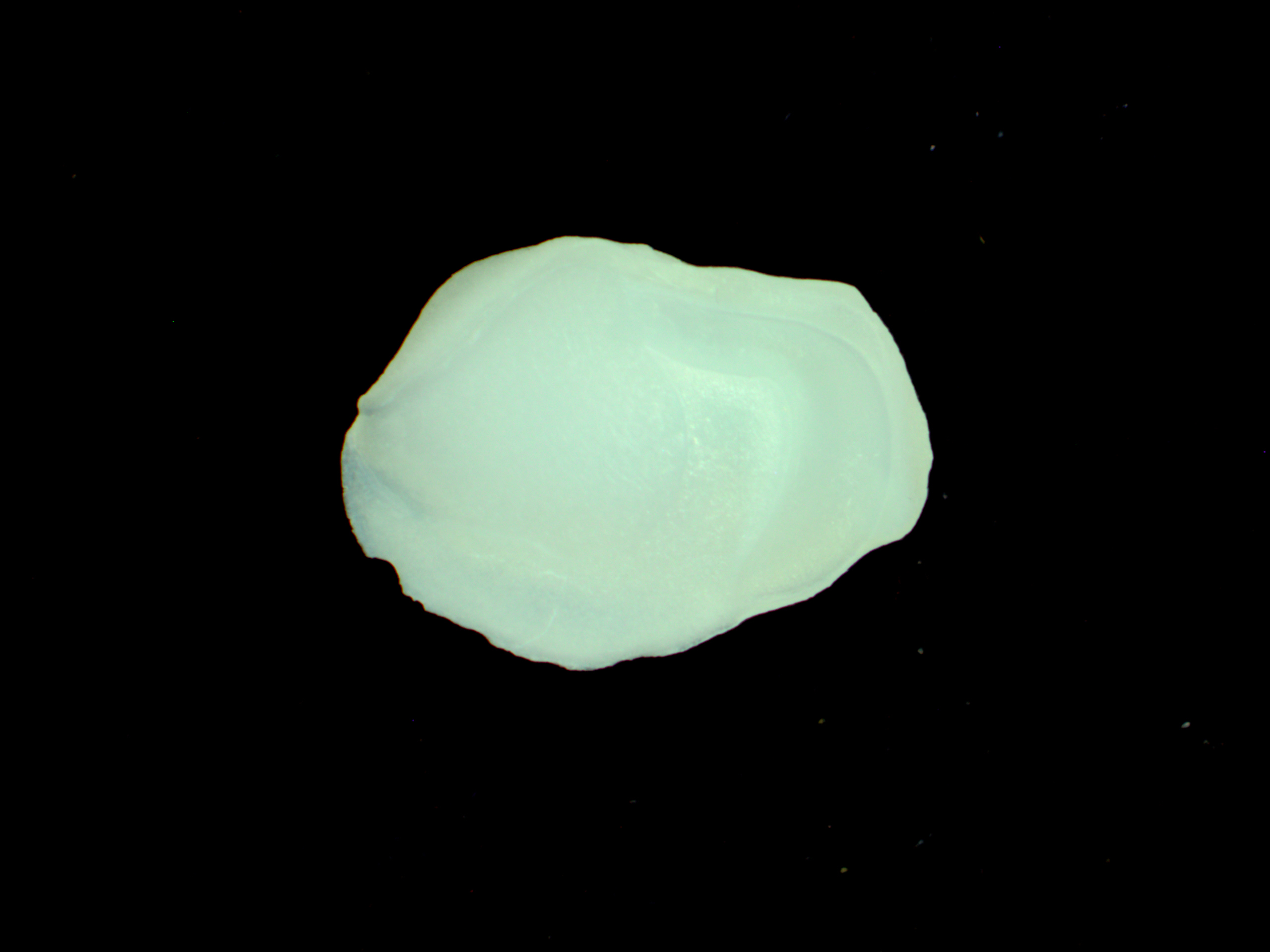

Supplement: Supplemental Information 11 [file peerj-04-1664-s011.zip › DenRus/training/522R1.jpg]

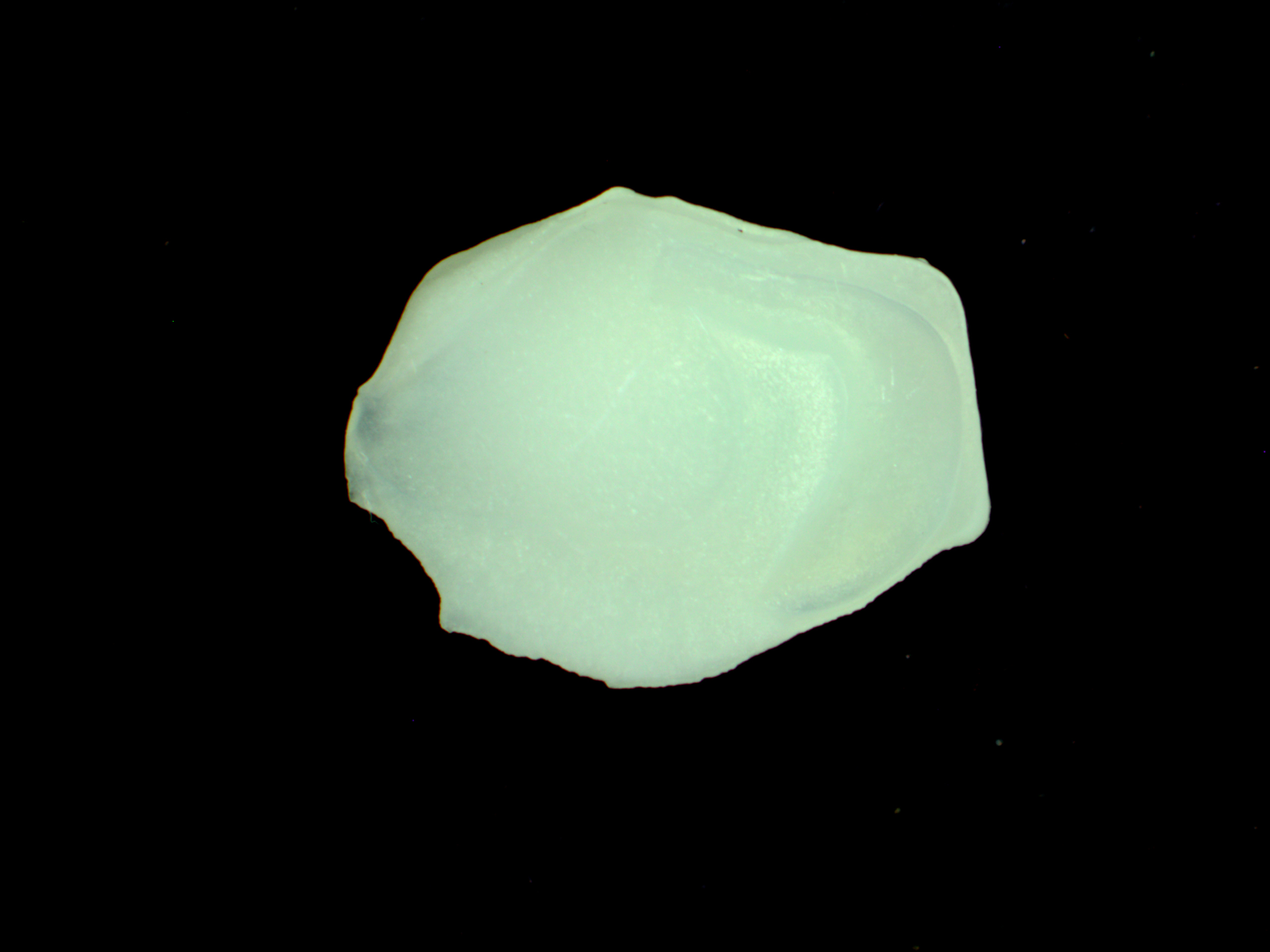

Supplement: Supplemental Information 11 [file peerj-04-1664-s011.zip › DenRus/training/523R1.jpg]

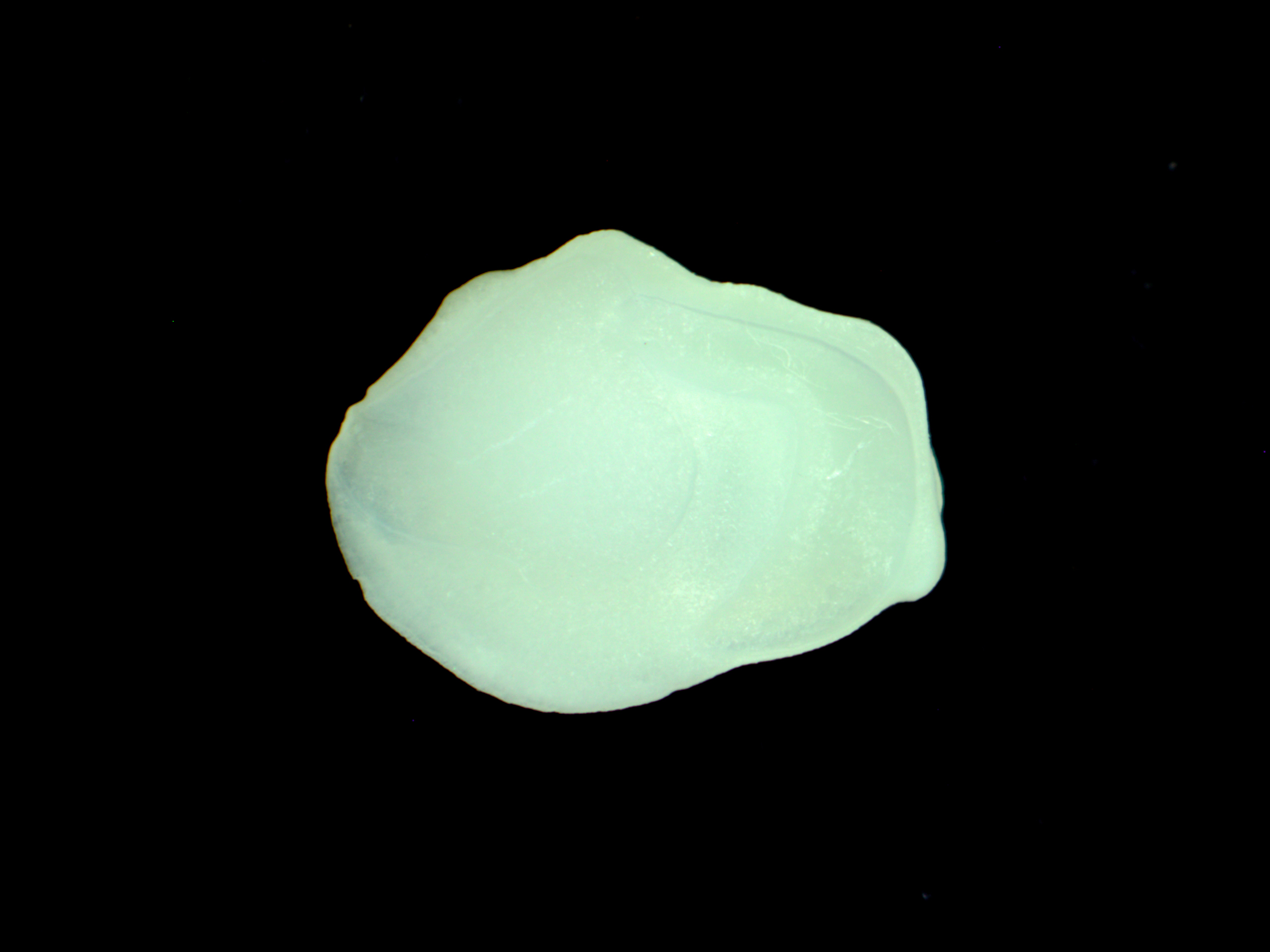

Supplement: Supplemental Information 11 [file peerj-04-1664-s011.zip › DenRus/training/524R1.jpg]

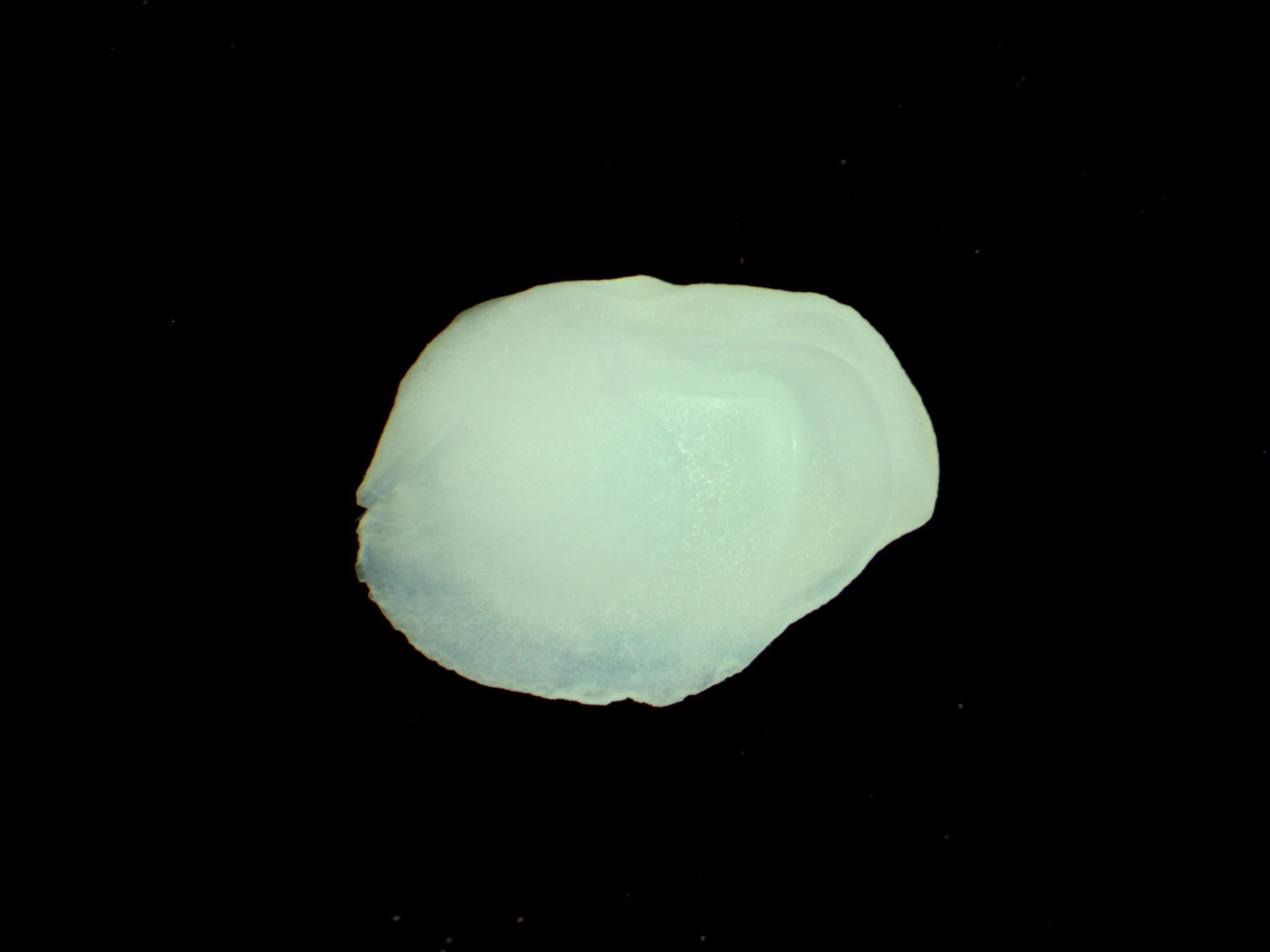

Supplement: Supplemental Information 11 [file peerj-04-1664-s011.zip › DenRus/training/525R1.jpg]

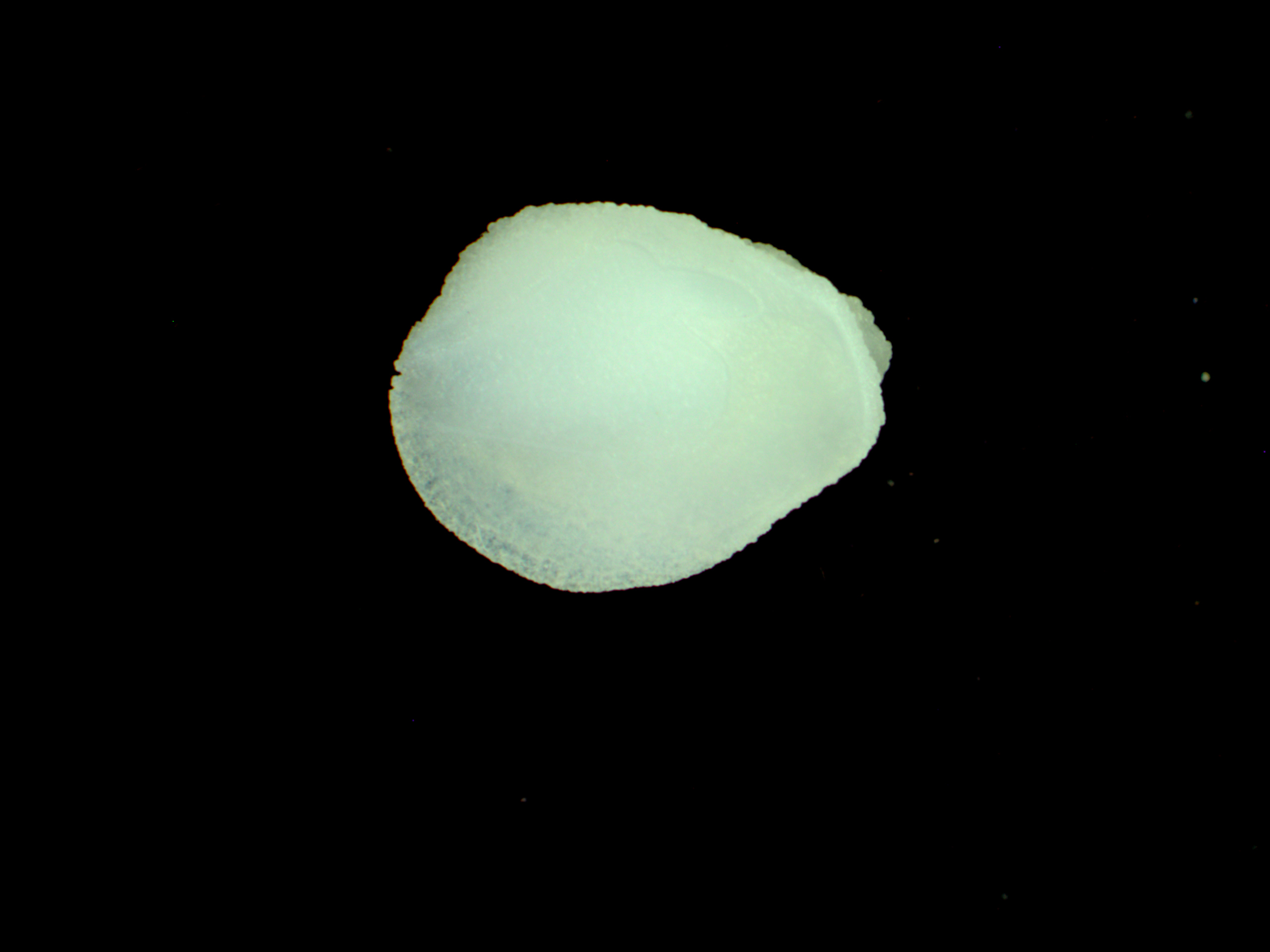

Supplement: Supplemental Information 11 [file peerj-04-1664-s011.zip › DenRus/training/526R1.jpg]

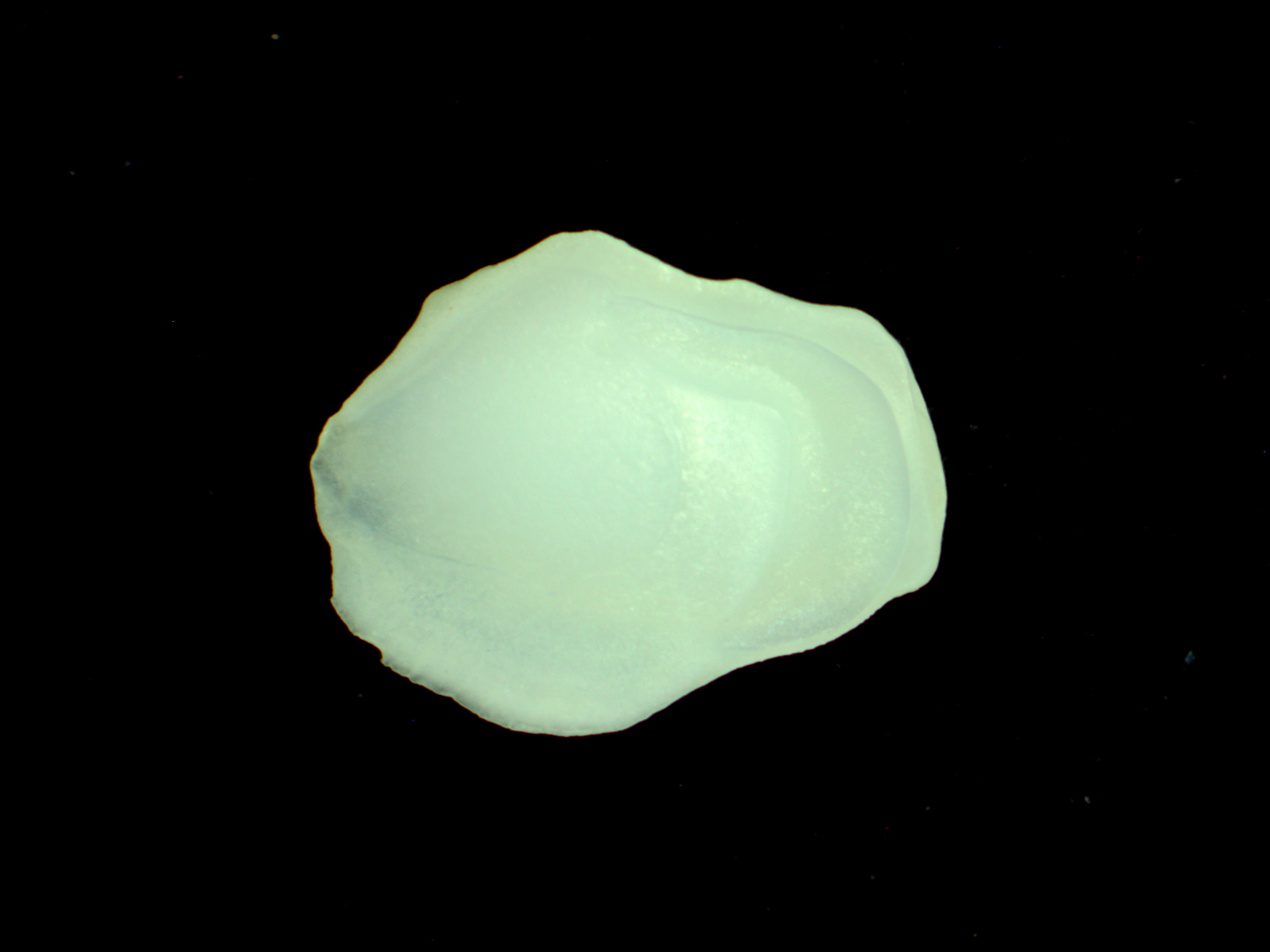

Supplement: Supplemental Information 11 [file peerj-04-1664-s011.zip › DenRus/training/5R1.jpg]

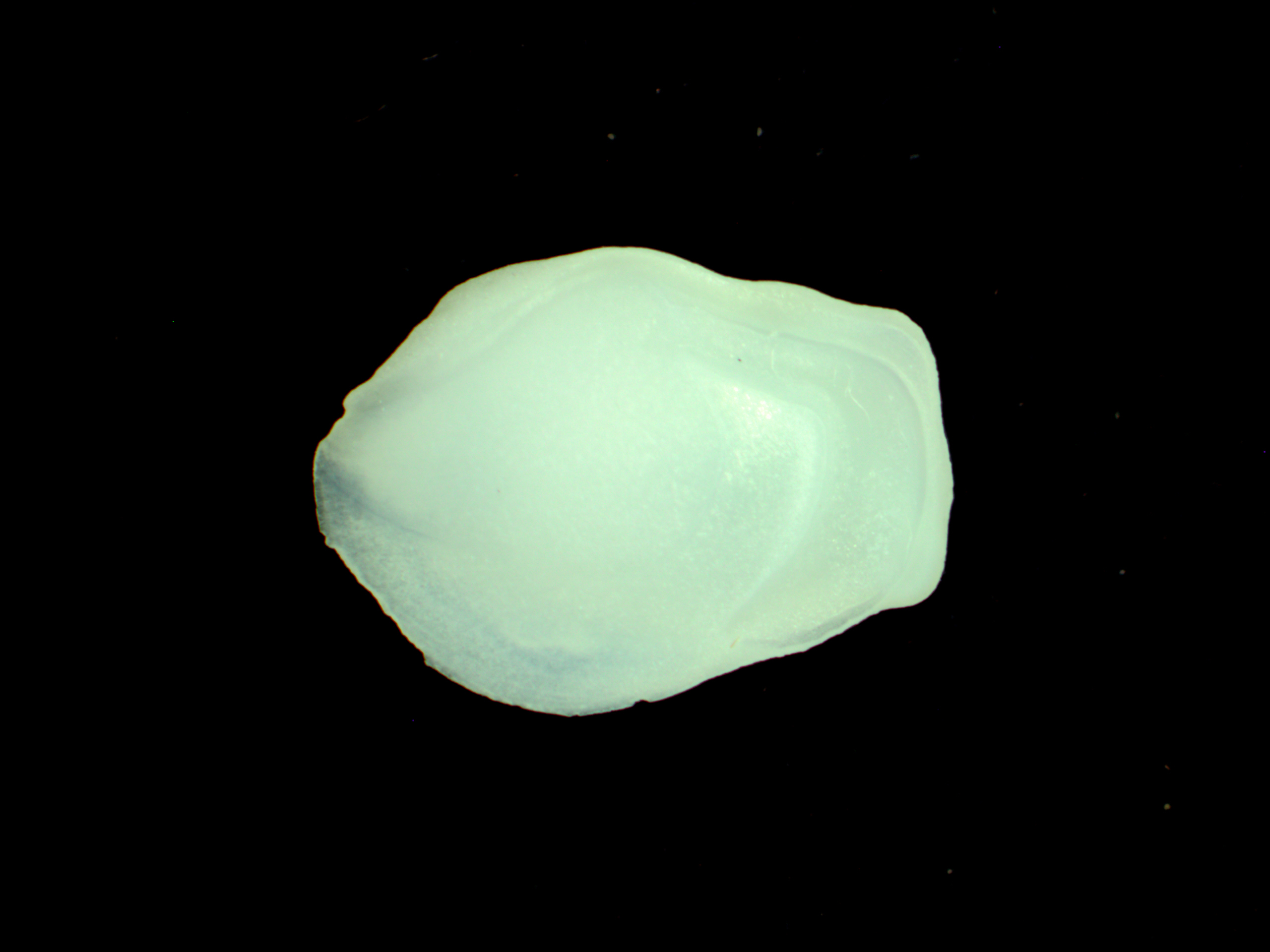

Supplement: Supplemental Information 11 [file peerj-04-1664-s011.zip › DenRus/training/6R1.jpg]

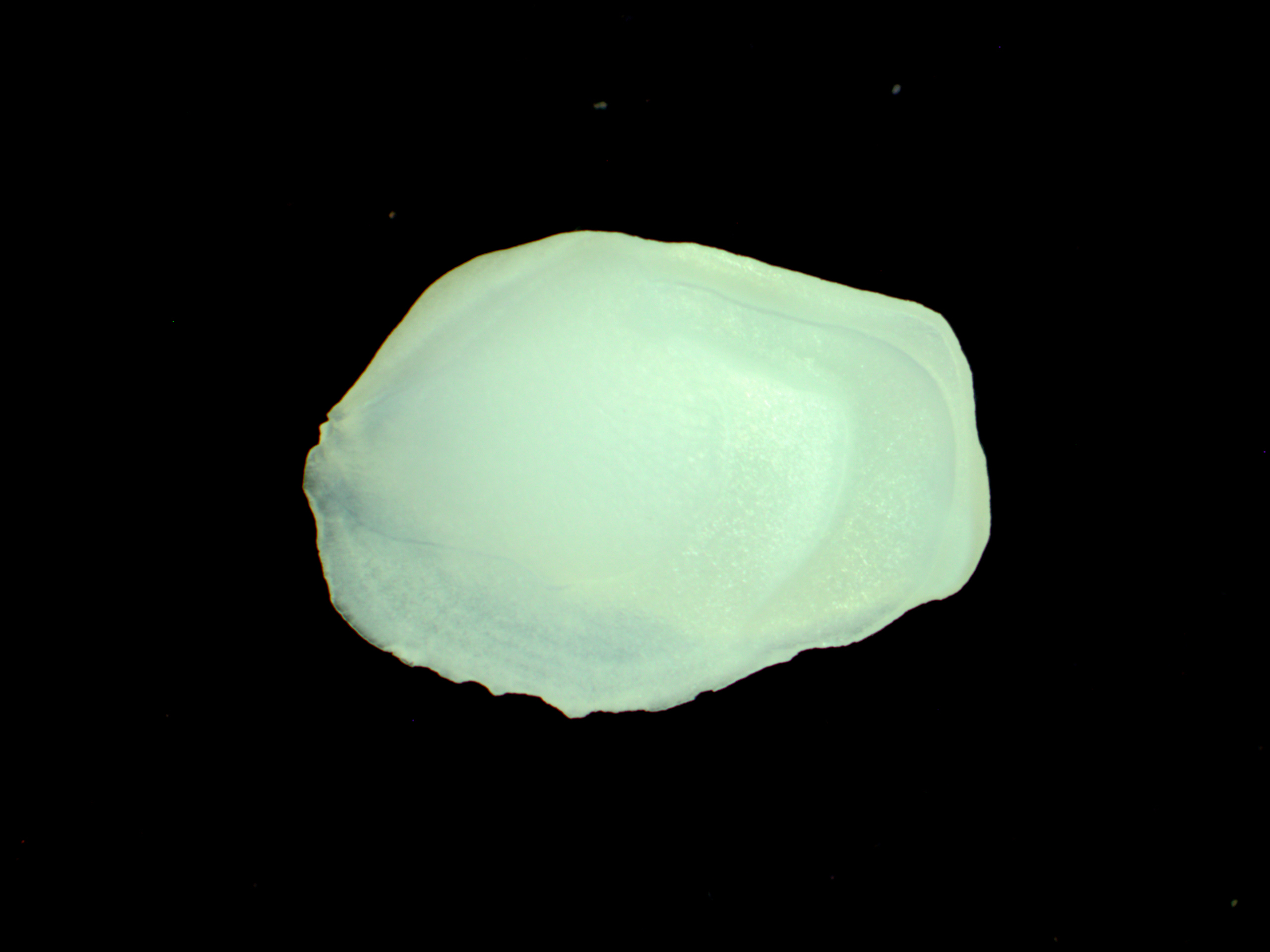

Supplement: Supplemental Information 11 [file peerj-04-1664-s011.zip › DenRus/training/7R1.jpg]

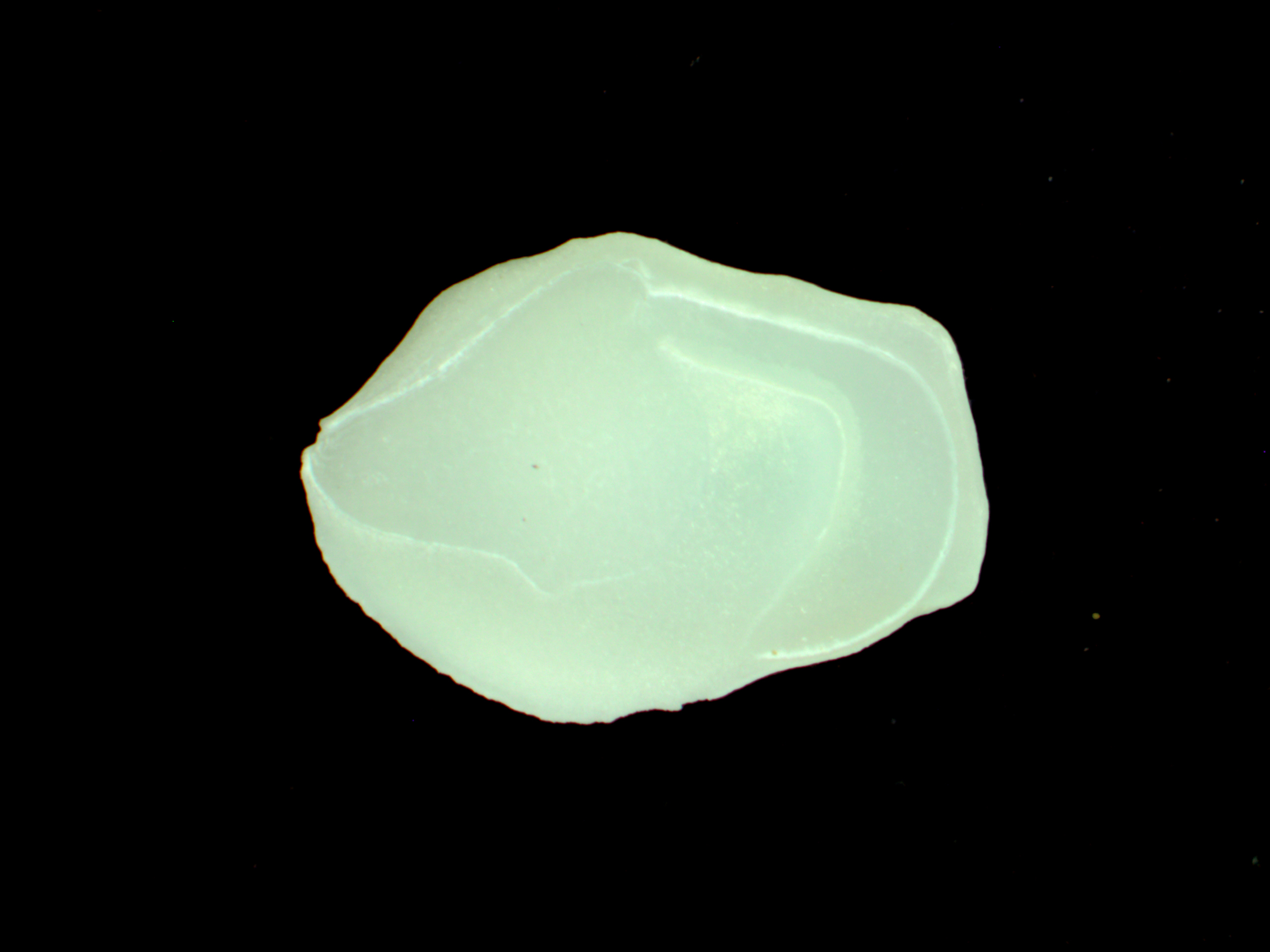

Supplement: Supplemental Information 11 [file peerj-04-1664-s011.zip › DenRus/training/F27R1.jpg]

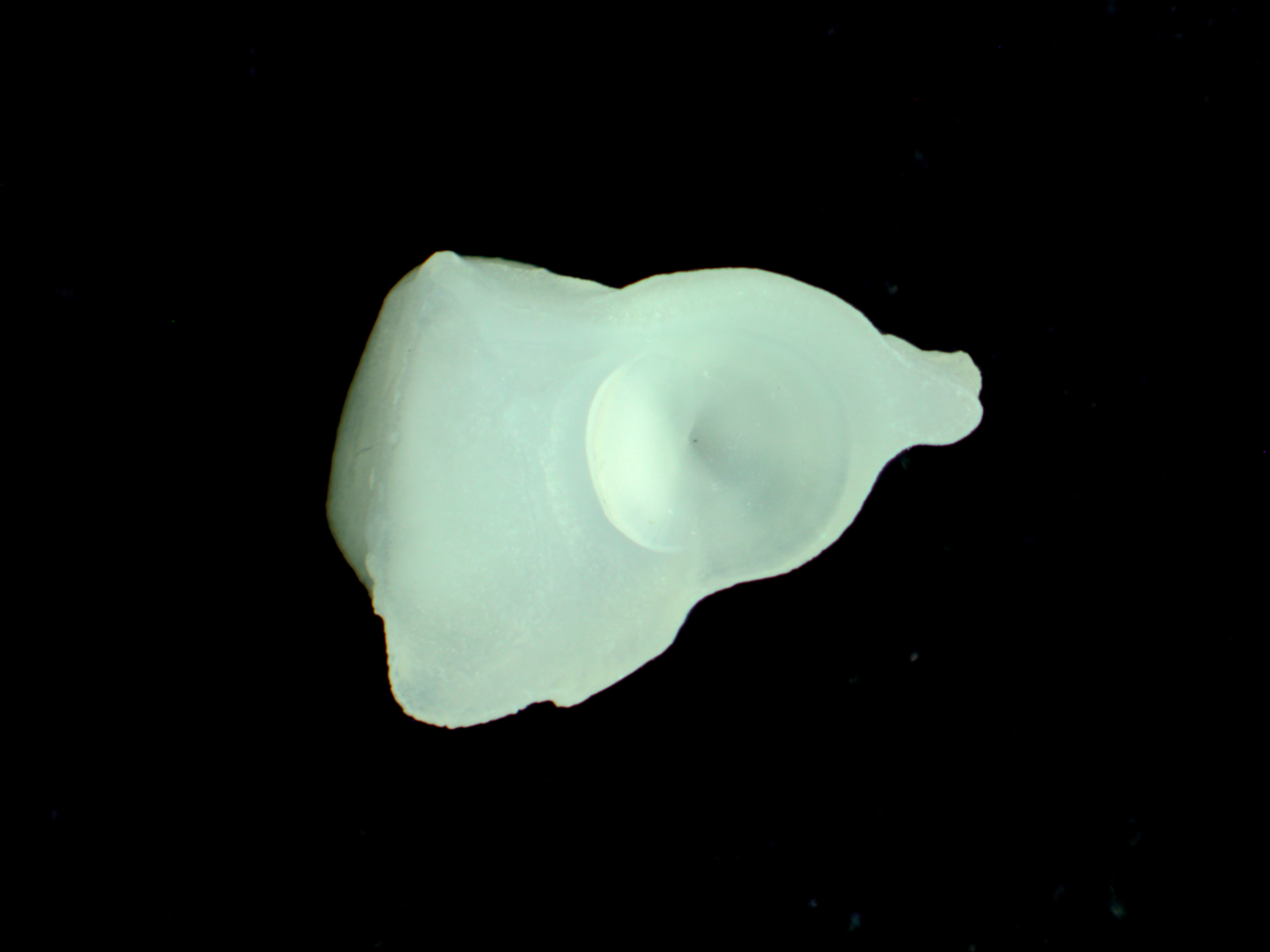

Supplement: Supplemental Information 12 [file peerj-04-1664-s012.zip › JohBel/testing/S78R1.jpg]

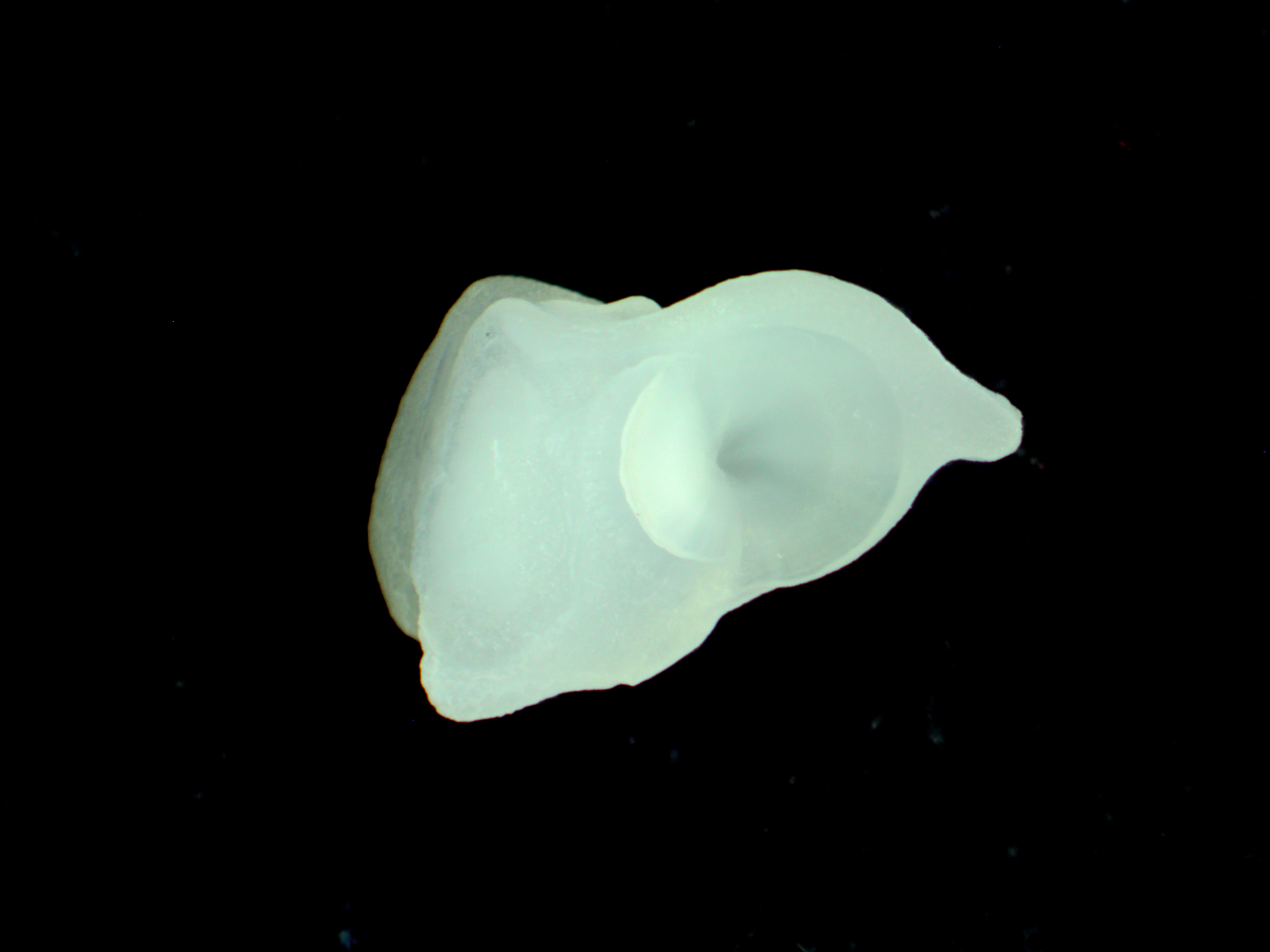

Supplement: Supplemental Information 12 [file peerj-04-1664-s012.zip › JohBel/testing/S79R1.jpg]

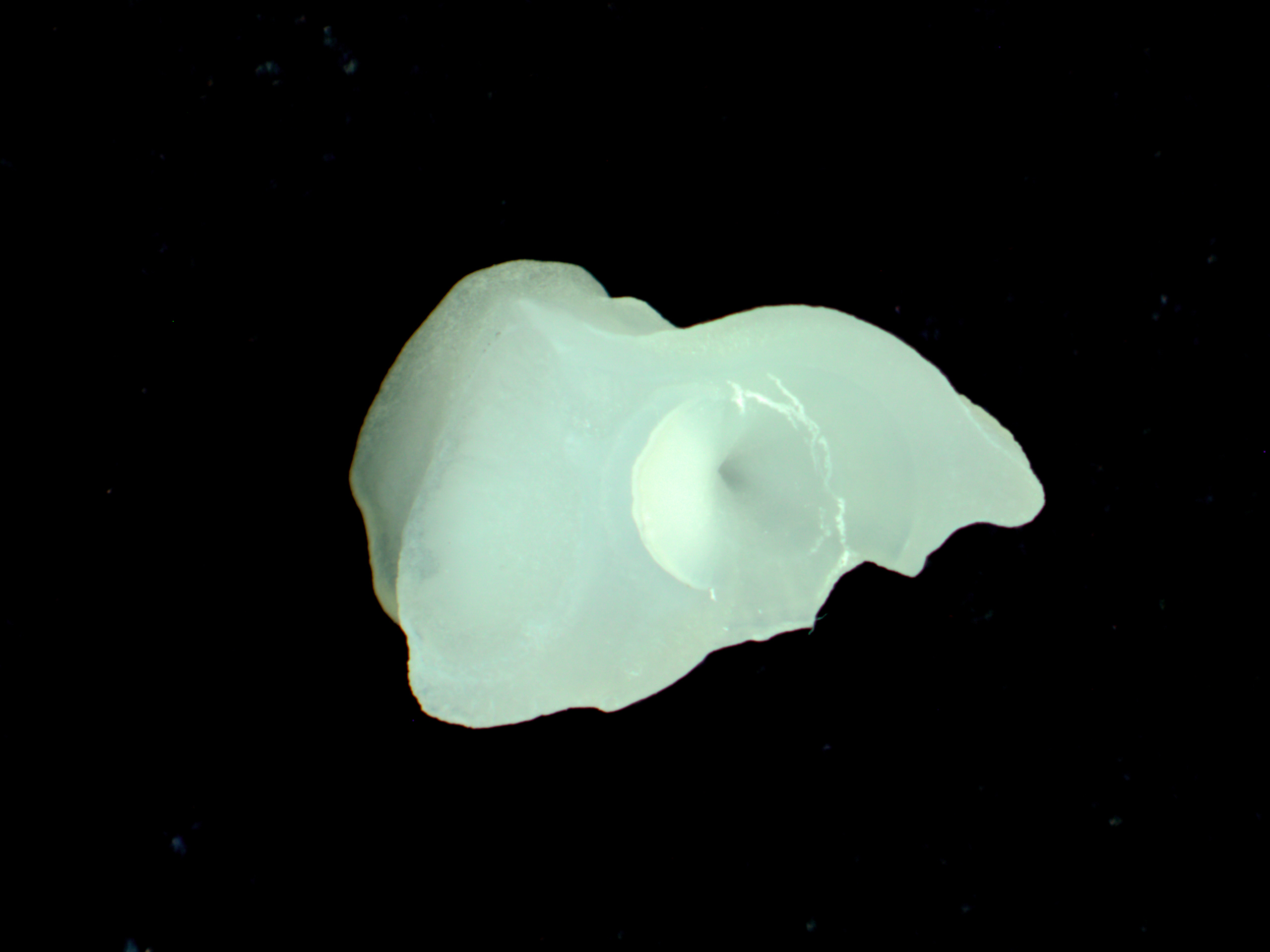

Supplement: Supplemental Information 12 [file peerj-04-1664-s012.zip › JohBel/testing/S80R1.jpg]

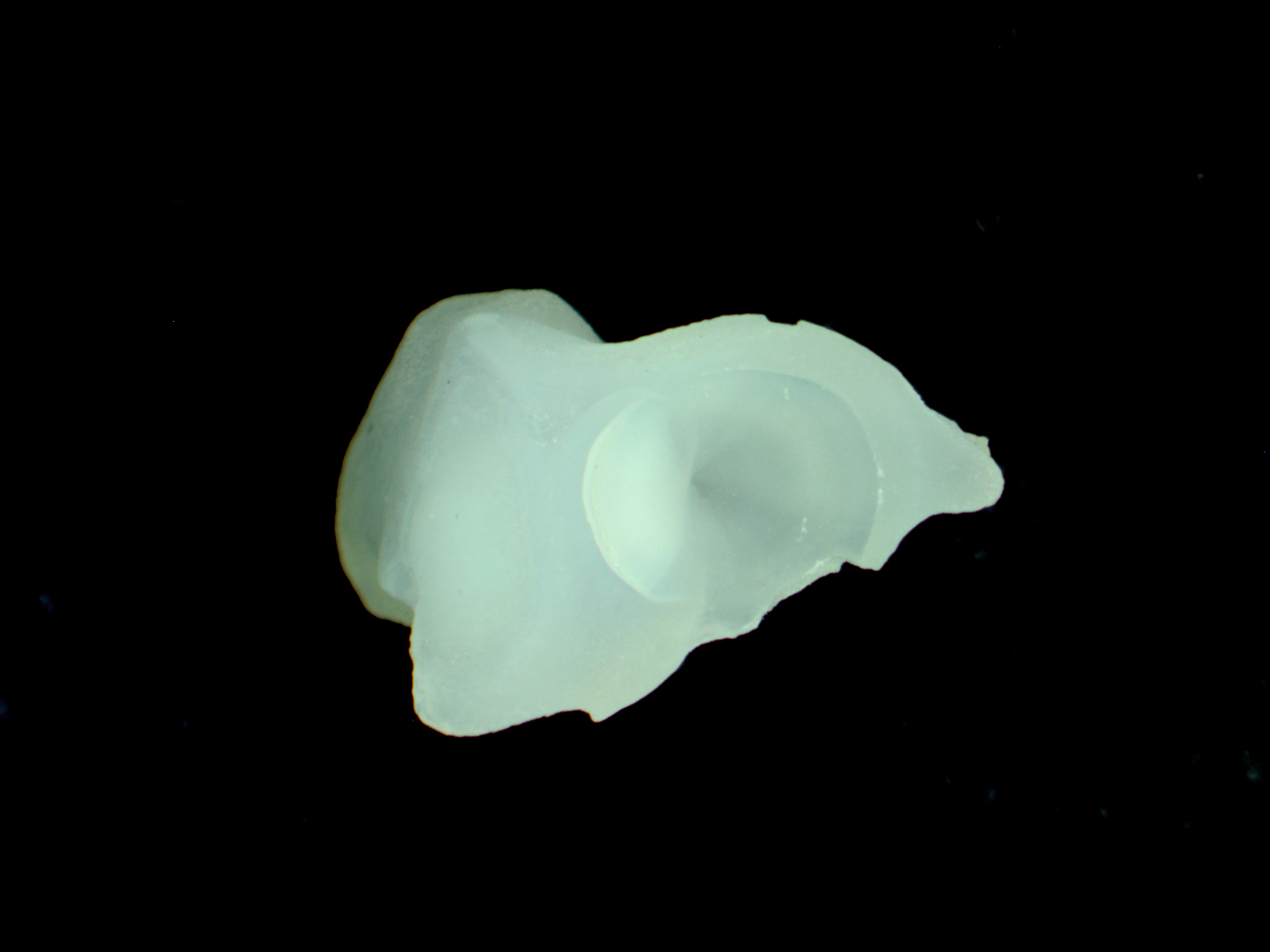

Supplement: Supplemental Information 12 [file peerj-04-1664-s012.zip › JohBel/testing/S81R1.jpg]

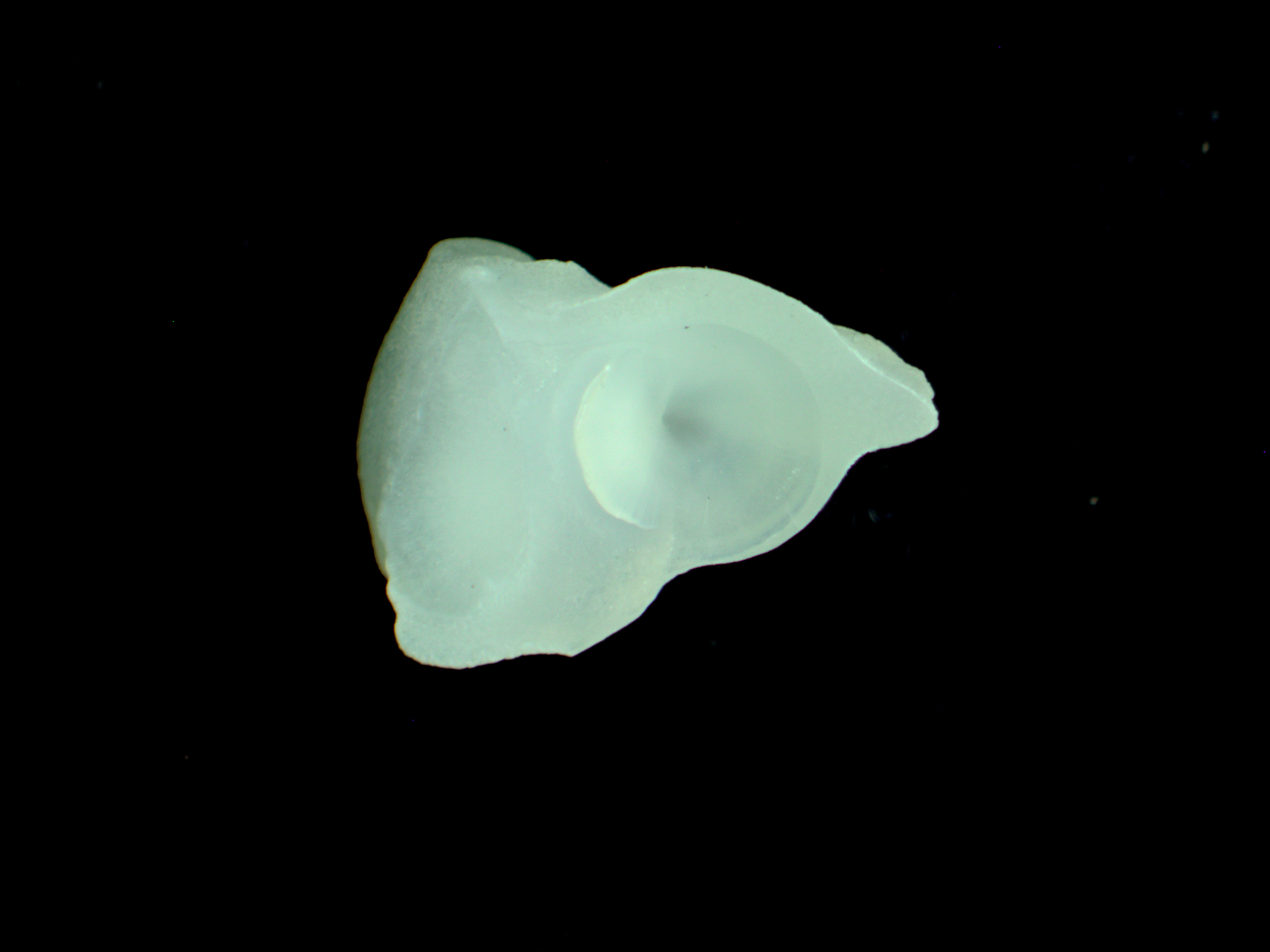

Supplement: Supplemental Information 12 [file peerj-04-1664-s012.zip › JohBel/testing/S82R1.jpg]

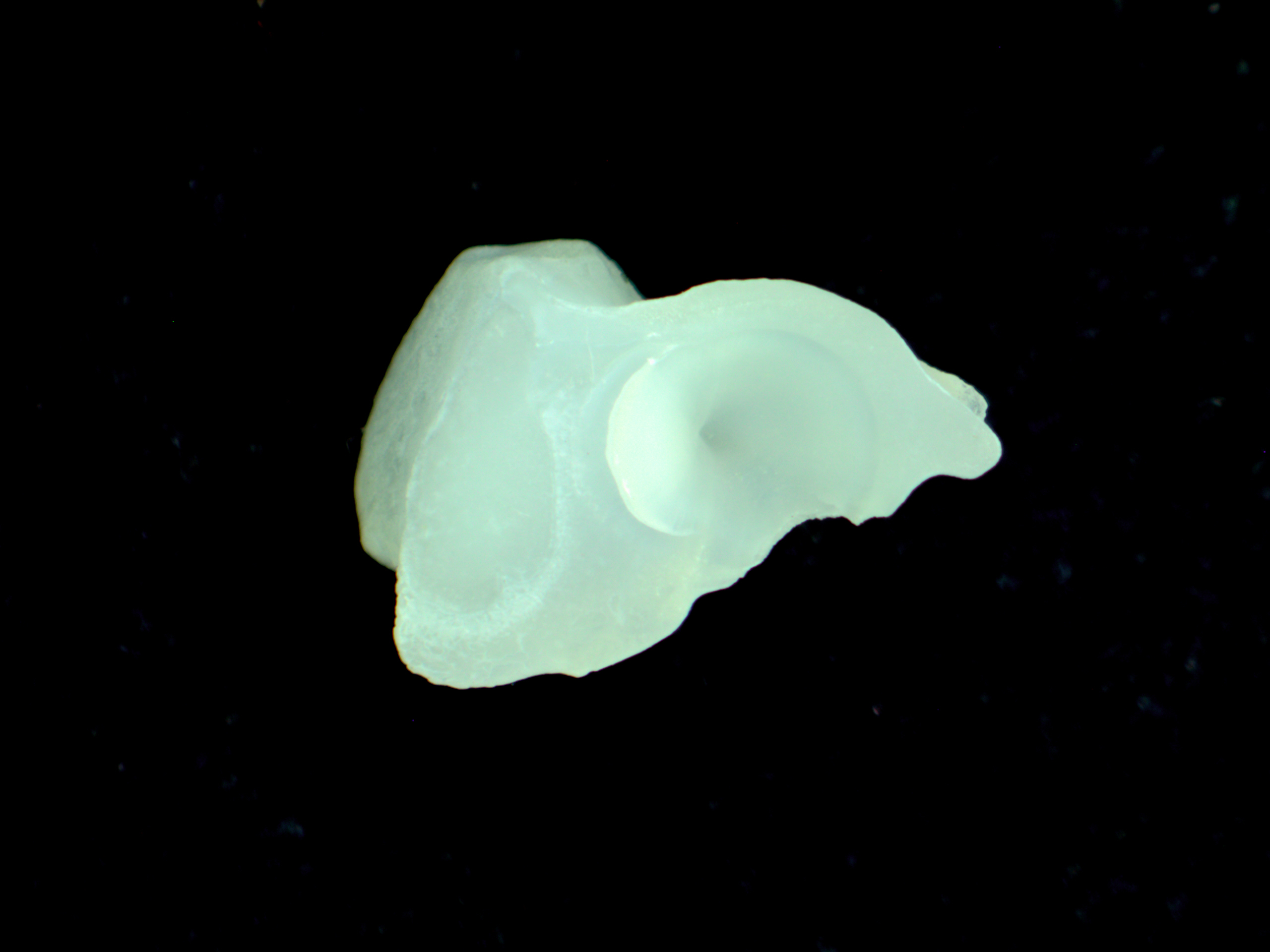

Supplement: Supplemental Information 12 [file peerj-04-1664-s012.zip › JohBel/testing/S83R1.jpg]

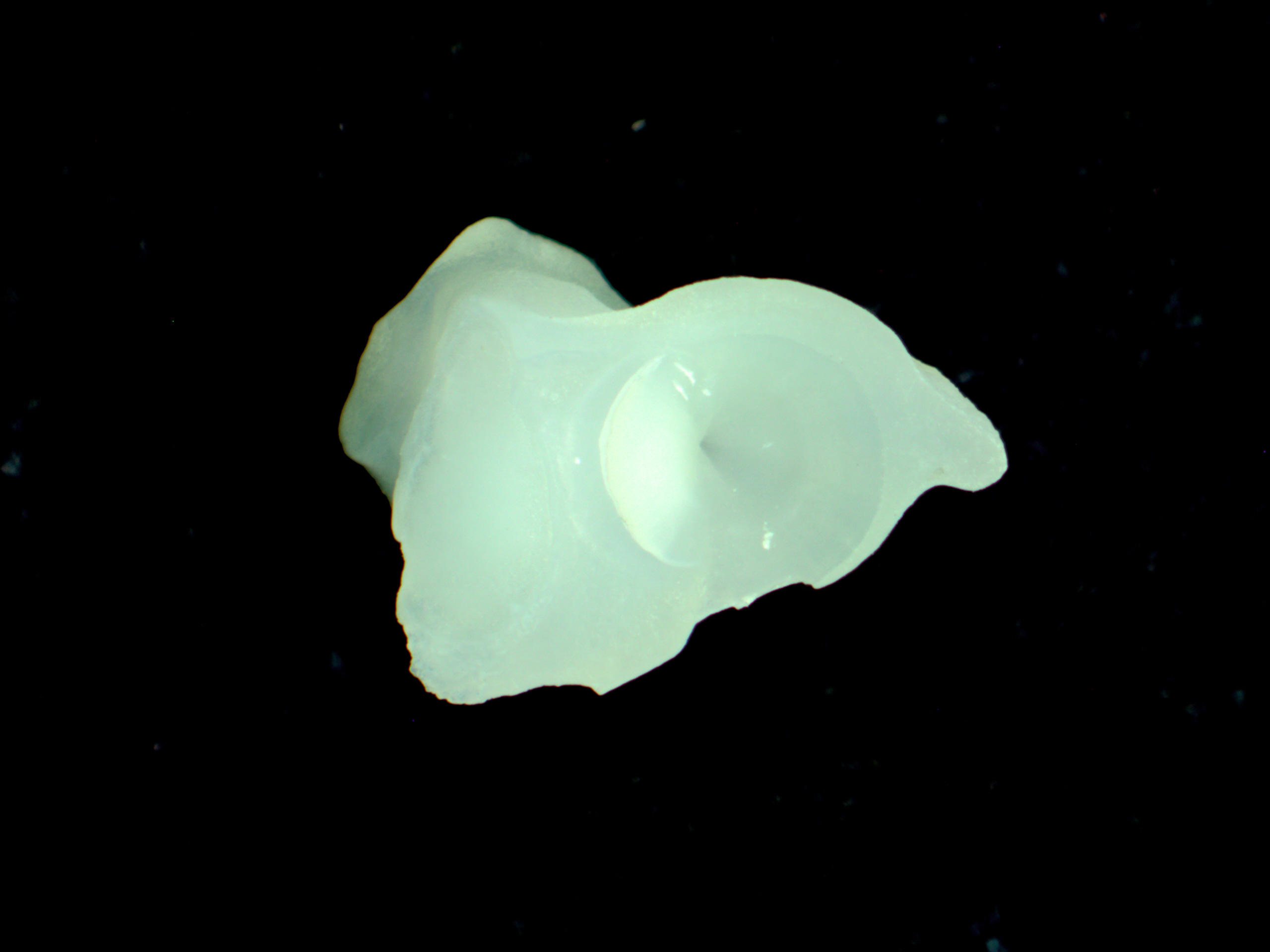

Supplement: Supplemental Information 12 [file peerj-04-1664-s012.zip › JohBel/testing/S84R1.jpg]

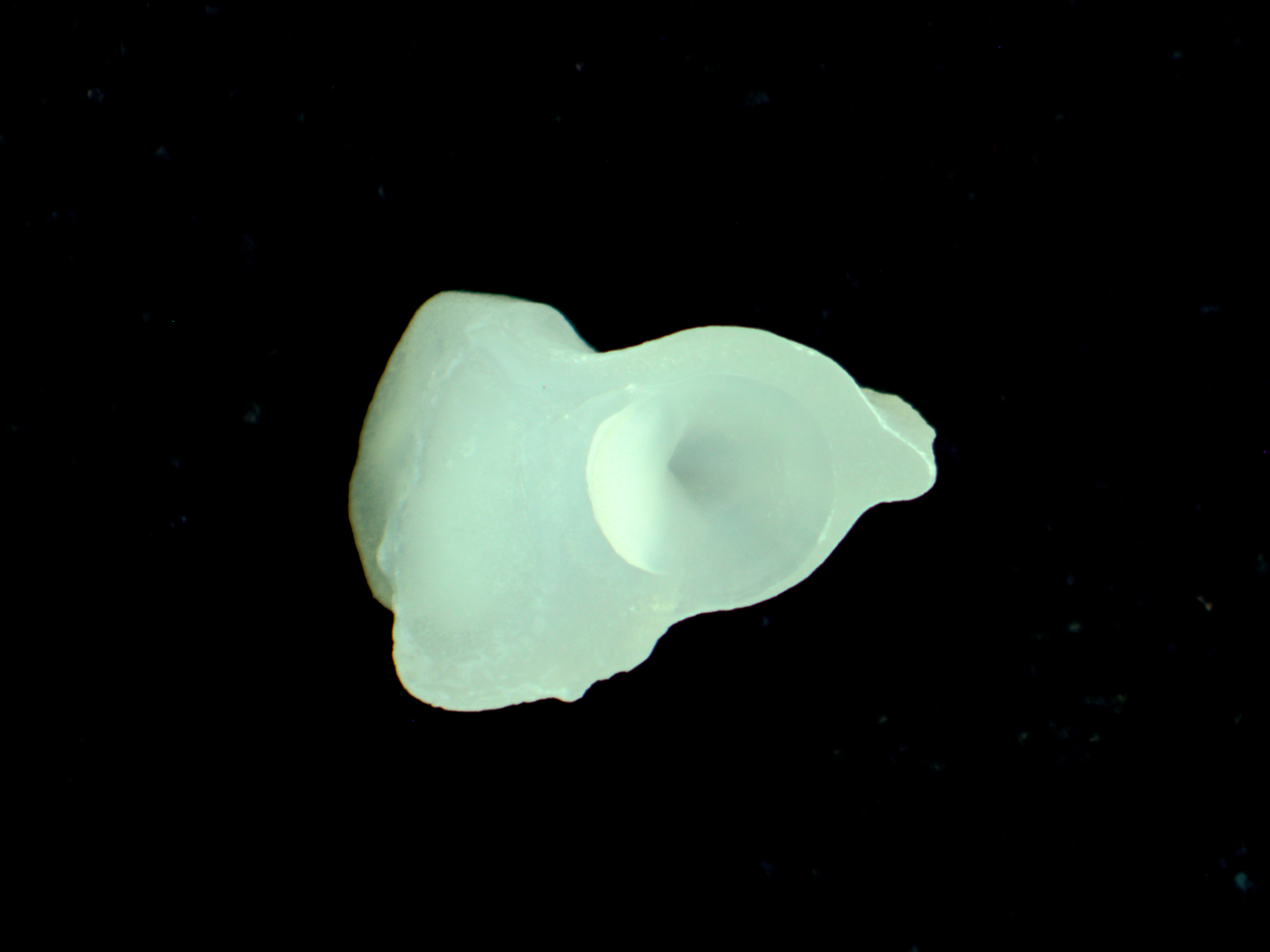

Supplement: Supplemental Information 12 [file peerj-04-1664-s012.zip › JohBel/testing/S85R1.jpg]

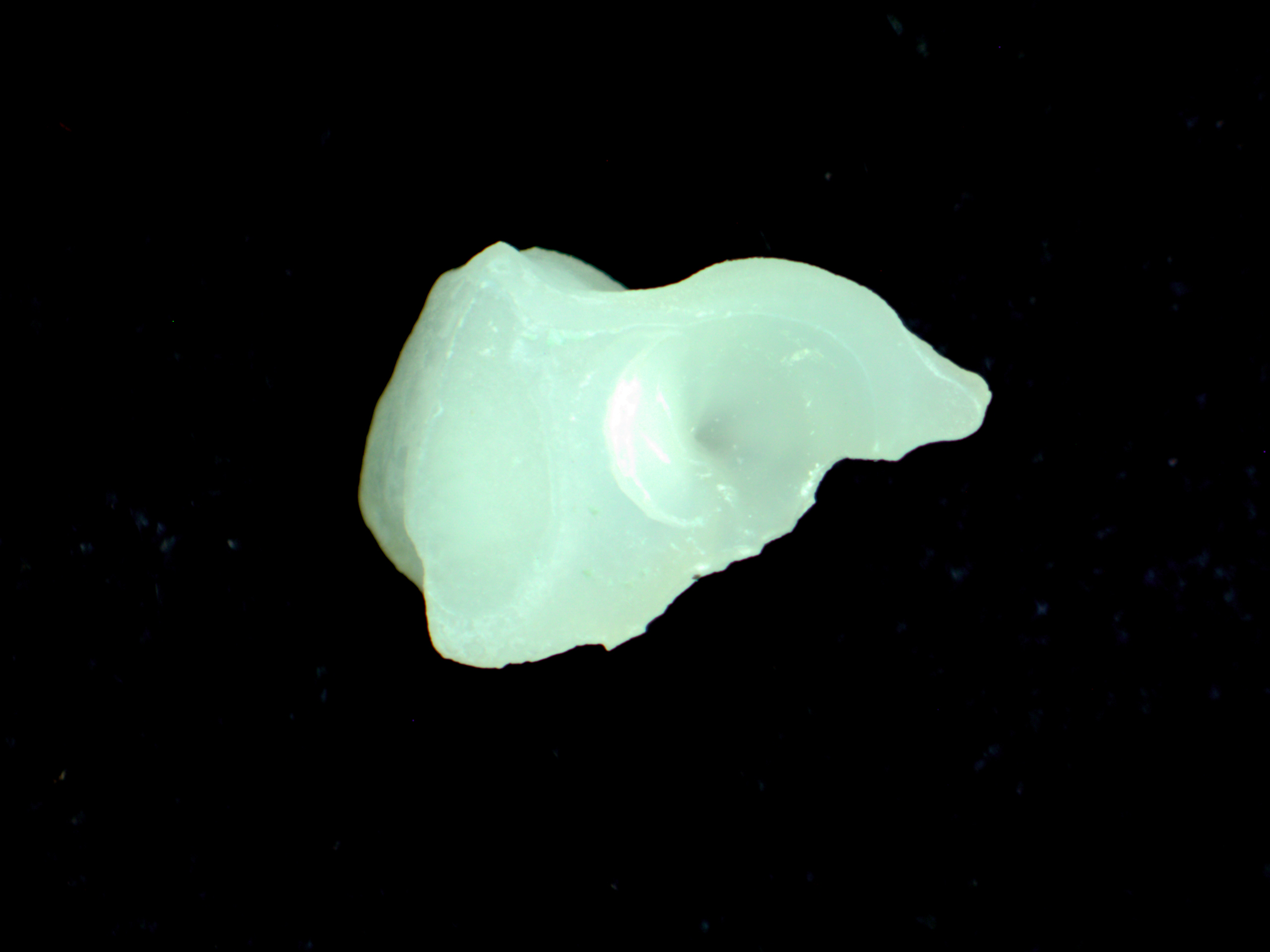

Supplement: Supplemental Information 12 [file peerj-04-1664-s012.zip › JohBel/testing/T26R1.jpg]

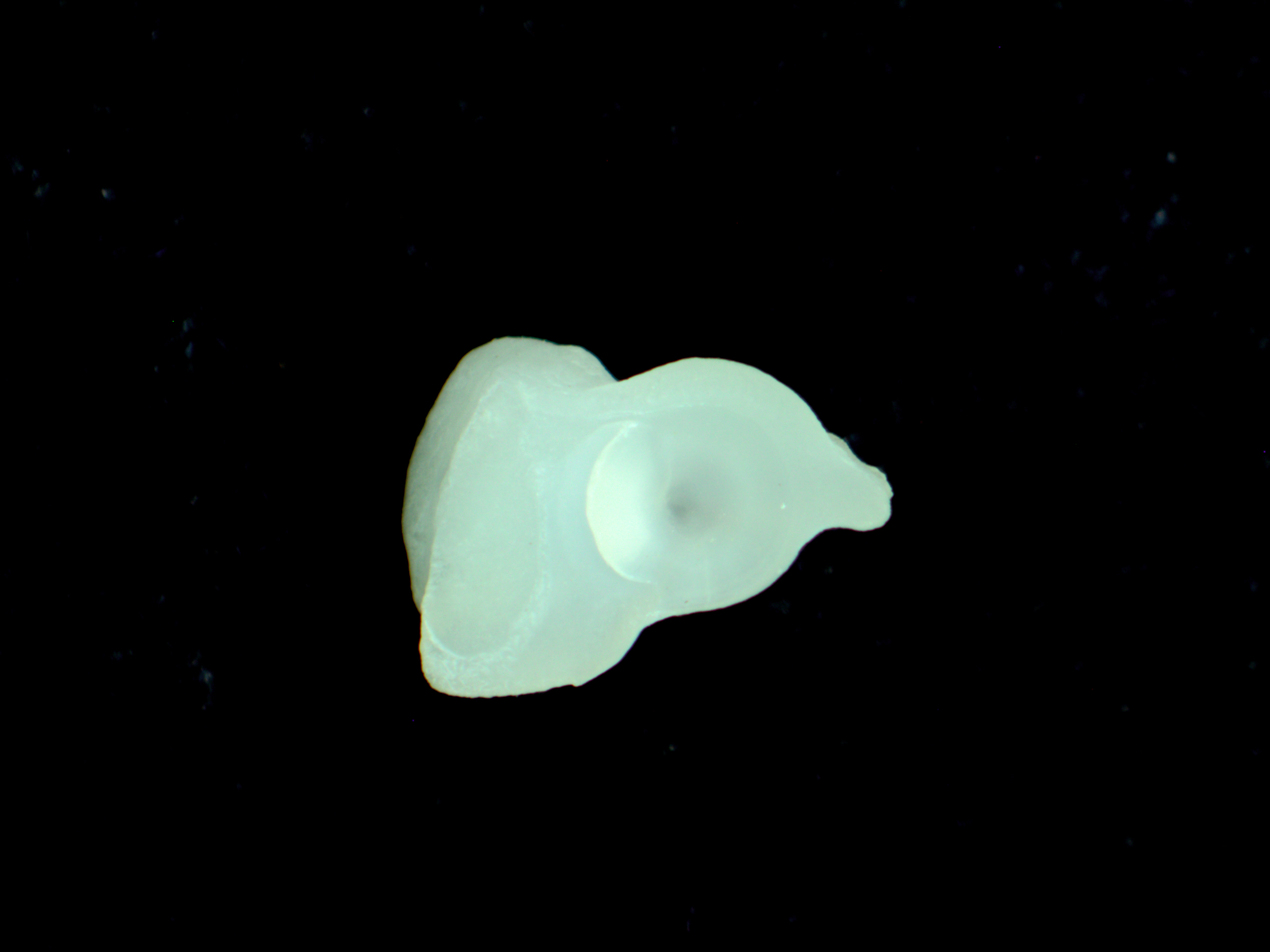

Supplement: Supplemental Information 12 [file peerj-04-1664-s012.zip › JohBel/testing/TO9R1.jpg]

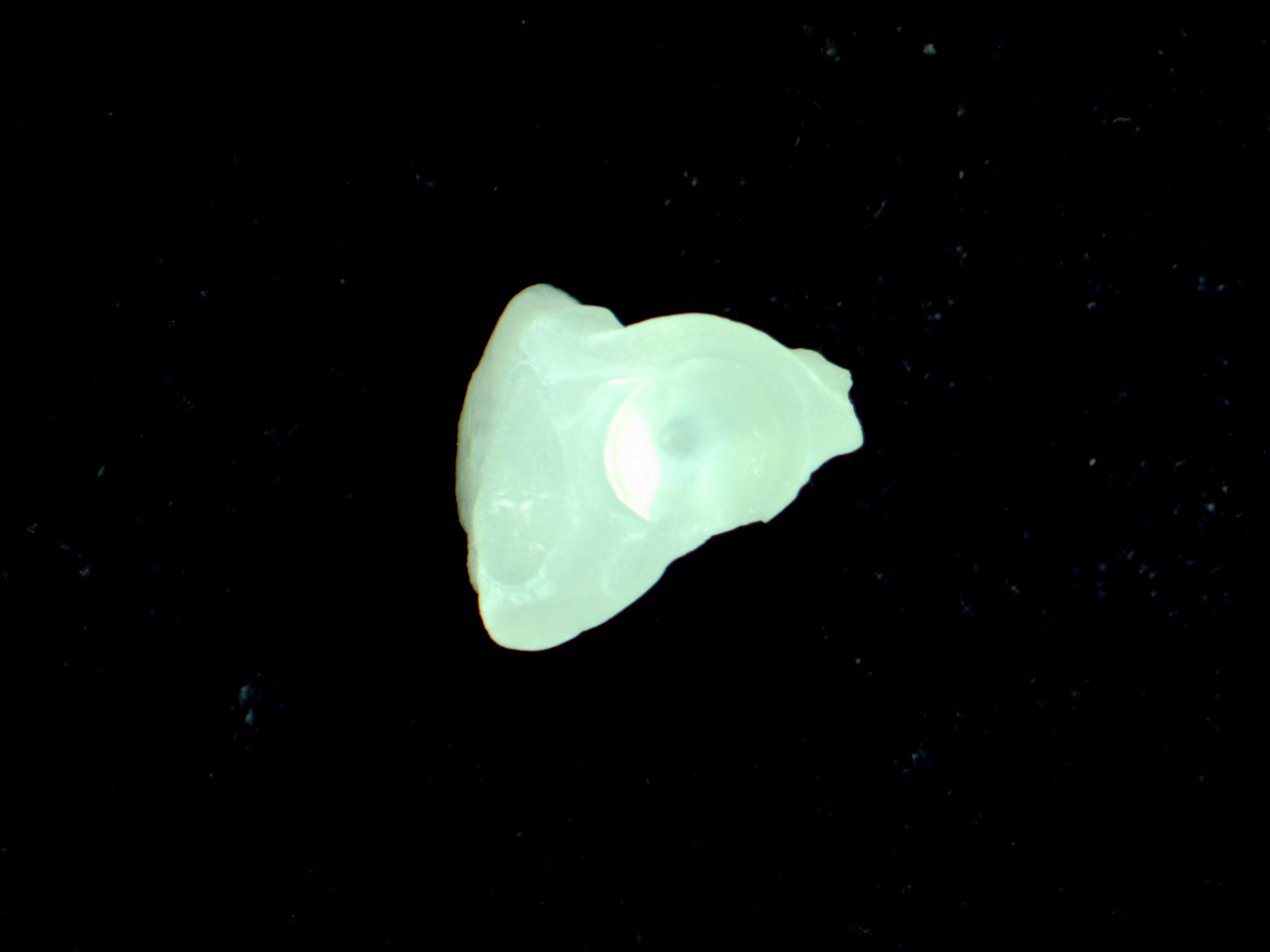

Supplement: Supplemental Information 12 [file peerj-04-1664-s012.zip › JohBel/training/F39R1.jpg]

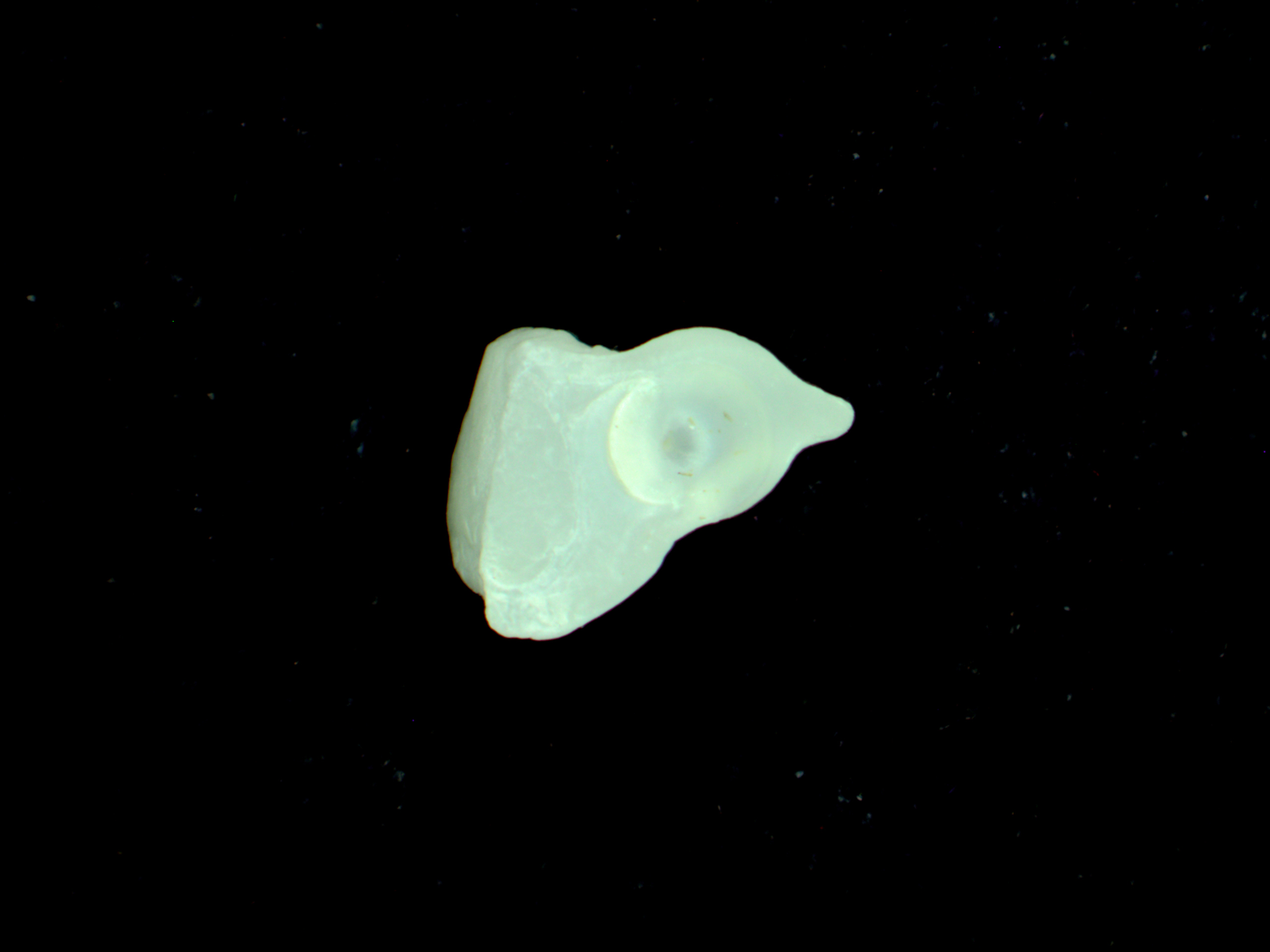

Supplement: Supplemental Information 12 [file peerj-04-1664-s012.zip › JohBel/training/O56R1.jpg]

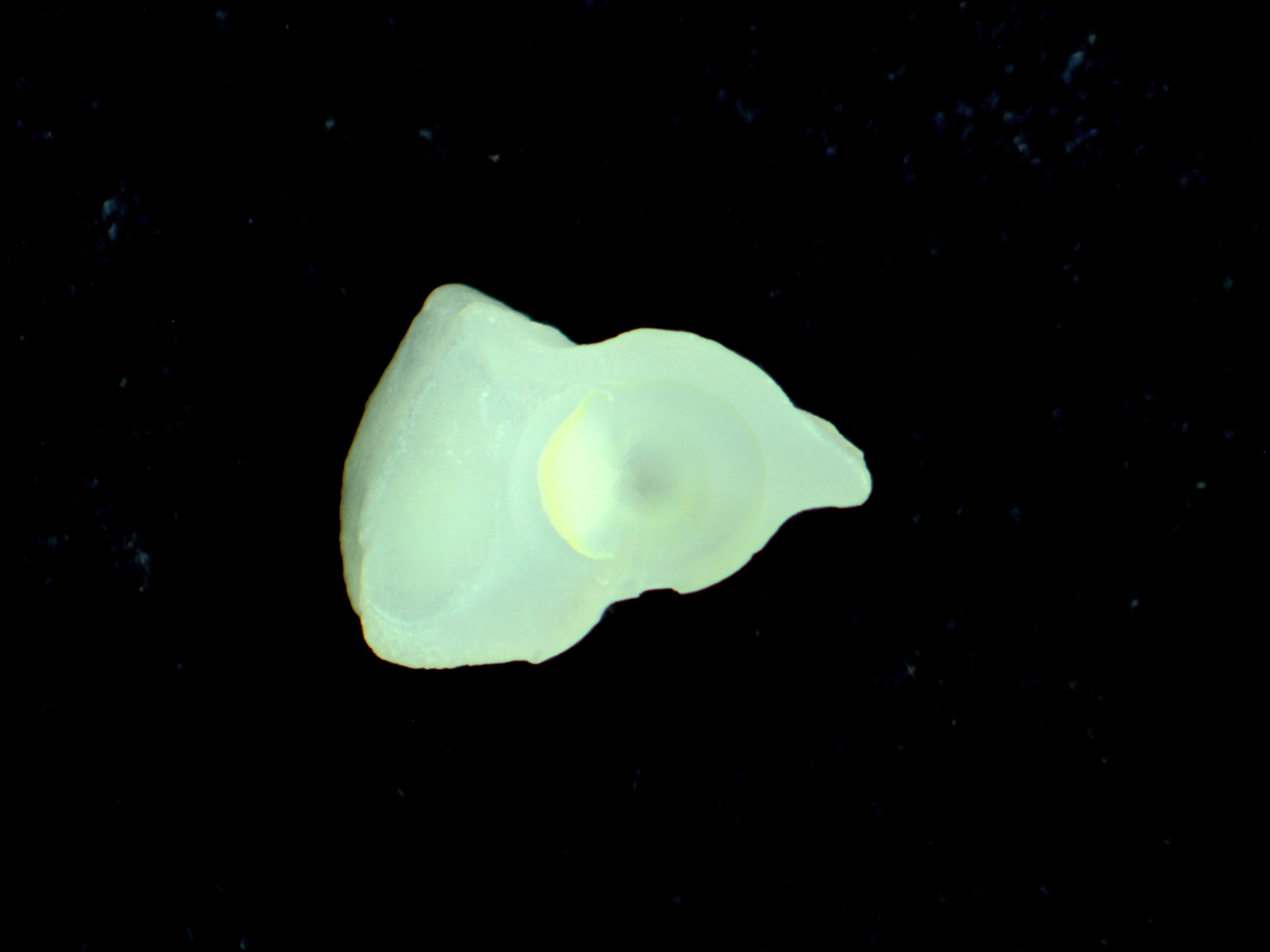

Supplement: Supplemental Information 12 [file peerj-04-1664-s012.zip › JohBel/training/Q02R1.jpg]
