# Supplementary figures and images for: Fully-automated identification of fish species based on otolith contour: using short-time Fourier transform and discriminant analysis (STFT-DA) (part 4 of 5)
Source: PeerJ. 2016 Feb 22;4:e1664. doi: 10.7717/peerj.1664 (PMC4768690; doi:10.7717/peerj.1664)

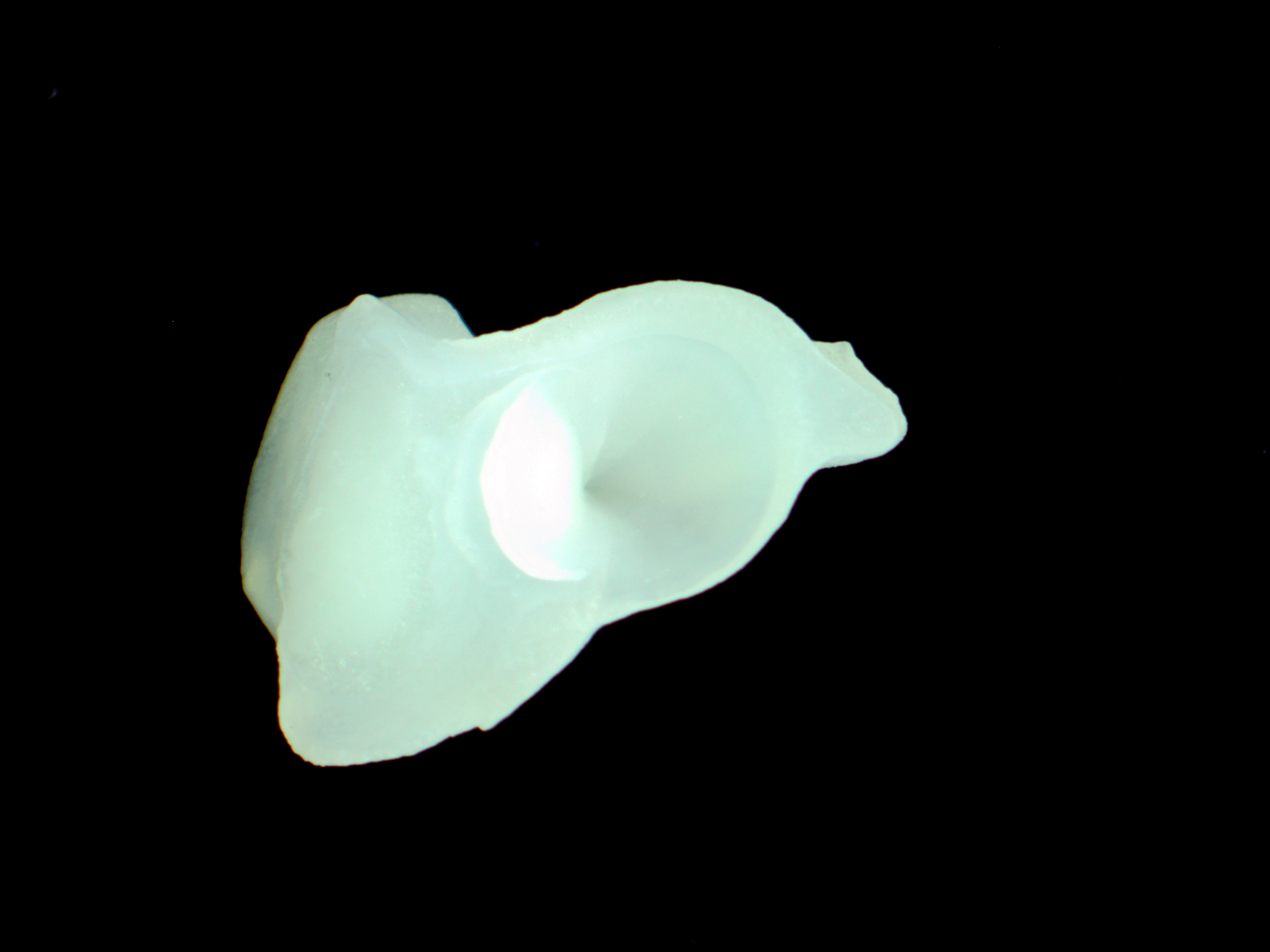

Supplement: Supplemental Information 12 [file peerj-04-1664-s012.zip › JohBel/training/S10R1.jpg]

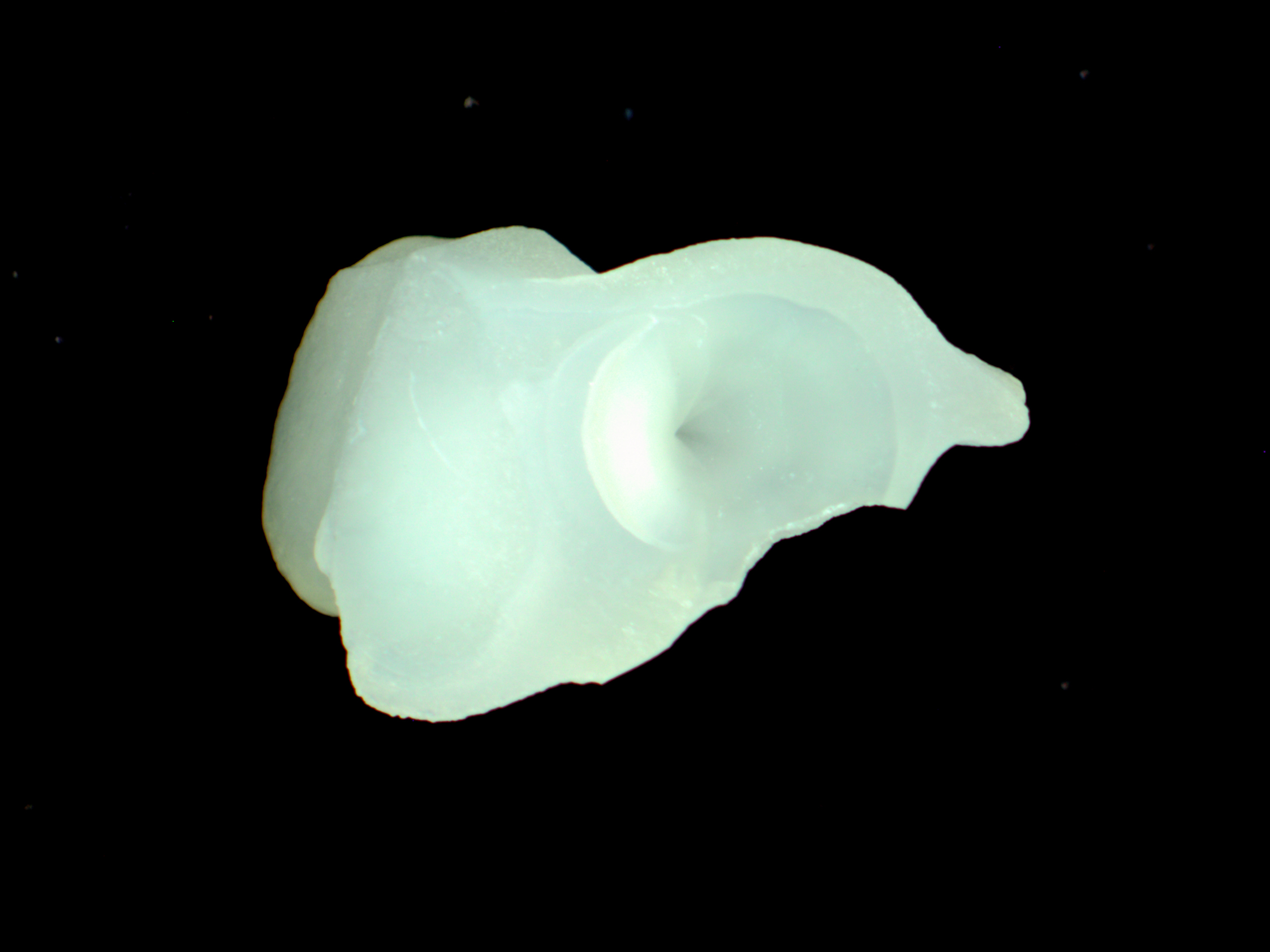

Supplement: Supplemental Information 12 [file peerj-04-1664-s012.zip › JohBel/training/S1R1.jpg]

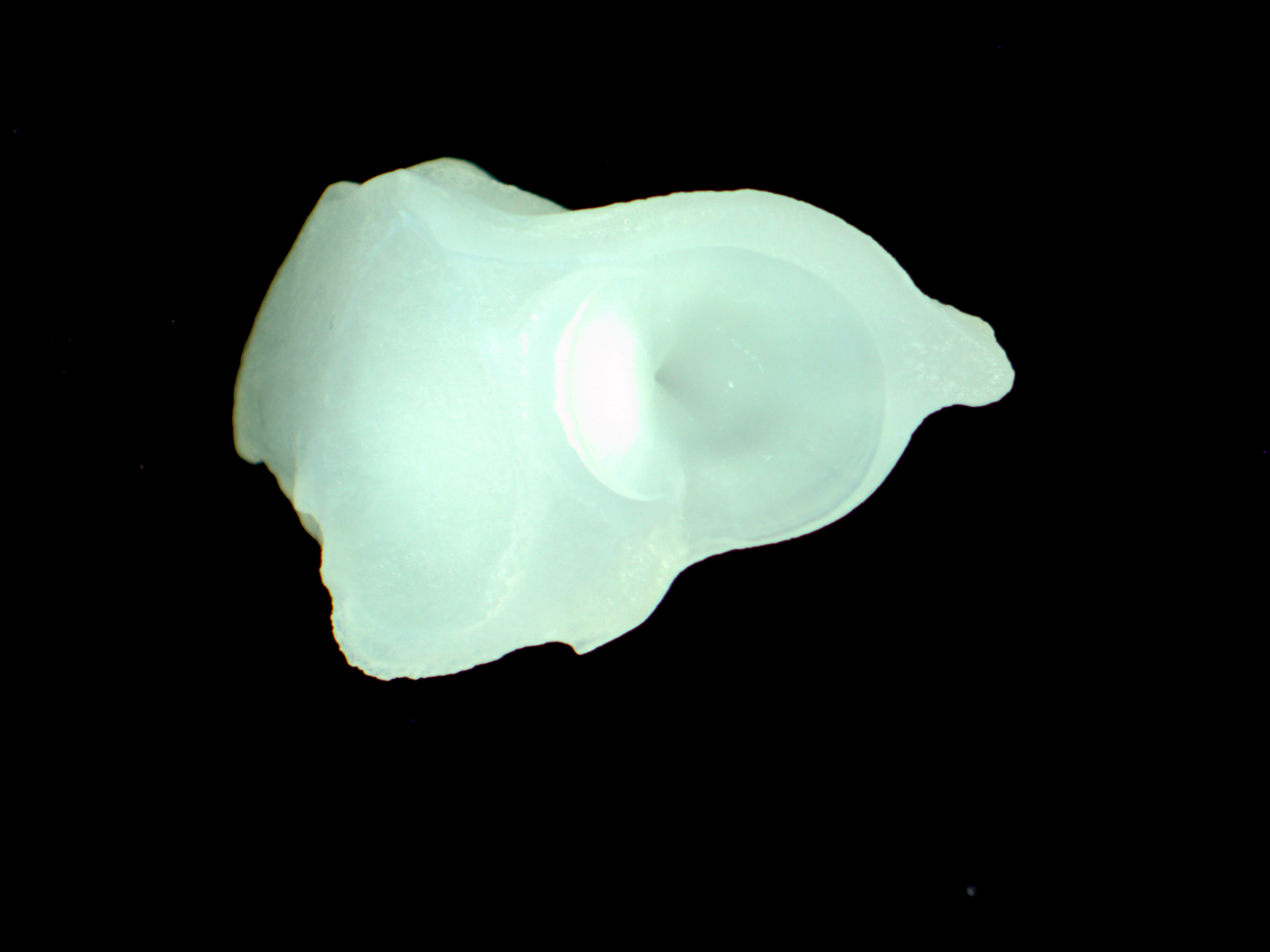

Supplement: Supplemental Information 12 [file peerj-04-1664-s012.zip › JohBel/training/S2R1.jpg]

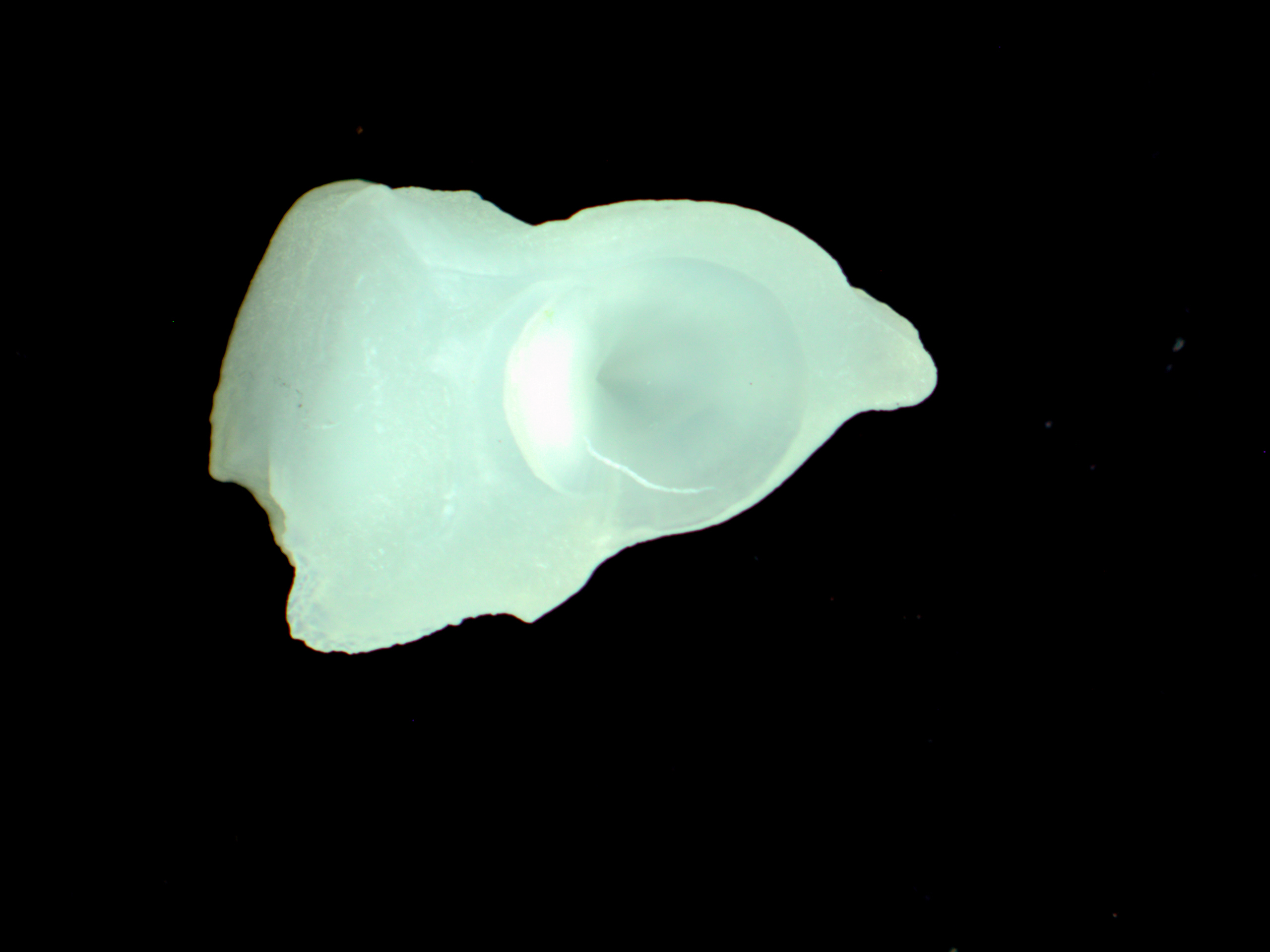

Supplement: Supplemental Information 12 [file peerj-04-1664-s012.zip › JohBel/training/S3R1.jpg]

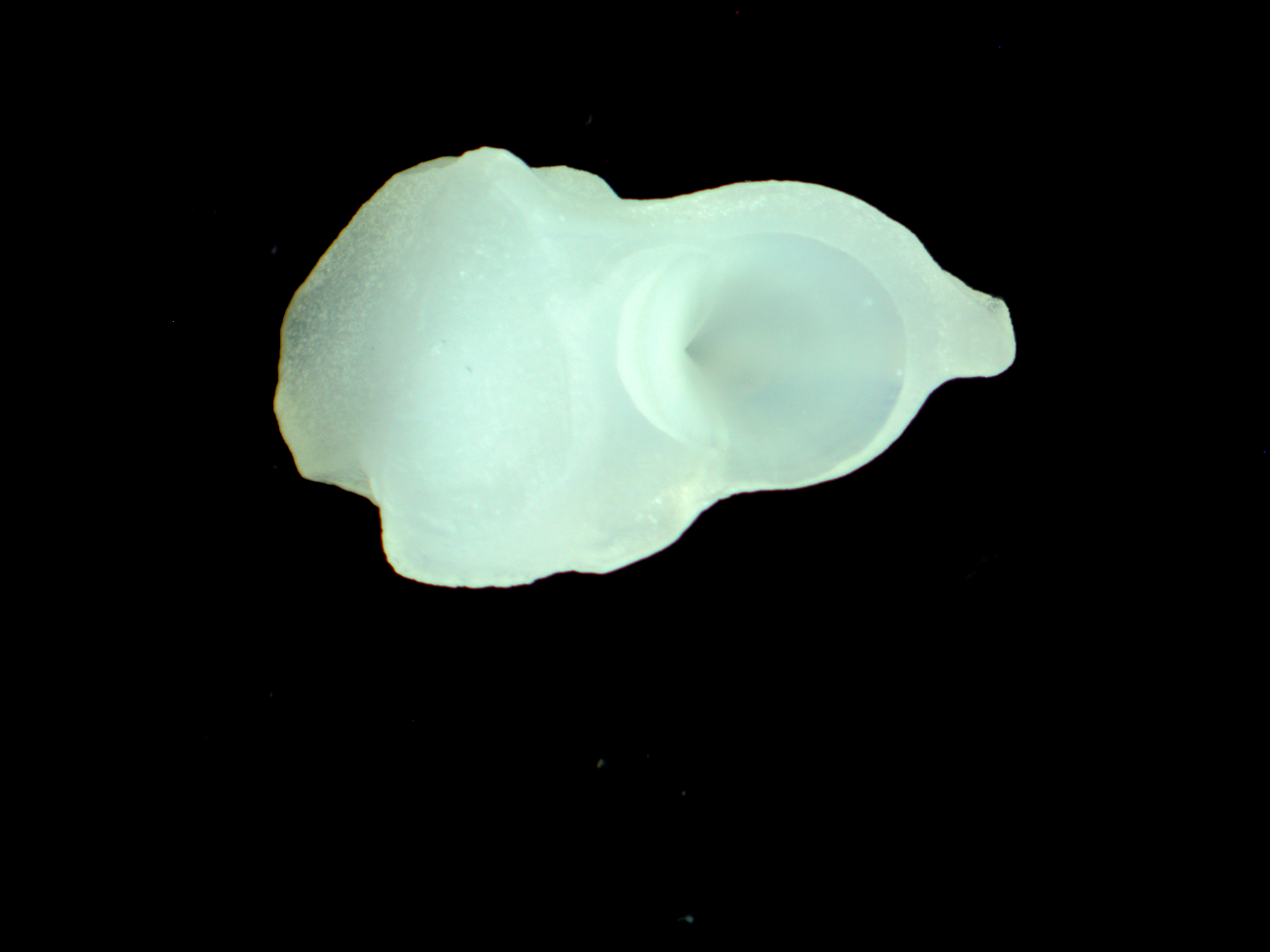

Supplement: Supplemental Information 12 [file peerj-04-1664-s012.zip › JohBel/training/S4R1.jpg]

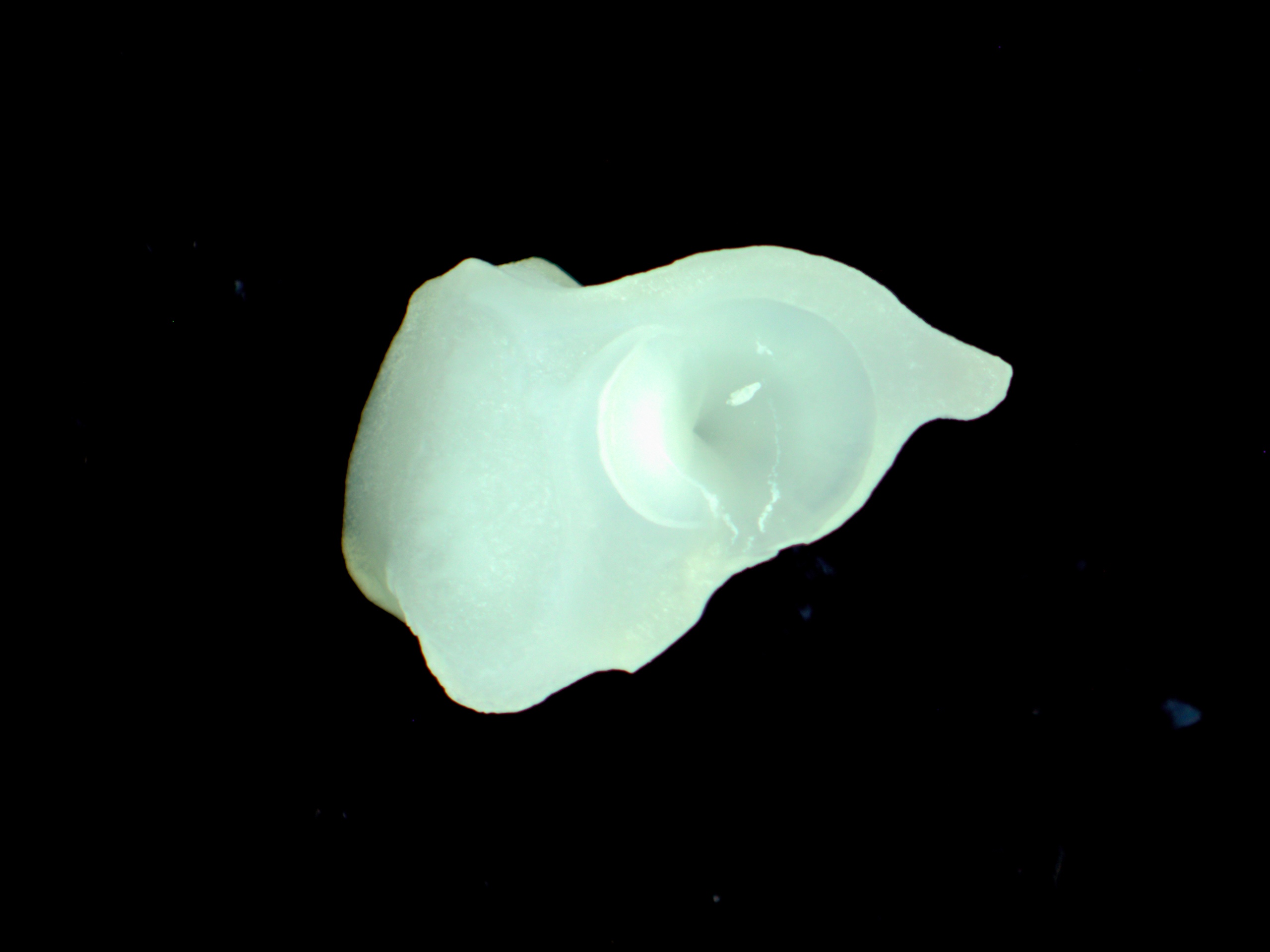

Supplement: Supplemental Information 12 [file peerj-04-1664-s012.zip › JohBel/training/S5R1.jpg]

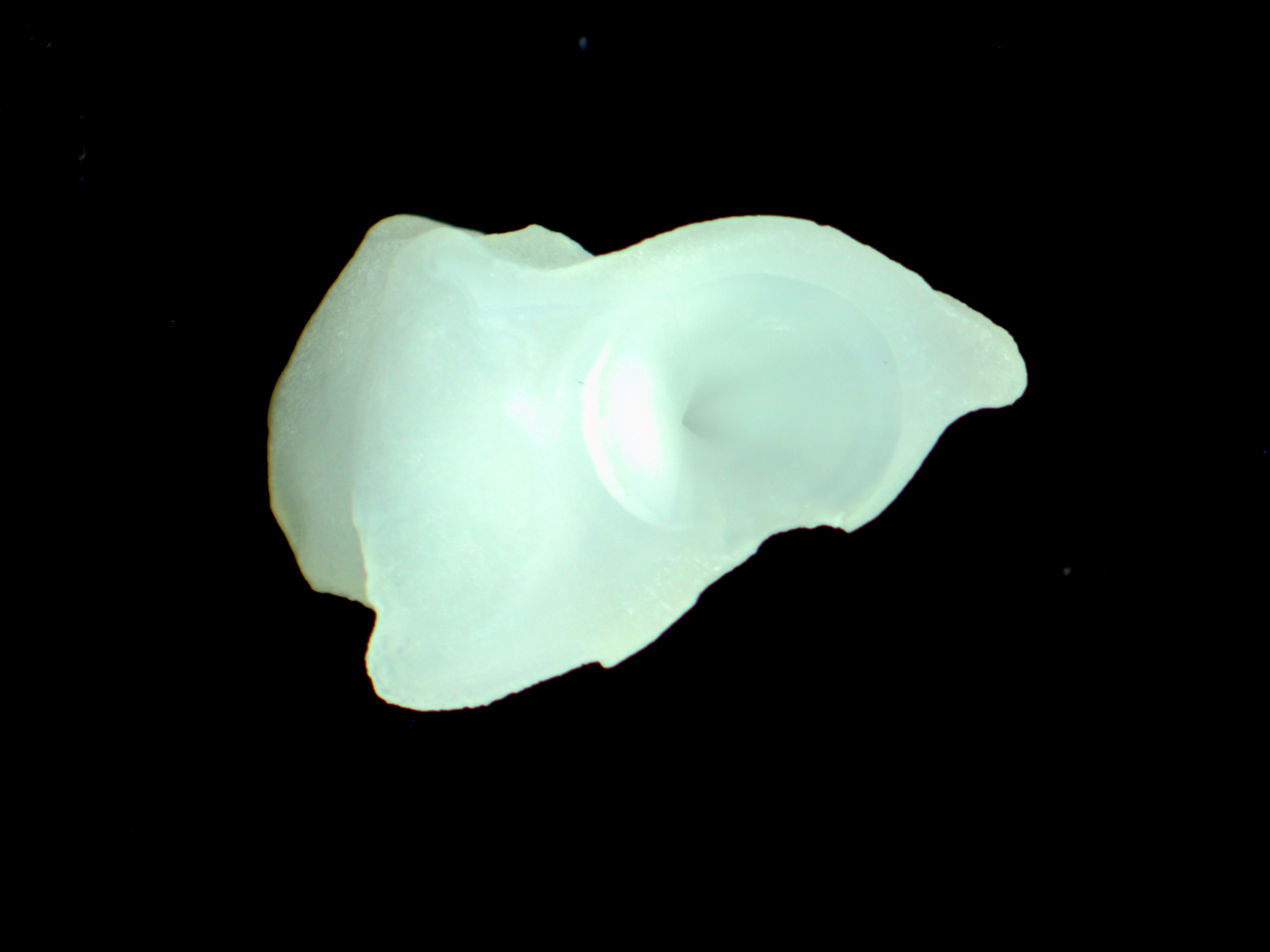

Supplement: Supplemental Information 12 [file peerj-04-1664-s012.zip › JohBel/training/S6R1.jpg]

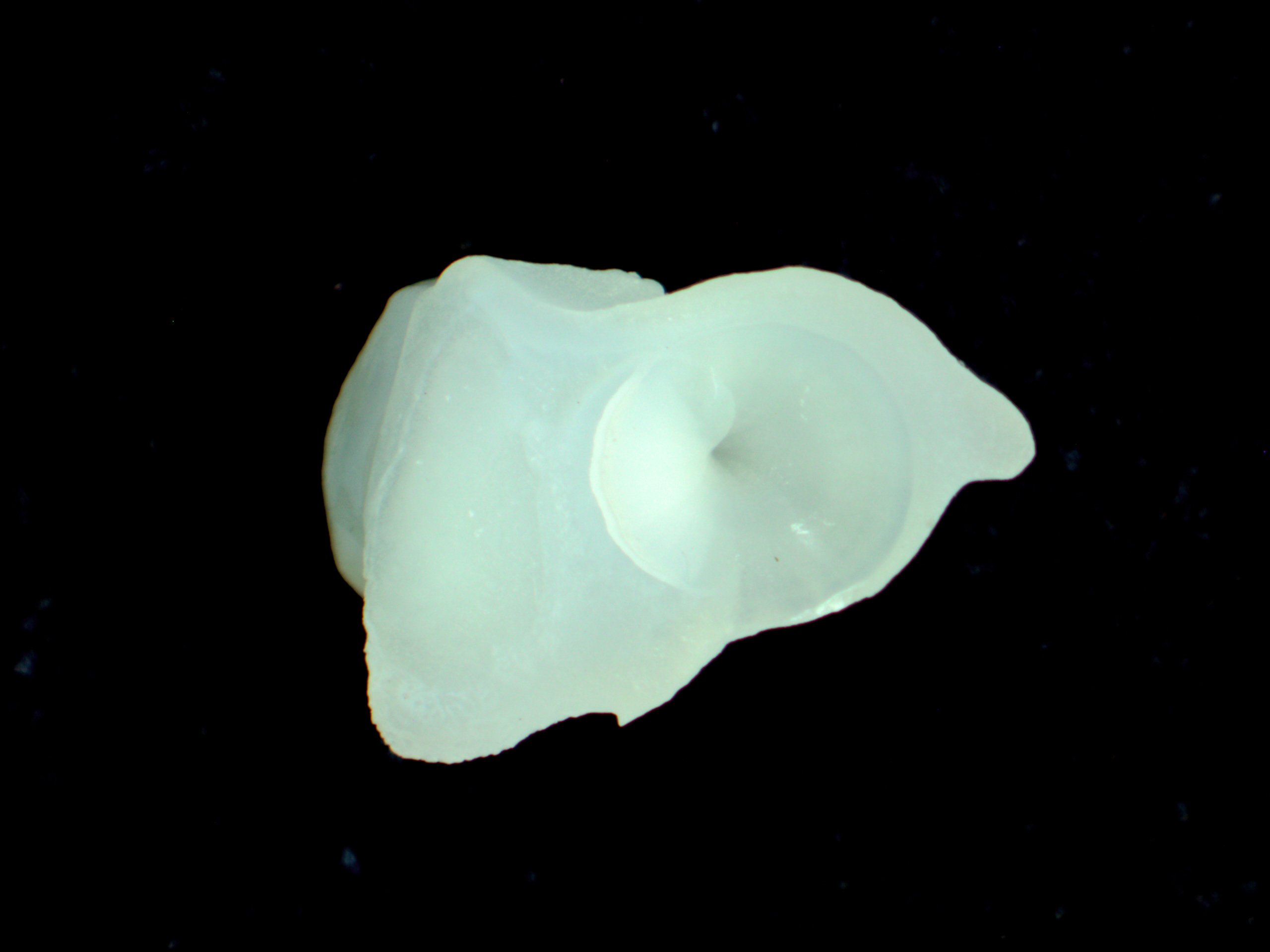

Supplement: Supplemental Information 12 [file peerj-04-1664-s012.zip › JohBel/training/S71R1.jpg]

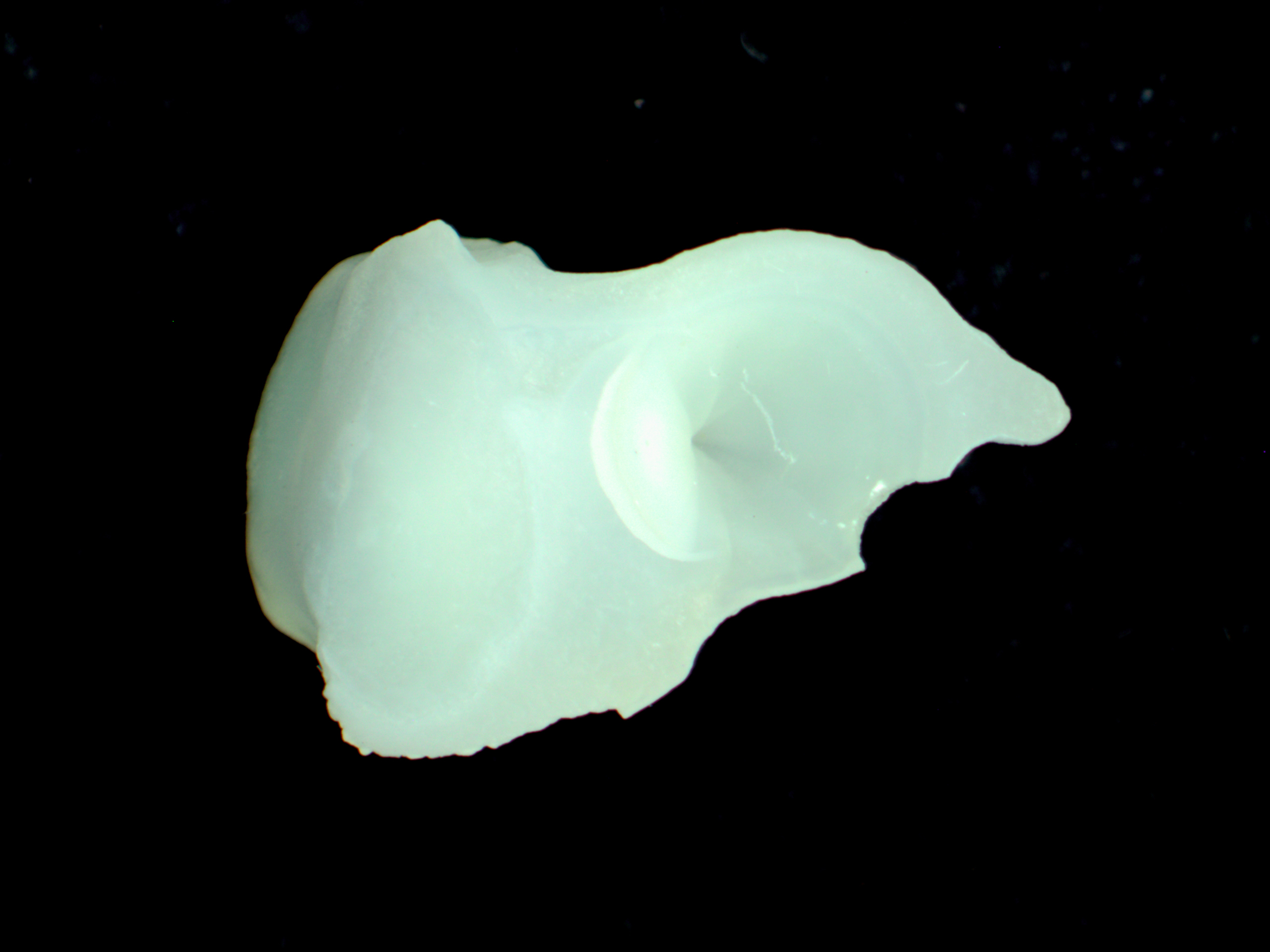

Supplement: Supplemental Information 12 [file peerj-04-1664-s012.zip › JohBel/training/S72R1.jpg]

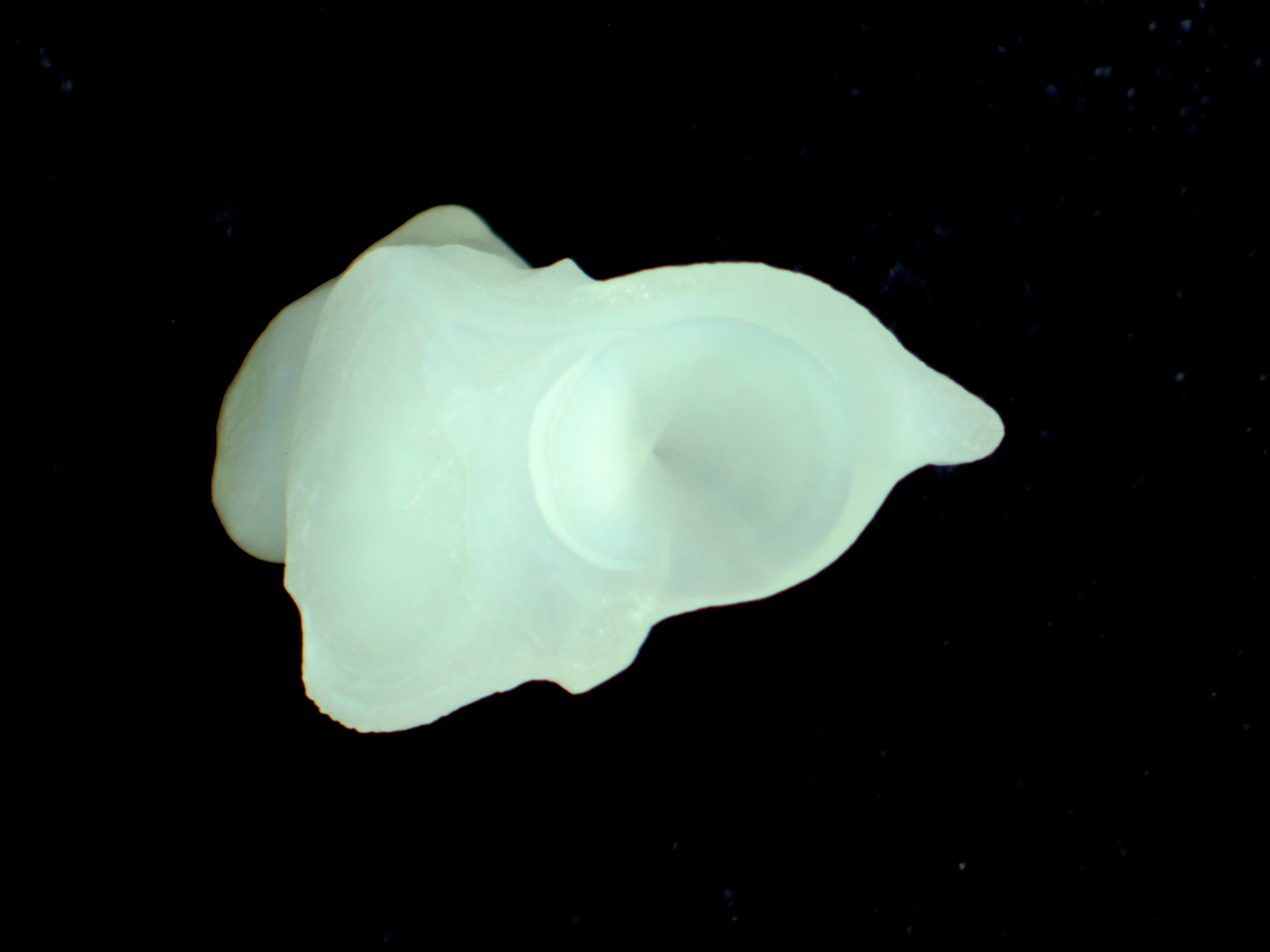

Supplement: Supplemental Information 12 [file peerj-04-1664-s012.zip › JohBel/training/S73R1.jpg]

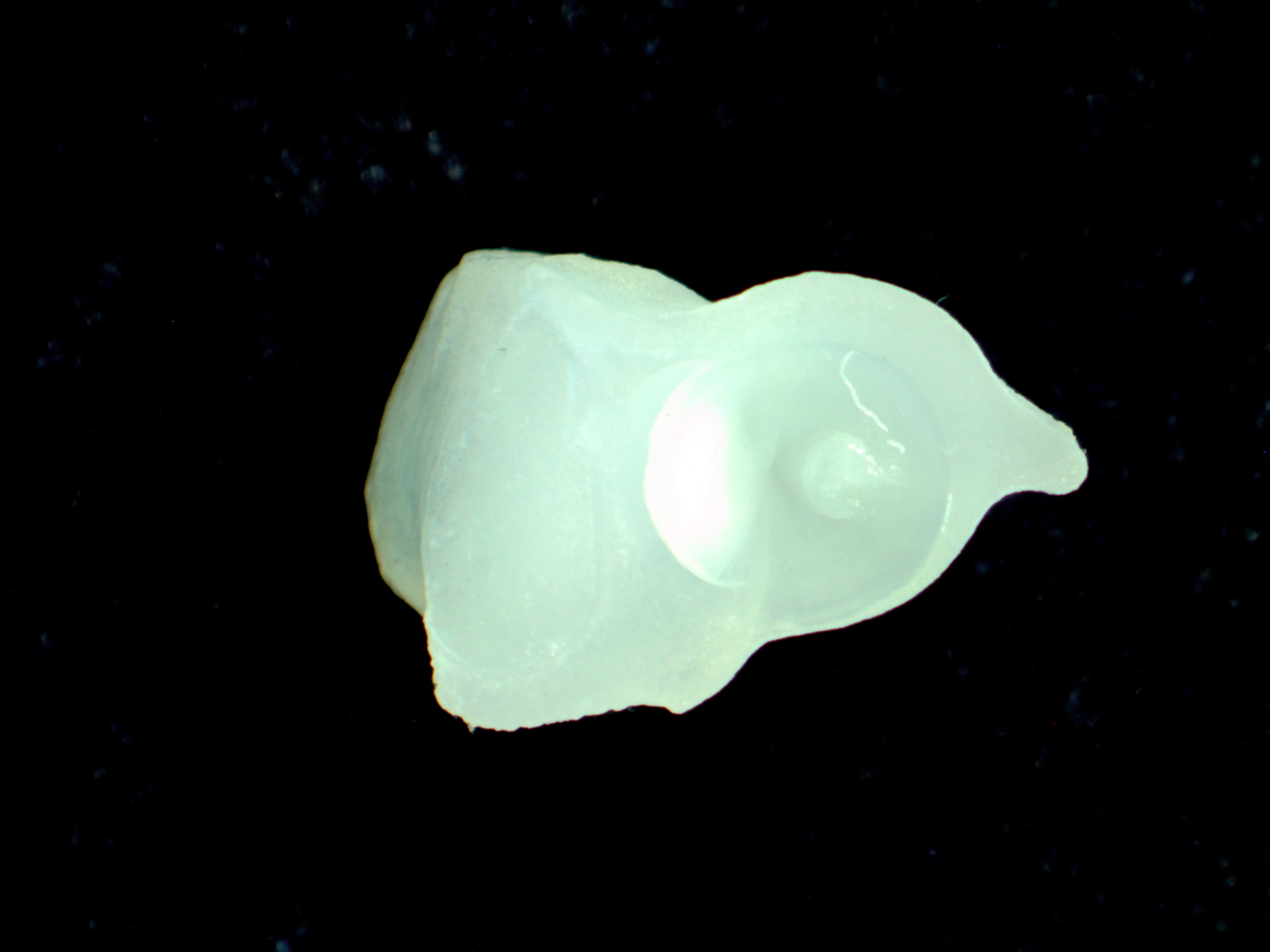

Supplement: Supplemental Information 12 [file peerj-04-1664-s012.zip › JohBel/training/S74R1.jpg]

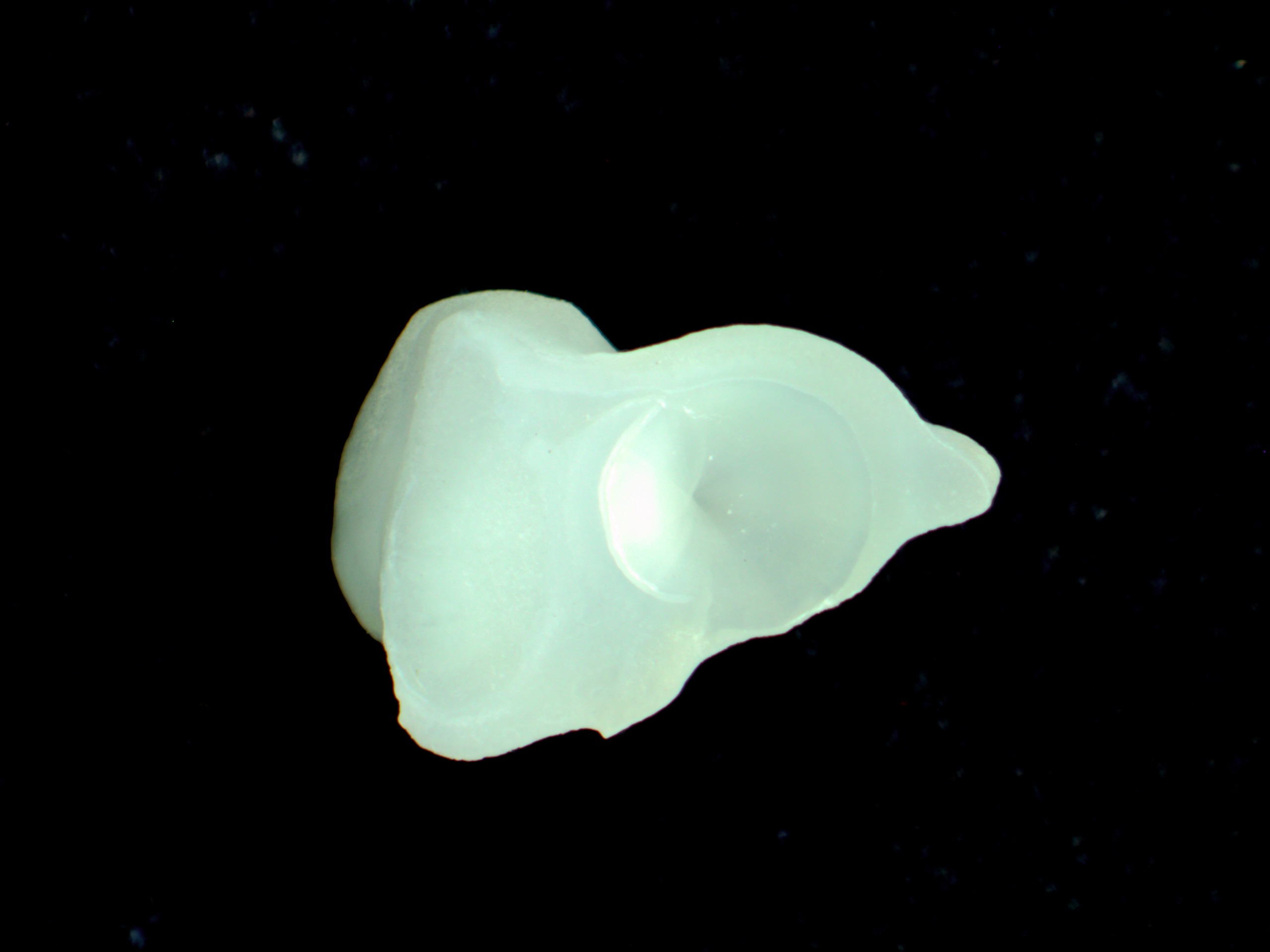

Supplement: Supplemental Information 12 [file peerj-04-1664-s012.zip › JohBel/training/S75R1.jpg]

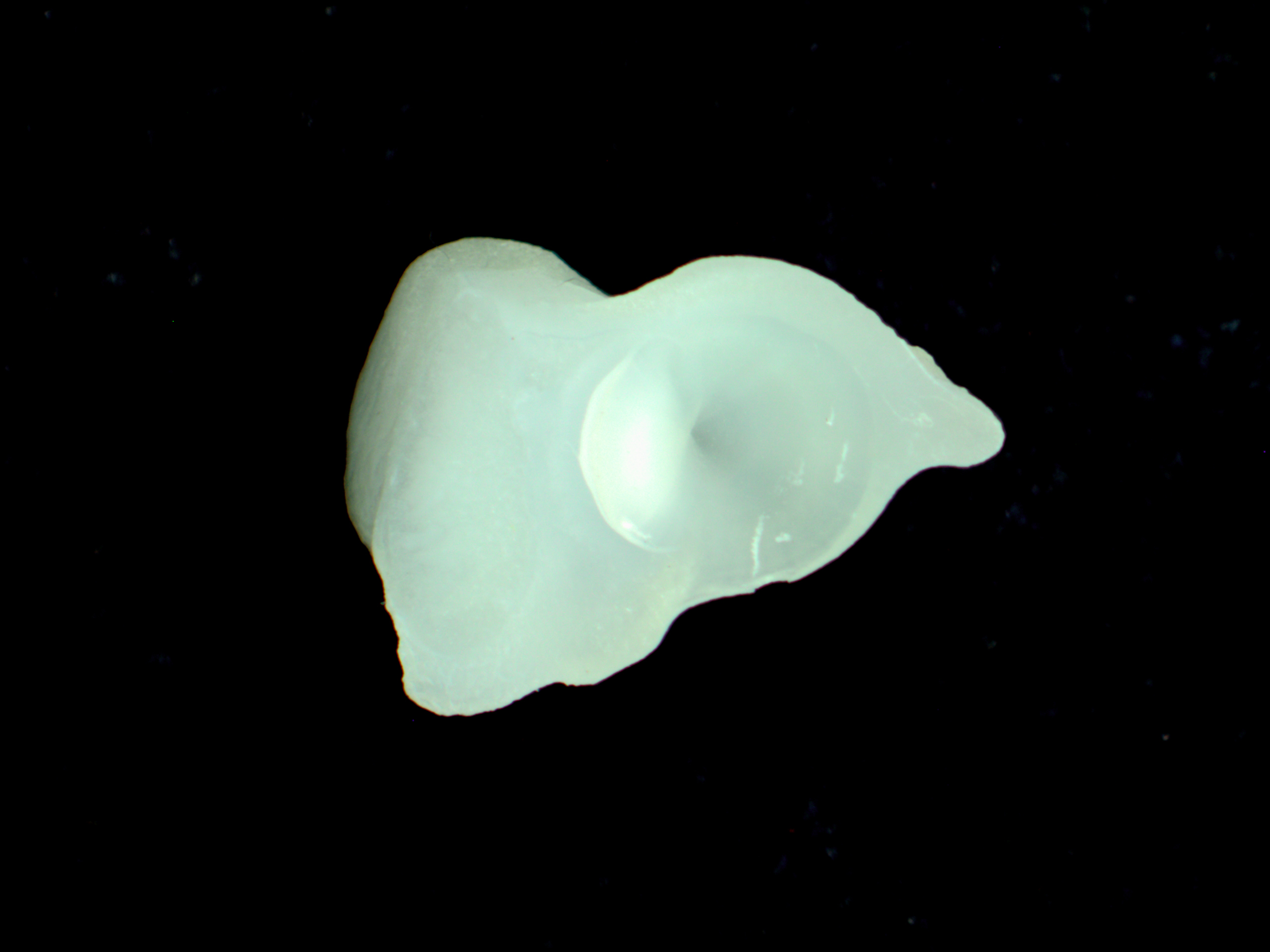

Supplement: Supplemental Information 12 [file peerj-04-1664-s012.zip › JohBel/training/S76R1.jpg]

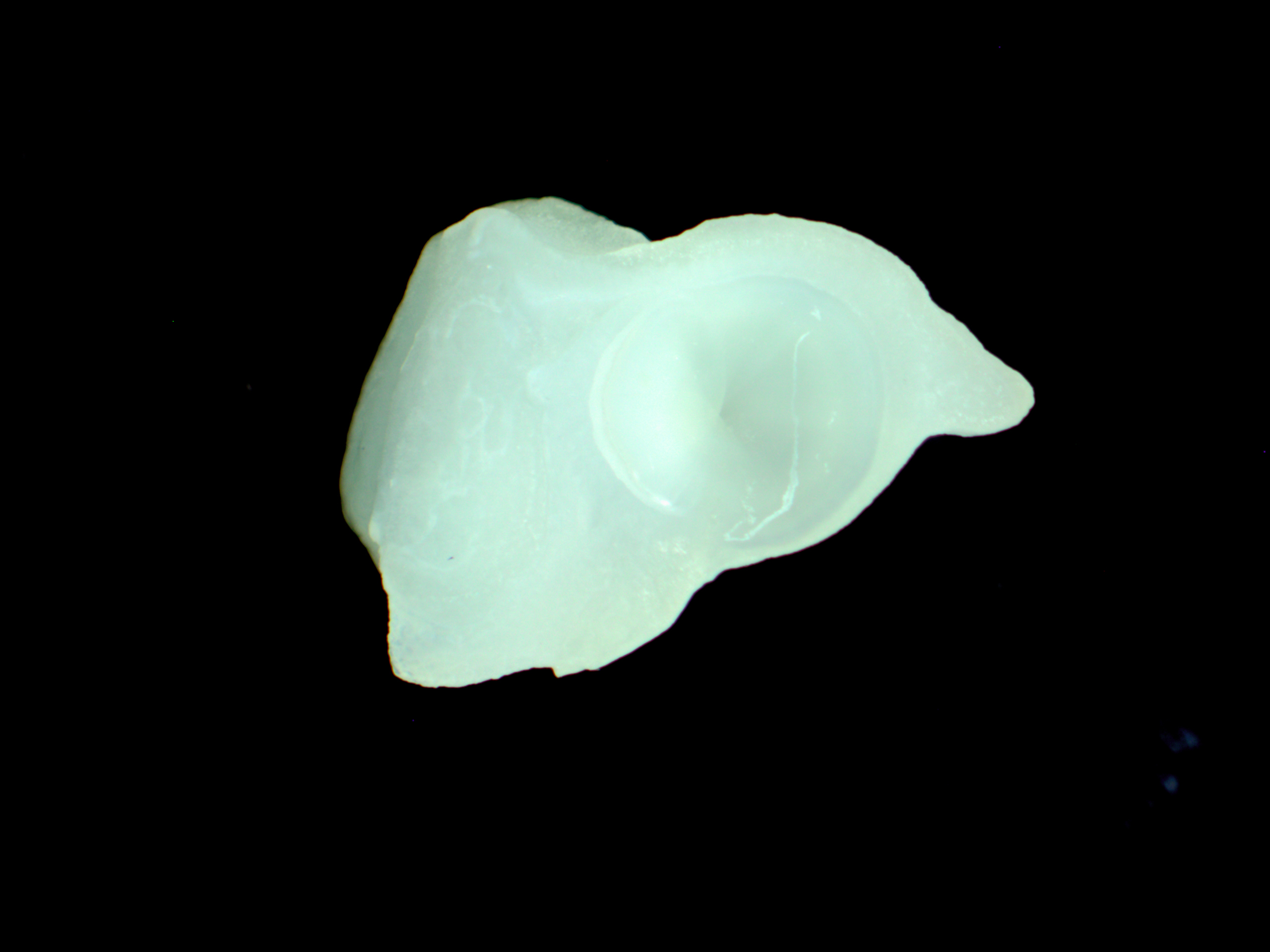

Supplement: Supplemental Information 12 [file peerj-04-1664-s012.zip › JohBel/training/S7R1.jpg]

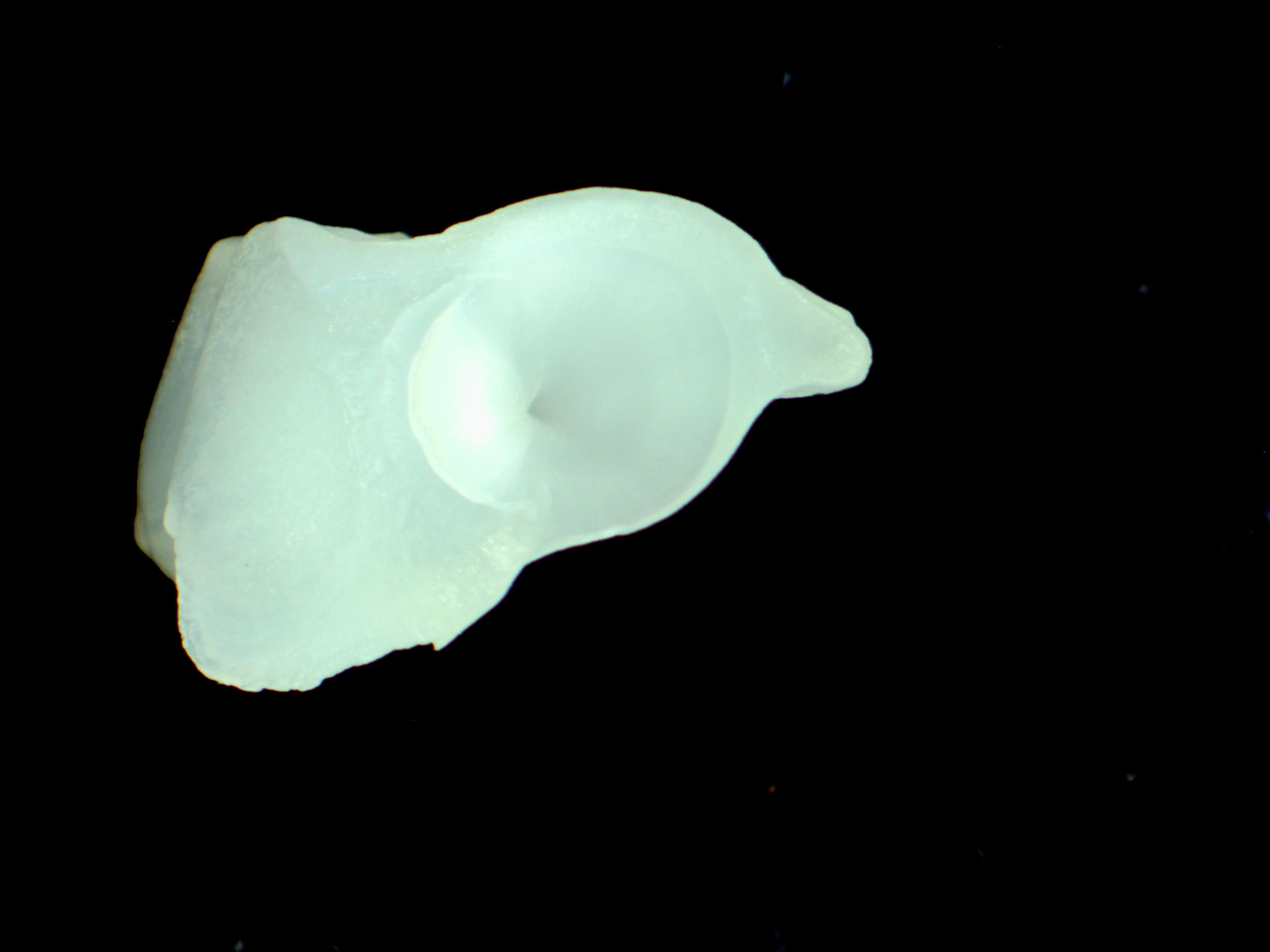

Supplement: Supplemental Information 12 [file peerj-04-1664-s012.zip › JohBel/training/S8R1.jpg]

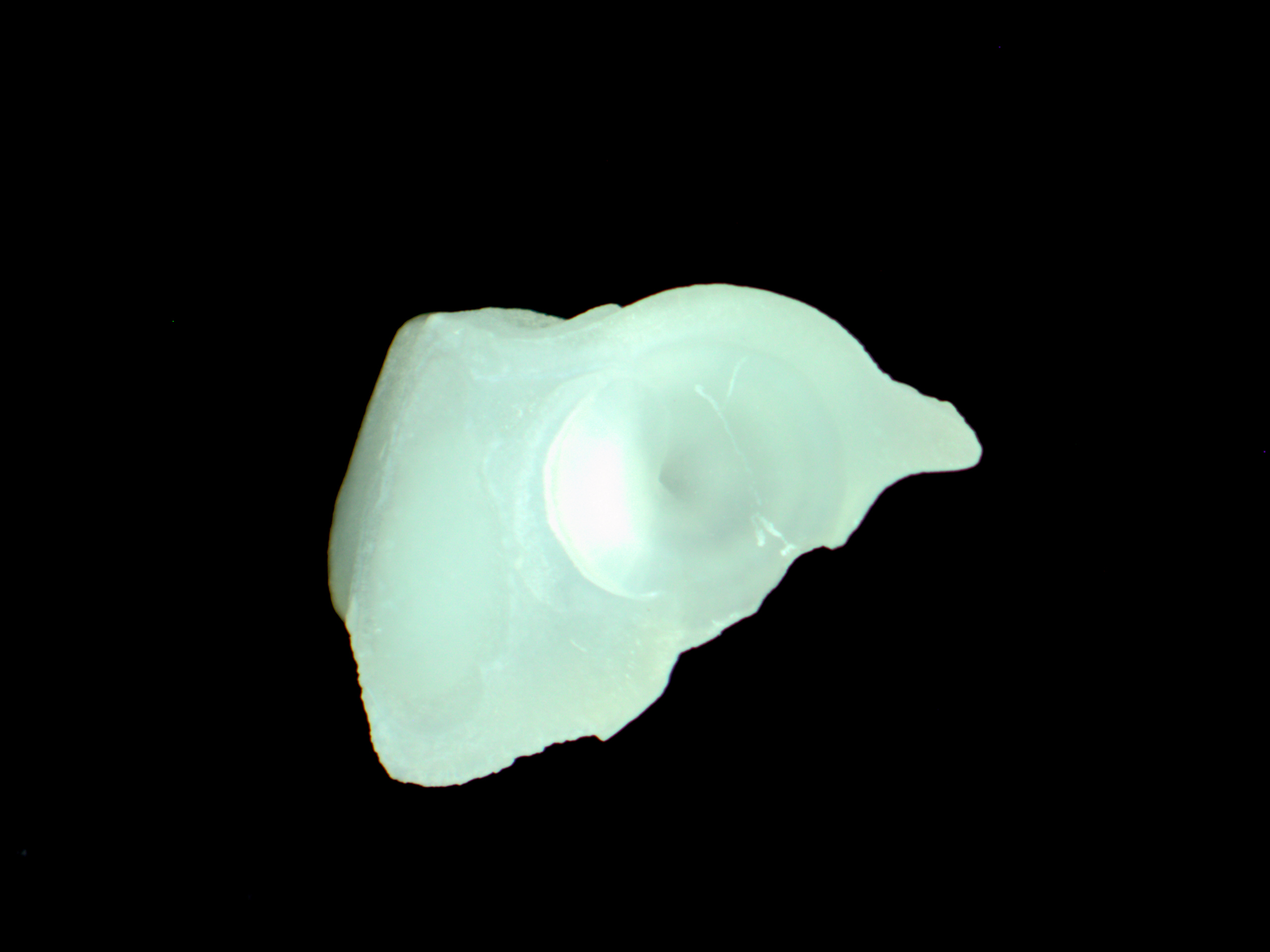

Supplement: Supplemental Information 12 [file peerj-04-1664-s012.zip › JohBel/training/S9R1.jpg]

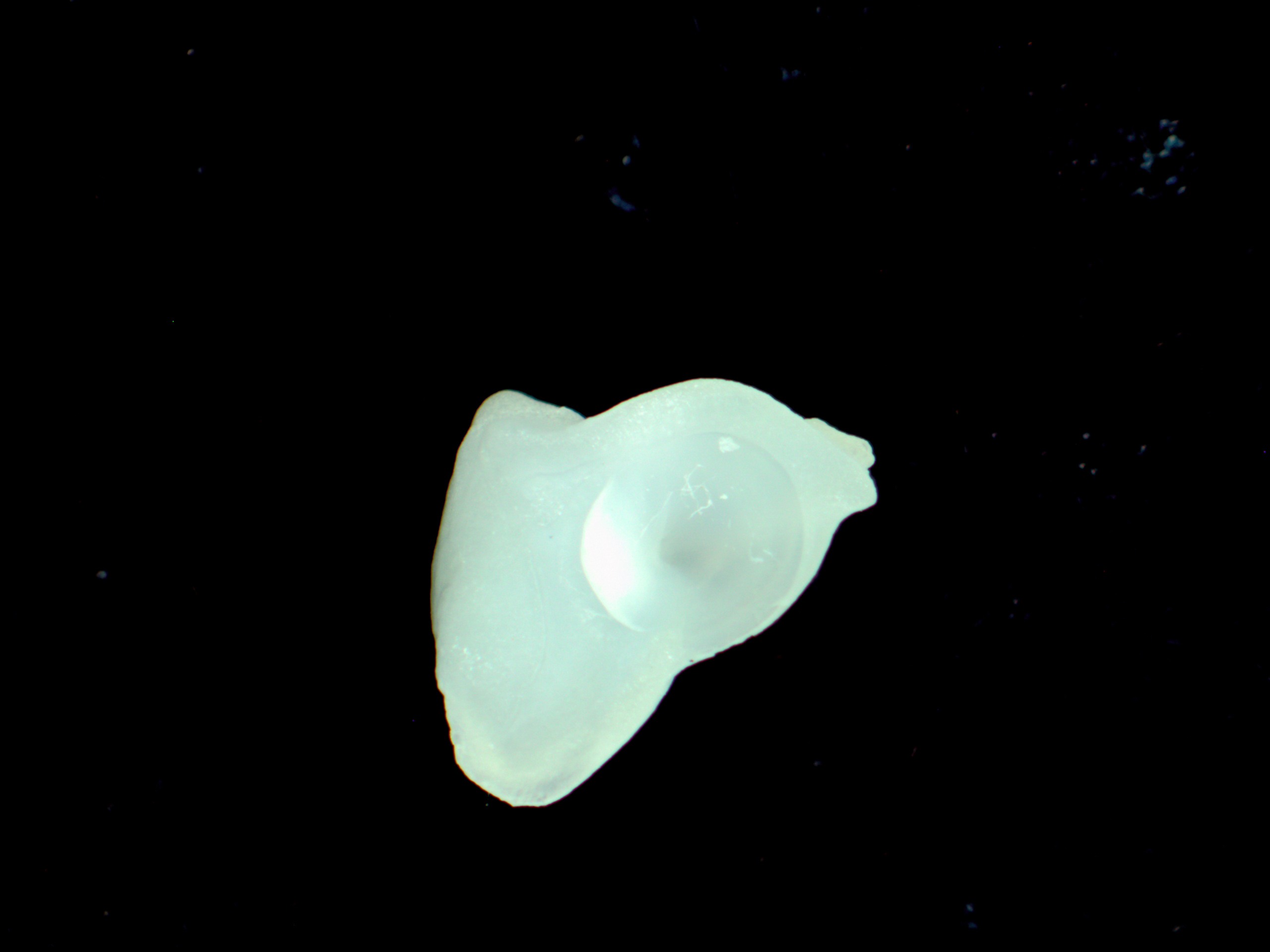

Supplement: Supplemental Information 13 [file peerj-04-1664-s013.zip › JohCar/testing/37R1.jpg]

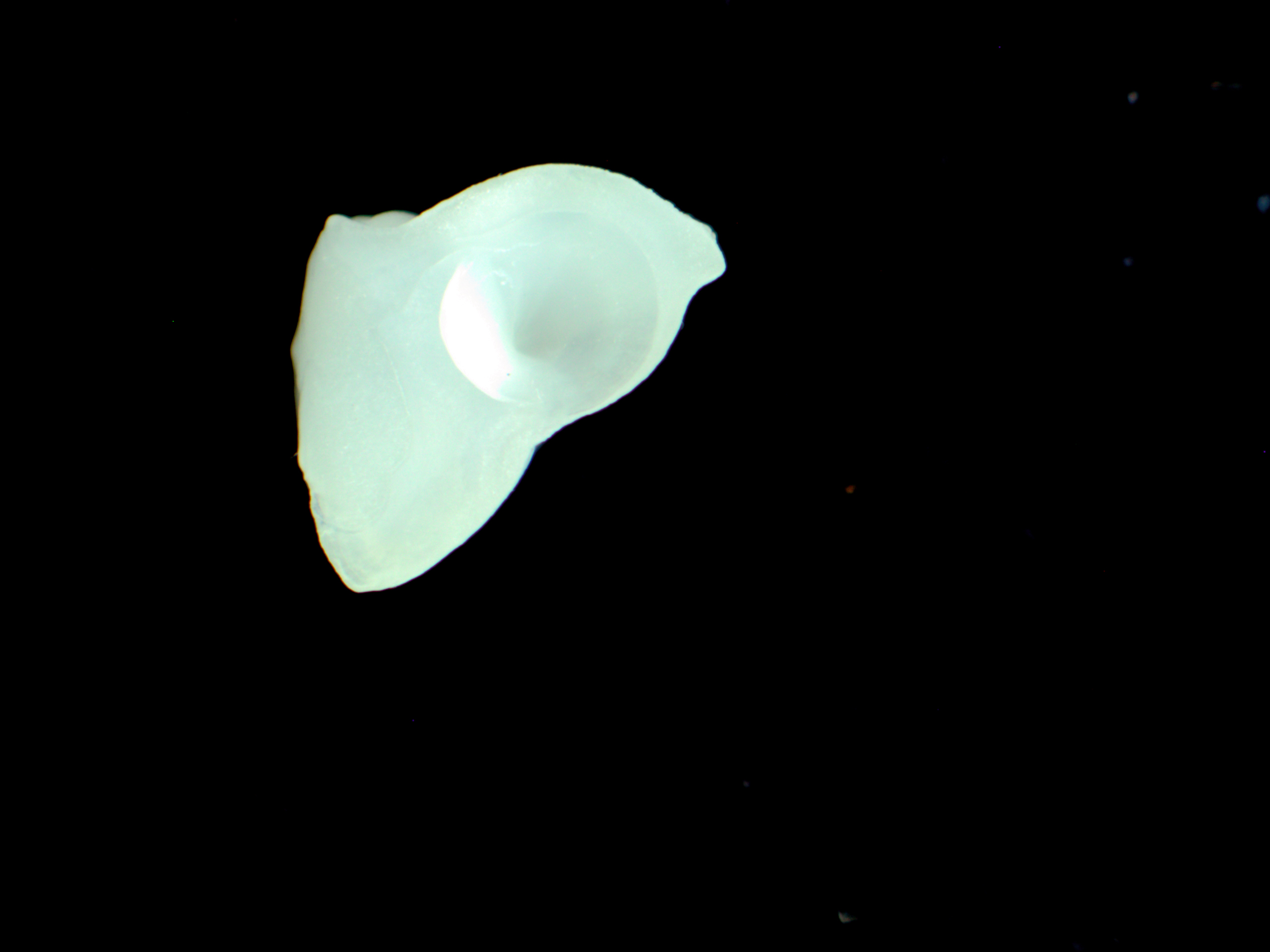

Supplement: Supplemental Information 13 [file peerj-04-1664-s013.zip › JohCar/testing/38R1.jpg]

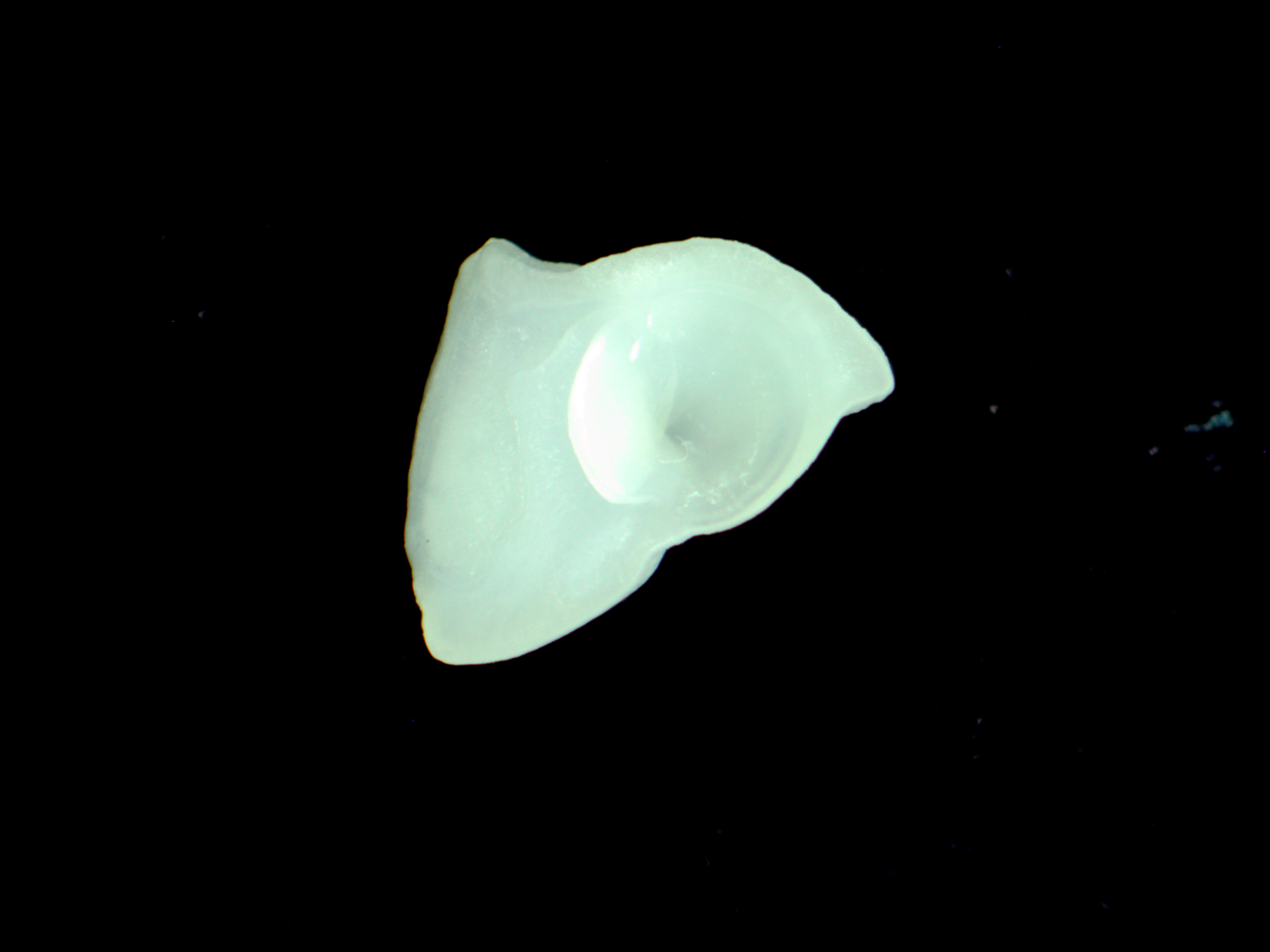

Supplement: Supplemental Information 13 [file peerj-04-1664-s013.zip › JohCar/testing/39R1.jpg]

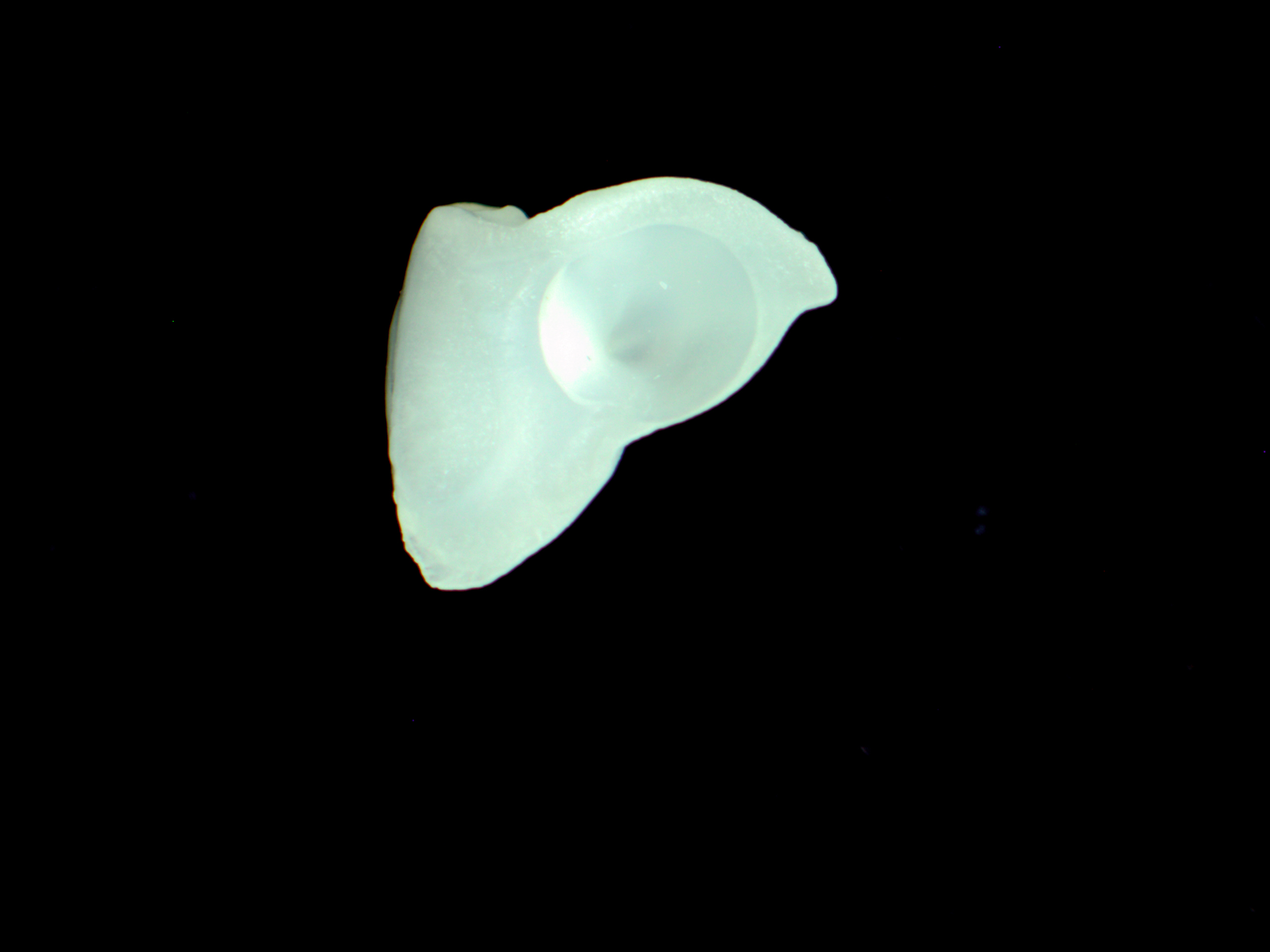

Supplement: Supplemental Information 13 [file peerj-04-1664-s013.zip › JohCar/testing/40R1.jpg]

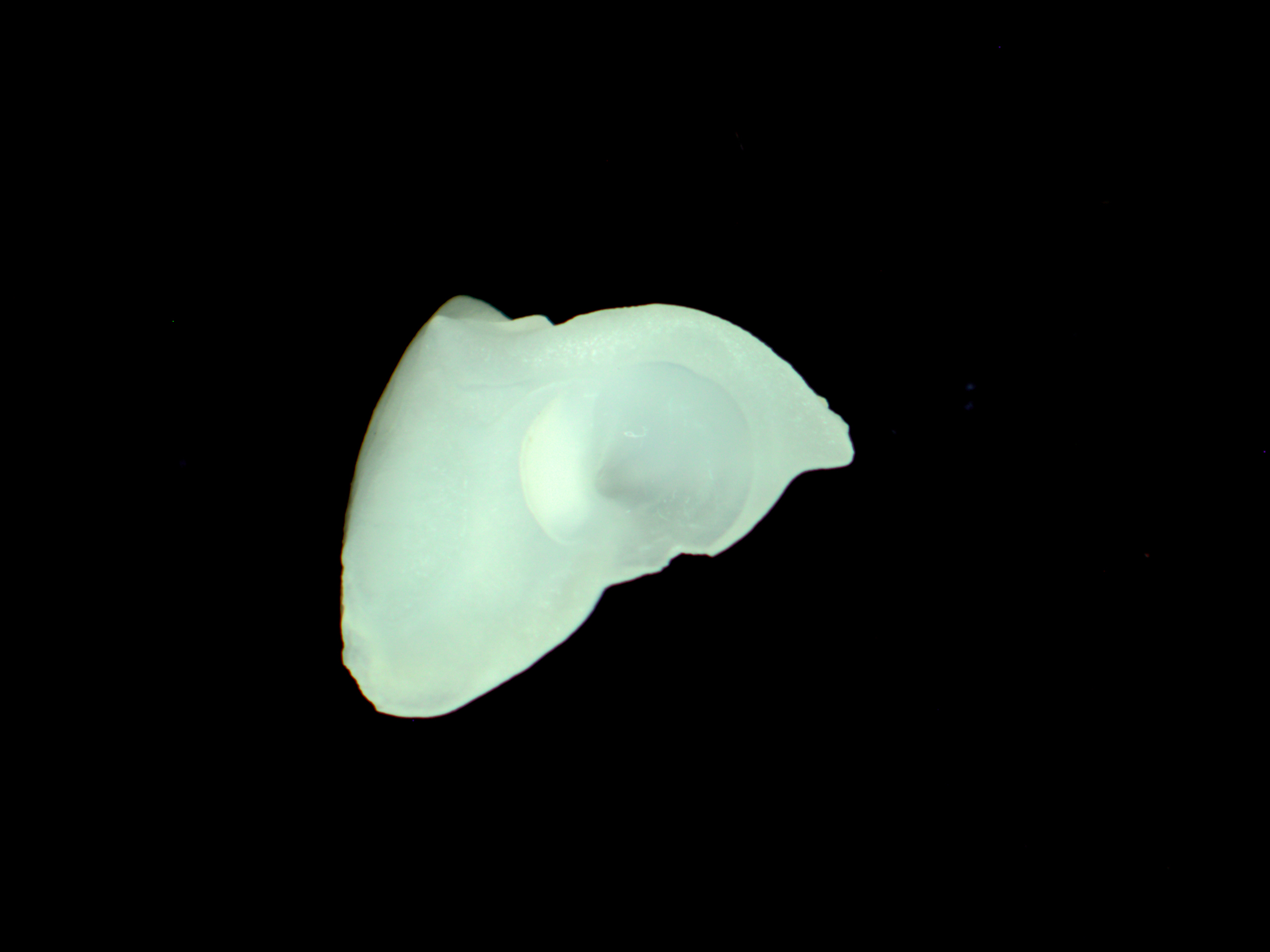

Supplement: Supplemental Information 13 [file peerj-04-1664-s013.zip › JohCar/testing/41R1.jpg]

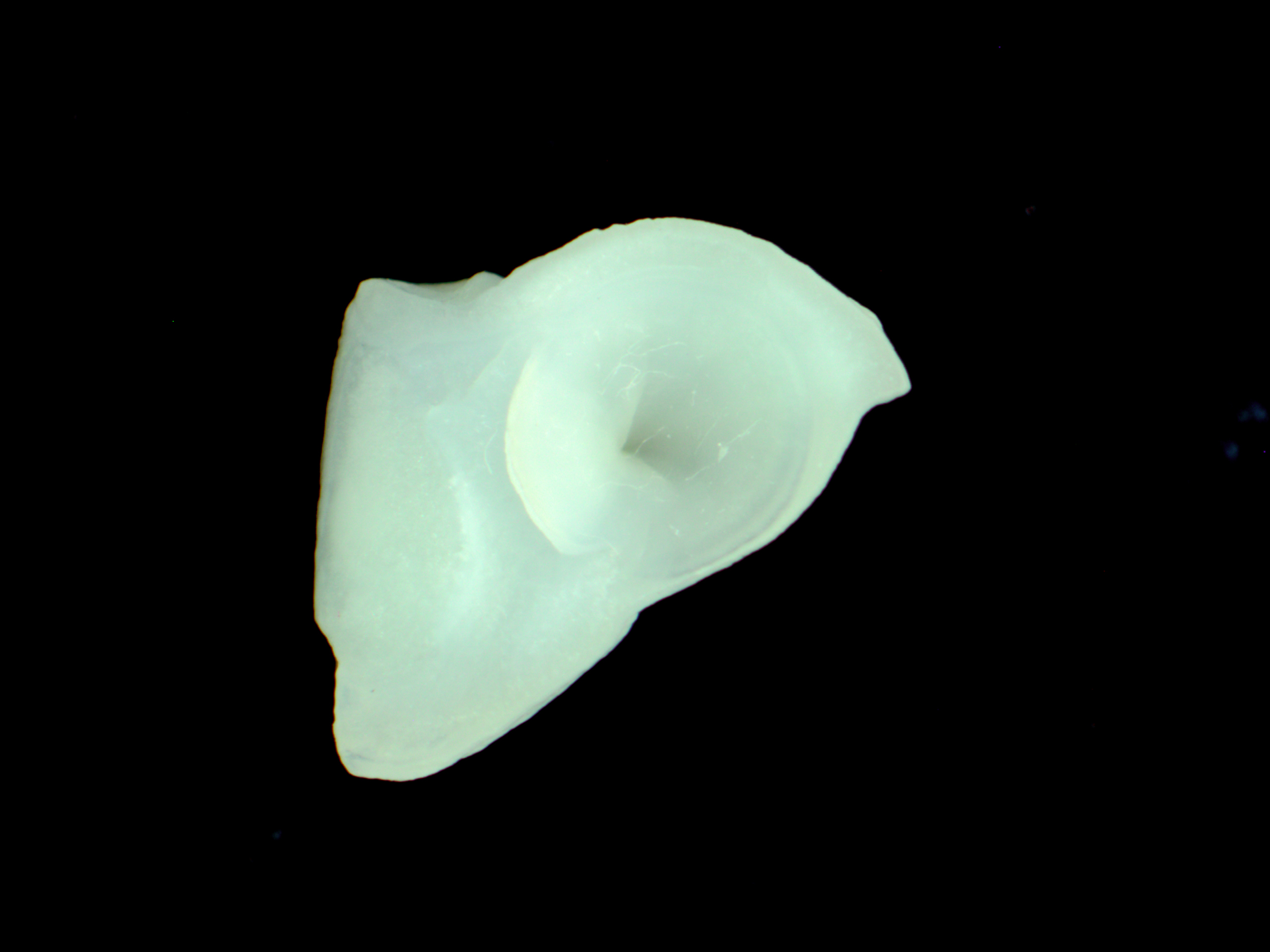

Supplement: Supplemental Information 13 [file peerj-04-1664-s013.zip › JohCar/testing/42R1.jpg]

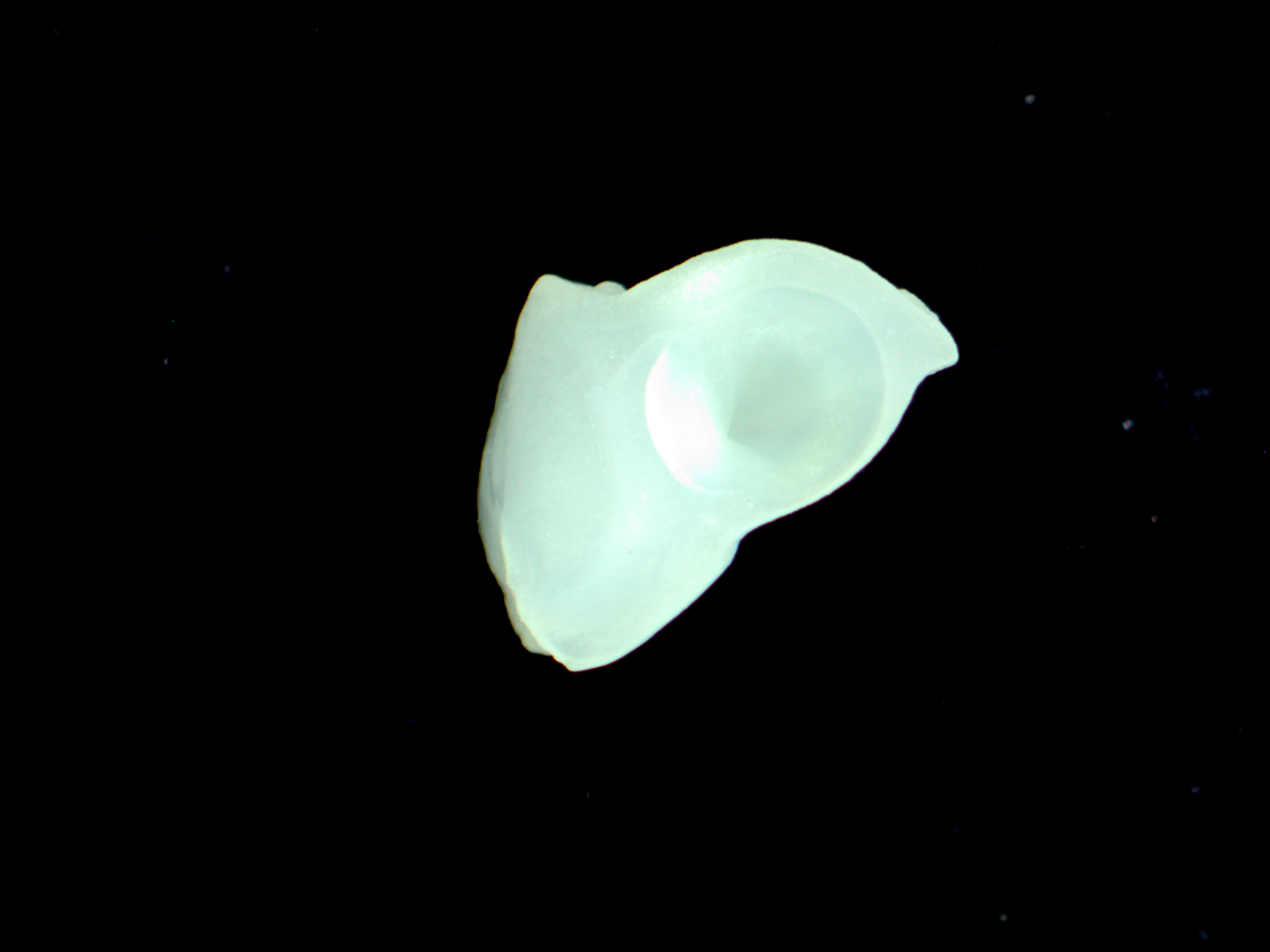

Supplement: Supplemental Information 13 [file peerj-04-1664-s013.zip › JohCar/testing/43R1.jpg]

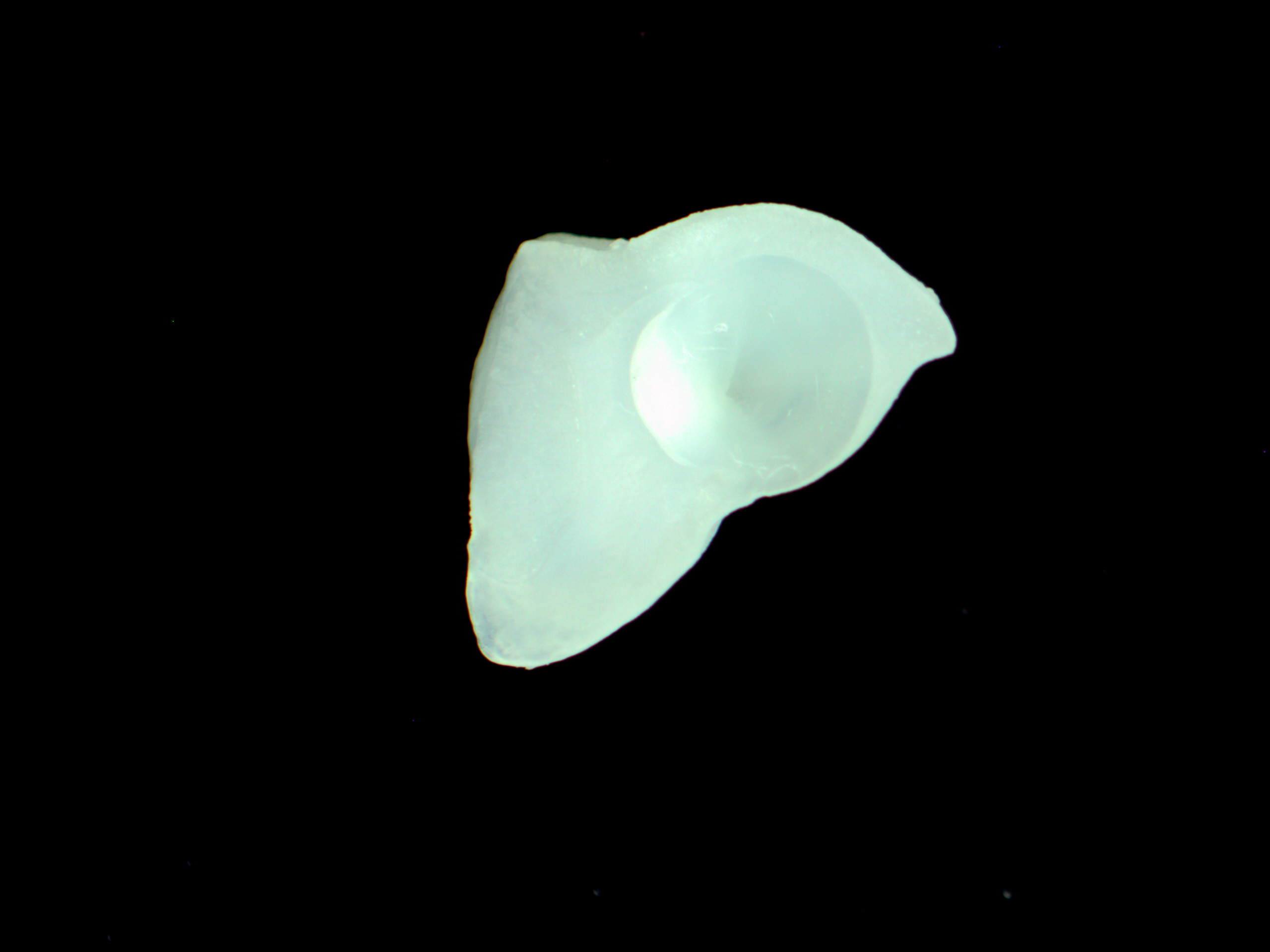

Supplement: Supplemental Information 13 [file peerj-04-1664-s013.zip › JohCar/testing/44R1.jpg]

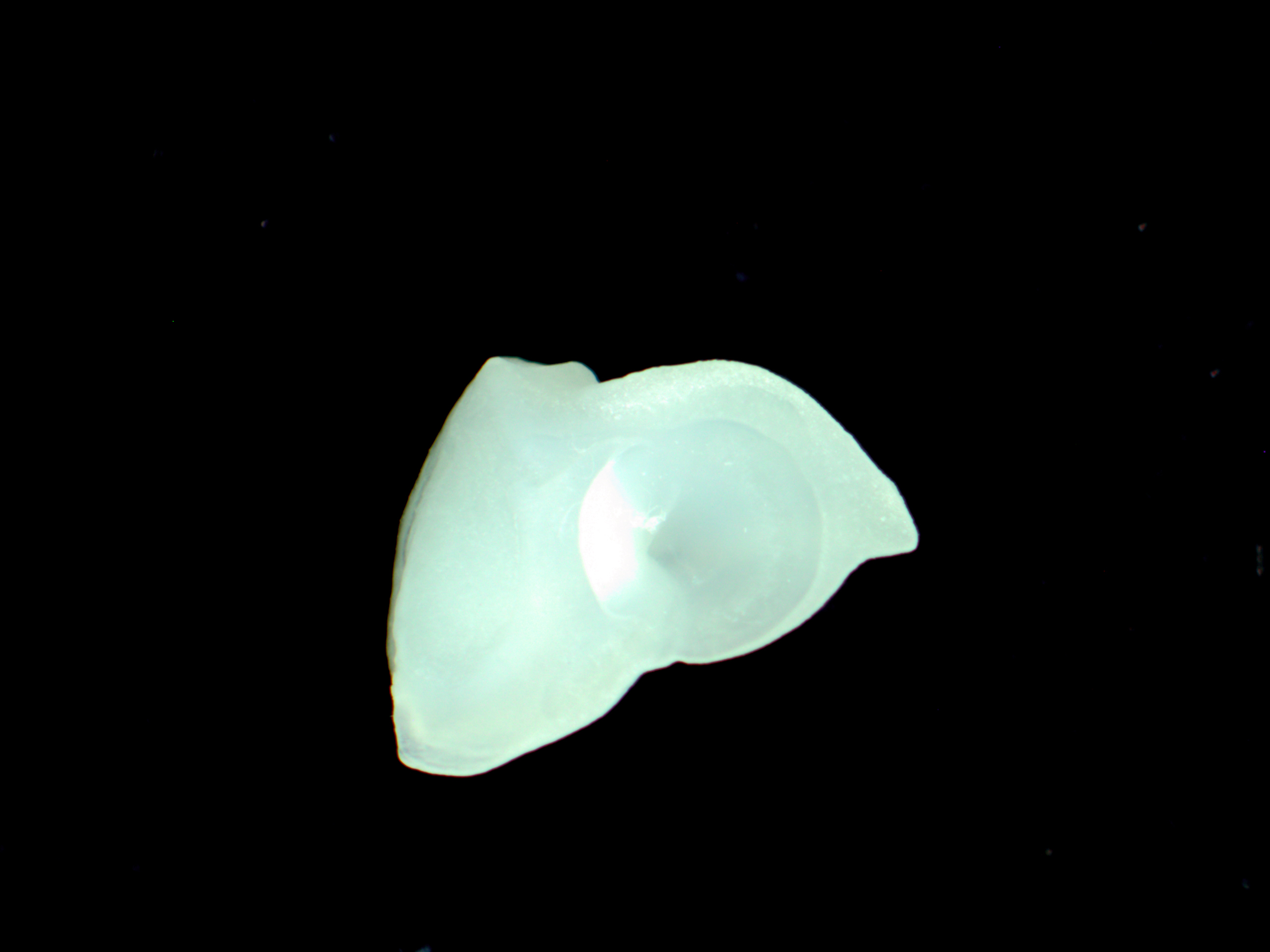

Supplement: Supplemental Information 13 [file peerj-04-1664-s013.zip › JohCar/testing/46R1.jpg]

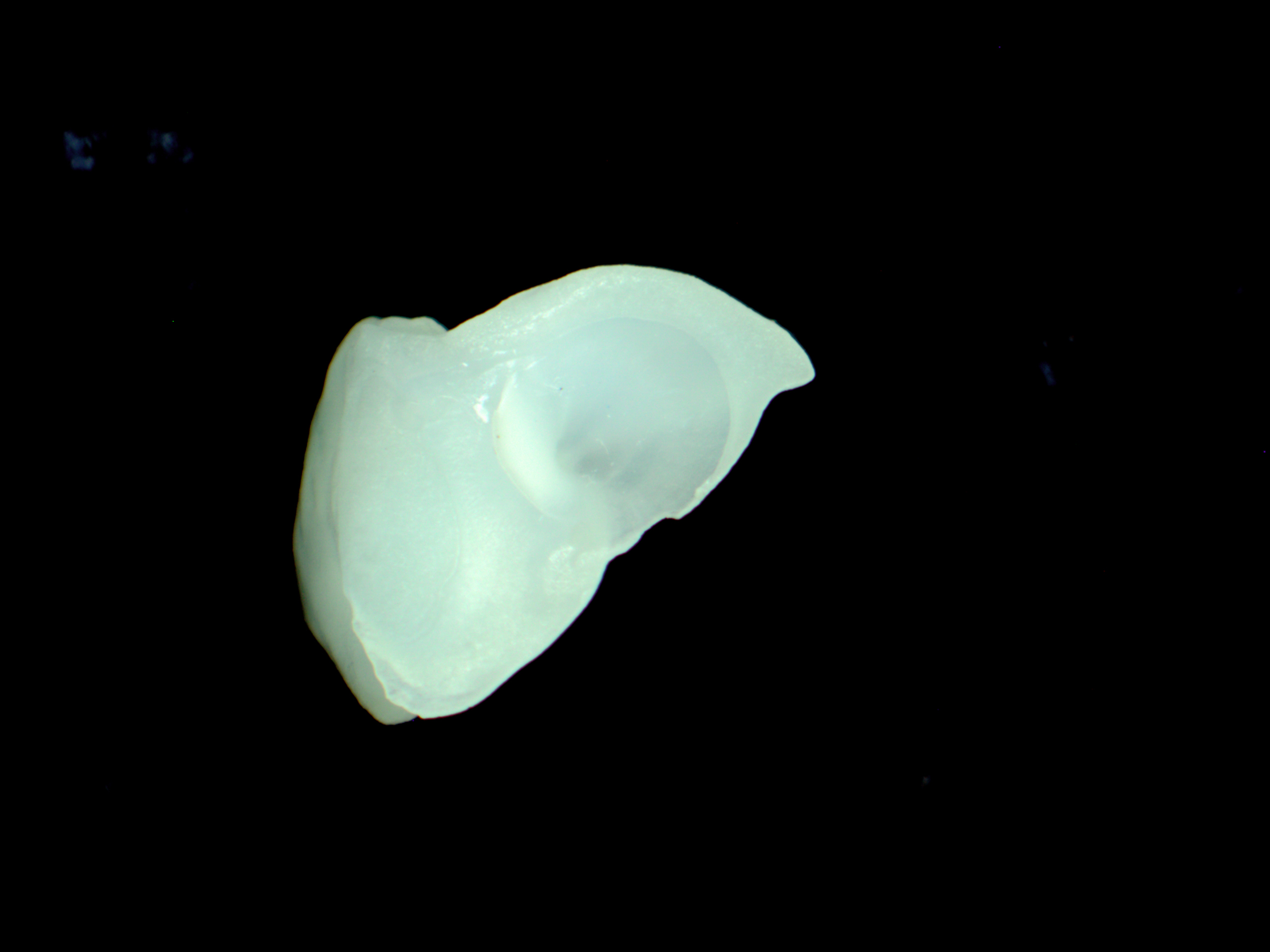

Supplement: Supplemental Information 13 [file peerj-04-1664-s013.zip › JohCar/testing/47R1.jpg]

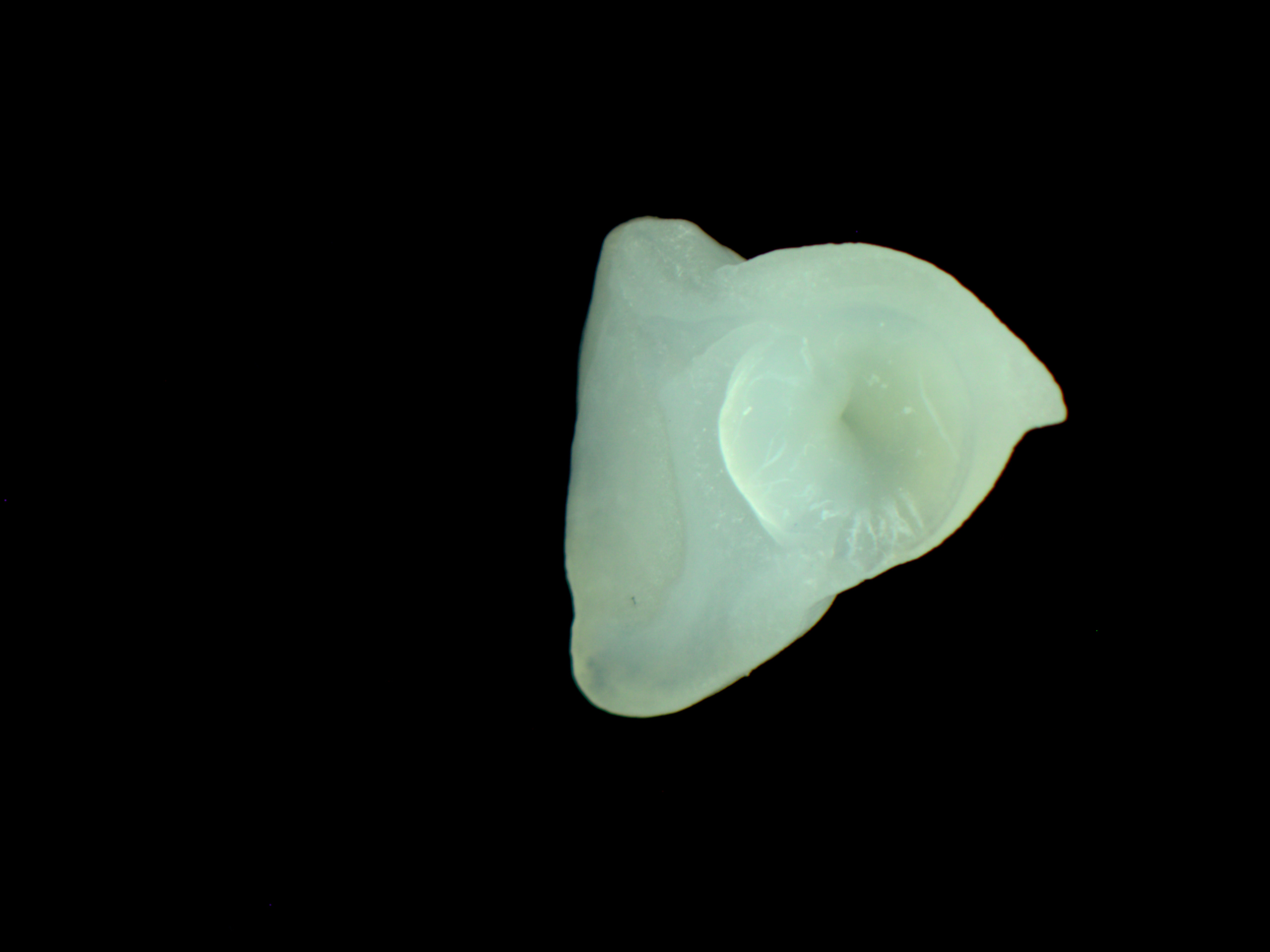

Supplement: Supplemental Information 13 [file peerj-04-1664-s013.zip › JohCar/training/16R1.jpg]

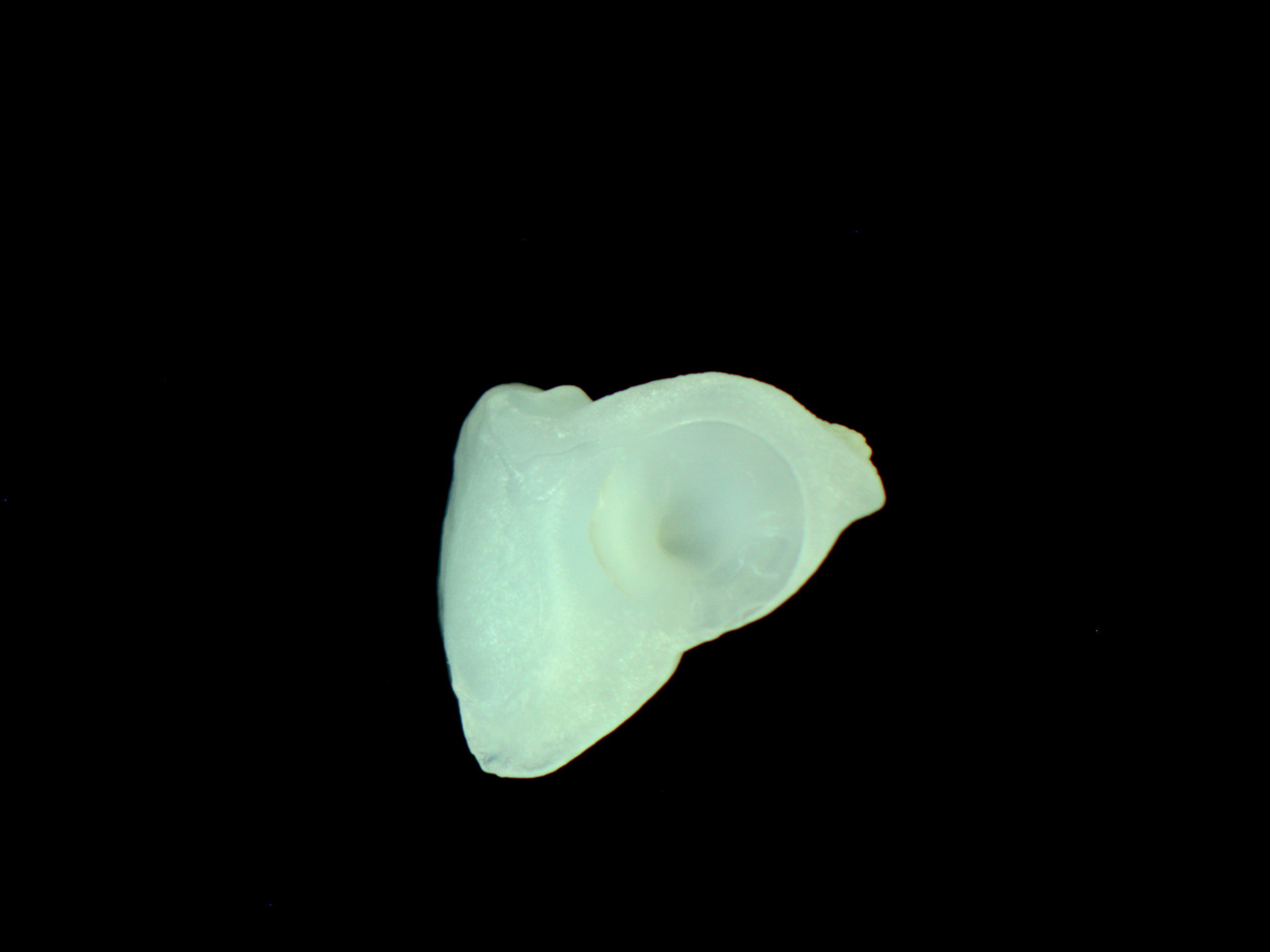

Supplement: Supplemental Information 13 [file peerj-04-1664-s013.zip › JohCar/training/17R1.jpg]

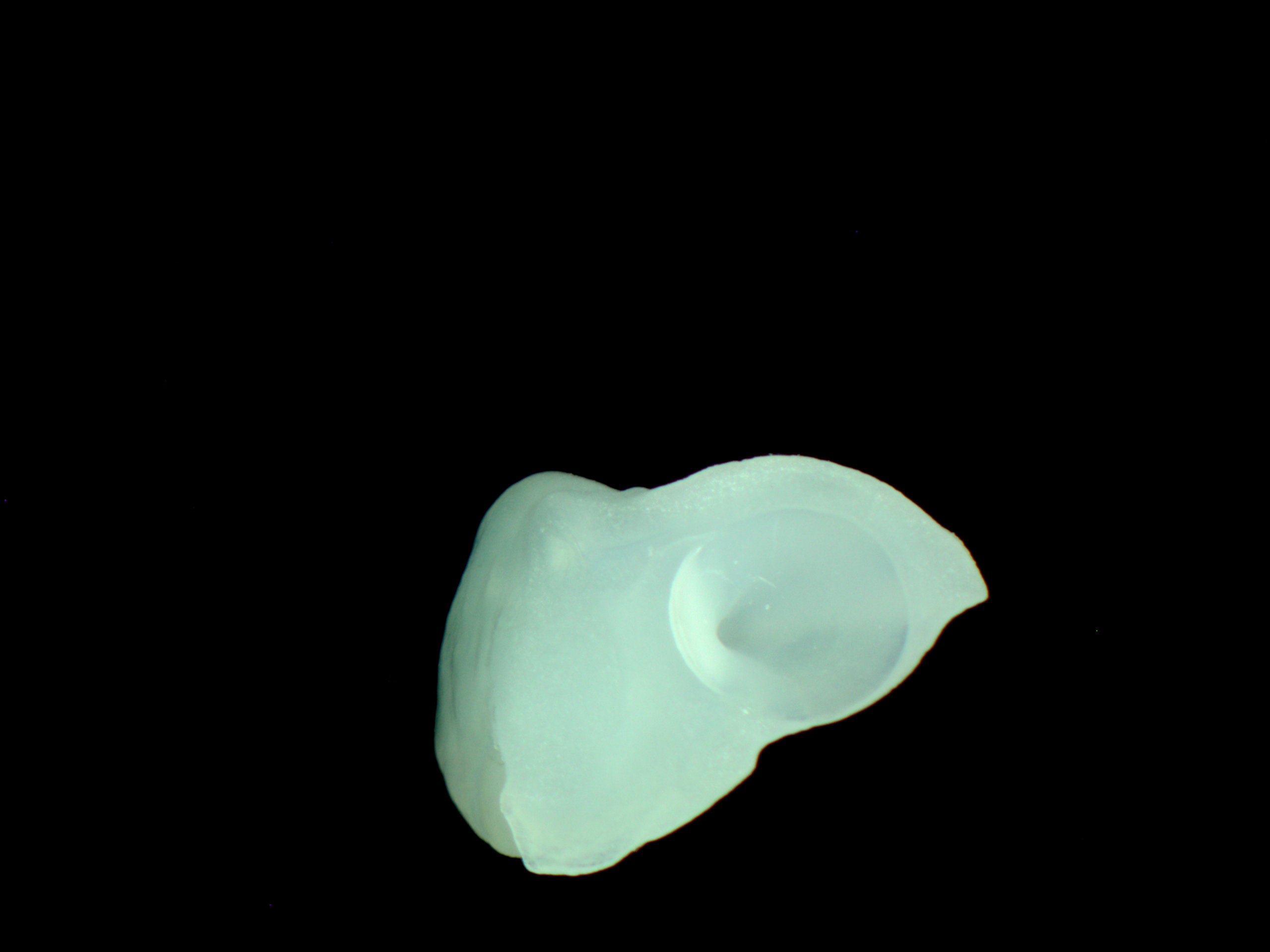

Supplement: Supplemental Information 13 [file peerj-04-1664-s013.zip › JohCar/training/18R1.jpg]

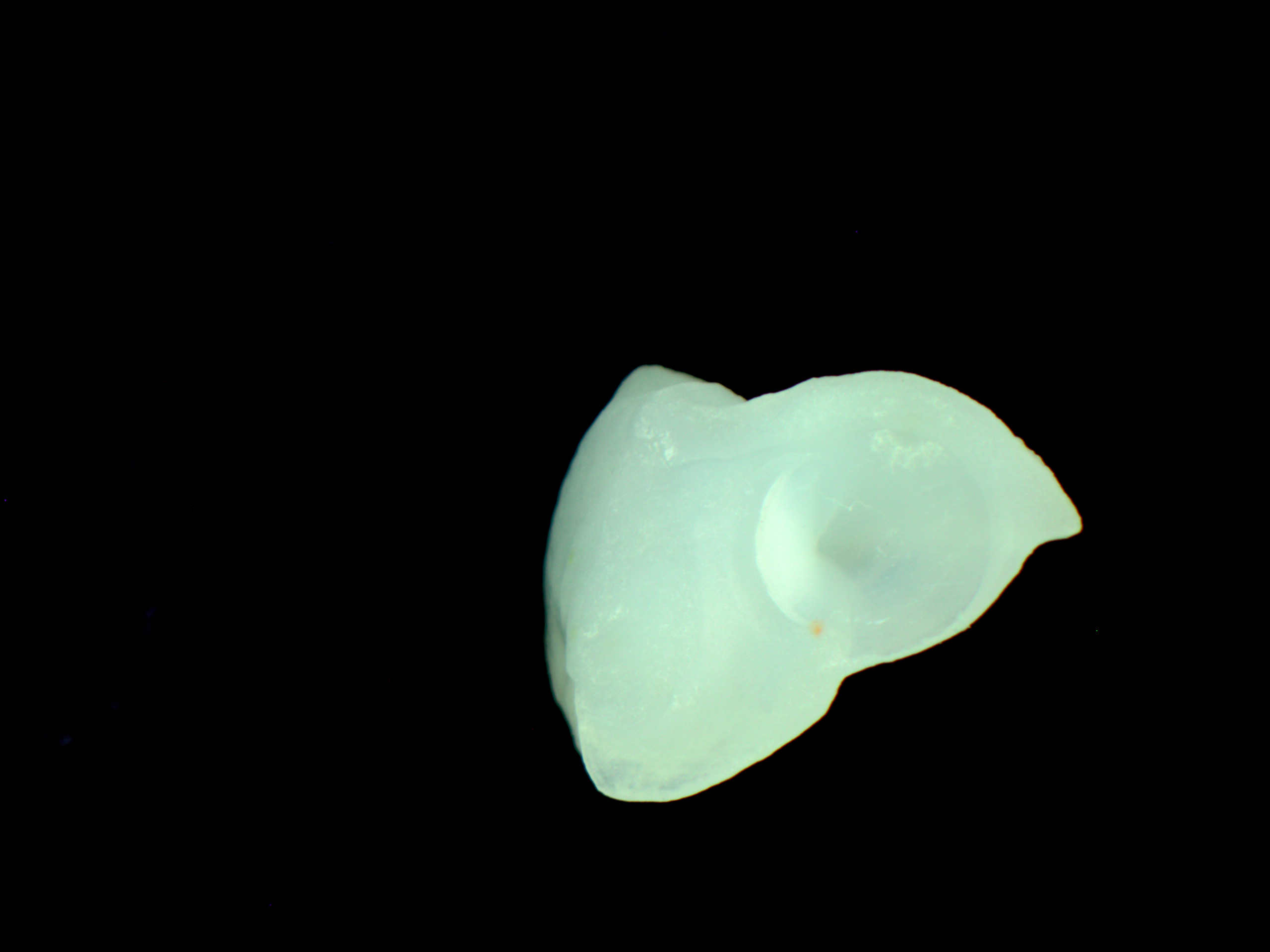

Supplement: Supplemental Information 13 [file peerj-04-1664-s013.zip › JohCar/training/19R1.jpg]

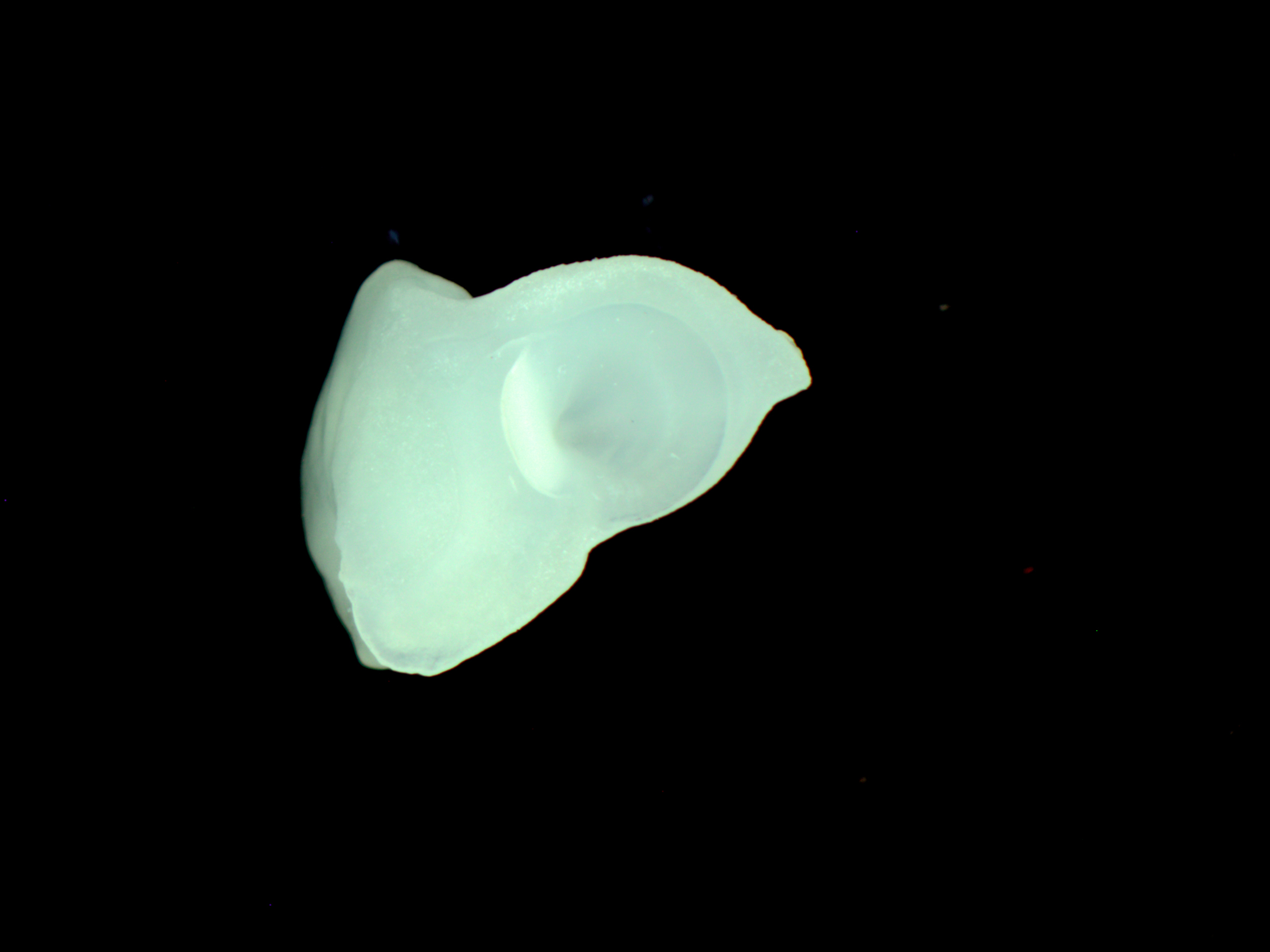

Supplement: Supplemental Information 13 [file peerj-04-1664-s013.zip › JohCar/training/20R1.jpg]

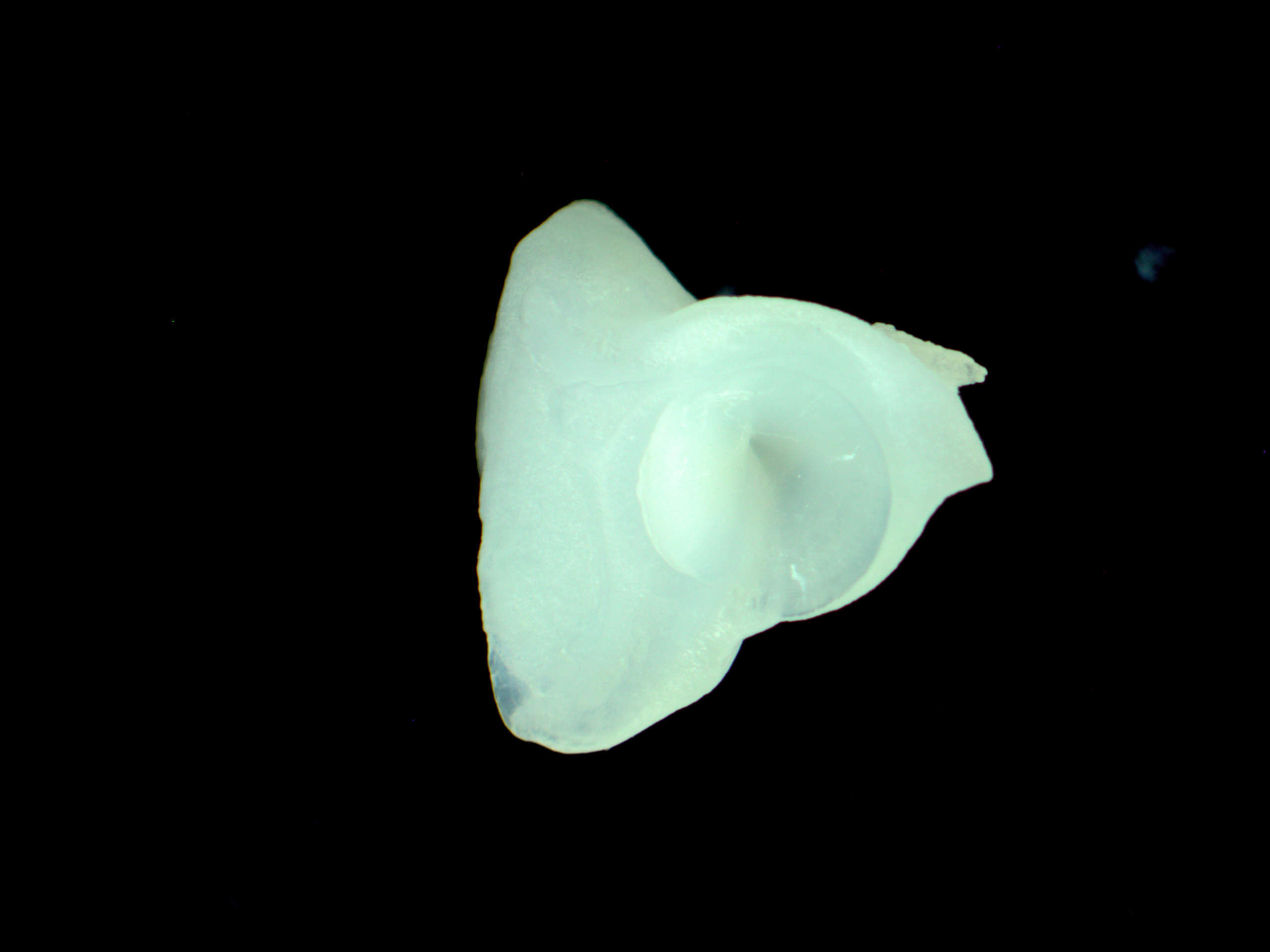

Supplement: Supplemental Information 13 [file peerj-04-1664-s013.zip › JohCar/training/21R1.jpg]

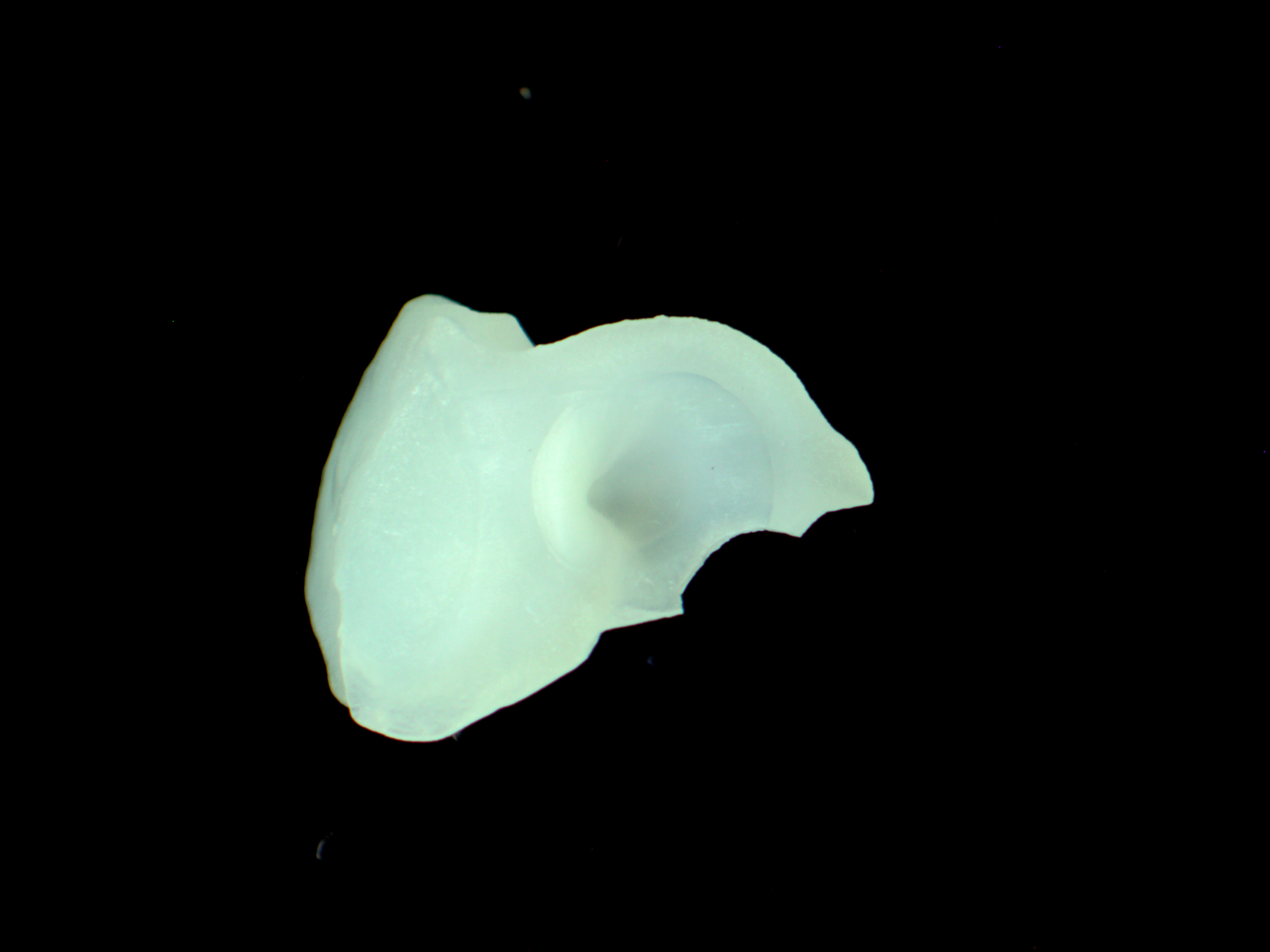

Supplement: Supplemental Information 13 [file peerj-04-1664-s013.zip › JohCar/training/22R1.jpg]

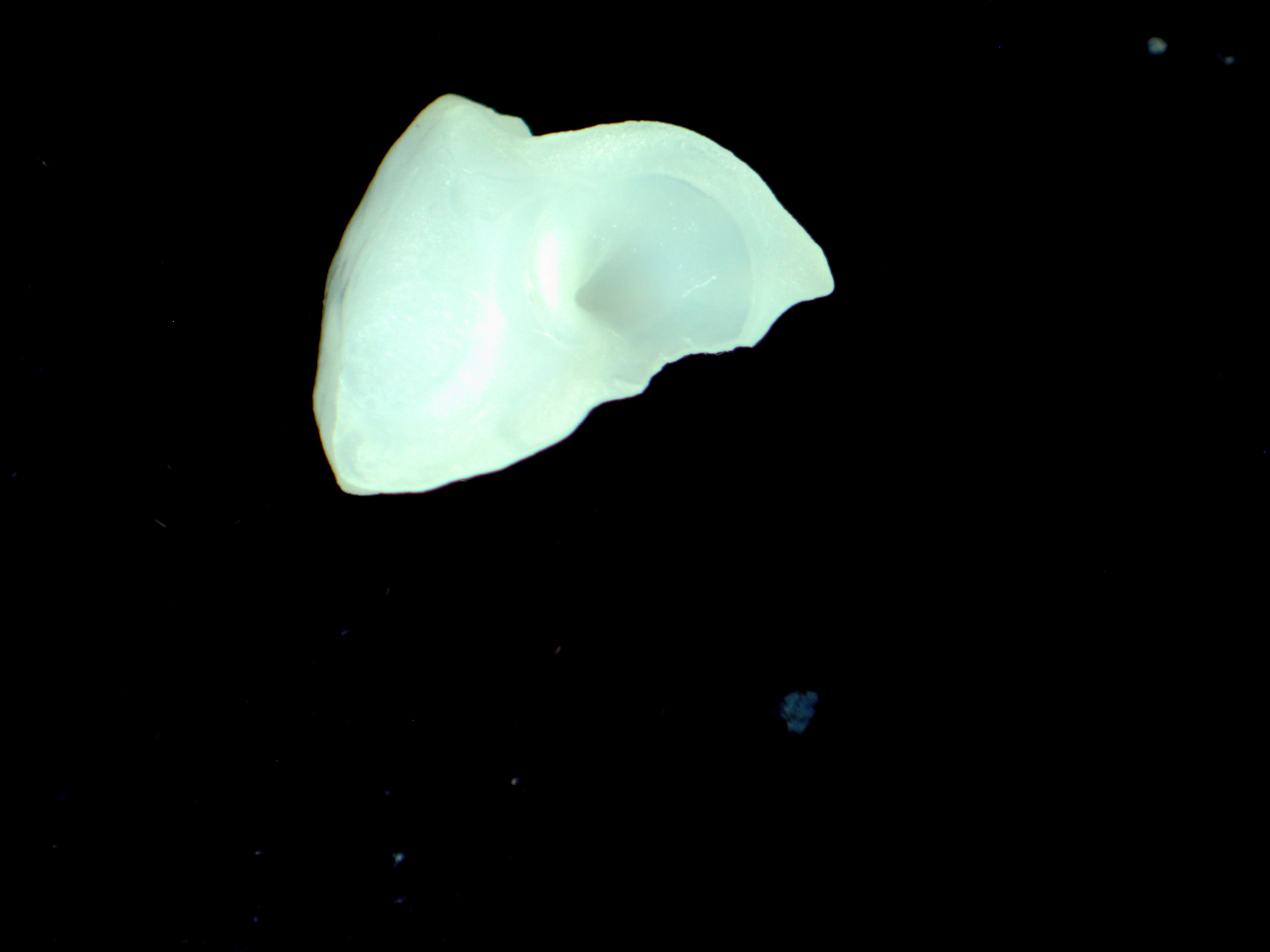

Supplement: Supplemental Information 13 [file peerj-04-1664-s013.zip › JohCar/training/23R1.jpg]

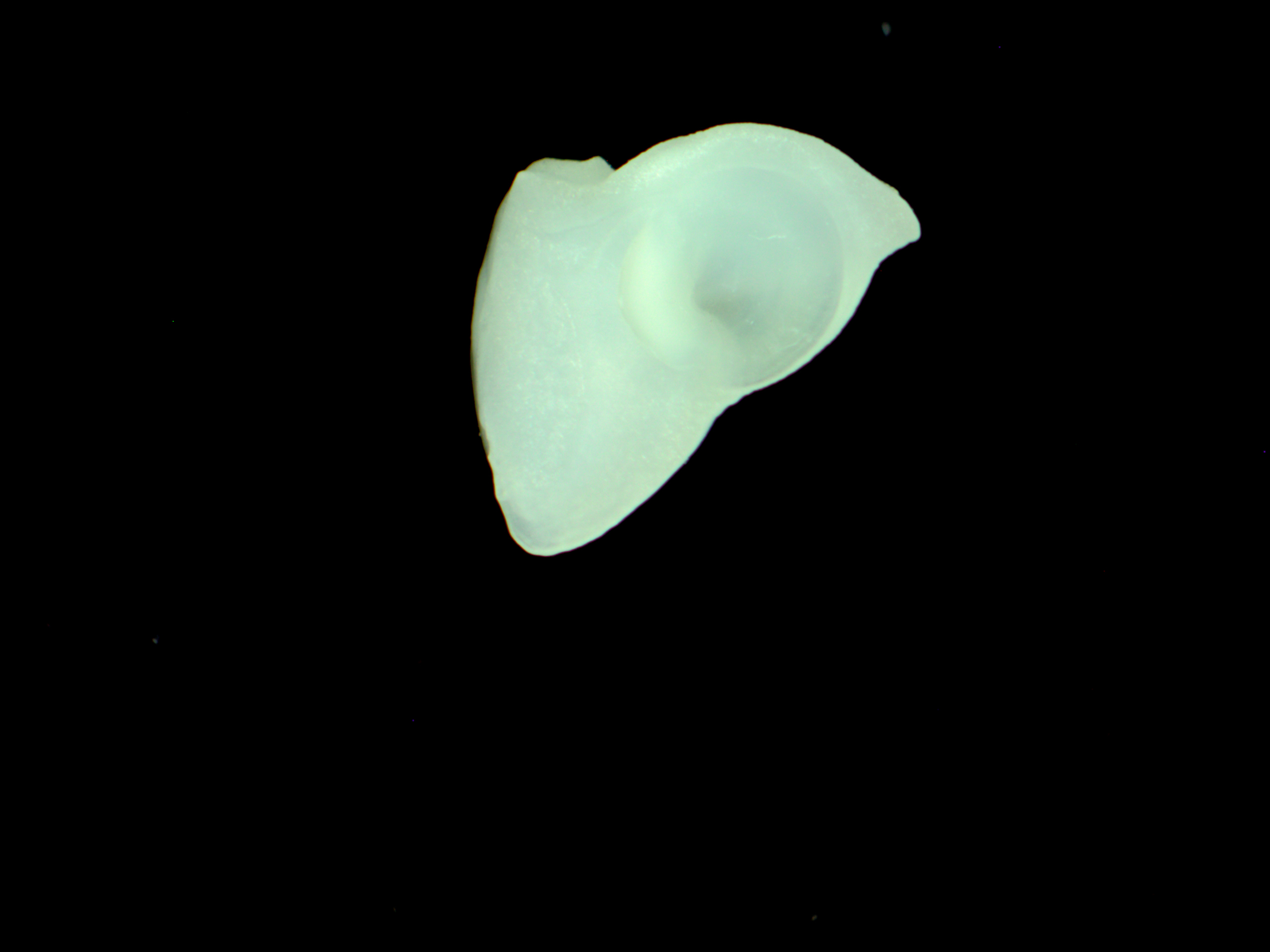

Supplement: Supplemental Information 13 [file peerj-04-1664-s013.zip › JohCar/training/24R1.jpg]

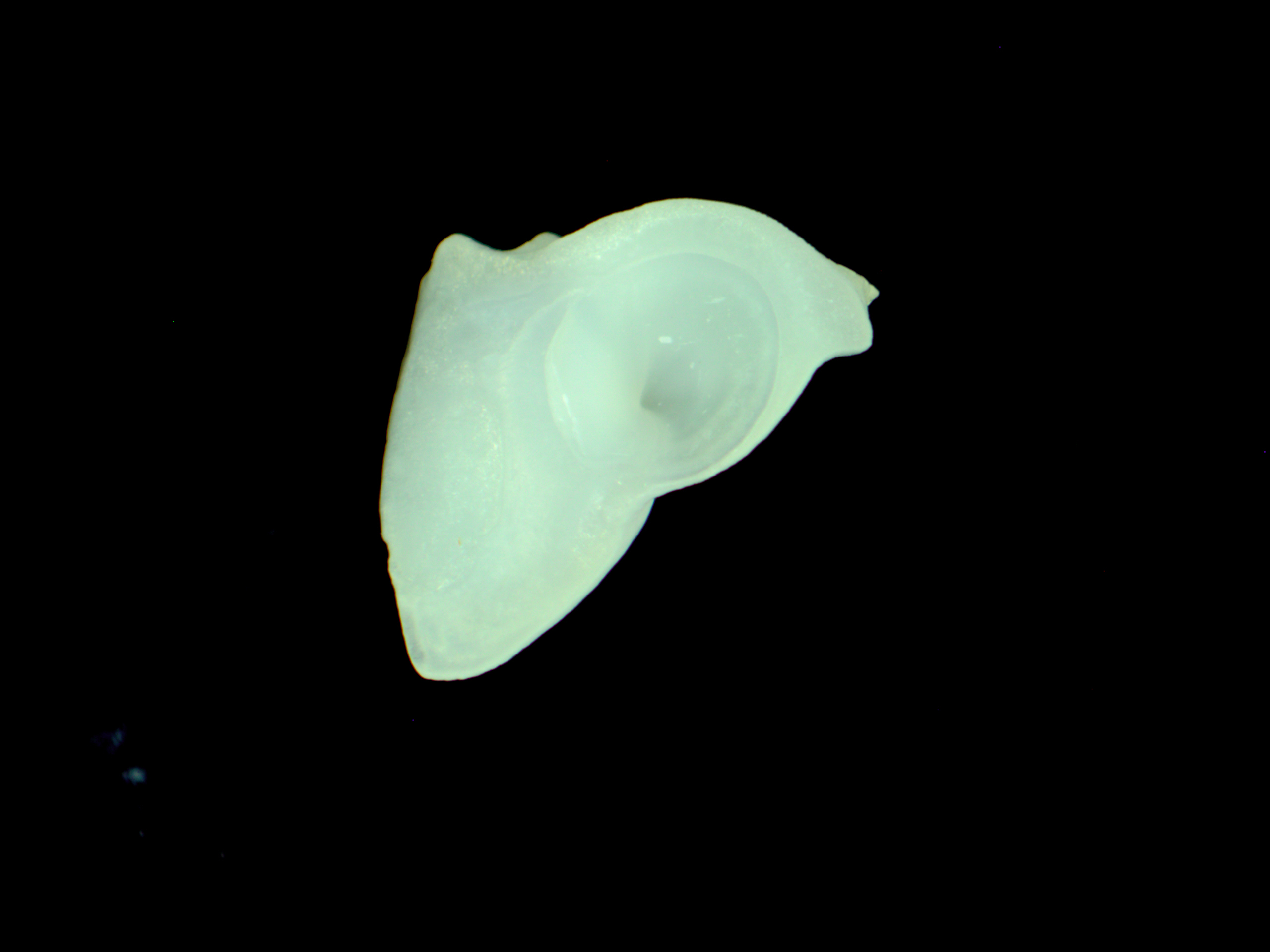

Supplement: Supplemental Information 13 [file peerj-04-1664-s013.zip › JohCar/training/25R1.jpg]

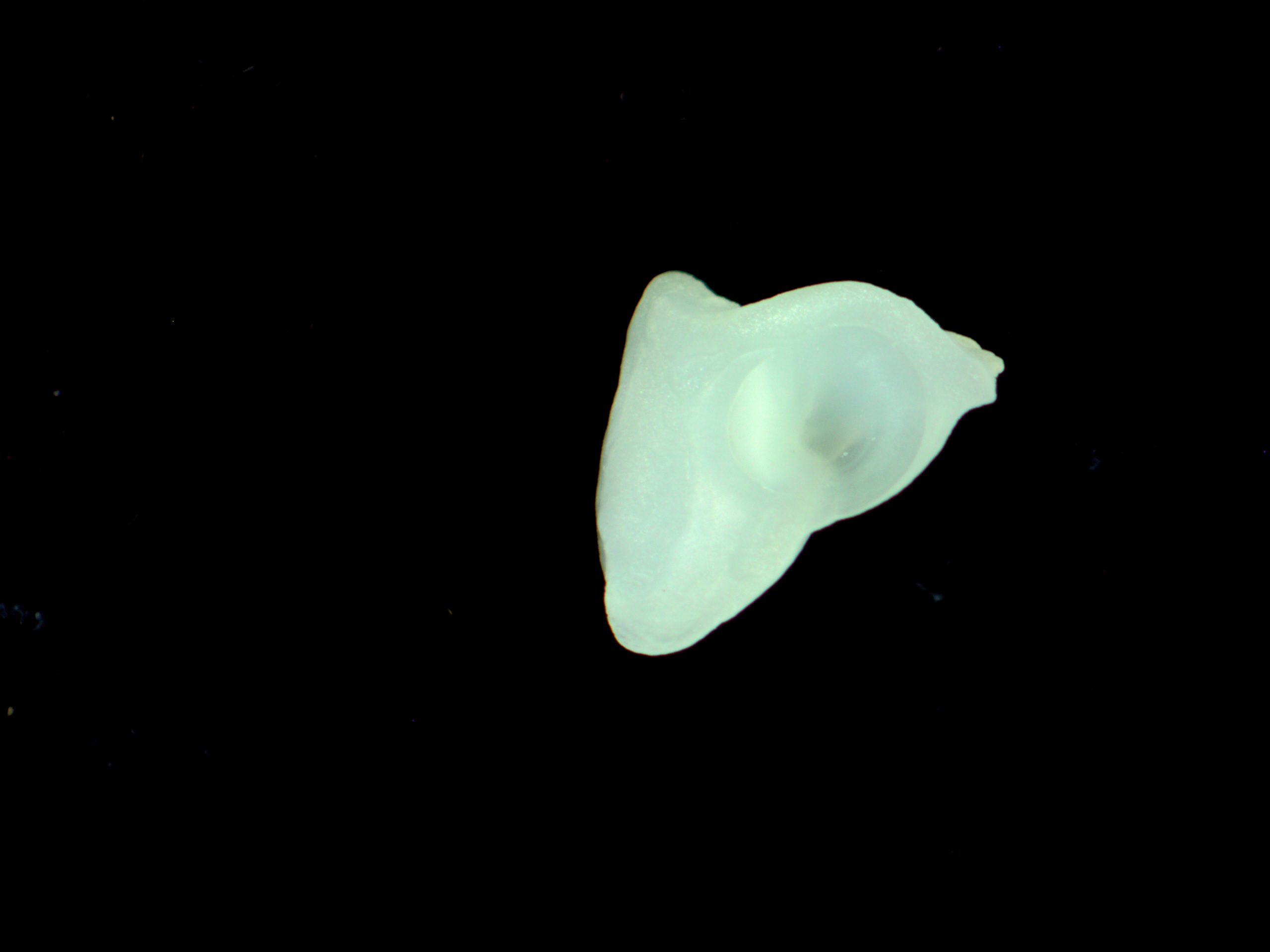

Supplement: Supplemental Information 13 [file peerj-04-1664-s013.zip › JohCar/training/26R1.jpg]

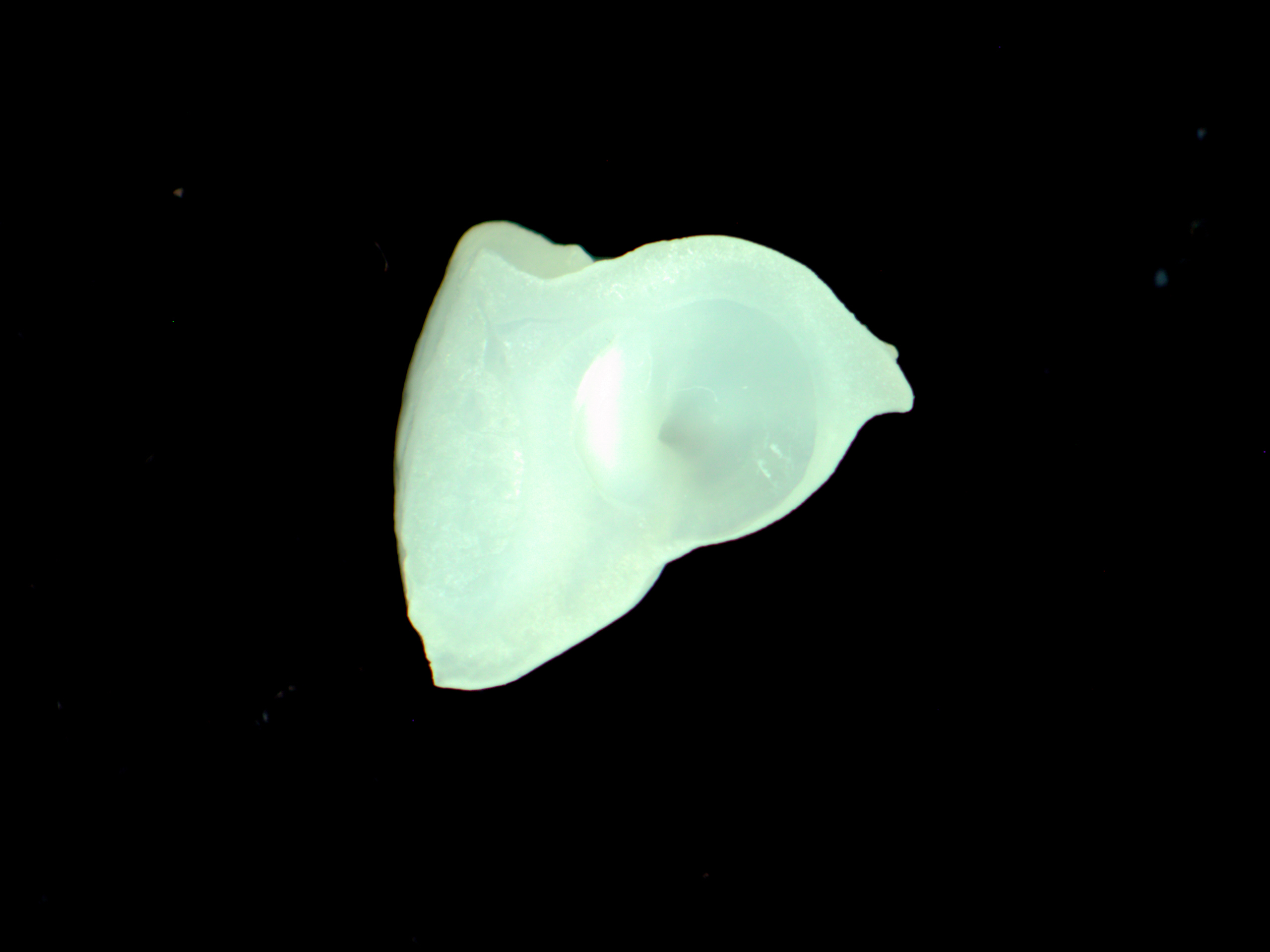

Supplement: Supplemental Information 13 [file peerj-04-1664-s013.zip › JohCar/training/28R1.jpg]

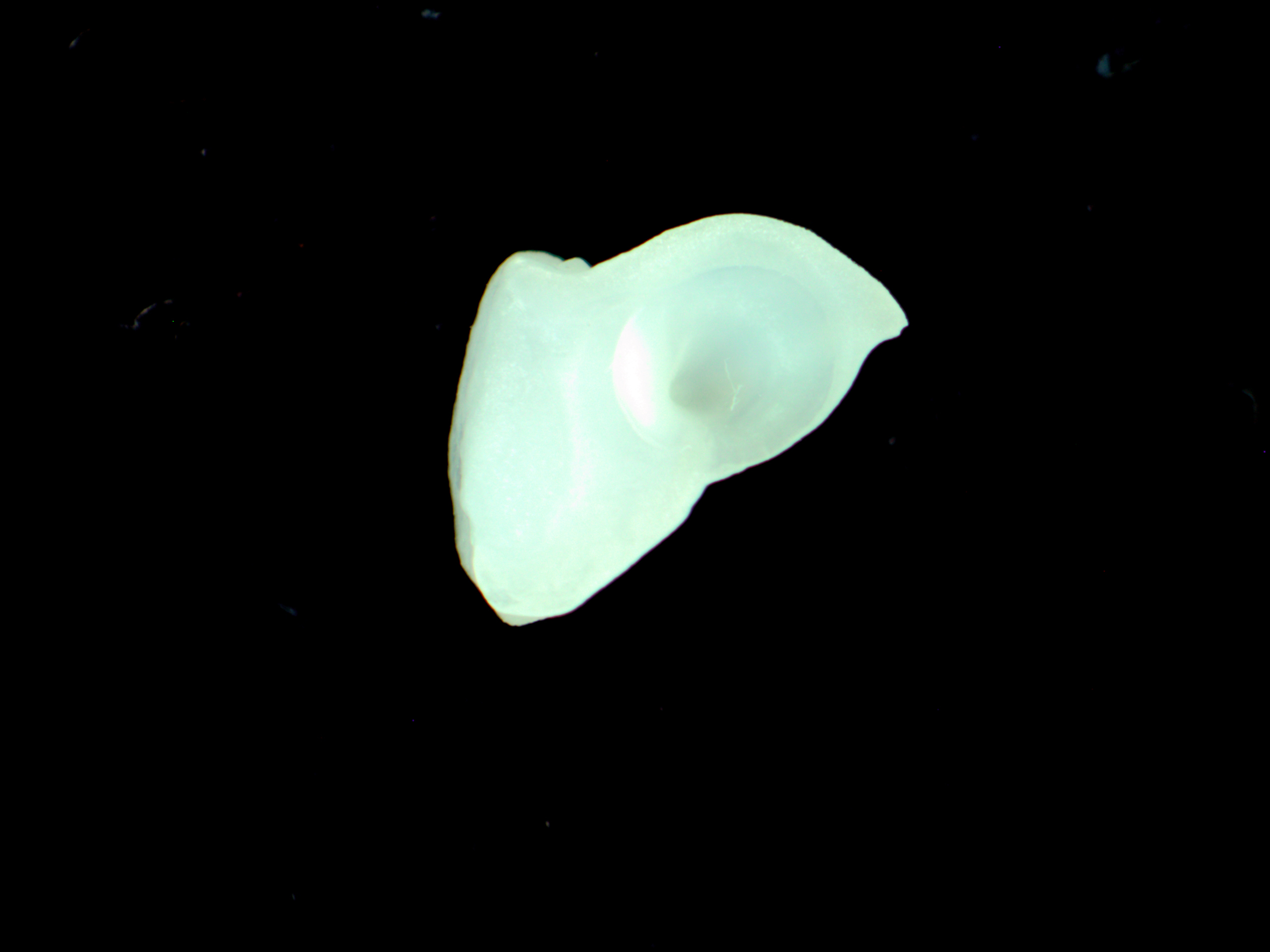

Supplement: Supplemental Information 13 [file peerj-04-1664-s013.zip › JohCar/training/29R1.jpg]

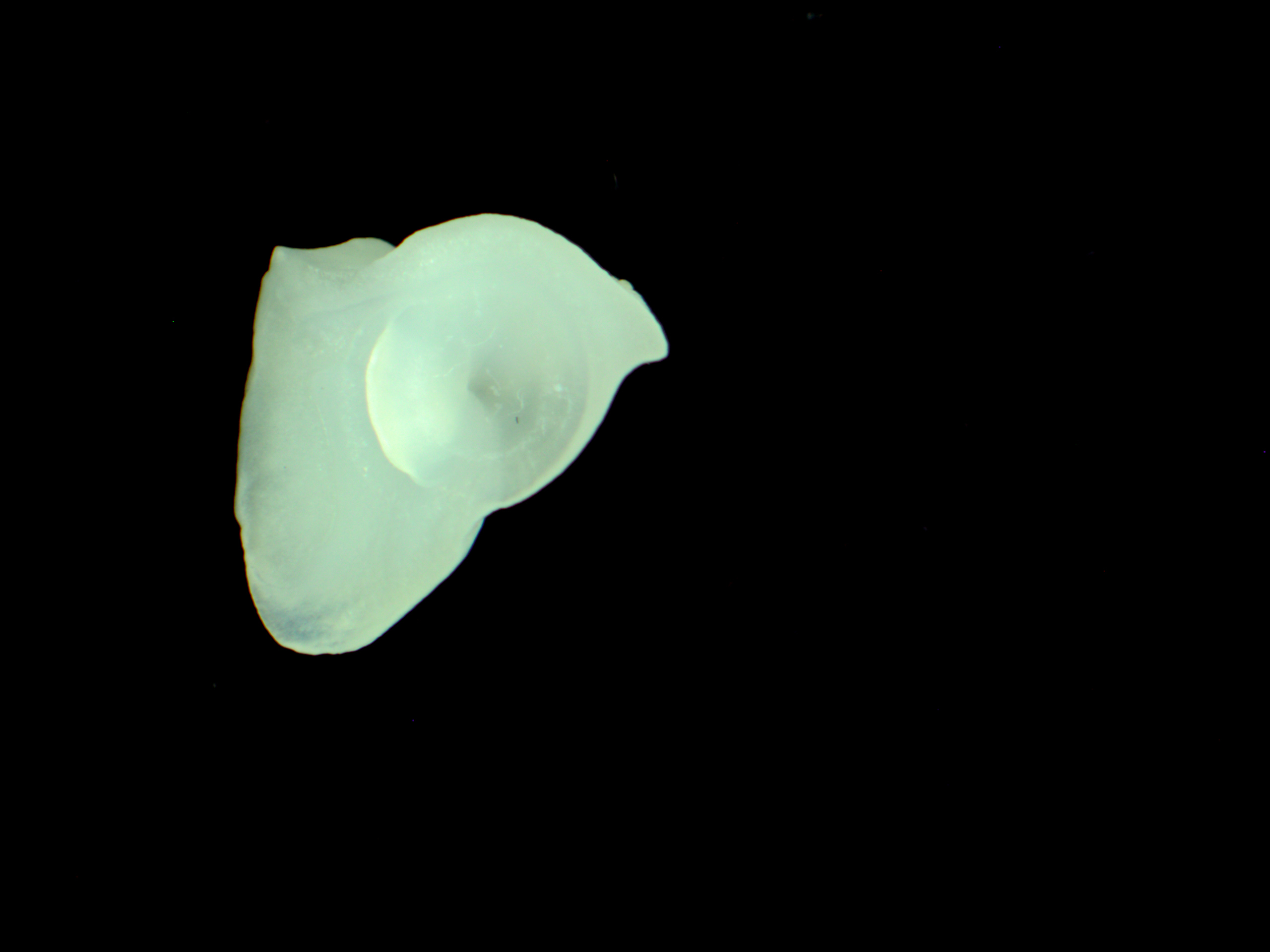

Supplement: Supplemental Information 13 [file peerj-04-1664-s013.zip › JohCar/training/31R1.jpg]

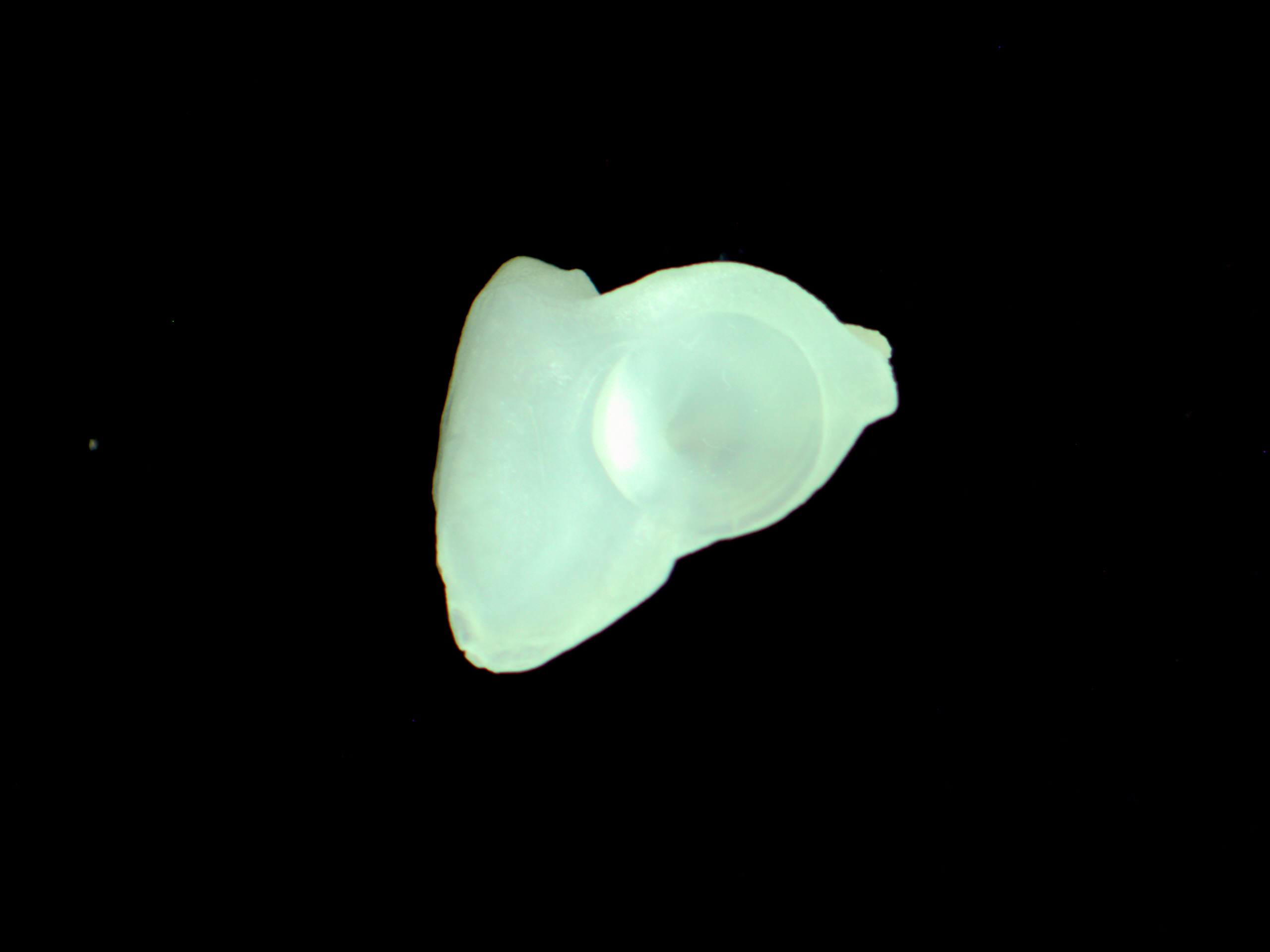

Supplement: Supplemental Information 13 [file peerj-04-1664-s013.zip › JohCar/training/32R1.jpg]

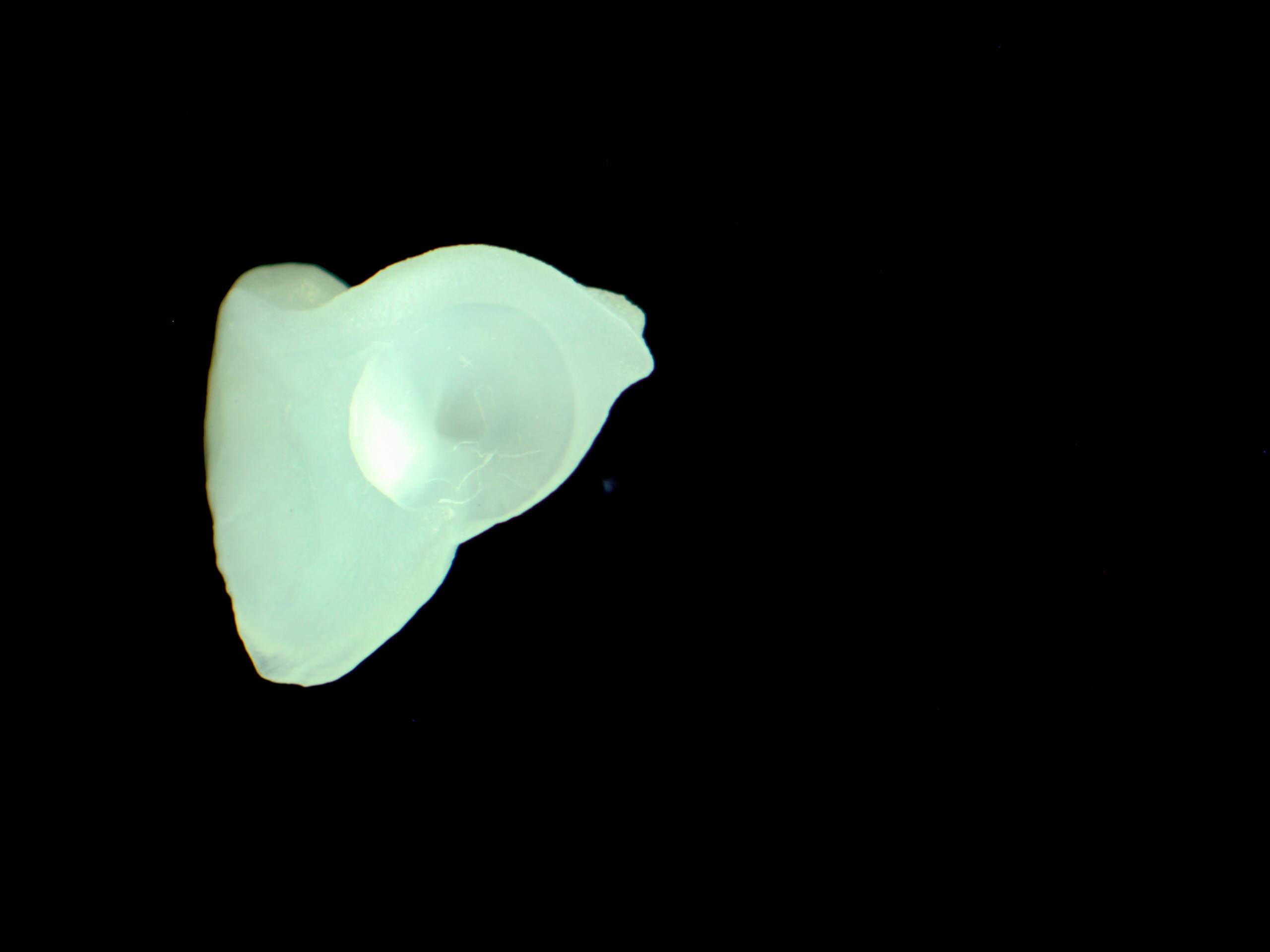

Supplement: Supplemental Information 13 [file peerj-04-1664-s013.zip › JohCar/training/33R1.jpg]

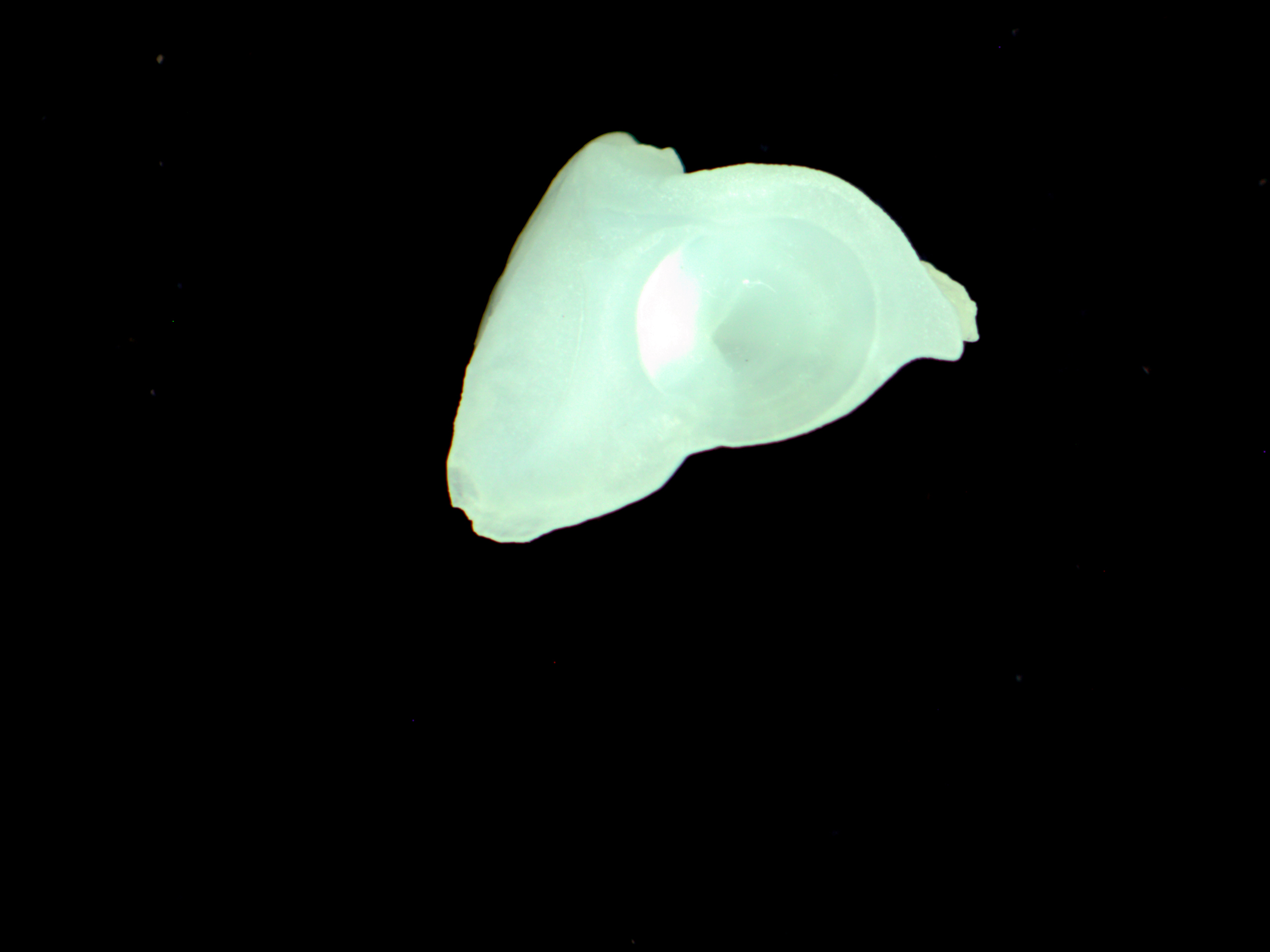

Supplement: Supplemental Information 13 [file peerj-04-1664-s013.zip › JohCar/training/34R1.jpg]

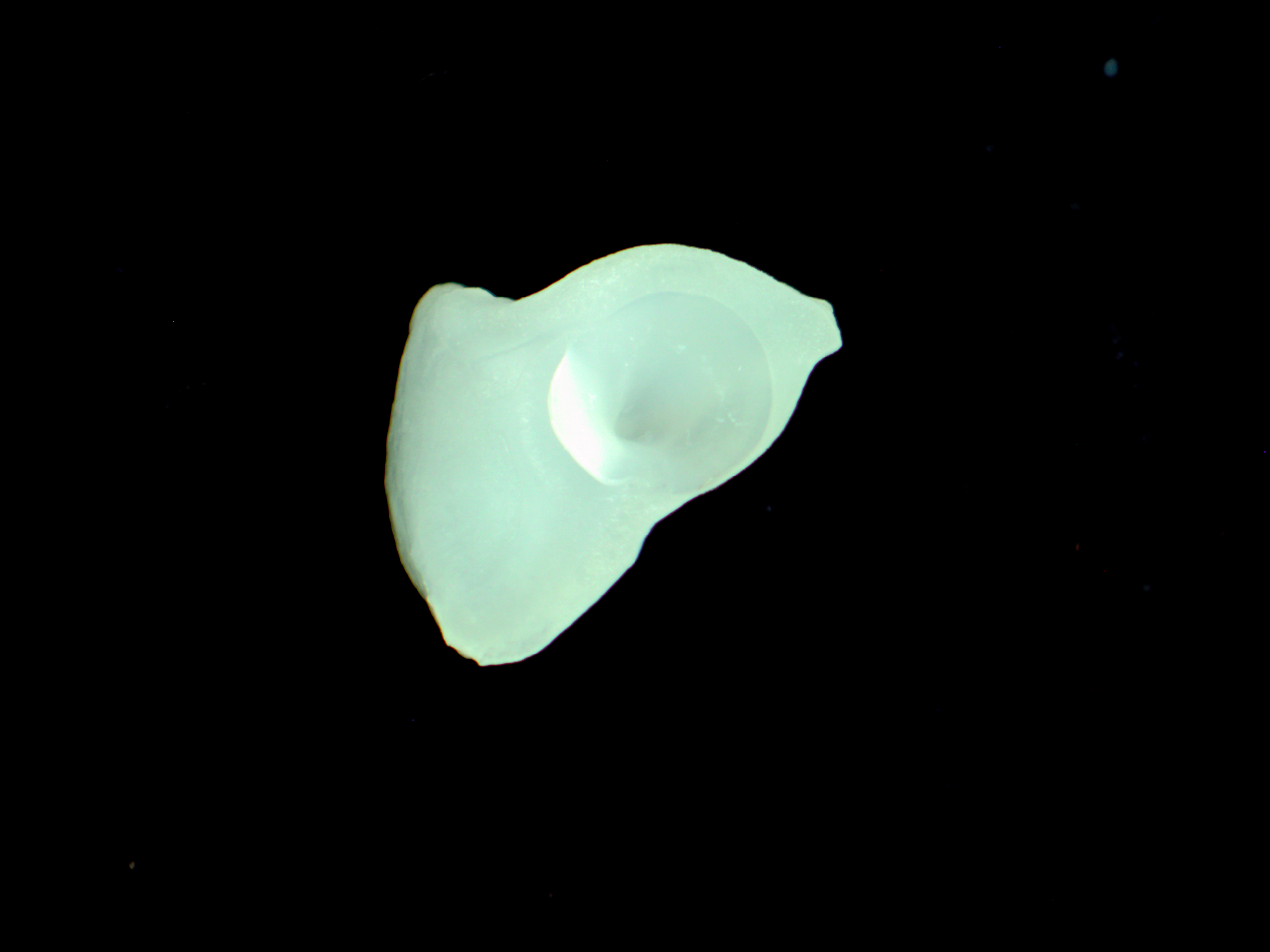

Supplement: Supplemental Information 13 [file peerj-04-1664-s013.zip › JohCar/training/35R1.jpg]

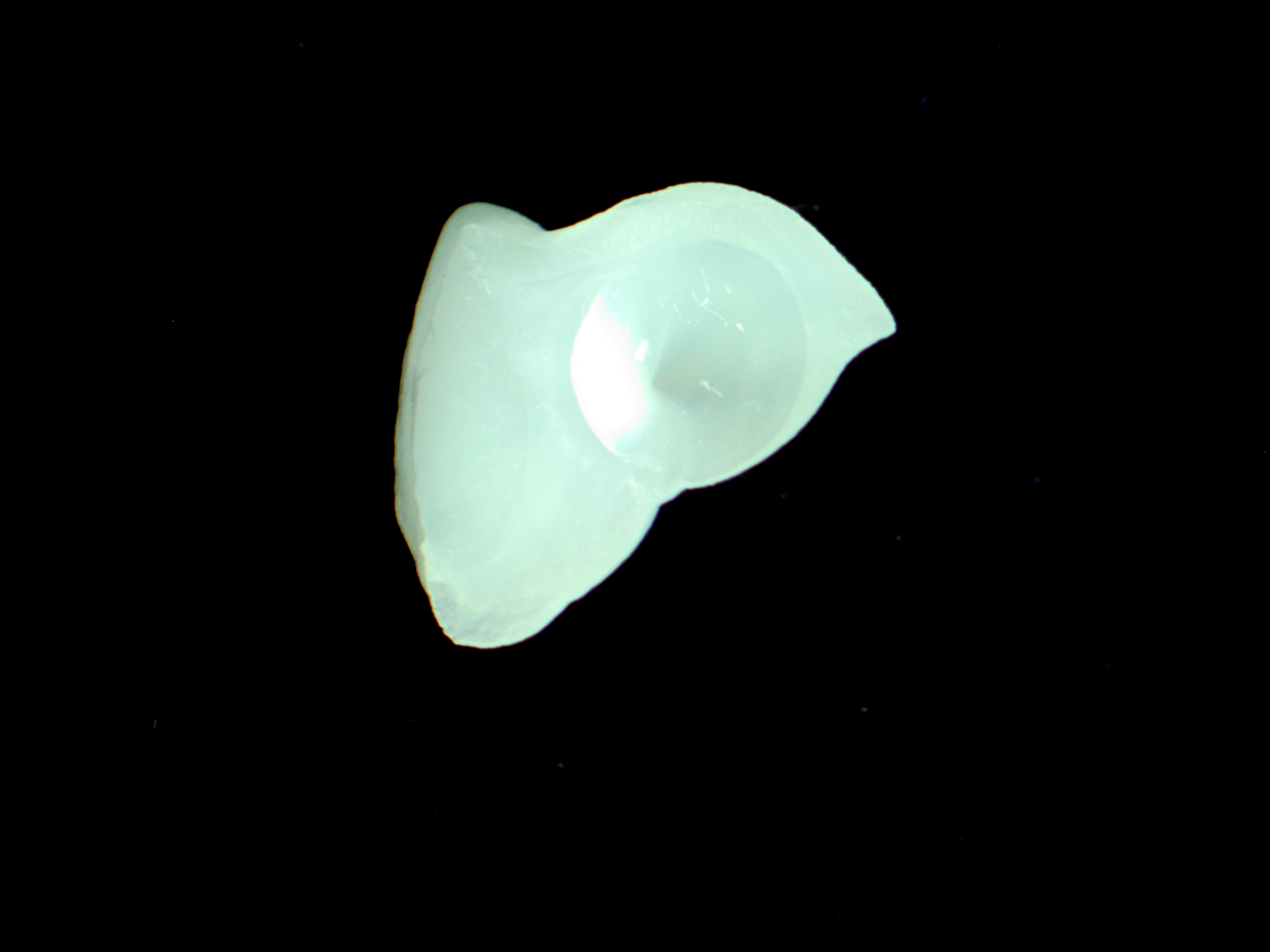

Supplement: Supplemental Information 13 [file peerj-04-1664-s013.zip › JohCar/training/36R1.jpg]

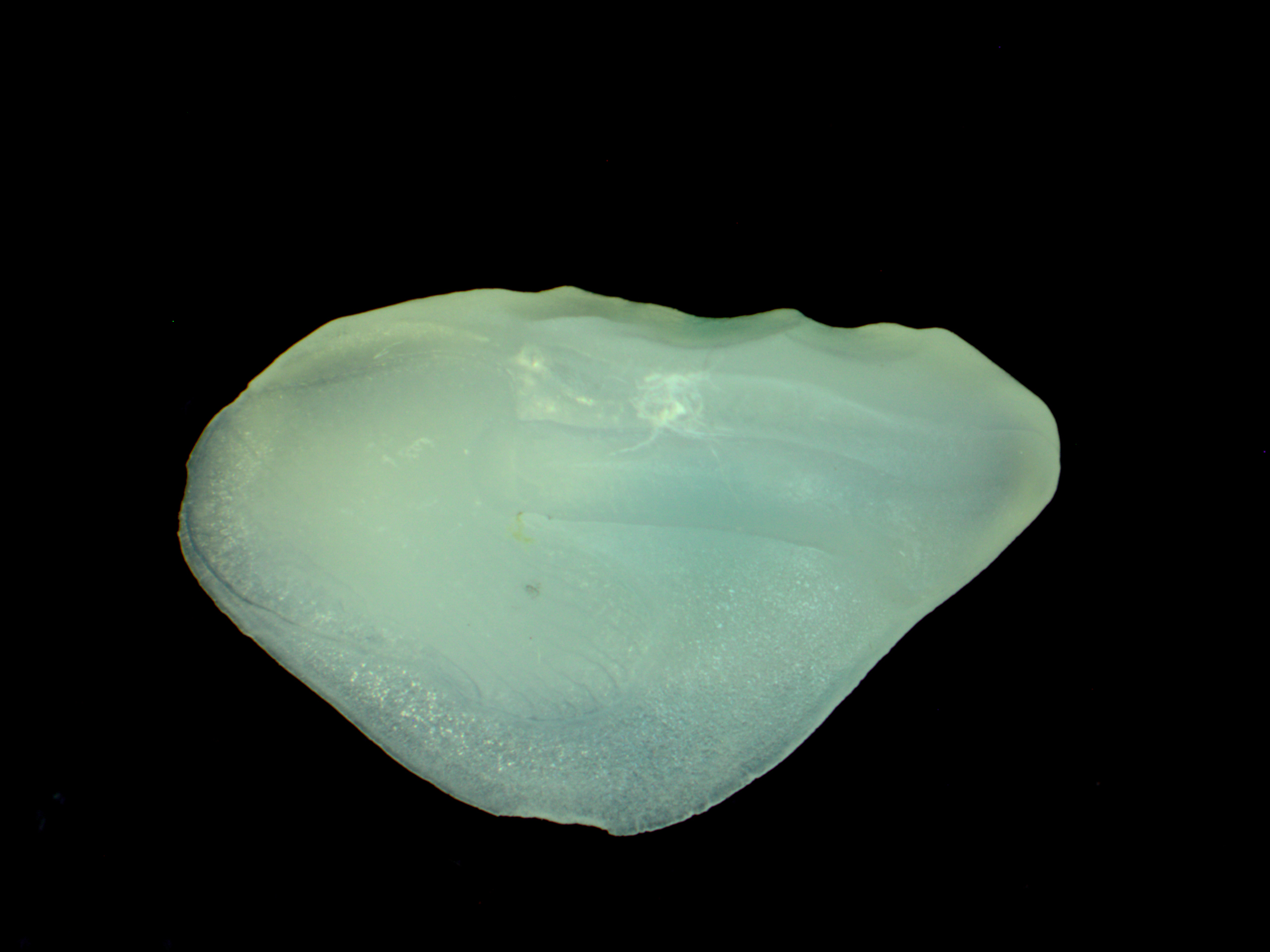

Supplement: Supplemental Information 14 [file peerj-04-1664-s014.zip › OtoRub/testing/S102R1.jpg]

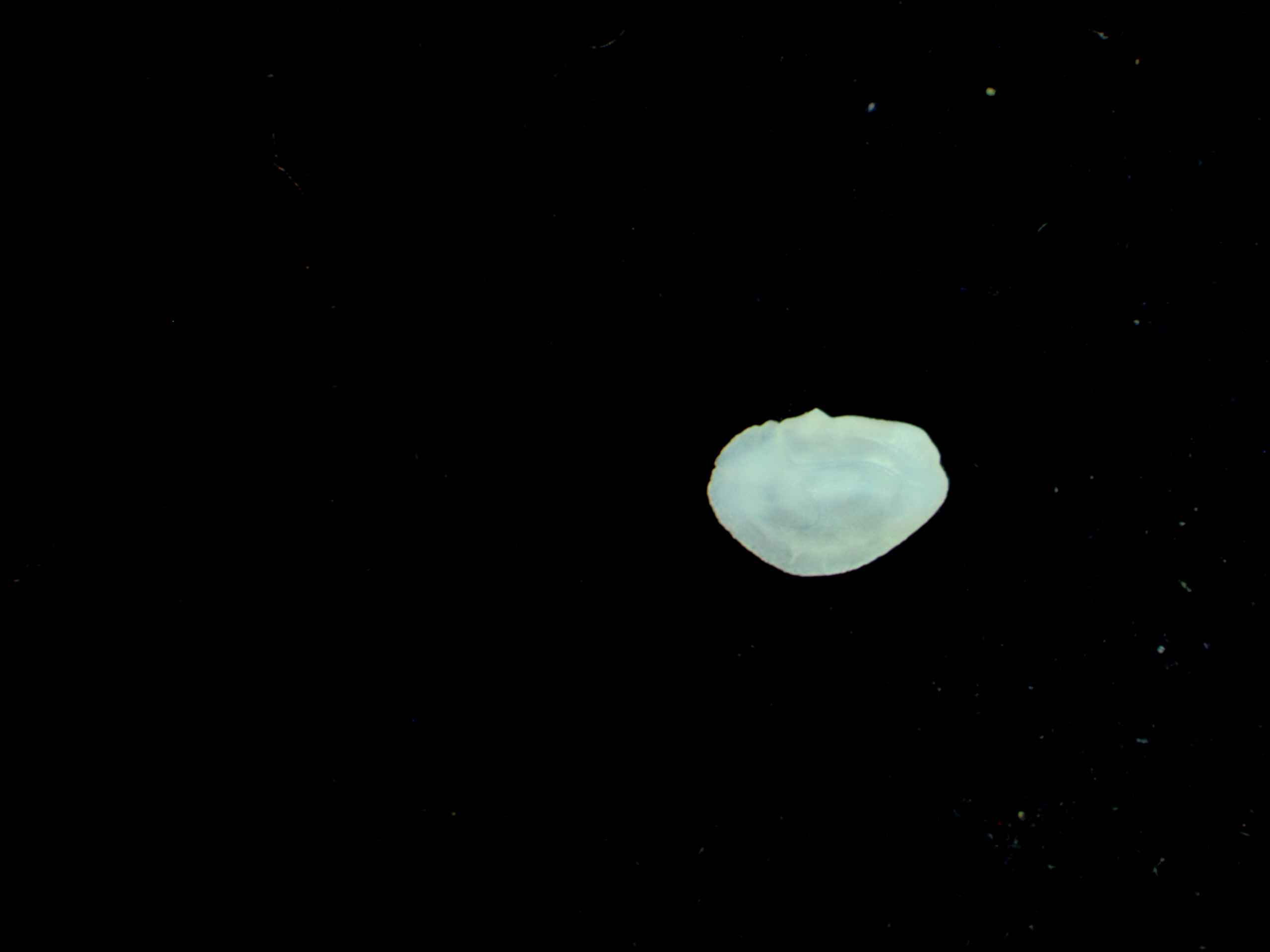

Supplement: Supplemental Information 14 [file peerj-04-1664-s014.zip › OtoRub/testing/S18R1.jpg]

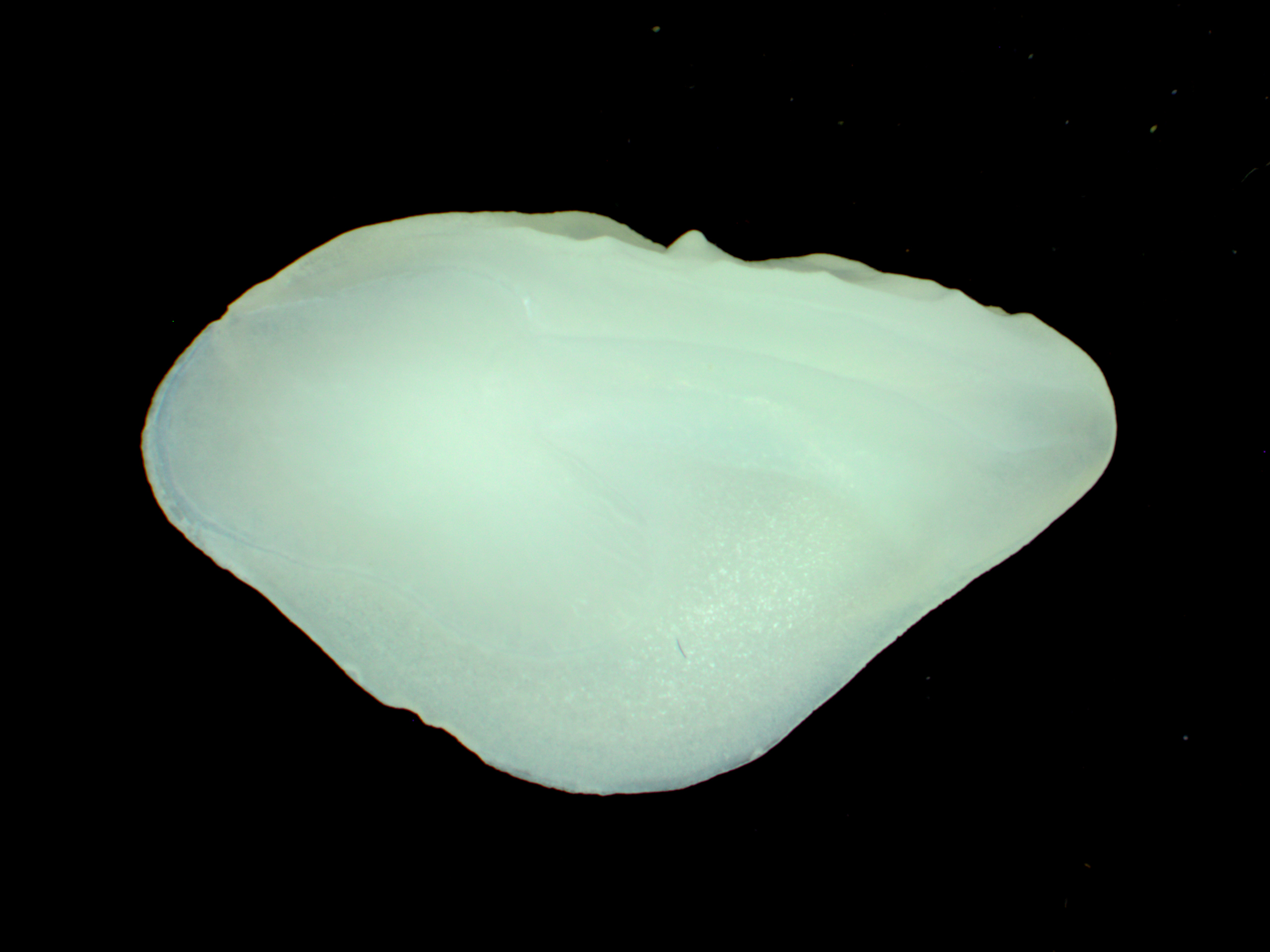

Supplement: Supplemental Information 14 [file peerj-04-1664-s014.zip › OtoRub/testing/S46R1.jpg]

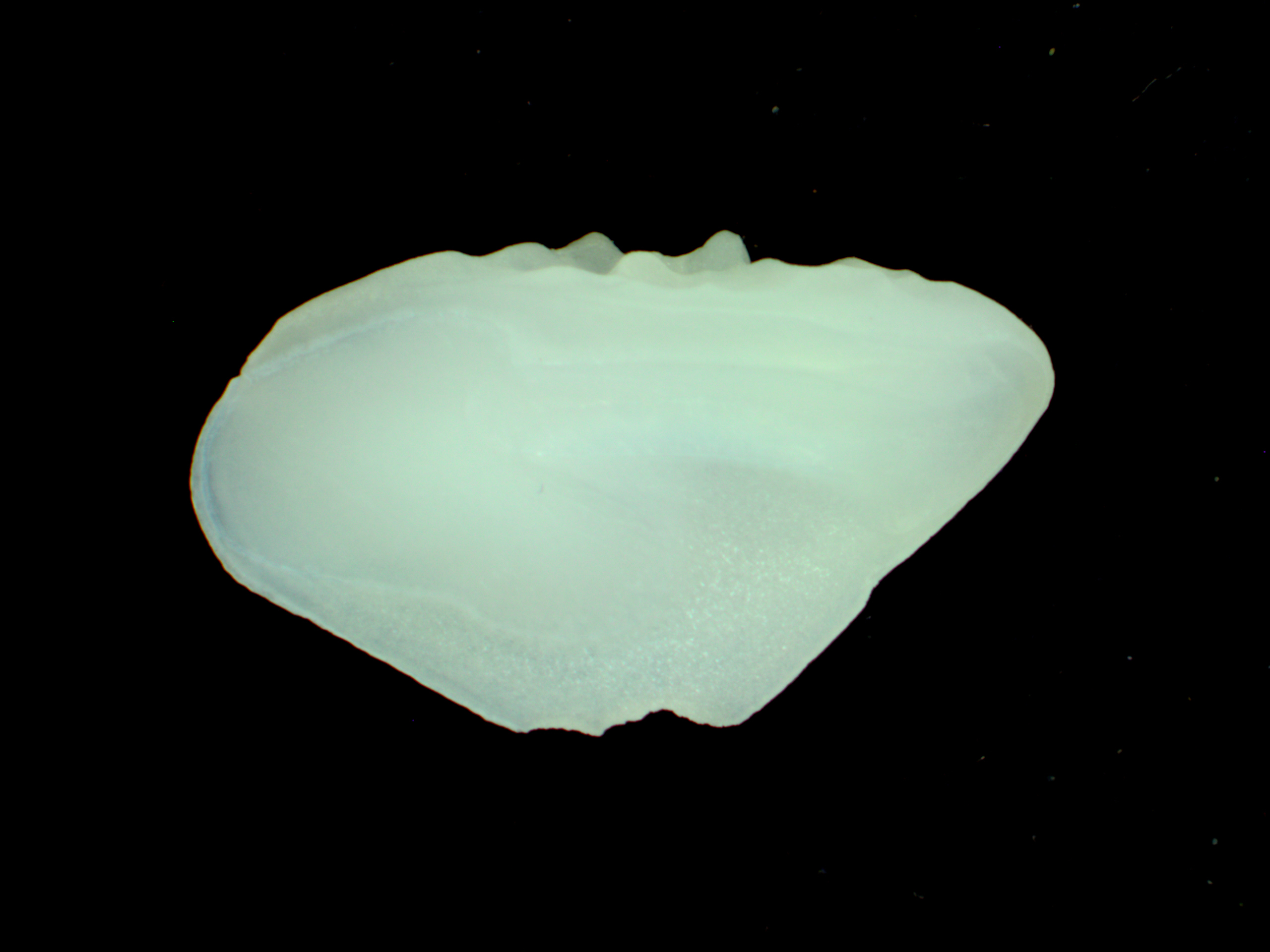

Supplement: Supplemental Information 14 [file peerj-04-1664-s014.zip › OtoRub/testing/S47R1.jpg]

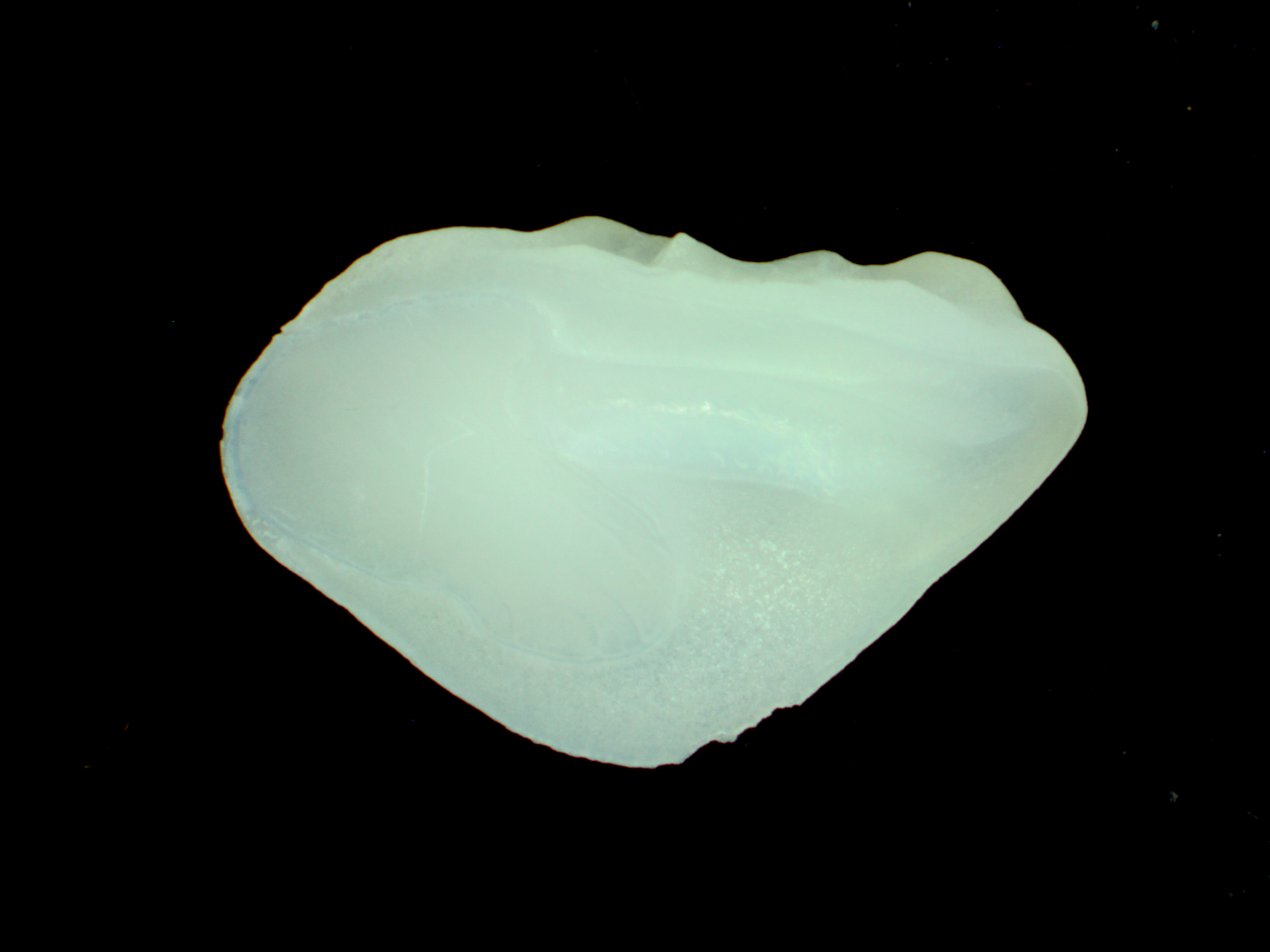

Supplement: Supplemental Information 14 [file peerj-04-1664-s014.zip › OtoRub/testing/S50R1.jpg]

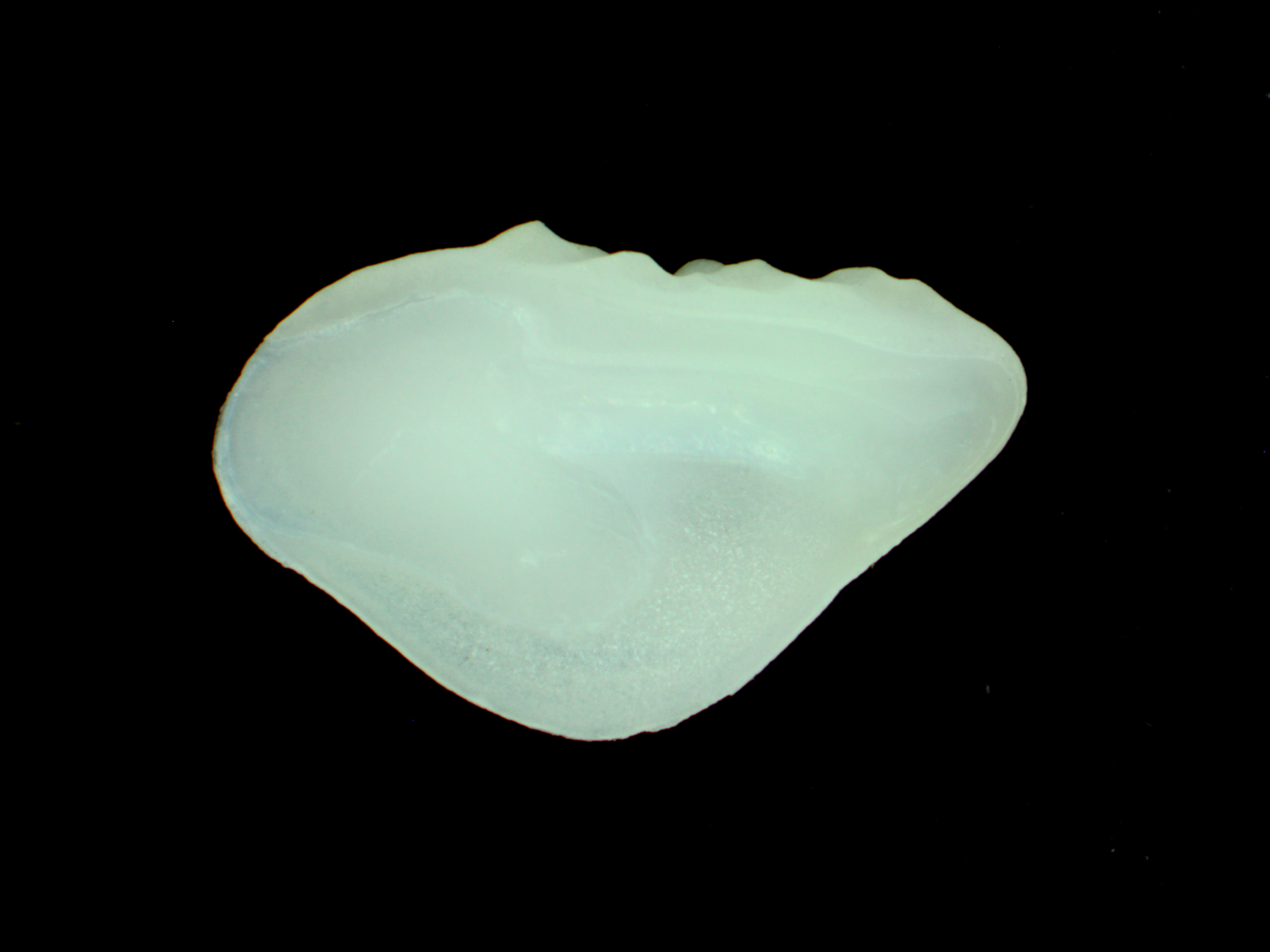

Supplement: Supplemental Information 14 [file peerj-04-1664-s014.zip › OtoRub/testing/S51R1.jpg]

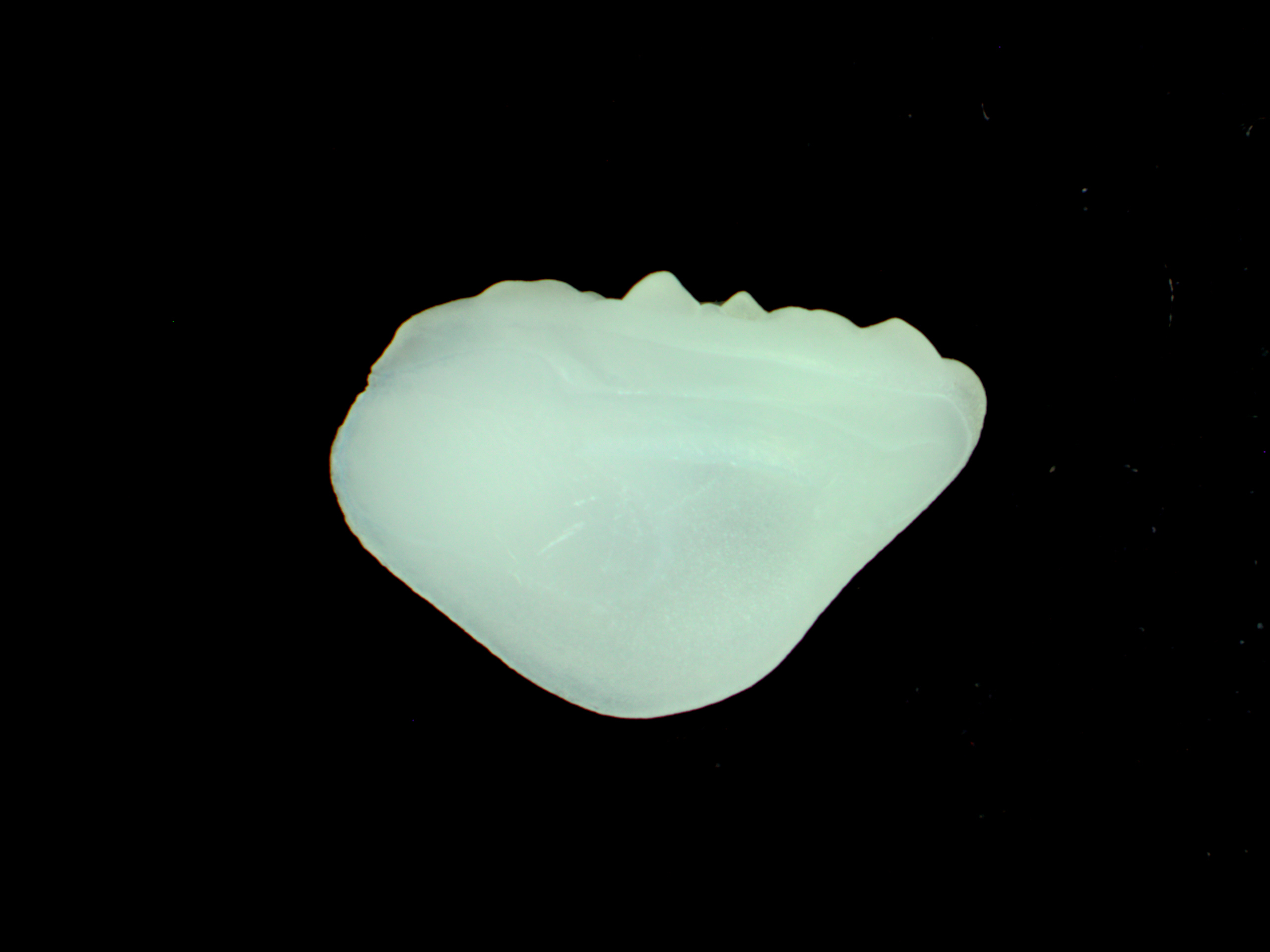

Supplement: Supplemental Information 14 [file peerj-04-1664-s014.zip › OtoRub/testing/S52R1.jpg]

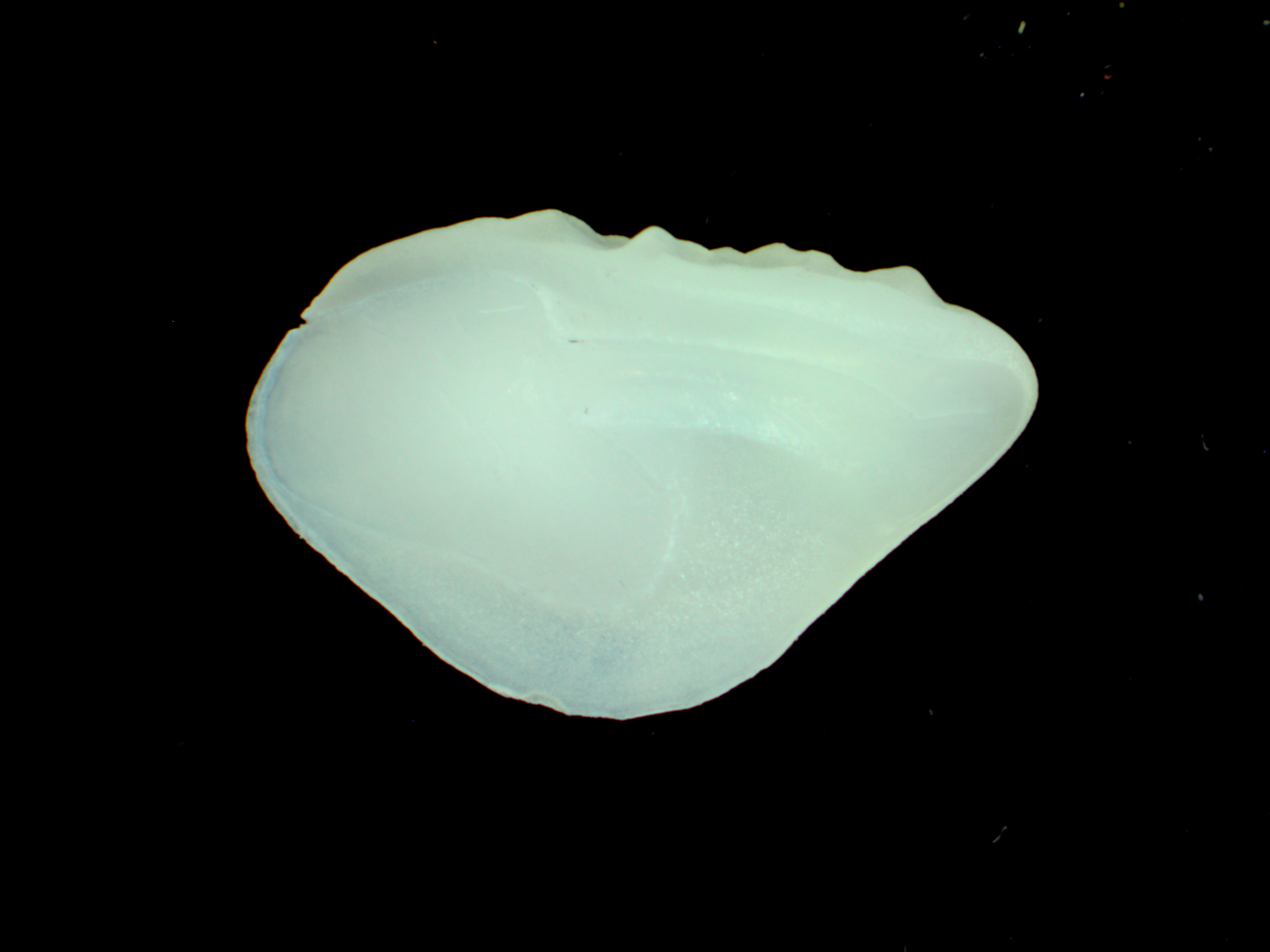

Supplement: Supplemental Information 14 [file peerj-04-1664-s014.zip › OtoRub/testing/S59R1.jpg]

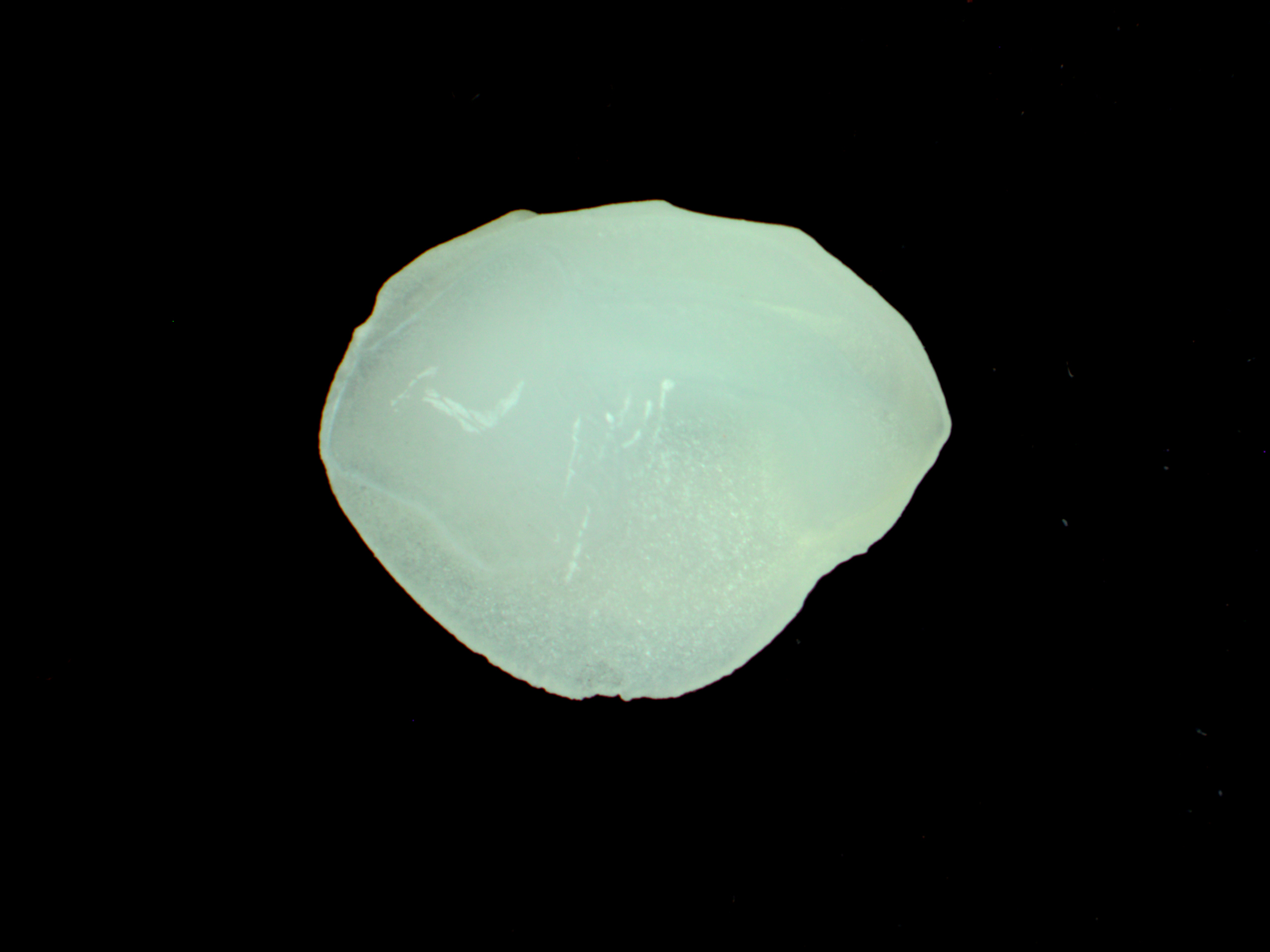

Supplement: Supplemental Information 14 [file peerj-04-1664-s014.zip › OtoRub/testing/S66R1.jpg]

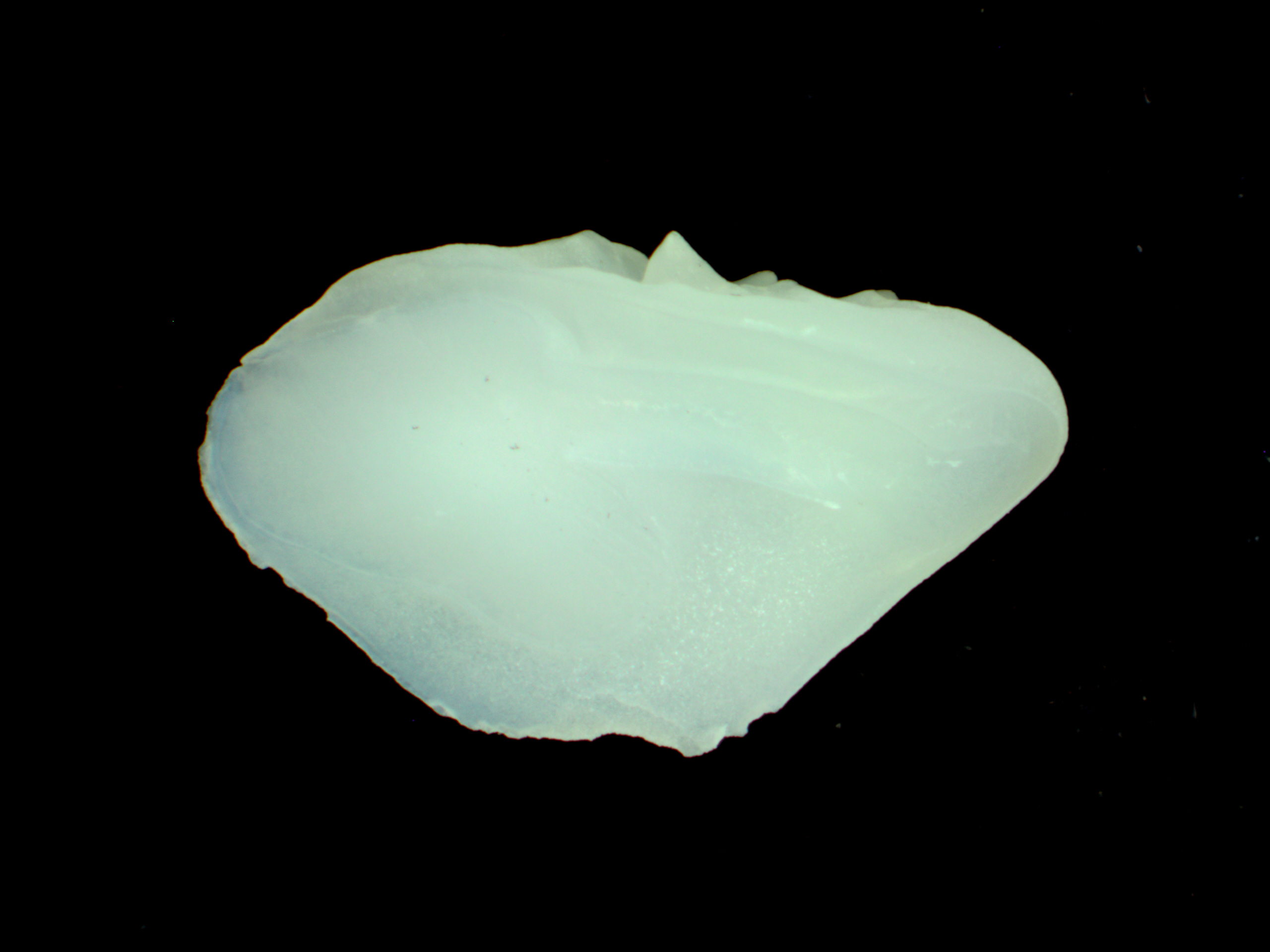

Supplement: Supplemental Information 14 [file peerj-04-1664-s014.zip › OtoRub/testing/S67R1.jpg]

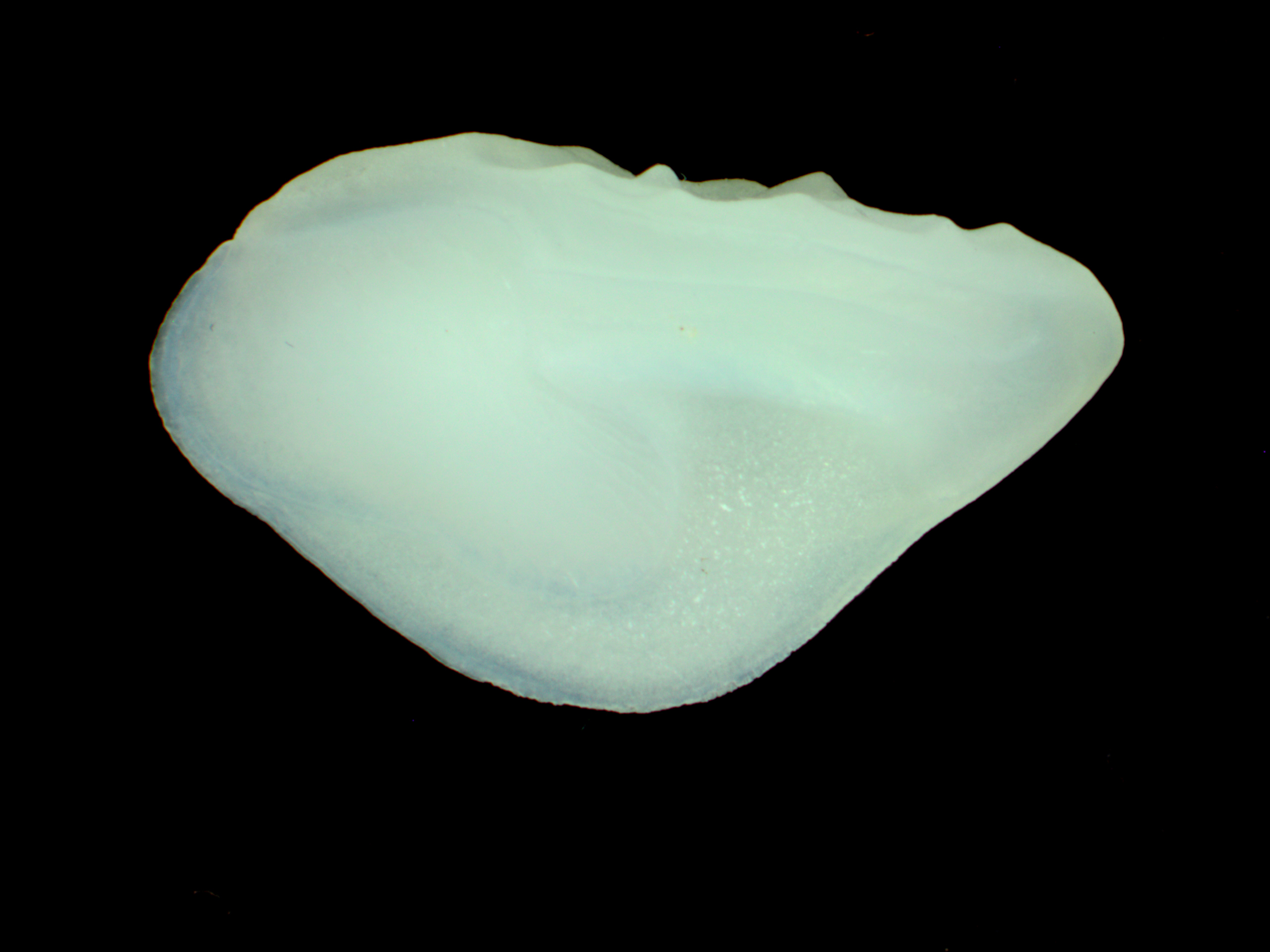

Supplement: Supplemental Information 14 [file peerj-04-1664-s014.zip › OtoRub/training/527R1.jpg]

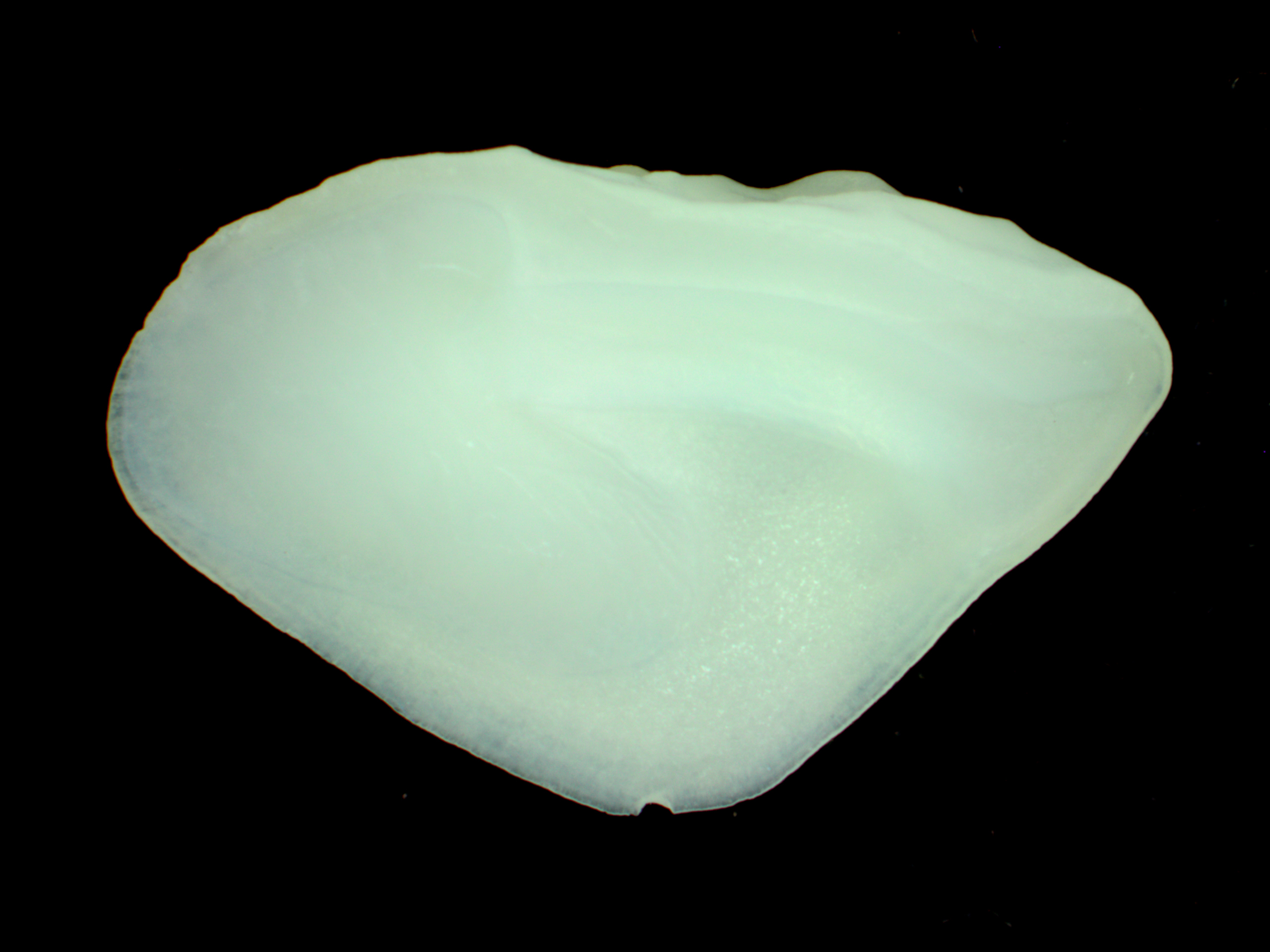

Supplement: Supplemental Information 14 [file peerj-04-1664-s014.zip › OtoRub/training/528R1.jpg]

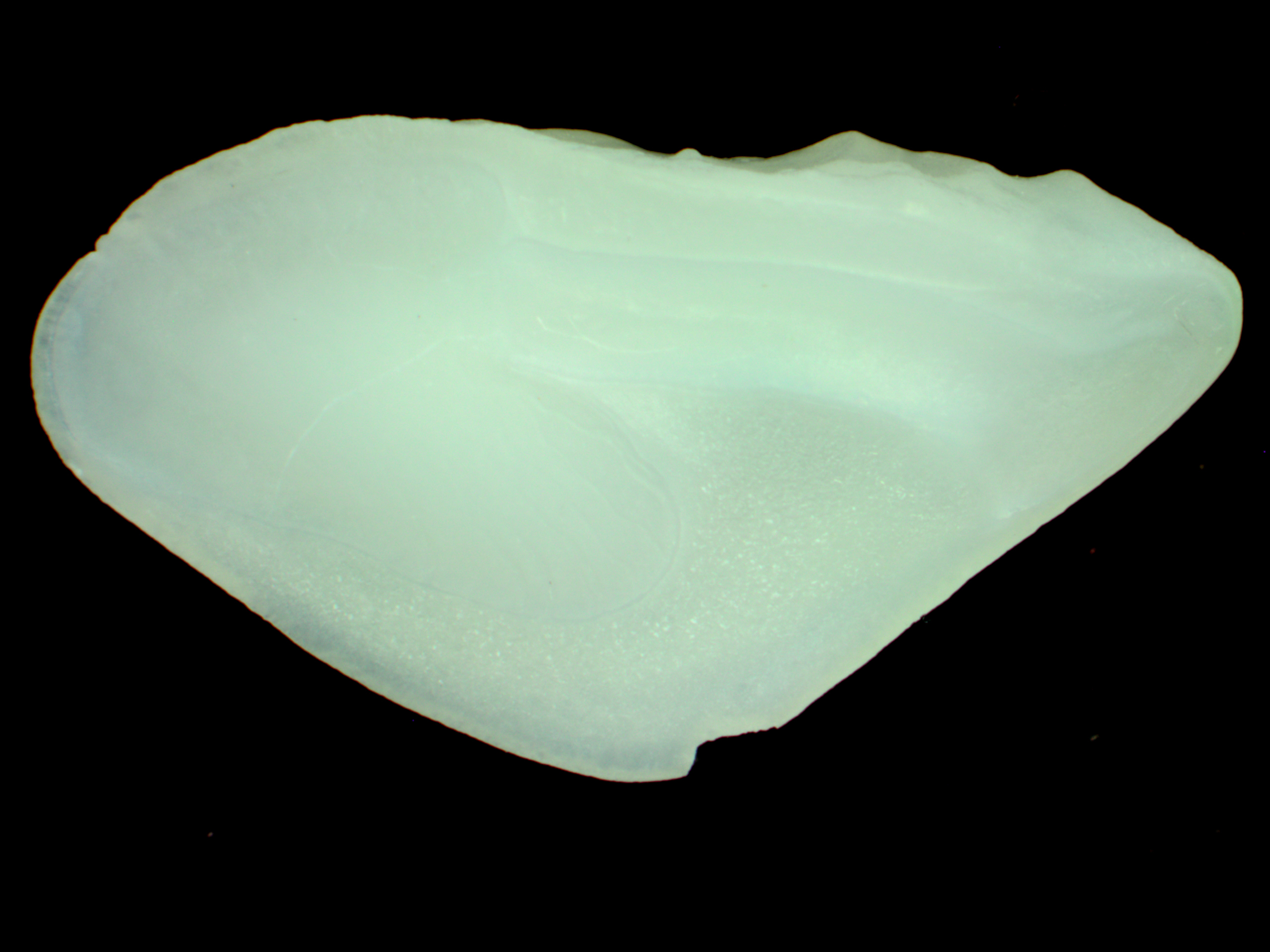

Supplement: Supplemental Information 14 [file peerj-04-1664-s014.zip › OtoRub/training/529R1.jpg]

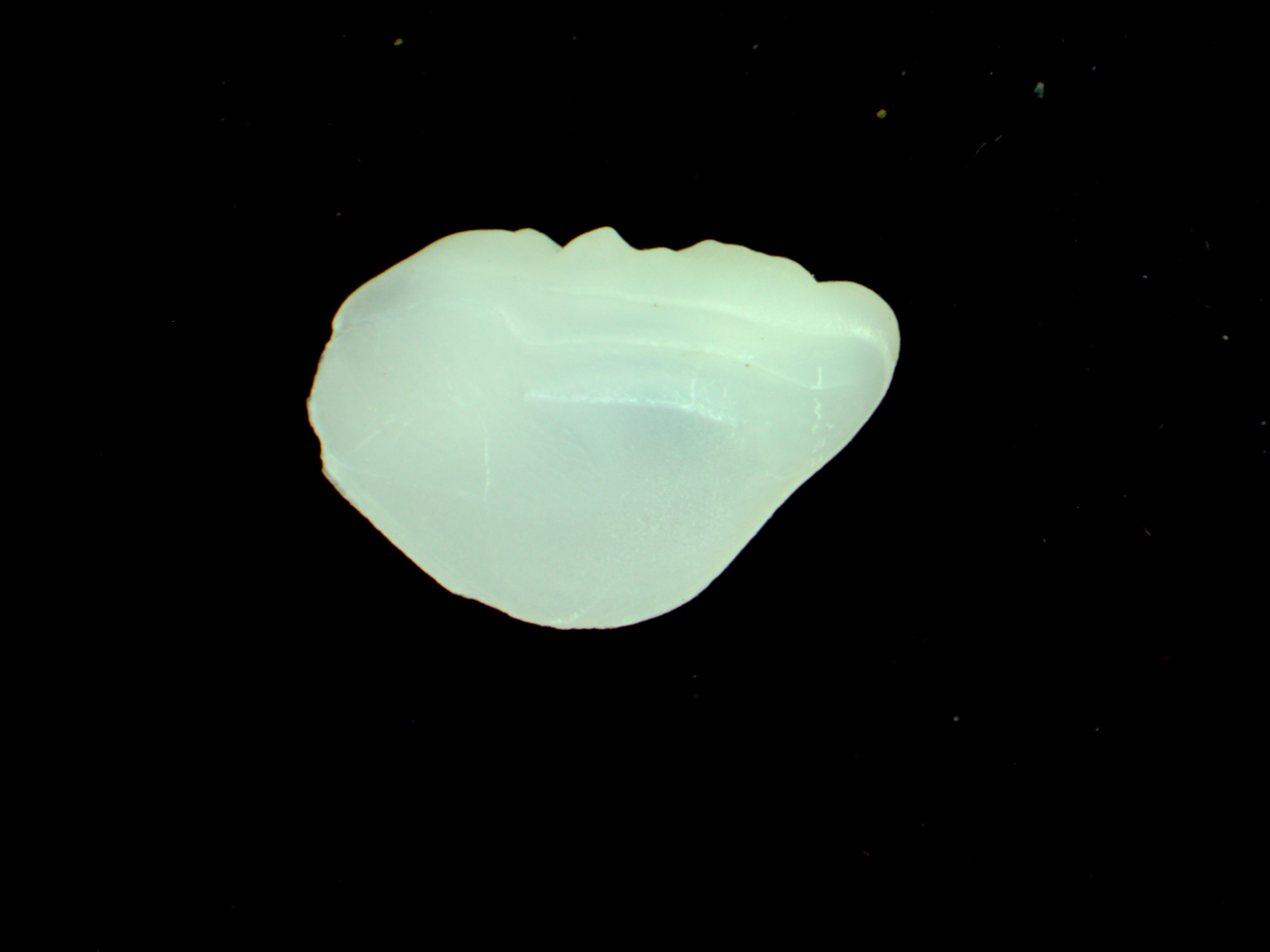

Supplement: Supplemental Information 14 [file peerj-04-1664-s014.zip › OtoRub/training/B77R1.jpg]

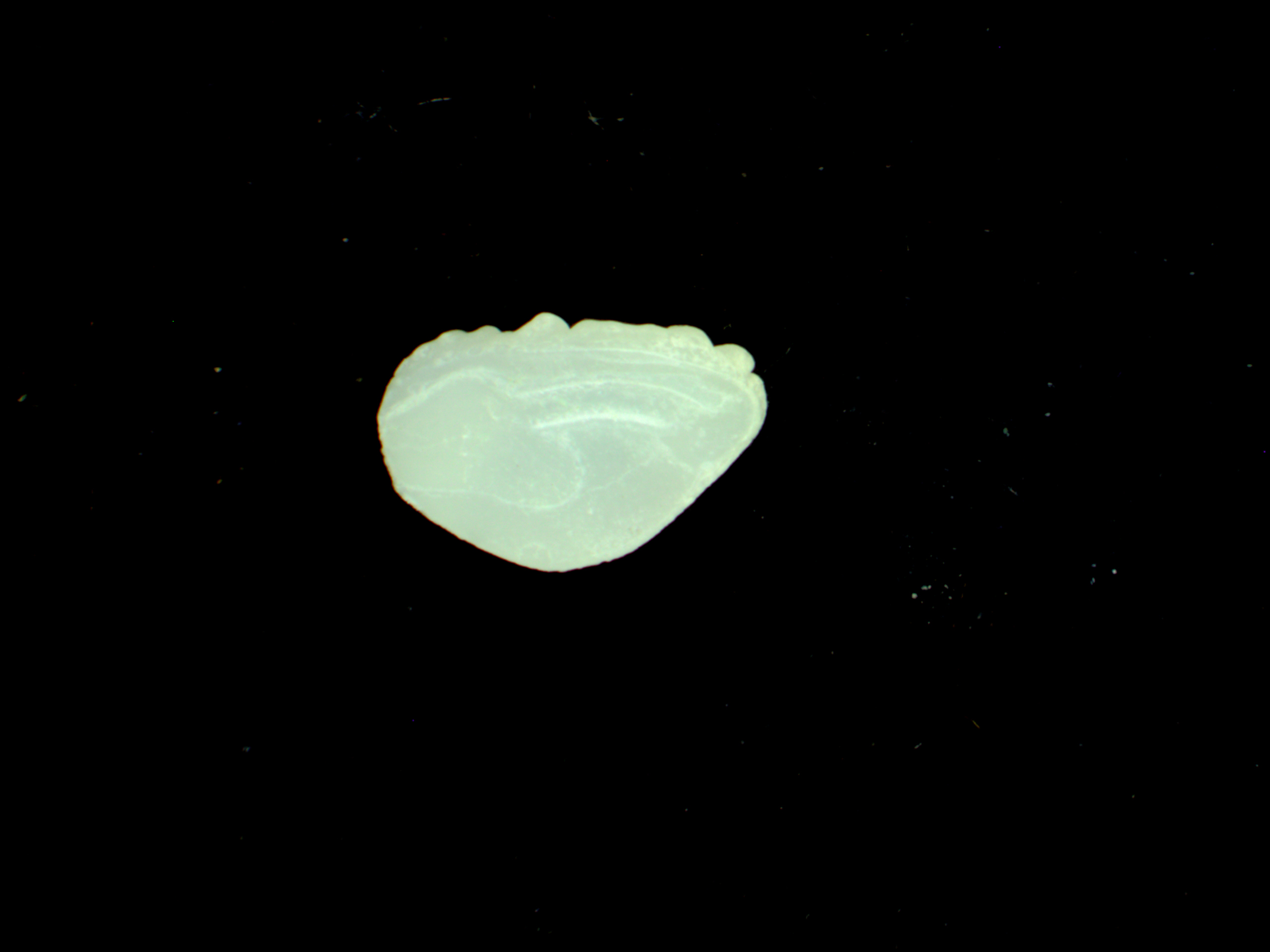

Supplement: Supplemental Information 14 [file peerj-04-1664-s014.zip › OtoRub/training/C77R1.jpg]

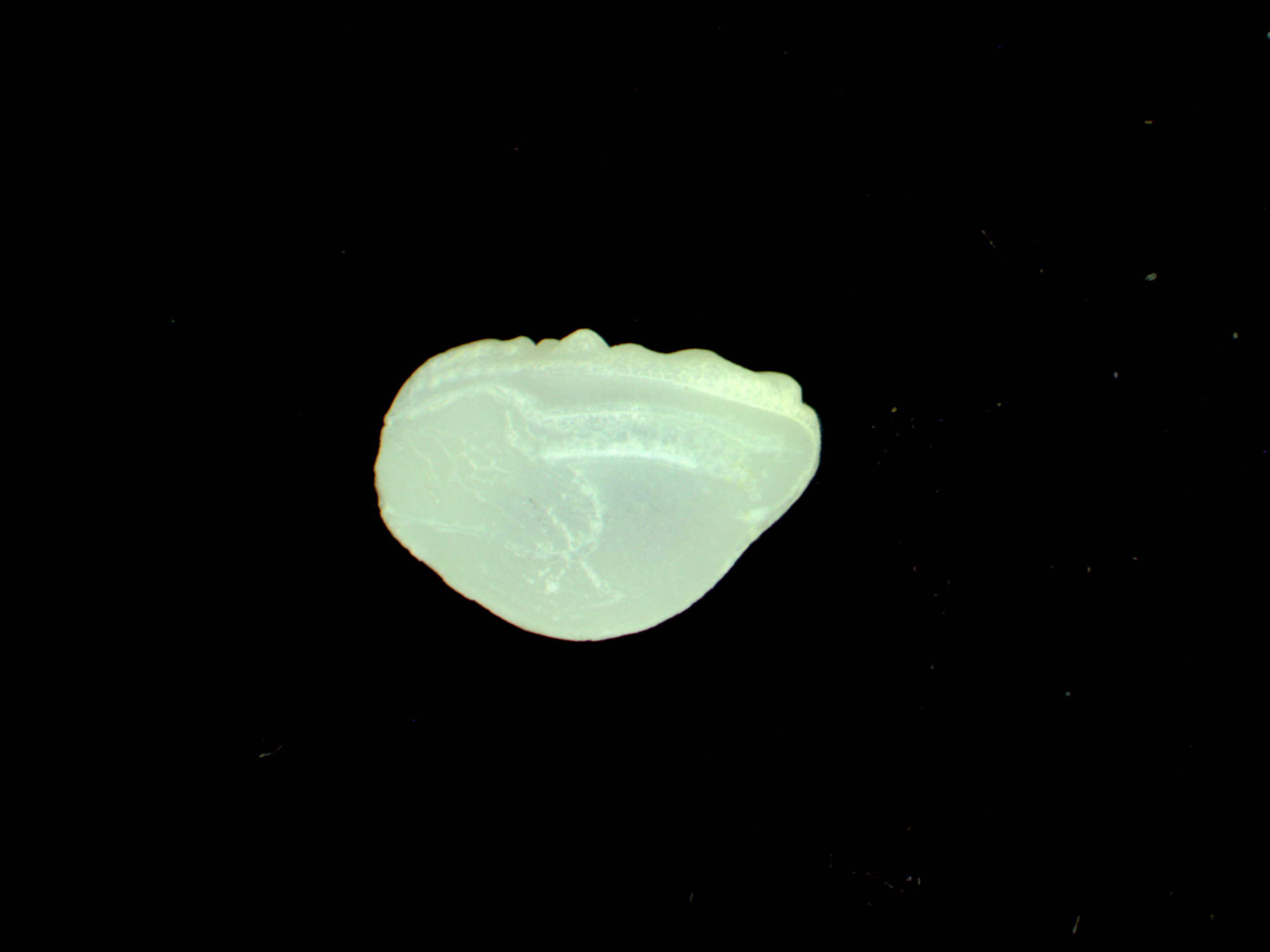

Supplement: Supplemental Information 14 [file peerj-04-1664-s014.zip › OtoRub/training/D40R1.jpg]

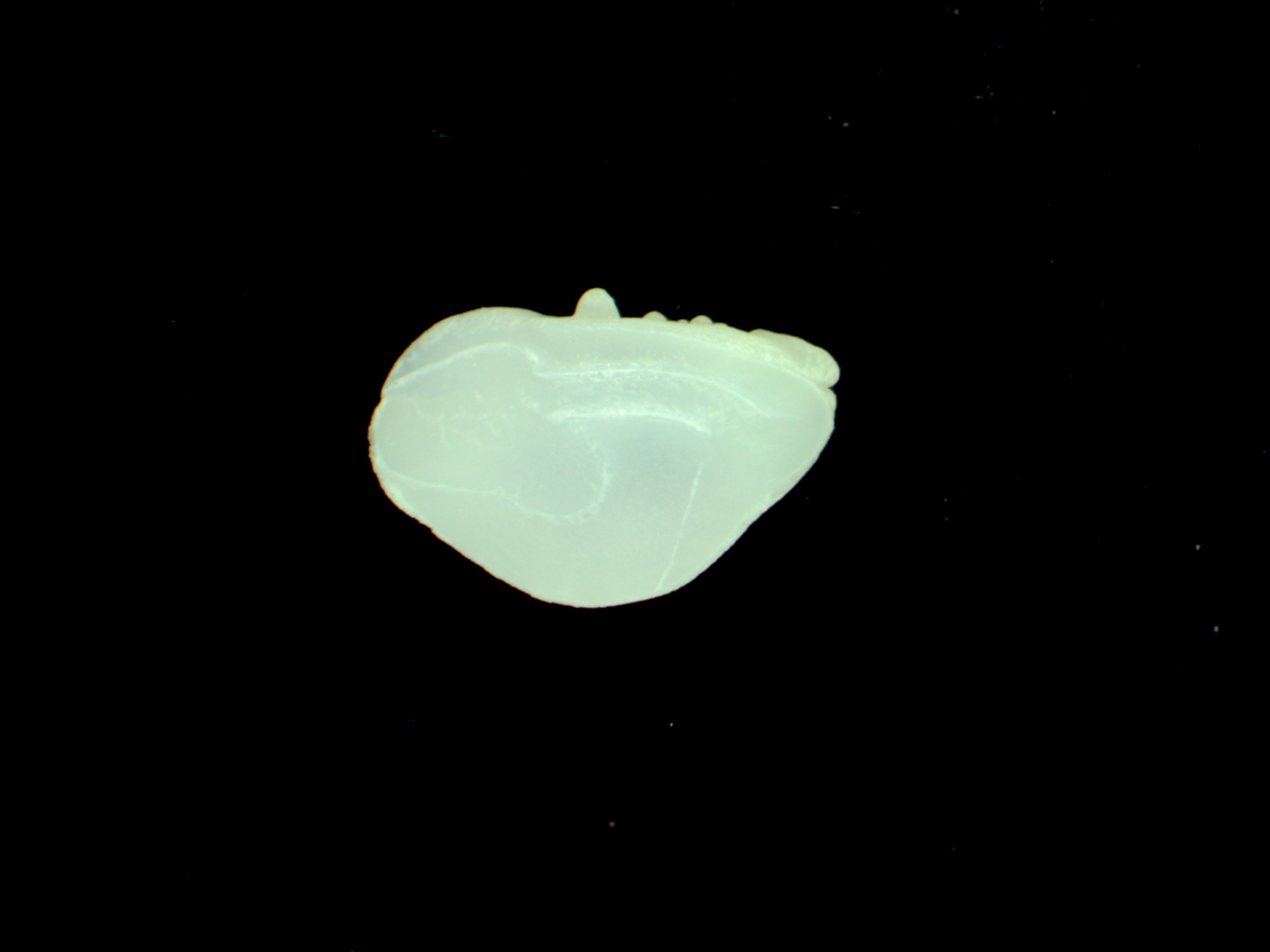

Supplement: Supplemental Information 14 [file peerj-04-1664-s014.zip › OtoRub/training/D55R1.jpg]

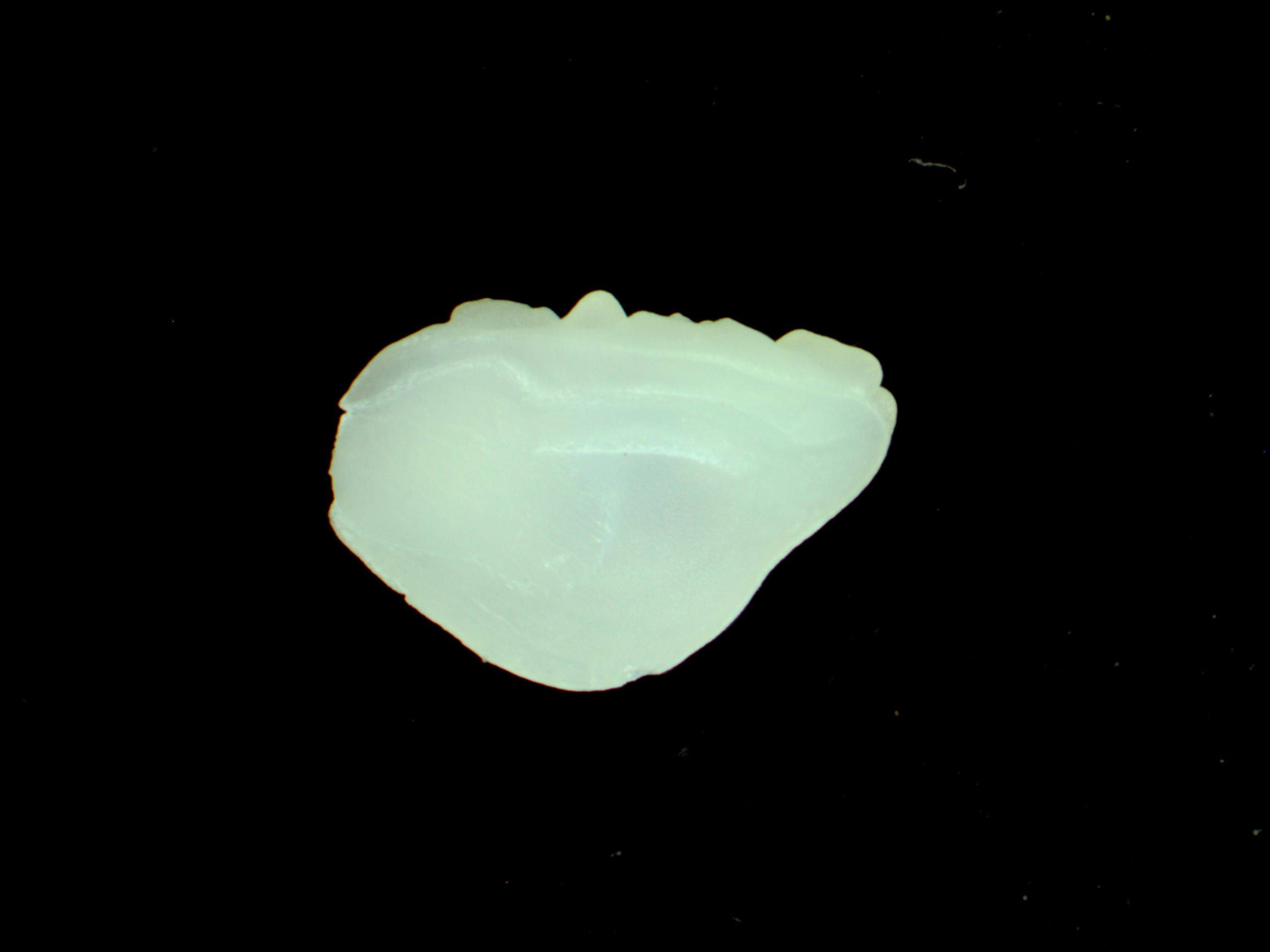

Supplement: Supplemental Information 14 [file peerj-04-1664-s014.zip › OtoRub/training/D76R1.jpg]

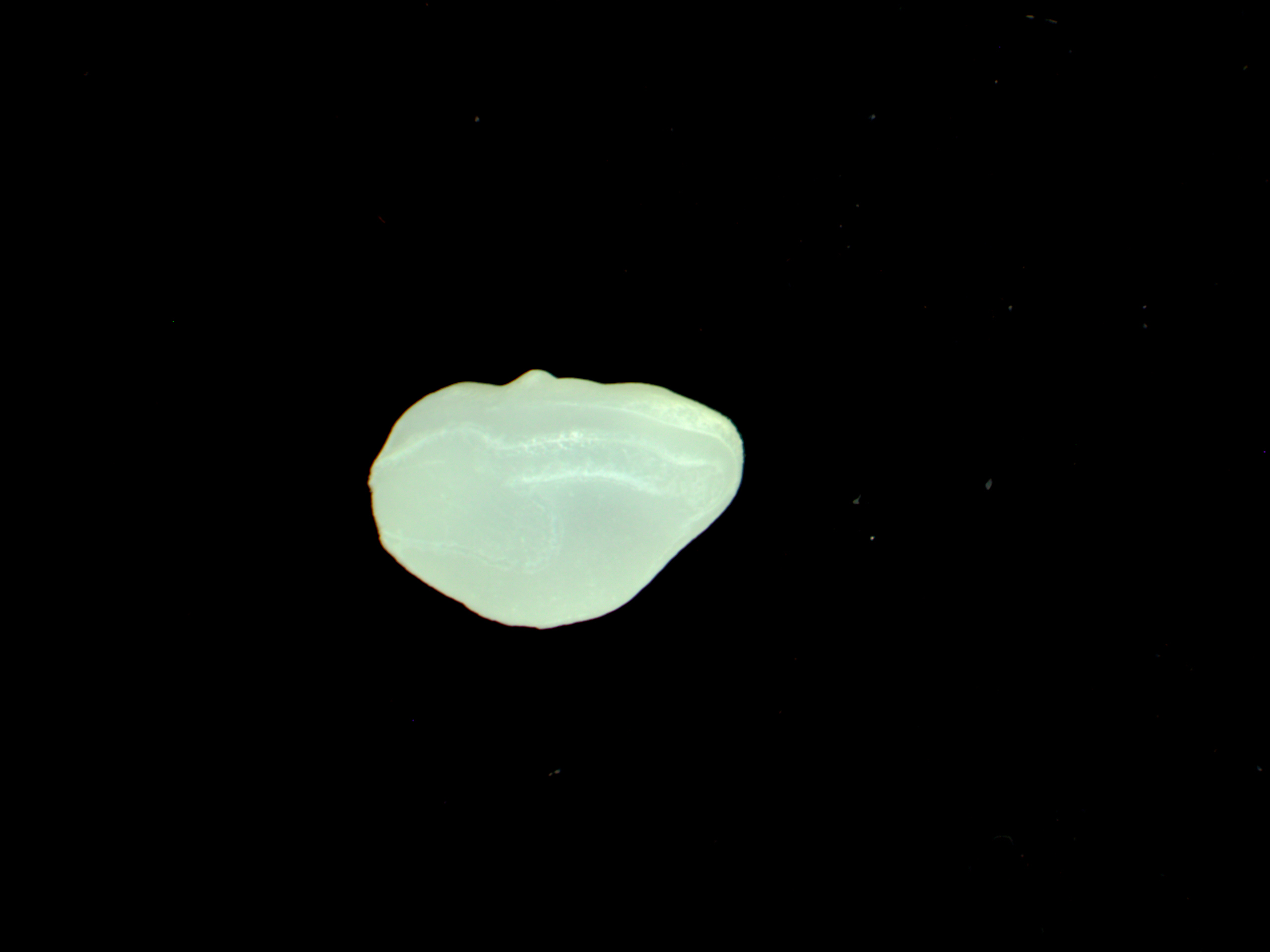

Supplement: Supplemental Information 14 [file peerj-04-1664-s014.zip › OtoRub/training/D98R1.jpg]

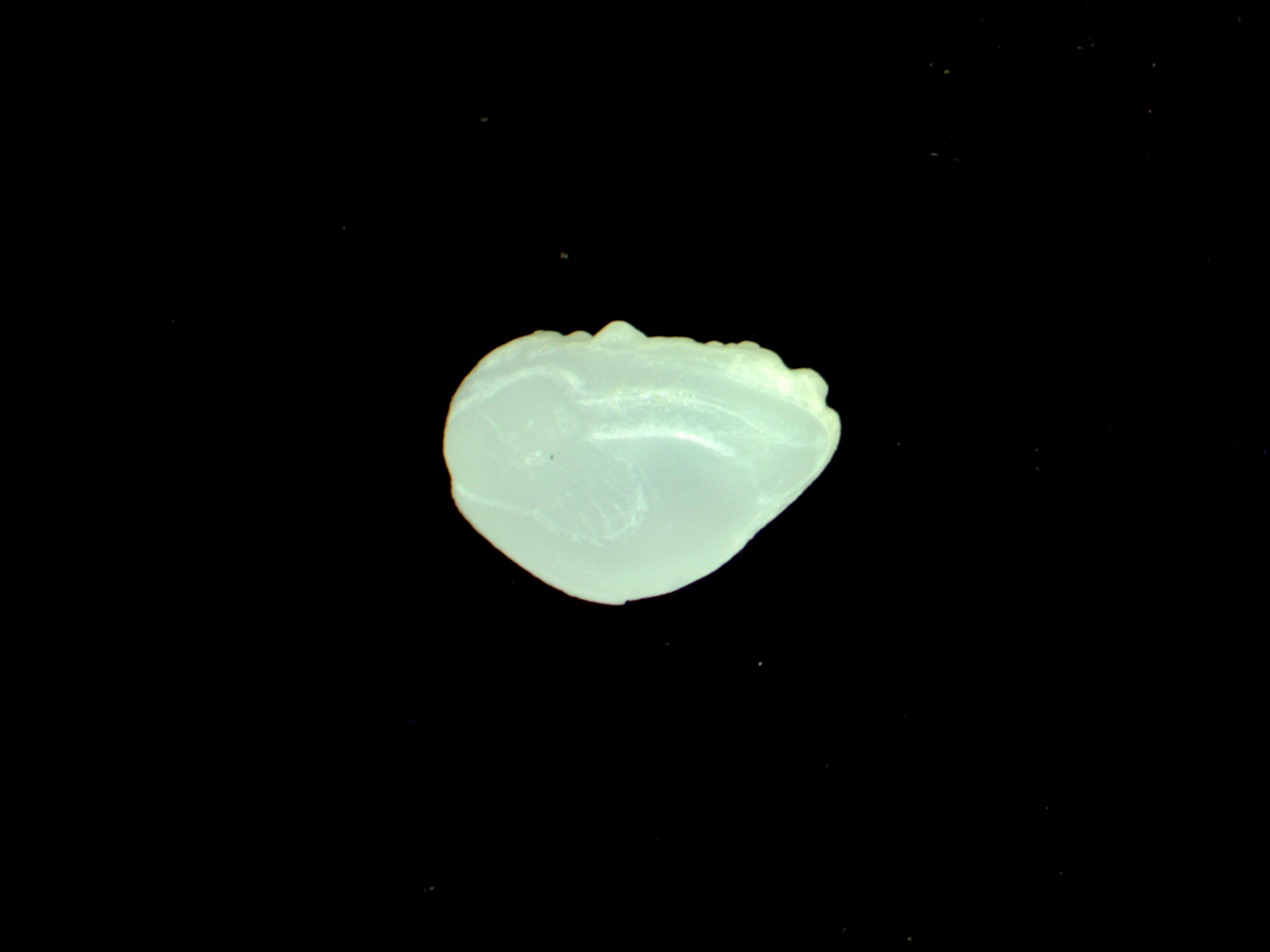

Supplement: Supplemental Information 14 [file peerj-04-1664-s014.zip › OtoRub/training/E87R1.jpg]

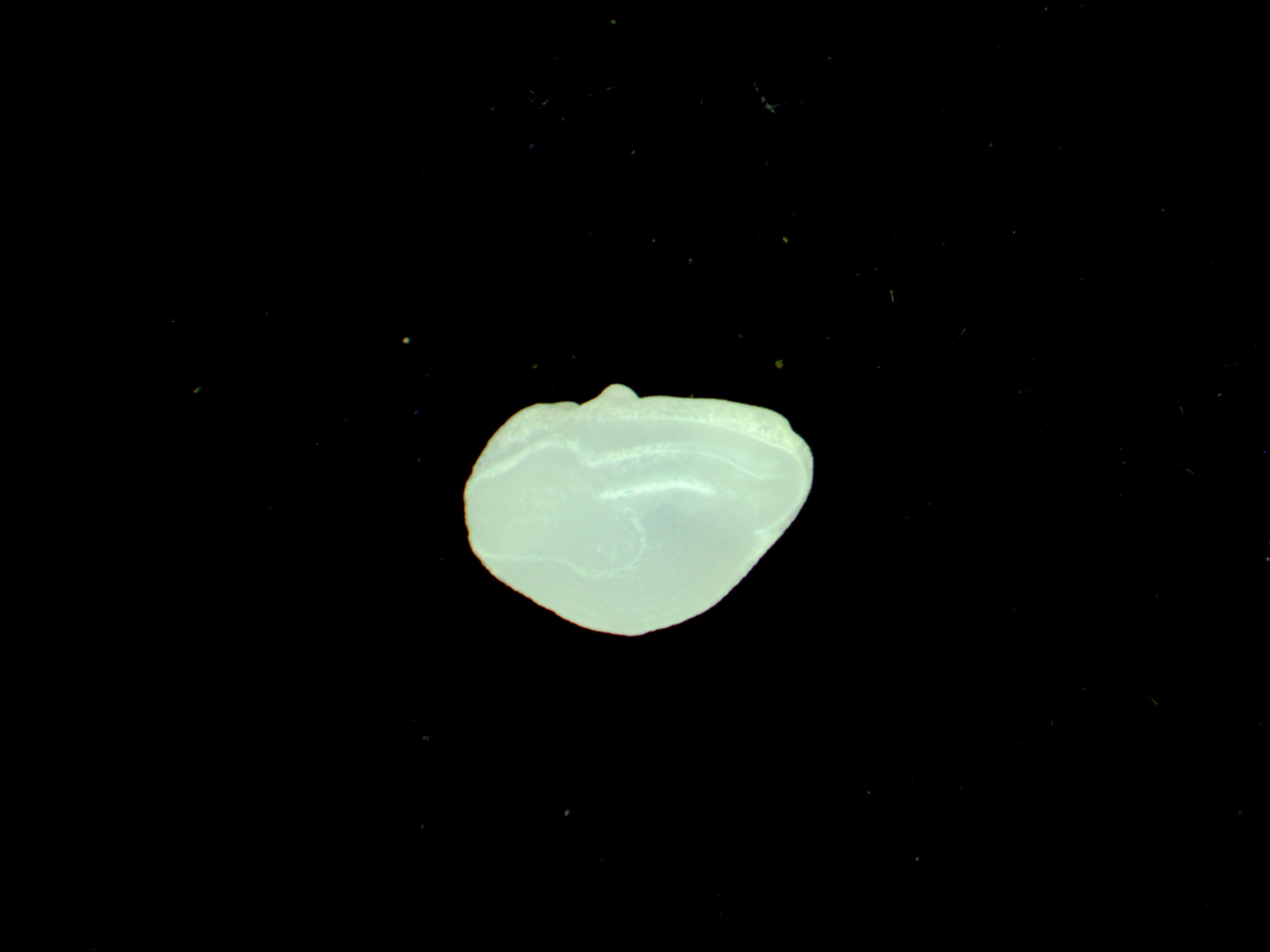

Supplement: Supplemental Information 14 [file peerj-04-1664-s014.zip › OtoRub/training/F47R1.jpg]

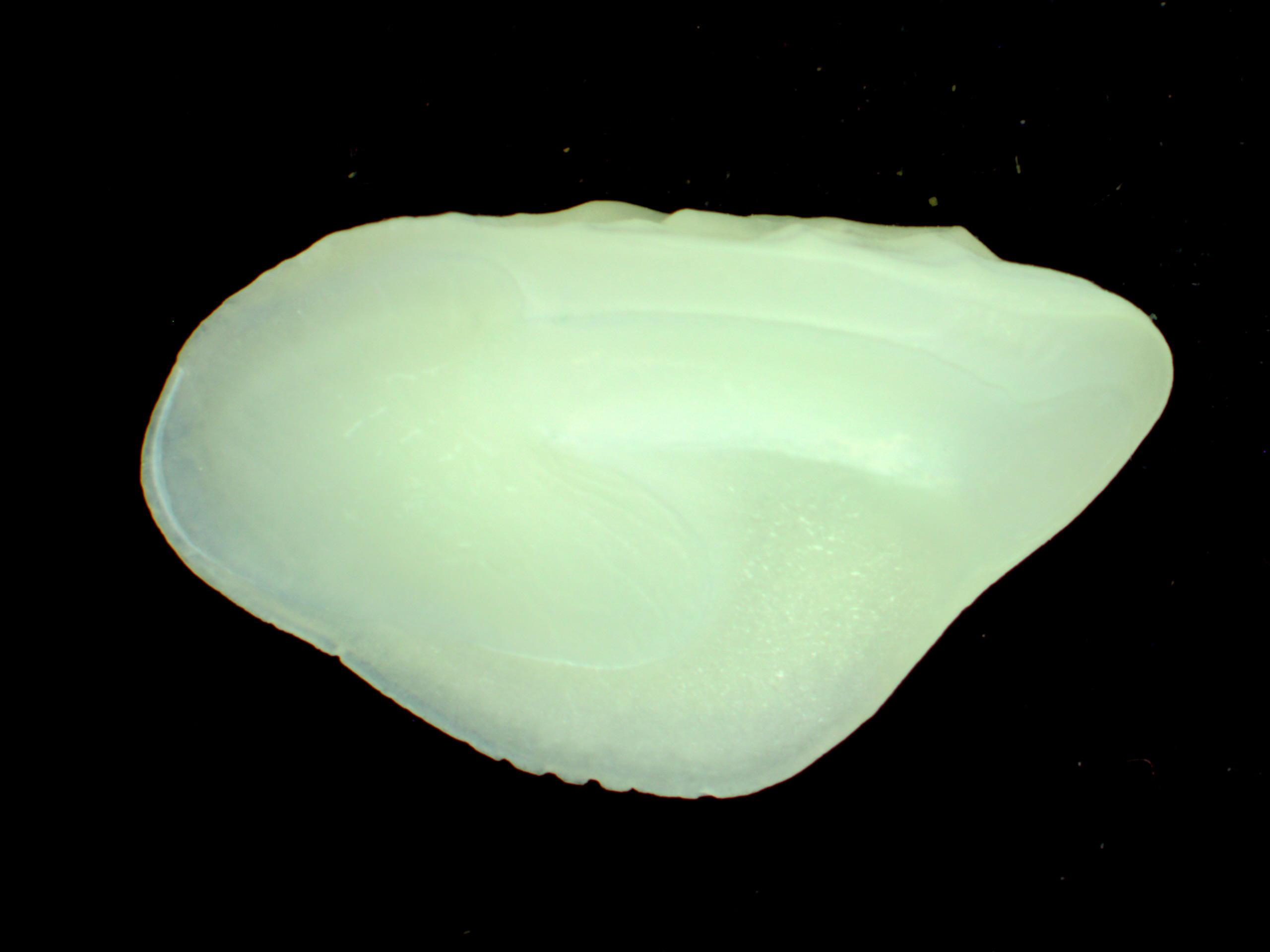

Supplement: Supplemental Information 14 [file peerj-04-1664-s014.zip › OtoRub/training/G66R1.jpg]

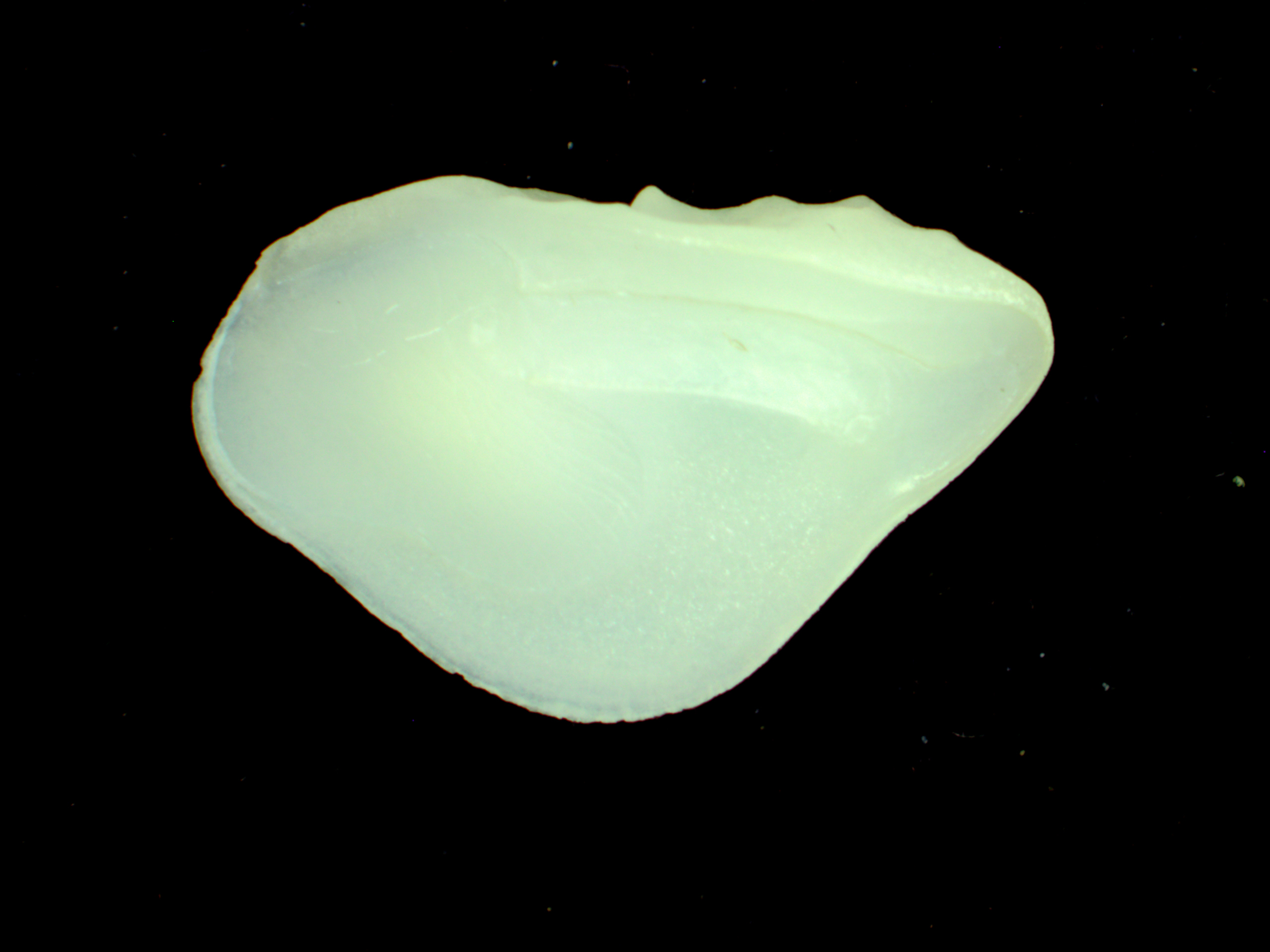

Supplement: Supplemental Information 14 [file peerj-04-1664-s014.zip › OtoRub/training/G68R1.jpg]

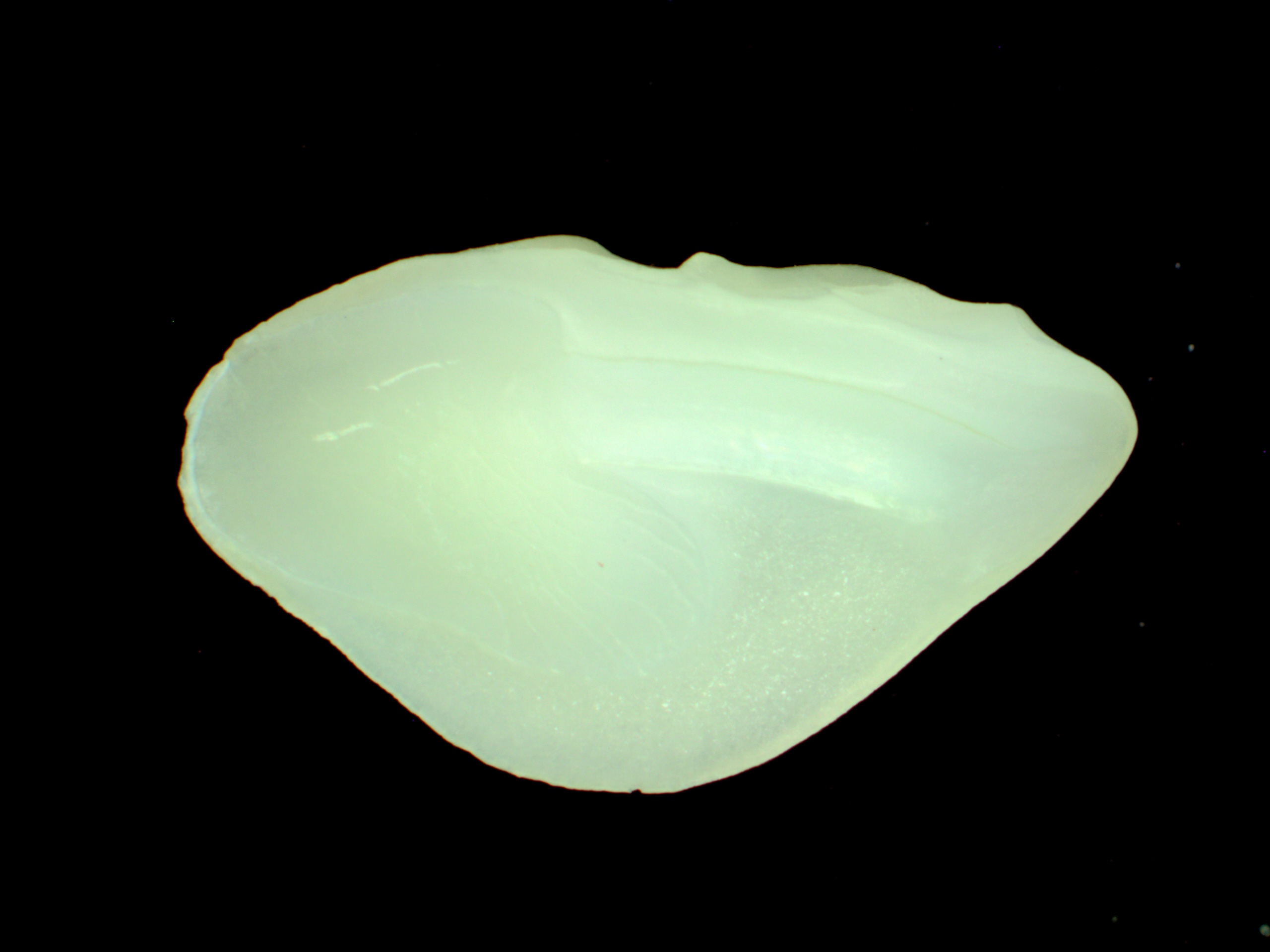

Supplement: Supplemental Information 14 [file peerj-04-1664-s014.zip › OtoRub/training/G96R1.jpg]

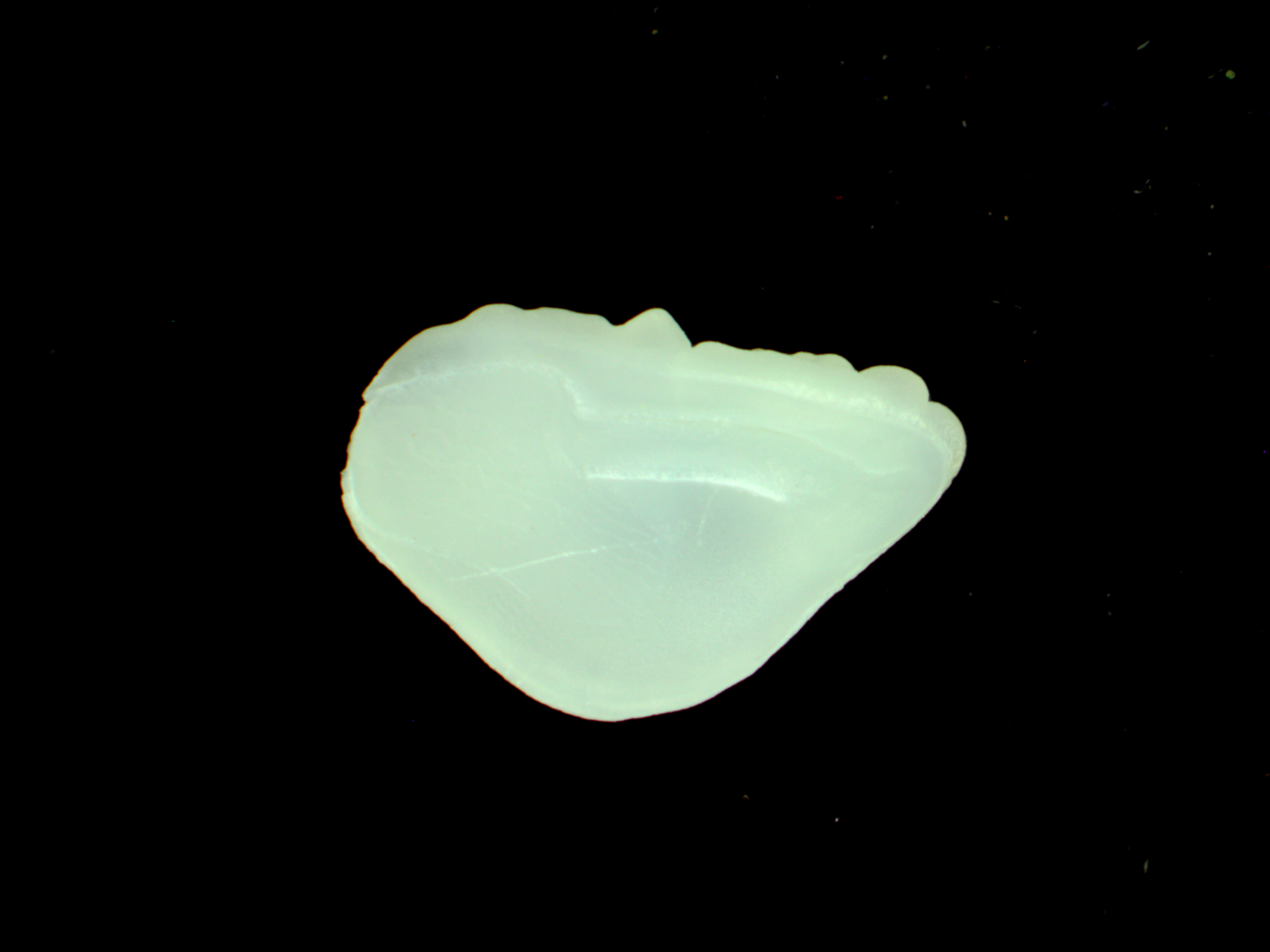

Supplement: Supplemental Information 14 [file peerj-04-1664-s014.zip › OtoRub/training/J23R1.jpg]

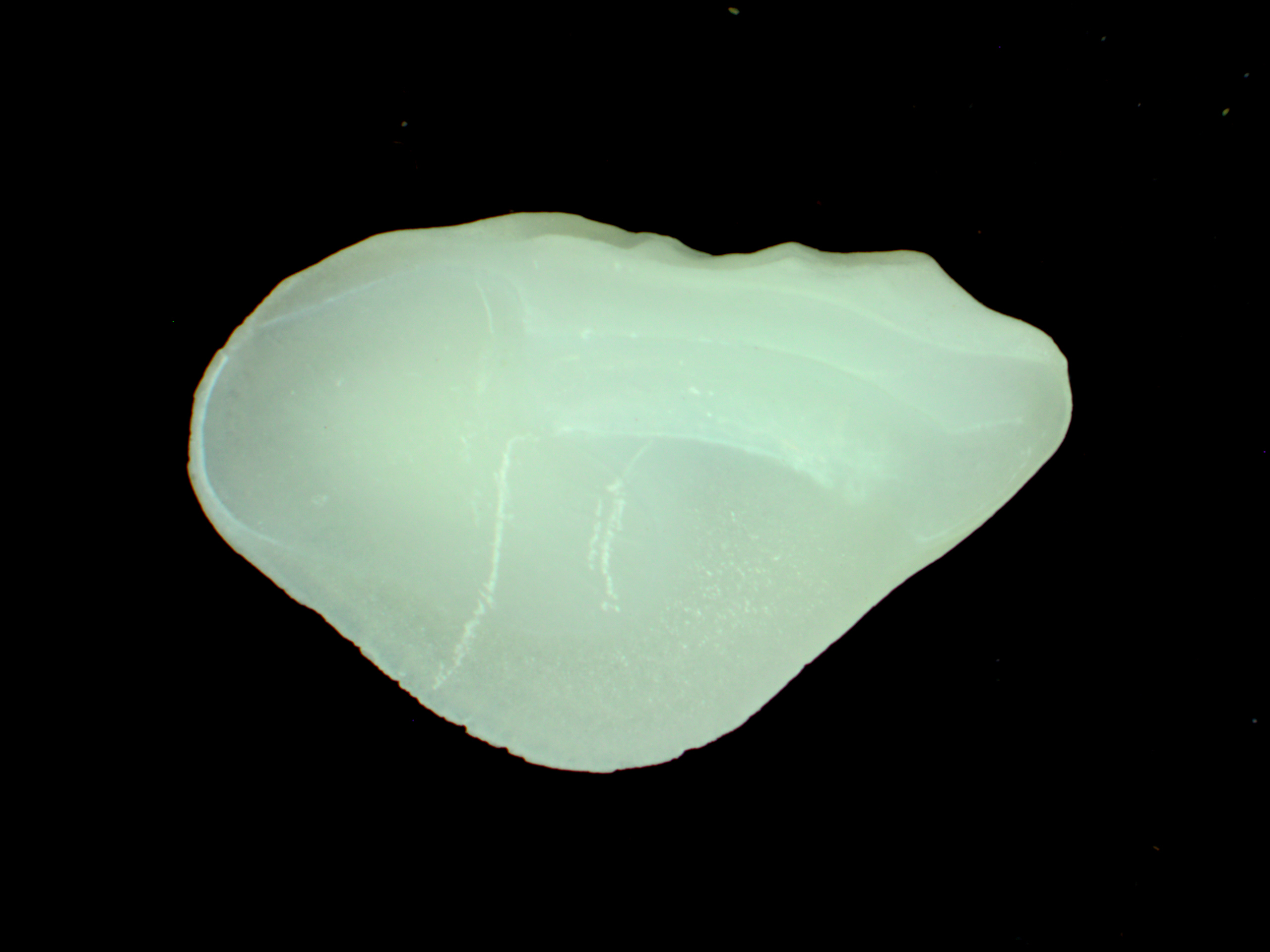

Supplement: Supplemental Information 14 [file peerj-04-1664-s014.zip › OtoRub/training/K54R1.jpg]

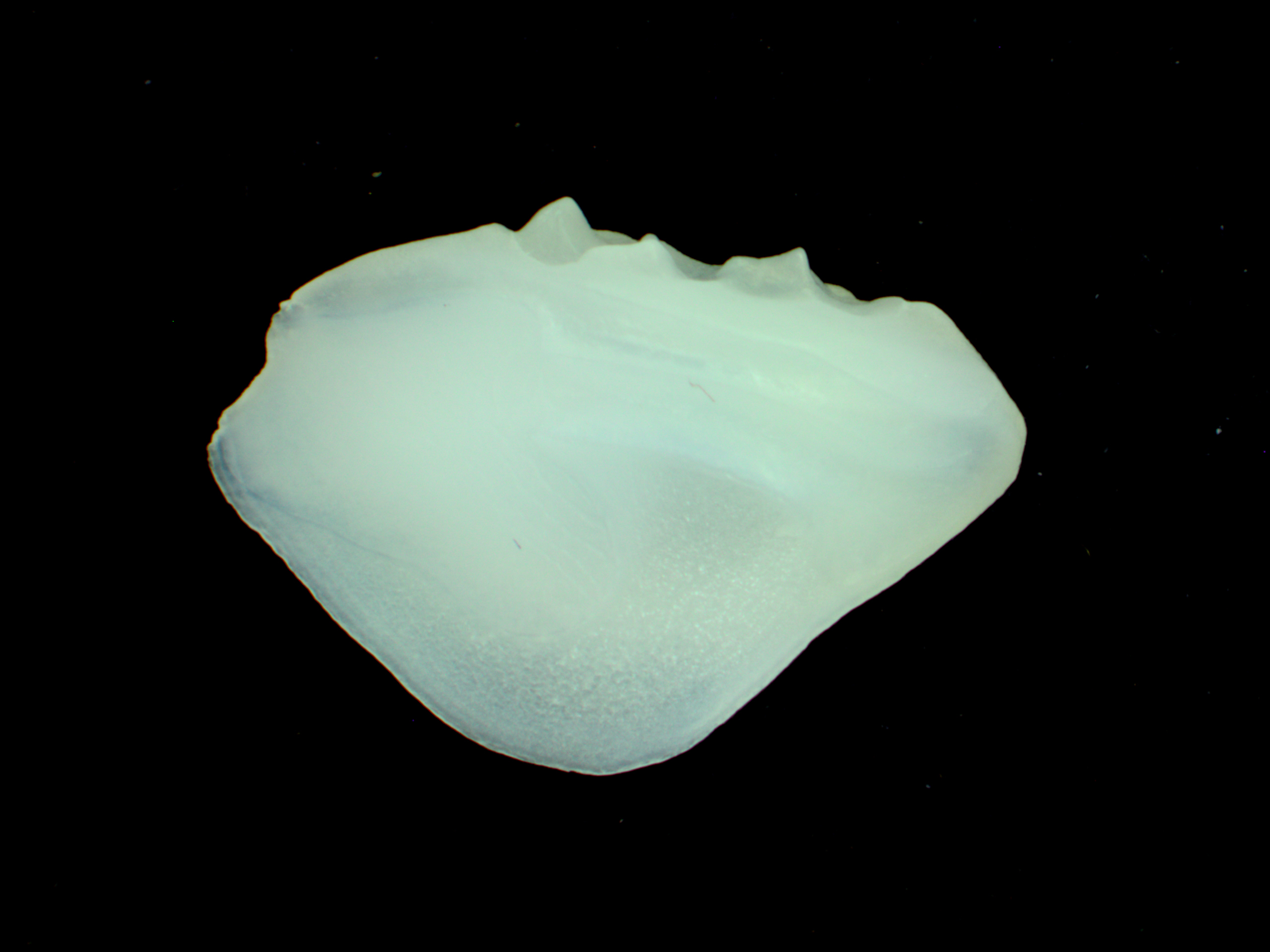

Supplement: Supplemental Information 14 [file peerj-04-1664-s014.zip › OtoRub/training/S11R1.jpg]

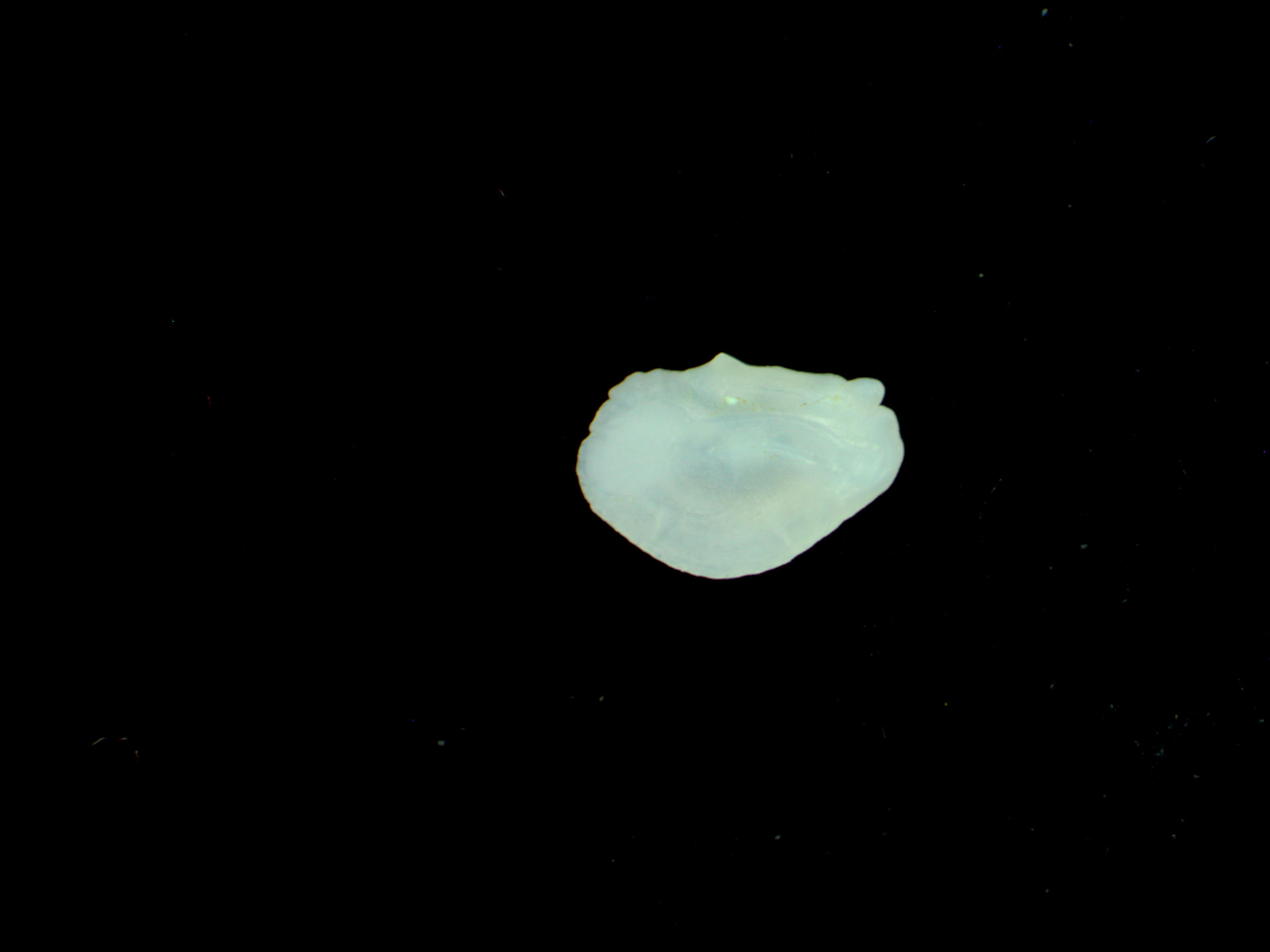

Supplement: Supplemental Information 14 [file peerj-04-1664-s014.zip › OtoRub/training/S12R1.jpg]

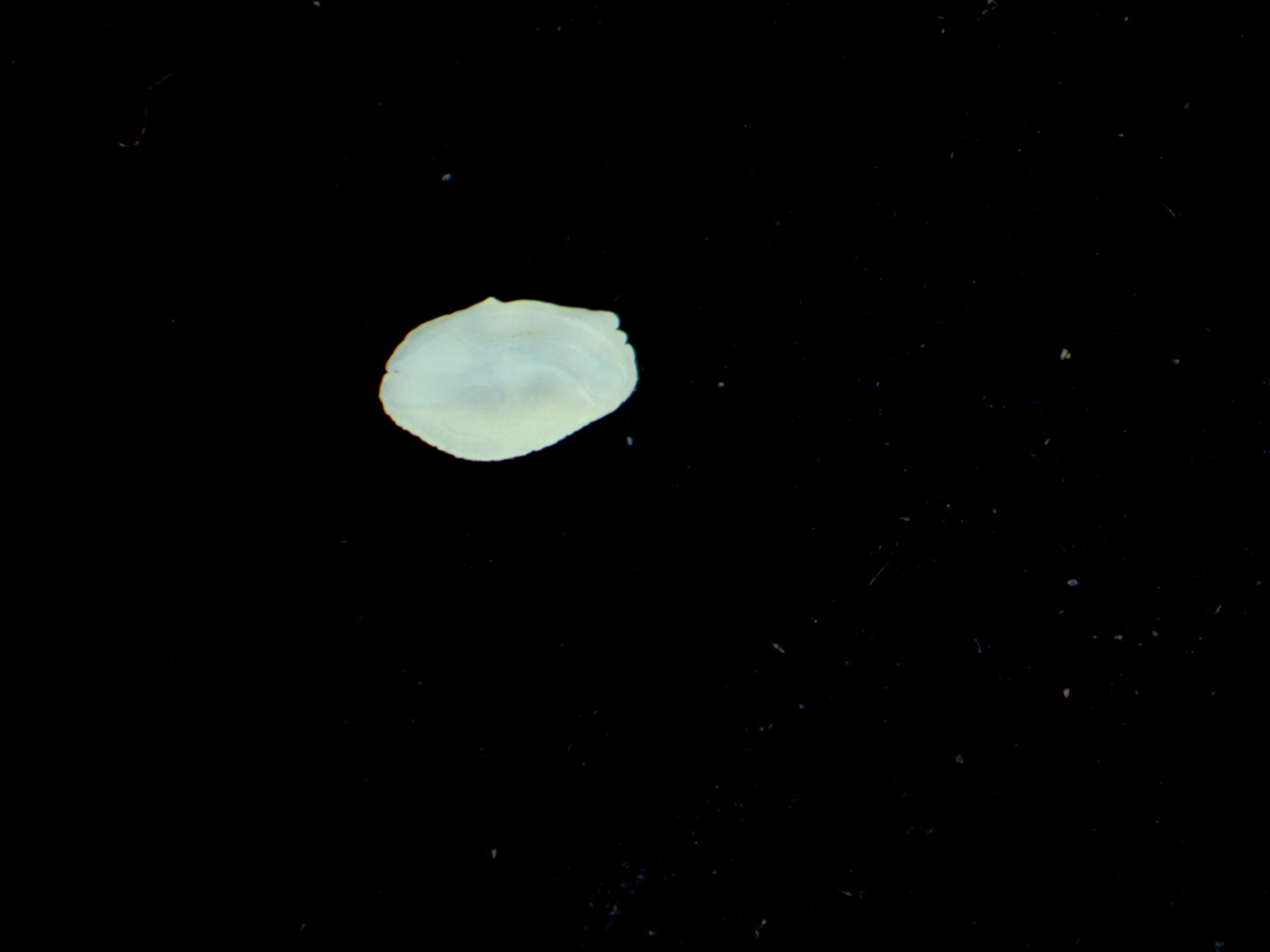

Supplement: Supplemental Information 14 [file peerj-04-1664-s014.zip › OtoRub/training/S15R1.jpg]

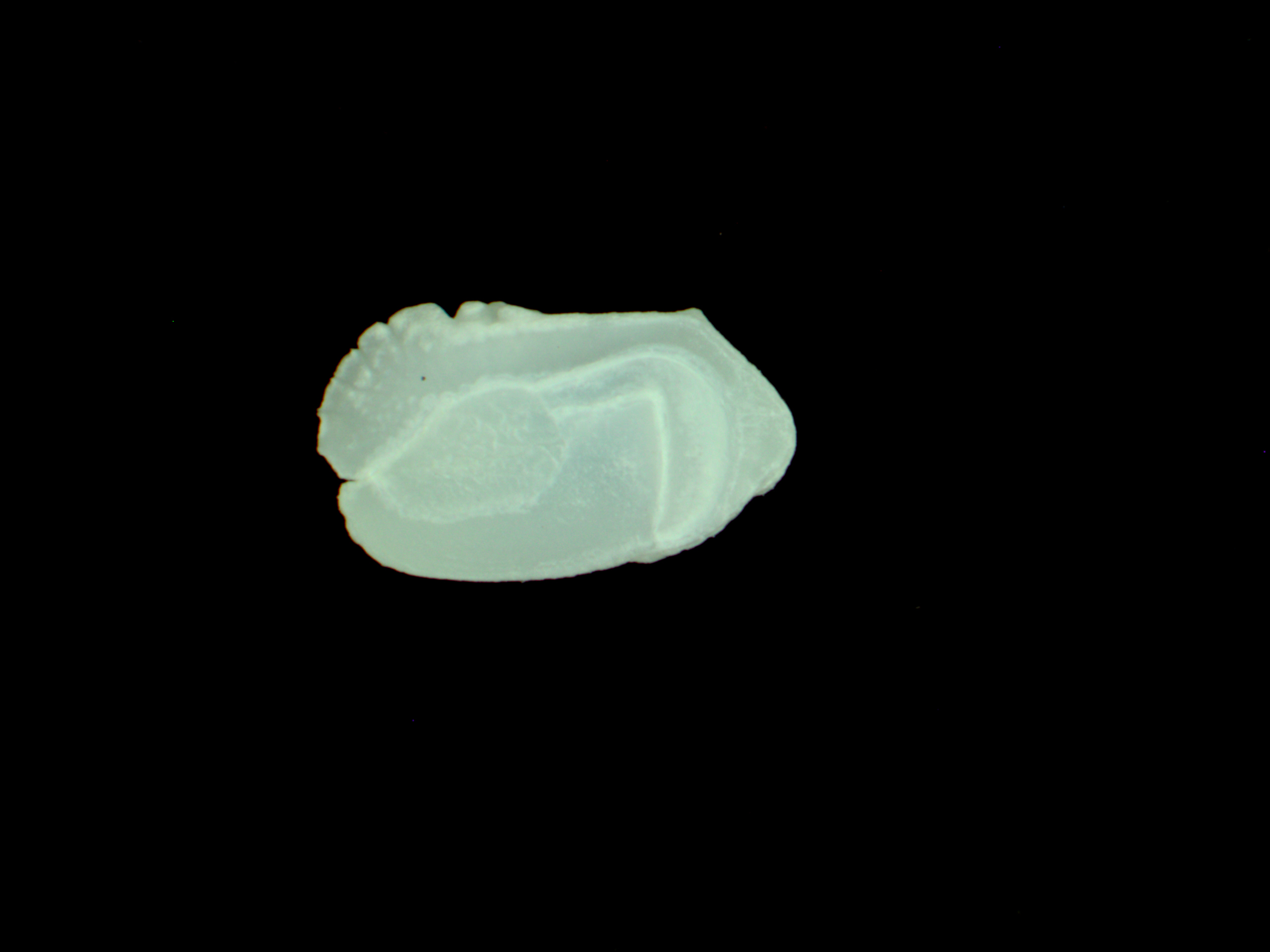

Supplement: Supplemental Information 15 [file peerj-04-1664-s015.zip › PanMic/testing/F85R1.jpg]

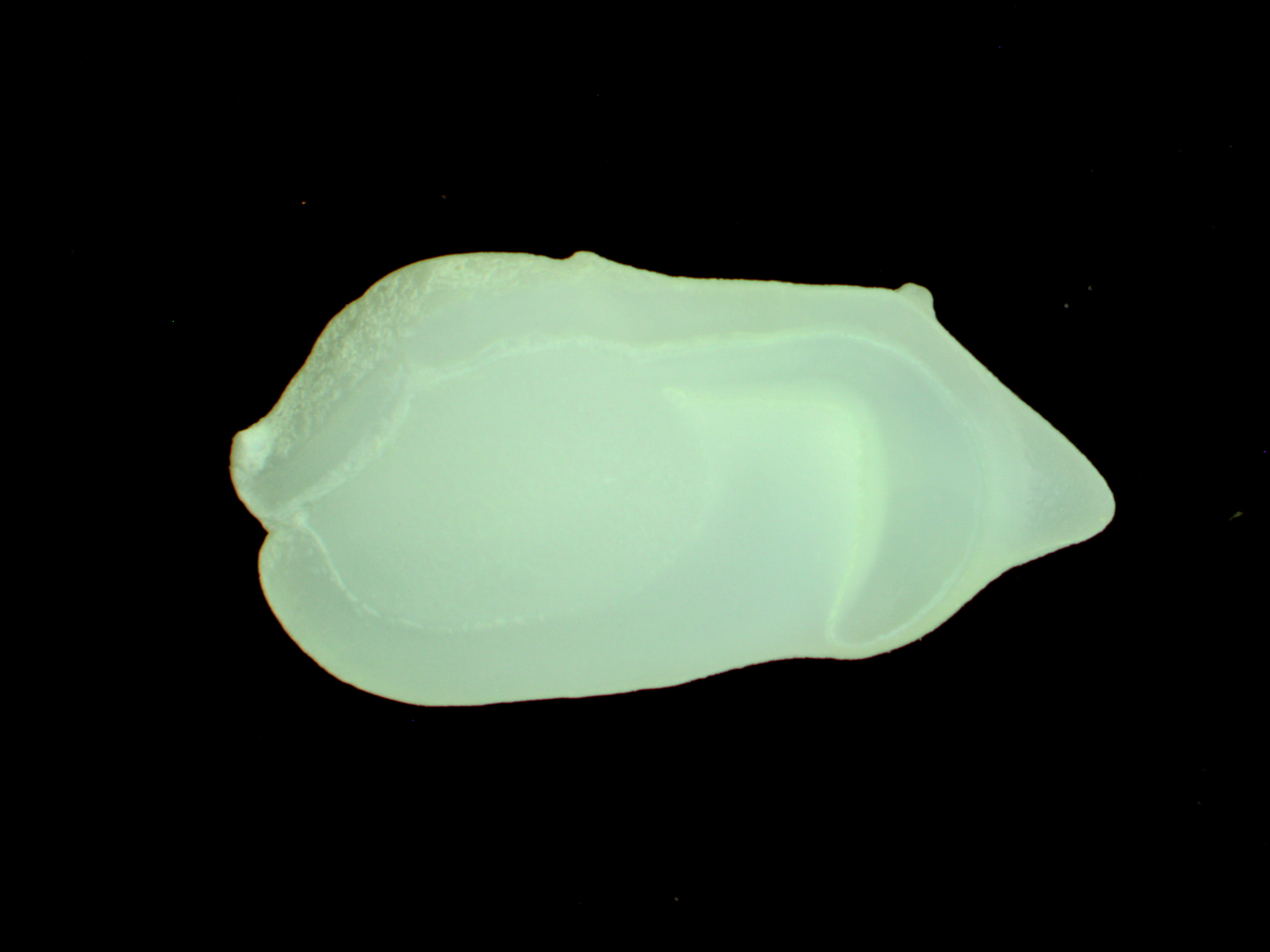

Supplement: Supplemental Information 15 [file peerj-04-1664-s015.zip › PanMic/testing/F89R1.jpg]

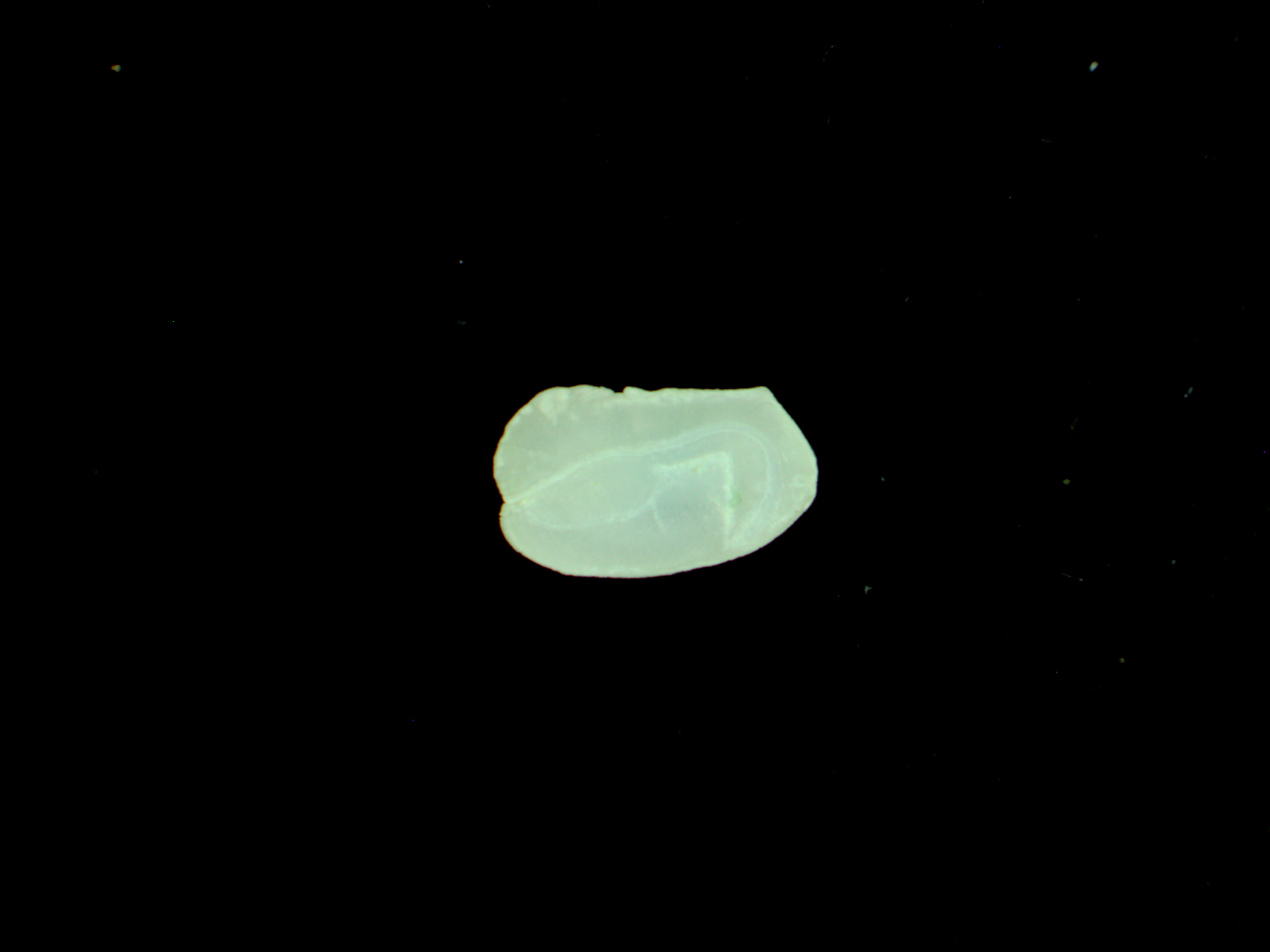

Supplement: Supplemental Information 15 [file peerj-04-1664-s015.zip › PanMic/testing/N51R1.jpg]

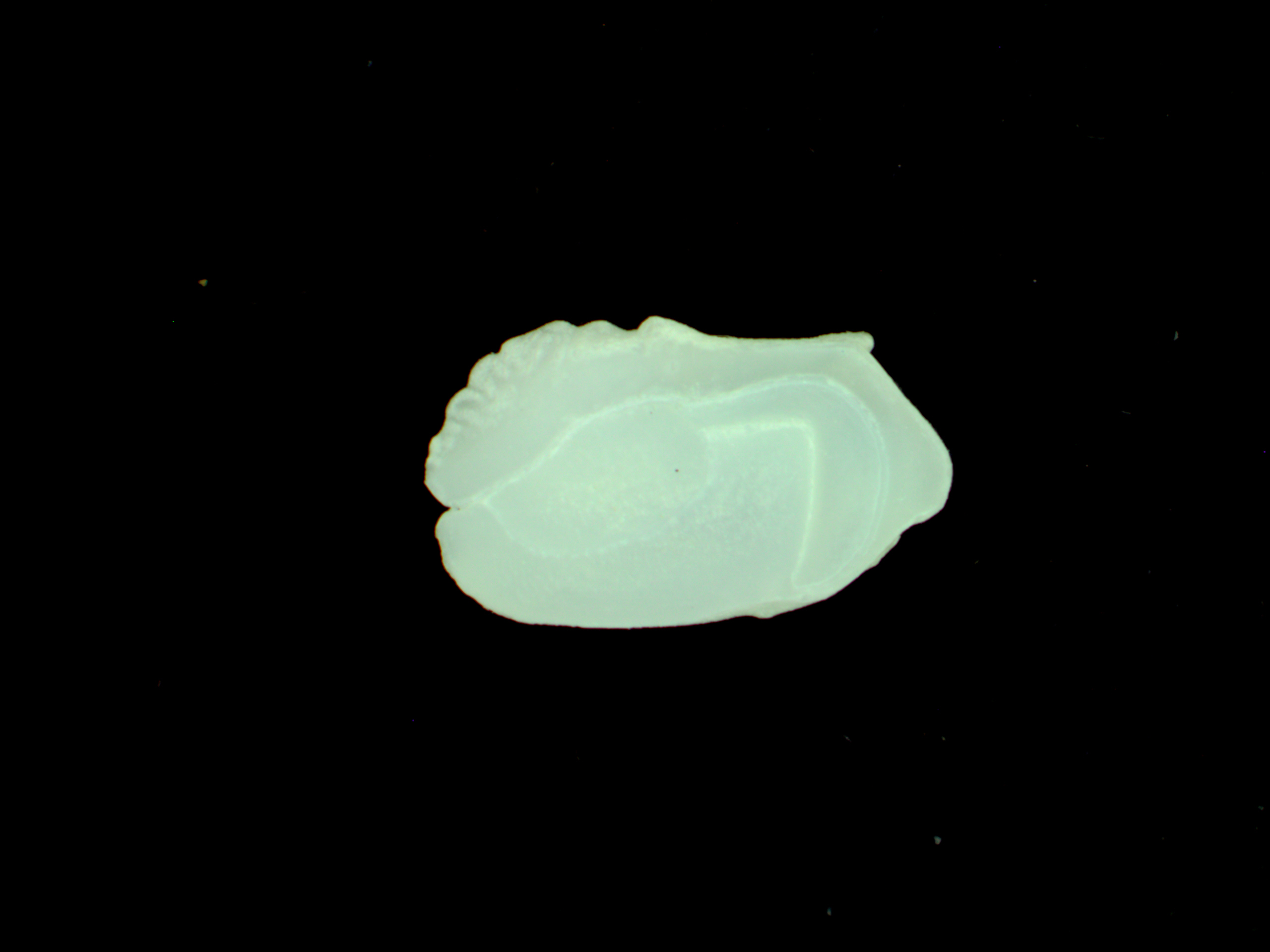

Supplement: Supplemental Information 15 [file peerj-04-1664-s015.zip › PanMic/testing/N75R1.jpg]

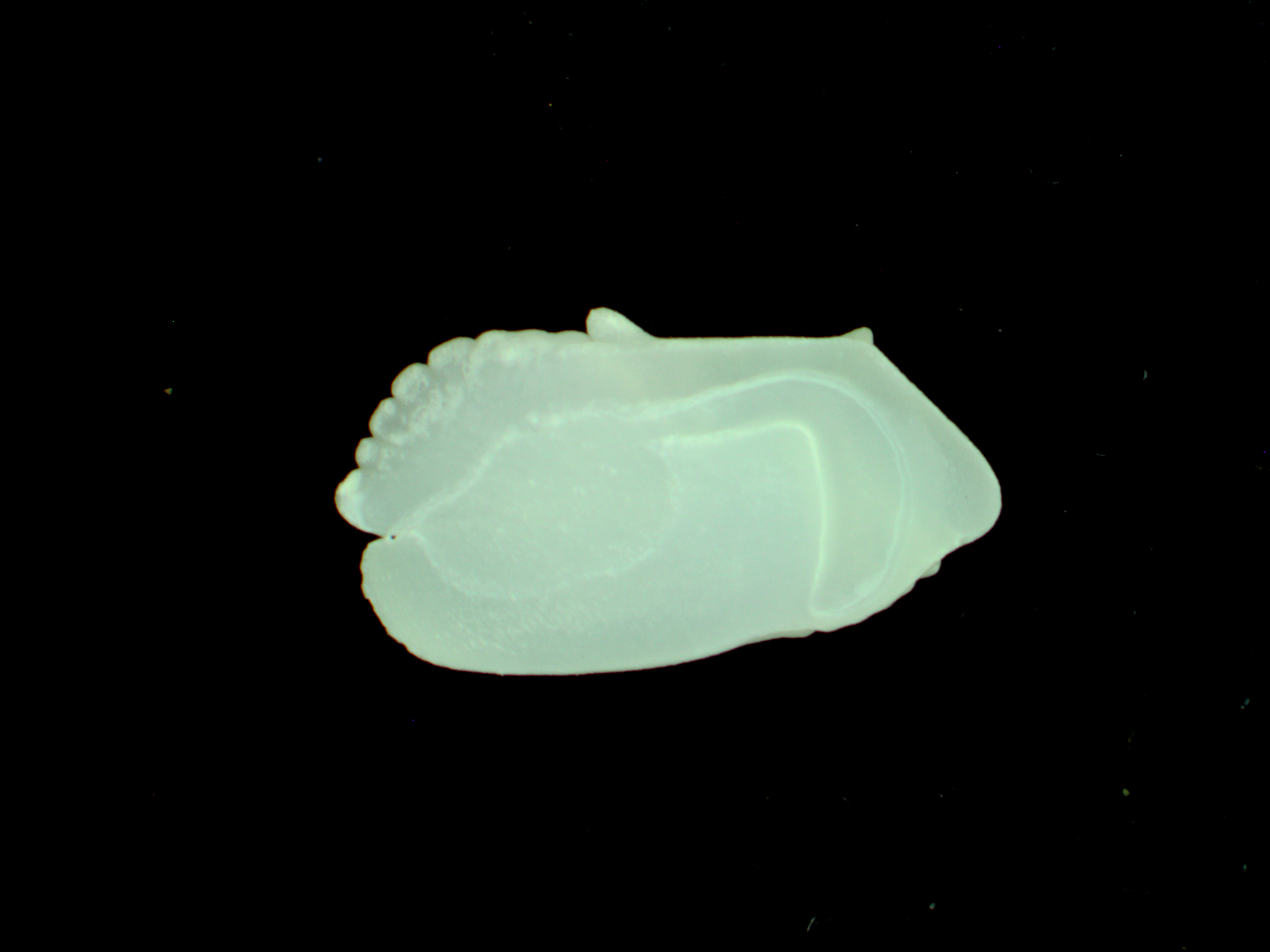

Supplement: Supplemental Information 15 [file peerj-04-1664-s015.zip › PanMic/testing/O81R1.jpg]

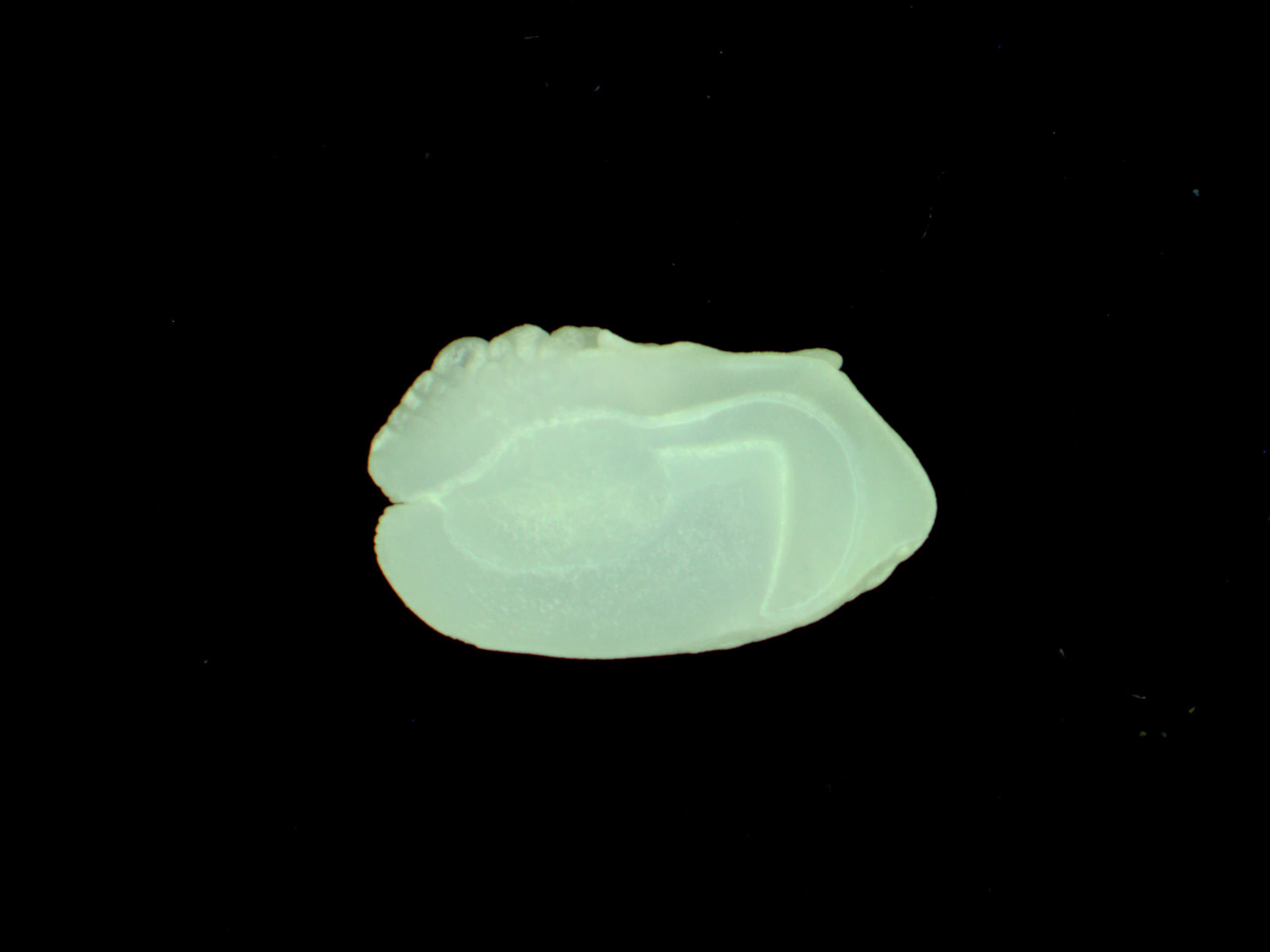

Supplement: Supplemental Information 15 [file peerj-04-1664-s015.zip › PanMic/testing/P06R1.jpg]

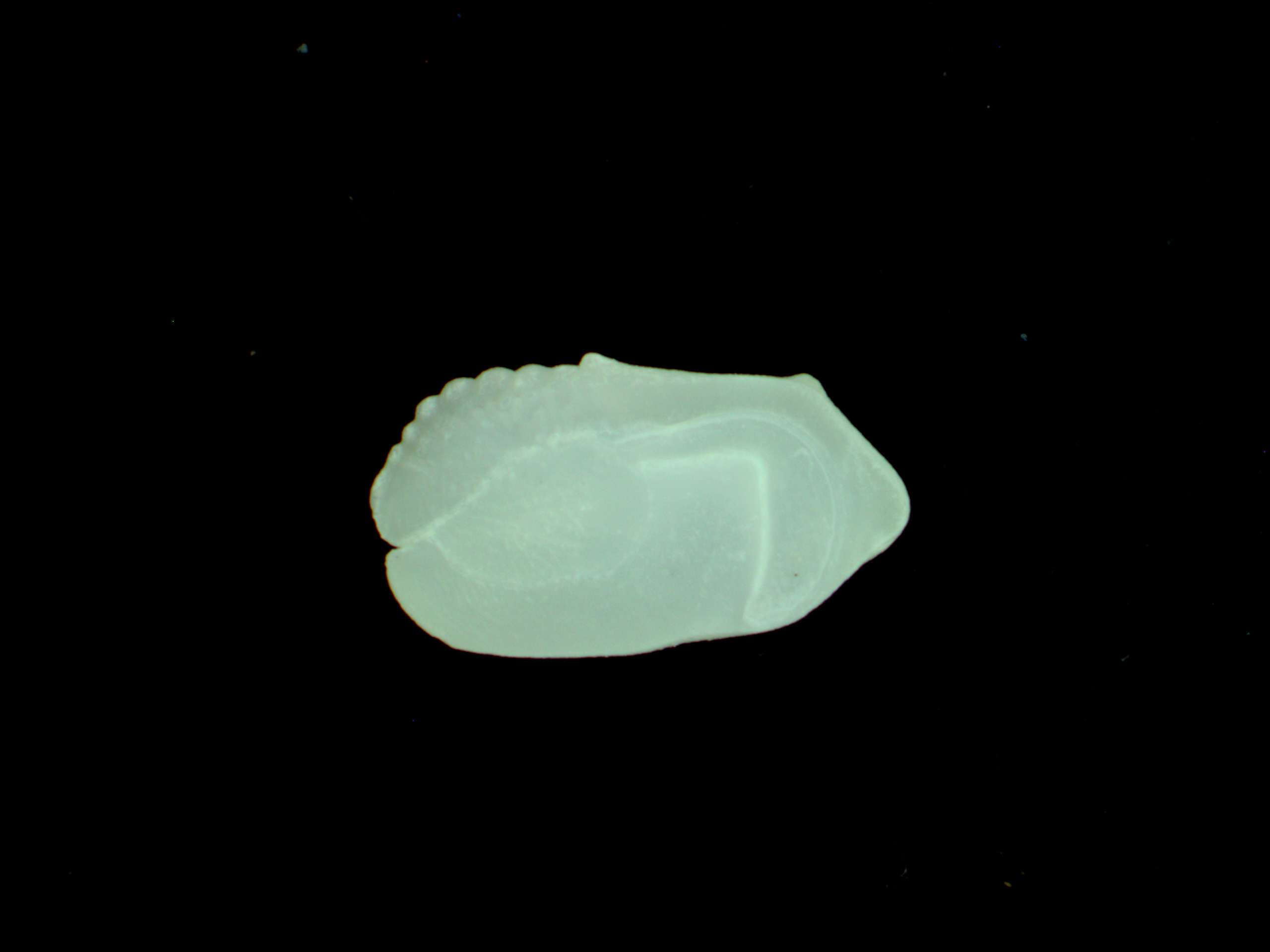

Supplement: Supplemental Information 15 [file peerj-04-1664-s015.zip › PanMic/testing/P13R1.jpg]

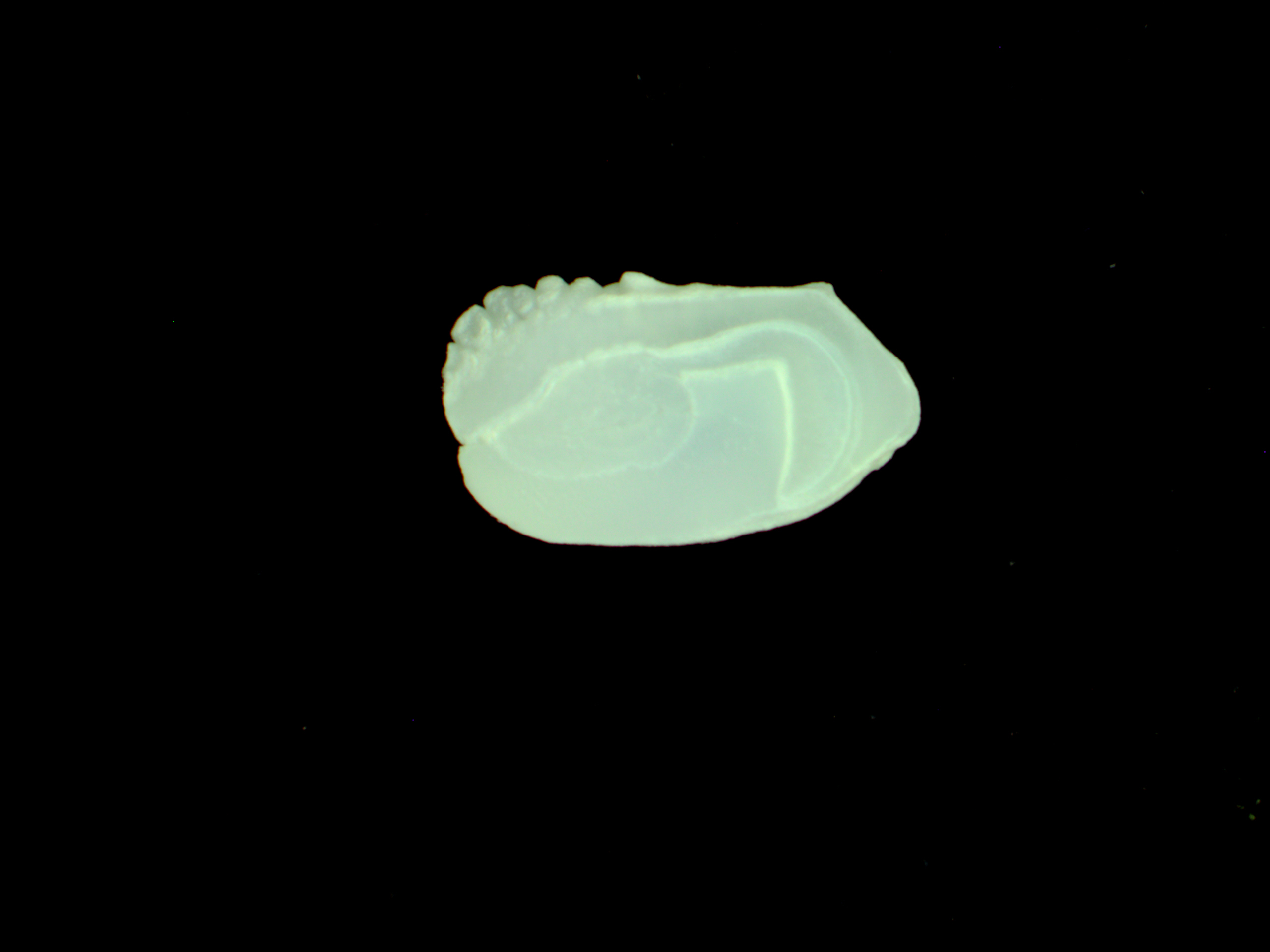

Supplement: Supplemental Information 15 [file peerj-04-1664-s015.zip › PanMic/testing/P62R1.jpg]

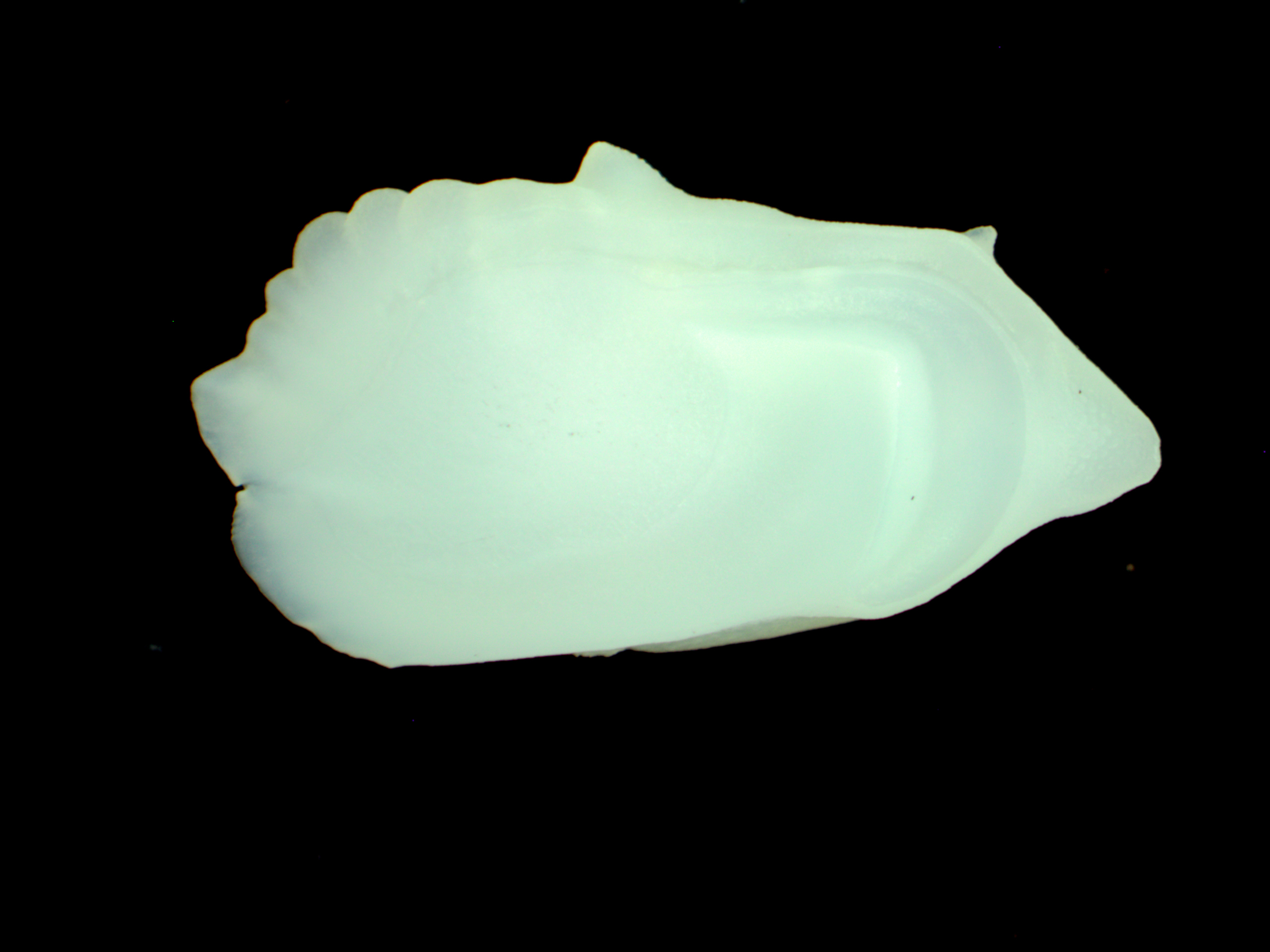

Supplement: Supplemental Information 15 [file peerj-04-1664-s015.zip › PanMic/testing/S29R1.jpg]

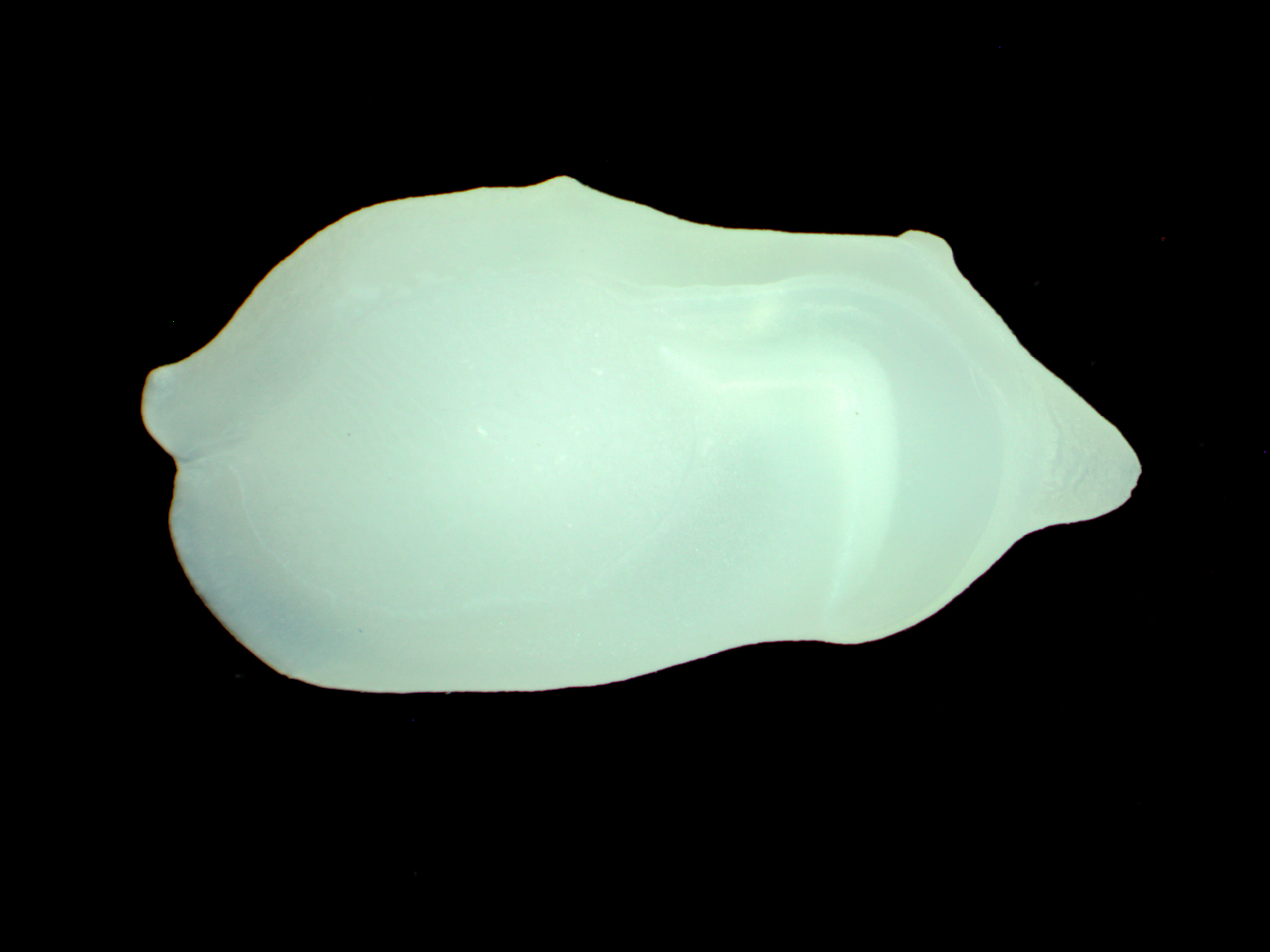

Supplement: Supplemental Information 15 [file peerj-04-1664-s015.zip › PanMic/testing/S41R1.jpg]

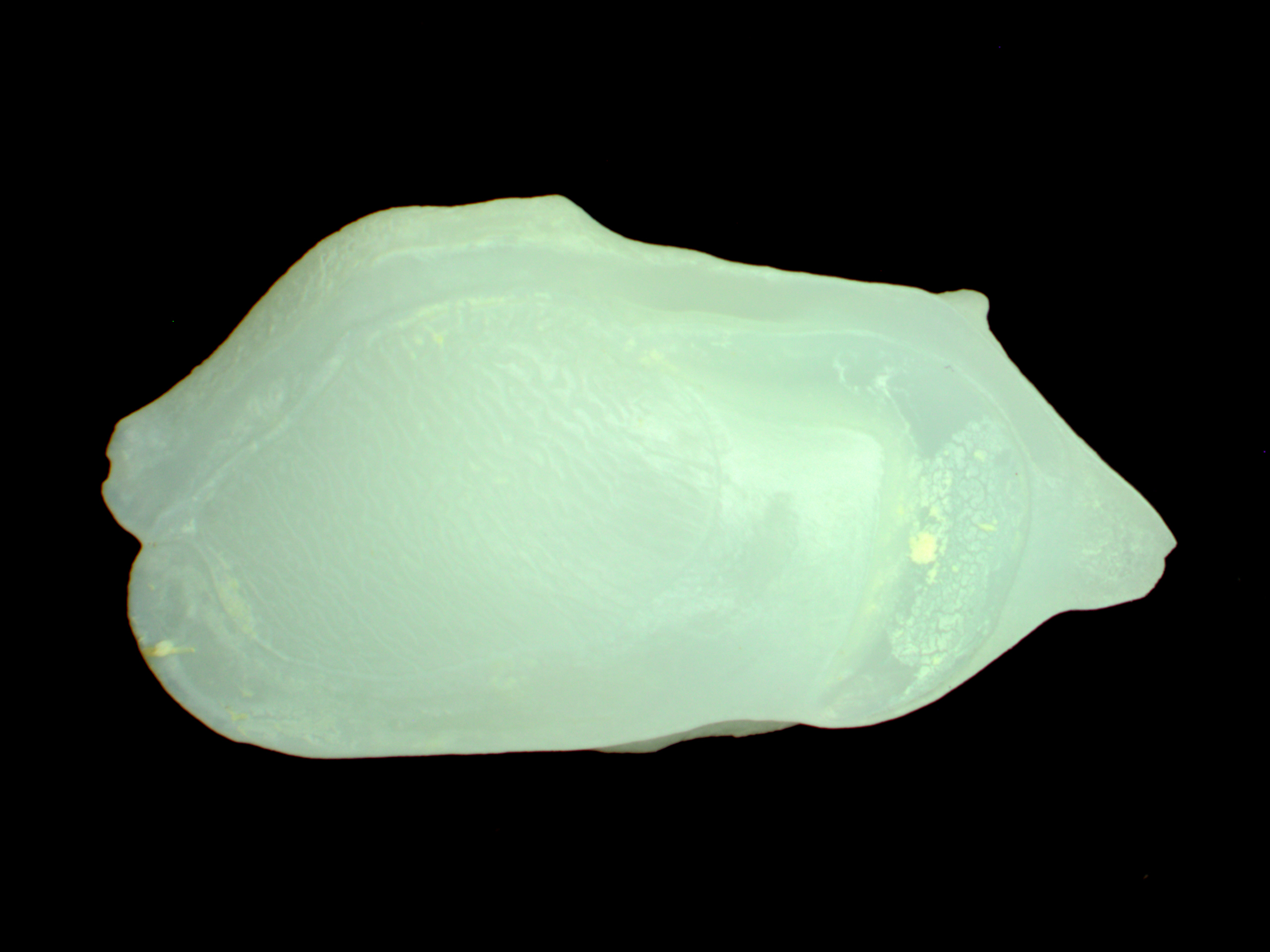

Supplement: Supplemental Information 15 [file peerj-04-1664-s015.zip › PanMic/training/50R1.jpg]

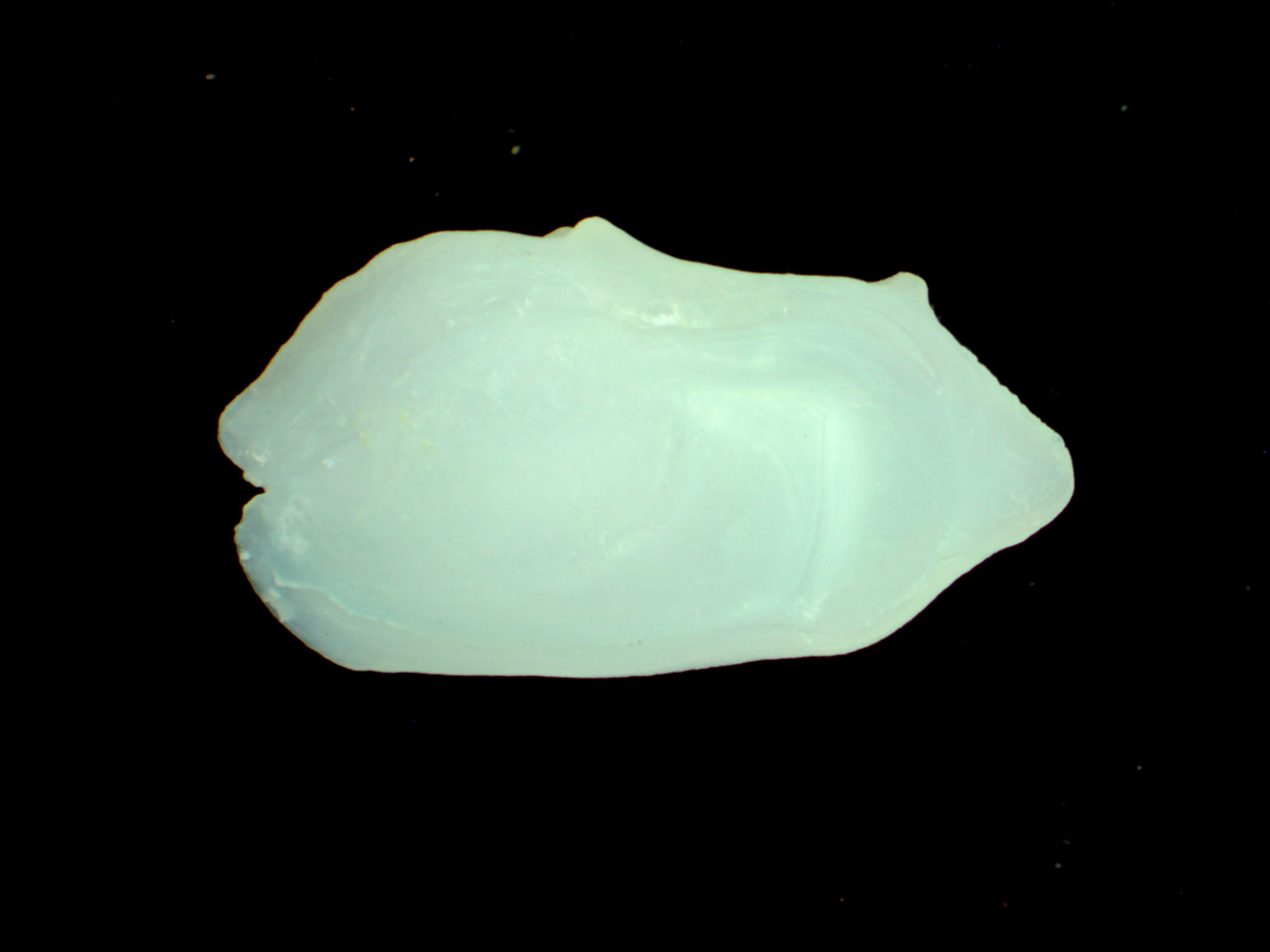

Supplement: Supplemental Information 15 [file peerj-04-1664-s015.zip › PanMic/training/515R1.jpg]

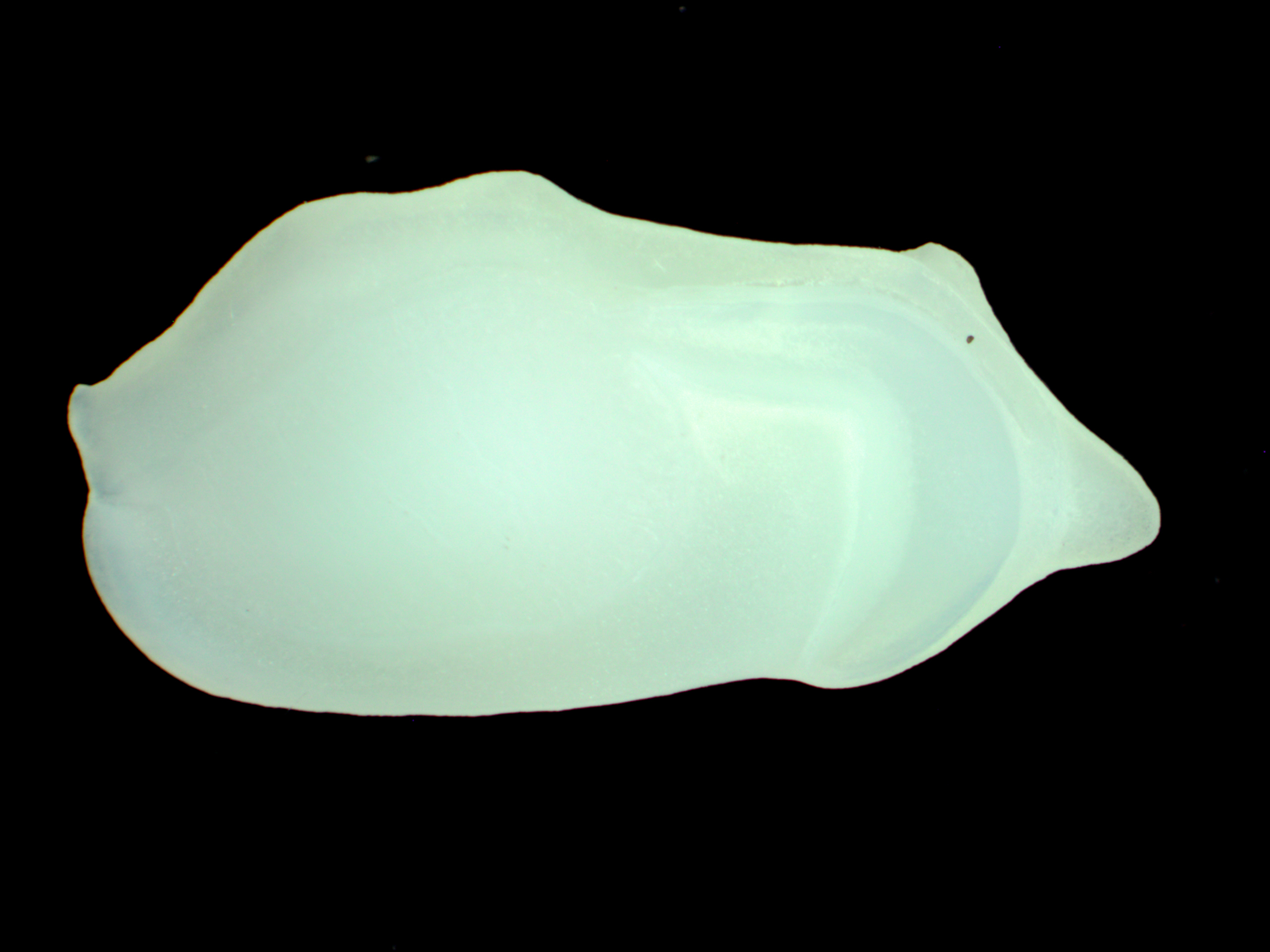

Supplement: Supplemental Information 15 [file peerj-04-1664-s015.zip › PanMic/training/516R1.jpg]

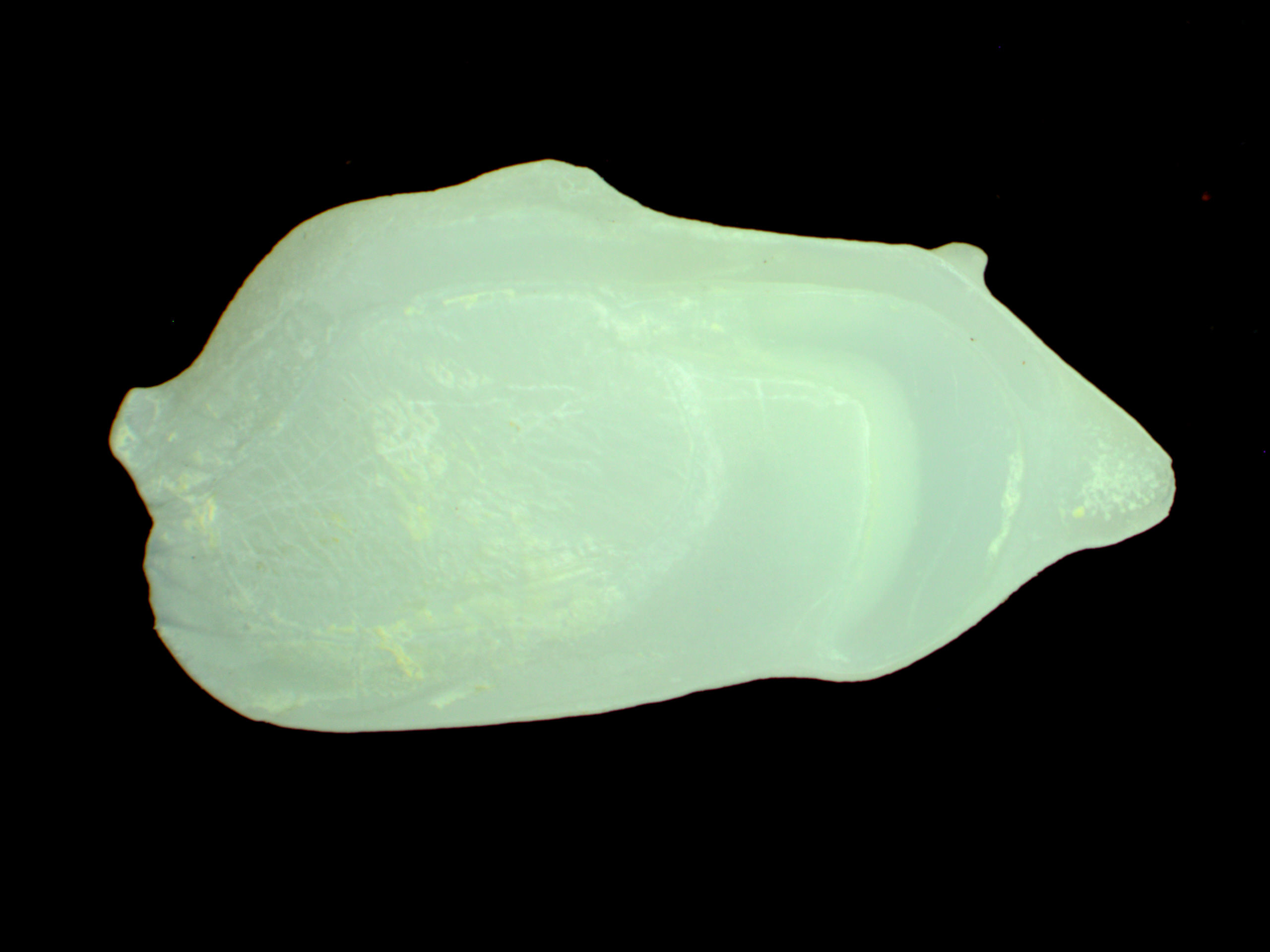

Supplement: Supplemental Information 15 [file peerj-04-1664-s015.zip › PanMic/training/51R1.jpg]

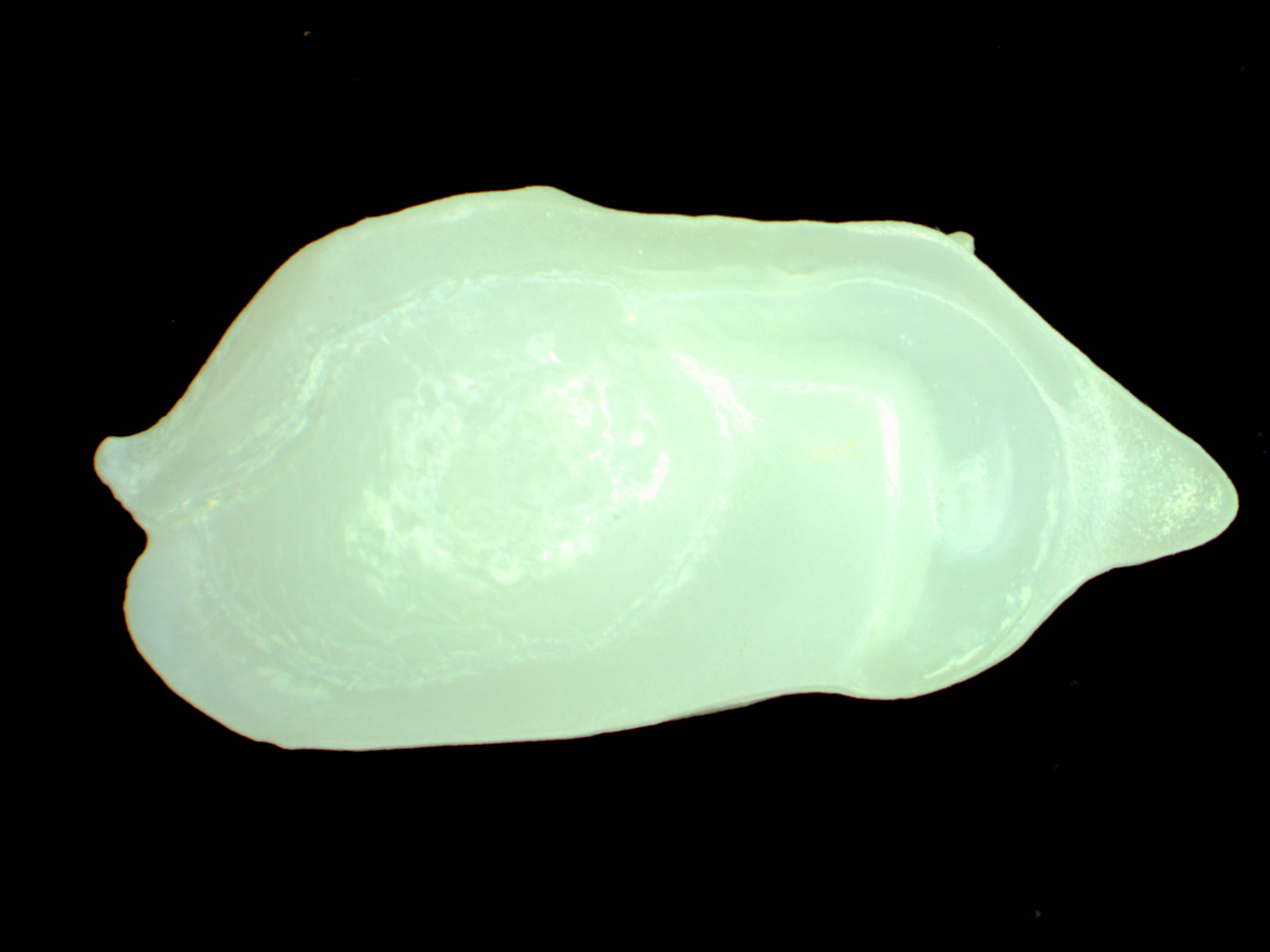

Supplement: Supplemental Information 15 [file peerj-04-1664-s015.zip › PanMic/training/52R1.jpg]

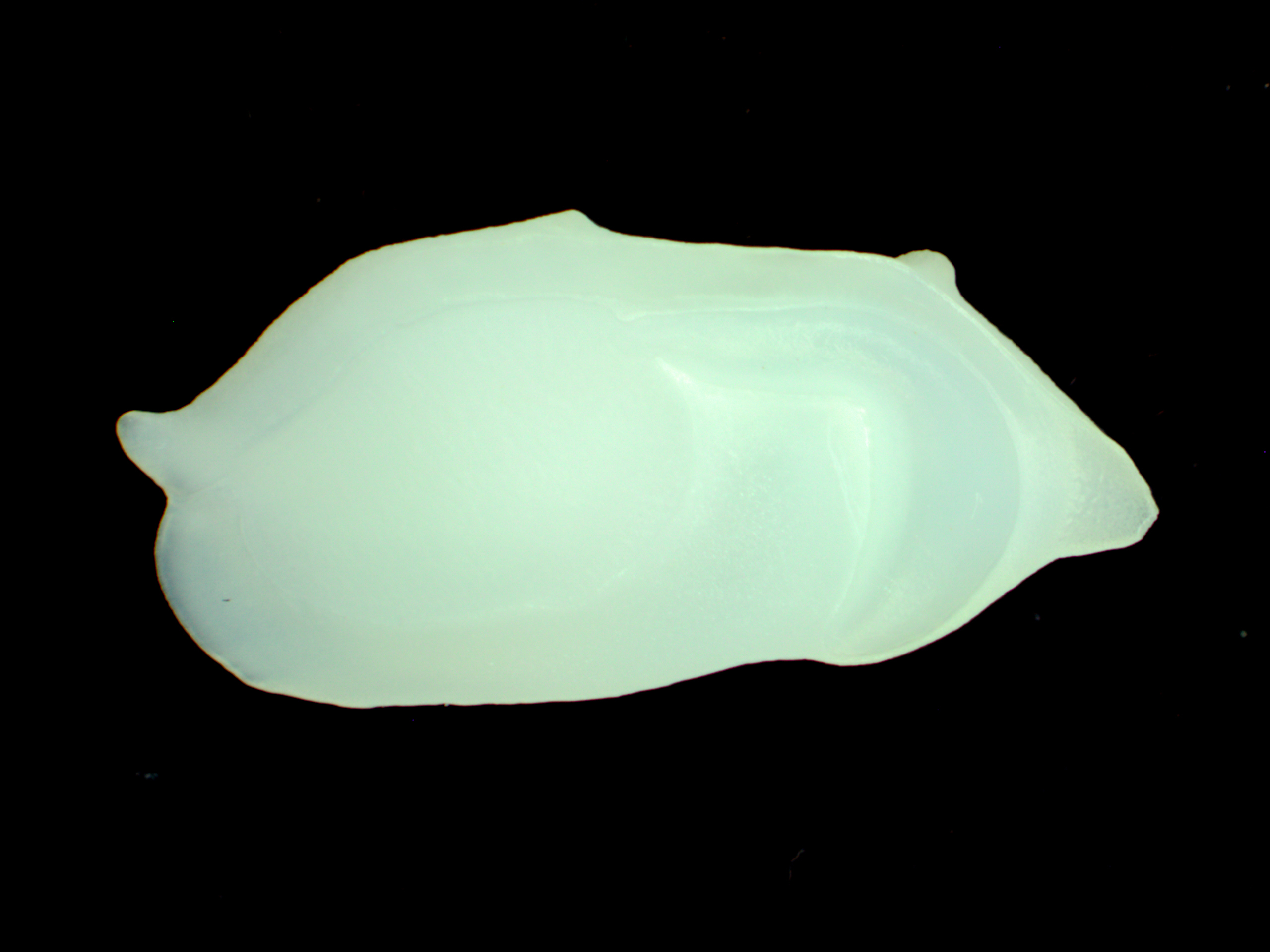

Supplement: Supplemental Information 15 [file peerj-04-1664-s015.zip › PanMic/training/53R1.jpg]

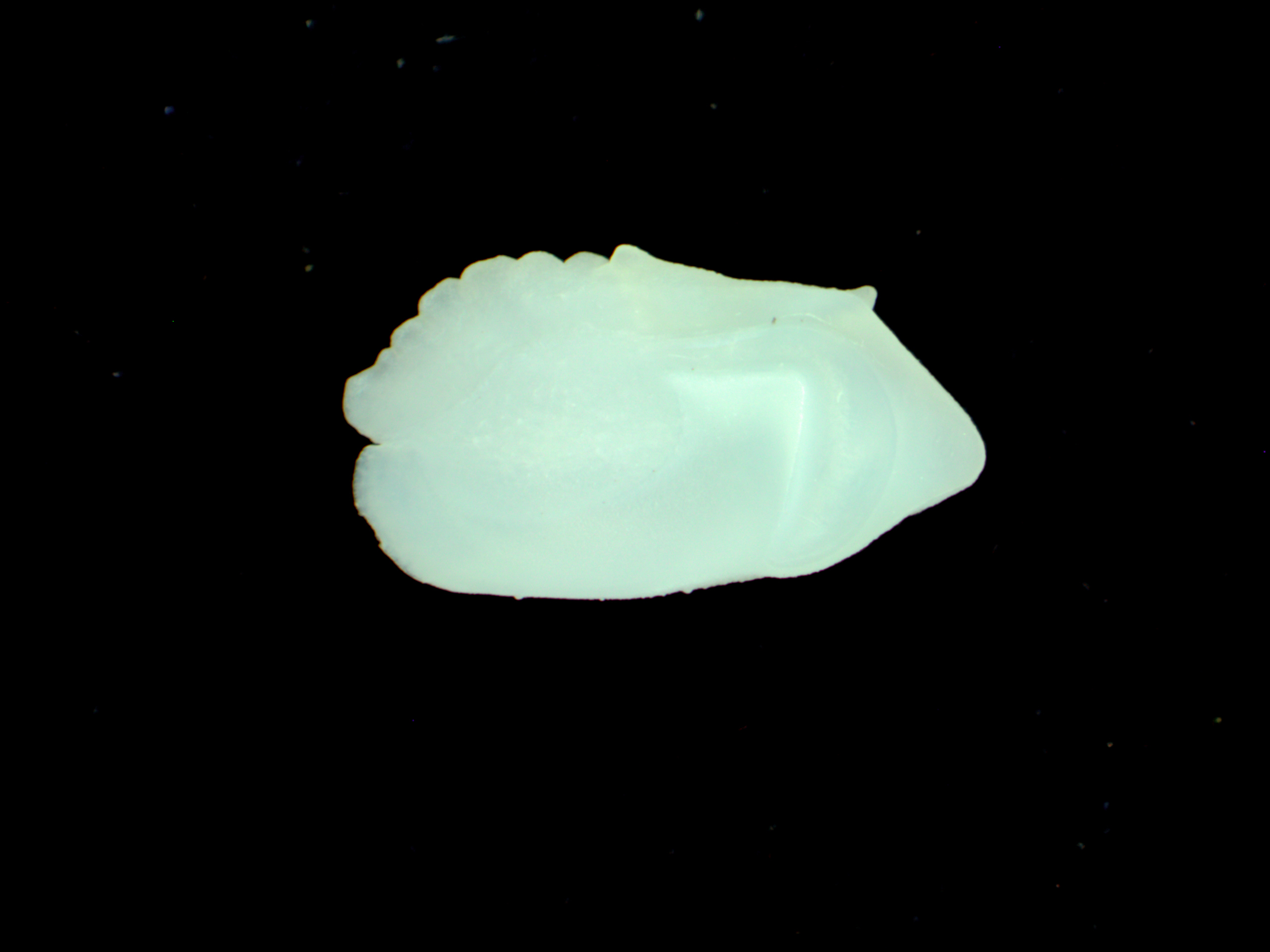

Supplement: Supplemental Information 15 [file peerj-04-1664-s015.zip › PanMic/training/540R1.jpg]

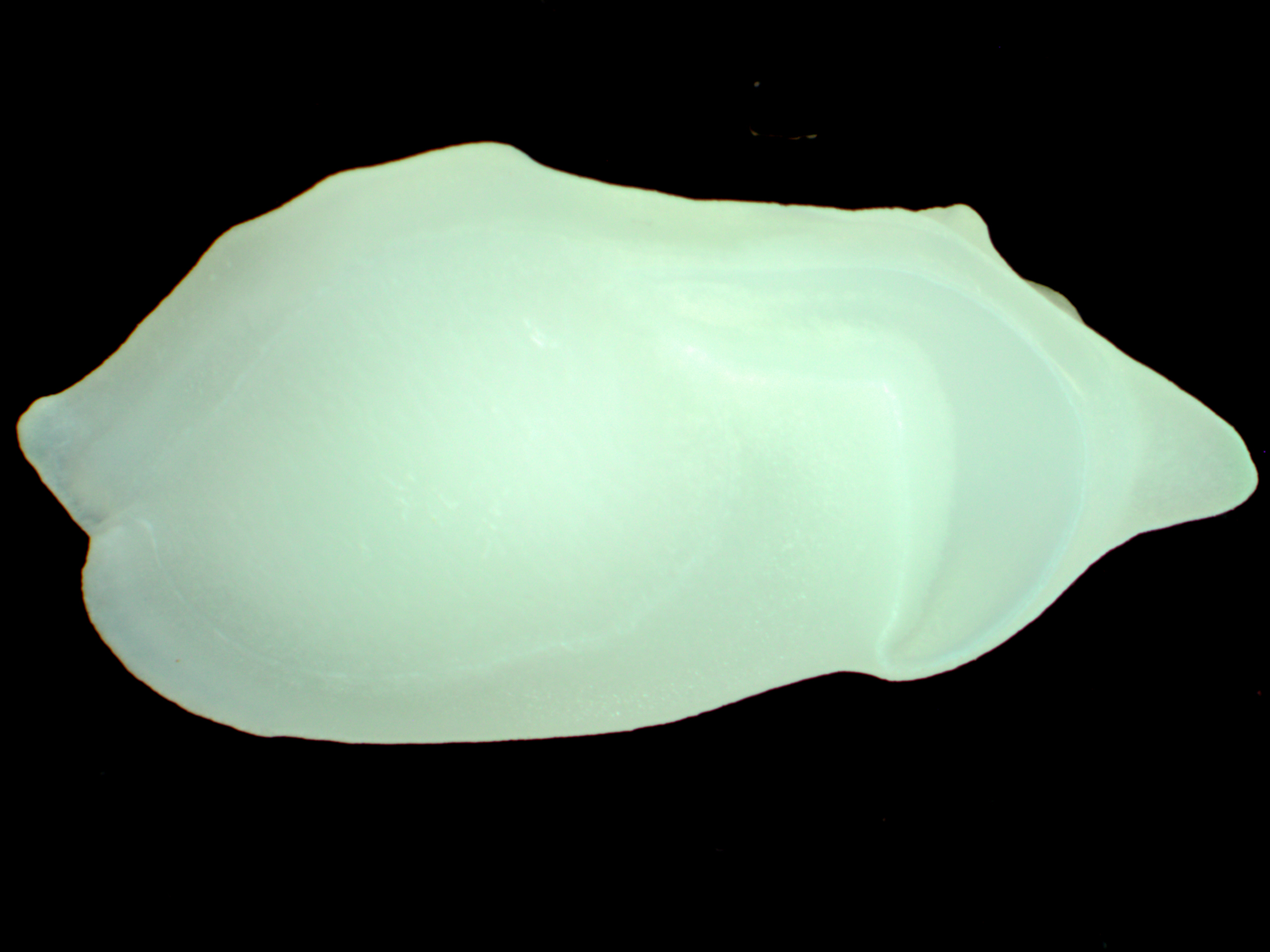

Supplement: Supplemental Information 15 [file peerj-04-1664-s015.zip › PanMic/training/543R1.jpg]

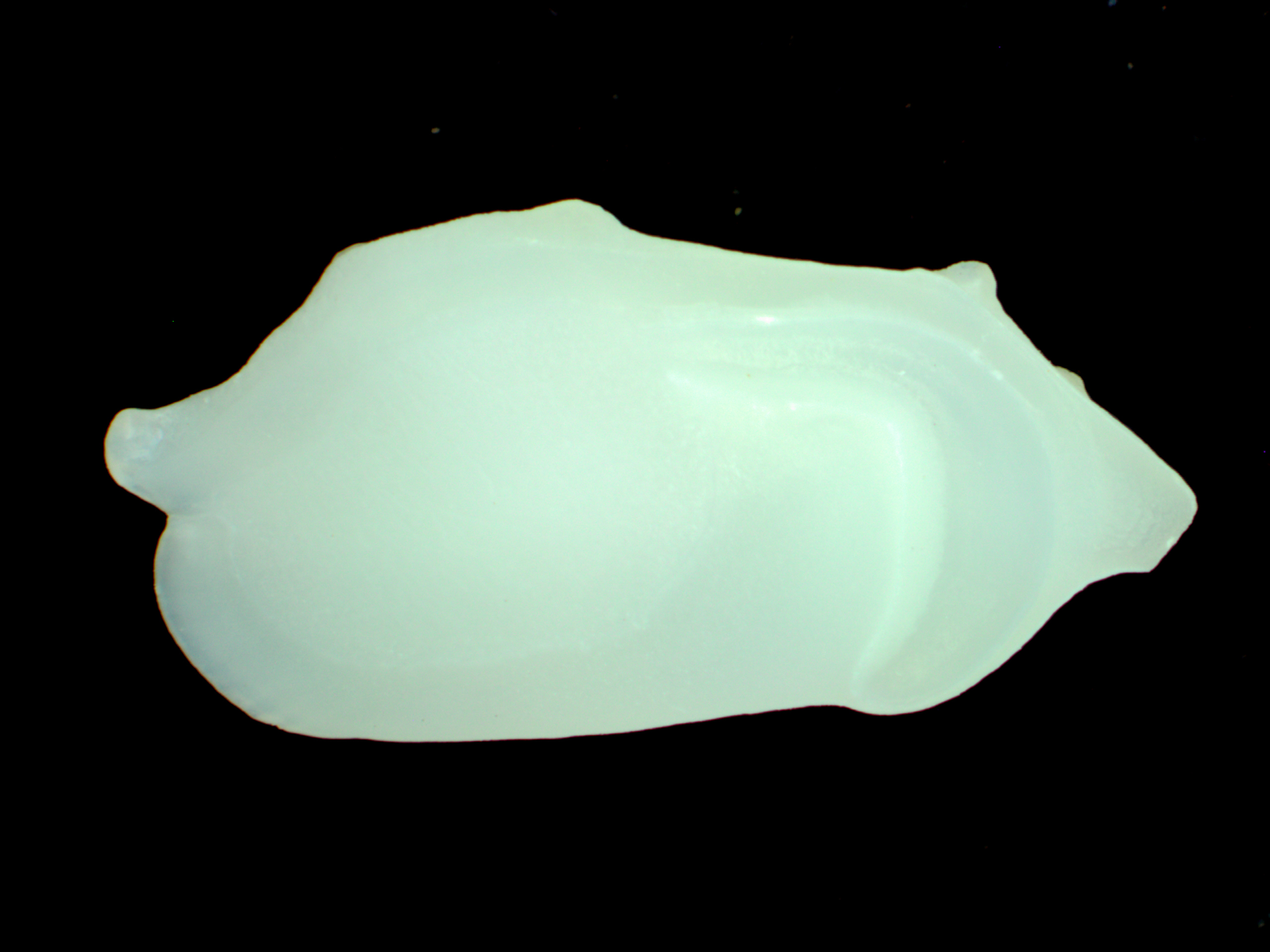

Supplement: Supplemental Information 15 [file peerj-04-1664-s015.zip › PanMic/training/544R1.jpg]

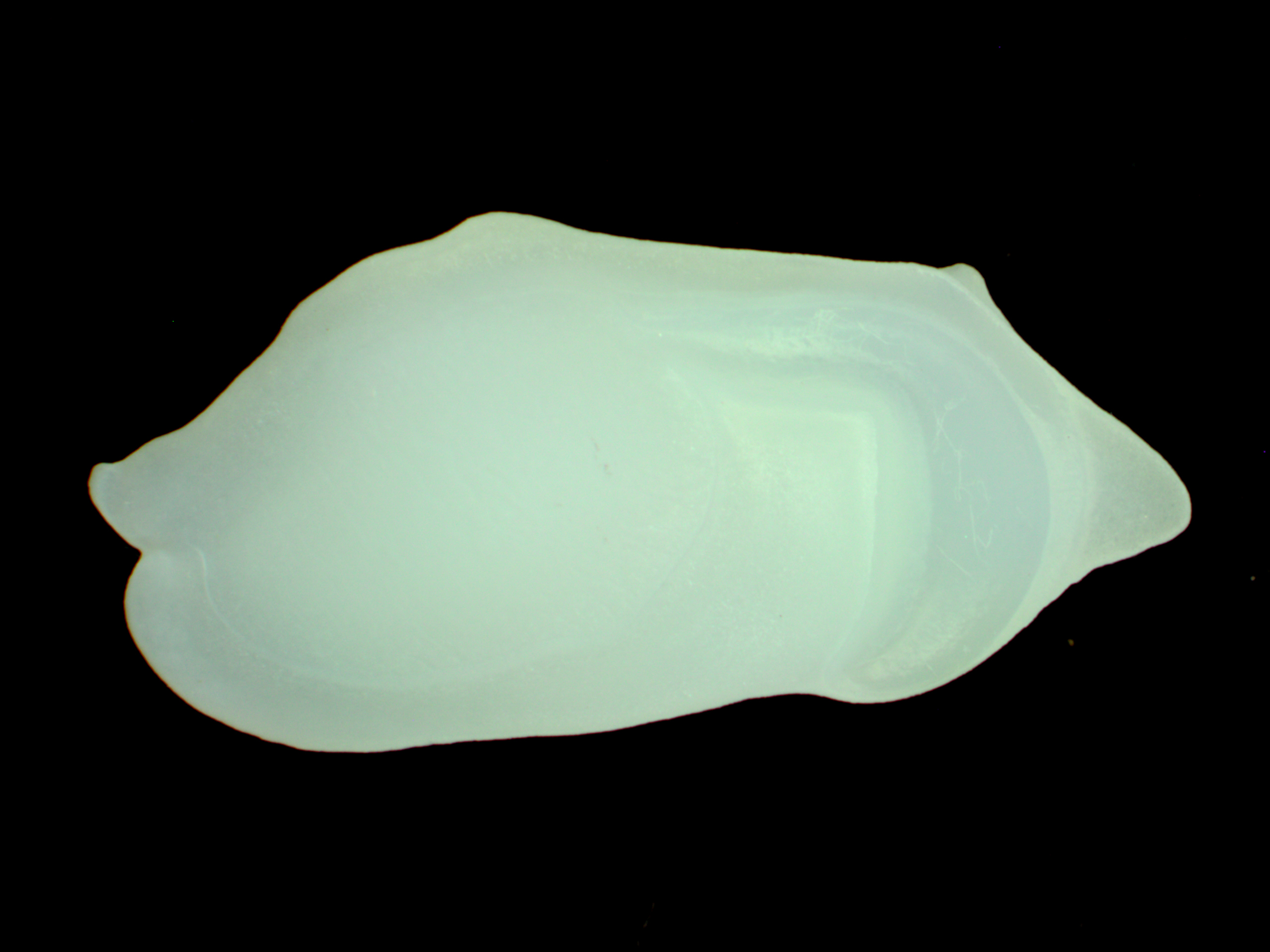

Supplement: Supplemental Information 15 [file peerj-04-1664-s015.zip › PanMic/training/545R1.jpg]

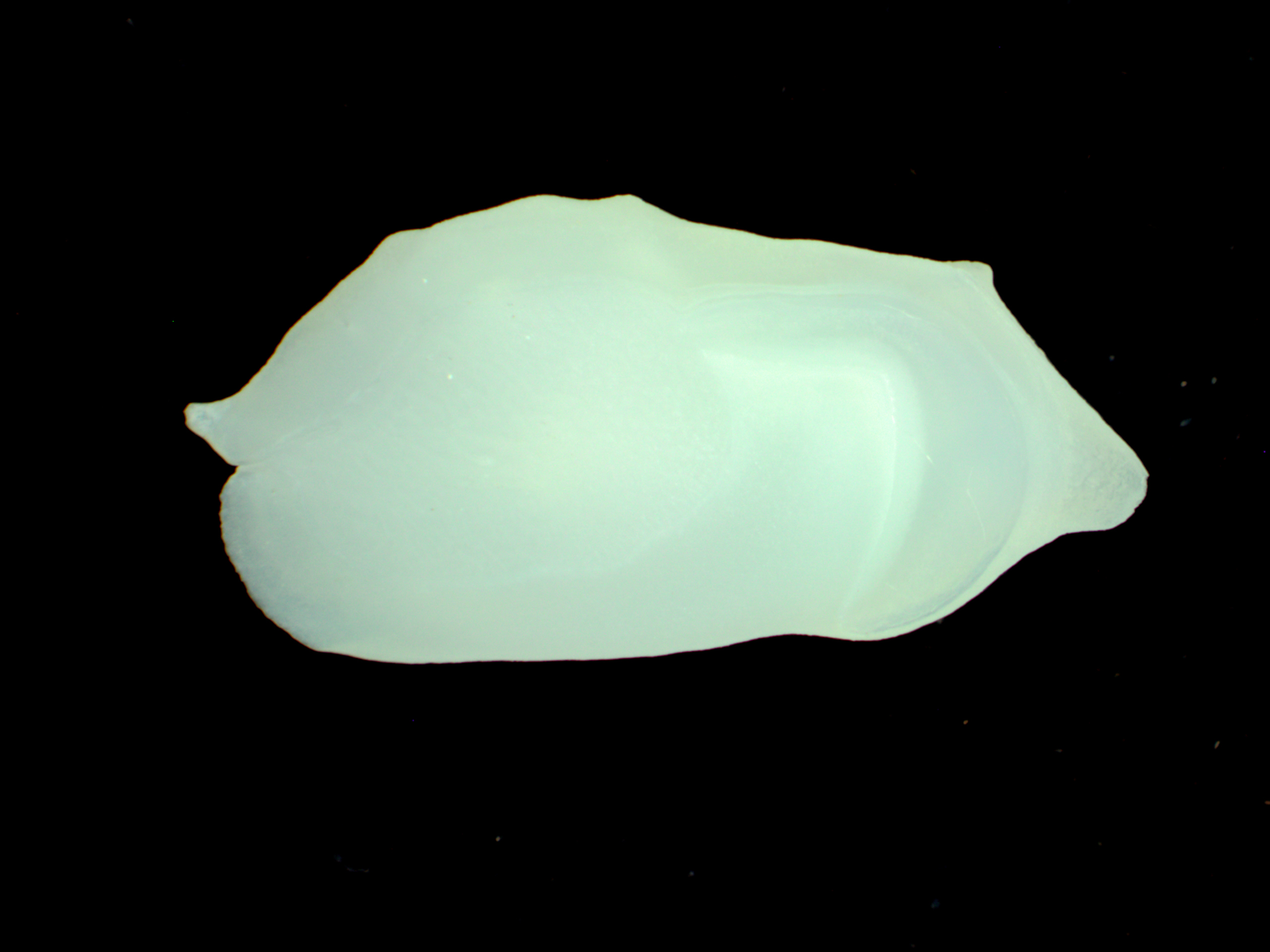

Supplement: Supplemental Information 15 [file peerj-04-1664-s015.zip › PanMic/training/54R1.jpg]

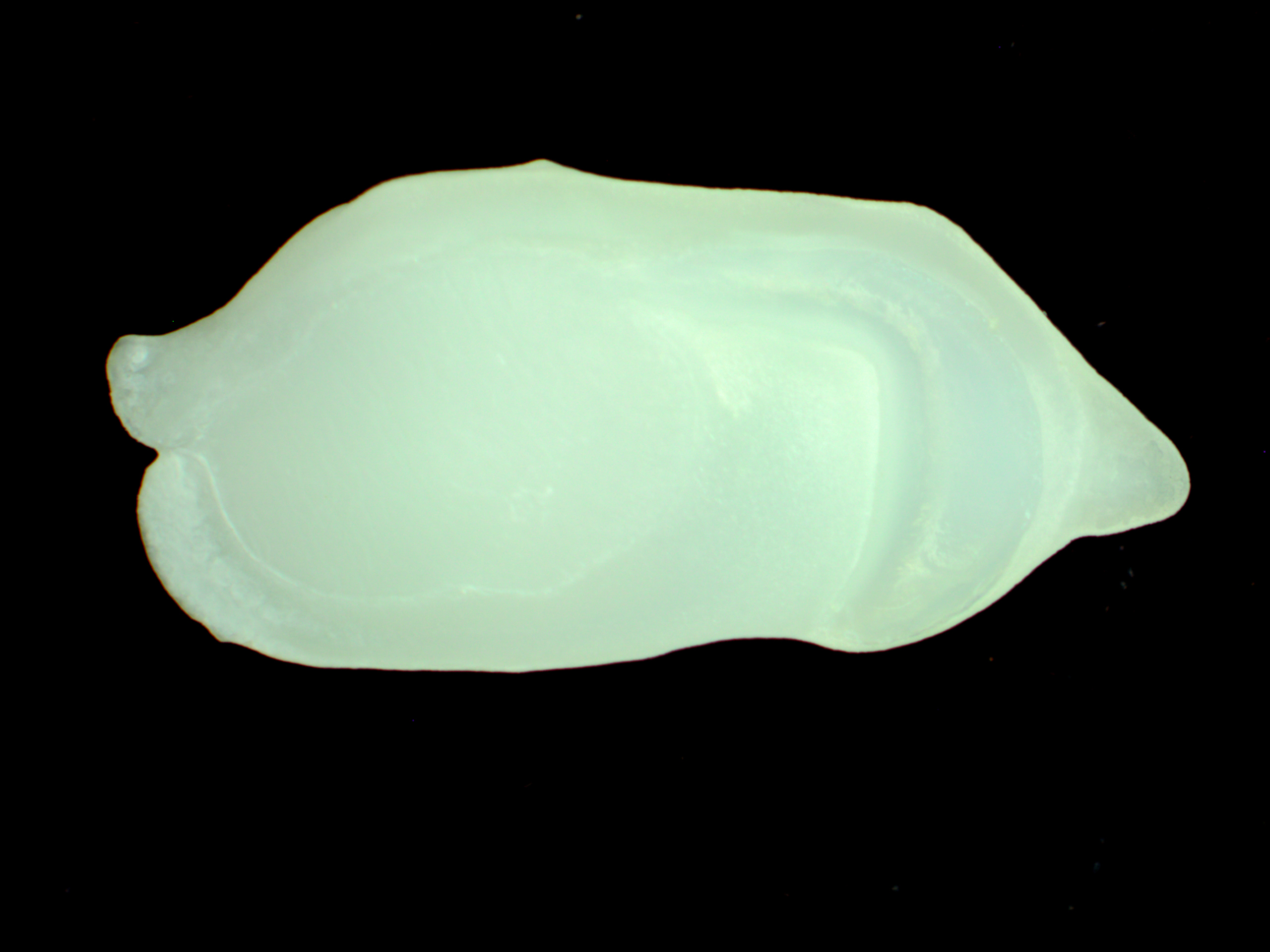

Supplement: Supplemental Information 15 [file peerj-04-1664-s015.zip › PanMic/training/55R1.jpg]

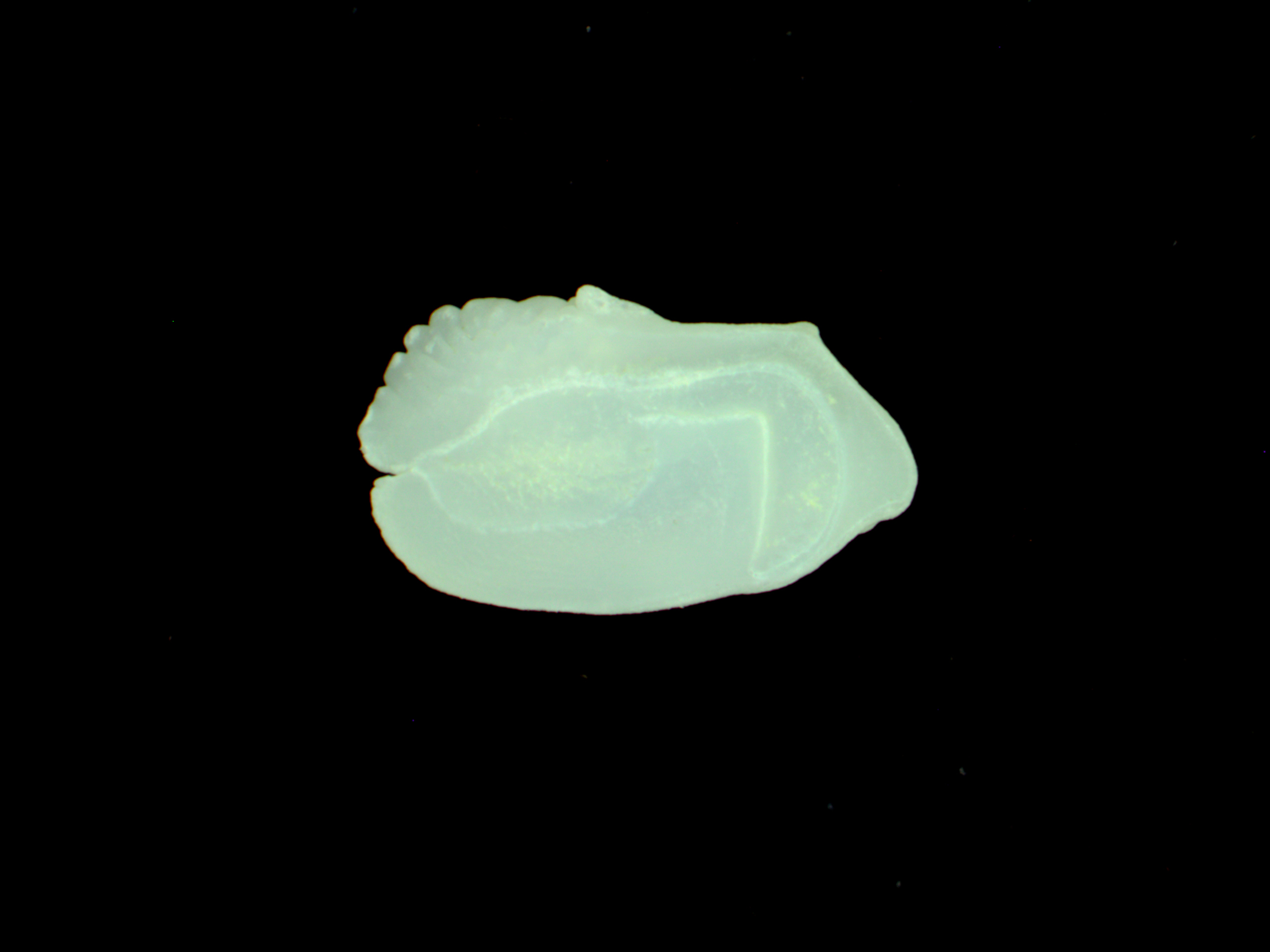

Supplement: Supplemental Information 15 [file peerj-04-1664-s015.zip › PanMic/training/B78R1.jpg]

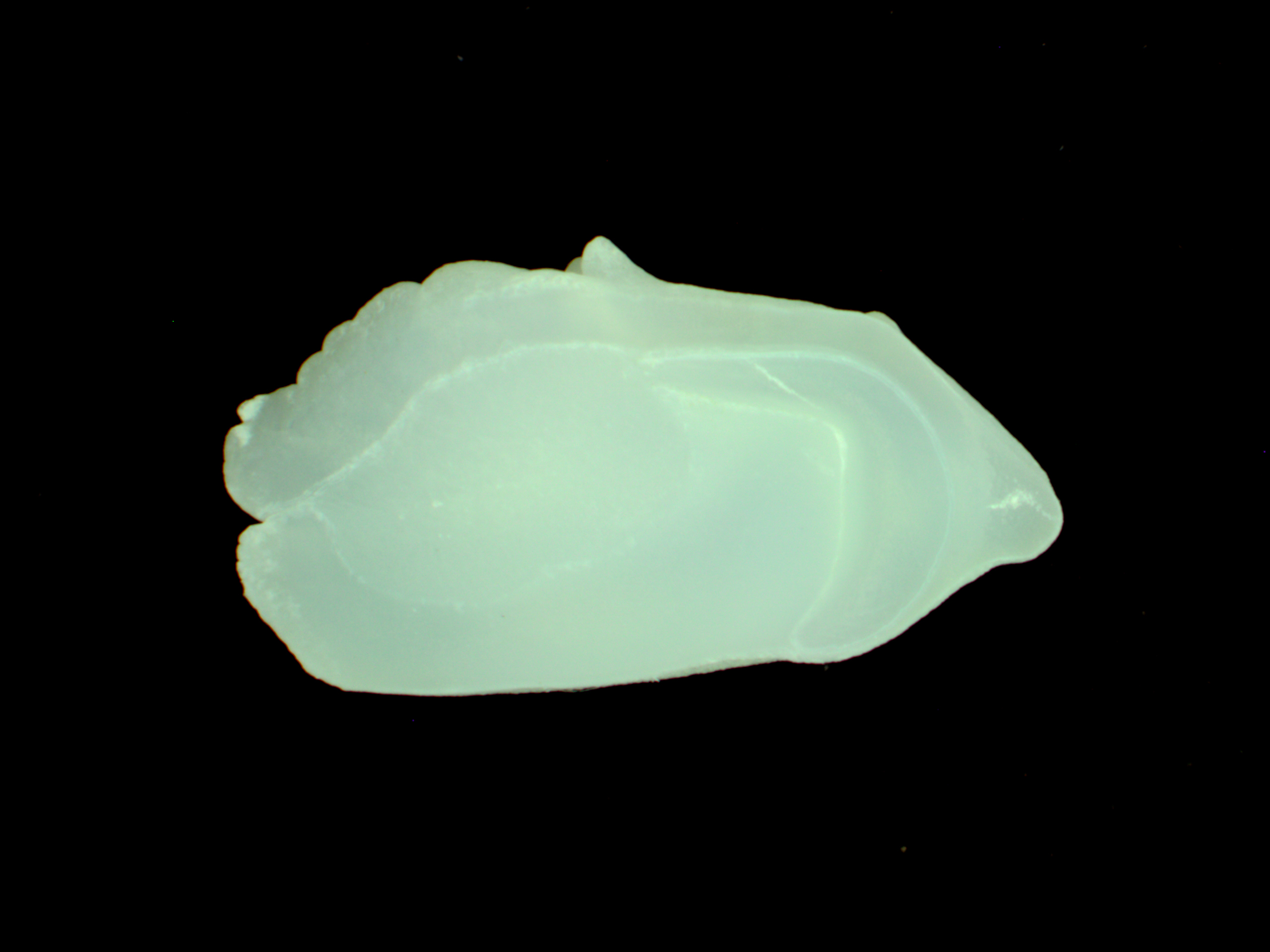

Supplement: Supplemental Information 15 [file peerj-04-1664-s015.zip › PanMic/training/B96R1.jpg]

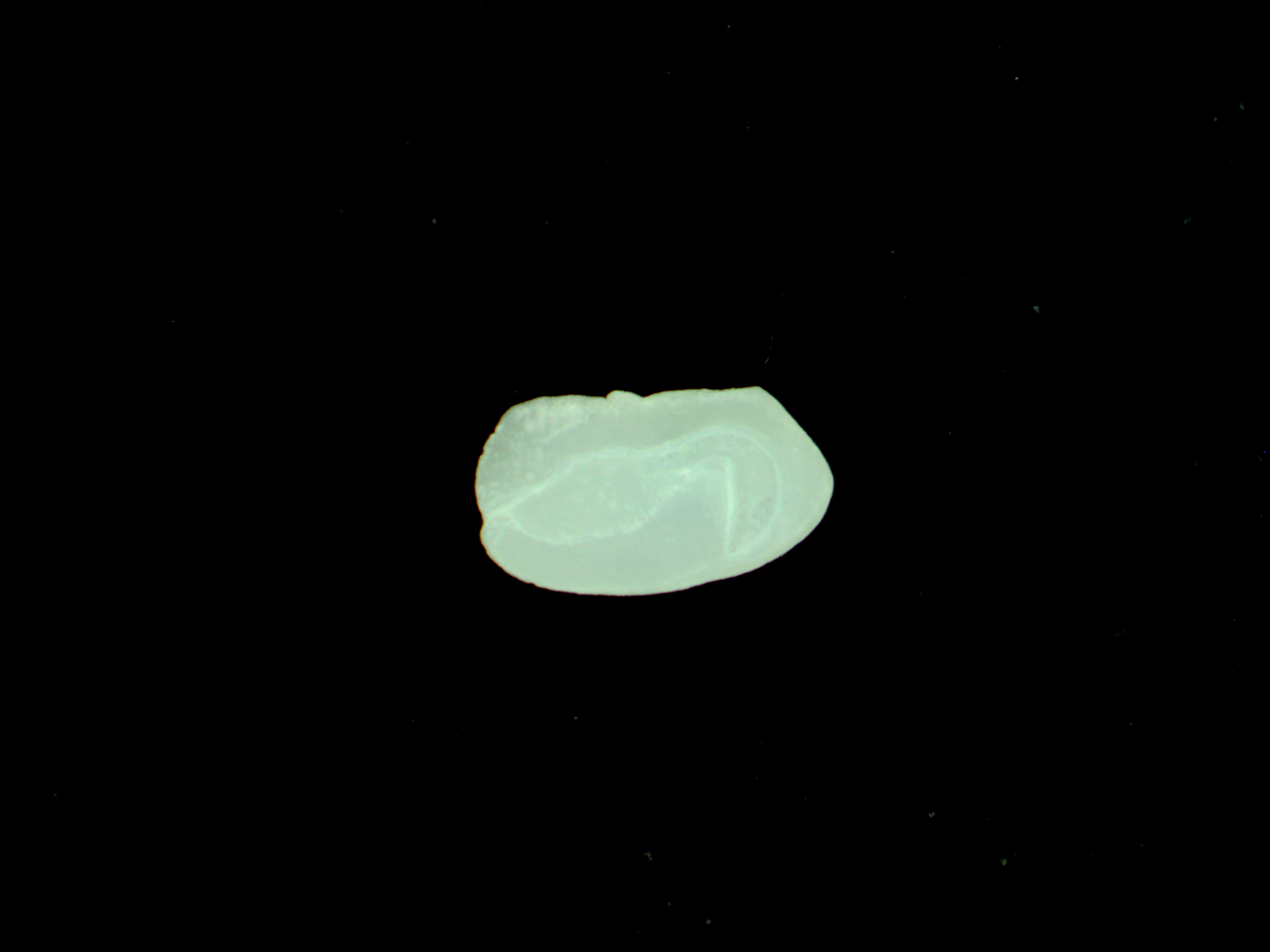

Supplement: Supplemental Information 15 [file peerj-04-1664-s015.zip › PanMic/training/D81R1.jpg]

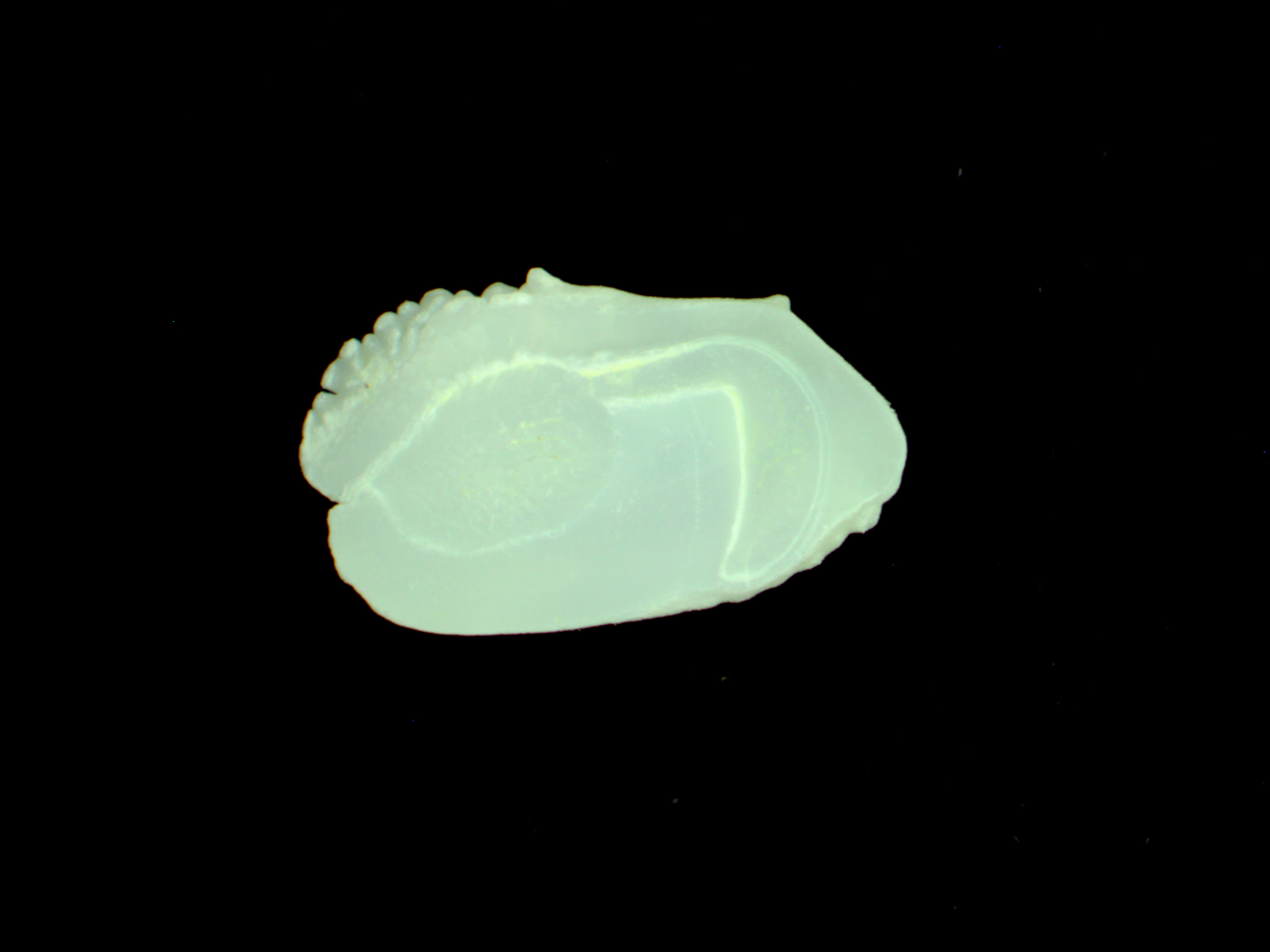

Supplement: Supplemental Information 15 [file peerj-04-1664-s015.zip › PanMic/training/E74R1.jpg]
