# Supplementary figures and images for: Fully-automated identification of fish species based on otolith contour: using short-time Fourier transform and discriminant analysis (STFT-DA) (part 5 of 5)
Source: PeerJ. 2016 Feb 22;4:e1664. doi: 10.7717/peerj.1664 (PMC4768690; doi:10.7717/peerj.1664)

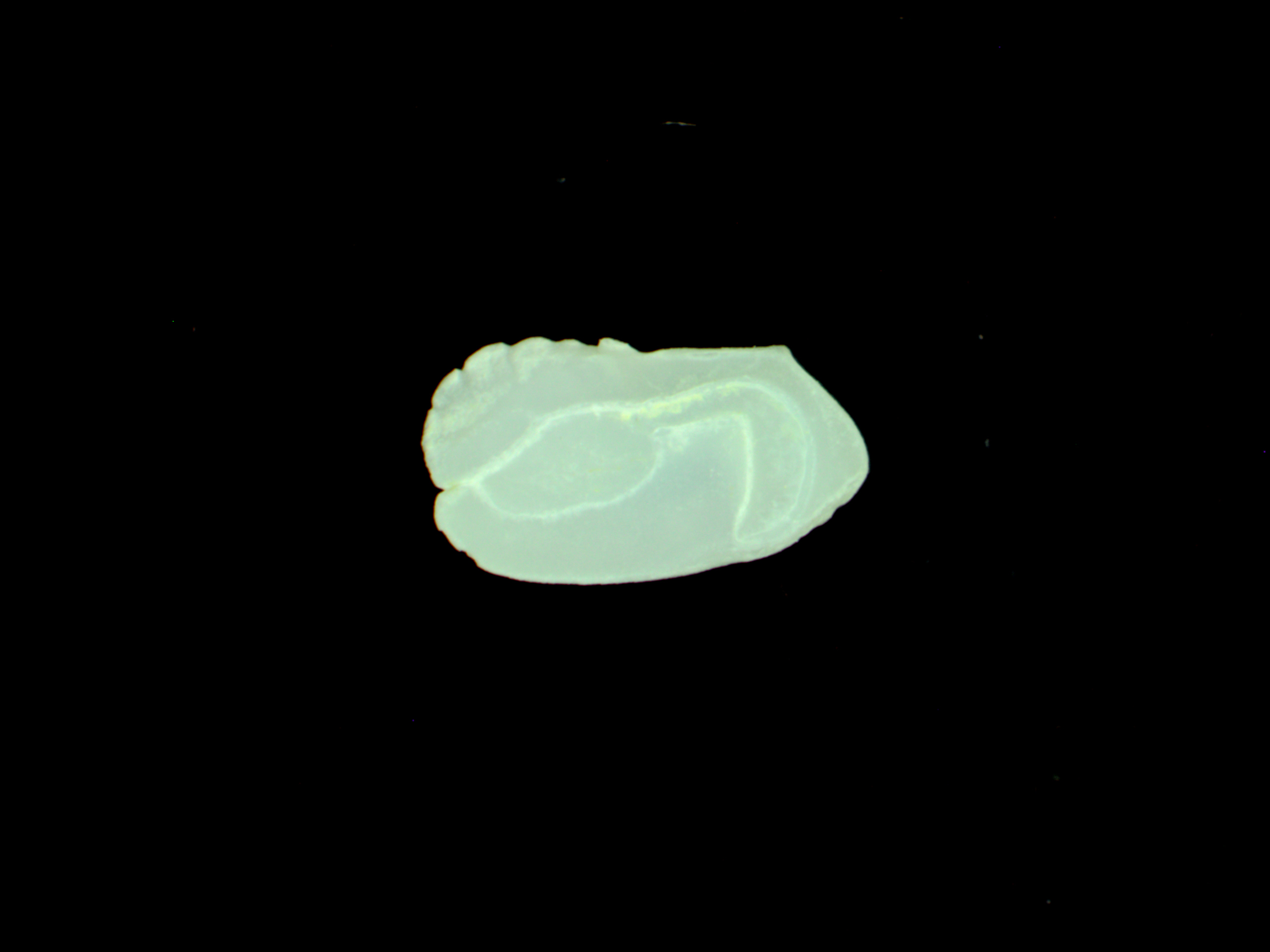

Supplement: Supplemental Information 15 [file peerj-04-1664-s015.zip › PanMic/training/E93R1.jpg]

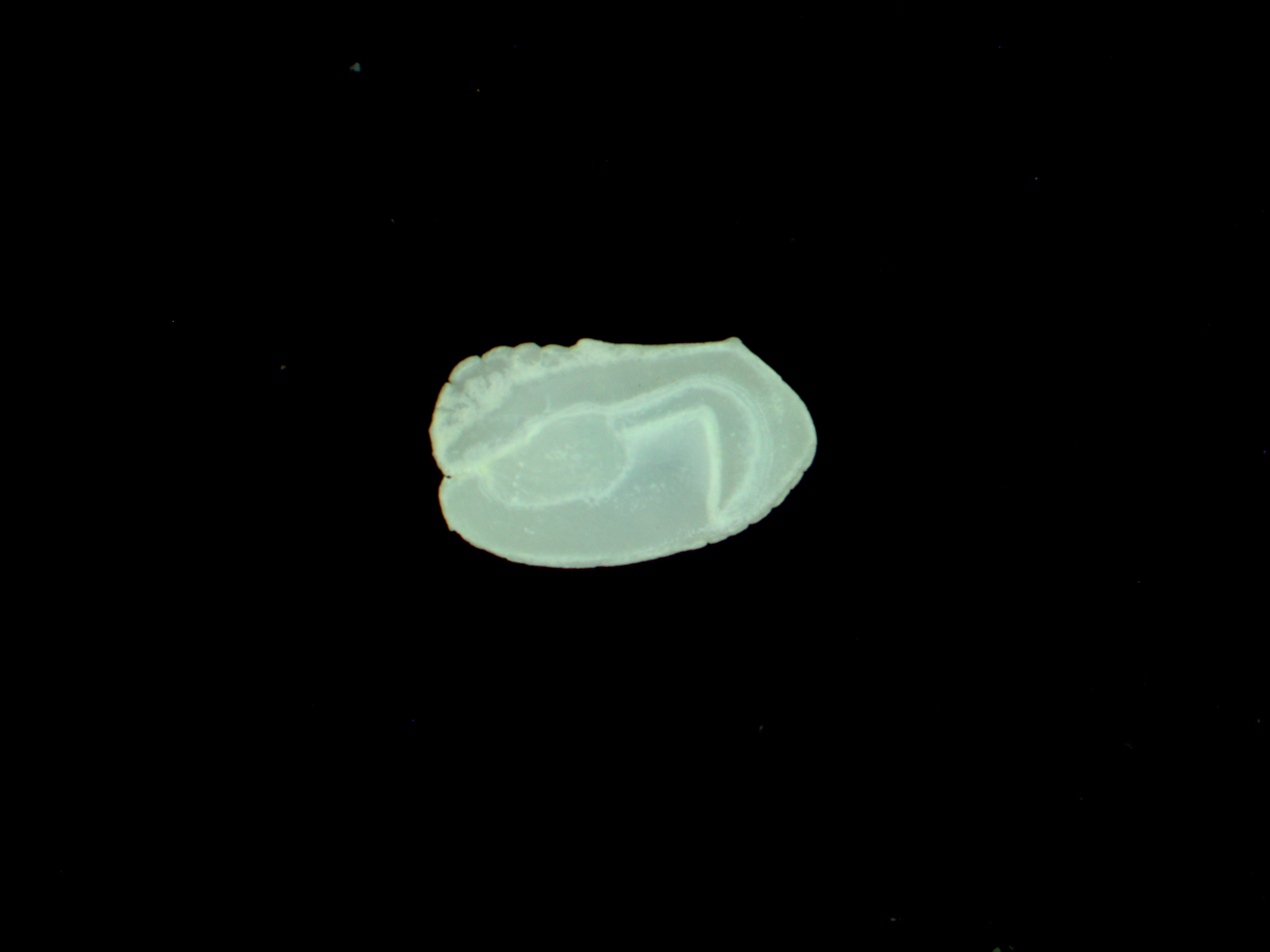

Supplement: Supplemental Information 15 [file peerj-04-1664-s015.zip › PanMic/training/F41R1.jpg]

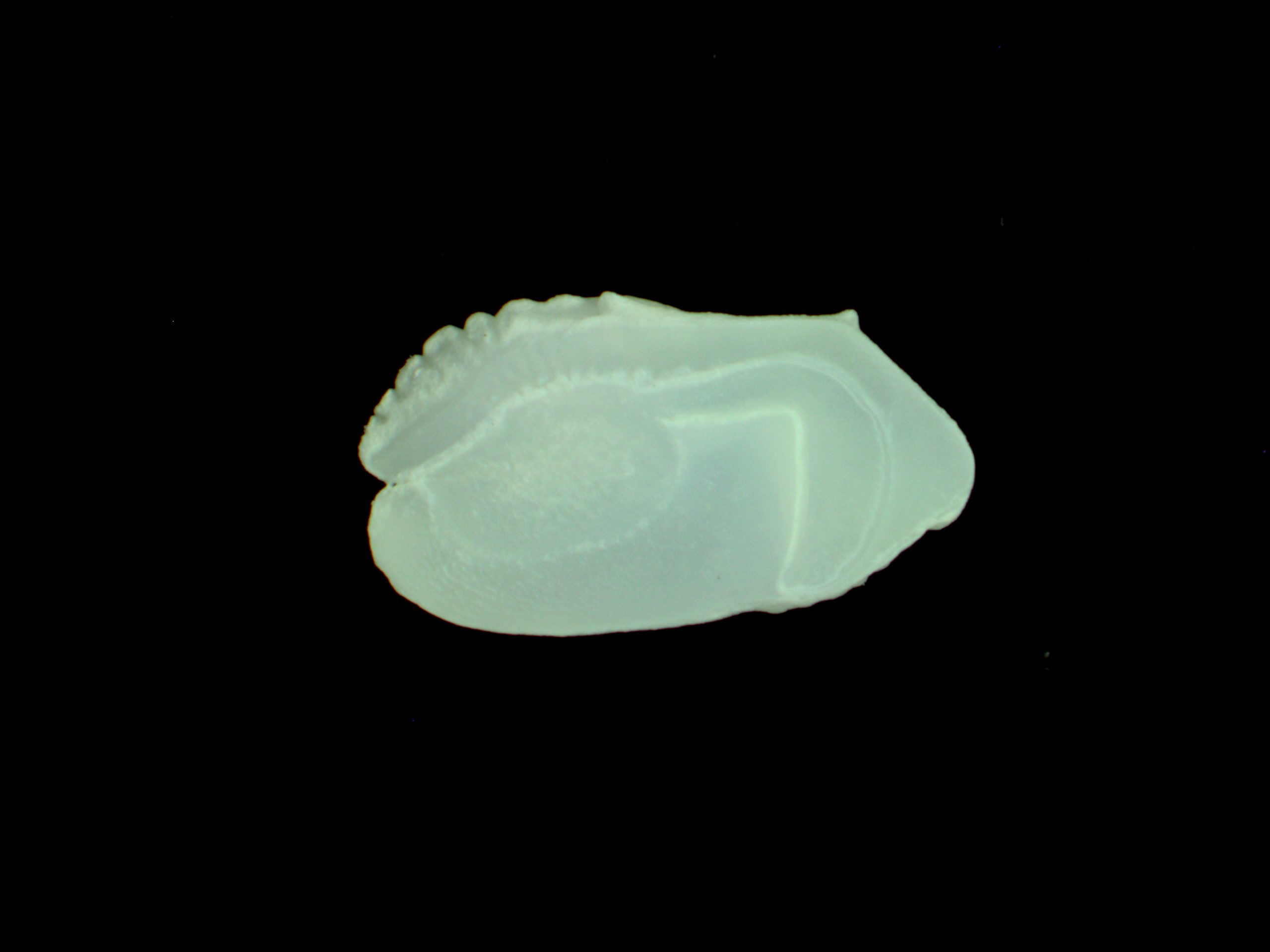

Supplement: Supplemental Information 15 [file peerj-04-1664-s015.zip › PanMic/training/F75R1.jpg]
